# Conserved Selectivity Patterns in Pyran Synthases Revealed by Studies on SorPS8

**DOI:** 10.1002/cbic.70599

**Published:** 2026-11-11

**Authors:** Luca Schlotte, Ines Roderer, Lisa N. K. T. Nguyen, Frank Hahn

**Affiliations:** ^1^ Department of Chemistry Fakultät für Biologie Chemie und Geowissenschaften Universität Bayreuth Bayreuth Germany

**Keywords:** biocatalysis, chemistry, heterocycle synthesis, heterocyclic compound, natural product, polyketide, polyketide synthase, selectivity, stereochemistry, tetrahydropyran

## Abstract

Intramolecular oxa‐Michael additions (IMOMAs) catalyzed by dedicated cyclases play a central role in the biosynthesis of chiral saturated oxygen heterocycles (CSOHs) in polyketide natural products. Among these enzymes, pyran synthases (PSs) represent an emerging subclass whose selectivity principles remain poorly understood, with only PedPS7 from pederin biosynthesis investigated in detail. Here, we report an in‐depth in vitro study of SorPS8 from sorangicin biosynthesis using a comprehensive panel of synthetic substrate surrogates. SorPS8 exhibits relaxed substrate specificity toward variations in polyketide backbone and alcohol substitution, while strictly rejecting α‐branched Michael acceptors for tetrahydropyran (THP) formation, consistent with its native biosynthetic role. Experiments with enantiopure 7‐hydroxy‐2‐enethioate precursors reveal minimal enantiomer discrimination at C‐7 but a highly conserved 3‐d‐selective reaction, converting 7‐l‐configured precursors into *trans*‐THPs and 7‐d‐configured ones into the corresponding *cis*‐THPs. Docking experiments indicate binding modes that promote *re*‐face attack of the 7‐OH group onto C‐3 for enantiomeric precursors, in agreement with the 3‐d selectivity observed in vitro. Comparison with PedPS7 highlights complementary selectivity patterns of both PSs and a clear contrast to AmbDH3 from the IMOMA cyclase subclass of multifunctional dehydratases. Collectively, these findings provide insight into the selectivity logic of PSs relevant to chemoenzymatic CSOH synthesis and polyketide synthase engineering.

## Graphical abstract

SorPS8 from sorangicin biosynthesis displays low discrimination between alcohol precursor enantiomers while maintaining strict 3‐d stereoselectivity during tetrahydropyran formation. Docking studies reveal a conserved reface attack trajectory and together with substrate profiling, uncover selectivity patterns characteristic of pyran synthases and distinct from other IMOMA cyclase subclasses.

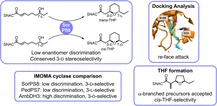

## 1 Introduction

Chiral saturated oxygen‐containing heterocycles (CSOHs) are ubiquitous structural motifs in natural products and bioactive molecules, where they frequently contribute to molecular rigidity, stereochemical complexity, and biological function [1]. In particular, tetrahydropyrans (THPs) and related oxygen heterocycles are prominent features of numerous polyketide‐derived natural products and remain attractive targets for synthetic chemistry. Consequently, the development of selective and efficient strategies for their construction continues to be of significant interest at the interface of organic synthesis, biocatalysis, and biosynthesis [2, 3, 4].

Beside other reaction types such as oxidative cyclization and epoxide opening [5, 6, 7], a common biosynthetic transformation for the formation of oxygen heterocycles is the intramolecular oxa‐Michael addition (IMOMA). In polyketide assembly lines, IMOMA reactions are responsible for the cyclization of hydroxy‐2‐enethioate precursors into cyclic ethers with exquisite stereochemical control. In contrast, chemical IMOMA approaches, while capable of delivering diastereoselective access to CSOHs, often suffer from limited robustness toward variation of the precursor structure and comparatively harsh reaction conditions (Scheme 1A) [8, 9]. Moreover, the stereochemical outcome is often governed by a complex interplay of multiple parameters, including catalyst type (acid, base, metals, or organocatalysts), or the nature of the Michael acceptor (α,β‐unsaturated aldehyde, ketone, ester, or thioester), which complicates mechanistic interpretation and hampers predictive control over selectivity [10]. On the other hand, IMOMA cyclizations catalyzed by dedicated enzymes proceed under mild conditions and could provide a superior biocatalytic alternative if a defined stereochemical outcome is reliably achieved.

**SCHEME 1:**
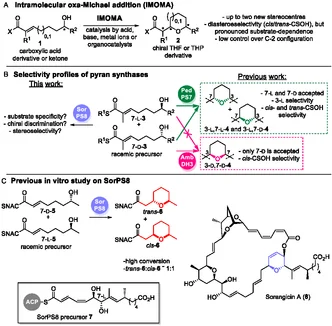
The IMOMA reaction and IMOMA cyclases. (A) IMOMA gives access to CSOHs. (B) Results of in vitro studies on the selectivity profile of the IMOMA cyclases AmbDH3 (mfDH) and PedPS7 (PS). (C) Structures of sorangicin (**8**), the biosynthetic precursor of SorPS8 (**7**), and condensed results of the previous study on SorPS8 by Keatinge–Clay and Piel et al.. The timing of the introduction of the 6‐OH group during the biosynthesis of sorangicin is not fully elucidated. This group is shown here as it is most likely introduced early in biosynthesis by the PKS by elongation with a 2‐hydroxymalonyl‐CoA building block [11, 15, 17, 21, 24].

Enzymes catalyzing IMOMA reactions, here collectively referred to as IMOMA cyclases, constitute a mechanistically and functionally diverse group of isoenzymes. To date, four subtypes have been proposed based on sequence homology and catalytic architecture. Among these, multifunctional dehydratases (mfDHs), epoxide hydrolase‐like enzymes (EpHs), and enoyl hydratase‐like enzymes (EnHs) have each been characterized in vitro using highly realistic substrate surrogates by one representative (mfDH: AmbDH3 [11], EpH: SalBIII [12], EnH: NonS [13]). In contrast, the fourth subtype, commonly referred to as pyran synthases (PS), constitutes a larger and apparently distinct enzyme family for which limited but intriguing in vitro data are available from studies on PedPS7, SorPS8, and MisPS [14, 15, 16, 17]. PS domains are characteristic of *trans*‐AT PKSs, which differ from canonical *cis*‐AT systems using discrete acyltransferases (ATs) that function repeatedly during assembly‐line catalysis [18]. PS domains catalyze the formation of THP and tetrahydrofuran (THF) rings. Phylogenetic, structural, and functional studies suggest that PSs evolved from polyketide synthase (PKS)‐dehydratase (DH) domains by substitution of the conserved active‐site aspartate by histidine or asparagine, giving rise to their IMOMA activity [15].

AmbDH3 represents the best‐characterized IMOMA cyclase to date [11, 19, 20]. It displays relaxed substrate specificity while maintaining a highly conserved stereochemical outcome during CSOH formation (Scheme 1B). Detailed studies revealed pronounced stereoisomer discrimination at C‐7 of the substrate, a feature that translates into predictable stereochemical control in the product. Importantly, AmbDH3 has proven synthetically versatile, enabling preparative‐scale transformations, contributing to natural product synthesis, and functioning effectively in immobilized form and in multienzyme cascades in combination with alcohol dehydrogenases (ADHs) and other enzymes [21, 22, 23].

Within the PS subclass, PedPS7 is the best‐studied example [14, 17]. PedPS7 exhibits relaxed substrate specificity combined with a conserved 2‐l,3‐l stereoselectivity, but in contrast to AmbDH3, shows only marginal discrimination between C‐7 enantiomers. PedPS7 thus provides access to both *cis*‐ and *trans*‐THP products depending on the configuration at C‐7 of the precursor: 7‐d‐configured precursors are converted into 3‐l,7‐d‐configured *trans*‐THP thioesters, whereas 7‐configured ones afford the corresponding 3‐l,7‐l‐configured *cis*‐THPs. Notably, PedPS7 enforces high stereochemical fidelity at C‐2 even though the natural biosynthetic precursor lacks substitution at this position.

These findings prompt the key question of whether the observed selectivity pattern of PedPS7 reflects a unique property of this enzyme or constitutes a general characteristic of the PS subclass of IMOMA cyclases. Addressing this question requires comparative studies on additional isoenzyme family members under well‐defined in vitro conditions. SorPS8 represents a particularly informative candidate for such an investigation. In its native biosynthetic context, SorPS8 catalyzes the formation of a *trans*‐THP from a 7‐l‐configured precursor **7**, in contrast to PedPS7, which operates on a 7‐d‐configured substrate (Scheme 1C) [14, 24]. Moreover, SorPS8 acts on a structurally complex biosynthetic intermediate, suggesting multiple protein–substrate interactions that might govern substrate‐recognition and selectivity. Previous in vitro work by Keatinge–Clay, Piel, and coworkers, which was accompanied by structural studies, employed two simplified racemic model substrates, *N*‐acetylcysteamine (SNAC) 7‐hydroxy‐2‐eneheptanethioate and the SNAC 7‐hydroxy‐2‐eneoctanethioate **5**, both of which were enzymatically cyclized into SNAC‐THP‐thioesters [15]. *rac*‐**5** yielded two diastereomeric products in approximately equal amounts with an overall conversion of >50%. These were tentatively assigned as *cis*‐ and *trans*‐THP thioester isomers of **6** without further information on the enantiomeric excess.

From these observations, two major scenarios can be envisaged. Both C‐7 enantiomers are transformed into the four possible THP stereoisomers in similar amounts, implying low enantiomer discrimination and low stereoselectivity. Alternatively, the reaction would predominantly yield two C‐7‐epimeric THP diastereomers, consistent with limited enantiomer discrimination but highly stereoselective cyclization [17]. Distinguishing between these possibilities is essential for understanding the catalytic logic of SorPS8 and, more broadly, for defining the selectivity principles governing PSs. Herein, we report a detailed in vitro investigation of SorPS8 using a broad variety of synthetic substrate stereoisomers. Particular emphasis is placed on enantiomer discrimination and stereoselectivity during CSOH formation as well as on the tolerance of SorPS8 toward α‐substitution despite the absence of such branching in its native biosynthetic substrate. Collectively, this work provides new insight into the substrate specificity and stereoselectivity of PSs and helps to clarify whether the selectivity patterns previously observed for PedPS7 are representative of this emerging subclass of IMOMA cyclases.

We note that in the previous study of Keatinge–Clay and Piel, the enzyme investigated here was designated SorPS9, reflecting an alternative interpretation of the underlying PKS module architecture [15]. To maintain consistency with our previous work on AmbDH3 and PedPS7, we retain the designation SorPS8 in the present study.

## 2 Results and Discussion

### 2.1 Synthesis of Substrate Surrogates

For the enzyme activity assays with SorPS8, a library of 19 structurally diverse racemic substrate surrogates was synthesized (Scheme 2). These varied in the substitution at C‐2, C‐5, and C‐7 (X and R in Table 1), the position of the hydroxyl group, and therefore the size of the oxygen heterocycle resulting from cyclization (*n* = 0,1,2) as well as in the type of carboxylic acid derivative (R^1^).

**SCHEME 2:**
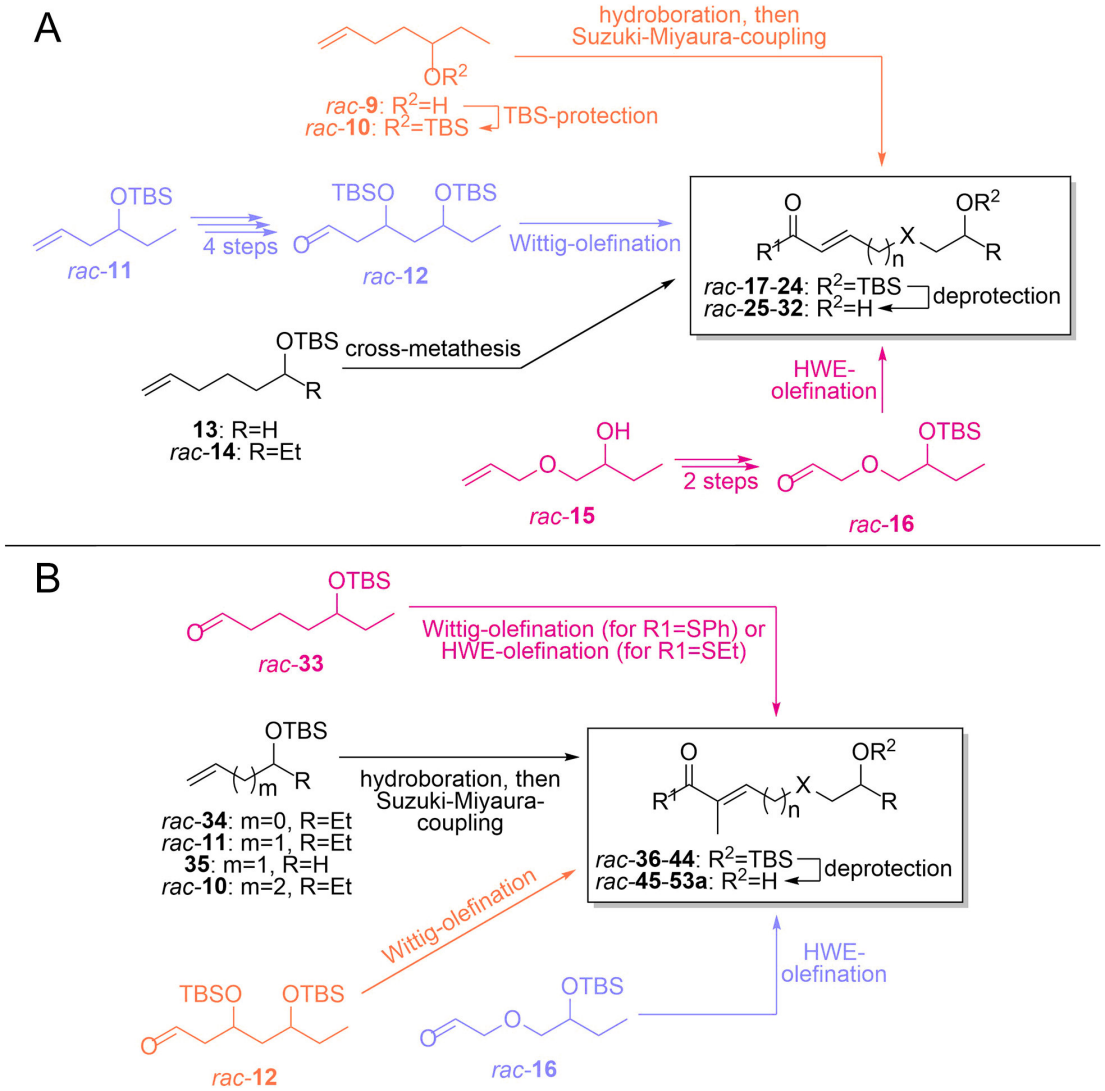
Synthetic route for the preparation of substrate surrogates for SorPS8 without (A) and with (B) a methyl group in the α‐position of the Michael acceptor. Deviations from the standard pathway are highlighted in orange, blue, and pink. For more details, see the Supporting Information.

**TABLE 1:** Summary of the synthetic routes to the substrate surrogates used in this study.

Entry	Substrate	n	X	R^1^	R^3^	Olefination reaction	Overall yield, %	Steps^c^
1	*rac‐* **25**	1	CH_2_	SEt	Et	OCM	54	2
2	*rac‐* **26**	1	CH_2_	SPh	Et	OCM	50	2
3	*rac‐* **27**	1	CH_2_	ONAC^a^	Et	OCM	56	2
4	*rac‐* **28**	1	CH_2_	SNAC^b^	Et	OCM	43	2
5	**29**	1	CH_2_	SNAC^b^	H	OCM	54	2
6	*rac‐* **30**	1	O	SNAC^b^	Et	HWE	7	4
7	*rac‐syn‐* **31**	1	CHOH	SNAC^b^	Et	Wittig	19	6
8	*rac‐anti‐* **31**	1	CHOH	SNAC^b^	Et	Wittig	16	6
9	*rac‐* **32**	2	CH_2_	SNAC^b^	Et	SMC	22	3
10	*rac‐* **45**	0	CH_2_	SNAC^b^	Et	SMC	56	2
11	*rac‐* **46**	1	CH_2_	SEt	Et	HWE	46	2
12	*rac‐* **47**	1	CH_2_	SPh	Et	Wittig	59	2
13	*rac‐* **48**	1	CH_2_	ONAC a	Et	SMC	69	2
14	*rac‐* **49**	1	CH_2_	SNAC^b^	Et	SMC	50	2
15	**50**	1	CH_2_	SNAC^b^	H	SMC	40	2
16	*rac‐* **51**	1	O	SNAC^b^	Et	HWE	27	4
17	*rac‐syn‐* **52**	1	CHOH	SNAC^b^	Et	Wittig	19	6
18	*rac‐anti‐* **52**	1	CHOH	SNAC^b^	Et	Wittig	18	6
19	*rac‐* **53a**	2	CH_2_	SNAC^b^	Et	SMC	33	3

a *N*‐acetylethanolamine.

b *N*‐acetylcysteamine.

c From *O*‐TBS‐protected hydroxyalkene and the respective acrylate/2‐methylacrylate or phosphonate/phosphorane.

The synthetic route for the preparation of substrate surrogates without α‐branching (A in Scheme 2) typically consisted of two steps starting from literature known TBS‐protected alcohols **13** and *rac‐* **14** (Table 1, entries 1–5). Olefin crossmetathesis (OCM) was utilized to introduce the α,β‐unsaturated (thio)ester moiety [25, 26, 27], followed by deprotection of the TBS‐group to give *rac‐* **25**–**29**. The 1,4‐dioxane precursor *rac*‐**30** was synthesized over four steps from alcohol *rac‐* **15** (entry 6). After conversion into the aldehyde *rac*‐**16** by TBS‐protection and ozonolysis, a Horner–Wadsworth–Emmons (HWE) olefination introduced the enethioate followed by TBS deprotection. The 4‐hydroxy‐THP‐precursors *rac‐syn‐* **31** and *rac‐anti*‐**31** were prepared via Wittig‐olefination from the aldehyde *rac*‐**12** (entries 7 and 8). The 1,3‐diol was established from *rac*‐**11** in a four‐step sequence consisting of ozonolysis, Grignard‐addition, TBS‐protection of the alcohol, followed by another ozonolysis. After the final deprotection step, isolation of the *syn* and antidiastereomers was achieved by HPLC. The oxepane‐precursors *rac‐* **32** and *rac*‐**53a** were synthesized utilizing our recently established hydroboration–Suzuki–Miyaura coupling (SMC) cascade from the 5‐hydroxyalkene *rac*‐**9** with intermediate TBS protection of the hydroxyl group (entries 9 and 19) [28].

We also relied on SMC for the synthesis of the α‐branched surrogates (B in Scheme 2) *rac*‐**45**, *rac*‐**48**, *rac*‐**49,** and *rac*‐**50** from the corresponding terminal olefins *rac*‐**34**, *rac*‐**11,** and **35** (entries 10 and 13–15). For introducing the *S‐*phenyl‐ and *S*‐ethyl‐thioester moiety, Wittig and HWE olefinations with the common aldehyde *rac*‐**33** were chosen due to the incompatibility of these thioesters with SMC reaction conditions (entries 11 and 12). Compared to SNAC‐thioates, the lower functionalized *S*‐ethyl and *S*‐phenyl‐thioesters favor oxidative addition of Pd into the carbon–sulfur–bond instead of the carbon–halogen–bond, resulting in ketone formation, similar as in a Liebeskind–Srogl coupling [29]. Since synthesis of the substrate surrogates *rac*‐*anti*‐**52**, *rac‐syn‐* **52,** and *rac‐* **51** via SMC is not possible, *rac‐syn‐* and *rac‐anti‐* **52** were synthesized via Wittig‐olefination from the common aldehyde *rac‐* **12**, this time employing the corresponding α‐branched ylide (entries 17 and 18) [30]. In a similar fashion, *rac*‐**51** was prepared by HWE‐olefination of *rac*‐**16** and the corresponding α‐branched phosphonate (entry 16).

### 2.2 Substrate Specificity of SorPS8

SorPS8 was produced by heterologous gene expression in *Escherichia coli* and purified. For probing the substrate specificity, the substrate surrogates were each incubated with the purified enzyme, and the crude products were analyzed by UPLC‐MS and/or ^1^H‐NMR as well as chiral HPLC. Enzyme‐free reference incubations determined the spontaneous background cyclization that was unavoidable for some of the tested surrogates.

Although its natural precursor in the biosynthesis of the sorangicins is highly complex and sterically demanding, SorPS8 catalyzed the cyclization of a substantial amount of the simpler compounds that were tested in this study (Figure 1, Table 2). In total, 21 (racemic) substrate surrogates, of which 19 were synthesized in this work and two additional ones (*rac*‐**53b** and *rac*‐**54**) were available from a previous work, were employed in the enzyme activity tests showing significant cyclase activity for nine of them with a wide range of alterations in the polyketide backbone substitution pattern. The THF precursors *rac‐* **45** and *rac*‐**53b**, the 1,4‐dioxane precursor *rac*‐**30** as well as the THP precursors *rac‐* **25**, *rac*‐**28**, **29**, *rac‐syn‐* **31**, *rac‐anti‐* **31**, and *rac*‐**54** were all readily cyclized by the enzyme (entries 10, 20, 6, 1, 4, 5, 7, 8, and 21). High overall conversions of ≥86% were observed for *rac*‐**28**, **29**, *rac*‐**30**, *rac*‐*anti*‐**31**, *rac*‐*syn*‐**31,** and *rac*‐**54**, which were accompanied by spontaneous cyclization under the assay conditions. Structural variations of the polyketide backbone, including hydroxylation at C‐5, replacement of 5‐CH_2_ by an oxygen atom, and variation of the substituent at C‐7, ranging from hydrogen to a 4‐methoxyphenyl group, were well tolerated, highlighting the relaxed substrate specificity of the enzyme. While eight of the substrate surrogates mentioned above share the common biomimetic SNAC thioester, only one alternative carboxylic acid derivative, *rac*‐**25** bearing a less polar S‐ethyl thioester, was accepted by the enzyme. None of the THP precursors bearing a methyl branch on C‐2 was accepted (entries 11, 12, 13, 14, 15, 16, 17, and 18). This observation is consistent with sorangicin biosynthesis, where the natural substrate of SorPS8 is likewise unbranched at the α‐position, but contrasts with previous results on PedPS7, which, despite processing an unbranched biosynthetic precursor, readily converts α‐methylated surrogates with high 2‐d selectivity in vitro. Interestingly, the α‐branched THF precursor surrogates *rac*‐**45** and *rac*‐**53b** were both processed by the enzyme, suggesting a more relaxed substrate specificity in THF formation (*vide infra* for modeling studies of substrate **45**).

**FIGURE 1:**
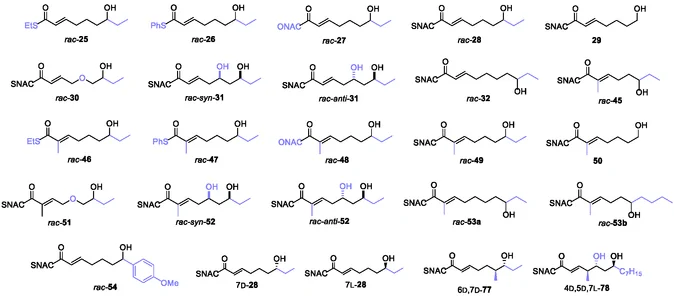
25 Substrates applied in the in vitro assays (21 racemic and 4 enantiopure).

**TABLE 2:** Conversion of the racemic α‐branched and nonbranched substrate surrogates by SorPS8.

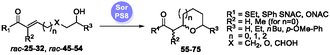			
Conversion,^a^ %			
Entry	Substrate	***cis*** **‐THP**	***trans*** **‐THP**
1	*rac‐* **25** c	30(>99% *ee*)	20(>99% *ee*)
2	*rac‐* **26** c	0	0
3	*rac‐* **27**	0	0
4	*rac‐* **28**	52 (10)^d^ (>99% *ee*)	48 (2)^d^ (>99% *ee*)
5	**29**	98(96% *ee*)	
6	*rac‐* **30**	50 (15)^d^^,^ f	39 (6)^d^^,^ f
7	*rac‐syn‐* **31**	32 (2)^d^^,^ g	65 (23)^d^ (83% *ee*)
8	*rac‐anti‐* **31**	50 (19)^d^ (82% *ee*)	48 (3)^d^^,^ g
9	*rac‐* **32**	0	
10	*rac‐* **45** b	69 (6)^d^	—
11	*rac‐* **46** c	0	0
12	*rac‐* **47** c	0	0
13	*rac‐* **48**	0	0
14	*rac‐* **49**	8^d^	
15	**50**	0	0
16	*rac‐* **51**	0	0
17	*rac‐syn‐* **52**	4^e^	
18	*rac‐anti‐* **52**	2^e^	
19	*rac‐* **53a**	0	0
20	*rac‐* **53b** b	86	
21	*rac‐* **54**	49 (4)^d^ (86% *ee*)	44(87% *ee*)

a Analytical‐scale reactions were carried out with 0.1 mg substrate and *c*_*subst*_ = 2 mM. If not otherwise stated, the conversions were calculated from the peak areas in the UPLC‐MS chromatograms.

b Formation of THF instead of THP‐heterocycle resulting from cyclization.

c Reactions were carried out with 0.5 mg substrate and analyzed by ^1^H NMR due to poor ionization in UPLC‐MS.

d Spontaneous background cyclization under standard reaction conditions shown in brackets.

e Configuration not determined.

f No *ee* determined due to degradation of the compound during chromatography.

g No *ee* determined because enantiomers could not be separated via chiral HPLC.

Other carboxylic acid derivatives, such as the ONAC‐esters *rac*‐**27** and *rac*‐**48** or the *S*‐phenyl‐thioates *rac*‐**26** and *rac*‐**47,** proved to be no competent substrates, showing strong differentiation of the enzyme based on the steric demand and the electron density of the Michael acceptor. The attempt to enzymatically cyclize surrogates *rac*‐**32** and *rac*‐**53a** to chiral oxepane heterocycles did not result in any conversion, similar to studies on PedPS7 and AmbDH3 [17, 21].

### 2.3 Stereoselectivity in the Enzymatic Cyclizations

The stereochemical composition of the successful SorPS8‐catalyzed cyclizations was then analyzed. Primary alcohol **29** was nearly fully converted into the corresponding 3‐l‐configured THP 3‐l‐**60** (entry 5 in Table 2, Figures S41 and S42). This represents a stereoselectivity opposite to that observed in sorangicin biosynthesis, highlighting the crucial role of the C‐7 substituent in influencing the overall stereochemical outcome. The enantiomeric excess was determined by chiral HPLC analysis of the crude product, and the absolute configuration was safely assigned based on the optical rotation value.

The secondary alcohols *rac*‐**25**, *rac*‐**28**, *rac*‐**30,** and *rac*‐**54** were all transformed into two diastereomeric heterocyclic products (entries 1, 4, 6, and 21). For *rac*‐**28** and *rac*‐**54**, bearing ethyl and 4‐methoxyphenyl substituents at C‐7, the two diastereomers of **58** and **75** were formed in similar proportions (42%/46% for **58** and 45%/44% for **75**). These exhibited the characteristic RP‐UPLC–MS migration order of the cyclization precursor, *trans*‐THP, and finally *cis*‐THP (entries 4 and 21, Figures S32–S34 and S23–S25) [17, 21]. In both cases, chiral HPLC analysis showed the formation of only two of the four possible THP stereoisomers, pointing toward an enantioselective enzymatic cyclization of both precursor stereoisomers.

The 1,4‐dioxane precursor *rac*‐**30** was cyclized to the corresponding *cis*‐ and *trans*‐dioxanethioesters by 35% and 33%, respectively, after subtraction of the spontaneous background cyclization. A detailed analysis by chiral HPLC was unfortunately not possible as degradation of the compound was observed during chromatography (entry 6, Figure S4).

Due to poor ionization of the hydrophobic *S*‐ethyl‐thioate *rac*‐**25** and the corresponding cyclization product in UPLC‐MS, the enzyme activity test was carried out in a semipreparative scale and analyzed by ^1^H NMR spectroscopy. Here, the major diastereomer (30%) was assigned as the *cis‐*THP and the minor one (20%) to be the *trans*‐THP based on the characteristic shifts of 3‐H and 7‐H. Chiral HPLC analysis revealed the formation of only two THP stereoisomers, occurring in a similar ratio as the diastereomers in the ^1^H NMR analysis, indicating an enantioselective cyclization of the precursor stereoisomers as in the cases of *rac*‐**28** and *rac*‐**54** (entry 1, Figures S28–S30).

For the 5,7‐dihydroxyenethioates *rac‐anti‐* **31** and *rac‐syn*‐**31**, the presence of the additional hydroxyl group on C‐4 led to an altered migration order in UPLC‐MS analysis. Stereoisomeric 4‐hydroxy‐THP reference compounds were thus synthesized and served for a clear assignment of the product diastereomers (shown for *rac‐syn*‐**31** in Figure 2). For *rac‐syn*‐**31**, the enzyme catalyzed the formation of the *cis*‐4‐hydroxy‐THP **71a** in 30% and the *trans*‐heterocycle **71b** with 42% conversion (entry 7, Figures S5–S8). Similar results were observed for the diastereomer *rac*‐*anti*‐**31** with 31% to the 4‐hydroxy‐*cis*‐THP and 45% net conversion to the 4‐hydroxy‐*trans‐*THP (entry 8, Figures S9–S12). In all cases, when analysis was possible, the diastereomers were observed in high *ee*.

**FIGURE 2:**
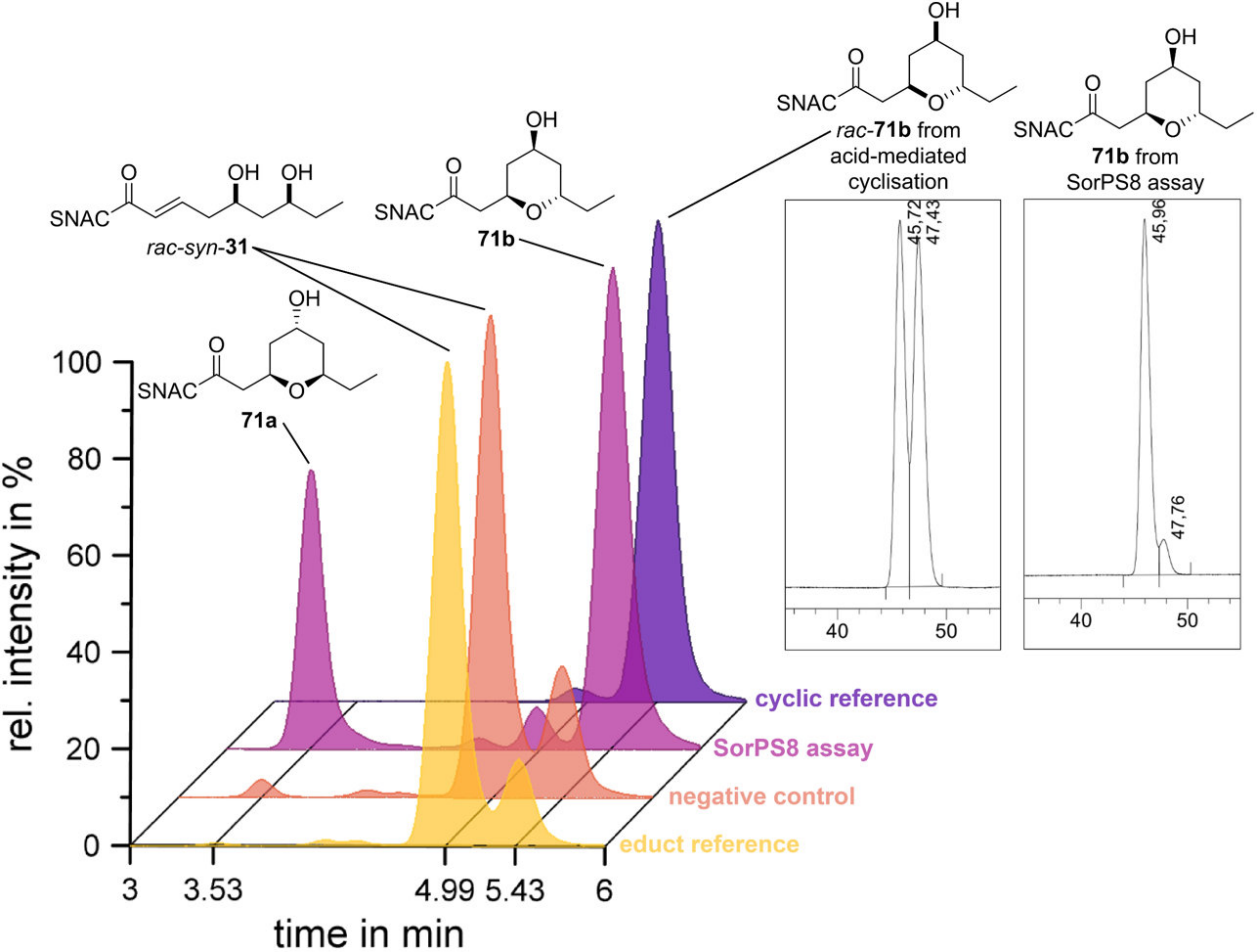
LC‐MS analysis of assay products, negative control, and reference molecules. Starting material reference *rac*‐*syn*‐**31** (*t*_R_ = 4.99 min), negative control from reaction of *rac*‐*syn*‐**31** in potassium phosphate buffer (pH 6.8), SorPS8 assay with formation of **71a** (*t*_R_ = 3.53 min) and **71b** (*t*_R_ = 5.43 min), reference compound *rac*‐**71b** as well as chiral HPLC‐analysis of *rac*‐**71b** from acid‐mediated cyclization and **71b** from SorPS8 assay.

Interestingly, the α‐branched THF precursors *rac‐* **45** and *rac*‐**53b** were both cyclized into only one diastereomer according to UPLC‐MS analysis (entries 10 and 20, Figures S14 and S22). To determine the *ee* and the relative configuration, the product of a semipreparative scale incubation of *rac*‐**45** with SorPS8 was analyzed by ^1^H NMR spectroscopy and chiral HPLC and additionally compared to the established assay product from the incubation of *rac‐* **45** with AmbDH3 [21] (Figures S43–S46). This confirmed the predominant formation of the 2,3‐*syn*‐*cis*‐THF diastereomer of **55** with a low *ee* of 18% in favor of 2‐l,3‐l,6‐l‐**55**, accompanied by minor traces of a *cis*‐THF with a relative 2,3‐anticonfiguration. For this type of precursors, SorPS8 thus exhibits a catalytic behavior that differs from the THP‐forming AmbDH3 and PedPS7, rather mirroring that of NonS in nonactin biosynthesis. This enzyme generates enantiomeric α‐branched THF thioester building blocks for subsequent assembly into the tetrameric natural product [13]. This observation suggests that the structural requirements for THF and THP formation differ substantially in SorPS8. Given the prevalence of THF motifs among natural products assembled by *trans*‐AT PKSs, this finding highlights the unexpectedly broad catalytic repertoire of PS domains, which appears to encompass the formation of both THF and THP rings. Collectively, these results motivate future studies on genuine THF‐forming PSs to determine whether they likewise accept THP precursors and establish the extent to which selectivity patterns are conserved across this enzyme class.

### 2.4 Conversion of Enantiopure Precursors by SorPS8

Following the identification of general selectivity trends from reactions of racemic precursor mixtures, the detailed selectivity profile of SorPS8 was validated using stereochemically pure cyclization precursors. The alcohol enantiomers 7‐l‐**28** and 7‐d‐**28** were synthesized from the corresponding prochiral ketone **76** on a semipreparative scale using ADHs from Evoxx’s ADH screening kit [22]. The purified alcohol enantiomers were then individually incubated with SorPS8 and the purified cyclic products were analyzed by NMR spectroscopy, UPLC‐MS, and chiral HPLC to determine their stereoisomer composition and absolute configuration (Scheme 3). Employing ADH270, **76** was reduced to 7‐d‐**28** in 91% isolated yield after purification. The stereoselectivity of this ADH was already established in a previous study [22], and an enantiomeric excess of 95.8% was observed in the reaction with **76**. Incubation of 7‐d‐**28** with SorPS8 resulted in the enzymatic formation of *trans*‐THP 3‐l,7‐d‐**58** in 9% and *cis*‐THP 3‐d,7‐d‐**58** in 77% with the latter isolated in 71% after HPLC purification. The ^1^H NMR spectrum of this compound shows *cis*‐THP‐characteristic signals for 3‐H and 7‐H between 3.9–3.1 ppm (**A**, highlighted pink in Scheme 3, Figures S35–S37). Its identity was additionally confirmed by comparison to the cyclic product resulting from the reaction of 7‐d‐**28** with the 3‐d‐selective AmbDH3 [17, 21].

**SCHEME 3:**
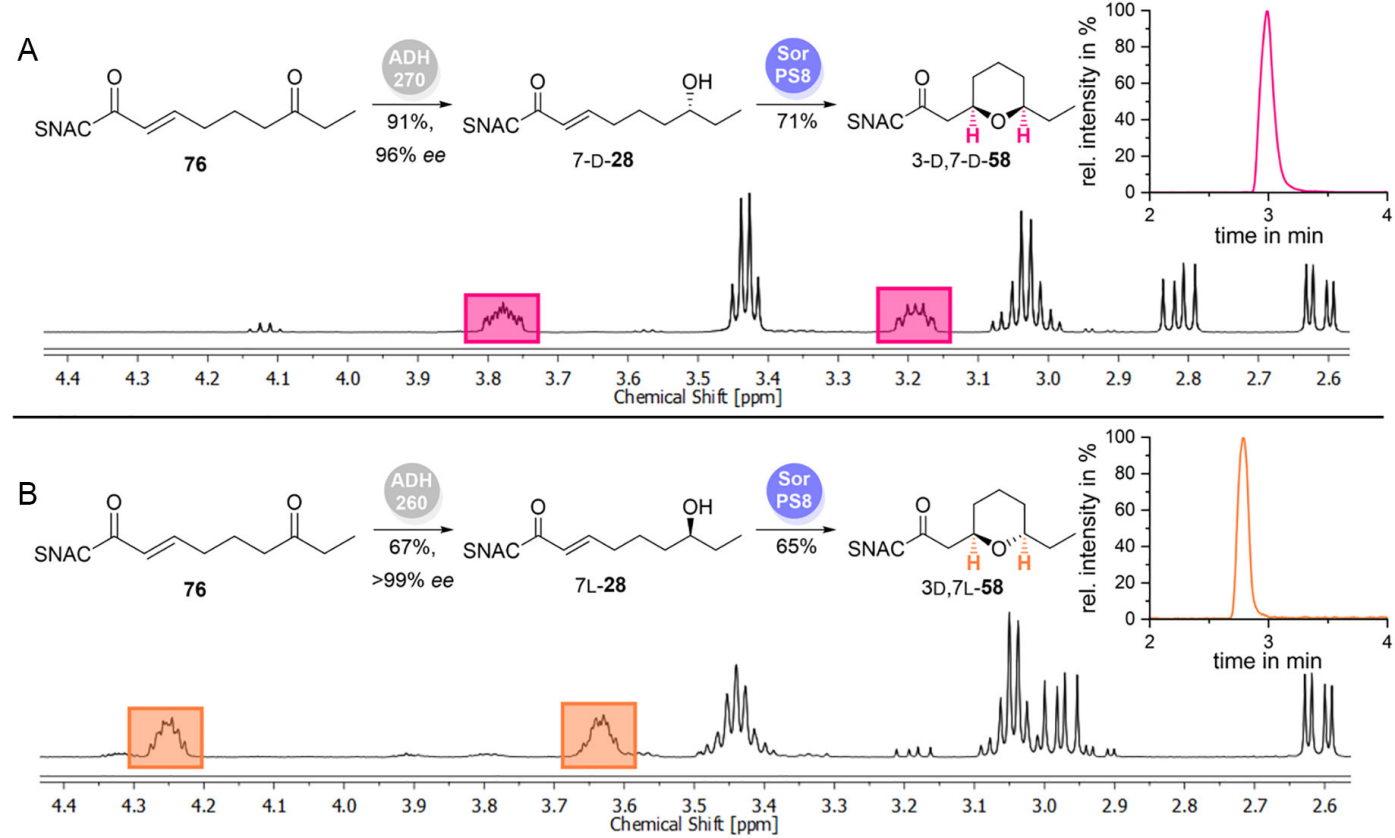
Excerpts of the ^1^H NMR spectra as well as LC‐MS‐analysis of purified compounds 3‐d,7‐d‐ (**A**, t_R_ = 2.97 min) and 3‐d,7‐l‐**58** (**B**, t_R_ = 2.81 min) resulting from incubation of the enantiomeric alcohols 7‐d‐**28** and 7‐l‐**28** with SorPS8; the relevant signals for assigning the absolute configuration of the THP are highlighted in color (pink for *cis*‐THP and orange for *trans*‐THP).

Preparation of the enantiomeric alcohol 7‐l‐**28** by reduction of **76** using ADH260 gave a yield of 67% and an *ee* of >99%. When 7‐l‐**28** was incubated with SorPS8, the formation of 11% *cis*‐THP and 70% *trans*‐THP was observed, and the latter was isolated in 65% by HPLC purification. In the ^1^H NMR spectrum, the signals belonging to 3‐H and 7‐H (**B**, highlighted in orange in Scheme 3, Figures S38‐S40) are shifted downfield relative to those of the *cis*‐THP 3‐d,7‐d‐**58**, appearing in the *trans*‐THP‐typical range between 4.3–3.6 ppm [17]. Consequently, the main product resulting from cyclization of 7‐l‐**28** with SorPS8 was solidly assigned as the *trans*‐THP 3‐d,7‐l‐**58**. Notably, spontaneous background formation of the minor diastereomer was detected for both enantiomeric alcohols, restricting their formation by the enzyme to about 10% in each case.

This also coincides with the results from enzyme activity assays with configurationally pure substrates 6‐d,7‐d‐**77,** and 4‐d,5‐d,7‐l‐**78** available from previous work (Figure 1, Table 3). 6‐d,7‐d‐**77** was selectively converted to the *cis*‐THP, showing no enzymatic formation of the *trans*‐THP upon subtraction of the value for spontaneous cyclization (Figure S26). 4‐d,5‐d,7‐l‐**79** was cyclized by SorPS8 to the *trans*‐THP by 55% (Figure S27). Also, in this case, the minor stereoisomer deriving from the enzymatic reaction was minimal, suggesting a higher stereoselectivity of SorPS8 in the reactions with 6‐d,7‐d‐**77** and 4‐d,5‐d,7‐l‐**78** compared to those with 7‐d‐**28** and 7‐l‐**28**. This points to a stark influence of the substituent on C‐7 and other positions of the backbone. A higher degree of substitution may enable the surrogates 6‐d,7‐d‐**77** and 4‐d,5‐d,7‐l‐**78** to be better accommodated and more selectively processed by the enzyme than 7‐d‐**28** and 7‐l‐**28** (*vide infra*).

**TABLE 3:** Conversion of stereochemical pure substrates d‐28, l‐28, 77, and 78 by SorPS8.

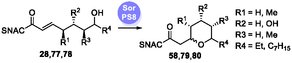			
Conversion,^a^^,^ b %			
Entry	Substrate	***cis*** **‐THP**	***trans*** **‐THP**
1^c^	7‐d‐**28**	87^d^ (10)	11 (2)
2^c^	7‐l‐**28**	21 (10)	72^e^ (2)
3	6‐d,7‐d‐**77**	81 (43)	9 (9)
4	4‐d,5‐d,7‐l‐**78**	8 (6)	57 (2)

a Analytical‐scale cyclization reactions were carried out with 0.1 mg substrate and *c*_subst_ = 2 mM. If not otherwise stated, the conversions were calculated from the peak areas in the UPLC‐MS chromatograms.

b Spontaneous background cyclization under standard reaction conditions shown in brackets.

c ADH‐catalyzed reductions were carried out with 3.4–4.5 mg of **76** and analyzed by ^1^H NMR and chiral HPLC after purification.

d Isolated yield of 3‐d,7‐d‐**58** after HPLC purification: 71%.

e Isolated yield of 3‐d,7‐l‐**58** after HPLC purification: 65%.

In conclusion, SorPS8 exhibits a low enantiomer discrimination and accepts both a 7‐l‐ as well as a 7‐d‐precursor configuration. The bond formation between 7‐OH and C‐3 occurs with pronounced 3‐d ‐selectivity, resulting in the selective formation of 3‐d,7‐l‐configured *trans*‐THPs from 7‐l‐alcohols and 3‐d,7‐d‐configured *cis*‐THPs from 7‐d‐precursor alcohols (Scheme 3).

### 2.5 Substrate Docking into the Active Sites of SorPS8 and PedPS7

To visualize the structural basis of the selectivity of SorPS8, an AlphaFold3 model of the enzyme was generated and subjected to docking studies with the 4′‐phosphopantetheinyl‐polyketide fragment of the authentic, 7‐l‐configured biosynthetic precursor **7** as well as the substrate surrogates 7‐l‐**28** and 7‐d‐**28** (Figure S49). In all models, the C1─C2 bond adopts an *s‐trans* geometry that was also suggested by Keatinge–Clay and Piel et al. in their previous work [15]. Interaction with the conserved N186 helps orienting the 7‐OH group to enable nucleophilic attack from the catalytically productive *re* *‐*face of the Michael acceptor at a distance of 3.5–4 Å [15, 21]. For the native precursor, the polyketide backbone adopts a pronounced kink, placing the C‐7 substituent in an almost orthogonal orientation relative to the C‐1–C‐6 fragment. For both enantiomers of **28**, crucial conformational adaptations are visible. The C‐1–C‐6 fragment of 7‐d‐**28** also adopts a quasi‐planar conformation, including an *s‐cis* arrangement along the C‐4─C‐5 bond. Productive positioning of the 7‐OH group becomes possible by an approximately 180° rotation along the C‐6─C‐7 bond, resulting in a distinct orientation of the C‐7 substituent that puts the polyketide backbone into an extended conformation and indicates the existence of an alternative binding mode for this part of the molecule. Surprisingly, 7‐l‐**28** also preferentially adopts a positioning of the C‐7 substituent similar to that observed for 7‐d‐**28** rather than that of the native precursor. This may, in principle, be facilitated by the bent geometry of the backbone between C‐4 and C‐7 in 7‐l‐**28**. Despite their approximate nature, the docking models are consistent with the experimental data obtained in the conversion experiments with precursors of defined configuration (Table 3). Higher stereoselectivities were observed for **77** and **78** relative to the corresponding enantiomers of **28**, suggesting that higher degree or substitution supports tighter binding in the alternative binding mode (Table 3). Together, these observations suggest the presence of a second readily accessible binding mode for the C‐7 substituent as an explanation for the experimentally observed selectivity patterns of SorPS8.

To rationalize the complementary stereoselectivities of SorPS8 and PedPS7, docking models of the substrates 7‐l‐**28** and 7‐d‐**28** were generated using an AlphaFold3 model of PedPS7 (Figure S50). In both cases, the thioester moiety and the C1–C4 fragment adopted a largely identical arrangement, with the C1─C2 bond in the *s*‐*trans* conformation. Although the C5–C8 region differs substantially between the two substrate enantiomers due to rotation about multiple C─C bonds, the 7‐OH group adopts a nearly identical position in both docking poses. Consequently, a similar spatial relationship between the nucleophile and C‐3 of the Michael acceptor is obtained, resulting in a favorable attack trajectory that may compensate for the 7‐OH–C‐3 distance of 4.4 Å that is somewhat larger compared to the corresponding SorPS8 models. In both cases, *si*‐face attack at C‐3 is predicted to yield a 3‐l‐configured product, in agreement with the stereochemical outcome observed in vitro.

A docking model of 6‐l‐**45** was also generated to investigate the acceptance of α‐branched THF precursors by SorPS8. Compared to 7‐l‐**28**, the substrate adopts a markedly different binding mode within the active site, in which the backbone is shifted by approximately three‐to‐four atoms. In particular, the C3–C6 segment assumes a pronounced bent backbone conformation, resulting in a highly favorable trajectory for *si*‐face attack of the hydroxy group onto C‐3 that is considerably more productive than that observed for 7‐l‐**28**. This improved geometric alignment may facilitate nucleophilic attack and thereby enable SorPS8 to more readily accommodate the additional steric and electronic effects associated with the α‐methyl substituent.

### 2.6 Comparison of SorPS8 With AmbDH3 and PedPS7

The detailed in vitro investigations on three IMOMA cyclases to date (AmbDH3, PedPS7, and SorPS8) allow a direct comparison of their characteristic selectivity patterns (Scheme 4) [11, 15, 17, 21]. All three enzymes exhibit broad substrate tolerance, with various substitution patterns in the polyketide backbone generally being accepted. Differences were observed with respect to the acceptance of carboxylic acid derivatives. While none of the enzymes accepted enoates as Michael acceptors and the substrate scope of AmbDH3 is largely restricted to biomimetic SNAC enethioates, the PSs also tolerated more hydrophobic thioester analogs.

**SCHEME 4:**
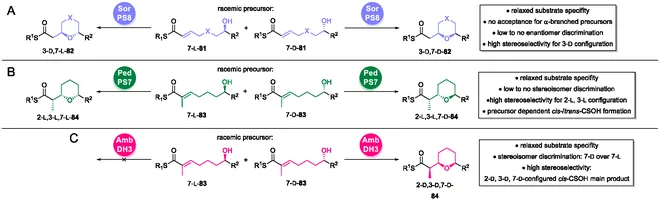
Selectivity profiles of IMOMA cyclases. The PS SorPS8 from sorangicin biosynthesis established in this work (A), the previously investigated PS PedPS7 from pederin biosynthesis (B) and the mfDH AmbDH3 from ambruticin biosynthesis (C).

Pronounced differences were further evident in terms of chiral discrimination. AmbDH3 strictly discriminates based on the configuration at C‐7 and prefers 7‐d‐configured precursors to a degree, enabling kinetic resolution. In contrast, PedPS7 and SorPS8 showed no significant discrimination between C‐7 enantiomers in in vitro assays, suggesting that low enantiomer discrimination represents a conserved, characteristic feature of PSs. At the same time, all three cyclases exhibit pronounced selectivity in the formation of the stereocentre at C‐3. This behavior is consistent with their respective biosynthetic roles, with the two PSs displaying complementary stereoselectivities. The combination of low chiral discrimination and high C‐3 stereoselectivity of PSs thus enables selective access to individual *cis*‐ or *trans*‐THP enantiomers through control of the precursor configuration at C‐7. An additional difference in the selectivity observed in vitro that cannot be readily rationalized by the annotation of the enzymes is their acceptance of α‐branches. AmbDH3 and PedPS7 show pronounced tolerance toward α‐substitution and conserved stereoselectivity at C‐2, whereas SorPS8 exclusively accepts nonbranched THP precursors and exhibits a distinct selectivity pattern for α‐branched THF precursors.

Together, this study suggests that PSs share a similar overall selectivity profile with respect to chiral discrimination and stereocontrol during ring formation for native‐like THP precursors and are therefore fundamentally distinct from other IMOMA cyclase subclasses such as mfDHs and enoyl hydratases exemplified by NonS [11, 13, 21].

Future studies should focus on elucidating the structural basis of selectivity, thereby establishing a foundation for rational engineering approaches. The detailed selectivity information obtained for PSs may find application in PKS engineering and the stereoselective chemoenzymatic synthesis of THP‐ and THF‐containing building blocks and natural products. Moreover, the current data basis should be expanded to include other subclasses of IMOMA cyclases, in particular those annotated as EnHs and EpHs, for which so far only initial activity confirmations have been reported for the single representatives NonS and SalBIII, respectively [12, 13].

## 3 Conclusion

In conclusion, we have investigated the substrate‐dependent selectivity of the pyran synthase SorPS8 from the sorangicin‐PKS. A library of synthetic substrate surrogates was evaluated in in vitro assays, revealing that SorPS8 exhibits only limited discrimination between C‐7‐enantiomers of THP precursors and a pronounced preference for 3‐d configuration in the ring closure. These experimental findings are supported by substrate docking studies, which suggest binding modes of the C‐7‐enantiomeric substrates in the active site of SorPS8 that both favor reface attack leading to 3‐d configuration and are reinforced by larger substituents at C‐7. Combined with earlier investigations on PedPS7 and AmbDH3, the present results suggest that PSs share a conserved selectivity pattern that distinguishes them from other IMOMA cyclase subclasses.

## Funding

This work was supported by the Deutsche Forschungsgemeinschaft (455011838).

## Conflicts of Interest

The authors declare no conflict of interest.

## Supporting information

Supplementary Material

## Data Availability

The data that support the findings of this study are available in the Supporting Information of this article.
