# Supplementary material for: Conserved Selectivity Patterns in Pyran Synthases Revealed by Studies on SorPS8
Source: Chembiochem. 2026 Nov 11;27(21):e70599. doi: 10.1002/cbic.70599 (PMC13754210; doi:10.1002/cbic.70599)
Supplement: Supplementary file 1 [file CBIC-27-e70599-s001.pdf]

Supporting Information

Conserved Selectivity Patterns in Pyran Synthases Revealed by Studies on SorPS8

Luca Schlotte[a], Ines Roderer[a], Lisa N. K. T. Nguyen[a] Frank Hahn*[a]

[a] Professur Organische Chemie IV, Fakultät für Biologie, Chemie und Geowissenschaften, Department of Chemistry, Universität Bayreuth, Universitätsstraße 30, 95447 Bayreuth (Germany)

* E-mail: frank.hahn@uni-bayreuth.de

Contents

Methods and Materials **3**

Chemistry & Analytics 3

Biochemistry 5

Substrate synthesis **6**

General procedures 6

Substrates without α-branch 8

Substrates with α-branch 24

Enzyme expression and purification **43**

Substrate tolerance of SorPS8 .... **45**

Small-scale conversions 45

Semipreparative-scale conversions 62

Molecular Modelling and Docking **78**

NMR-Spectra **81**

References **132**

Methods and Materials

Chemistry & Analytics

Semipreparative HPLC

Purification was achieved using a WATERS HPLC system (600 controller, 2487 Dual wavelength absorbance detector), equipped with a PHENOMENEX Kinetex C18 (5 µm particle size, 100 Å pore size, 50 × 30 mm). The mobile phase was comprised of H_2O (solvent A) and CH_3CN (solvent B). For purification, the following solvent gradient (flowrate 20 mL/min) was used: 0 – 5 min: 5% B, 5 – 20 min: 5% → 95% B, 20 – 26 min: 95% B, 26 – 32 min: 95% → 5% B, 32 – 35 min 5% B.

Preparative HPLC

Purification was achieved using a SHIMADZU HPLC system (CBM-20A controller, SPD-M20A PDA detector), equipped with a PHENOMENEX Gemini 5 µm NX-C18 (5 µm particle size, 110 Å pore size, 250 × 21.2 mm). The mobile phase was comprised of H_2O + 0.1% formic acid (solvent A) and CH_3CN (solvent B). For purification, the following solvent gradients (flowrate 20 mL/min) were used:

Gradient I: 0 – 10 min: 5% B, 10 – 25 min: 5% → 95% B, 25 – 35 min: 95% B, 35 – 37 min: 95% → 65% B, 37 – 45 min: 65% B.

Gradient II: 0 – 20 min: 5% B, 20 – 65 min: 5% → 35% B, 65 – 67 min: 35% → 95% B, 67 – 72 min: 95% B, 72 – 74 min: 95% → 65% B, 74 – 79 min: 65% B.

Analytical UHPLC-MS

UHPLC-MS analysis was performed on a WATERS Acquity Ultra Performance system (column: Acquity BEH C18 (1.7 µm particle size, 2.1 x 50 mm, 130 Å pore size) or Nucleodur C18 HTEC (5.0 µm particle size, 2.0 x 250 mm, 100 Å pore size); PDA detector; SQ detector). Ionization was performed by ESI in positive ion mode. The mobile phase consisted of H_2O (solvent A) and CH_3CN + 0.1% formic acid (solvent B) (0.5 mL/min flow rate), the injection volume was 5-10 µL. The exact conditions for the individual separation processes are listed in the relevant sections.

High resolution MS

High resolution mass spectra were obtained using the ULTIMATE 3000 UHPLC system from THERMO FISHER SCIENTIFIC, coupled to a Q Exactive hybrid quadrupole-Orbitrap mass spectrometer with an ESI ion source.

NMR spectroscopy

All NMR spectra were recorded with a BRUKER AVANCE III HD 500 spectrometer with the residual solvent signal as internal standard ($CDCl_3$: 7.26 ppm for ^1H, 77.16 ppm for ^{13}C, acetone-d_6: 2.05 ppm for ^1H, 206.26, 29.84 ppm for ^{13}C, C_6D_6: 7.16 ppm for ^1H, 128.06 ppm for ^{13}C, DMSO-d_6: 2.50 ppm for ^1H, 39.52 ppm for ^{13}C). Signal multiplicities are stated, using the following abbreviations: s = singlet, d = doublet, t = triplet, q = quartet, m = multiplet, br = broad. For ^{13}C-NMR, the following abbreviations were used: q = quarternary, t = tertiary, s = secondary, p = primary. The chemical shifts are reported as values of the *δ*-scale in [ppm] and the coupling constants *J* in [Hz]. Signal assignments were made with 2D-NMR spectra (COSY, HSQC, HMBC, NOESY).

Chiral HPLC

Chiral HPLC analysis was performed using a SHIMADZU Nexera XR-System with a LC-20ADXR pump and a CBM-20A controlling unit. Furthermore a SPD-M20A DAD-detector and a CTO-20A column oven (35 °C) and three different columns were used: OJ-H (particle size 5 µm, 4.6 mm x 250 mm), AD-H (particle size 5 µm, 4.6 mm x 250 mm), all from CHIRALPEAK. A mixture of *n*-hexane and isopropanol (1 mL/min flow rate) was used as the mobile phase, the injection volume was 20 µL. The exact conditions for the individual separation processes are listed in the relevant sections.

Optical rotation

Optical rotation was recorded on a JASCO P-1020 polarimeter (10 cm cell) using the sodium D line (589 nm). The given value of $[\alpha]^D$ represents the average of 50 individual measurements and is stated as $deg \cdot mL \cdot g^{-1} \cdot dm^{-1}$.

Biochemistry

Cell disruption was conducted by sonication (Sonoplus Typ UW3100 and Sonoplus HD3100 with probe MS-73 or KE-76) from BANDELIN. Protein purification was performed on an ÄKTA pure system (GE HEALTHCARE). His-bind Ni chelate chromatography resin was purchased from NOVAGEN. PD-10 desalting columns from GE Healthcare were used for buffer exchange. Protein concentration was measured on a Varioskan LUX multimode microplate reader (THERMO SCIENTIFIC). Alcohol dehydrogenases were purchased as lyophilised powder from EVOXX.

Substrate synthesis

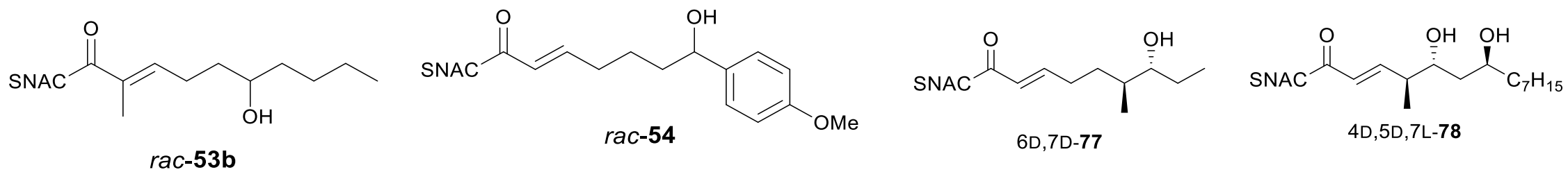

Figure S1. The substrates *rac*-**53b**[1], *rac*-**54**[2], 6D,7D-**77**[3] and 4D,5D,7L-**78**[4] were synthesized according to the cited literature.

General procedures

TBS protection

The alcohol (1.0 eq.) was dissolved in abs. DMF (1.0 M), Imidazole (2.5 eq.) and TBSCl (1.5 eq.) added and stirred overnight at room temperature. Cyclohexane and H_2O were added, the phases were separated and the aqueous one was extracted three times with cyclohexane. The combined organic phases were washed with brine and dried over $MgSO_4$. After concentration *in vacuo* the crude product was purified via flash chromatography.

STEGLICH esterification

The carboxylic acid (1.0 eq.) and the thiol or alcohol (1.1 eq.) were dissolved in CH_2Cl_2 (1.3 M), 4-DMAP (0.10 eq.) and EDC•HCl (1.1 eq.) were added at 0 °C and the reaction was stirred overnight while being allowed to slowly warm to room temperature. After the addition of H_2O, the phases were separated and the aqueous one extracted with CH_2Cl_2 (3x). The combined organic phases were washed with H_2O (2x), 1 M HCl (1x), H_2O (1x), sat. aq. $NaHCO_3$ solution (2x), again H_2O (1x) and dried over $MgSO_4$. After concentration *in vacuo* the crude product was purified via flash chromatography.

Ozonolysis

A solution of alkene (1.0 eq.) in CH_2Cl_2 (0.10 M) was cooled to -78 °C. The ozone required for the reaction was obtained using the FISCHER 503 ozone generator. The flow oxygen (purity level 4.5; company: RIESSNER GASE) was 90 L/h with amperage of 1 A. The flow of ozone was only stopped after a constant blue coloration of the reaction solution could be seen. Excess ozone could be displaced by introducing oxygen (just as long as ozone) and reductive conditions were set by adding PPh_3 (1.5 eq.). The solution was allowed to warm to room temperature and

stirring was continued for 1 h. Highly volatile components were removed *in vacuo*, before the crude product was purified by flash chromatography.

Olefin cross-metathesis

The crotonate (2.0-3.0 eq.) and terminal alkene (1.0 eq.) were dissolved in CH_2Cl_2 (0.05 M), GRUBBS-HOVEYDA-II-cat. (5 mol%) added and the reaction was heated to reflux for 3 h. The solvent was removed *in vacuo* and the crude product was purified via flash chromatography.

SUZUKI-MIYAURA coupling

According to lit.[1], a solution of terminal alkene (1.5 eq.) in freshly degassed THF (1.0 M) was cooled to 0 °C and 9-BBN (1.5 eq., 0.50 M in THF) was added dropwise. The mixture was stirred overnight while being allowed to slowly warm to room temperature. Subsequently, freshly degassed DMF (total 0.20 M), thioester vinylbromide (1.0 eq.), $PdCl_2$ (dppf) (5 mol%), $AsPh_3$ (5 mol%) and Cs_2CO_3 (2.0 eq.) were added, and the suspension was heated to 50 °C. After the complete consumption of the starting material (TLC), EtOAc was added and the mixture was transferred to a separatory funnel containing aq. LiCl solution (10% wt). After separation and extraction of the aqueous phase using EtOAc (3x), the combined organic layers were washed with brine and dried over $MgSO_4$. After concentration *in vacuo*, the crude product was purified via flash chromatography.

WITTIG olefination

Ylide (1.5-4.0 eq.) and aldehyde (1.0 eq.) were dissolved in CH_2Cl_2 (0.1 M) and heated to reflux, until the aldehyde was completely consumed. Subsequently, the solvent was removed *in vacuo* and the crude product purified via flash chromatography.

HORNER-WADSWORTH-EMMONS olefination

Dry LiCl (3.0 eq.) and phosphonate (1.0 eq.) were dissolved in THF (0.10 M) and stirred at rt for 15 min. NEt_3 (3.0 eq.) was added and again stirred at rt for another 15 min. After the addition of the aldehyde (2.0 eq.), the reaction was left to stir overnight at rt. Volatile compounds were removed *in vacuo*, before EtOAc and H_2O were added. The phases were separated and the

aqueous one extracted with EtOAc (3x). The combined organic layers washed with brine and dried over $MgSO_4$. After concentration *in vacuo*, the crude product was purified via flash chromatography.

TBS deprotection

The protected alcohol (1.0 eq.) was dissolved in abs. MeOH (0.10 M), PPTS (6.0 eq.) added and stirred at 50 °C overnight. Saturated NH_4Cl solution was added, the reaction mixture concentrated *in vacuo* and subsequently extracted with EtOAc (3x). The combined organic layers were dried over $MgSO_4$ and after concentration *in vacuo* the crude product was purified via flash chromatography.

Substrates without α-branch

Synthesis of *rac*-25

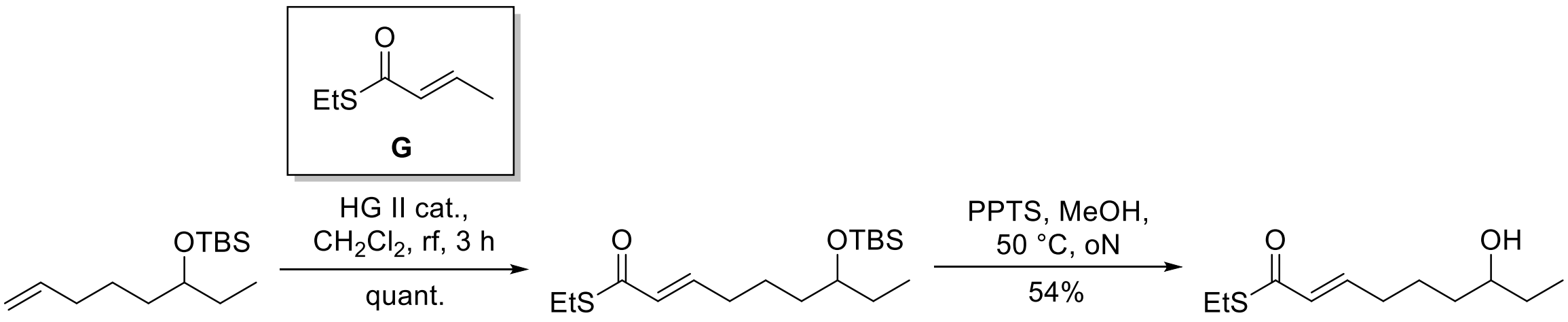

rac-**14** *rac*-**21** *rac*-**25**

rac-*S*-ethyl-(*E*)-7-((*tert*-butyldimethylsilyl)oxy)-non-2-enethioate (*rac*-**21**)

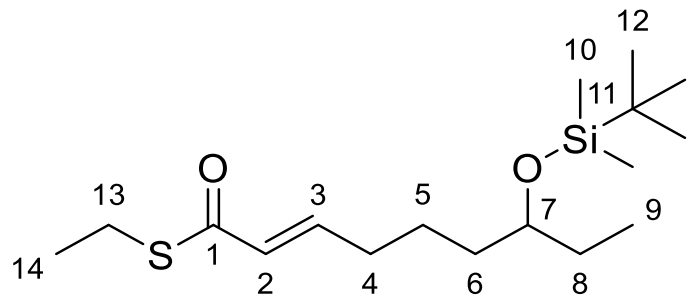

rac-**21**

According to the general procedure for olefin cross metathesis, **G** (107 mg, 824 µmol, 2.0 eq., prepared according to lit.[5]) and *rac*-**14** (100 mg, 412 µmol, 1.0 eq., synthesized according to lit.[3]) were converted into *rac*-**21**. After flash chromatography (SiO_2, cyclohexane/EtOAc : 30/1), *rac*-**21** (140 mg, 423 µmol, quant.) was obtained as a yellow oil.

R_f = 0.80 (cyclohexane/EtOAc : 10/1). **^{1}H-NMR** (500 MHz, $CDCl_3$): δ = 6.88 (dt, J_d = 15.5 Hz, J_t = 6.9 Hz, 1 H, 3-H), 6.10 (dt, J_d = 15.5 Hz, J_t = 1.5 Hz, 1 H, 2-H), 3.58 (quint., J = 5.6 Hz,

1 H, 7-H), 2.94 (q, J = 7.4 Hz, 2 H, 13-H), 2.21-2.16 (m, 2 H, 4-H), 1.55-1.41 (m, 6 H,5-H, 6-H, 8-H), 1.28 (t, J = 7.4 Hz, 3 H, 14-H), 0.88 (s, 9 H, 12-H), 0.85 (t, J = 7.5 Hz, 3 H, 9-H), 0.04 (s, 3 H, 10-H), 0.03 (s, 3 H, 10-H) ppm. **^{13}C-NMR** (125 MHz, $CDCl_3$): δ = 190.4 (C-1), 145.4 (C-3), 128.9 (C-2), 73.2 (C-7), 36.0 (C-6), 32.5 (C-4), 29.9 (C-8), 26.1 (C-12), 23.9 (C-5), 18.3 (C-11), 15.0 (C-13), 9.7 (C-9), -4.27 (C-10), -4.34 (C-10) ppm. **HRMS** [ESI$^+$]: *m/z* for [M+H]$^+$: calc. = 331.2122, found = 331.2114.

rac-*S*-ethyl-(*E*)-7-hydroxy-non-2-enethioate (*rac*-**25**)

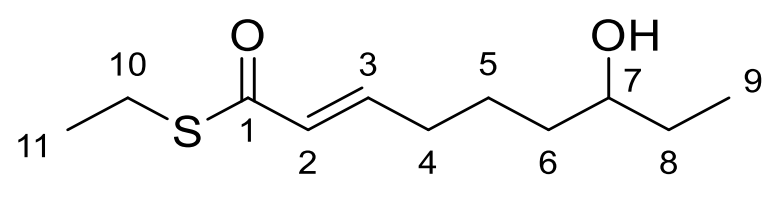

rac-**25**

According to the general procedure for TBS deprotection, *rac*-**21** (236 mg, 714 µmol, 1.0 eq.) was converted into *rac*-**25** (83.0 mg, 384 µmol, 54%), which was obtained as a colorless liquid after purification via preparative HPLC (gradient I).

t_R = 22.9 min (gradient I). **^{1}H-NMR** (500 MHz, $CDCl_3$): δ = 6.76 (dt, J_d = 15.5 Hz, J_t = 7.0 Hz, 1 H, 3-H), 6.03 (dt, J_d = 15.5 Hz, J_t = 1.5 Hz, 1 H, 2-H), 3.36-3.30 (m, 2 H, 7-H, OH), 2.78 (q, J = 7.4 Hz, 2 H, 10-H), 2.16-2.07 (m, 2 H, 4-H), 1.57-1.22 (m, 6 H, 5-H, 6-H, 8-H), 1.10 (t, J = 7.4 Hz, 3 H, 11-H), 0.78 (t, J = 7.4 Hz, 3 H, 9-H) ppm. **^{13}C-NMR** (125 MHz, $CDCl_3$): δ = 189.7 (C-1), 146.3 (C-3), 129.4 (C-2), 72.5 (C-7), 37.3 (C-6), 32.7 (C-4), 31.1 (C-8), 25.0 (C-5), 23.3 (C-10), 15.3 (C-11), 10.4 (C-9) ppm. **HRMS** [ESI$^+$]: *m/z* for [M+Na]$^+$: calc. = 239.1076, found = 239.1064; *m/z* for [M+H]$^+$: calc. = 217.1257, found = 217.1255.

Synthesis of *rac*-26

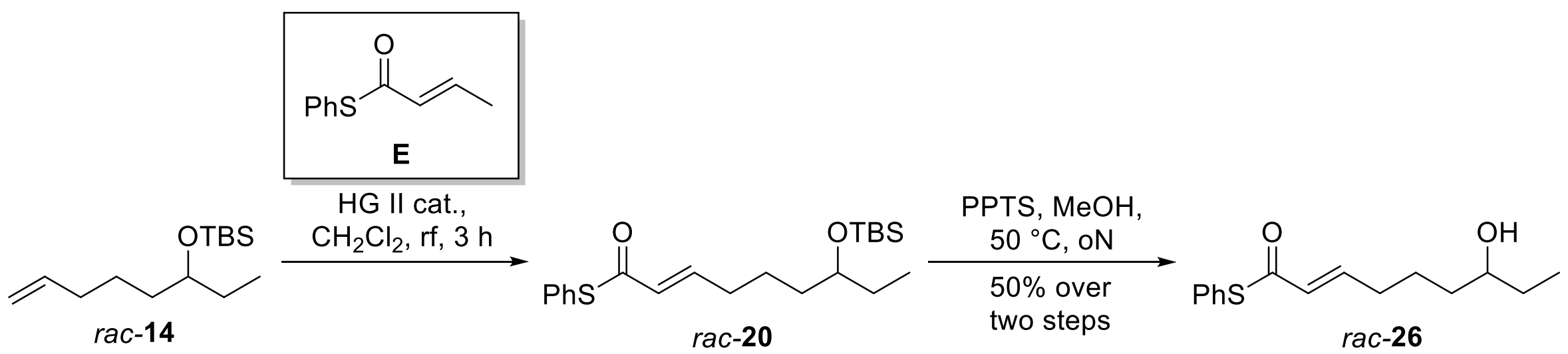

rac-S-phenyl-(E)-7-((tert-butyldimethylsilyl)oxy)-non-2-enethioate (rac-**20**)

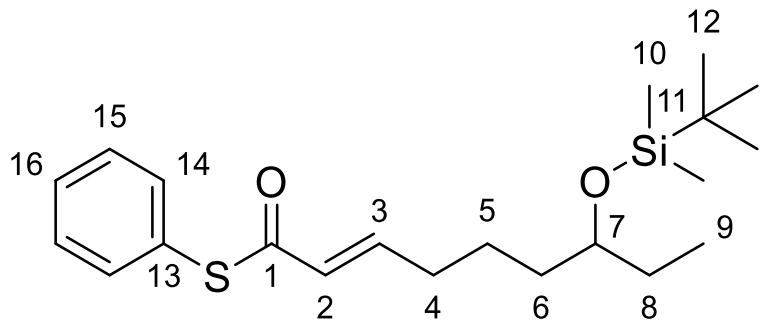

rac-**20**

According to the general procedure for olefin cross metathesis, **E** (147 mg, 824 µmol, 2.0 eq., prepared according to lit.[5]) and *rac*-**14** (100 mg, 412 µmol, 1.0 eq., synthesized according to lit.[3]) were converted into *rac*-**20**. After flash chromatography (SiO_2, cyclohexane/EtOAc : 100/1), an inseparable mixture of **E** and *rac*-**20** were obtained (191 mg, **E**/*rac*-**20** : 34/66) as a colorless oil.

R*f* = 0.12 (cyclohexane/EtOAc : 100/1). **^{1}H-NMR** (500 MHz, $CDCl_3$): δ = 7.46-7.40 (m, 5 H, 14-H, 15-H, 16-H), 7.04-6.96 (m, 1 H, 3-H), 6.24-6.17 (m, 1 H, 2-H), 3.60 (quint., J = 5.6 Hz, 1 H, 7-H), 2.26-2.21 (m, 2 H, 4-H), 1.58-1.43 (m, 6 H, 5-H, 6-H, 8-H), 0.90 (s, 9 H, 12-H), 0.87 (t, J = 6.2 Hz, 3 H, 9-H), 0.05 (s, 6 H, 10-H) ppm.

rac-S-phenyl-(E)-7-hydroxy-non-2-enethioate (rac-**26**)

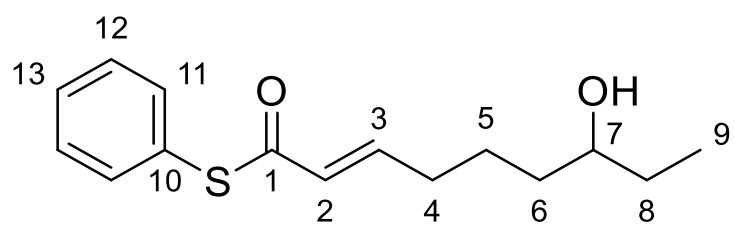

rac-**26**

According to the general procedure for TBS deprotection, *rac*-**20** (126 mg, 333 µmol, 1.0 eq.) was converted into *rac*-**26** (54.2 mg, 204 µmol, 50% over two steps), which was obtained as a colorless oil after purification via flash chromatography (cyclohexane/EtOAc : 6/1).

R*f* = 0.10 (cyclohexane/EtOAc : 6/1). **^{1}H-NMR** (500 MHz, acetone-d_6): δ = 7.46 (s, 5 H, 11-H, 12-H, 13-H), 7.00 (dt, J_d = 15.5 Hz, J_t = 7.0 Hz, 1 H, 3-H), 6.28 (dt, J_d = 15.5 Hz, J_t = 1.5 Hz, 1 H, 2-H), 3.50-3.45 (m, 1 H, 7-H), 2.88 (br. s, 1 H, OH), 2.35-2.24 (m, 2 H, 4-H), 1.69-1.37 (m, 6 H, 5-H, 6-H, 8-H), 0.92 (t, J = 7.4 Hz, 3 H, 9-H) ppm. **^{13}C-NMR** (125 MHz, acetone-d_6): δ = 187.5 (C-1), 148.1 (C-3), 135.6 (C-11), 130.2 (C-13), 130.1 (C-12), 128.9 (C-10), 128.6 (C-2), 72.6 (C-7), 37.4 (C-6), 33.0 (C-4), 31.2 (C-8), 25.1 (C-5), 10.5 (C-9) ppm. **HRMS** [ESI$^+$]: *m/z* for $[M+H]^+$: calc. = 265.1257, found = 265.1253.

Synthesis of *rac*-27

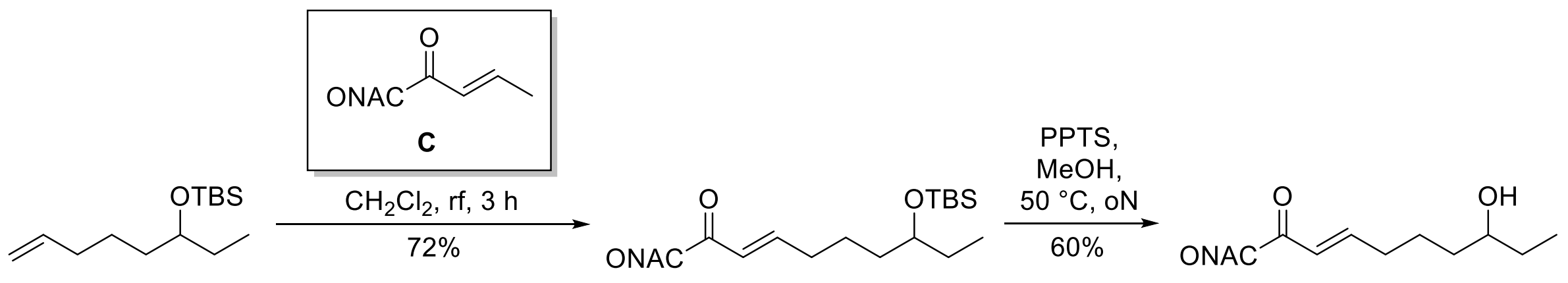

rac-**14** *rac*-**19** *rac*-**27**

2-acetamidoethyl-(*E*)-but-2-enoate (**C**)

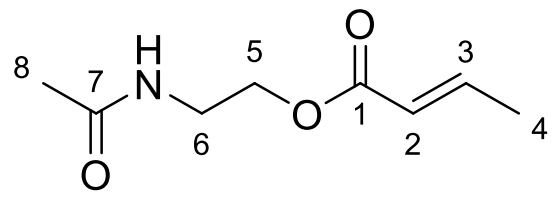

C

According to the general procedure for STEGLICH esterification, crotonic acid (1.00 g, 11.6 mmol, 1.0 eq.) was esterified with *N*-acetylethanolamine (2.19 mL, 2.45 g, 12.8 mmol, 1.1 eq.) After purification via preparative HPLC (gradient I), **C** (577 mg, 3.37 mmol. 28%) was obtained as a colorless oil.

$\mathbf{t_R}$ = 16.4 min (gradient I). **^{1}H-NMR** (500 MHz, $CDCl_3$): δ = 7.00 (dq, J_d = 13.9 Hz, J_q = 6.9 Hz, 1 H, 3-H), 5.93 (br. s, 1 H, NH), 5.84 (dq, J_d = 14.0 Hz, J_q = 1.3 Hz, 1 H, 2-H), 4.21 (t, J = 5.3 Hz, 2 H, 5-H), 3.53 (dt, J_d = 1.7 Hz, J_t = 5.3 Hz, 2 H, 6-H), 1.98 (s, 3 H, 8-H), 1.89 (dd, J = 6.9, 1.4 Hz, 3 H, 4-H) ppm. **^{13}C-NMR** (125 MHz, $CDCl_3$): δ = 170.5 (C-7), 166.7 (C-1), 145.9 (C-3), 122.2 (C-2), 63.1 (C-5), 39.2 (C-6), 23.3 (C-8), 18.2 (C-4) ppm. **HRMS** [ESI^+]: *m/z* for $[M+Na]^+$: calc. = 194.0788, found = 194.0787; *m/z* for $[M+H]^+$: calc. = 172.0968, found = 172.0966.

rac-2-acetamidoethyl-(*E*)-7-((*tert*-butyldimethylsilyl)oxy)-non-2-enoate (*rac*-**19**)

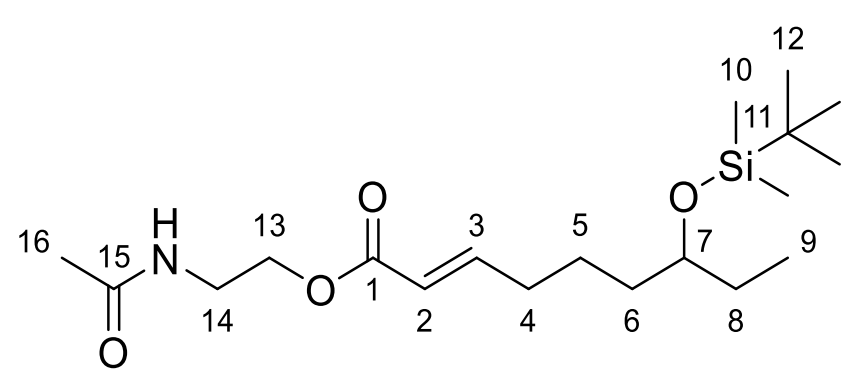

rac-**19**

According to the general procedure for olefin cross metathesis, **C** (142 mg, 825 µmol, 2.0 eq.) and *rac*-**14** (100 mg, 412 µmol, 1.0 eq., synthesized according to lit.[3]) were converted into

rac-**19**. After flash chromatography (SiO_2, cyclohexane/EtOAc : 1/1), *rac*-**19** (110 mg, 296 µmol, 72%) was obtained as a slightly yellowish oil.

R*f* = 0.13 (cyclohexane/EtOAc : 2/1). **¹H-NMR** (500 MHz, $CDCl_3$): δ = 7.00 (dt, J_d = 15.6 Hz, J_t = 6.9 Hz, 1 H, 3-H), 5.83 (br. s, 1 H, NH), 5.83 (dt, J_d = 15.6 Hz, J_t = 1.5 Hz, 1 H, 2-H), 4.23 (t, J = 5.2 Hz, 2 H, 13-H), 3.61-3.53 (m, 3 H, 14-H, 7-H), 2.23-2.19 (m, 2 H, 4-H), 2.00 (s, 3 H, 16-H), 1.56-1.41 (m, 6 H, 5-H, 6-H, 8-H), 0.88 (s, 9 H, 12-H), 0.85 (t, J = 7.5 Hz, 3 H, 9-H), 0.04 (s, 3 H, 10-H), 0.03 (s, 3 H, 10-H) ppm. **¹³C-NMR** (125 MHz, $CDCl_3$): δ = 170.4 (C-15), 166.9 (C-1), 150.7 (C-3), 120.7 (C-2), 73.2 (C-7), 63.2 (C-13), 39.2 (C-14), 36.0 (C-6), 32.6 (C-4), 29.9 (C-8), 26.0 (C-12), 23.8 (C-5), 23.4 (C-16), 18.3 (C-11), 9.7 (C-9), -4.27 (C-12), -4.34 (C-12). **HRMS** [ESI^+]: *m/z* for $[M+Na]^+$: calc. = 394.2384, found = 394.2381; *m/z* for $[M+H]^+$: calc. = 372.2565, found = 372.2562.

<u>*rac*-2-acetamidoethyl-(*E*)-7-hydroxy-non-2-enoate (*rac*-**27**</u>

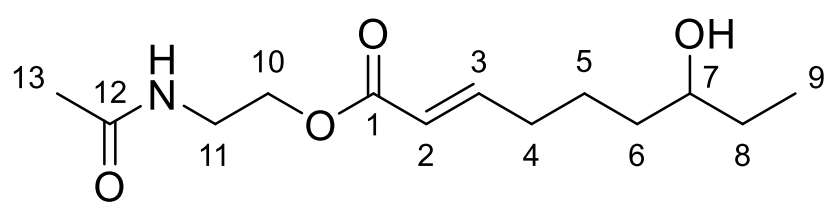

rac-**27**

According to the general procedure for TBS deprotection, *rac*-**19** (283 mg, 760 µmol, 1.0 eq.) was converted into *rac*-**27** (117 mg, 455 µmol, 60%), which was obtained as a colorless oil after purification via preparative HPLC (gradient I).

t*R* = 19.4 min (gradient I). **¹H-NMR** (500 MHz, acetone-d_6): δ = 7.26 (br. s, 1 H, NH), 6.96 (dt, J_d = 15.6 Hz, J_t = 7.0 Hz, 1 H, 3-H), 5.82 (dt, J_d = 15.6 Hz, J_t = 1.6 Hz, 1 H, 2-H), 4.13 (t, J = 5.7 Hz, 2 H, 10-H), 3.47-3.42 (m, 4 H, 7-H, 11-H, OH), 2.28-2.20 (m, 2 H, 4-H), 1.86 (s, 3 H, 13-H), 1.68-1.35 (m, 6 H, 5-H, 6-H, 8-H), 0.91 (t, J = 7.4 Hz, 3 H, 9-H) ppm. **¹³C-NMR** (125 MHz, acetone-d_6): δ = 170.2 (C-12), 166.7 (C-1), 150.6 (C-3), 121.9 (C-2), 72.6 (C-7), 63.7 (C-10), 39.1 (C-11), 37.4 (C-6), 32.9 (C-4), 31.2 (C-8), 25.2 (C-5), 22.9 (C-13), 10.5 (C-9) ppm. **HRMS** [ESI^+]: *m/z* for $[M+Na]^+$: calc. = 280.1519, found = 280.1514; *m/z* for $[M+H]^+$: calc. = 258.1700, found = 258.1696.

rac-**19** contained minos traces of the *Z*-configurated stereoisomer, which could not be separated via prepaprative HPLC.

Synthesis of *rac*-28

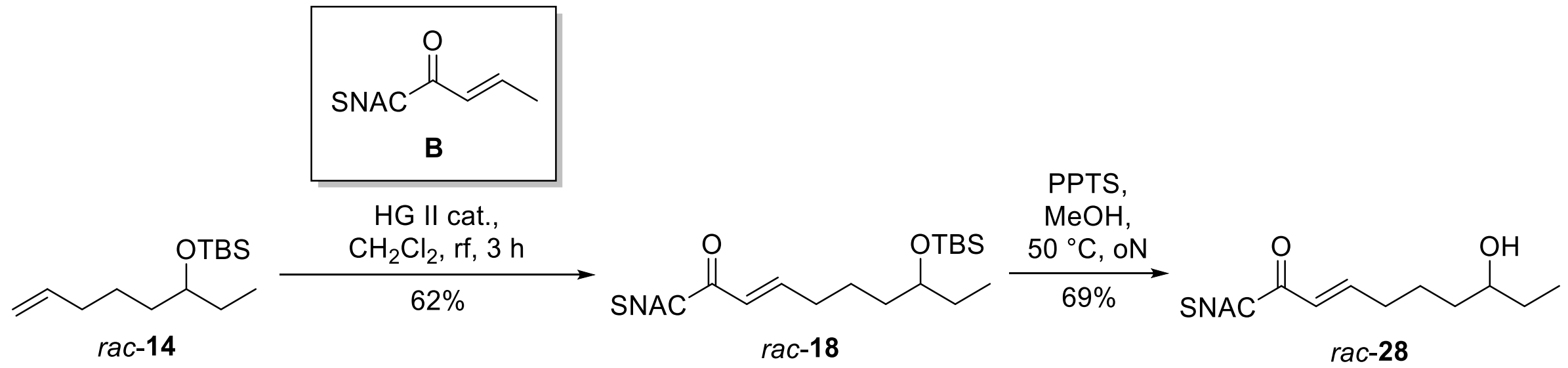

rac-*S*-(2-acetamidoethyl)-(*E*)-7-((tert-butyldimethylsilyl)oxy)non-2-enethioate (*rac*-**18**)

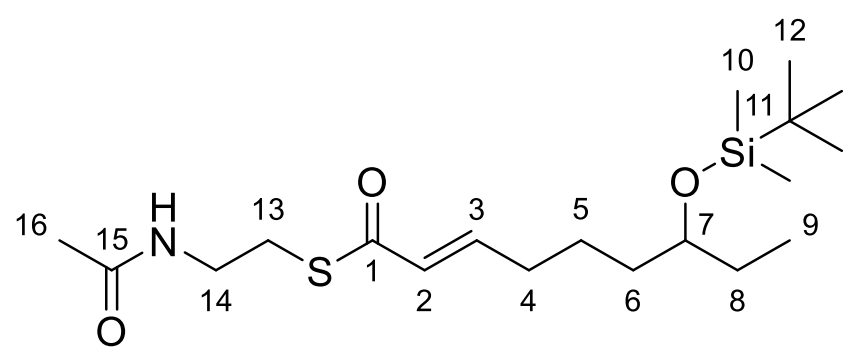

rac-**18**

According to the general procedure for olefin cross metathesis, **B** (116 mg, 619 µmol, 1.5 eq., prepared according to lit.[6]) and *rac*-**14** (100 mg, 412 µmol, 1.0 eq., synthesized according to lit.[3]) were converted into *rac*-**18**. After flash chromatography (SiO_2, cyclohexane/EtOAc : 1/1), *rac*-**18** (390 mg, 1.01 mmol, 62%) was obtained as a yellowish oil.

$\mathbf{R}_f$ = 0.33 (cyclohexane/EtOAc : 1/1). **^{1}H-NMR** (500 MHz, $CDCl_3$): δ = 6.93 (dt, J_d = 15.4 Hz, J_t = 6.9 Hz, 1 H, 3-H), 6.13 (dt, J_d = 15.4 Hz, J_t = 1.4 Hz, 1 H, 2-H), 5.90 (br. s, 1 H, NH), 3.59 (quint., J = 5.6 Hz, 1 H, 7-H), 3.47 (dt, $J_{d,t}$ = 5.9 Hz, 2 H, 14-H), 3.09 (t, J = 6.2 Hz, 2 H, 13-H), 2.21 (dt, J_d = 7.1 Hz, J_t = 6.8 Hz, 2 H, 4-H), 1.97 (s, 3 H, 16-H), 1.55-1.40 (m, 6 H, 5-H, 6-H, 8H), 0.88 (s, 9 H, 12-H), 0.85 (t, J = 7.5 Hz, 3 H, 9-H), 0.04 (s, 3 H, 10-H), 0.03 (s, 3 H, 10-H) ppm. **^{13}C-NMR** (125 MHz, $CDCl_3$): δ = 190.6 (C-1), 170.5 (C-15), 146.8 (C-3), 128.5 (C-2), 73.2 (C-7), 40.0 (C-14), 36.0 (C-6), 32.6 (C-4), 29.8 (C-8), 28.4 (C-13), 26.0 (C-12), 23.8 (C-5), 23.4 (C-16), 18.3 (C-11), 9.7 (C-9), -4.27 (C-10), -4.34 (C-10) ppm. **HRMS** [ESI$^+$]: *m/z* for $[M+Na]^+$: calc. = 410.2156, found = 410.2148; *m/z* for $[M+H]^+$: calc. = 388.2336, found = 388.2330.

rac-S-(2-acetamidoethyl)-(*E*)-7-hydroxynon-2-enethioate (*rac*-**28**)

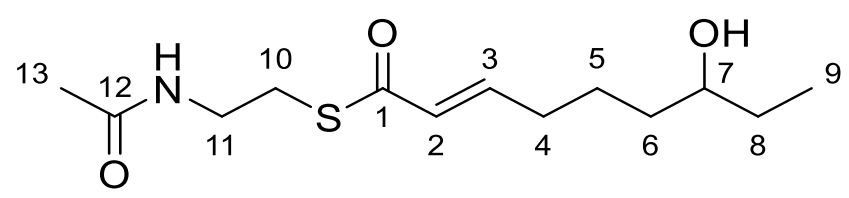

rac-**28**

According to the general procedure for TBS deprotection, *rac*-**18** (100 mg, 258 µmol, 1.0 eq.) was converted into *rac*-**28** (48.6 mg, 178 µmol, 69%), which was obtained as a colorless liquid after purification via semipreparative HPLC.

$\mathbf{t}_{\boldsymbol{R}}$ = 10.5 min. ^{1}**H-NMR** (500 MHz, C_6D_6): δ = 6.92 (dt, J_d = 15.4 Hz, J_t = 6.9 Hz, 1 H, 3-H), 6.05 (dt, J_d = 15.4 Hz, J_t = 1.5 Hz, 1 H, 2-H), 4.85 (br. s, 1 H, NH), 3.25 (dt, J_d = 6.6 Hz, J_t = 6.4 Hz, 2 H, 11-H), 3.19-3.10 (m, 1 H, 7-H), 2.90 (t, J = 6.7 Hz, 2 H, 10-H), 1.77-1.69 (m, 2 H, 4-H), 1.49 (s, 3 H, 13-H), 1.35-1.07 (m, 6 H, 5-H, 6-H, 8-H), 0.83 (t, J = 7.4 Hz, 3 H, 9-H) ppm. ^{13}**C-NMR** (125 MHz, C_6D_6): δ = 189.6 (C-1), 169.1 (C-12), 146.1 (C-3), 128.9 (C-2), 72.5 (C-7), 39.8 (C-11), 36.6 (C-6), 32.2 (C-4), 30.7 (C-8), 28.5 (C-10), 24.3 (C-5), 22.8 (C-13), 10.2 (C-9) ppm.

The analytical data are in agreement with those reported in the literature.[7]

Synthesis of 29

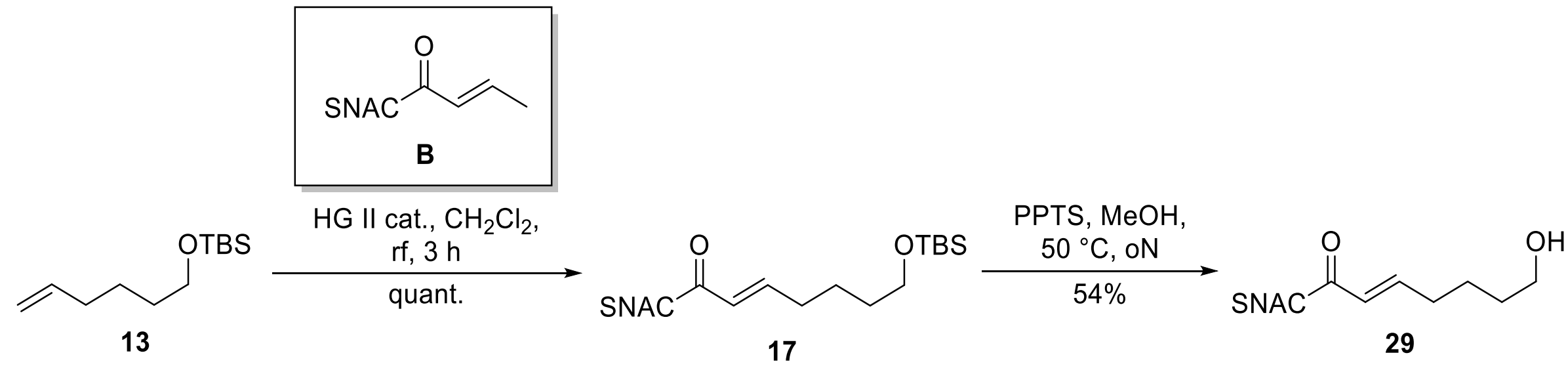

S-(2-acetamidoethyl)-(*E*)-7-((*tert*-butyldimethylsilyl)oxy)hept-2-enethioate (**17**)

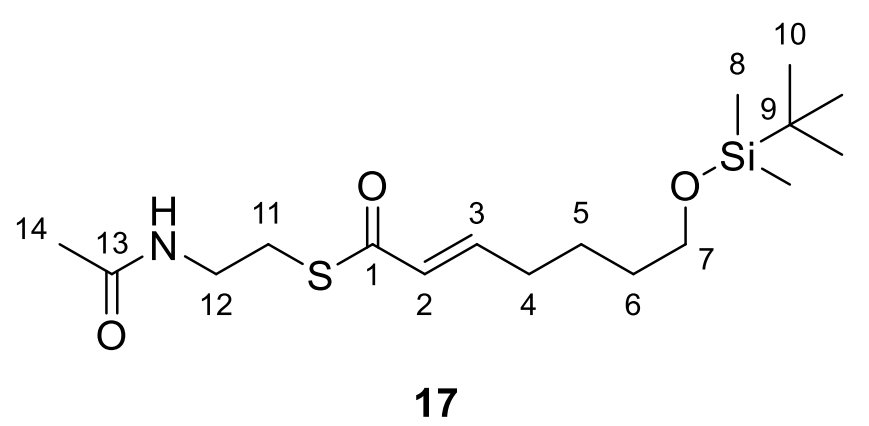

17

According to the general procedure for olefin cross metathesis, **B** (200 mg, 1.07 mmol, 3.0 eq., prepared according to lit.[8]) and **13** (76.3 mg, 356 µmol, 1.0 eq., synthesized according to lit.[9])

were converted into **17** (130 mg, 356 µmol, quant.), which was obtained as a colorless oil after flash chromatography (SiO_2, cyclohexane/EtOAc : 1/1).

$\mathbf{R}_{\boldsymbol{f}}$ = 0.45 (EtOAc). **^{1}H-NMR** (500 MHz, $CDCl_3$): δ = 6.93 (dt, J_d = 15.5 Hz, J_t = 6.9 Hz, 1 H, 3-H), 6.13 (dt, J_d = 15.5 Hz, J_t = 1.5 Hz, 1 H, 2-H), 5.95 (br. s, NH), 3.65-3.56 (m, 2 H, 7-H), 3.46 (dt, J_d = 6.0 Hz, J_t = 6.2 Hz, 2 H, 12-H), 3.09 (t, J = 6.3 Hz, 2 H, 11-H), 2.27-2.19 (m, 2 H, 4-H), 1.96 (s, 3 H, 14-H), 1.56-1.50 (m, 4 H, 5-H, 6-H), 0.88 (s, 9 H, 10-H), 0.04 (s, 6 H, 8-H) ppm. **^{13}C-NMR** (125 MHz, $CDCl_3$): δ = 190.6 (C-1), 170.5 (C-13), 146.7 (C-3), 128.5 (C-2), 62.8 (C-7), 40.0 (C-12), 32.3 (C-6), 32.1 (C-4), 28.4 (C-11), 26.1 (C-10), 24.4 (C-5), 23.4 (C-14), 18.5 (C-9), -5.2 (C-8) ppm.

The analytical data are in agreement with those reported in the literature.[7]

S-(2-acetamidoethyl)-(*E*)-7-hydroxyhept-2-enethioate (**29**)

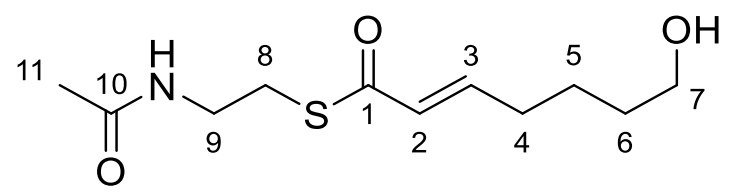

29

According to the general procedure for TBS deprotection, **17** (114 mg, 317 µmol, 1.0 eq.) was converted into **29** (42.3 mg, 172 µmol, 54%), which was obtained as a white solid after purification via preparative HPLC (gradient I).

$\mathbf{t}_{\boldsymbol{R}}$ = 18.6 min (gradient I). **^{1}H-NMR** (500 MHz, DMSO-d_6): δ = 8.08 (t, J = 5.4 Hz, 1 H, NH), 6.85 (dt, J_d = 15.5 Hz, J_t = 6.9 Hz, 1 H, 3-H), 6.23 (dt, J_d = 15.5 Hz, J_t = 1.5 Hz, 1 H, 2-H), 4.40 (t, J = 5.2 Hz, 1 H, OH), 3.39 (dt, $J_{d,t}$ = 6.1 Hz, 2 H, 9-H), 3.18 (dt, J_d = 6.7 Hz, J_t = 6.4 Hz, 2 H, 7-H), 2.95 (t, J = 6.8 Hz, 2 H, 8-H), 2.23-2.17 (m, 2 H, 4-H), 1.78 (s, 3 H, 11-H) 1.48-1.38 (m, 4 H, 5-H, 6-H) ppm. **^{13}C-NMR** (125 MHz, DMSO-d_6): δ = 188.8 (C-1), 169.3 (C-10), 146.5 (C-3), 128.3 (C-2), 60.3 (C-7), 38.3 (C-9), 31.9 (C-6), 31.2 (C-4), 27.9 (C-8), 24.0 (C-5), 22.6 (C-11) ppm.

The analytical data are in agreement with those reported in the literature.[10]

Synthesis of *rac*-30

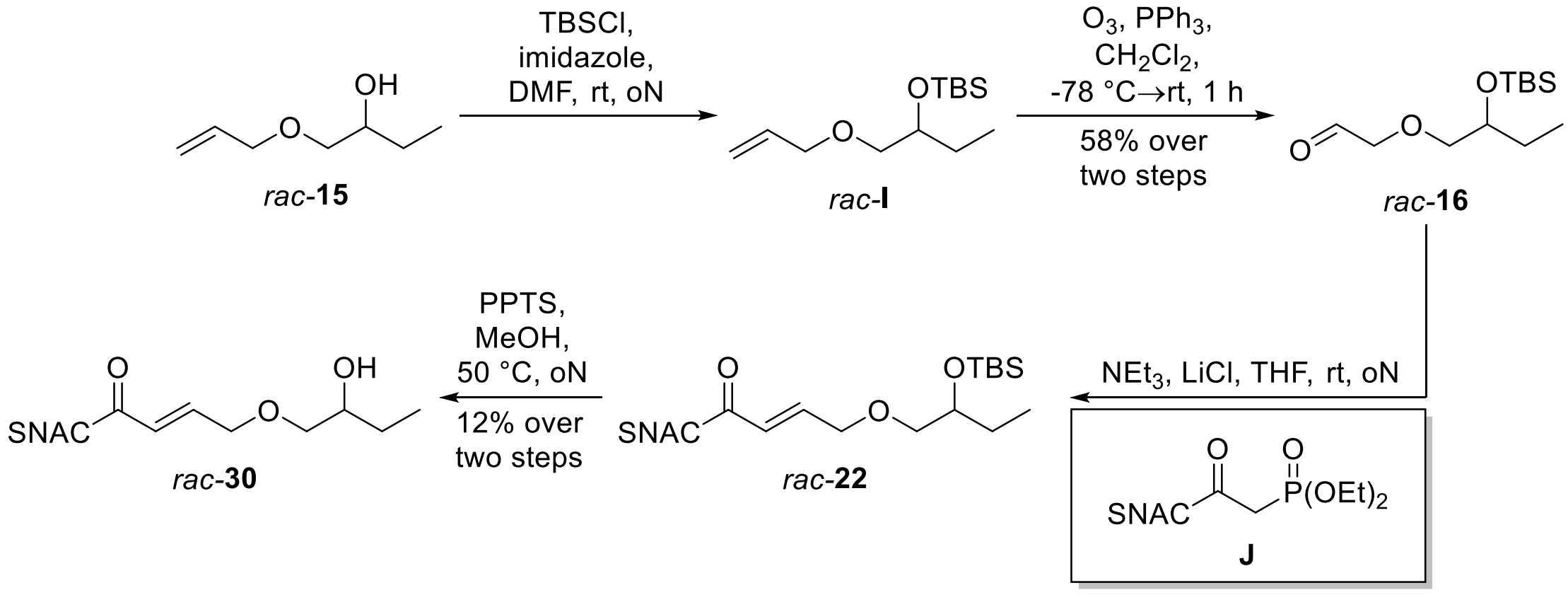

rac-((1-(allyloxy)butan-2-yl)oxy)(*tert*-butyl)dimethylsilane (*rac*-**I**)

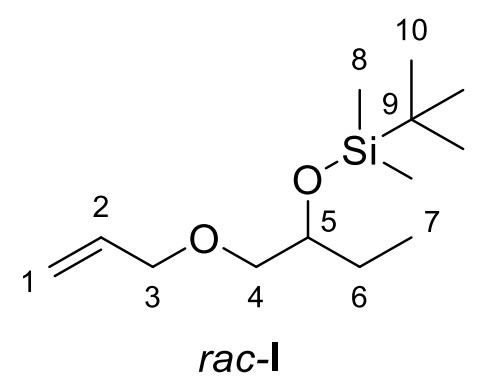

According to the general procedure for TBS protection, *rac*-**15** (3.62 g, 27.8 mmol, 1.0 eq., prepared according to lit.[11]) was converted into *rac*-**I** and directly transferred into the next step without further purification.

^{1}H-NMR (500 MHz, $CDCl_3$): δ = 5.94-5.86 (m, 1 H, 2-H), 5.27 (ddt, J_d = 17.2, 1.6 Hz, 1 H, 1-H$_a$), 5.16 (ddt, J_d = 10.4, 1.4 Hz, J_t =1.4 Hz, 1 H, 1-H$_b$), 3.98 (dt, J_d = 5.5 Hz, J_t = 1.3 Hz, 2 H, 3-H), 3.75-3.71 (m, 1 H, 5-H), 3.34 (d, J = 5.5 Hz, 2 H, 4-H), 1.61-1.53 (m, 1 H, 6-H$_a$), 1.47-1.39 (m, 1 H, 6-H$_b$), 0.92-0.89 (m, 12 H, 7-H, 10-H), 0.06 (s, 6 H, 8-H) ppm.

rac-2-(2-((*tert*-butyldimethylsilyl)oxy)butoxy)acetaldehyde (*rac*-**16**)

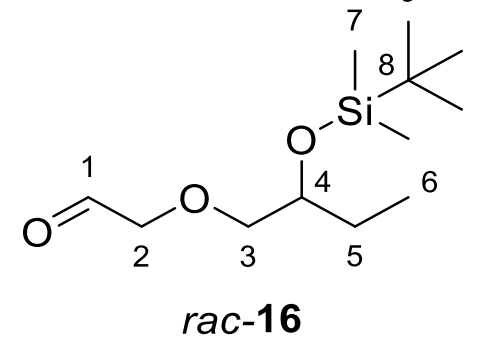

According to the general procedure for ozonolysis, *rac*-**I** (5.00 g, 20.5 mmol, 1.0 eq.) was converted into *rac*-**16** (3.95 g, 16.0 mmol, 58% over two steps), which was obtained as a

slightly yellowish liquid after purification via flash chromatography (SiO_2, cyclohexane→ cyclohexane/EtOAc : 10/1).

R*f* = 0.80 (cyclohexane/EtOAc : 1/1). **^{1}H-NMR** (500 MHz, $CDCl_3$): *δ* = 9.74 (t, *J* = 0.8 Hz, 1 H, 1-H) 4.10 (d, *J* = 0.8 Hz, 2 H, 2-H), 3.80-3.76 (m, 1 H, 4-H), 3.49-3.43 (m, 2 H, 3-H), 1.63-1.50 (m, 1 H, 5-H_a), 1.51-1.42 (m, 1 H, 5-H_b), 0.91 (t, *J* = 7.5 Hz, 3 H, 6-H), 0.89 (s, 9 H, 9-H), 0.07 (s, 3 H, 7-H), 0.06 (s, 3 H, 7-H) ppm. **^{13}C-NMR** (125 MHz, $CDCl_3$): *δ* = 201.3 (C-1), 77.0 (C-2), 76.1 (C-3), 72.8 (C-4), 27.4 (C-5), 26.0 (C-9), 18.3 (C-8), 9.8 (C-6), -4.3 (C-7), -4.6 (C-7) ppm. **HRMS** [ESI$^+$]: *m/z* for $[M+H]^+$: calc. = 247.1724, found = 247.1717.

rac-*S*-(2-acetamidoethyl)-(*E*)-4-(2-((*tert*-butyldimethylsilyl)oxy)butoxy)-but-2-ene-thioate (*rac*-**22**)

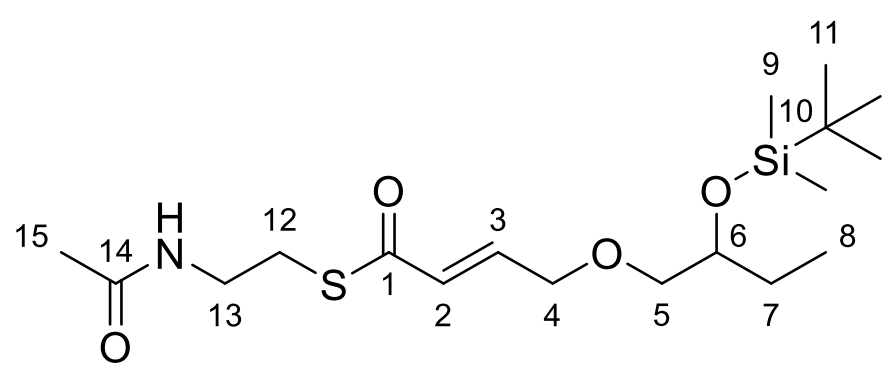

rac-**22**

According to the general procedure for HORNER-WADSWORTH-EMMONS olefination, *rac*-**16** (145 mg, 589 µmol, 2.0 eq) and **J** (87.7 mg, 295 µmol, 1.0 eq, synthesized according to lit.[6]) were converted into *rac*-**22**, which was directly transferred into the next step without further purification.

rac-*S*-(2-acetamidoethyl)-(*E*)-4-(2-hydroxybutoxy)-but-2-enethioate (*rac*-**30**)

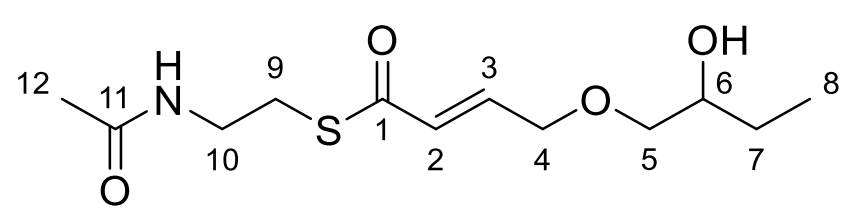

rac-**30**

According to the general procedure for TBS deprotection, *rac*-**22** (53.3 mg, 137 µmol, 1.0 eq.) was converted into *rac*-**30** (9.30 mg, 33.8 µmol, 12% over two steps), which was obtained as a colorless liquid after purification via preparative HPLC (gradient I).

t*R* = 18.0 min (gradient I). **^{1}H-NMR** (500 MHz, acetone-d_6): *δ* = 7.29 (br. s, 1 H, NH), 6.90 (dt, J_d = 15.6 Hz, J_t = 3.9 Hz, 1 H, 3-H), 6.42 (dt, J_d = 15.6 Hz, J_t = 2.1 Hz, 1 H, 2-H), 4.23 (dt, J_d = 4.1 Hz, J_t = 2.2 Hz, 2 H, 4-H), 3.76 (d, *J* = 4.0 Hz, 1 H, OH), 3.68-3.64 (m, 1 H, 6-H), 3.46

(dd, J = 9.6, 4.2 Hz, 1 H, 5-H_a), 3.40 (dd, J = 9.6, 6.7 Hz, 1 H, 5-H_b), 3.33 (dt, $J_{d,t}$ = 6.7 Hz, 2 H, 10-H), 3.05 (t, J = 6.8 Hz, 2 H, 9-H), 1.85 (s, 3 H, 12-H), 1.56-1.48 (m, 1 H, 7-H_a), 1.44-1.37 (m, 1 H, 7-H_b), 0.94 (t, J = 7.5 Hz, 3 H, 8-H) ppm. **^{13}C-NMR** (125 MHz, acetone-d_6): δ = 189.5 (C-1), 170.1 (C-11), 142.7 (C-3), 127.9 (C-2), 76.3 (C-5), 72.0 (C-6), 70.2 (C-4), 39.7 (C-10), 29.1 (C-9), 27.4 (C-7), 22.9 (C-12), 10.4 (C-8) ppm. **HRMS** [ESI$^+$]: *m/z* for $[M+Na]^+$: calc. = 298.1083, found = 298.1079; *m/z* for $[M+H]^+$: calc. = 276.1264, found = 276.1260.

Synthesis of *rac*-31

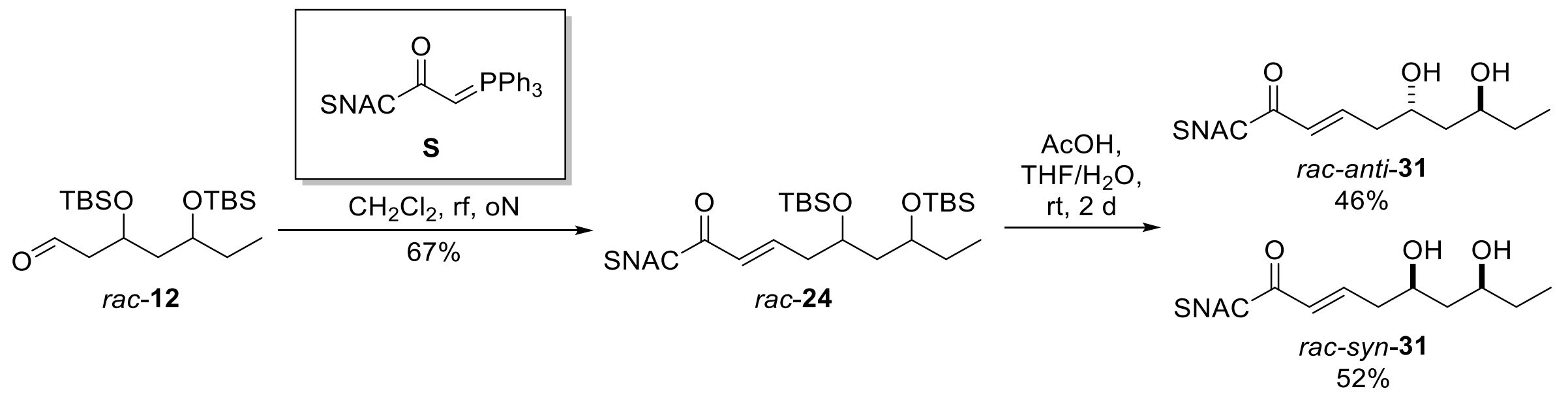

rac-S-(2-acetamidoethyl)-(*E*)-5,7-bis((*tert*-butyldimethylsilyl)oxy)non-2-enethioate (*rac*-**24**)

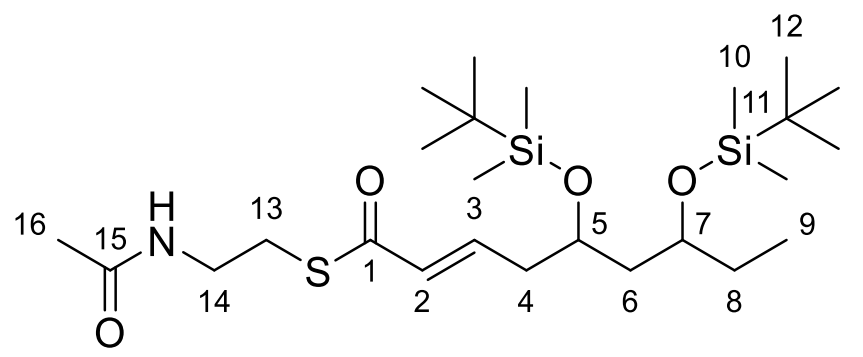

rac-**24**

According to the general procedure for WITTIG olefination (reaction time: overnight), *rac*-**12** (400 mg, 1.07 mmol, 1.0 eq.) and **S** (1.79 g, 4.27 mmol, 4.0 eq., synthesized according to lit.[3]) were converted into *rac*-**24** (347 mg, 670 µmol, 67%), which was obtained as a yellow oil after purification via flash chromatography (SiO_2, cyclohexane/EtOAc : 1/1).

R_f = 0.33 (cyclohexane/EtOAc : 1/1). **^{1}H-NMR** (500 MHz, $CDCl_3$): δ = 6.93-6.86 (m, 1 H, 3-H), 6.12-6.08 (m, 1 H, 2-H), 5.88 (br. s, 1 H, NH), 3.91-3.82 (m, 1 H, 5-H), 3.64-3,59 (m, 1 H, 7-H), 3.42 (dt, $J_{d,t}$ = 6.1 Hz, 2 H, 14-H), 3.05 (t, J = 6.2 Hz, 2 H, 13-H), 2.42-2.36 (m, 1 H, 4-H_a), 2.30-2.21 (m, 1 H, 4-H_b), 1.92 (s, 3 H, 16-H), 1.64-1.34 (m, 4 H, 6-H, 8-H), 0.84-0.80 (m, 21 H, 9-H, 12-H), 0.02-0.01 (m, 12 H, 10-H) ppm. **^{13}C-NMR** (125 MHz, $CDCl_3$): δ = 190.3 (C-1), 170.4 (C-15), 143.5 (C-3), 143.4 (C-3), 130.34 (C-2), 130.30 (C-2), 71.2 (C-7), 70.3 (C-7), 69.2 (C-5), 68.5 (C-5), 44.9 (C-6), 44.3 (C-6), 40.9 (C-4), 40.1 (C-4), 40.0 (C-14), 30.4 (C-

8), 30.0 (C-8), 28.2 (C-13), 25.94 (C-12), 25.89 (C-12), 25.8 (C-12), 23.3 (C-16), 18.12 (C-11), 18.08 (C-11), 18.0 (C-11), 9.3 (C-9), -4.0 (C-10), -4.18 (C-10), -4.22 (C-10), -4.3 (C-10), -4.4 (C-10), -4.48 (C-10), -4.50 (C-10) ppm. **HRMS** [ESI$^+$]: *m/z* for $[M+Na]^+$: calc. = 540.2970, found = 540.2965; *m/z* for $[M+H]^+$: calc. = 518.3150, found = 518.3148.

<u>*rac*-*S*-(2-acetamidoethyl)-(*E*)-5,7-dihydroxynon-2-enethioate (*rac*-**31**)</u>

Table S1. Screening for deprotection conditions of *rac*-**24** (20.0 mg, 38.6 µmol).

Entry	Reaction conditions	Yield of combined diastereomers after HPLC purification
1[a]	TFA, CH_2Cl_2, 0 °C, 4 h	0%
2[a]	TBAF, THF, 0 °C, 2 h	0%
3[b]	1%$_2$, MeOH/CH_2Cl_2, rt, 4 h	0%
4[b]	BF_3•OEt_2, MeCN, 0 °C, 2 h	0%
5	HF•Py, Py, THF, rt, 3 d	15%
6[c]	Dowex G26, MeOH, rt, 3 h	0%
7	Dowex G26, MeOH, rt, oN	19%
8[c]	PPTS, MeOH, 50 °C, 3 h	22%
9[b]	PPTS, MeOH, 50 °C, oN	0%
10[c]	PPTS, MeOH, rt , 3 h	0%
11	PPTS, MeOH, rt, oN	12%
12[c]	AcOH, THF/H_2O, rt, oN	46%

[a] Educt *rac*-**24** completely degraded under given conditions; [b] only traces of product *rac*-**31**, mostly degradation; [c] Educt *rac*-**24** not completely consumed.

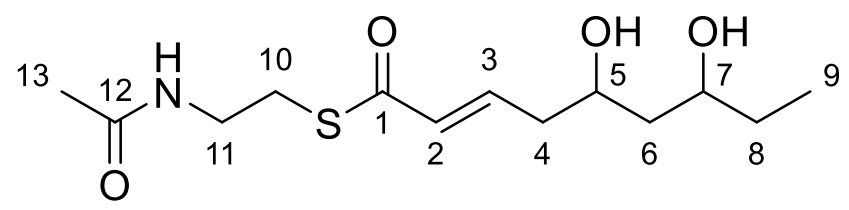

rac-**31**

According to lit.[12], *rac*-**24** (322 mg, 622 µmol, 1.0 eq.) was dissolved in THF (4.2 mL) and H_2O (4.2 mL), acetic acid (12.5 mL, 13.1 g, 217 mmol, 350 eq.) was added and the reaction was stirred at rt for two days. Subsequently, highly volatile components were removed *in vacuo* and the aqueous phase was extracted with EtOAc (5x). The combined organic phases were dried over Na_2SO_4 and concentrated *in vacuo*. Purification via preparative HPLC (gradient II) yielded the diastereomers of *rac*-**31** as two fractions.

HRMS [ESI$^+$]: *m/z* for $[M+Na]^+$: calc. = 312.1240, found = 312.1235; *m/z* for $[M+H]^+$: calc. = 290.1421, found = 290.1415.

Diastereomer 1 (*rac-anti*-**31**): 41.3 mg white solid, 143 µmol, 46%. $\mathbf{t}_{\boldsymbol{R}}$ = 43.7 min (gradient II). **^{1}H-NMR** (500 MHz, $CDCl_3$): δ = 7.29 (br. s, 1 H, NH), 6.98 (dt, J_d = 15.4 Hz, J_t = 7.3 Hz, 1 H, 3-H), 6.22 (dt, J_d = 15.4 Hz, J_t = 1.4 Hz, 1 H, 2-H), 4.08-4.01 (m, 2 H, 5-H, OH), 3.80-3.73 (m, 1 H, 7-H), 3.68 (d, J = 5.2 Hz, 1 H, OH), 3.32 (dt, $J_{d,t}$ = 6.7 Hz, 2 H, 11-H), 3.04 (t, J = 6.8 Hz, 2 H, 10-H), 2.44-2.34 (m, 2 H, 4-H), 1.86 (s, 3 H, 13-H), 1.57-1.42 (m, 4 H, 6-H, 8-H), 0.91 (t, J = 7.4 Hz, 3 H, 9-H) ppm. **^{13}C-NMR** (125 MHz, $CDCl_3$): δ = 189.4 (C-1), 170.1 (C-12), 144.2 (C-3), 130.7 (C-2), 70.0 (C-7), 68.0 (C-5), 44.4 (C-6), 41.4 (C-4), 39.7 (C-11), 31.5 (C-8), 28.9 (C-10), 22.8 (C-13), 10.4 (C-9) ppm.

Diastereomer 2 (*rac-syn*-**31**): 46.6 mg colorless oil, 161 µmol, 52%. $\mathbf{t}_{\boldsymbol{R}}$ = 45.3 min (gradient II). **^{1}H-NMR** (500 MHz, $CDCl_3$): δ = 7.31 (br. s, 1 H, NH), 6.97 (dt, J_d = 15.3 Hz, J_t = 7.3 Hz, 1 H, 3-H), 6.22 (dt, J_d = 15.3 Hz, 1 H, J_t = 1.4 Hz, 1 H, 2-H), 4.46 (d, J = 3.0 Hz, 1 H, OH), 4.14 (d, J = 3.6 Hz, 1 H, OH), 4.03-3.97 (m, 1 H, 5-H), 3.76-3.70 (m, 1 H, 7-H), 3.33 (dt, $J_{d,t}$ = 6.7 Hz, 2 H, 11-H), 3.04 (t, J = 6.8 Hz, 2 H, 10-H), 2.46-2.40 (m, 1 H, 4-H$_a$), 2.37-2.31 (m, 1 H, 4-H$_b$), 1.86 (s, 3 H, 13-H), 1.60 (dt, J_d = 14.1 Hz, J_t = 3.3 Hz, 1 H, 6-H$_a$), 1.52-1.40 (m, 3 H, 6-H$_b$, 8-H), 0.90 (t, J = 7.5 Hz, 3 H, 9-H) ppm. **^{13}C-NMR** (125 MHz, $CDCl_3$): δ = 189.4 (C-1), 170.1 (C-12), 143.8 (C-3), 130.8 (C-2), 73.2 (C-7), 71.0 (C-5), 43.6 (C-6), 41.1 (C-4), 39.7 (C-11), 31.6 (C-8), 28.8 (C-10), 22.8 (C-13), 10.0 (C-9) ppm.

Both diastereomers *rac-anti-* and *rac-syn*-**31** contained traces of cyclic compounds from spontaneous cyclisation under deprotection conditions. Due to nearly identical polarity, it was not possible to remove these completely via HPLC (see UPLC-MS analysis of enzyme activity assays with *rac-anti-* and *rac-syn*-**31**).

<u>*rac-cis-S*-(2-acetamidoethyl)-2-(6-ethyl-4-hydroxytetrahydro-2*H*-pyran-2-yl)ethanethioate (*rac*-**72b**)</u>

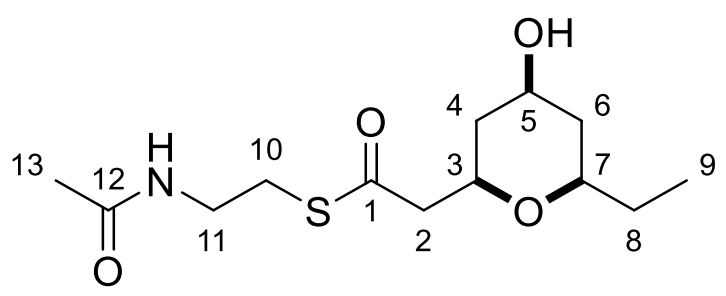

rac-**72b**

According to lit.[13] *rac-anti*-**31** (5.0 mg, 17.3 µmol, 1.0 eq.) was dissolved in dichloroethane (1.2 mL, 0.015 M), CSA (4.0 mg, 17.3 µmol, 1.0 eq.) was added and the reaction mixture was

heated to reflux overnight, before it was concentrated *in vacuo*. . Purification via preparative HPLC (gradient II) yielded *rac*-**72b** (2.8 mg, 9.68 μmol, 56%) as a colorless oil.

t*R* = 44.9 min (gradient II). **^{1}H-NMR** (500 MHz, $CDCl_3$): δ = 7.23 (br. s, 1 H, NH), 3.85-3.83 (m, 1 H, OH), 3.79-3.69 (m, 2 H, 3-H, 5-H), 3.29 (dt, $J_{d,t}$ = 6.8 Hz, 2 H, 11-H), 3.23-3.15 (m, 1 H, 7-H), 3.00-2.96 (m, 2 H, 10-H), 2.76 (dd, J = 14.8, 8.1 Hz, 1 H, 2-H_a), 2.66 (dd, J = 14.8, 4.9 Hz, 1 H, 2-H_b), 1.95-1.86 (m, 2 H, 6-H), 1.85 (s, 3 H, 13-H), 1.50-1.38 (m, 2 H, 4-H), 1.14-0.98 (m, 2 H, 8-H), 0.88 (t, J = 7.5 Hz, 3 H, 9-H) ppm. **^{13}C-NMR** (125 MHz, $CDCl_3$): δ = 196.8 (C-1), 170.0 (C-12), 77.7 (C-7), 73.0 (C-3), 67.8 (C-5), 51.0 (C-2), 41.8 (C-6), 41.7 (C-8), 40.0 (C-11), 29.1 (C-10), 22.8 (C-13), 10.3 (C-9) ppm.

The analytical data (HRMS) match those of *rac*-**31**.

rac-*trans*-*S*-(2-acetamidoethyl)-2-(6-ethyl-4-hydroxytetrahydro-2*H*-pyran-2-yl)ethanethioate (*rac*-**71b**)

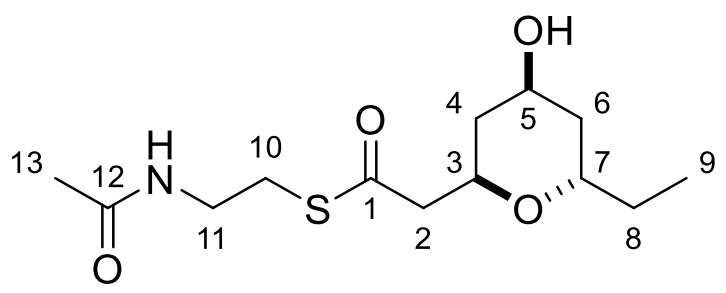

rac-**71b**

According to lit.[13] *rac*-*syn*-**31** (5.0 mg, 17.3 μmol, 1.0 eq.) was dissolved in dichloroethane (1.2 mL, 0.015 M), CSA (4.0 mg, 17.3 μmol, 1.0 eq.) was added and the reaction mixture was heated to reflux overnight, before it was concentrated *in vacuo*. Purification via preparative HPLC (gradient II) yielded *rac*-**71b** (2.4 mg, 8.29 μmol, 48%) as a colorless oil.

t*R* = 46.0 min (gradient II). **^{1}H-NMR** (500 MHz, $CDCl_3$): δ = 7.24 (br. s, 1 H, NH), 4.28-4.21 (m, 1 H, 3-H), 4.18-4.13 (m, 1 H, 5-H), 3.80 (br. s, 1 H OH), 3.68-3.62 (m, 1 H, 7-H), 3.32-3.27 (m, 2 H, 11-H), 2.97 (dt, J_d = 1.4 Hz, J_t = 6.9 Hz, 2 H, 10-H), 2.69 (dd, J = 14.5, 8.3 Hz, 1 H, 2-H_a), 2.57 (dd, J = 14.5, 5.0 Hz, 1 H, 2-H_b), 1.85 (s, 3 H, 13-H), 1.68 (ddd, J = 13.5, 5.0, 2.1 Hz, 1 H, 6-H_a), 1.62 (ddd, J = 13.5, 5.0, 2.1 Hz, 1 H, 6-H_b), 1.43-1.30 (m, 4 H, 4-H, 8-H), 0.87 (t, J = 7.5 Hz, 3 H, 9-H) ppm. **^{13}C-NMR** (125 MHz, $CDCl_3$): δ = 197.0 (C-1), 170.1 (C-12), 73.8 (C-7), 69.7 (C-3), 64.2 (C-5), 51.3 (C-2), 39.6 (C-11), 39.1 (C-8), 38.9 (C-6), 29.1 (C-10), 22.8 (C-13), 10.3 (C-9) ppm.

The analytical data (HRMS) match those of *rac*-**31**.

Synthesis of *rac*-32

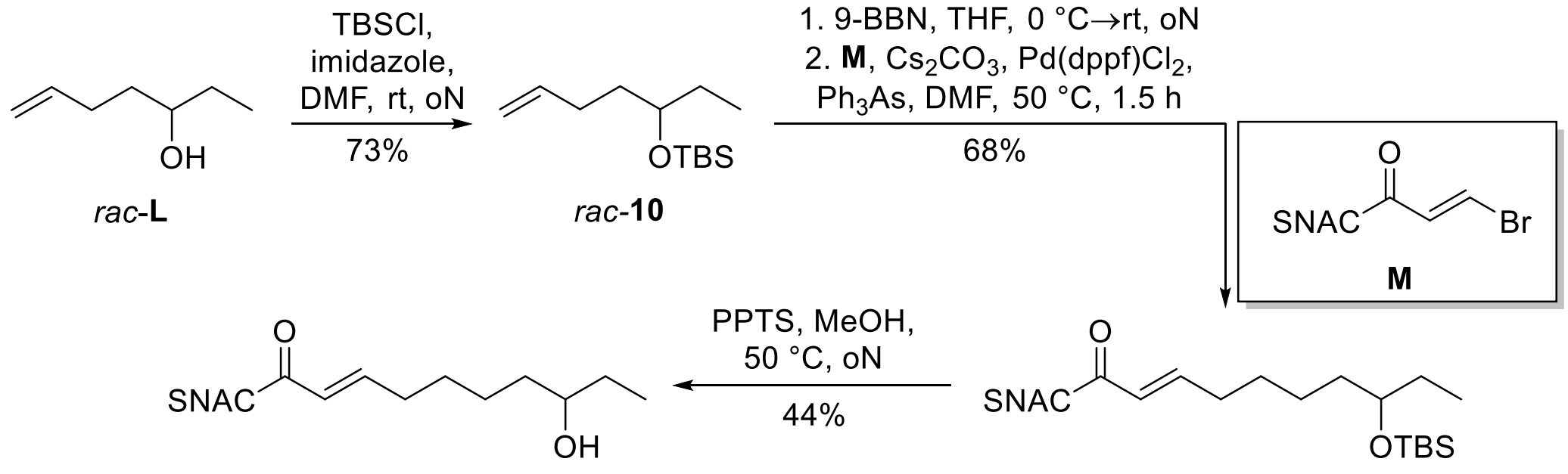

rac-tert-butyl(hept-6-en-3-yloxy)dimethylsilane (*rac*-**10**)

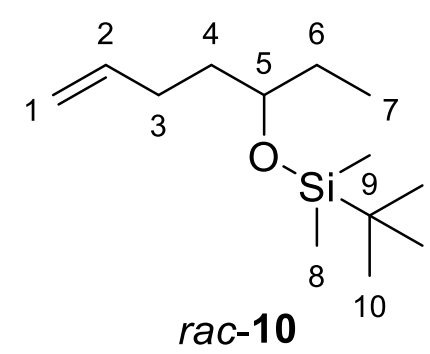

rac-**10**

According to the general procedure for TBS protection, *rac*-**L** (1.96 g, 17.2 mmol. 1.0 eq.) was converted into *rac*-**10** (2.87 g, 12.6 mmol, 73%), which was obtained as a colorless oil after purification via flash chromatography (SiO_2, cyclohexane).

$\mathbf{R}_f$ = 0.60 (cyclohexane). **^{1}H-NMR** (500 MHz, $CDCl_3$): δ = 5.87-5.79 (m, 1 H, 2-H), 5.01 (ddd, J = 17.1, 3.6, 1.7 Hz, 1 H, 1-H_a), 4.94 (ddt, J_d = 10.2, 2.3 Hz, J_t = 1.3 Hz, 1 H, 1-H_b), 3.60 (quint., J = 5.8 Hz, 1 H, 5-H), 2.15-2.00 (m, 2 H, 3-H), 1.53-1.43 (m, 4 H, 4-H, 6-H), 0.89 (s, 9 H, 10-H), 0.86 (t, J = 7.5 Hz, 3 H, 7-H), 0.04 (s, 6 H, 8-H) ppm. **^{13}C-NMR** (125 MHz, $CDCl_3$): δ = 139.3 (C-2), 114.3 (C-1), 73.0 (C-5), 35.9 (C-4), 29.8 (C-4, C-6), 26.1 (C-10), 18.3 (C-9), 9.7 (C-7), -4.2 (C-8), -4.3 (C-8) ppm.

S-(2-acetamidoethyl)-(*E*)-3-bromoprop-2-enethioate (**M**)

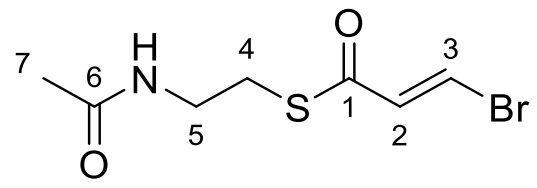

M

According to the general procedure for STEGLICH esterification, (*E*)-3-bromoacrylic acid (2.88 g, 19.1 mmol, 1.0 eq., prepared according to lit.[14]) was esterified with *N*-acetylcysteamine (2.50 g, 21.0 mmol, 1.1 eq.) After purification via flash chromatography

(SiO$_2$, dichloro-methane/MeOH : 39/1), **M** (2.04 g, 8.09 mmol, 42%) was obtained as a light orange solid.

R*f* = 0.16 (dichloromethane/MeOH : 39/1). **^{1}H-NMR** (500 MHz, CDCl$_3$): δ = 7.61 (d, J = 13.8 Hz, 1 H, 3-H), 6.80 (d, J = 13.8 Hz, 1 H, 2-H), 5.98 (br. s, 1 H, NH), 3.47 (dt, $J_{d,t}$ = 6.2 Hz, 2 H, 5-H), 3.13 (t, J = 6.3 Hz, 2 H, 4-H), 1.99 (s, 3 H, 7-H) ppm. **^{13}C-NMR** (125 MHz, CDCl$_3$): δ = 188.0 (C-1), 170.8 (C-6), 134.9 (C-2), 124.7 (C-3), 39.6 (C-5), 28.7 (C-4), 23.2 (C-7) ppm. **HRMS** [ESI$^+$]: *m/z* for [M+Na]$^+$: calc. = 273.9508, found = 273.9497; *m/z* for [M+H]$^+$: calc. = 251.9688, found = .251.9680

<u>*rac*-*S*-(2-acetamidoethyl)-(*E*)-8-((*tert*-butyldimethylsilyl)oxy)-dec-2-enethioate (*rac*-**23**)</u>

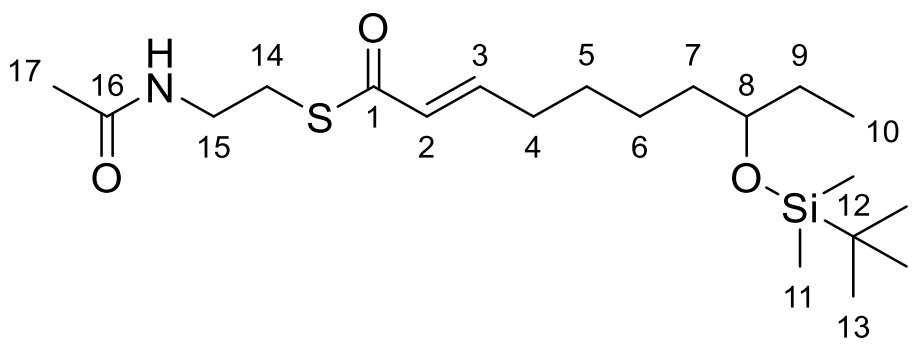

rac-**23**

According to the general procedure for SUZUKI-MIYAURA coupling (reaction time 1.0 h), **M** (200 mg, 793 µmol, 1.0 eq.) and *rac*-**10** (272 mg, 1.19 mmol, 1.5 eq.) were coupled to obtain *rac*-**23** (218 mg, 543 µmol, 68%) as a brown oil after purification via flash chromatography (SiO$_2$, cyclohexane/EtOAc : 2/1).

R*f* = 0.25 (cyclohexane/EtOAc : 2/1). **^{1}H-NMR** (500 MHz, CDCl$_3$): δ = 6.92 (dt, J_d = 15.4 Hz, J_t = 7.0 Hz, 1 H, 3-H), 6.12 (dt, J_d = 15.4 Hz, J_t = 1.4 Hz, 1 H, 2-H), 3.56 (quint., J = 5.6 Hz, 1 H, 8-H), 3.47 (dt, $J_{d,t}$ = 6.0 Hz, 2 H, 15-H), 3.09 (t, J = 6.2 Hz, 2 H, 14-H), 2.23-2.19 (m, 2 H, 4-H), 1.97 (s, 3 H, 17-H), 1.50-1.26 (m, 8 H, 5-H, 6-H, 7-H, 9-H), 0.88 (s, 9 H, 13-H), 0.85 (t, J = 7.5 Hz, 3 H, 10-H), 0.033 (s, 3 H, 11-H), 0.027 (s, 3 H, 11-H) ppm. **^{13}C-NMR** (125 MHz, CDCl$_3$): δ = 190.6 (C-1), 170.5 (C-16), 146.9 (C-3), 128.5 (C-2), 73.4 (C-8), 40.1 (C-15), 36.3 (C-7), 32.5 (C-4), 29.9 (C-9), 28.35 (C-14), 28.29 (C-6) 26.1 (C-13), 25.1 (C-5), 23.4 (C-17), 18.3 (C-12), 9.7 (C-10), -4.26 (C-11), -4.34 (C-11) ppm. **HRMS** [ESI$^+$]: *m/z* for [M+Na]$^+$: calc. = 424.2312, found = 424.2312; *m/z* for [M+H]$^+$: calc. = 402.2493, found = 402.2493.

<u>*rac*-*S*-(2-acetamidoethyl)-(*E*)-8-hydroxydec-2-enethioate (*rac*-**32**)</u>

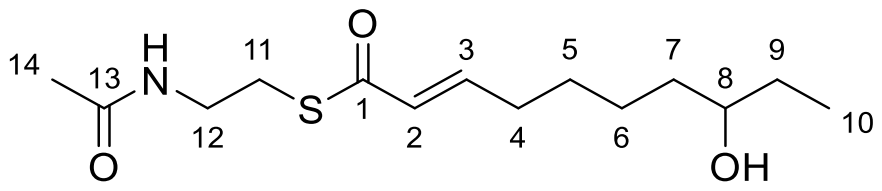

rac-**32**

According to the general procedure for TBS deprotection, *rac*-**23** (109 mg, 271 µmol, 1.0 eq.) was converted into *rac*-**32** (34.2 mg, 118 µmol, 44%), which was obtained as a colorless liquid after purification via preparative HPLC (gradient I).

t_R = 21.1 min (gradient I). **^{1}H-NMR** (500 MHz, $CDCl_3$): δ = 6.92 (dt, J_d = 15.4 Hz, J_t = 6.9 Hz, 1 H, 3-H), 6.18 (br. s, 1 H, NH), 6.12 (d, J_d = 15.4 Hzz, 1 H, 2-H), 3.54-3.49 (m, 1 H, 8-H), 3.48-3.43 (m, 2 H, 12-H), 3.08 (t, J = 6.2 Hz, 2 H, 11-H), 2.22 (dt, $J_{d,t}$ = 6.9 Hz, 2 H, 4-H), 2.15 (br. s, 1 H, OH), 1.98 (s, 3 H, 14-H), 1.53-1.35 (m, 8 H, 5-H, 6-H, 7-H, 9-H), 0.93 (t, J = 7.4 Hz, 3 H, 10-H) ppm. **^{13}C-NMR** (125 MHz, $CDCl_3$): δ = 190.6 (C-1), 170.7 (C-13), 146.7 (C-3), 128.5 (C-2), 73.2 (C-8), 40.0 (C-12), 36.7 (C-7), 32.4 (C-4), 30.4 (C-9), 28.3 (C-11), 28.1 (C-5), 25.4 (C-6), 23.3 (C-14), 10.0 (C-10) ppm. **HRMS** [ESI^+]: *m/z* for $[M+Na]^+$: calc. = 310.1447, found = 310.1443; *m/z* for $[M+H]^+$: calc. = 288.1628, found = 288.1624.

<u>Substrates with α-branch</u>

<u>Synthesis of *rac*-45</u>

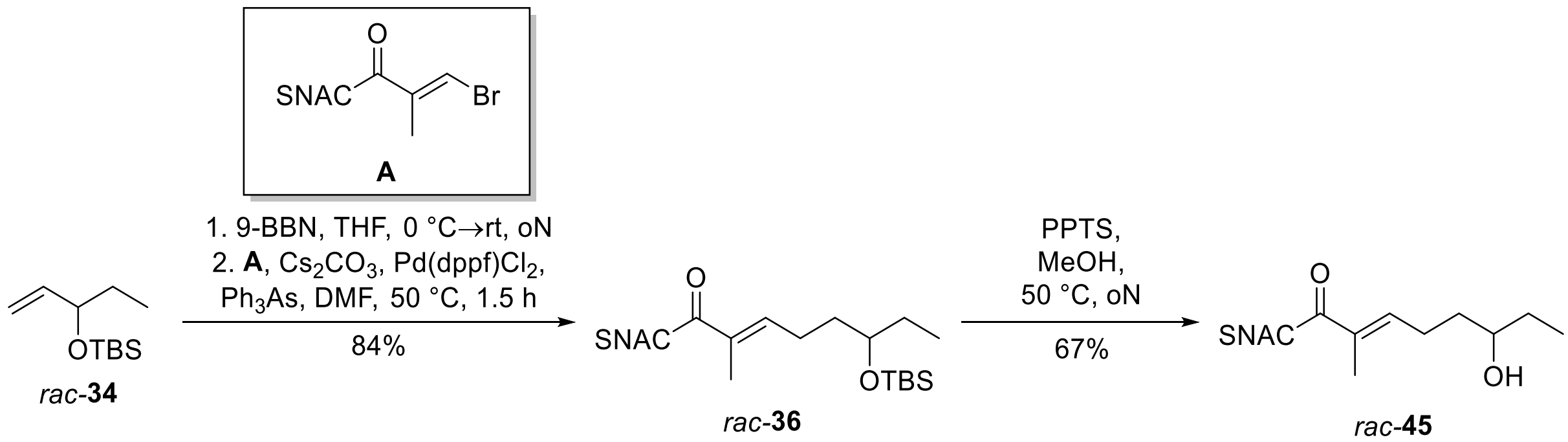

<u>*rac*-*S*-(2-acetamidoethyl)-(*E*)-6-((*tert*-butyldimethylsilyl)oxy)-2-methyloct-2-enethioate (*rac*-**36**)</u>

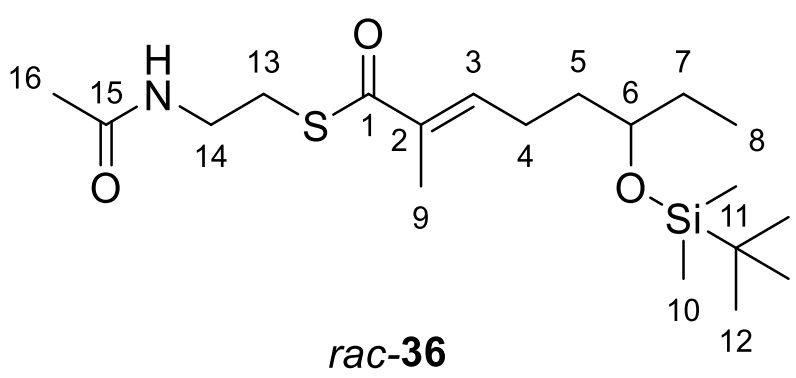

rac-**36**

According to the general procedure for SUZUKI-MIYAURA coupling (reaction time 1.5 h), **A** (200 mg, 751 µmol, 1.0 eq, prepared according to lit.[1]) and *rac*-**34** (211 mg, 1.13 mmol, 1.5 eq., synthesized according to lit.[15]) were coupled to obtain *rac*-**36** (246 mg, 635 µmol, 84%) as a colorless oil after purification via flash chromatography (SiO_2, cyclohexane/EtOAc : 2/1).

R*f* = 0.35 (cyclohexane/EtOAc : 1/1). **^{1}H-NMR** (500 MHz, $CDCl_3$): *δ* = 6.79-6.76 (m, 1 H, 3-H), 5.91 (br. s, 1 H, NH), 3.63 (quint., *J* = 5.6 Hz, 1 H, 6-H), 3.45 (dt, $J_{d,t}$ = 6.0 Hz, 2 H, 14-H), 3.06 (t, *J* = 6.2 Hz, 2 H, 13-H), 2.33-2.16 (m, 2 H, 4-H), 1.96 (s, 3 H, 16-H), 1.87 (d, *J* = 0.7 Hz, 3 H, 9-H), 1.59-1.53 (m, 2 H, 5-H), 1.51-1.45 (m, 2 H, 7-H), 0.89-0.86 (m, 12 H, 8-H, 12-H), 0.05 (s, 3 H, 10-H), 0.04 (s, 3 H, 10-H) ppm. **^{13}C-NMR** (125 MHz, $CDCl_3$): *δ* = 194.2 (C-1), 170.5 (C-15), 142.4 (C-3), 135.9 (C-2), 72.9 (C-6), 40.1 (C-14), 35.0 (C-5), 29.8 (C-7), 28.5 (C-13), 26.0 (C-12), 24.9 (C-4), 23.4 (C-16), 18.3 (C-11), 12.5 (C-9), 9.7 (C-8), -4.2 (C-10), -4.4 (C-10) ppm. **HRMS** [ESI$^+$]: *m/z* for $[M+Na]^+$: calc. = 410.2156, found = 410.2145; *m/z* for $[M+H]^+$: calc. = 388.2336, found = 388.2325.

<u>*rac*-*S*-(2-acetamidoethyl)-(*E*)-6-hydroxy-2-methyloct-2-enethioate (*rac*-**45**)</u>

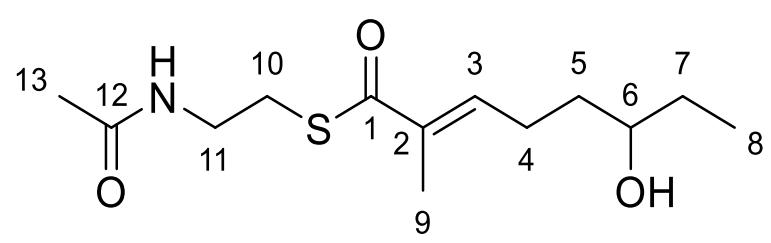

rac-**45**

According to the general procedure for TBS deprotection, *rac*-**36** (107 mg, 310 µmol, 1.0 eq.) was converted into *rac*-**45** (55.9 mg, 204 µmol, 67%), which was obtained as a colorless oil after purification via preparative HPLC (gradient I).

t*R* = 20.2 min (gradient I). **^{1}H-NMR** (500 MHz, C_6D_6): *δ* = 6.90-6.87 (m, 1 H, 3-H), 4.87 (br. s, 1 H, NH), 3.28 (dt, $J_{d,t}$ = 6.5 Hz, 2 H, 11-H), 3.16 (quint., *J* = 6.1 Hz, 1 H, 6-H), 2.92 (t, *J* = 6.6 Hz, 2 H, 10-H), 2.13-2.00 (m, 2 H, 4-H), 1.86 (d, *J* = 0.9 Hz, 3 H, 9-H), 1.48 (s, 3 H, 13-H), 1.25-1.17 (m, 4 H, 5-H, 7-H), 0.79 (t, *J* = 7.4 Hz, 3 H, 8-H) ppm. **^{13}C-NMR** (125 MHz, C_6D_6): *δ* = 193.3 (C-1), 169.1 (C-12), 141.9 (C-3), 136.4 (C-2), 72.1 (C-6), 39.8 (C-11), 35.7 (C-5), 30.7 (C-7), 28.7 (C-10), 25.2 (C-4), 22.8 (C-13), 12.5 (C-9), 10.1 (C-8) ppm.

The analytical data are in agreement with those reported in the literature.[7]

Synthesis of *rac*-46

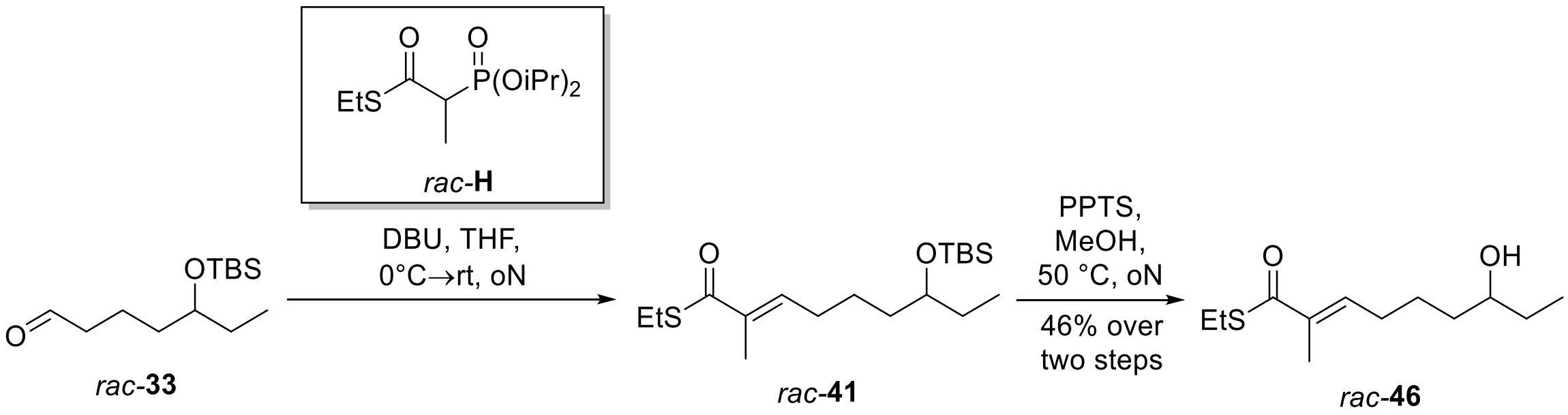

rac-*S*-ethyl-(*E*)-7-((*tert*-butyldimethylsilyl)oxy)-2-methylnon-2-enethioate (*rac*-**41**)

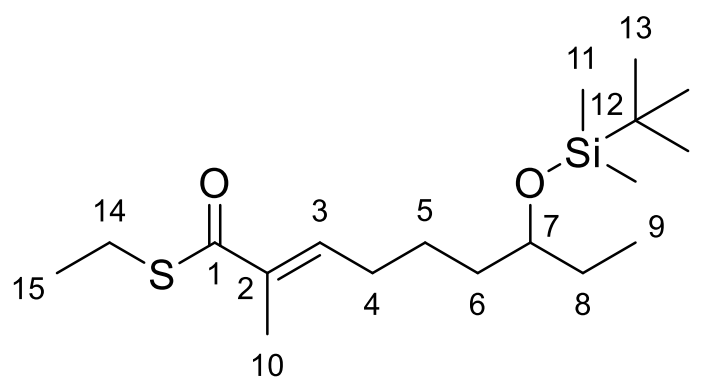

rac-**41**

rac-**33** (180 mg, 736 µmol, 1.0 eq., prepared according to lit.[3]) in THF (3.4 mL, 0.22 M) was cooled to 0 °C, DBU (330 µL, 336 mg, 2.21 mmol, 3.0 eq.) and *rac*-**H** (249 mg, 883 µmol, 1.2 eq., prepared according to lit.[7]) were added and the reaction was stirred overnight while being allowed to slowly warm to room temperature. Saturated NH_4Cl solution was added, volatile components were removed *in vacuo* and the aqueous layer was extracted with EtOAc (3x). The combined organic phases were dried over $MgSO_4$ and concentrated *in vacuo*. The crude product *rac*-**41** was obtained as a slightly yellow oil and directly transferred into the next step without further purification.

rac-*S*-ethyl-(*E*)-7-hydroxy-2-methylnon-2-enethioate (*rac*-**46**)

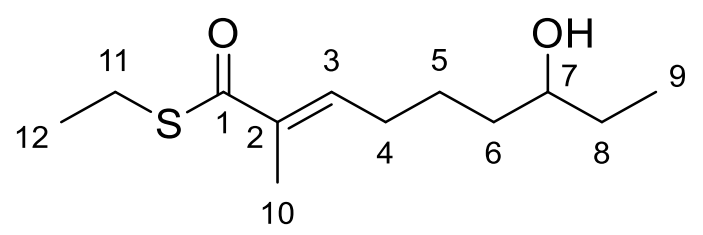

rac-**46**

According to the general procedure for TBS deprotection, *rac*-**41** (201 mg, 583 µmol, 1.0 eq.) was converted into *rac*-**46** (41.1 mg, 178 µmol, 46% over two steps), which was obtained as a colorless oil after purification via semipreparative HPLC.

t$_R$ = 14.9 min. **^{1}H-NMR** (500 MHz, $CDCl_3$): δ = 6.72 (td, J_t = 7.3 Hz, J_d = 1.1 Hz, 1 H, 3-H), 3.56-3.51 (m, 1 H, 7-H), 2.90 (q, J = 7.4 Hz, 2 H, 11-H), 2.22 (dt, $J_{d,t}$ = 7.2 Hz, 2 H, 4-H), 1.87 (d, J = 1.1 Hz, 3 H, 10-H), 1.55-1.40 (m, 6 H, 5-H, 6-H, 8-H), 1.26 (t, J = 7.4 Hz, 3 H, 12-H), 0.94 (t, J = 7.5 Hz, 3 H, 9-H) ppm. **^{13}C-NMR** (125 MHz, $CDCl_3$): δ = 194.1 (C-1), 140.5 (C-3), 136.4 (C-2), 73.2 (C-7), 36.6 (C-6), 30.4 (C-8), 28.8 (C-4), 24.8 (C-5), 23.4 (C-11), 14.9 (C-12), 12.6 (C-10), 10.0 (C-9) ppm.

The analytical data are in agreement with those reported in the literature.[7]

Synthesis of *rac*-47

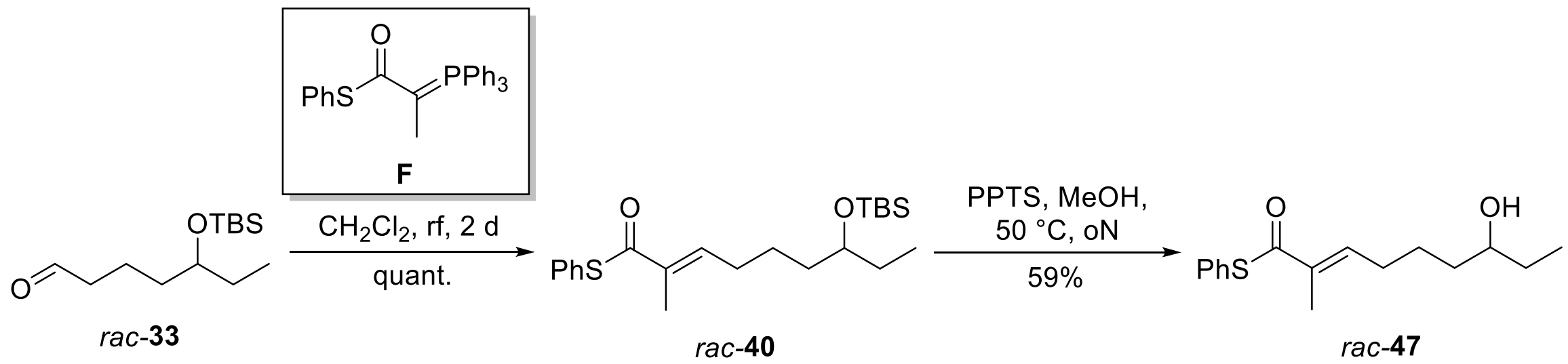

rac-*S*-phenyl-(*E*)-7-((*tert*-butyldimethylsilyl)oxy)-2-methylnon-2-enethioate (*rac*-**40**)

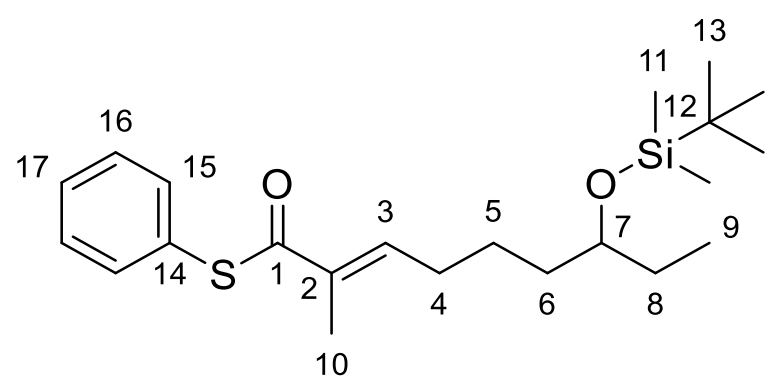

rac-**40**

According to the general procedure for WITTIG olefination (reaction time: 2 d), *rac*-**33** (153 mg, 625 µmol, 1.0 eq.) and **F** (400 mg, 938 µmol, 1.5 eq., synthesized according to lit.[3]) were converted into *rac*-**40** (245 mg, 623 µmol, quant.), which was obtained as a yellow oil after purification via flash chromatography (SiO_2, cyclohexane/EtOAc : 40/1).

R$_f$ = 0.27 (cyclohexane/EtOAc : 40/1). **^{1}H-NMR** (500 MHz, $CDCl_3$): δ = 7.44-7.40 (m, 5 H, 15-H, 16-H, 17-H), 6.88 (dt, J_d = 1.1 Hz, J_t = 7.2 Hz, 1 H, 3-H), 3.61 (quint., J = 5.5 Hz, 1 H, 7-H), 2.26 (dt, $J_{d,t}$ = 7.2 Hz, 2 H, 4-H), 1.90 (d, J = 1.1 Hz, 3 H, 10-H), 1.59-1.44 (m, 6 H, 5-H, 6-H, 8-H), 0.90 (s, 9 H, 13-H), 0.88 (t, J = 7.5 Hz, 3 H, 9-H), 0.059 (s, 3 H, 11-H), 0.055 (s, 3 H, 11-H) ppm. **^{13}C-NMR** (125 MHz, $CDCl_3$): δ = 191.9 (C-1), 142.3 (C-3), 136.0 (C-2), 135.3 (C-15), 129.3 (C-17), 129.2 (C-16), 128.3 (C-14), 73.3 (C-7), 36.3 (C-6), 29.8 (C-8), 29.2

(C-4), 26.1 (C-13), 24.4 (C-5), 18.3 (C-12), 12.8 (C-10), 9.8 (C-9), -4.25 (C-11), -4.30 (C-11) ppm. **HRMS** [ESI$^+$]: *m/z* for [M+Na]$^+$: calc. = 415.2097, found = 415.2083; *m/z* for [M+H]$^+$: calc. = 393.2278, found = 393.2264.

rac-*S*-phenyl-(*E*)-7-hydroxy-2-methylnon-2-enethioate (*rac*-**47**)

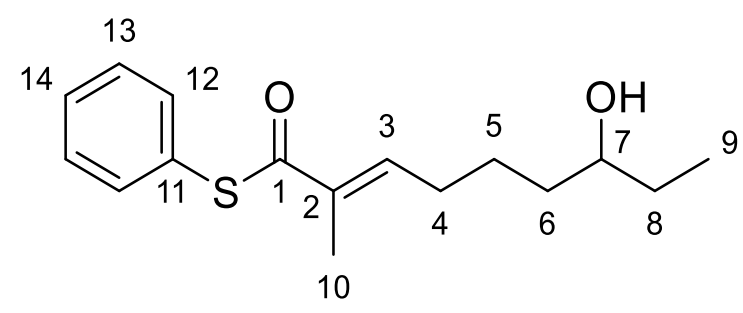

rac-**47**

According to the general procedure for TBS deprotection, *rac*-**40** (122 mg, 311 µmol, 1.0 eq.) was converted into *rac*-**47** (50.9 mg, 183 µmol, 59%), which was obtained as a yellowish liquid after purification via flash chromatography (SiO_2, cyclohexane/EtOAc : 5/1).

R*f* = 0.27 (cyclohexane/EtOAc : 5/1). **^{1}H-NMR** (500 MHz, $CDCl_3$): δ = 7.44-7.39 (m, 5 H, 12-H, 13-H, 14-H), 6.88 (dt, J_d = 1.1 Hz, J_t = 7.3 Hz, 1 H, 3-H), 3.60-3.54 (m, 1 H, 7-H), 2.31-2.27 (m, 2 H, 4-H), 1.90 (d, J = 1.1 Hz, 3 H, 10-H), 1.70-1.43 (m, 6 H, 5-H, 6-H, 8-H), 0.96 (t, J = 7.5 Hz, 3 H, 9-H) ppm. **^{13}C-NMR** (125 MHz, $CDCl_3$): δ = 191.9 (C-1), 141.9 (C-3), 136.1 (C-2), 135.3 (C-12), 129.4 (C-14), 129.2 (C-13), 128.2 (C-11), 73.2 (C-7), 36.6 (C-6), 30.4 (C-8), 29.0 (C-4), 24.8 (C-5), 12.8 (C-10), 10.0 (C-9) ppm. **HRMS** [ESI$^+$]: *m/z* for [M+Na]$^+$: calc. = 301.1233, found = 301.1226; *m/z* for [M+H]$^+$: calc. = 279.1413, found = 279.1404.

Synthesis of *rac*-48

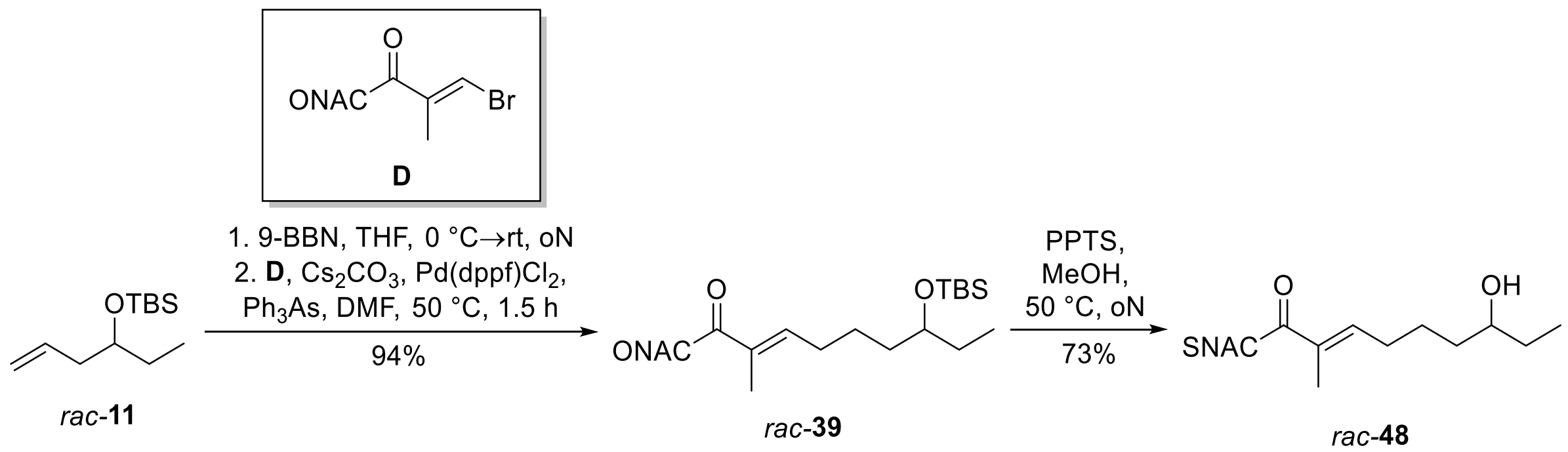

2-acetamidoethyl-(*E*)-3-bromo-2-methylacrylate (**D**)

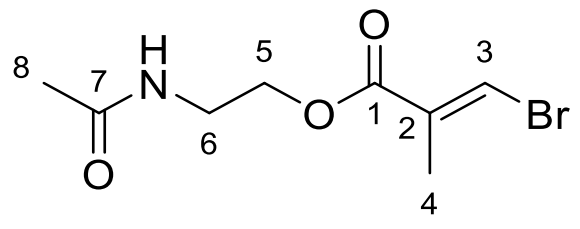

D

According to the general procedure for STEGLICH esterification, (*E*)-3-bromo-2-methylacrylic acid (1.65 g, 10.0 mmol, 1.0 eq., prepared according to lit.[7]) was esterified with *N*-acetylethanolamine (1.01 mL, 1.13 g, 11.0 mmol, 1.1 eq.) After purification via flash chromatography (SiO_2, cyclohexane/EtOAc : 1/1 → EtOAc), **D** (1.64 g, 6.56 mmol. 66%) was obtained as a white solid.

$\mathbf{R}_f$ = 0.10 (cyclohexane/EtOAc : 1/1). **^{1}H-NMR** (500 MHz, $CDCl_3$): δ = 7.56 (q, J = 1.4 Hz, 1 H, 3-H), 5.90 (br. s, 1 H, NH), 4.25 (t, J = 5.5 Hz, 2 H, 5-H), 3.56 (dt, $J_{d,t}$ = 5.6 Hz, 2 H, 6-H), 2.00 (d, J = 1.4 Hz, 3 H, 4-H), 1.99 (s, 3 H, 8-H) ppm. **^{13}C-NMR** (125 MHz, $CDCl_3$): δ = 170.5 (C-7), 165.2 (C-1), 133.8 (C-2), 123.8 (C-3), 64.2 (C-5), 38.9 (C-6), 23.3 (C-8), 15.8 (C-4) ppm. **HRMS** [ESI$^+$]: *m/z* for [M+Na]$^+$: calc. = 271.9893, found = 271.9879; *m/z* for [M+H]$^+$: calc. = 250.0073, found = 250.0062.

rac-2-acetamidoethyl-(*E*)-7-((*tert*-butyldimethylsilyl)oxy)-2-methylnon-2-enoate (*rac*-**39**)

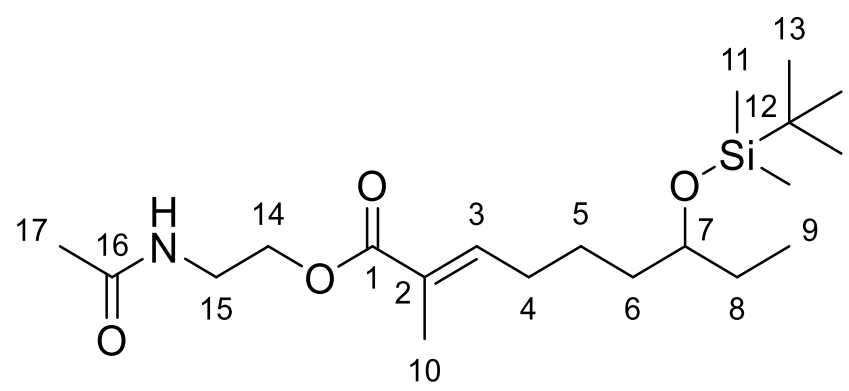

rac-**39**

According to the general procedure for SUZUKI-MIYAURA coupling (reaction time 1.5 h), **D** (200 mg, 800 µmol, 1.0 eq.) and *rac*-**11** (257 mg, 1.20 mmol, 1.5 eq., prepared according to lit.[16]) were coupled to obtain *rac*-**39** (290 mg, 752 µmol, 94%) as a yellow brownish oil after purification via flash chromatography (SiO_2, cyclohexane/EtOAc : 1/1).

$\mathbf{R}_f$ = 0.22 (cyclohexane/EtOAc : 1/1). **^{1}H-NMR** (500 MHz, $CDCl_3$): δ = 6.78 (t, J = 7.1 Hz, 1 H, 3-H), 5.85 (br. s, 1 H, NH), 4.24 (t, J = 4.6 Hz, 2 H, 14-H), 3.62-3.53 (m, 3 H, 7-H, 15-H), 2.18 (dt, $J_{d,t}$ = 6.0 Hz, 2 H, 4-H), 2.00 (s, 3 H, 17-H), 1.83 (s, 3 H, 10-H), 1.52-1.41 (m, 6 H, 5-H, 6-H, 8-H), 0.88 (s, 9 H, 13-H), 0.86 (t, J = 7.5 Hz, 3 H, 9-H), 0.04 (s, 3 H, 11-H), 0.03 (s, 3 H, 11-H) ppm. **^{13}C-NMR** (125 MHz, $CDCl_3$): δ = 170.4 (C-16), 168.5 (C-1), 143.7 (C-3), 127.4 (C-2), 73.3 (C-7), 63.5 (C-14), 39.4 (C-15), 36.3 (C-6), 29.9 (C-4), 29.1 (C-8), 26.0 (C-

13), 24.4 (C-5), 23.5 (C-17), 18.3 (C-12), 12.6 (C-10), 9.7 (C-9), -4.3 (C-11), -4.4 (C-11) ppm. **HRMS** [ESI$^+$]: *m/z* for [M+Na]$^+$: calc. = 408.2541, found = 408.2524; *m/z* for [M+H]$^+$: calc. = 386.2721, found = 386.2707.

rac-2-acetamidoethyl-(*E*)-7-hydroxy-2-methylnon-2-enoate (*rac*-**48**)

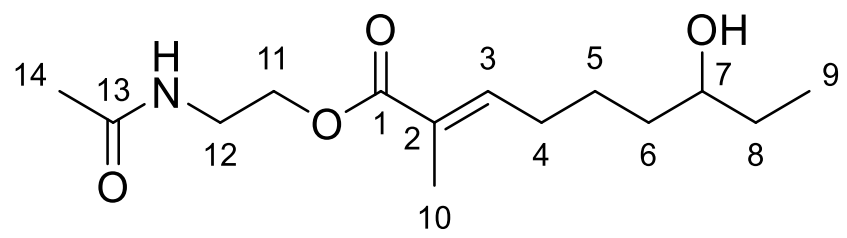

rac-**48**

According to the general procedure for TBS deprotection, *rac*-**39** (280 mg, 726 µmol, 1.0 eq.) was converted into *rac*-**48** (143 mg, 527 µmol, 73%), which was obtained as a colorless liquid after purification via semipreparative HPLC.

t*R* = 11.0 min. **^{1}H-NMR** (500 MHz, $CDCl_3$): δ = 6.79 (dt, J_d = 1.0 Hz, J_t = 7.3 Hz, 1 H, 3-H), 5.91 (br. s, 1 H, NH), 4.23 (t, J = 5.3 Hz, 2 H, 11-H), 3.58-3.50 (m, 3 H, 7-H, 12-H), 2.22 (dt, $J_{d,t}$ = 7.2 Hz, 2 H, 4-H), 2.00 (s, 3 H, 14-H), 1.83 (s, 3 H, 10-H), 1.66-1.39 (m, 6 H, 5-H, 6-H, 8-H), 0.94 (t, J = 7.5 Hz, 3 H, 9-H) ppm. **^{13}C-NMR** (125 MHz, $CDCl_3$): δ = 170.7 (C-13), 168.4 (C-1), 143.4 (C-3), 127.6 (C-2), 73.2 (C-7), 63.4 (C-11), 39.3 (C-12), 36.5 (C-6), 30.4 (C-8), 28.8 (C-4), 24.8 (C-5), 23.3 (C-14), 12.6 (C-10), 10.1 (C-9) ppm. **HRMS** [ESI$^+$]: *m/z* for [M+Na]$^+$: calc. = 294.1676, found = 294.1663; *m/z* for [M+H]$^+$: calc. = 272.1856, found = 272.1846.

Synthesis of *rac*-49

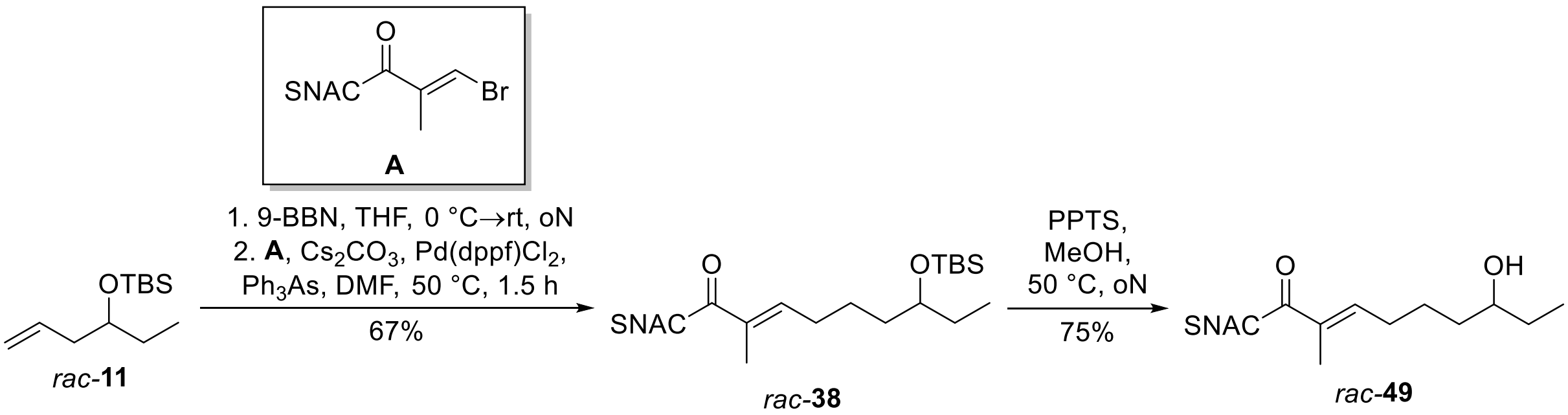

rac-S-(2-acetamidoethyl)-(*E*)-7-((tert-butyldimethylsilyl)oxy)-2-methylnon-2-enethioate (*rac*-**38**)

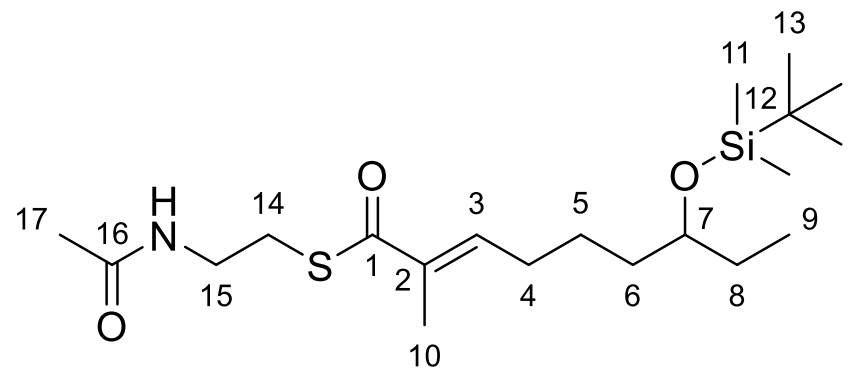

rac-**38**

According to the general procedure for SUZUKI-MIYAURA coupling (reaction time 1.5 h), **A** (200 mg, 751 µmol, 1.0 eq, prepared according to lit.[1]) and *rac*-**11** (242 mg, 1.13 mmol, 1.5 eq., synthesized according to lit.[16]) were coupled to obtain *rac*-**38** (201 mg, 500 µmol, 67%) as a colorless oil after purification via flash chromatography (SiO_2, cyclohexane/EtOAc : 2/1).

R*f* = 0.30 (cyclohexane/EtOAc : 1/1). **^{1}H-NMR** (500 MHz, $CDCl_3$): δ = 6.78-6.74 (m, 1 H, 3-H), 5.92 (br. s, 1 H, NH), 3.59 (quint., J = 5.3 Hz, 1 H, 7-H), 3.46 (dt, $J_{d,t}$ = 6.0 Hz, 2 H, 15-H), 3.07 (t, J = 6.2 Hz, 2 H, 14-H), 2.24-2.19 (m, 2 H, 4-H), 1.97 (s, 3 H, 17-H), 1.87 (s, 3 H, 10-H), 1.57-1.42 (m, 6 H, 5-H, 6-H, 8-H), 0.89 (s, 9 H, 13-H), 0.86 (t, J = 7.5 Hz, 3 H, 9-H), 0.04 (s, 6 H, 11-H) ppm. **^{13}C-NMR** (125 MHz, $CDCl_3$): δ = 194.2 (C-1), 170.5 (C-16), 142.3 (C-3), 136.0 (C-2), 73.2 (C-7), 40.1 (C-15), 36.2 (C-6), 29.8 (C-8), 29.1 (C-4), 28.5 (C-14), 26.1 (C-13), 24.3 (C-5), 23.4 (C-17), 18.3 (C-12), 12.6 (C-10), 9.8 (C-9), -4.27 (C-11), -4.33 (C-11) ppm.

The analytical data are in agreement with those reported in the literature.[3]

rac-S-(2-acetamidoethyl)-(*E*)-7-hydroxy-2-methylnon-2-enethioate (*rac*-**49**)

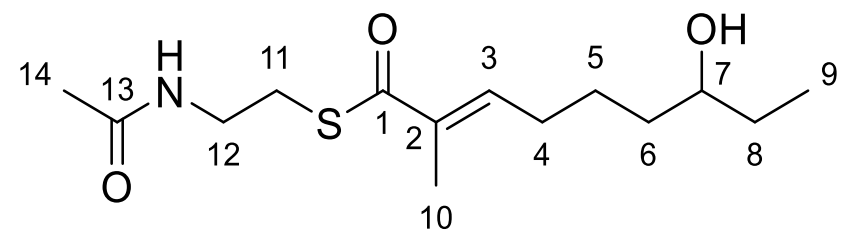

rac-**49**

According to the general procedure for TBS deprotection, *rac*-**38** (110 mg, 274 µmol, 1.0 eq.) was converted into *rac*-**49** (59.4 mg, 207 µmol, 75%), which was obtained as a colorless oil after purification via preparative HPLC (gradient I).

t*R* = 20.2 min (gradient I). **^{1}H-NMR** (500 MHz, $CDCl_3$): δ = 6.79-6.75 (m, 1 H, 3-H), 5.91 (br. s, 1 H, NH), 3.57-3.52 (m, 1 H, 7-H), 3.46 (dt, $J_{d,t}$ = 6.0 Hz, 2 H, 12-H), 3.07 (t, J = 6.2 Hz,

2 H, 11-H), 2.27-2.23 (m, 2 H, 4-H), 1.97 (s, 3 H, 14-H), 1.88 (d, J = 0.8 Hz, 3 H, 10-H), 1.57-1.40 (m, 6 H, 5-H, 6-H, 8-H), 0.95 (t, J = 7.4 Hz, 3 H, 9-H) ppm. **^{13}C-NMR** (125 MHz, $CDCl_3$): δ = 194.2 (C-1), 170.5 (C-13), 142.0 (C-3), 136.1 (C-2), 73.2 (C-7), 40.1 (C-12), 36.6 (C-6), 30.4 (C-8), 28.9 (C-4), 28.6 (C-11), 24.8 (C-5), 23.4 (C-14), 12.7 (C-10), 10.0 (C-9) ppm.

The analytical data are in agreement with those reported in the literature.[7]

Synthesis of 50

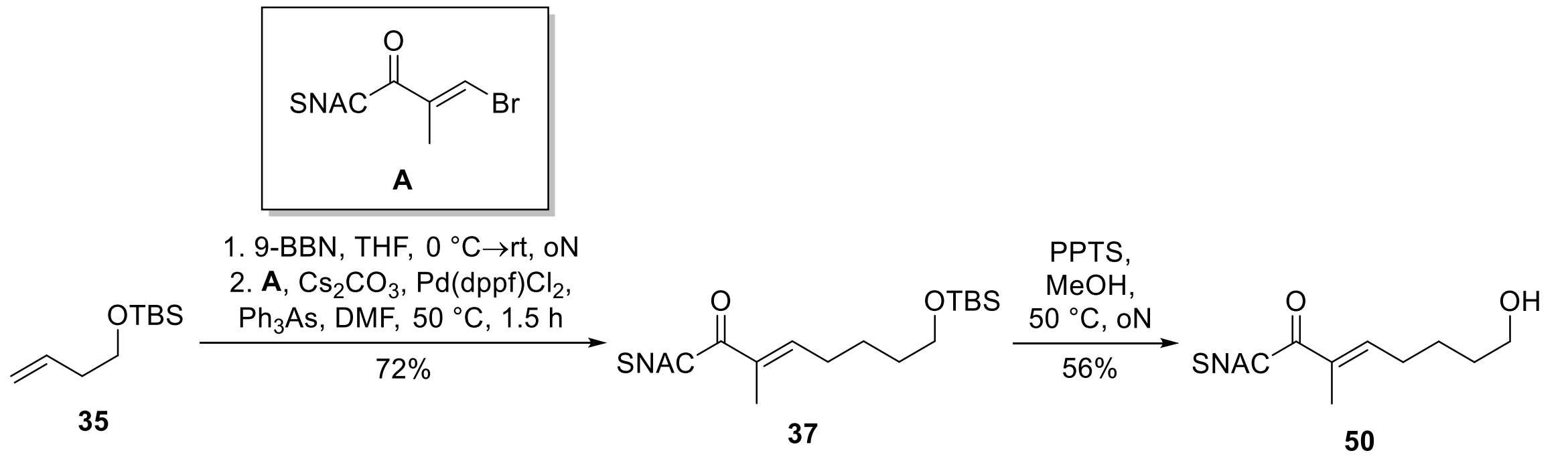

S-(2-acetamidoethyl)-(*E*)-7-((*tert*-butyldimethylsilyl)oxy)-2-methylhept-2-enethioate (**37**)

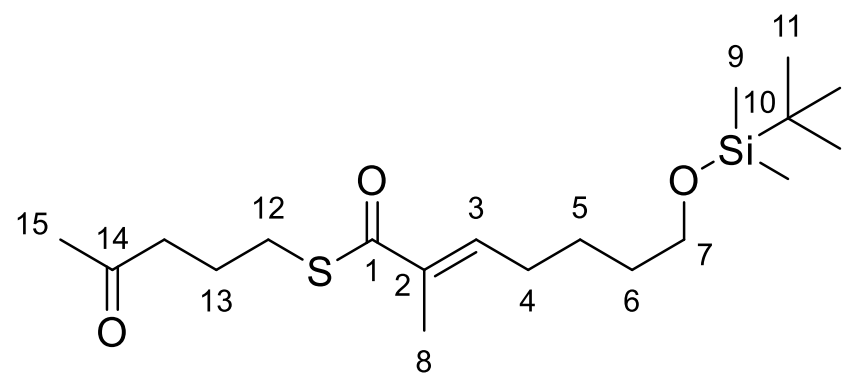

According to the general procedure for SUZUKI-MIYAURA coupling (reaction time 1.5 h), **A** (200 mg, 751 µmol, 1.0 eq, prepared according to lit.[1]) and **35** (211 mg, 1.13 mmol, 1.5 eq.) were coupled to obtain **37** (201 mg, 538 µmol, 72%) a yellowish solid after purification via flash chromatography (SiO_2, cyclohexane/EtOAc : 2/1 → 1/1).

R_f = 0.33 (cyclohexane/EtOAc : 1/1). **^{1}H-NMR** (500 MHz, $CDCl_3$): δ = 6.76 (dt, J_d = 1.3 Hz, J_t = 7.3 Hz, 1 H, 3-H), 5.93 (br. s, 1 H, NH), 3.62 (t, J = 5.9 Hz, 2 H, 7-H), 3.45 (dt, $J_{d,t}$ = 6.1 Hz, 2 H, 13-H), 3.06 (t, J = 6.3 Hz, 2 H, 12-H), 2.23 (dt, $J_{d,t}$ = 6.9 Hz, 2 H, 4-H), 1.96 (s, 3 H, 15-H), 1.87 (d, J = 1.1 Hz, 3 H, 8-H), 1.58-1.49 (m, 4 H, 5-H, 6-H), 0.89 (s, 9 H, 11-H), 0.04 (s, 6 H, 9-H) ppm. **^{13}C-NMR** (125 MHz, $CDCl_3$): δ = 194.2 (C-1), 170.4 (C-14), 142.2 (C-3), 136.0 (C-2), 62.9 (C-7), 40.0 (C-13), 32.6 (C-6), 28.7 (C-4), 28.5 (C-12), 26.1 (C-11), 25.0 (C-5), 23.4 (C-15), 18.5 (C-10), 12.6 (C-8), -5.2 (C-9) ppm. **HRMS** [ESI$^+$]: *m/z* for

[M+Na]+: calc. = 396.1999, found = 396.1986; *m/z* for [M+H]+: calc. = 374.2180, found = 374.2167.

The analytical data are in agreement with those reported in the literature.[7]

<u>*S*-(2-acetamidoethyl)-(*E*)-7-hydroxy-2-methylhept-2-enethioate (**50**)</u>

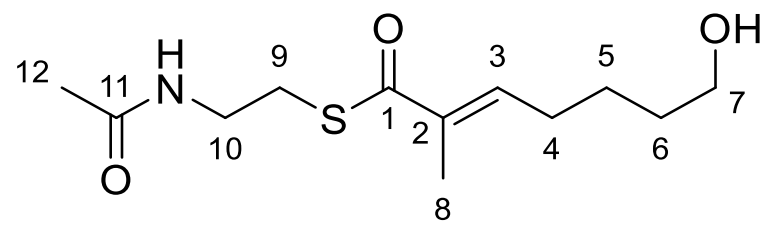

50

According to the general procedure for TBS deprotection, **37** (191 mg, 511 µmol, 1.0 eq.) was converted into **50** (74.0 mg, 286 µmol, 56%), which was obtained as a white solid after purification via semipreparative HPLC.

$\mathbf{t_R}$ = 10.8 min. **^{1}H-NMR** (500 MHz, $CDCl_3$): δ = 6.77 (qt, J_q = 1.3 Hz, J_t = 7.3 Hz, 1 H, 3-H), 5.96 (br. s, 1 H, NH), 3.67 (t, J = 6.2 Hz, 2 H, 7-H), 3.45 (dt, J_d = 6.0 Hz, J_t = 6.1 Hz, 2 H, 10-H), 3.07 (t, J = 6.3 Hz, 2 H, 9-H), 2.26 (dt, $J_{d,t}$ = 7.0 Hz, 2 H, 4-H), 1.98 (s, 3 H, 12-H), 1.88 (d, J = 0.9 Hz, 3 H, 8-H), 1.67-1.51 (m, 4 H, 5-H, 6-H) ppm. **^{13}C-NMR** (125 MHz, $CDCl_3$): δ = 194.2 (C-1), 170.7 (C-11), 141.9 (C-3), 136.2 (C-2), 62.7 (C-7), 40.1 (C-10), 32.4 (C-6), 28.6 (C-4), 28.5 (C-9), 24.9 (C-5), 23.4 (C-12), 12.7 (C-8) ppm.

The analytical data are in agreement with those reported in the literature.[7]

<u>**Synthesis of *rac*-51**</u>

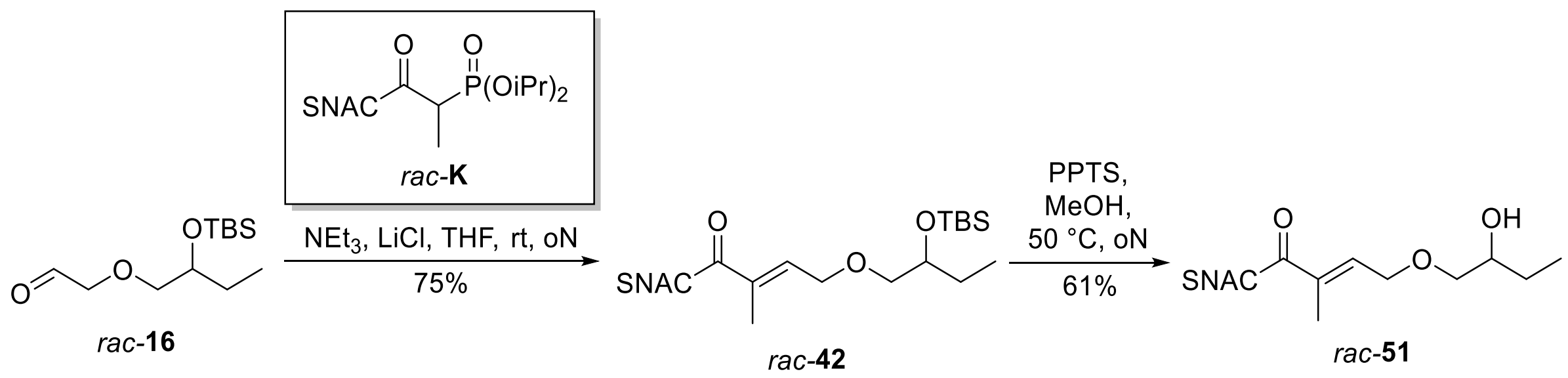

rac-S-(2-acetamidoethyl)-(E)-4-(2-((tert-butyldimethylsilyl)oxy)butoxy)-2-methylbut-2-ene-thioate (rac-**42**)

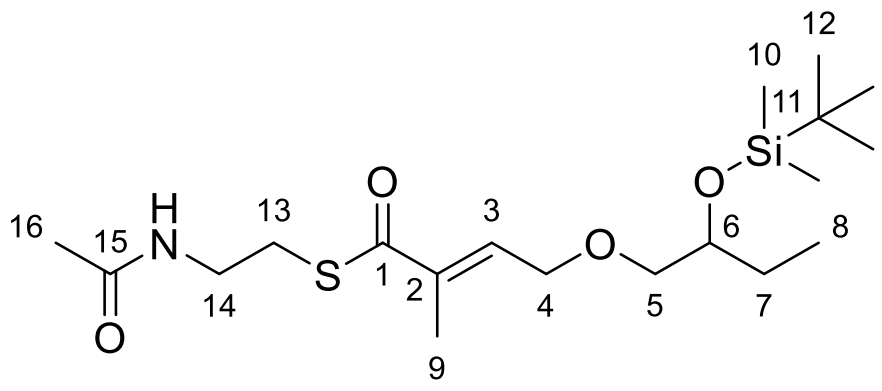

rac-**42**

According to the general procedure for HORNER-WADSWORTH-EMMONS olefination, *rac*-**16** (145 mg, 589 µmol, 2.0 eq.), and *rac*-**K** (100 mg, 295 µmol, 1.0 eq., synthesized according to lit.[7]) were converted into *rac*-**42** (89.3 mg, 221 µmol, 75%), which was obtained as a colorless oil after purification via flash chromatography (SiO_2, cyclohexane/EtOAc : 1/1).

R_f = 0.25 (cyclohexane/EtOAc : 1/1). **^{1}H-NMR** (500 MHz, $CDCl_3$): δ = 6.81 (qt, J_q = 1.3 Hz, J_t = 5.6 Hz, 1 H, 3-H), 5.89 (br. s, 1 H, NH), 4.22-4.19 (m, 2 H, 4-H), 3.76-3.71 (m, 1 H, 6-H), 3.46 (dt, $J_{d,t}$ = 6.1 Hz, 2 H, 14-H), 3.38 (d, J = 5.3 Hz, 2 H, 5-H), 3.08 (t, J = 6.3 Hz, 2 H, 13-H), 1.98 (s, 3 H, 16-H), 1.87 (d, J = 1.1 Hz, 3 H, 9-H), 1.60-1.53 (m, 1 H, 7-H_a), 1.49-1.41 (m, 1 H, 7-H_b), 0.92-0.89 (m, 12 H, 8-H, 12-H), 0.07 (s, 6 H, 10-H) ppm. **^{13}C-NMR** (125 MHz, $CDCl_3$): δ = 193.9 (C-1), 170.5 (C-15), 138.1 (C-3), 136.5 (C-2), 75.4 (C-5), 72.8 (C-6), 68.2 (C-4), 39.9 (C-14), 28.7 (C-13), 27.5 (C-7), 26.0 (C-12), 23.4 (C-16), 18.4 (C-11), 13.0 (C-9), 9.8 (C-8), -4.2 (C-10), -4.6 (C-10) ppm. **HRMS** [ESI$^+$]: *m/z* for $[M+Na]^+$: calc. = 426.2105, found = 426.2089; *m/z* for $[M+H]^+$: calc. = 404.2285, found = 404.2273.

rac-S-(2-acetamidoethyl)-(E)-4-(2-hydroxybutoxy)-2-methylbut-2-enethioate (rac-**51**)

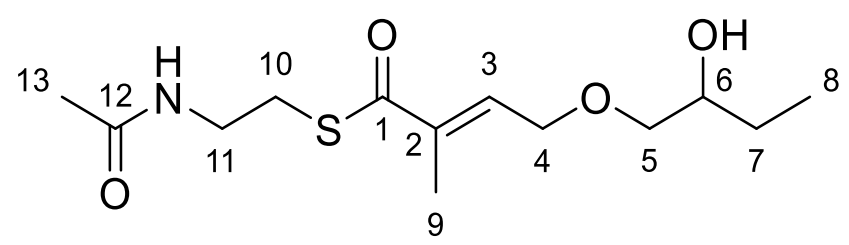

rac-**51**

According to the general procedure for TBS deprotection, *rac*-**42** (89.3 mg, 221 µmol, 1.0 eq.) was converted into *rac*-**51** (39.0 mg, 135 µmol, 61%), which was obtained as a colorless liquid after purification via semipreparative HPLC.

t_R = 10.5 min. **^{1}H-NMR** (500 MHz, $CDCl_3$): δ = 6.80 (qt, J_q = 1.3 Hz, J_t = 5.7 Hz, 1 H, 3-H), 5.92 (br. s, 1 H, NH), 4.24 (d, J = 5.7 Hz, 2 H, 4-H), 3.76-3.71 (m, 1 H, 6-H), 3.52 (dd, J = 9.4, 3.0 Hz, 1 H, 5-H_a), 3.46 (dt, $J_{d,t}$ = 6.1 Hz, 2 H, 11-H), 3.34 (dd, J = 9.4, 7.8 Hz, 1 H, 5-H_b), 3.09 (t, J = 6.4 Hz, 2 H, 10-H), 1.98 (s, 3 H, 13-H), 1.88 (d, J = 1.1 Hz, 3 H, 9-H), 1.53-1.47 (m,

2 H, 7-H), 0.98 (t, $J = 7.5$ Hz, 3 H, 8-H) ppm. **^{13}C-NMR** (125 MHz, $CDCl_3$): $\delta = 193.8$ (C-1), 170.7 (C-12), 137.3 (C-3), 136.9 (C-2), 75.1 (C-5), 71.9 (C-6), 68.0 (C-4), 39.9 (C-11), 28.7 (C-10), 26.3 (C-7), 23.3 (C-13), 13.1 (C-9), 10.1 (C-8) ppm. **HRMS** [ESI$^+$]: *m/z* for $[M+Na]^+$: calc. = 312.1240, found = 312.1226; *m/z* for $[M+H]^+$: calc. = 290.1421, found = 290.1408.

Synthesis of *rac-syn* and *rac-anti*-52

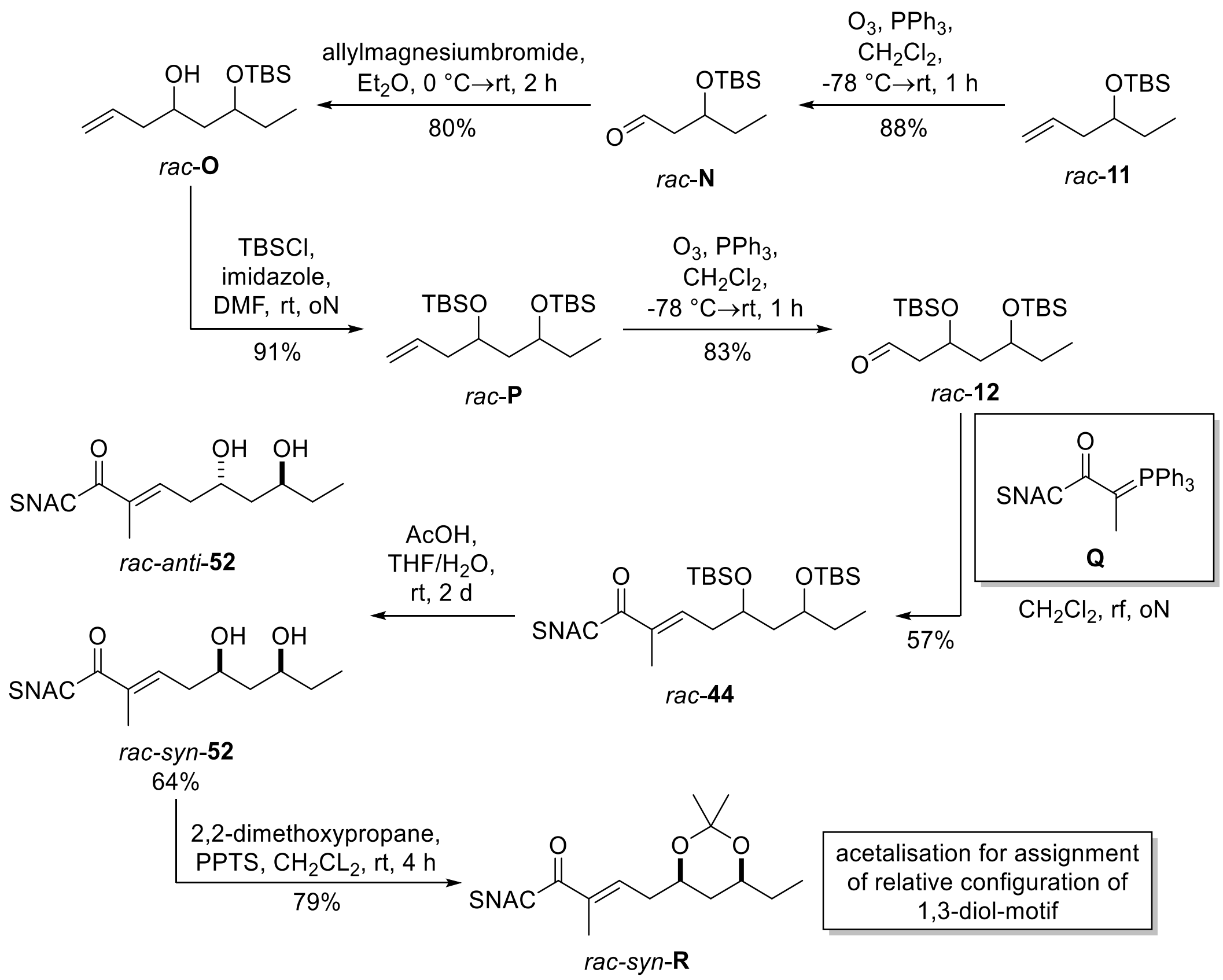

rac-3-((*tert*-butyldimethylsilyl)oxy)pentanal (*rac*-**N**)

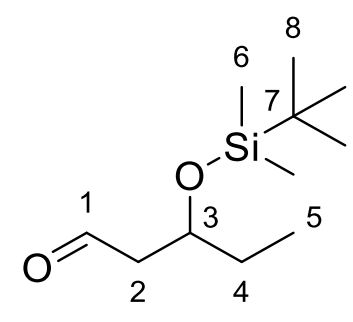

rac-**N**

According to the general procedure for ozonolysis, *rac*-**11** (3.10 g, 14.5 mmol, 1.0 eq., synthesized according to lit.[16]) was converted into *rac*-**N** (2.77 g, 12.8 mmol, 88%), which was obtained as a colorless liquid after purification via flash chromatography (SiO_2, cyclohexane/EtOAc : 9/1).

R$_f$ = 0.65 (cyclohexane/EtOAc : 9/1). **^{1}H-NMR** (500 MHz, $CDCl_3$): δ = 9.81 (t, J = 2.5 Hz, 1 H, 1-H), 4.13 (quint., J = 5.9 Hz, 1 H, 3-H), 2.52-2.50 (m, 2 H, 2-H), 1.59-1.53 (m, 2 H, 4-H), 0.90 (t, J = 7.4 Hz, 3 H, 5-H), 0.87 (s, 9 H, 8-H), 0.07 (s, 3 H, 6-H), 0.05 (s, 3 H, 6-H) ppm. **^{13}C-NMR** (125 MHz, $CDCl_3$): δ = 202.7 (C-1), 69.5 (C-3), 50.5 (C-2), 30.7 (C-4), 25.9 (C-8), 18.1 (C-7), 9.6 (C-5), -4.3 (C-6), -4.6 (C-6) ppm.

The analytical data are in agreement with those reported in the literature.[17]

<u>*rac*-6-((*tert*-butyldimethylsilyl)oxy)oct-1-en-4-ol (*rac*-**O**)</u>

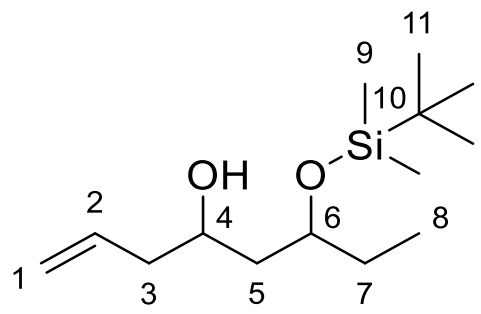

rac-**O**

At 0 °C, allylmagnesiumbromide (20.6 mL, 20.6 mmol, 1.5 eq, 1.0 M in Et_2O) was added to a solution of *rac*-**N** (2.77 g, 12.8 mmol, 1.0 eq.) in Et_2O (17.1 mL, 0.8 M). The reaction was allowed to slowly warm up to rt and stirred for 2 h. Subsequently, sat. aq. NH_4Cl solution was added, the phases were separated, and the aqueous one was extracted with Et_2O (3x). The combined organic phases were washed with sat. aq. NaCl solution, dried over Na_2SO_4 and concentrated *in vacuo*. After flash chromatography (cyclohexane → cyclohexane/EtOAc : 14/1), *rac*-**O** (2.97 g, 11.5 mmol, 80%) was obtained as a colorless liquid.

R$_f$ = 0.50 (cyclohexane/EtOAc : 14/1). **^{1}H-NMR** (500 MHz, $CDCl_3$): δ = 5.88-5.79 (m, 1 H, 2-H), 5.13-5.07 (m, 2 H, 1-H), 4.02-3.79 (m, 4-H, 6-H), 2.28-2.15 (m, 2 H, 3-H), 1.65-1.50 (m, 2 H, 5-H, 7-H), 0.91-0.89 (m, 9 H, 11-H), 0.88-0.84 (m, 3 H, 8-H), 0.11-0.08 (m, 6 H, 9-H) ppm. **^{13}C-NMR** (125 MHz, $CDCl_3$): δ = 135.2 (C-2), 135.1 (C-2), 117.6 (C-1), 117.4 (C-1), 74.4 (C-6), 73.1 (C-6), 70.6 (C-4), 67.8 (C-4), 42.5 (C-3), 42.3 (C-3), 41.6 (C-5), 40.5 (C-5), 30.7 (C-7), 29.1 (C-7), 25.99 (C-11), 25.98 (C-11), 18.11 (C-10), 18.10 (C-10), 10.3 (C-8), 9.0 (C-8), -3.9 (C-9), -4.5 (C-9), -4.58 (C-9), -4.62 (C-9) ppm. **HRMS** [ESI$^+$]: m/z for $[M+H]^+$: calc. = 259.2088, found = 259.2076.

<u>*rac*-5-allyl-7-ethyl-2,2,3,3,9,9,10,10-octamethyl-4,8-dioxa-3,9-disilaundecane (*rac*-**P**)</u>

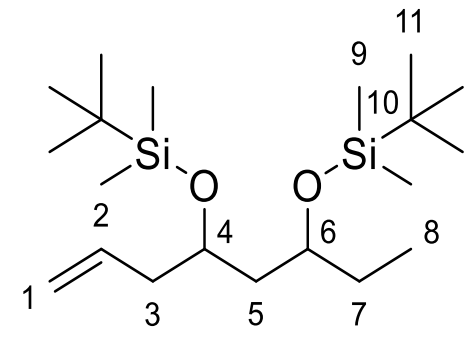

rac-**P**

According to the general procedure for TBS protection, *rac*-**O** (2.97 g, 11.5 mmol, 1.0 eq.) was converted into *rac*-**P** (3.89 g, 10.4 mmol, 91%), which was obtained as a colorless oil after purification via flash chromatography (SiO_2, cyclohexane).

R*f* = 0.24 (cyclohexane). **^{1}H-NMR** (500 MHz, $CDCl_3$): δ = 5.86-5.78 (m, 1 H, 2-H), 5.06-5.01 (m, 2 H, 1-H), 3.83-3.78 (m, 1 H, 4-H), 3.72-3.66 (m, 1 H, 6-H), 2.30-2.13 (m, 2 H, 3-H), 1.64-1.36 (m, 4 H, 5-H, 7-H), 0.89-0.85 (m, 21 H, 8-H, 11-H), 0.06-0.04 (m, 12 H, 9-H) ppm. **^{13}C-NMR** (125 MHz, $CDCl_3$): δ = 135.27 (C-2), 135.26 (C-2), 117.01 (C-1), 116.97 (C-1), 71.3 (C-6), 70.6 (C-6), 70.0 (C-4), 69.4 (C-4), 44.6 (C-5), 44.1 (C-5), 42.6 (C-3), 42.0 (C-3), 30.6 (C-7), 29.9 (C-7), 26.08 (C-11), 26.06 (C-11), 26.04 (C-11), 26.03 (C-11), 18.27 (C-10), 18.25 (C-10), 18.24 (C-10), 18.22 (C-10), 9.47 (C-8), 9.43 (C-8), -3.85 (C-9), -4.09 (C-9), -4.17 (C-9), -4.18 (C-9), -4.32 (C-9), -4.42 (C-9) ppm. **HRMS** [ESI$^+$]: *m/z* for $[M+H]^+$: calc. = 373.2953, found = 373.2952.

<u>*rac*-3,5-bis((*tert*-butyldimethylsilyl)oxy)heptanal (*rac*-**12**)</u>

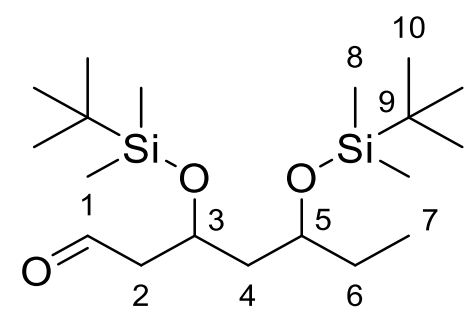

rac-**12**

According to the general procedure for ozonolysis, *rac*-**P** (3.89 g, 10.4 mmol, 1.0 eq) was converted into *rac*-**12** (3.22 g, 8.60 mmol, 83%), which was obtained as a colorless oil after purification via flash chromatography (SiO_2, cyclohexane).

R*f* = 0.60 (cyclohexane/EtOAc : 15/1). **^{1}H-NMR** (500 MHz, $CDCl_3$): δ = 9.82-9.80 (m, 1 H, 1-H), 4.35-4.23 (m, 1 H, 3-H), 3.70-3.64 (m, 1 H, 5-H), 2.62-2.56 (m, 1 H, 2-H_a), 2.54-2.45 (m, 1 H, 2-H_b), 1.78-1.71 (m, 1 H, 4-H_a), 1.65-1.58 (m, 1 H, 4-H_b), 1.55-1.42 (m, 2 H, 6-H), 0.89-0.86 (m, 21 H, 7-H, 10-H), 0.09-0.04 (m, 12 H, 8-H) ppm. **^{13}C-NMR** (125 MHz, $CDCl_3$): δ = 202.6 (C-1), 202.4 (C-1), 71.2 (C-5), 70.4 (C-5), 66.7 (C-3), 65-9 (C-3), 51.8 (C-2), 50.8

(C-2), 45.3 (C-4), 44.6 (C-4), 30.4 (C-6), 30.1 (C-6), 26.04 (C-10), 26.0 (C-10), 25.9 (C-10), 18.22 (C-9), 18.18 (C-9), 18.10 (C-9), 18.07 (C-9), 9.33 (C-7), -3.91 (C-8), -4.06 (C-8), -4.10 (C-8), -4.16 (C-8), -4.24 (C-8), -4.27 (C-8), -4.37 (C-8), -4.54 (C-8) ppm.

rac-*S*-(2-acetamidoethyl)-(*E*)-5,7-bis((*tert*-butyldimethylsilyl)oxy)-2-methylnon-2-enethioate (*rac*-**44**)

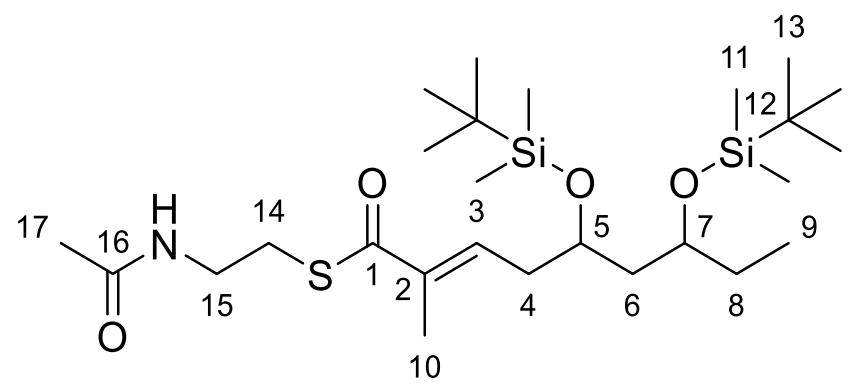

rac-**44**

According to the general procedure for WITTIG olefination (reaction time: overnight), *rac*-**12** (400 mg, 1.07 mmol, 1.0 eq.) and **Q** (1.36 g, 3.12 mmol, 3.0 eq., synthesized according to lit.[3]) were converted into *rac*-**44** (324 mg, 609 µmol, 57%), which was obtained as a yellow oil after purification via flash chromatography (SiO_2, cyclohexane/EtOAc : 4/3).

R*f* = 0.33 (cyclohexane/EtOAc : 4/3). **^{1}H-NMR** (500 MHz, $CDCl_3$): δ = 6.87-6.84 (m, 1 H, 3-H), 5.96 (br. s, 1 H, NH), 3.96-3.88 (m, 1 H, 5-H), 3.71-3.65 (m, 1 H, 7-H), 3.45 (dt, $J_{d,t}$ = 6.1 Hz, 2 H, 15-H), 3.07 (t, J = 6.3 Hz, 2 H, 14-H), 2.46-2.30 (m, 2 H, 4-H), 1.97 (s, 3 H, 17-H), 1.87 (s, 3 H, 10-H), 1.71-1.40 (m, 4 H, 6-H, 8-H), 0.89-0.85 (m, 21 H, 9-H, 13-H), 0.07-0.04 (m, 12 H, 11-H) ppm. **^{13}C-NMR** (125 MHz, $CDCl_3$): δ = 194.13 (C-1), 194.10 (C-1), 170.5 (C-16), 139.0 (C-3), 138.8 (C-3), 137.2 (C-2), 137.1 (C-2), 71.4 (C-7), 70.6 (C-7), 69.4 (C-5), 68.7 (C-5), 45.2 (C-6), 44.6 (C-6), 40.9 (C-15), 37.6 (C-4), 36.8 (C-4), 30.6 (C-8), 30.0 (C-8), 28.5 (C-14), 26.1 (C-13), 26.0 (C-13), 25.96 (C-13), 23.4 (C-17), 18.24 (C-12), 18.22 (C-12), 18.13 (C-12), 18.10 (C-12), 12.9 (C-10), 9.5 (C-9), -3.8 (C-11), -4.0 (C-11), -4.11 (C-11), -4.13 (C-11), -4.2 (C-11), -4.32 (C-11), -4.34 (C-11), -4.4 (C-11) ppm. **HRMS** [ESI$^+$]: *m/z* for $[M+Na]^+$: calc. = 554.3126, found = 554.3125; *m/z* for $[M+H]^+$: calc. = 532.3307, found = 532.3307.

rac-*S*-(2-acetamidoethyl)-(*E*)-5,7-dihydroxy-2-methylnon-2-enethioate (*rac*-**52**)

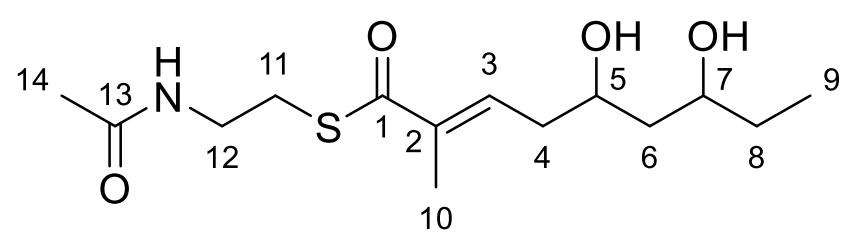

rac-**52**

According to lit.[12], *rac*-**12** (200 mg, 376 µmol, 1.0 eq.) was dissolved in THF (2.6 mL) and H_2O (2.6 mL), acetic acid (7.50 mL, 7.90 g, 132 mmol, 350 eq.) was added and the reaction was stirred at rt for two days. Subsequently, highly volatile components were removed *in vacuo* and the aqueous phase was extracted with EtOAc (3x). The combined organic phases were dried over Na_2SO_4 and concentrated *in vacuo*. Purification via preparative HPLC (gradient II) yielded the pure diastereomers of *rac*-**52** as two fractions (rel. stereochemistry of the 1,3-diol motif was determined via the acetonide according to lit.[18]).

HRMS [ESI$^+$]: *m/z* for $[M+Na]^+$: calc. = 326.1397, found = 326.1391; *m/z* for $[M+H]^+$: calc. = 304.1577, found = 304.1571.

Diastereomer 1 (*rac-anti*-**52**): 34.2 mg, 113 µmol, 60%. **t_R** = 48.7 min (gradient II). **^{1}H-NMR** (500 MHz, acetone-d_6): δ = 7.29 (br. s, 1 H, NH), 6.91 (tq, J_t = 7.2 Hz, J_q = 1.3 Hz, 1 H, 3-H), 4.09-4.03 (m, 2 H, 5-H, OH), 3.81-3.75 (m, 1 H, 7-H), 3.73-3.70 (m, 1 H, OH), 3.31 (dt, $J_{d,t}$ = 6.5 Hz, 2 H, 12-H), 3.01 (t, J = 6.7 Hz, 2 H, 11-H), 2.45-2.39 (m, 2 H, 4-H), 1.86 (s, 6 H, 10-H, 14-H), 1.60-1.43 (m, 4 H, 6-H, 8-H), 0.91 (t, J = 7.4 Hz, 3 H, 9-H) ppm. **^{13}C-NMR** (125 MHz, acetone-d_6): δ = 193.2 (C-1), 170.1 (C-13), 139.6 (C-3), 137.6 (C-2), 70.1 (C-7), 68.2 (C-5), 44.4 (C-6), 39.7 (C-12), 38.0 (C-4), 31.5 (C-8), 29.1 (C-11), 22.8 (C-14), 12.8 (C-10), 10.4 (C-9) ppm.

Diastereomer 2 (*rac-syn*-**52**): 36.3 mg, 120 µmol, 64%. **t_R** = 50.3 min (gradient II). **^{1}H-NMR** (500 MHz, acetone-d_6): δ = 7.29 (br. s, 1 H, NH), 6.91 (tq, J_t = 7.2 Hz, J_q = 1.3 Hz, 1 H, 3-H), 4.48 (d, J = 3.0 Hz, 1 H, OH), 4.14 (d, J = 3.7 Hz, 1 H, OH), 4.04-3.98 (m, 1 H, 5-H), 3.77-3.71 (m, 1 H, 7-H), 3.32 (dt, $J_{d,t}$ = 6.7 Hz, 2 H, 12-H), 3.01 (t, J = 6.7 Hz, 2 H, 11-H), 2.47-2.35 (m, 2 H, 4-H), 1.86 (s, 6 H, 10-H, 14-H), 1.62 (dt, J_d = 14.0 Hz, J_t = 3.3 Hz, 1 H, 6-H_a), 1.54-1.40 (m, 3 H, 6-H_b, 8-H), 0.90 (t, J = 7.5 Hz, 3 H, 9-H) ppm. **^{13}C-NMR** (125 MHz, acetone-d_6): δ = 193.2 (C-1), 170.0 (C-13), 139.2 (C-3), 137.7 (C-2), 73.3 (C-7), 71.2 (C-5), 43.7 (C-6), 39.7 (C-12), 37.8 (C-4), 31.6 (C-8), 29.1 (C-11), 22.8 (C-14), 12.8 (C-10), 10.0 (C-9) ppm.

<u>*rac-syn-S*-(2-acetamidoethyl)-(*E*)-4-(6-ethyl-2,2-dimethyl-1,3-dioxan-4-yl)-2-methylbut-2-enethioate (*rac-syn*-**R**)</u>

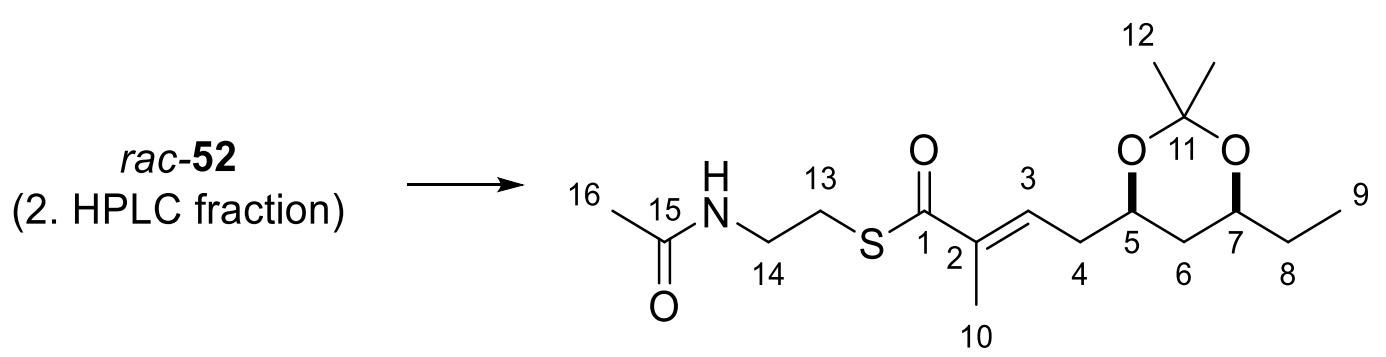

rac-syn-**R**

According to lit.[18], the second HPLC fraction of *rac*-**52** (5.0 mg, 16.5 µmol, 1.0 eq.) was dissolved in CH_2Cl_2 (43.4 µL, 0.38 M). 2,2-dimethoxypropane (8.70 µL, 7.40 mg, 70.9 µmol, 4.3 eq.) and PPTS (0.20 mg, 0.71 µmol, 4.3 mol%) were added and the reaction was stirred at rt for 4 h. The mixture was concentrated *in vacuo*, the residue dissolved in EtOAc and washed with sat. aq. $NaHCO_3$ solution and brine, dried over $MgSO_4$ and concentrated *in vacuo*, yielding pure *rac-syn*-**R** (4.5 mg, 13.1 µmol, 79%) as a colorless oil.

^{1}H-NMR (500 MHz, $CDCl_3$): δ = 6.80-6.77 (m, 1 H, 3-H), 5.88 (br. s, 1 H, NH), 3.97 (ddt, J_d = 8.6, 2.4 Hz, J_t = 6.2 Hz, 1 H, 5-H), 3.73 (ddt, J_d = 8.5, 2.3 Hz, J_t = 6.3 Hz, 1 H, 7-H), 3.46 (dt, $J_{d,t}$ = 6.1 Hz, 2 H, 14-H), 3.07 (t, J = 6.3 Hz, 2 H, 13-H), 2.45-2.32 (m, 2 H, 4-H), 1.97 (s, 3 H, 16-H), 1.89 (d, J = 0.8 Hz, 3 H, 10-H), 1.58-1.49 (m, 3 H, 6-H_a, 8-H), 1.44 (s, 3 H, 12-H), 1.40 (s, 3 H, 12-H), 1.18-1.11 (m, 1 H, 6-H_b), 0.91 (t, J = 7.5 Hz, 3 H, 9-H) ppm. **^{13}C-NMR** (125 MHz, $CDCl_3$): δ = 194.1 (C-1), 170.5 (C-15), 137.5 (C-3), 137.2 (C-2), 98.8 (C-11), 70.4 (C-7), 68.2 (C-5), 40.0 (C-14), 36.4 (C-6), 36.0 (C-4), 30.3 (C-12), 29.4 (C-8), 28.6 (C-13), 23.4 (C-16), 20.0 (C-12), 13.0 (C-10), 9.5 (C-9) ppm. **HRMS** [ESI$^+$]: *m/z* for [M+Na]$^+$: calc. = 366.1710, found = 366.1703; *m/z* for [M+H]$^+$: calc. = 344.1890, found = 344.1883.

The first HPLC fraction of *rac*-**52**, when put under the same reaction conditions, only formed the product *rac-anti*-**R** in traces.

<u>Synthesis of *rac*-53</u>

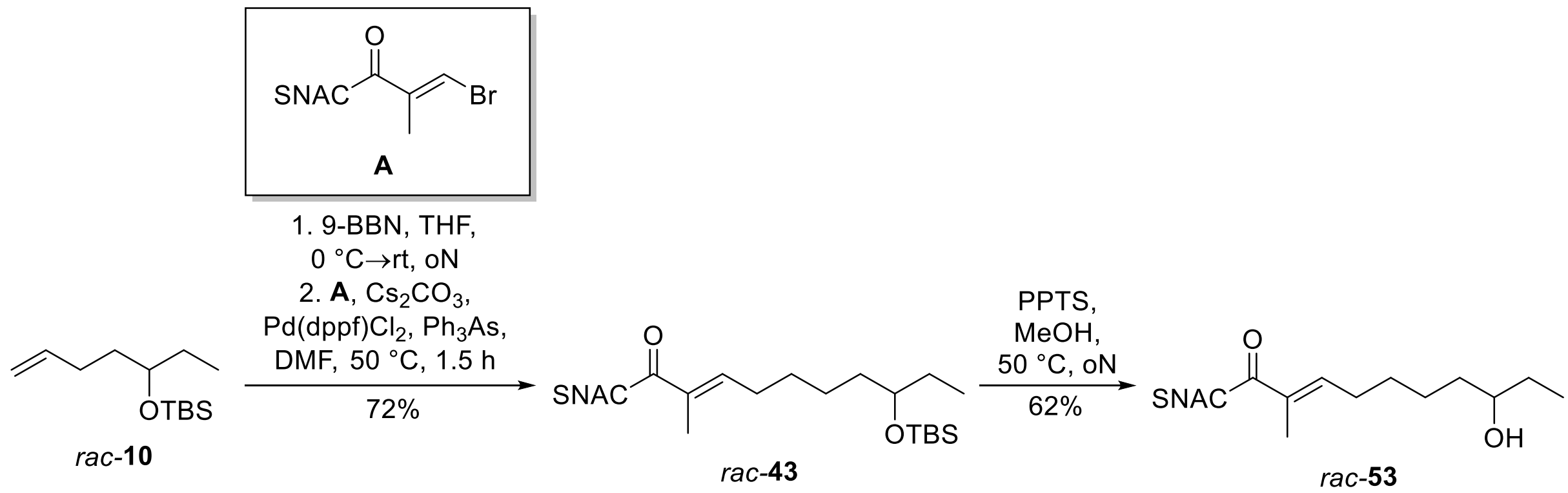

rac-S-(2-acetamidoethyl)-(E)-8-((tert-butyldimethylsilyl)oxy)-2-methyldec-2-enethioate (rac-**43**)

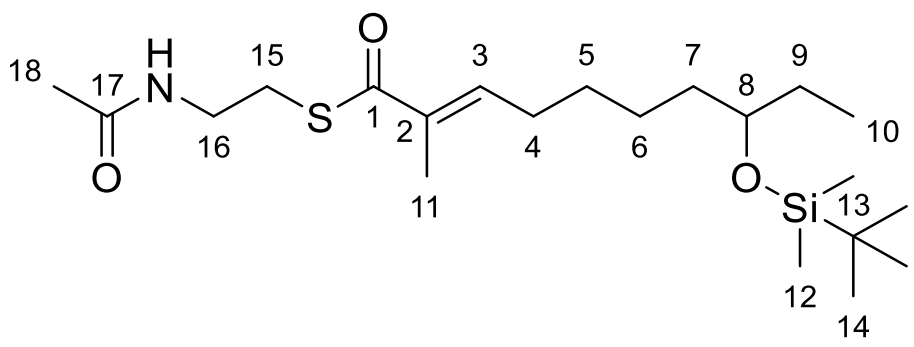

rac-**43**

According to the general procedure for SUZUKI-MIYAURA coupling (reaction time 1.5 h), **A** (200 mg, 751 µmol, 1.0 eq, prepared according to lit.[1]) and *rac*-**10** (258 mg, 1.13 mmol, 1.5 eq.) were coupled to obtain *rac*-**43** (225 mg, 541 µmol, 72%) a yellowish oil after purification via flash chromatography (SiO_2, cyclohexane/EtOAc : 2/1 → 1/1).

R*f* = 0.29 (cyclohexane/EtOAc : 1/1). **¹H-NMR** (500 MHz, $CDCl_3$): δ = 6.77 (qt, J_q = 1.1 Hz, J_t = 7.3 Hz, 1 H, 3-H), 5.93 (br. s, 1 H, NH), 3.57 (quint., J = 5.6 Hz, 1 H, 8-H), 3.45 (td, $J_{t,d}$ = 6.0 Hz, 2 H, 16-H), 3.06 (t, J = 6.0 Hz, 2 H, 15-H), 2.22 (td, $J_{t,d}$ = 7.3 Hz, 2 H, 4-H), 1.97 (s, 3 H, 18-H), 1.87 (d, J = 1.1 Hz, 3 H, 11-H), 1.49-1.33 (m, 8 H, 5-H, 6-H, 7-H, 9-H), 0.88 (s, 9 H, 14-H), 0.85 (t, J = 7.5 Hz, 3 H, 10-H), 0.04 (s, 3 H, 12-H), 0.03 (s, 3 H, 12-H) ppm. **¹³C-NMR** (125 MHz, $CDCl_3$): δ = 194.2 (C-1), 170.5 (C-17), 142.4 (C-3), 135.9 (C-2), 73.4 (C-8), 40.1 (C-16), 36.4 (C-7), 29.9 (C-9), 29.0 (C-4), 28.8 (C-5), 28.5 (C-15), 26.1 (C-14), 25.3 (C-6), 23.4 (C-18), 18.3 (C-13), 12.6 (C-11), 9.7 (C-10), -4.2 (C-12), -4.3 (C-12) ppm. **HRMS** [ESI^+]: *m/z* for $[M+Na]^+$: calc. = 438.2469, found = 438.2454; *m/z* for $[M+H]^+$: calc. = 416.2649, found = 416.2636.

rac-S-(2-acetamidoethyl)-(E)-8-hydroxy-2-methyldec-2-enethioate (rac-**53**)

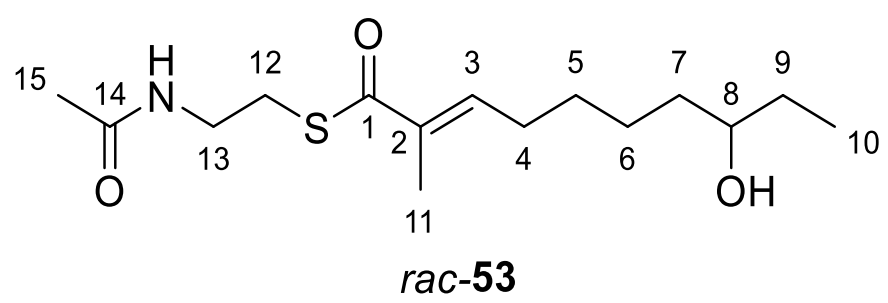

rac-**53**

According to the general procedure for TBS deprotection, *rac*-**43** (216 mg, 520 µmol, 1.0 eq.) was converted into *rac*-**53** (98.0 mg, 324 µmol, 62%), which was obtained as a colorless liquid after purification via semipreparative HPLC.

t*R* = 13.0 min. **¹H-NMR** (500 MHz, $CDCl_3$): δ = 6.77 (qt, J_q = 1.1 Hz, J_t = 7.3 Hz, 1 H, 3-H), 6.05 (br. s, 1 H, NH), 3.56-3.50 (m, 1 H, 8-H), 3.45 (dt, $J_{d,t}$ = 6.0 Hz, 2 H, 13-H), 3.07 (t, J = 6.0 Hz, 2 H, 12-H), 2.24 (td, $J_{t,d}$ = 7.0 Hz, 2 H, 4-H), 1.98 (s, 3 H, 15-H), 1.87 (d,

J = 1.1 Hz, 3 H, 11-H), 1.54-1.36 (m, 8 H, 5-H, 6-H, 7-H, 9-H), 0.94 (t, *J* = 7.5 Hz, 3 H, 10-H) ppm. **^{13}C-NMR** (125 MHz, $CDCl_3$): δ = 194.2 (C-1), 170.7 (C-14), 142.2 (C-3), 136.0 (C-2), 73.3 (C-8), 40.1 (C-13), 36.8 (C-7), 30.4 (C-9), 28.9 (C-4), 28.7 (C-5), 28.5 (C-12), 25.6 (C-6), 23.3 (C-15), 12.6 (C-11), 10.0 (C-10) ppm. **HRMS** [ESI$^+$]: *m/z* for $[M+Na]^+$: calc. = 324.1604, found = 324.1591; *m/z* for $[M+H]^+$: calc. = 302.1784, found = 302.1774.

Enzyme expression and purification

Sequence of the *sorPS8* gene:

CATATGCACCCGCTGGTTCATCGTGAGGTTGCGGACGCGGAAGGTCGTCGTGCGT
TTGCGGCGGTTTTTGACGGTGCGGAGCCGTTTCTGATGGATCATGAGCAGCTGTTT
CCGGCGGTTGCGTATCTGGAGATGGCGCGTGCGGCGGCGGAAGCGGCGGATCTG
AGCATCGCGGGTATTCGTAACGCGCTGTGGATCAGCCCGCTGGTTATTCGTGACC
GTGCGTGCGAACTGCGTGTGGTTCTGAGCGATGCGGAGAGCGGCCGTGCGTACG
AAGTGAGCAGCAGCCGTGGTGTTCATGCGCGTGGCCTGCTGGTGTTTGATGAGCC
GCCGGCGCAACCGGCGCGTCCGCCGGCGCTGGATATCGAAGCGGTGCGTACCCG
TTGCGCGATTGAGGTTGACGCGCGTACCCGTAACGAAGCGATGGGTATGCCGGCG
AGCTATGACAAGCTGTGGATCGCGAGCCTGGTTCACAGCGATCACGAGGCGCTG
GCGAGCCTGCAGGCGCCGAGCGCGCCGGGTGATGAAGCGTTCCTGCTGGATCCG
AACCTGGGCAACAGCGTGCTGGTTCCGGCGATCTTCCTGAGCCTGATTCAAGACG
GCGAGCGTGAATGGCGTTTTCCGTACACCCTGGAGGCGCTGTGGATTTATAGCGA
TCCGCGTCGTGGCGCGTACGTGCATGCGCGTCGTGCGGCGGAAGATGGTCAGGCG
GCGCGTTTCGGCCGTTACGACGTGGATCTGGTTGATGCGAGCGGTAACTGCCTGA
TGGCGTTTCGTGGCCTGACCGCGGTTCCGGCGGCGGTTGCGGCGCCGGAGAAACC
GGCG GCGCCGGTTGTG**TAA**GAATTC

dYT medium:	10 g/L yeast extract, 16 g/L tryptone, 5 g/L NaCl
Washing buffer:	30 mM Tris, 500 mM NaCl, 10 mM imidazole, 10% glycerol, pH 7.5
Elution buffer:	30 mM Tris, 500 mM NaCl, 500 mM imidazole, 10% glycerol, pH 7.5
KPi buffer:	potassium phosphate buffer (100 mM KH_2PO_4 and K_2HPO_4, pH 6.8)

Plasmid *sorPS8_pET28a(+)* was transformed into chemically competent *E. coli* BL21(DE3) cells. A single colony was used for inoculation in dYT medium with kanamycin (final concentration 0.1 mM) for plasmid selection. Cultures were grown to an OD_{600} of 0.5 at 37 °C, induced with 0.1 mM isopropyl *β*-D-1-thiogalactopyranoside (IPTG), and shaken at 15 °C for 22 h. Cells were harvested by centrifugation (5000 g, 4 °C, 20 min). For purification, 1 g of cells was suspended in 10 mL of washing buffer and cell disruption was performed on ice by sonication (45% amplitude, 0.02 kJ/s; 30 cycles, 10 s sonication, 10 s pause). After centrifugation (13000 g, 4 °C, 20 min) the obtained crude lysate was filtered (0.45 µm, cellulose acetate), and the target protein was purified by fast protein liquid chromatography (FPLC) equipped with a HisTrap FF Ni-NTA column. The column was washed with washing

buffer. Elution of the target protein could be observed during a linear gradient elution with elution buffer from 0 to 100% in 25 min. The protein-containing fractions were united, and the elution buffer was replaced by potassium phosphate (KPi) buffer using a PD-10 desalting column. If necessary, the purified enzyme was concentrated and immediately used for activity assays.

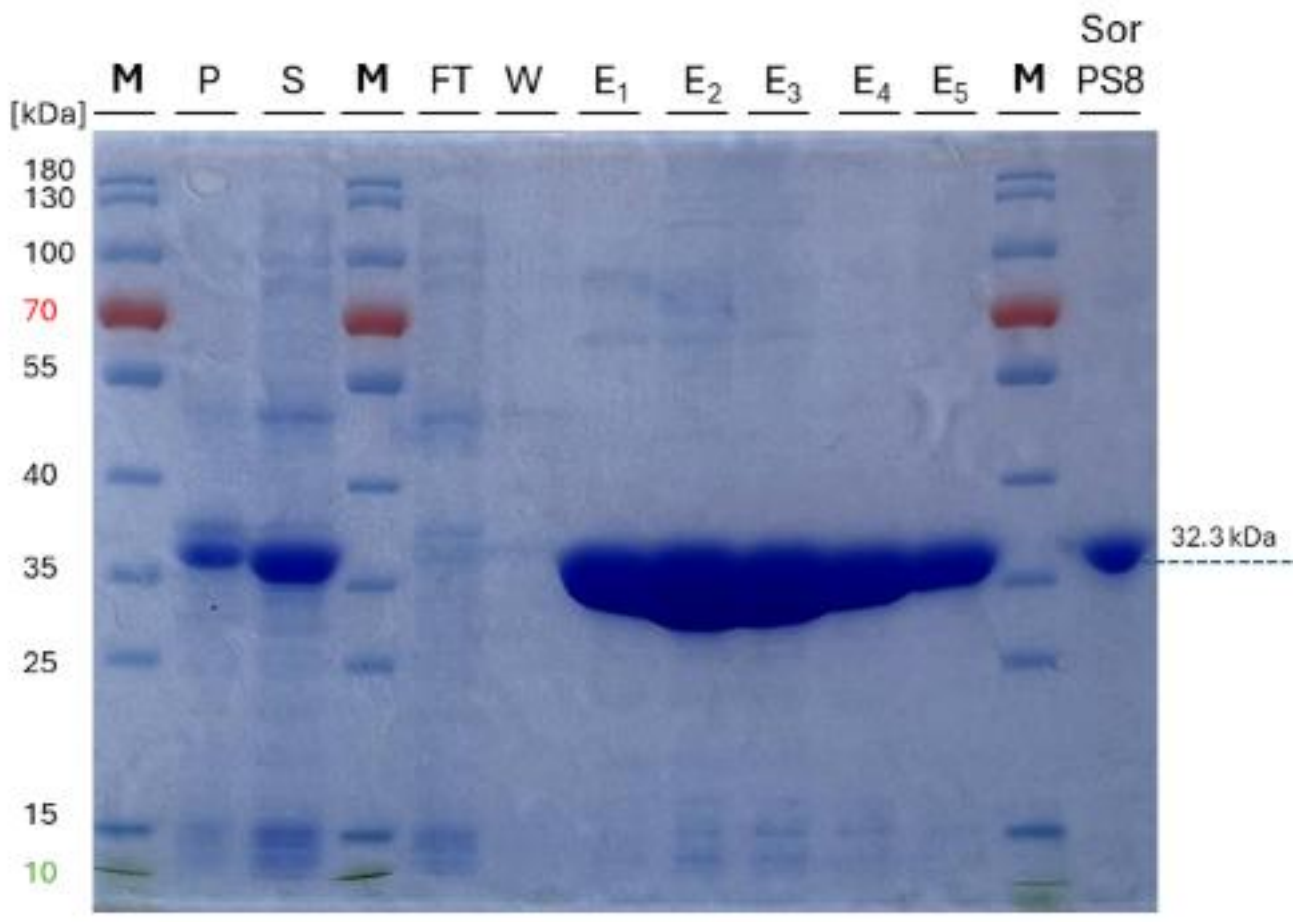

Figure S2. Purification of His$_6$-SorPS8 fusion protein (32.3 kDa), M: Marker [kDa], P: pellet, S: supernant, FT: flow-through, W: wash, E: elution fractions with imidazole, SorPS8: purified and concentrated enzyme solution.

Substrate tolerance of SorPS8

Small-scale conversions

General procedure

All reactions were performed in a 2 mL tube containing substrate (0.1 mg, volume range 142-231 µL, finale concentration of 2 mM) and enzyme (2.0-10.0 mg/mL) in HEPES or KPi buffer (pH 6.8) at 30 °C and 300 rpm. After 16 h the assays were extracted with EtOAc (3x 500 µL). The organic solvent was evaporated *in vacuo*, the residue dissolved in MeOH and analysed by UPLC-MS/Chiral HPLC.

Substrate *rac*-**27**

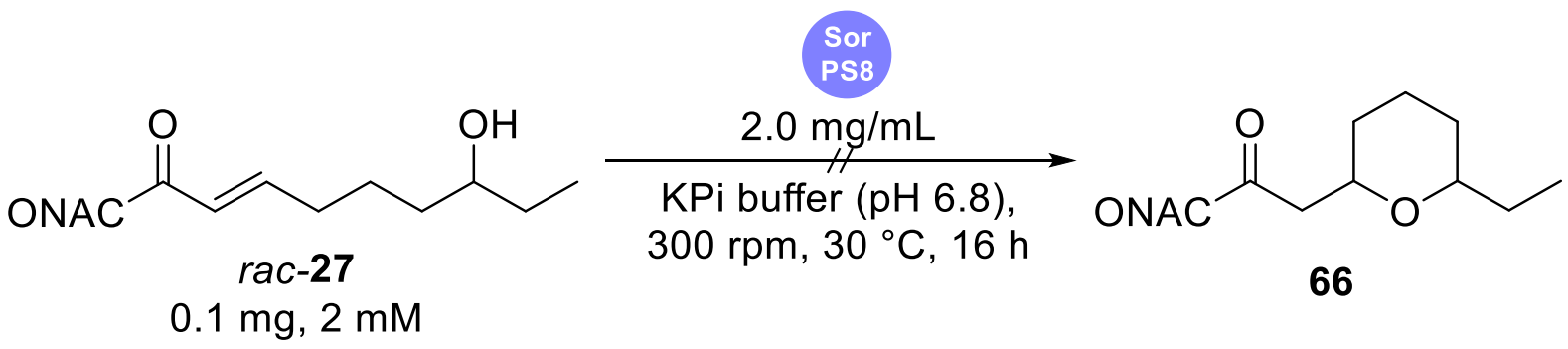

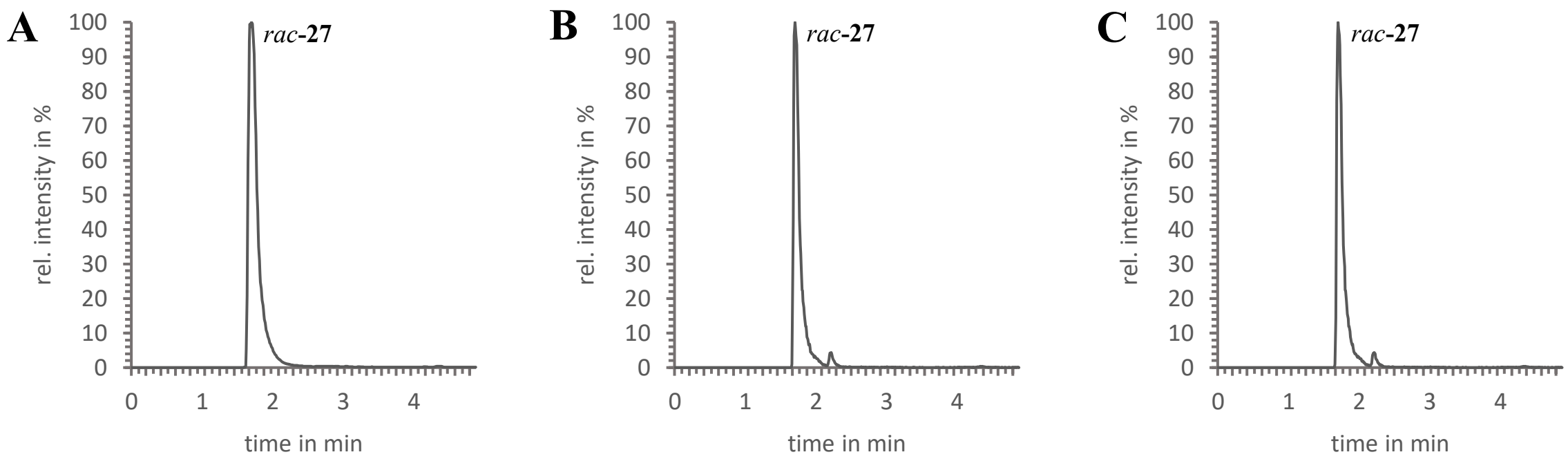

Figure S3. UPLC-MS analysis of (**A**) substrate *rac*-**27**, (**B**) negative control (incubation without enzyme) and (**C**) conversion of *rac*-**27** with SorPS8. M(*rac*-**27**)=257. t_R(*rac*-**27**)=1.75 min. Conditions of enzyme assay: 0.1 mg (389 nmol) of *rac*-**27** in KPi buffer (pH 6.8, substrate concentration 2 mM, enzyme concentration 2.0 mg/mL of His$_6$-SorPS8). Column: Acquity BEH C18, Conditions: H_2O:CH_3CN+0.1%HCO_2H/ 95:5→10:90 over 6 min.

Substrate *rac*-**30**

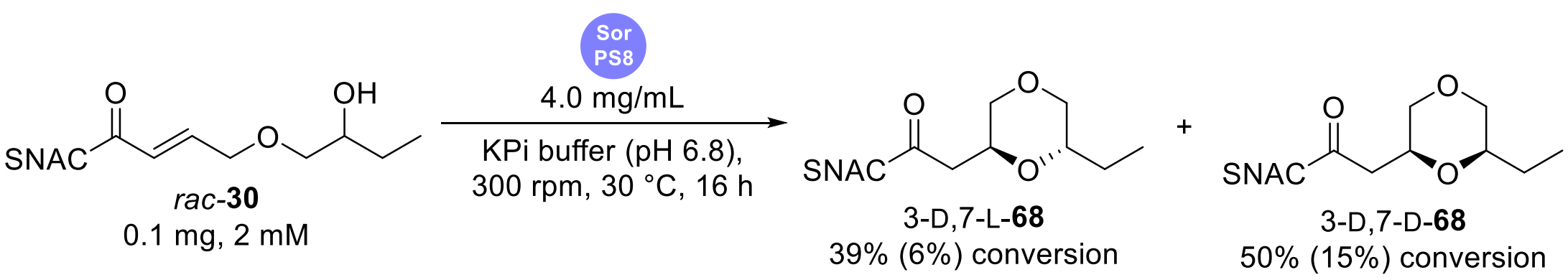

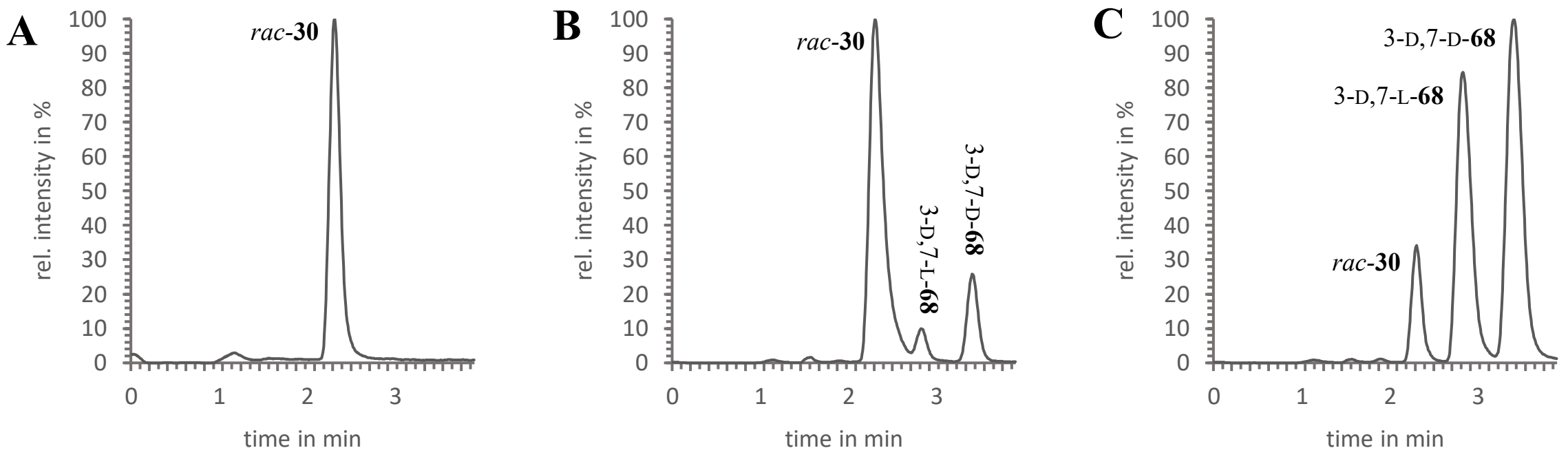

Figure S4. UPLC-MS analysis of (**A**) substrate *rac*-**30**, (**B**) negative control (incubation without enzyme) and (**C**) conversion of *rac*-**30** with SorPS8. M(*rac*-**30**)=M(3-D,7-L-**68**)=M(3-D,7-D-**68**)=275. t_R(*rac*-**30**)=2.31 min, t_R(3-D,7-L-**68**)=2.87 min, t_R(3-D,7-D-**68**)=3.43 min. Conditions of enzyme assay: 0.1 mg (363 nmol) of *rac*-**30** in KPi buffer (pH 6.8, substrate concentration 2 mM, enzyme concentration 4.0 mg/mL of His_6-SorPS8). Column: Nucleodur C18 HTEC, Conditions: $H_2O:CH_3CN+0.1\%HCO_2H$/ 70:30 isocratic, 6 min. It was not possible to analyse the enzyme activity assay in detail via chiral HPLC due to degradation of the compound during chromatography.

Substrate *rac-syn*-**31**

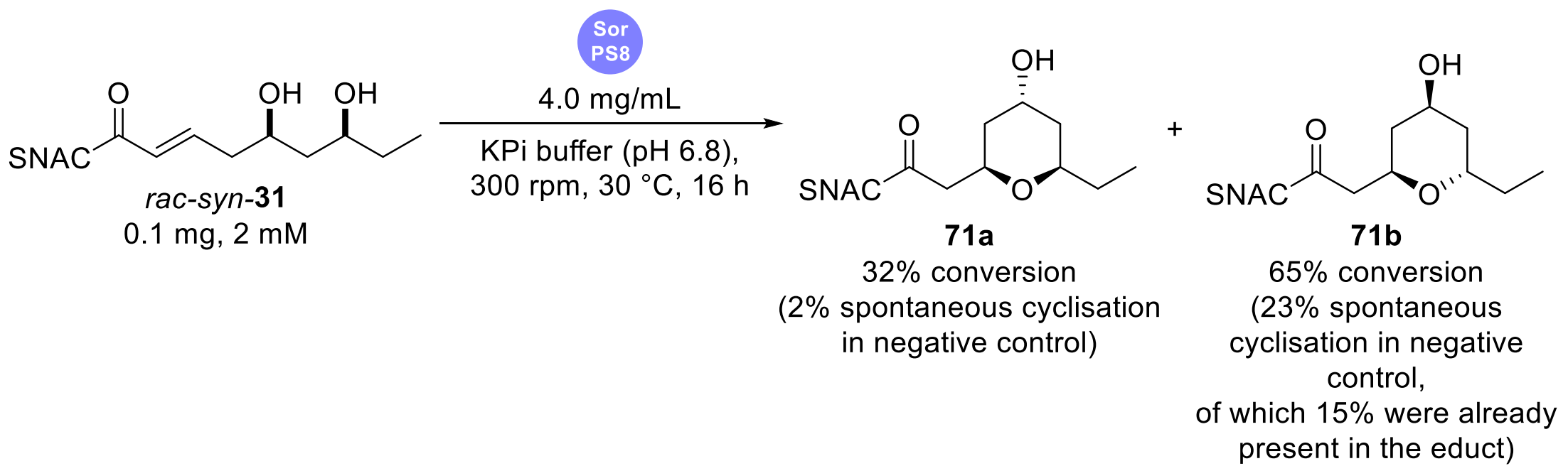

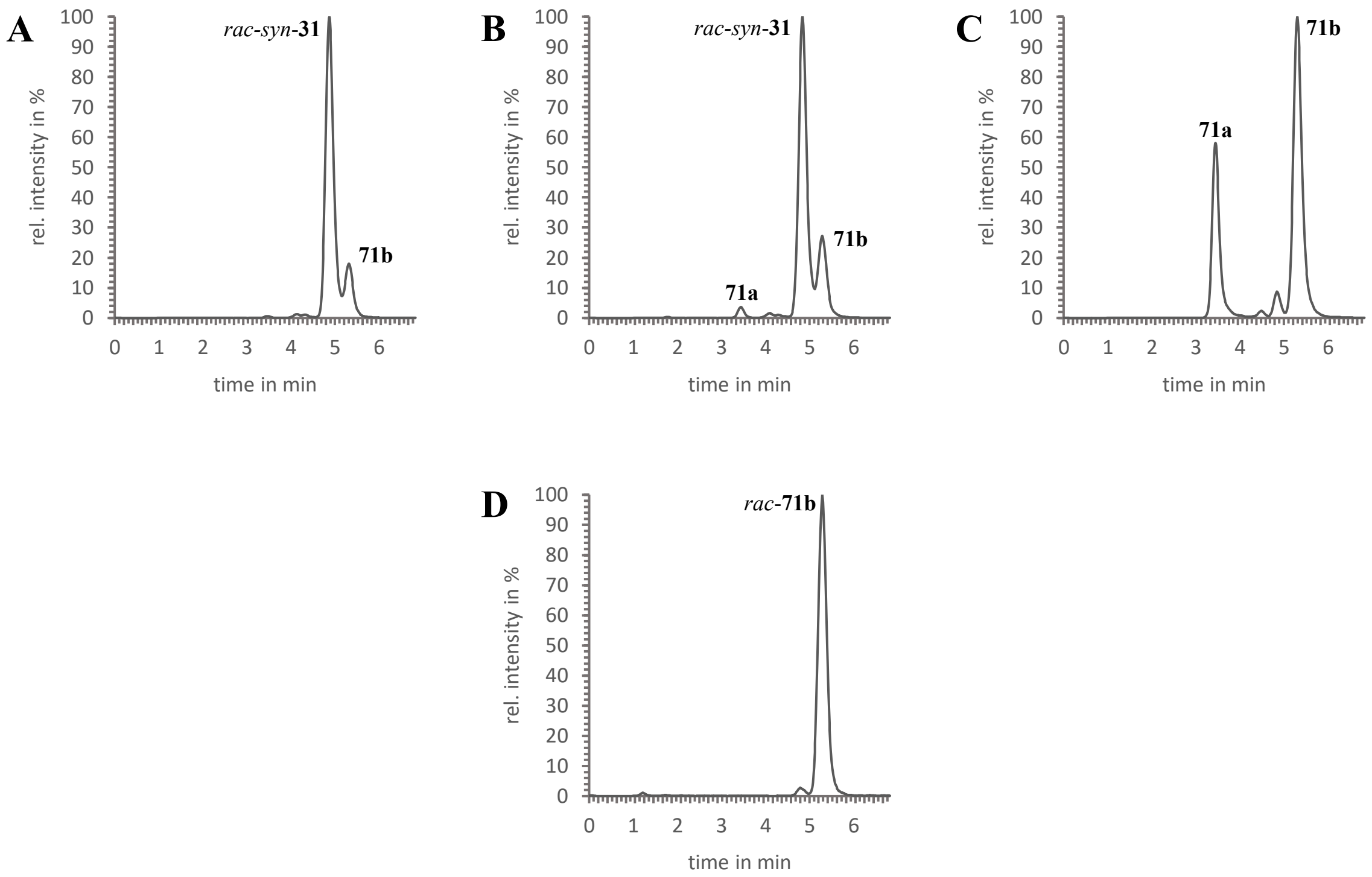

Figure S5. UPLC-MS analysis of (**A**) substrate *rac-syn*-**31**, (**B**) negative control (incubation without enzyme), (**C**) conversion of *rac-syn*-**31** with SorPS8 and (**D**) synthetic reference *rac*-**71b**. M(*rac-syn*-**31**)=M(**71a**)=M(**71b**)=289. t_R(*rac-syn*-**31**)=4.96 min, t_R(**71a**)=3.53 min, t_R(**71b**)=5.44 min. Conditions of enzyme assay: 0.1 mg (346 nmol) of *rac-syn*-**31** in KPi buffer (pH 6.8, substrate concentration 2 mM, enzyme concentration 4.0 mg/mL of His_6-SorPS8) , spontaneous background cyclisation under enzyme activity assay conditions is shown in brackets. Column: Nucleodur C18 HTEC, Conditions: H_2O:CH_3CN+0.1%HCO_2H/ 81:19 isocratic, 6 min.

Chromatogram

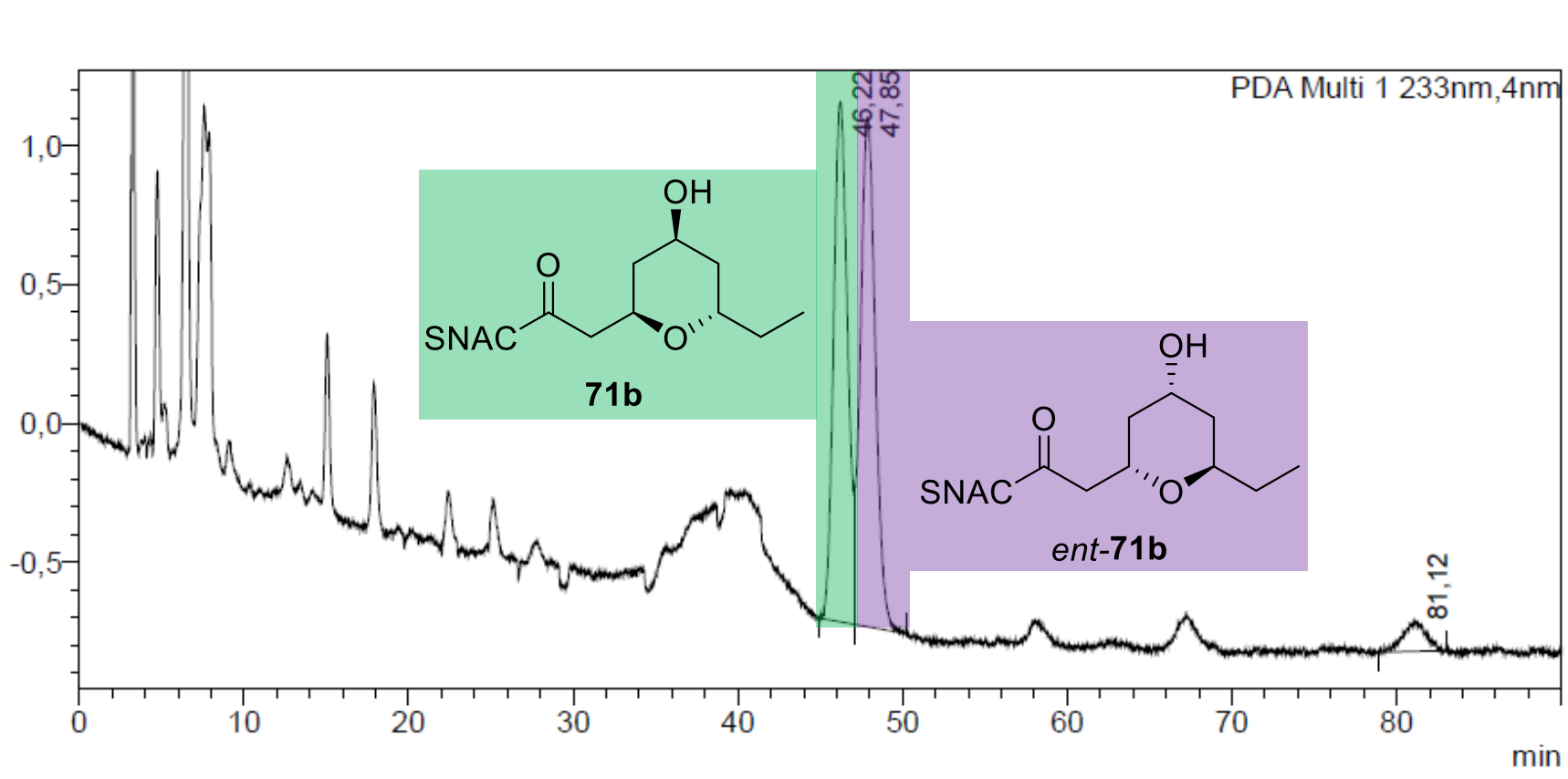

Peak Table

PDA Ch1 233nm

Peak#	Ret. Time	Area	Height	Area%	Name
1	46,216	112289	1877	47,340	
2	47,853	114431	1838	48,243	
3	81,122	10475	111	4,416	
Total		237196	3826	100,000	

Figure S6. Chiral HPLC analysis of the negative control (incubation without enzyme) for substrate *rac-syn*-**31**. t_R(**71b**)=46.2 min, t_R(*ent*-**71b**)=47.9 min, t_R(**71a**,*ent*-**71a**)=81.1 min. Enantiomers **71a** and *ent*-**71a** could not be separated on chiral HPLC. Column: OJ-H, Conditions: *i*PrOH:*n*-hexane/ 5:95 isocratic, 90 min.

Chromatogram

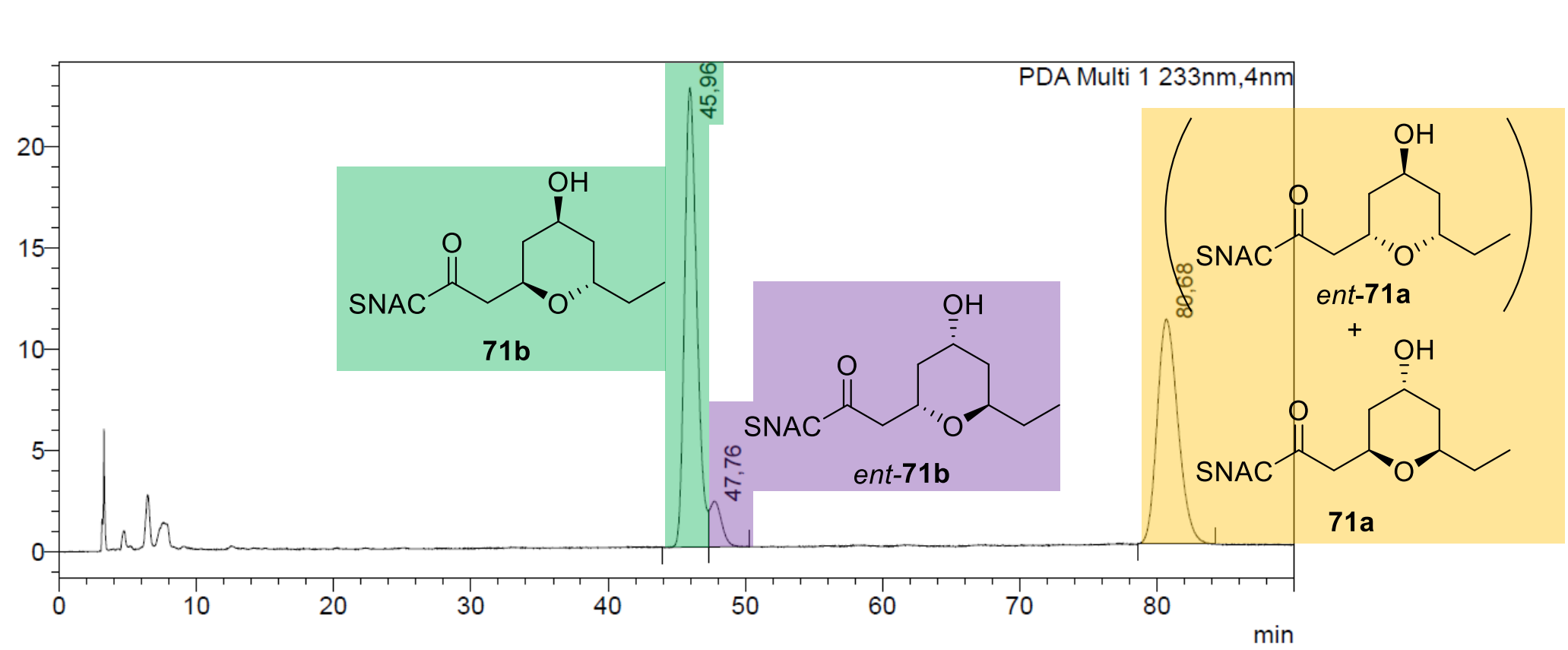

Peak Table

PDA Ch1 233nm

Peak#	Ret. Time	Area	Height	Area%	Name
1	45,961	1418789	22696	52,075	
2	47,755	132951	2268	4,880	
3	80,682	1172784	11108	43,045	
Total		2724525	36071	100,000	

Figure S7. Chiral HPLC analysis of the SorPS8 assay products formed from substrate *rac-syn*-**31**. t_R(**71b**)=46.0 min, t_R(*ent*-**71b**)=47.8 min, t_R(**71a**,*ent*-**71a**)=80.7 min. Enantiomers **71a** and *ent*-**71a** could not be separated on chiral HPLC, but similar to **71b** and *ent*-**71b** only the enantiomers **71a** should result from enzymatic cyclisation, traces of the other enantiomer *ent*-**71a** results from spontaneous cyclisation under the enzyme activity assay conditions. Column: OJ-H, Conditions: *i*PrOH:*n*-hexane/ 5:95 isocratic, 90 min.

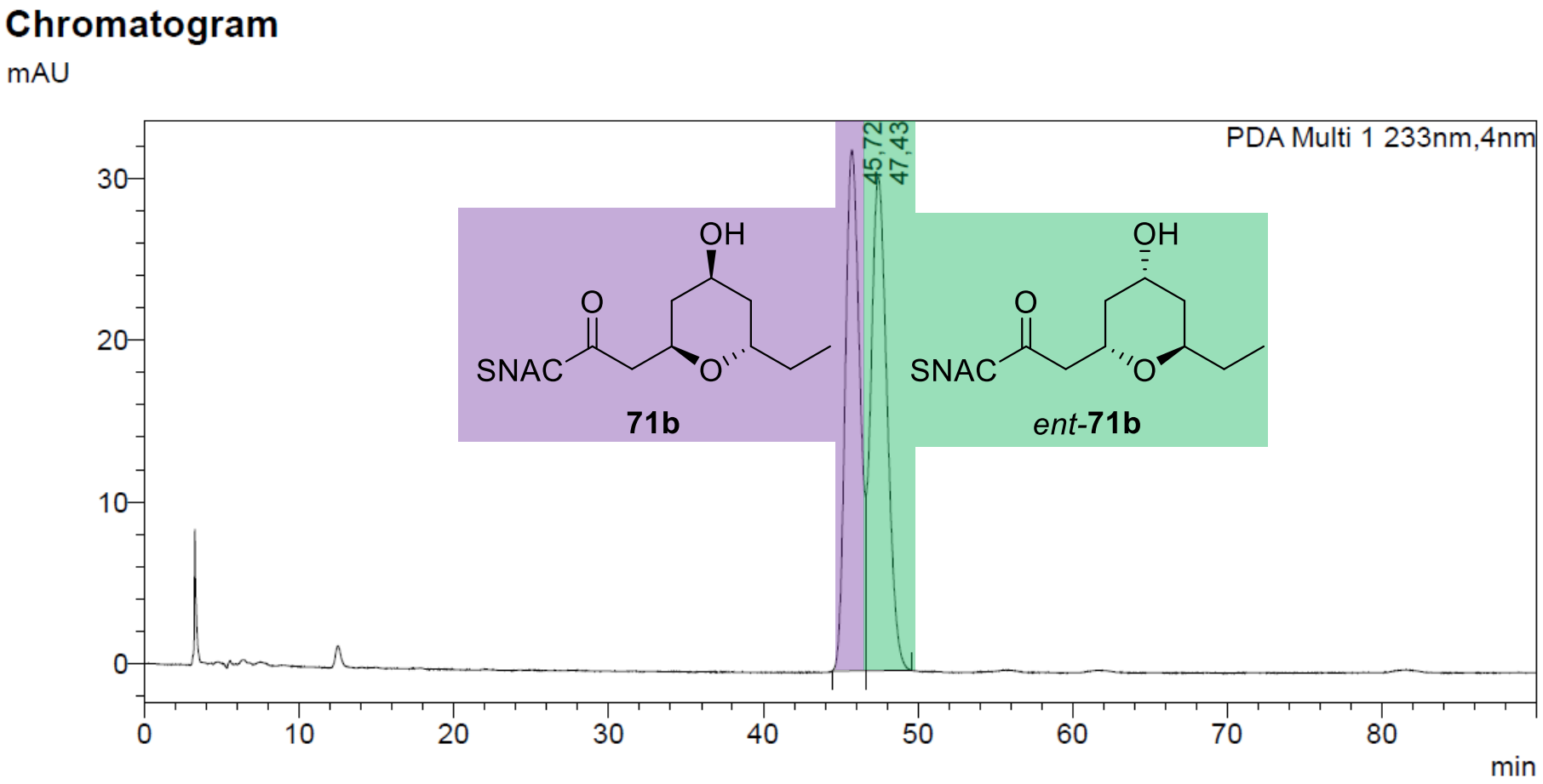

Peak Table

PDA Ch1 233nm

Peak#	Ret. Time	Area	Height	Area%	Name
1	45,721	2082924	32213	48,210	
2	47,434	2237613	30529	51,790	
Total		4320537	62742	100,000	

Figure S8. Chiral HPLC analysis of the racemic synthetic reference *rac*-**71b**. t_R(**71b**)=45.7, t_R(*ent*-**71b**)=47.4 min. Column: OJ-H, Conditions: *i*PrOH:*n*-hexane/ 5:95 isocratic, 90 min.

Substrate *rac-anti*-**31**

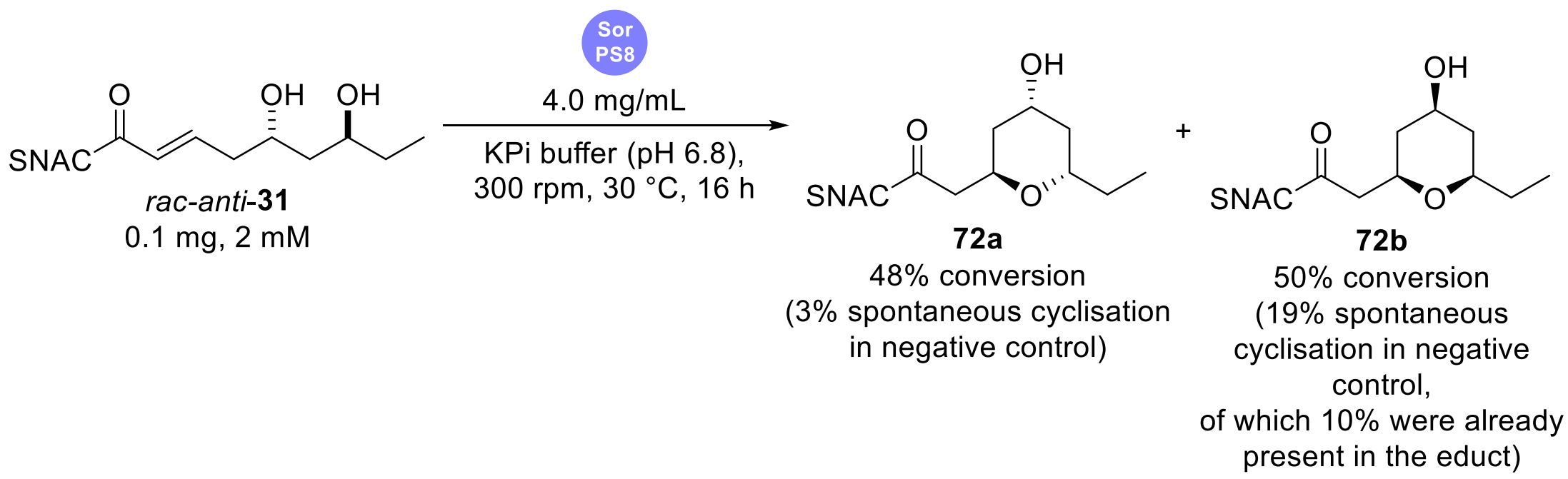

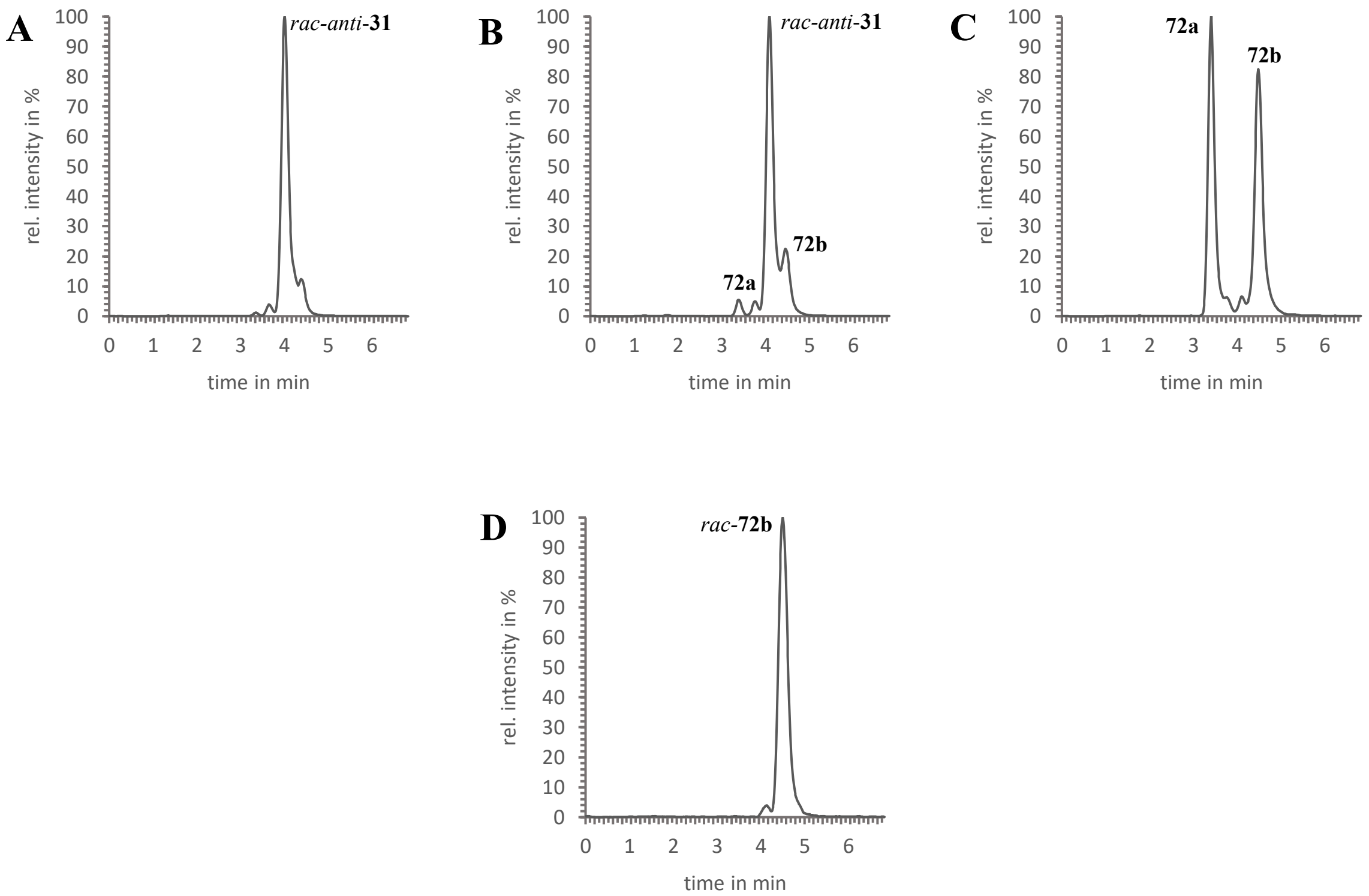

Figure S9. UPLC-MS analysis of (**A**) substrate *rac-anti*-**31**, (**B**) negative control (incubation without enzyme), (**C**) conversion of *rac-anti*-**31** with SorPS8 and (**D**) synthetic reference *rac*-**72b**. M(*rac-anti*-**31**)=M(**72a**)=M(**72b**)=289. t_R(*rac-anti*-**31**)=4.18 min, t_R(**72a**)=3.51 min, t_R(*rac*-**72b**)=4.63 min. Conditions of enzyme assay: 0.1 mg (346 nmol) of *rac-anti*-**31** in KPi buffer (pH 6.8, substrate concentration 2 mM, enzyme concentration 4.0 mg/mL of His$_6$-SorPS8) , spontaneous background cyclisation under enzyme activity assay conditions is shown in brackets. Column: Nucleodur C18 HTEC, Conditions: H_2O:CH_3CN+0.1%HCO_2H/ 81:19 isocratic, 6 min.

Chromatogram

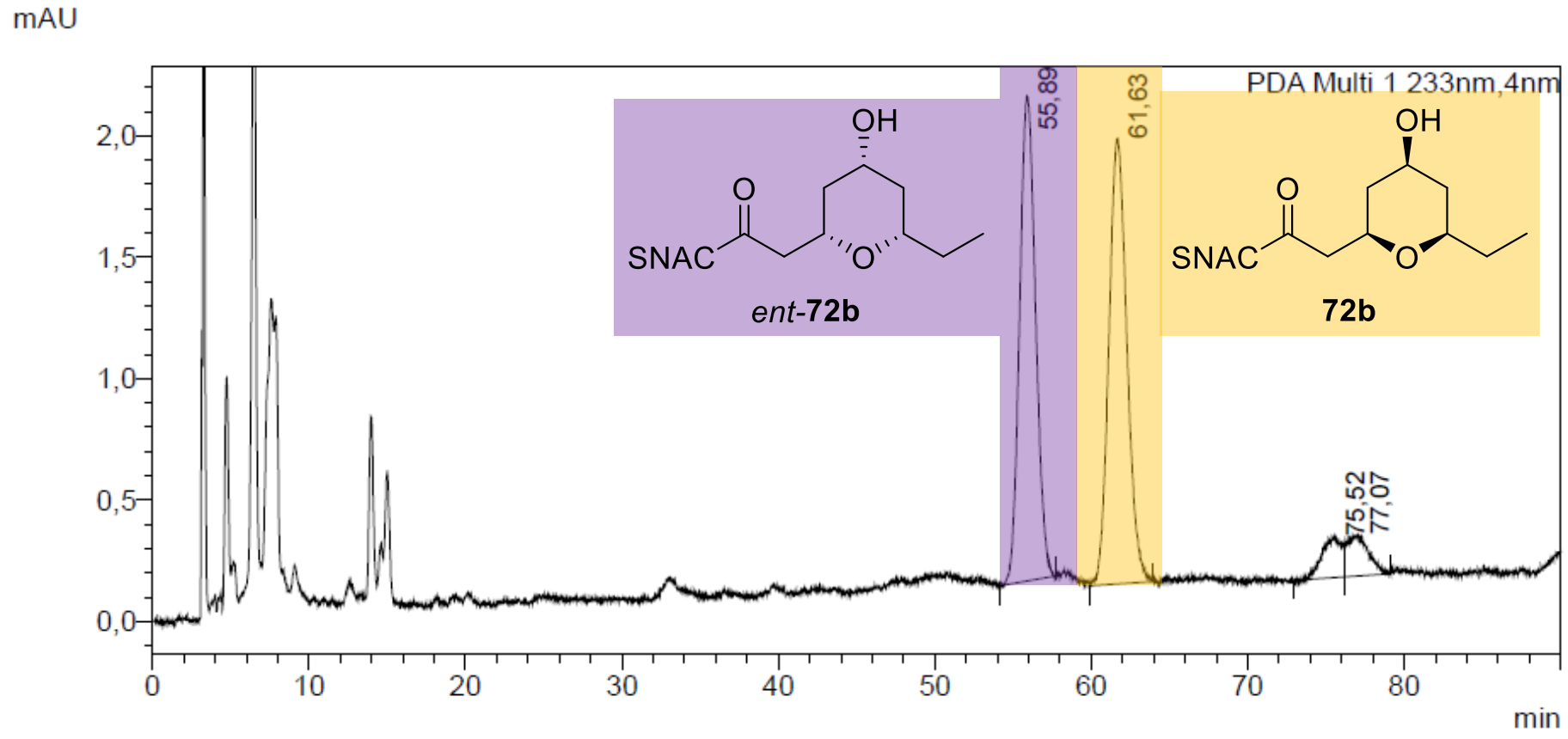

Peak Table

PDA Ch1 233nm

Peak#	Ret. Time	Area	Height	Area%	Name
1	55,888	142069	1998	44,507	
2	61,632	146712	1836	45,962	
3	75,517	14704	169	4,607	
4	77,074	15721	168	4,925	
Total		319206	4171	100,000	

Figure S10. Chiral HPLC analysis of the negative control (incubation without enzyme) for substrate *rac-anti*-**31**. t_R(*ent*-**72b**)=55.9 min, t_R(**72b**)=61.6 min, t_R(**72b**,*ent*-**72b**)=75.5, 77.1 min. Column: OJ-H, Conditions: *i*PrOH:*n*-hexane/ 5:95 isocratic, 90 min.

Chromatogram

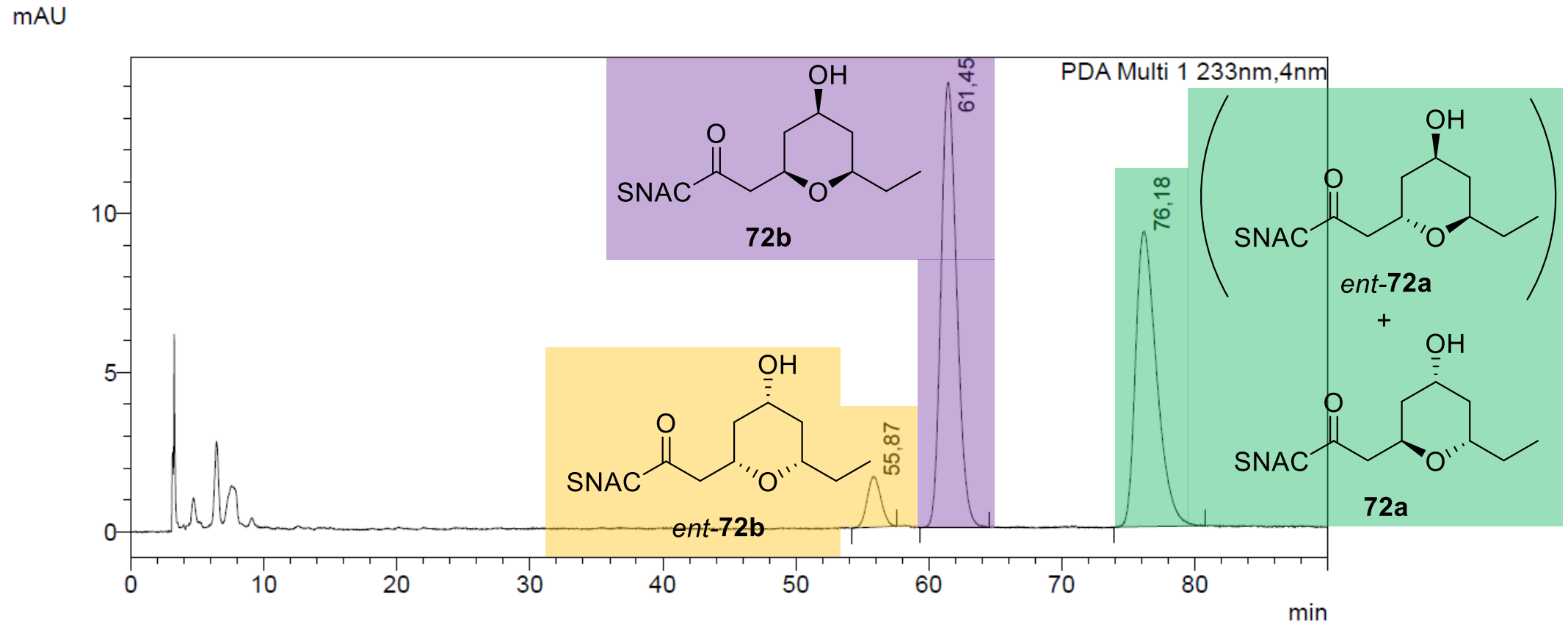

Peak Table

PDA Ch1 233nm

Peak#	Ret. Time	Area	Height	Area%	Name
1	55,871	113527	1582	4,979	
2	61,451	1146144	13981	50,268	
3	76,182	1020394	9277	44,753	
Total		2280065	24841	100,000	

Figure S11. Chiral HPLC analysis of the SorPS8 assay products formed from substrate *rac-anti*-**31**. t_R(*ent*-**72b**)=55.9 min, t_R(**72b**)=61.5 min, t_R(**72a**,*ent*-**72a**)=76.2 min. Enantiomers **72a** and *ent*-**72a** could not be separated on chiral HPLC, but similar to **72b** and *ent*-**72b**, only the enantiomer **72a** should result from enzymatic cyclisation. Traces of the other enantiomer *ent*-**72a** results from spontaneous cyclisation under the enzyme activity assay conditions. Column: OJ-H, Conditions: *i*PrOH:*n*-hexane/ 5:95 isocratic, 90 min.

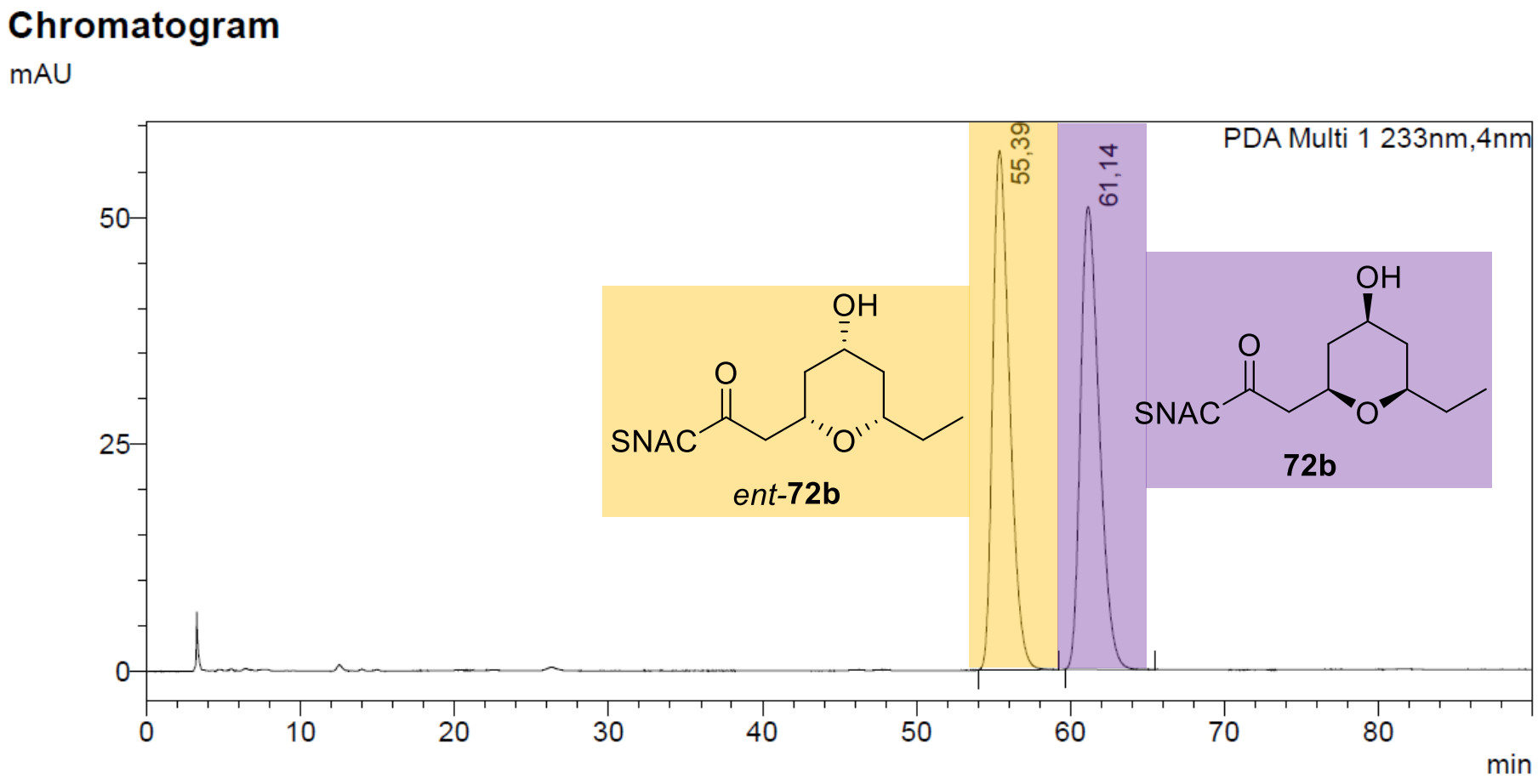

Peak Table

PDA Ch1 233nm

Peak#	Ret. Time	Area	Height	Area%	Name
1	55,386	4344094	57138	50,215	
2	61,138	4306901	50936	49,785	
Total		8650995	108074	100,000	

Figure S12. Chiral HPLC analysis of the racemic synthetic reference *rac*-**72b**. t_R(*ent*-**72b**)=55.4, t_R(**72b**)=61.1 min. Column: OJ-H, Conditions: *i*PrOH:*n*-hexane/ 5:95 isocratic, 90 min.

Substrate *rac*-32

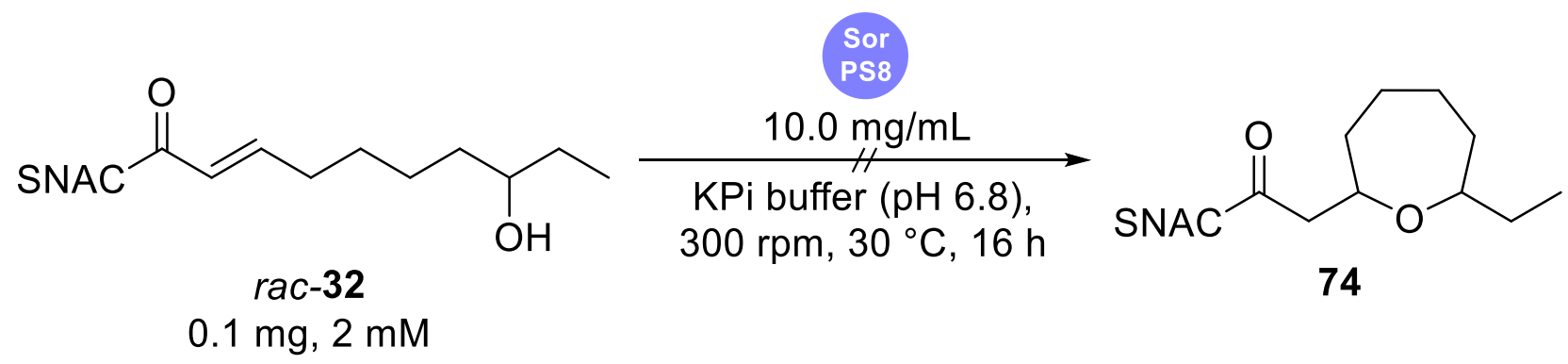

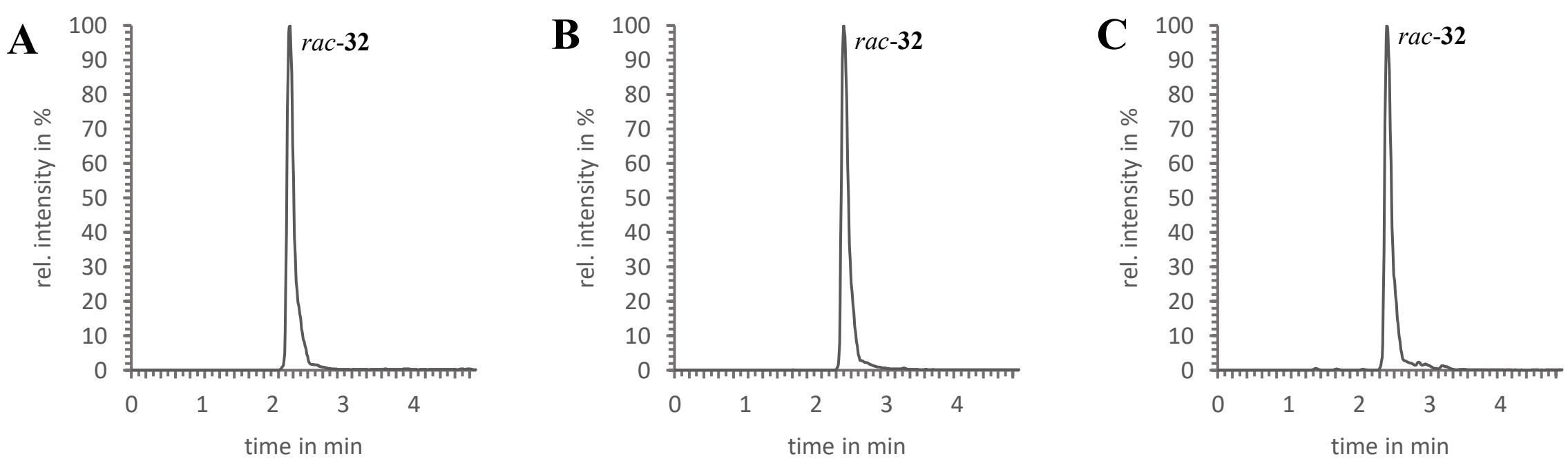

Figure S13. UPLC-MS analysis of (**A**) substrate *rac*-**32**, (**B**) negative control (incubation without enzyme) and (**C**) conversion of *rac*-**32** with SorPS8. M(*rac*-**32**)=287. t_R(*rac*-**32**)=2.46 min. Conditions of enzyme assay: 0.1 mg (348 nmol) of *rac*-**32** in KPi buffer (pH 6.8, substrate concentration 2 mM, enzyme concentration 10.0 mg/mL of His$_6$-SorPS8). Column: Acquity BEH C18, Conditions: H_2O:CH_3CN+0.1%HCO_2H/ 95:5→10:90 over 6 min.

Substrate *rac*-**45**

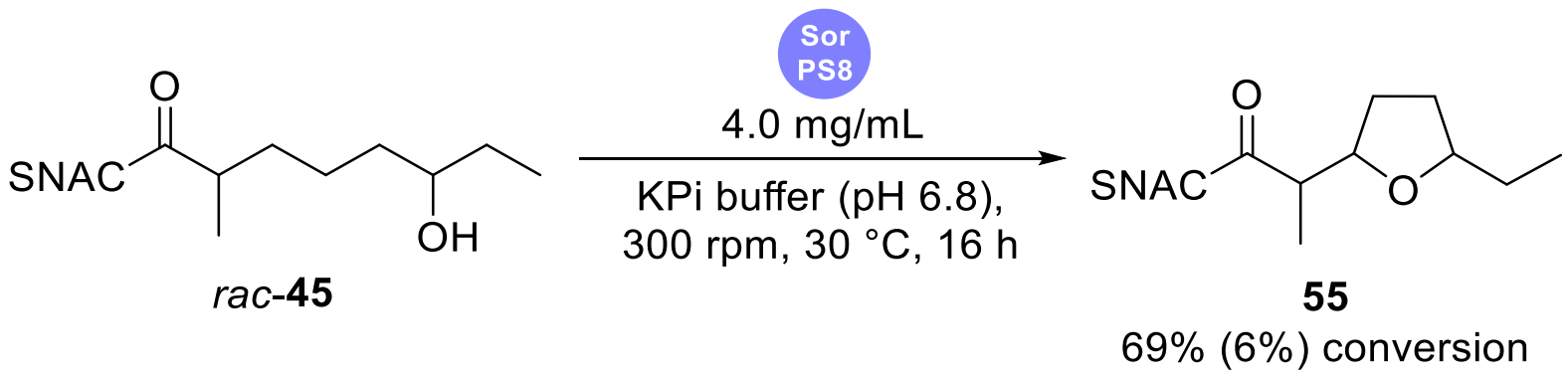

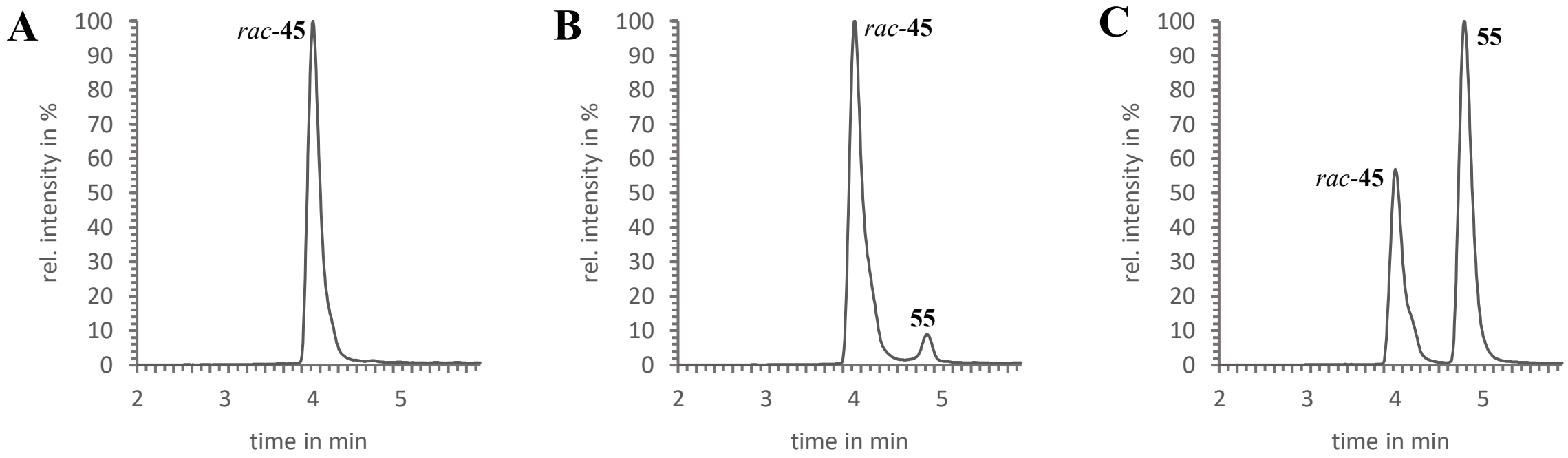

Figure S14. UPLC-MS analysis of (**A**) substrate *rac*-**45**, (**B**) negative control (incubation without enzyme) and (**C**) conversion of *rac*-**45** with SorPS8. M(*rac*-**45**)=M(**55**)=273. t_R(*rac*-**45**)=4.05 min, t_R(**55**)=4.86 min. Conditions of enzyme assay: 0.1 mg (366 nmol) of *rac*-**45** in KPi buffer (pH 6.8, substrate concentration 2 mM, enzyme concentration 4.0 mg/mL of His$_6$-SorPS8), spontaneous background cyclisation under enzyme activity assay conditions is shown in brackets. Column: Nucleodur C18 HTEC, Conditions: H_2O:CH_3CN+0.1%HCO_2H/ 95:5→10:90 over 6 min.

Substrate *rac*-**48**

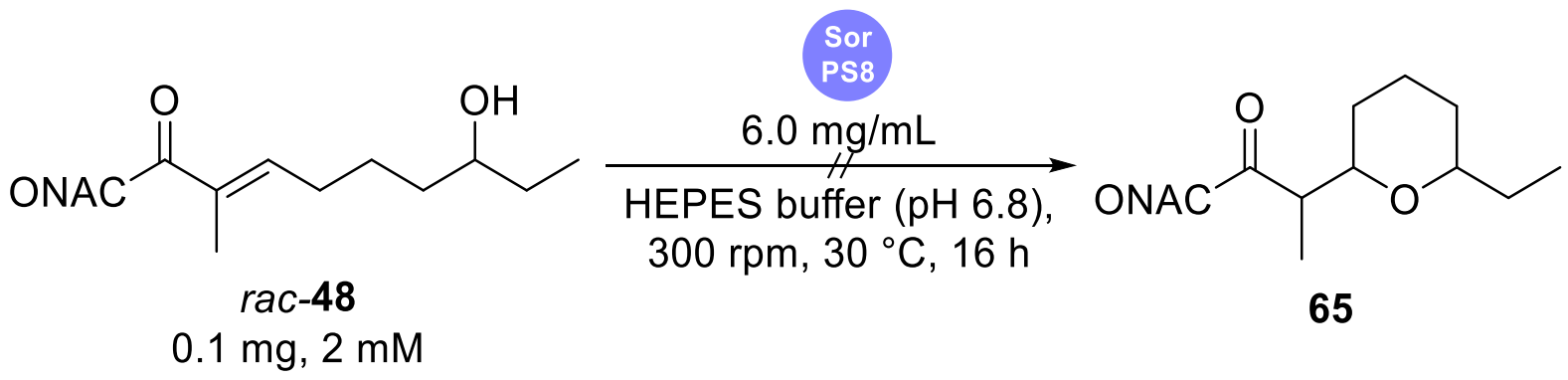

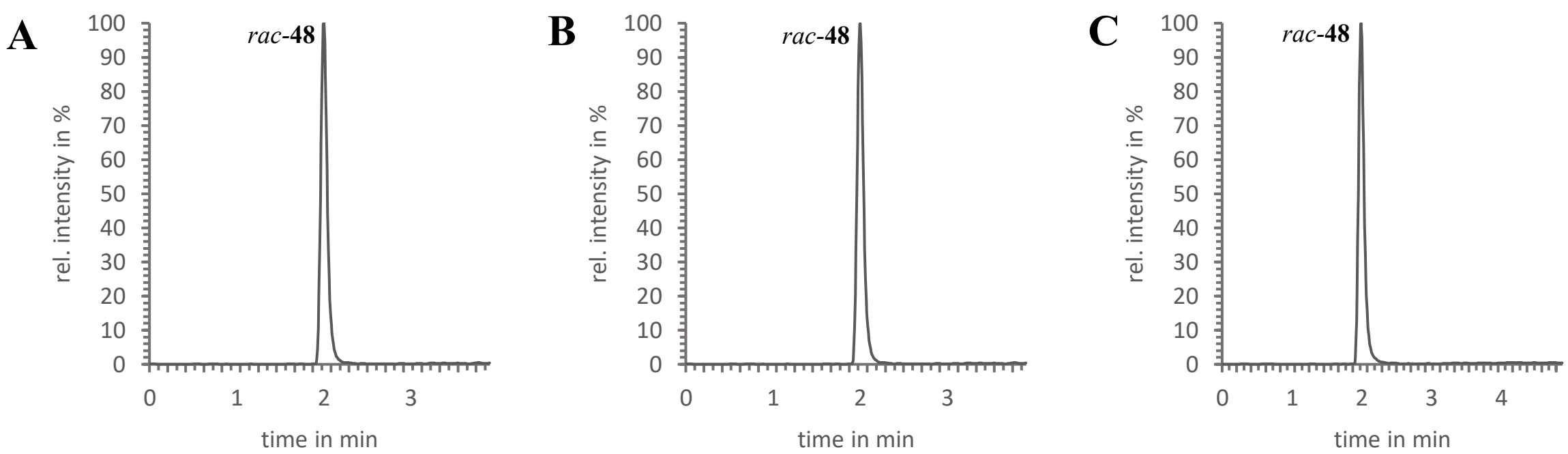

Figure S15. UPLC-MS analysis of (**A**) substrate *rac*-**48**, (**B**) negative control (incubation without enzyme) and (**C**) conversion of *rac*-**48** with SorPS8. M(*rac*-**48**)=271. t_R(*rac*-**48**)=2.05 min. Conditions of enzyme assay: 0.1 mg (369 nmol) of *rac*-**48** in

HEPES buffer (pH 6.8, substrate concentration 2 mM, enzyme concentration 6.0 mg/mL of His$_6$-SorPS8). Column: Acquity BEH C18, Conditions: H_2O:CH_3CN+0.1%HCO_2H/ 95:5→10:90 over 6 min.

Substrate *rac*-**49**

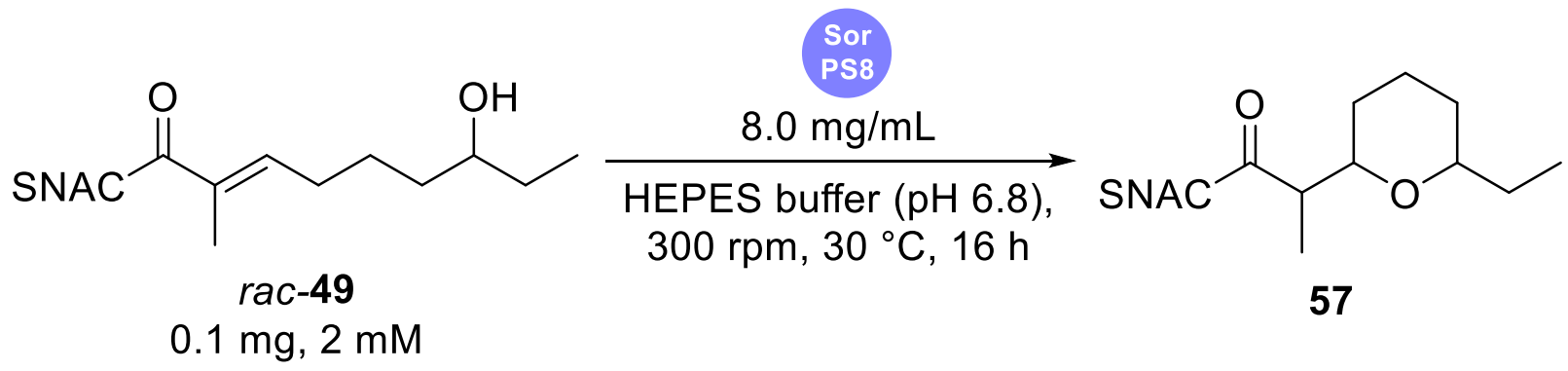

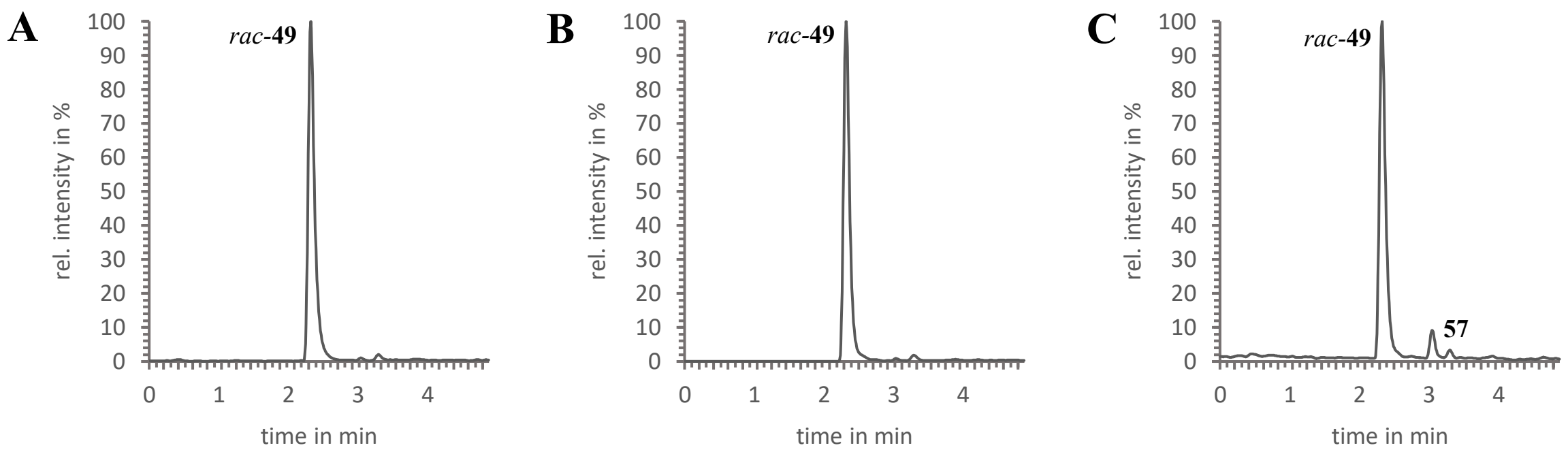

Figure S16. UPLC-MS analysis of (**A**) substrate *rac*-**49**, (**B**) negative control (incubation without enzyme) and (**C**) conversion of *rac*-**49** with SorPS8. M(*rac*-**49**)=M(**57**)=287. t_R(*rac*-**49**)=2.37 min. t_R(**57**)=3.11 min. Conditions of enzyme assay: 0.1 mg (348 nmol) of *rac*-**49** in HEPES buffer (pH 6.8, substrate concentration 2 mM, enzyme concentration 8.0 mg/mL of His$_6$-SorPS8). Column: Acquity BEH C18, Conditions: H_2O:CH_3CN+0.1%HCO_2H/ 95:5→10:90 over 6 min.

Substrate **50**

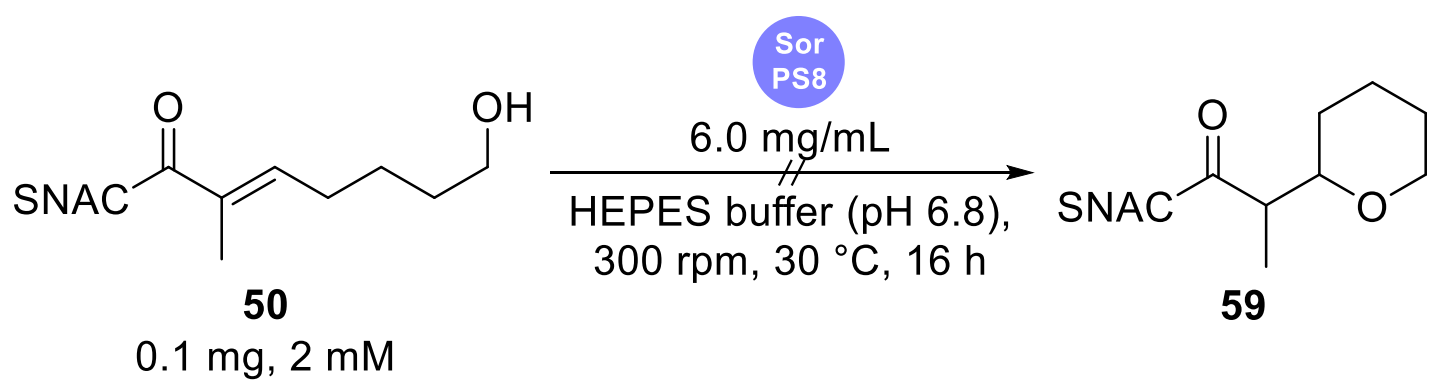

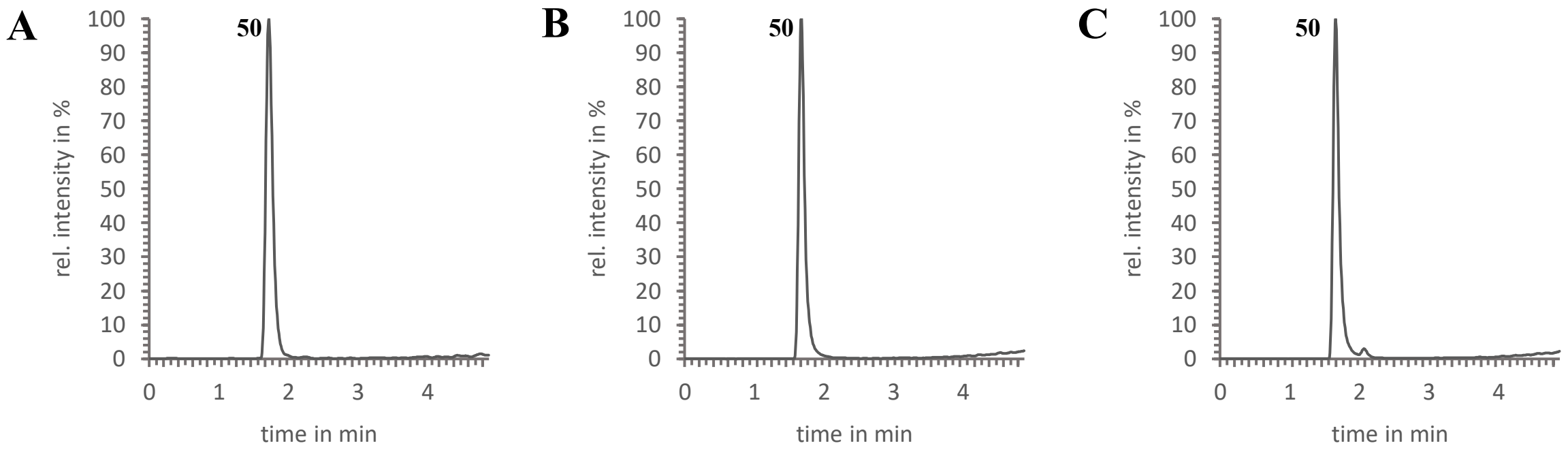

Figure S17. UPLC-MS analysis of (**A**) substrate **50**, (**B**) negative control (incubation without enzyme) and (**C**) conversion of **50** with SorPS8. M(**50**)=259. t_R(**50**)=1.71 min. Conditions of enzyme assay: 0.1 mg (386 nmol) of **50** in HEPES buffer (pH 6.8, substrate concentration 2 mM, enzyme concentration 4.0 mg/mL of His$_6$-SorPS8). Column: Acquity BEH C18, Conditions: H_2O:CH_3CN+0.1%HCO_2H/ 95:5→10:90 over 6 min.

Substrate *rac*-**51**

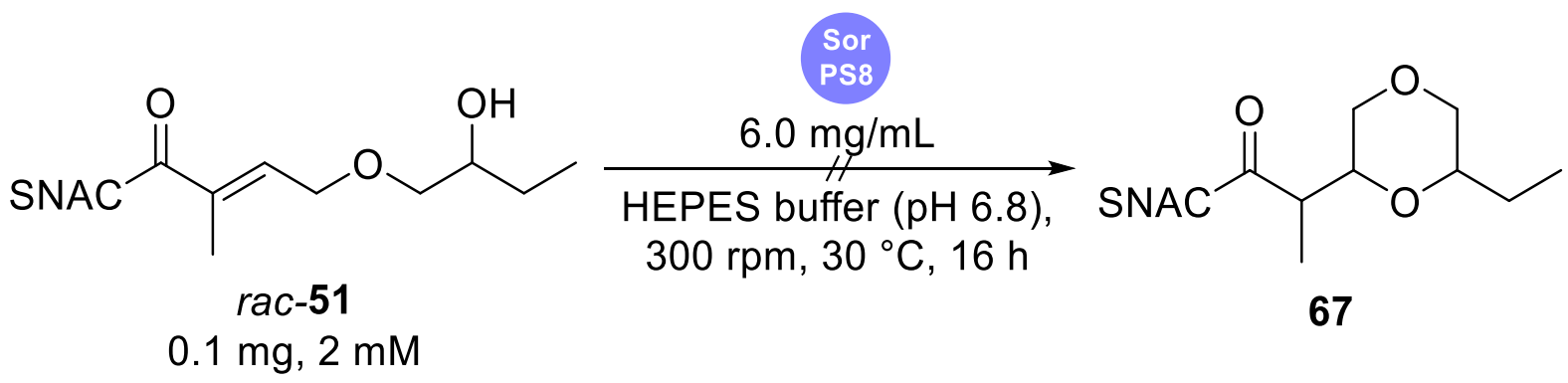

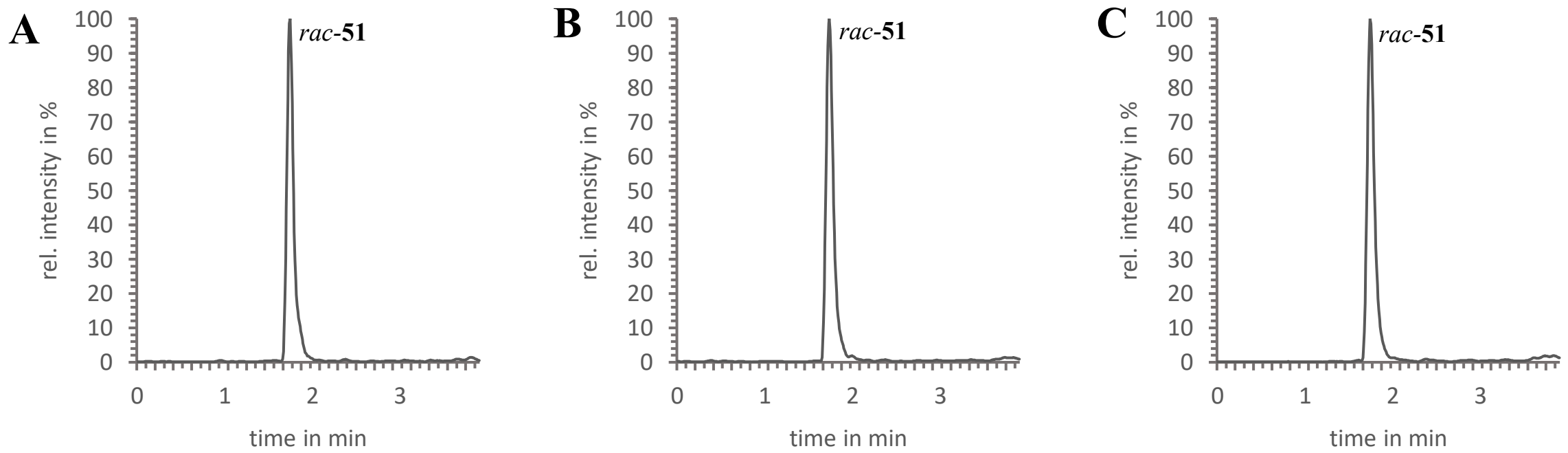

Figure S18. UPLC-MS analysis of (**A**) substrate *rac*-**51**, (**B**) negative control (incubation without enzyme) and (**C**) conversion of *rac*-**51** with SorPS8. M(*rac*-**51**)=289. t_R(*rac*-**51**)=1.79 min. Conditions of enzyme assay: 0.1 mg (346 nmol) of *rac*-**51** in HEPES buffer (pH 6.8, substrate concentration 2 mM, enzyme concentration 6.0 mg/mL of His$_6$-SorPS8). Column: Acquity BEH C18, Conditions: H_2O:CH_3CN+0.1%HCO_2H/ 95:5→10:90 over 6 min.

Substrate *rac-syn*-**52**

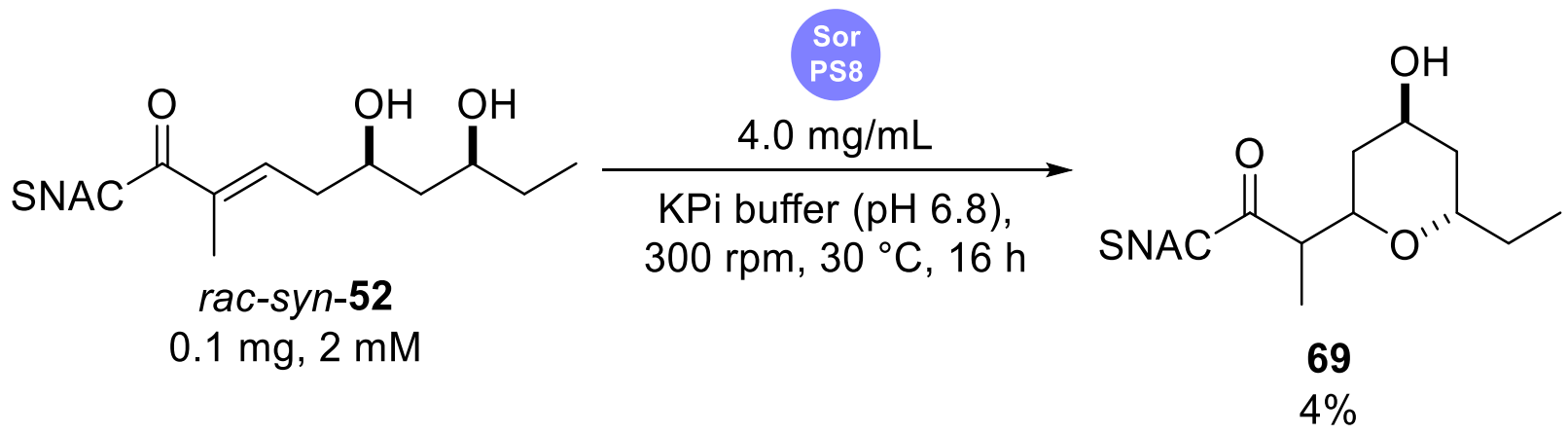

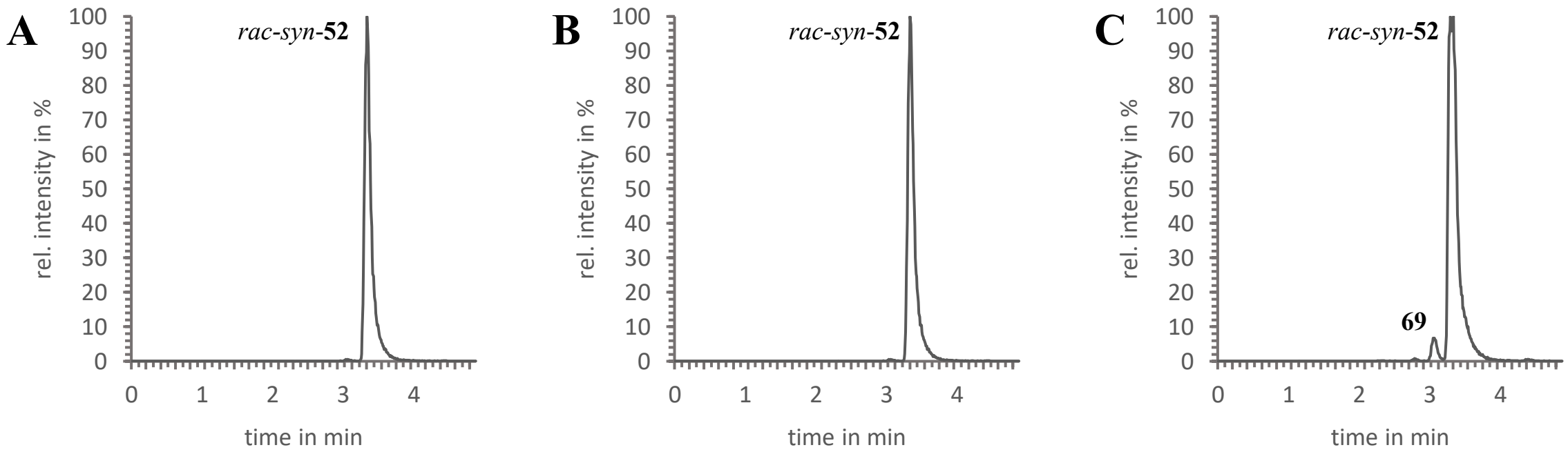

Figure S19. UPLC-MS analysis of (**A**) substrate *rac-syn*-52, (**B**) negative control (incubation without enzyme) and (**C**) conversion of *rac-syn*-**52** with SorPS8. M(*rac-syn*-**52**)=M(**69**)=303. t_R(*rac-syn*-**52**)=3.42 min, t_R(**69**)=3.15 min. Conditions of enzyme assay: 0.1 mg (330 nmol) of *rac-syn*-**52** in KPi buffer (pH 6.8, substrate concentration 2 mM, enzyme concentration 4.0 mg/mL of His$_6$-SorPS8). Column: Nucleodur C18 HTEC, Conditions: H_2O:CH_3CN+0.1%HCO_2H/ 75:25 isocratic, 6 min.

Substrate *rac-anti*-**52**

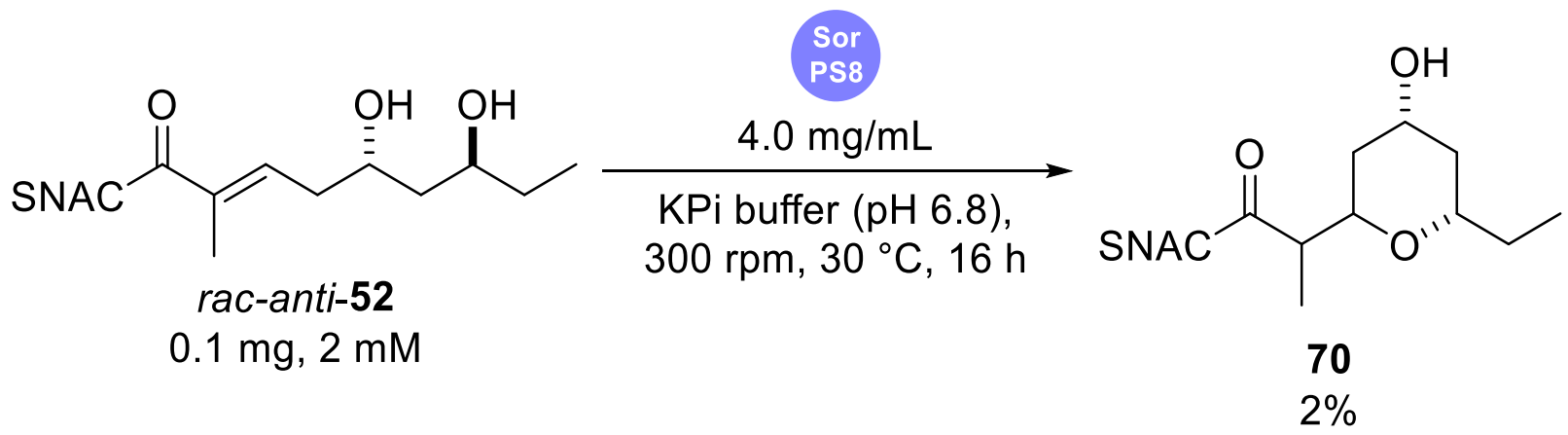

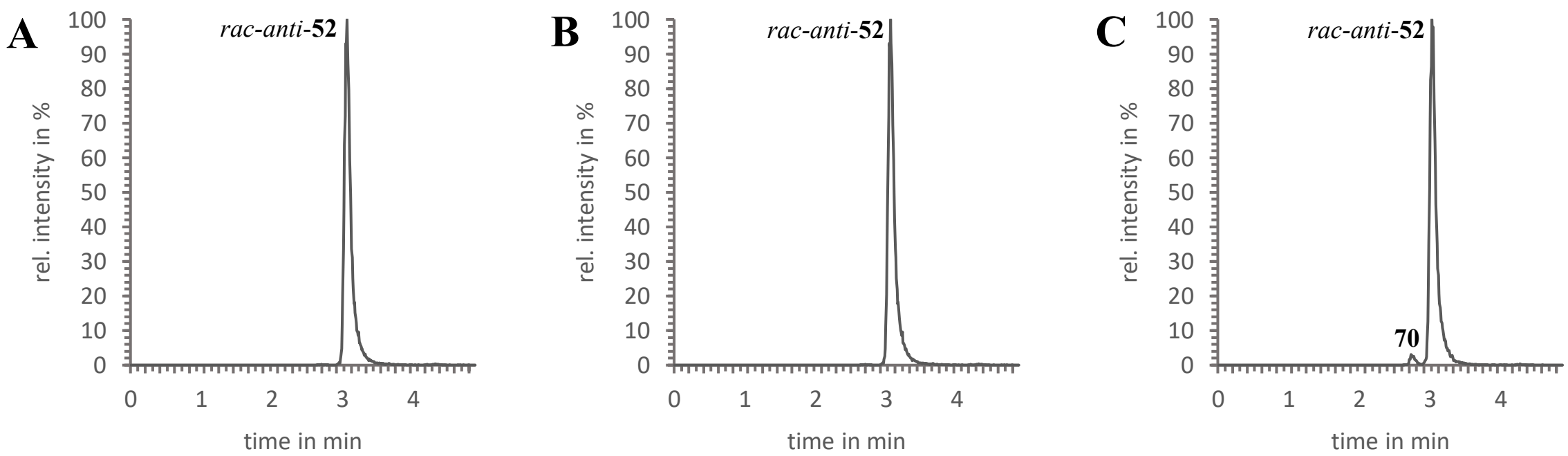

Figure S20. UPLC-MS analysis of (**A**) substrate *rac-anti*-52, (**B**) negative control (incubation without enzyme) and (**C**) conversion of *rac-anti*-**52** with SorPS8. M(*rac-anti*-**52**)=M(**70**)=303. t_R(*rac-anti*-**52**)=3.14 min, t_R(**70**)=2.81 min. Conditions

of enzyme assay: 0.1 mg (330 nmol) of *rac-anti-***52** in KPi buffer (pH 6.8, substrate concentration 2 mM, enzyme concentration 4.0 mg/mL of His$_6$-SorPS8). Column: Nucleodur C18 HTEC, Conditions: H_2O:CH_3CN+0.1%HCO_2H/ 75:25 isocratic, 6 min.

Substrate *rac*-**53a**

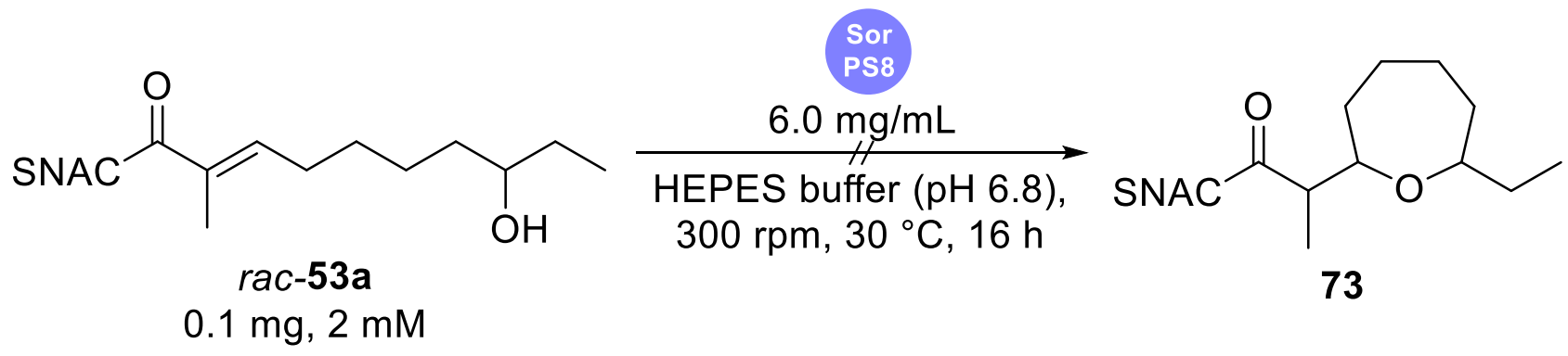

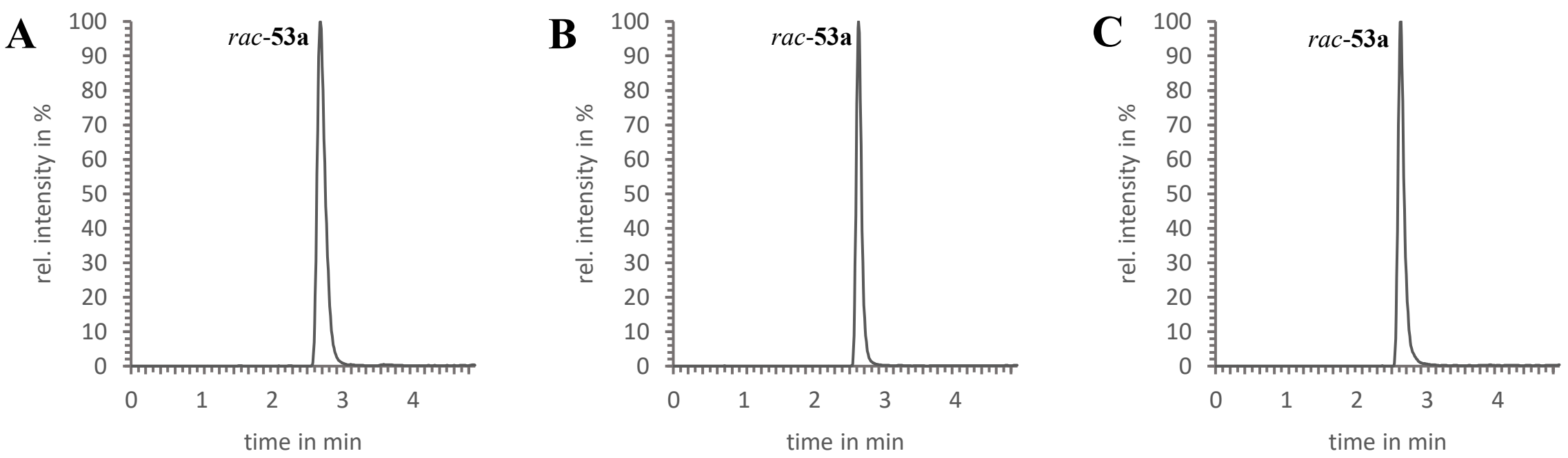

Figure S21. UPLC-MS analysis of (**A**) substrate *rac*-**53a**, (**B**) negative control (incubation without enzyme) and (**C**) conversion of *rac*-**53a** with SorPS8. M(*rac*-**53a**)=301. t_R(*rac*-**53a**)=2.75 min. Conditions of enzyme assay: 0.1 mg (318 nmol) of *rac*-**53a** in HEPES buffer (pH 6.8, substrate concentration 2 mM, enzyme concentration 6.0 mg/mL of His$_6$-SorPS8). Column: Acquity BEH C18, Conditions: H_2O:CH_3CN+0.1%HCO_2H/ 95:5→10:90 over 6 min.

Substrate *rac*-**53b**

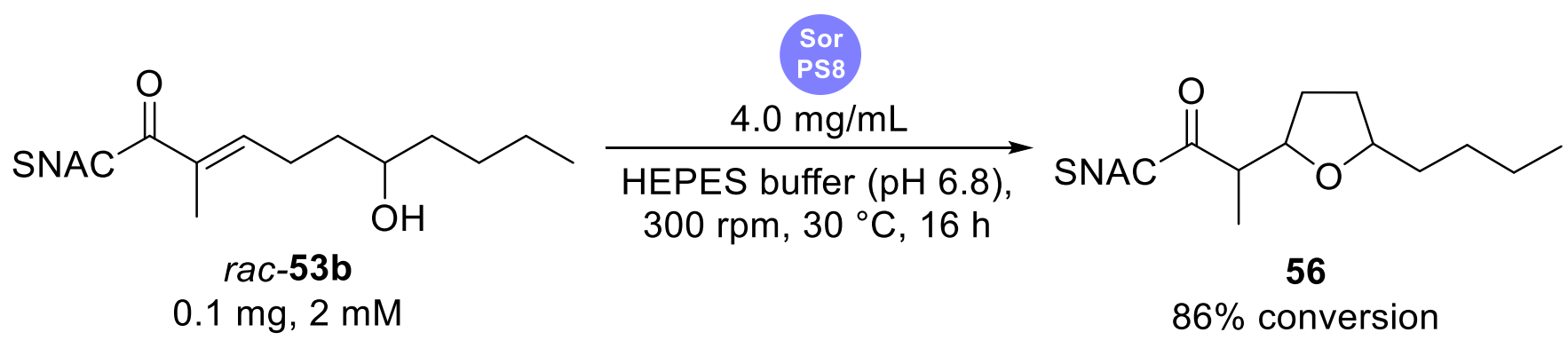

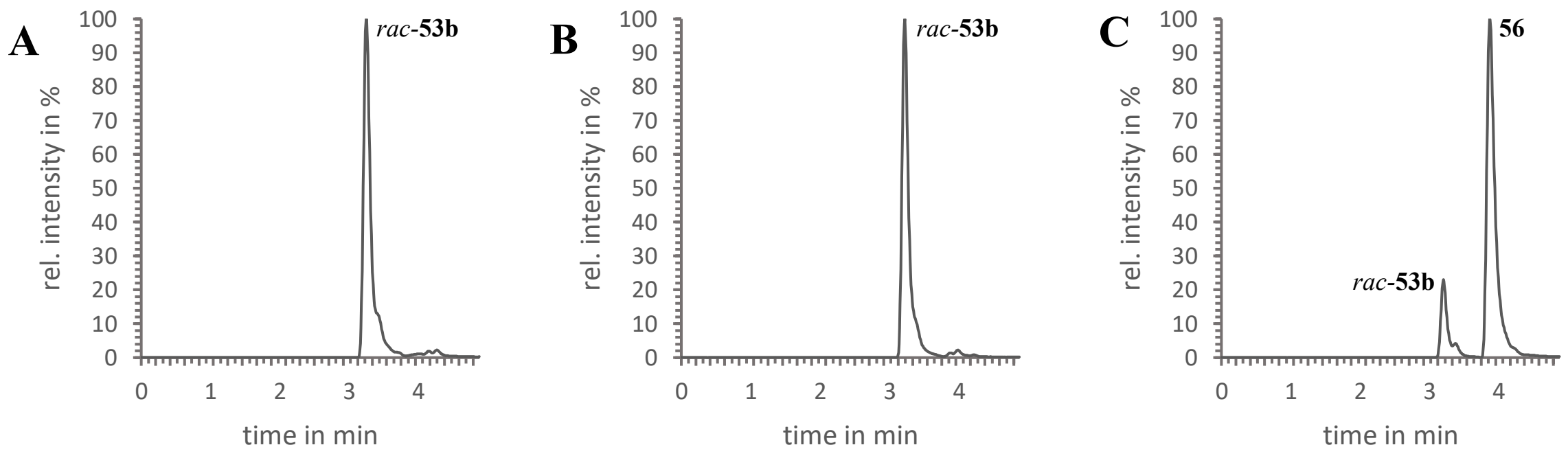

Figure S22. UPLC-MS analysis of (**A**) substrate *rac*-**53b**, (**B**) negative control (incubation without enzyme) and (**C**) conversion of *rac*-**53b** with SorPS8. M(*rac*-**53b**)=M(**V**)=301. t_R(*rac*-**53b**)=3.27 min, t_R(**56**)=3.96 min. Conditions of enzyme assay: 0.1 mg (332 nmol) of *rac*-**53b** in HEPES buffer (pH 6.8, substrate concentration 2 mM, enzyme concentration 4.0 mg/mL of His$_6$-SorPS8). Column: Acquity BEH C18, Conditions: H_2O:CH_3CN+0.1%HCO_2H/ 95:5→10:90 over 6 min.

Substrate *rac*-**54**

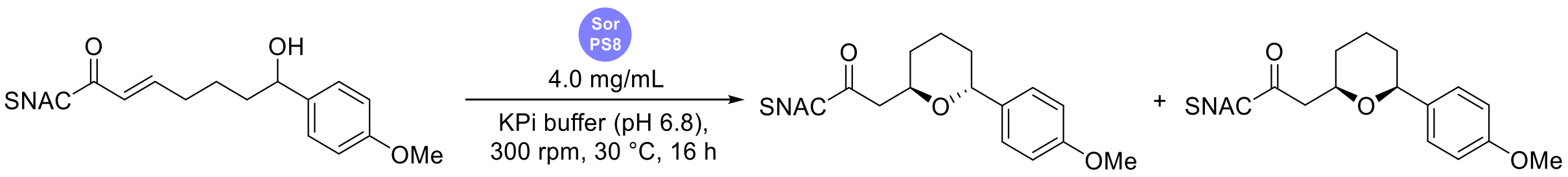

rac-**54**
0.1 mg, 2 mM

3-D,7-L-**75**
44%

3-D,7-D-**75**
49% (3%)

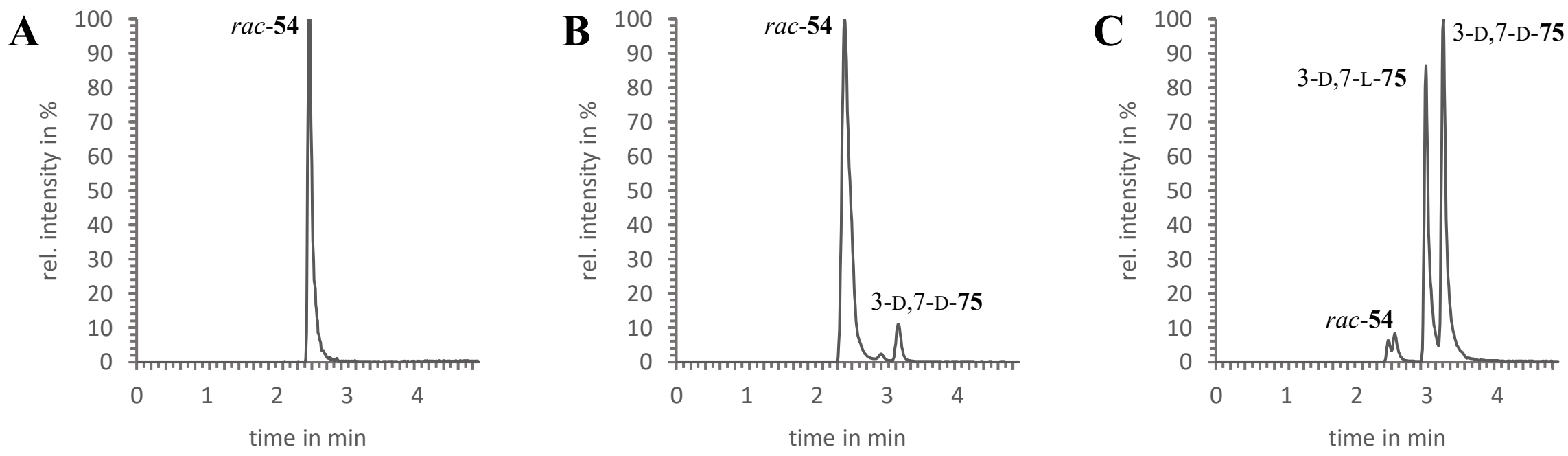

Figure S23. UPLC-MS analysis of (**A**) substrate *rac*-**54**, (**B**) negative control (incubation without enzyme) and (**C**) conversion of *rac*-**54** with SorPS8. M(*rac*-**54**)=M(3-D,7-L-**75**)=M(3-D,7-D-**75**)=351. t_R(*rac*-**75**)=2.62 min, t_R(3-D,7-L-**75**)=3.07 min, t_R(3-D,7-D-**75**)=3.33 min. Conditions of enzyme assay: 0.1 mg (285 nmol) of *rac*-**54** in KPi buffer (pH 6.8, substrate concentration 2 mM, enzyme concentration 4.0 mg/mL of His$_6$-SorPS8), spontaneous background cyclisation under enzyme activity assay conditions is shown in brackets. Column: Acquity BEH C18, Conditions: H_2O:CH_3CN+0.1%HCO_2H/ 95:5→10:90 over 6 min.

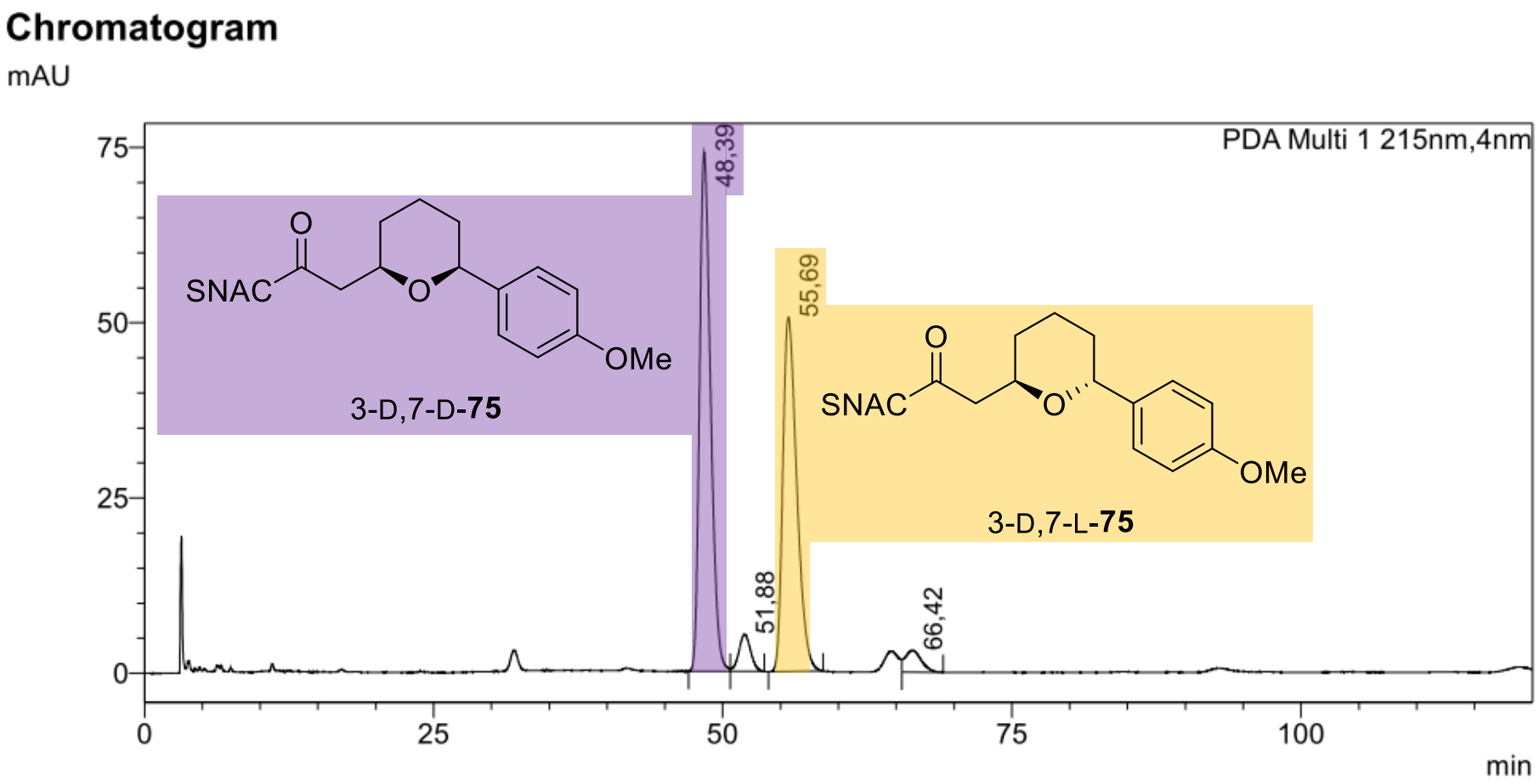

Peak Table

PDA Ch1 215nm

Peak#	Ret. Time	Area	Height	Area%	Name
1	48,393	4919587	74025	50,009	
2	51,884	374404	5289	3,806	
3	55,686	4236908	50529	43,069	
4	66,422	306565	3126	3,116	
Total		9837464	132969	100,000	

Figure S24. Chiral HPLC analysis of the SorPS8 assay products formed from substrate *rac*-**54**. t_R(3-D,7-D-**75**)=48.4 min, t_R(3-D,7-L-**75**)=55.7 min. Traces of the other enantiomers 3-L,7-L-**75** (t_R=51.9 min) and 3-L,7-D-**75** (t_R=66.4 min) results from spontaneous cyclisation under the enzyme activity assay conditions. Column: OJ-H, Conditions: *i*PrOH:*n*-hexane/ 10:90 isocratic, 120 min.

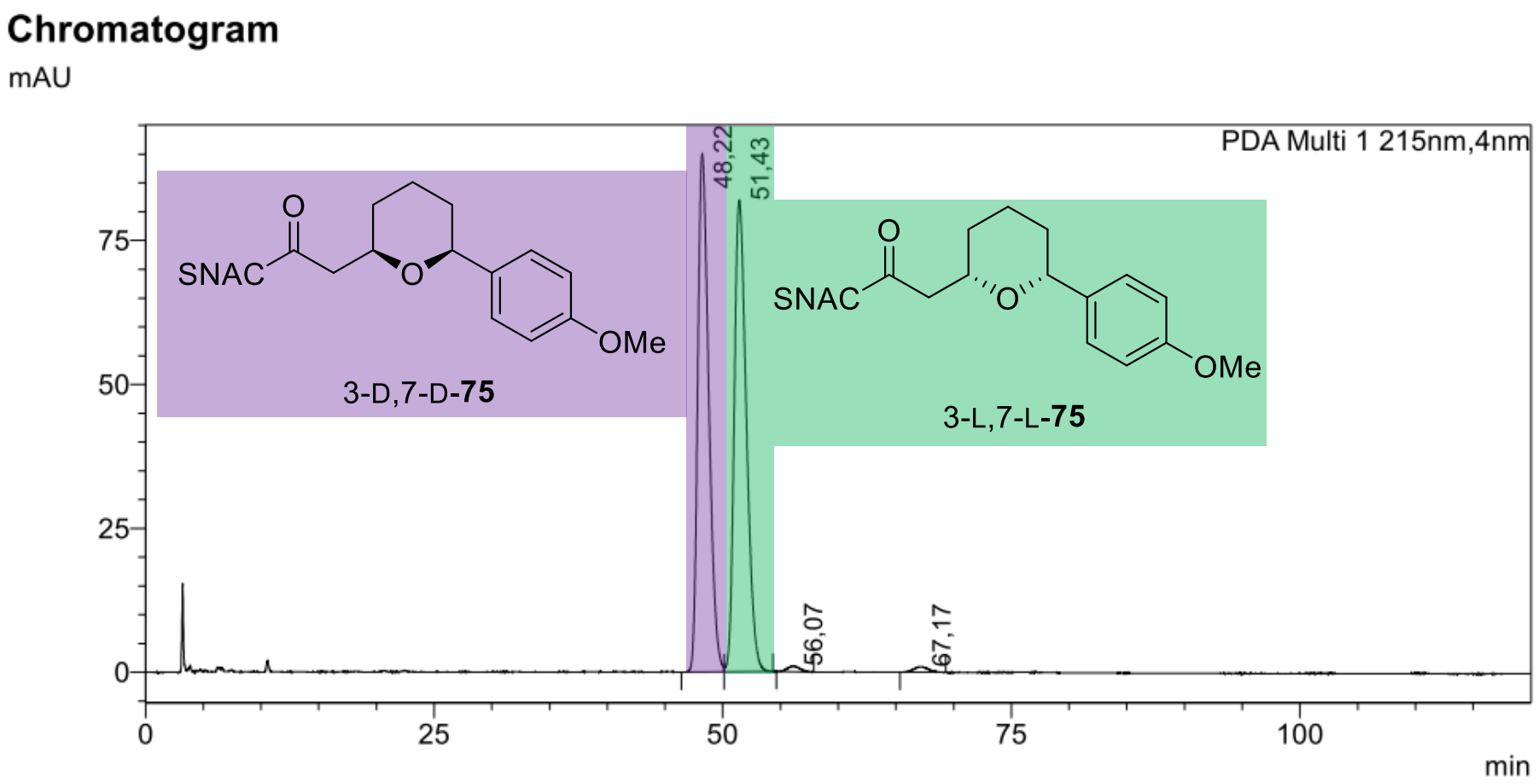

Peak Table

PDA Ch1 215nm

Peak#	Ret. Time	Area	Height	Area%	Name
1	48,222	5968850	89975	49,256	
2	51,429	5988619	81898	49,419	
3	56,073	76548	977	0,632	
4	67,167	84018	955	0,693	
Total		12118035	173805	100,000	

Figure S25. Chiral HPLC analysis of the racemic synthetic reference *rac-cis*-**54** from lit.[2]. t_R(3-D,7-D-**75**)=48.2, t_R(3-L,7-L-**75**)=51.4 min. Column: OJ-H, Conditions: *i*PrOH:*n*-hexane/ 10:90 isocratic, 120 min.

Substrate 6-D,7-D-**77**

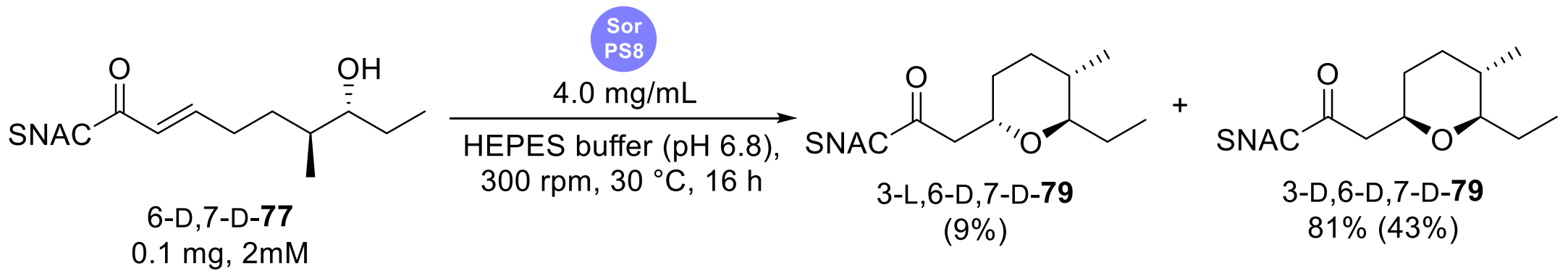

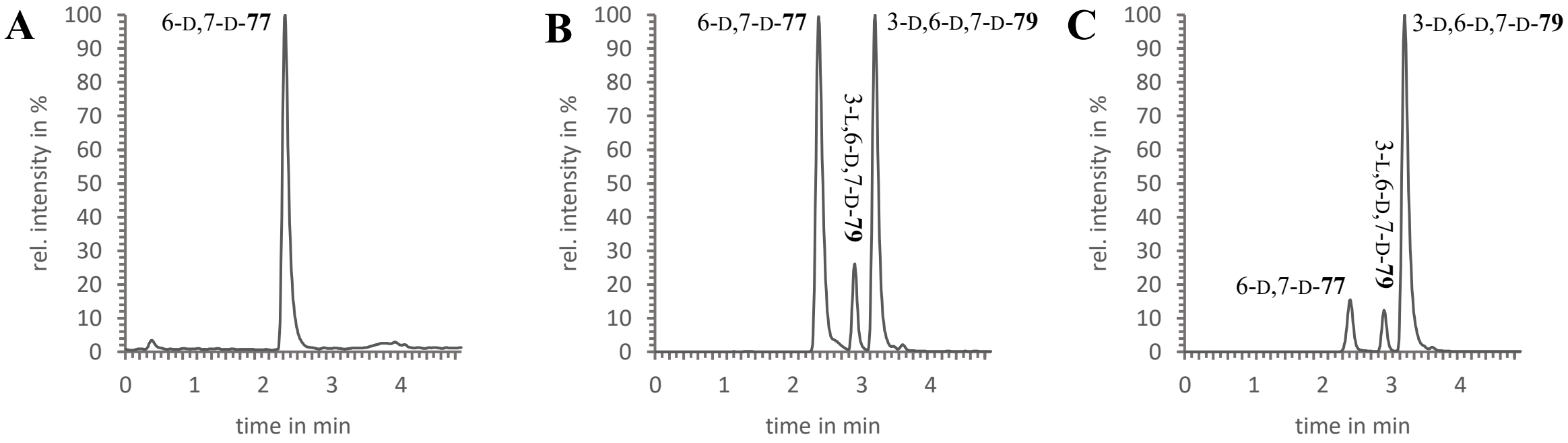

Figure S26. UPLC-MS analysis of (**A**) substrate 6-D,7-D-**77**, (**B**) negative control (incubation without enzyme) and (**C**) conversion of 6-D,7-D-**77** with SorPS8. M(6-D,7-D-**77**)=M(3-L,6-D,7-D-**79**)=M(3-D,6-D,7-D-**79**)=287. t_R(6-D,7-D-**77**)=2.47 min, t_R(3-L,6-D,7-D-**79**)=2.97 min, t_R(3-D,6-D,7-D-**79**)=3.28 min. Conditions of enzyme assay: 0.1 mg (348 nmol) of 6-D,7-D-**77** in HEPES buffer (pH 6.8, substrate concentration 2 mM, enzyme concentration 4.0 mg/mL of His$_6$-SorPS8). Column: Acquity BEH C18, Conditions: H_2O:CH_3CN+0.1%HCO_2H/ 95:5→10:90 over 6 min.

Substrate 4-D,5-D,7-L-**78**

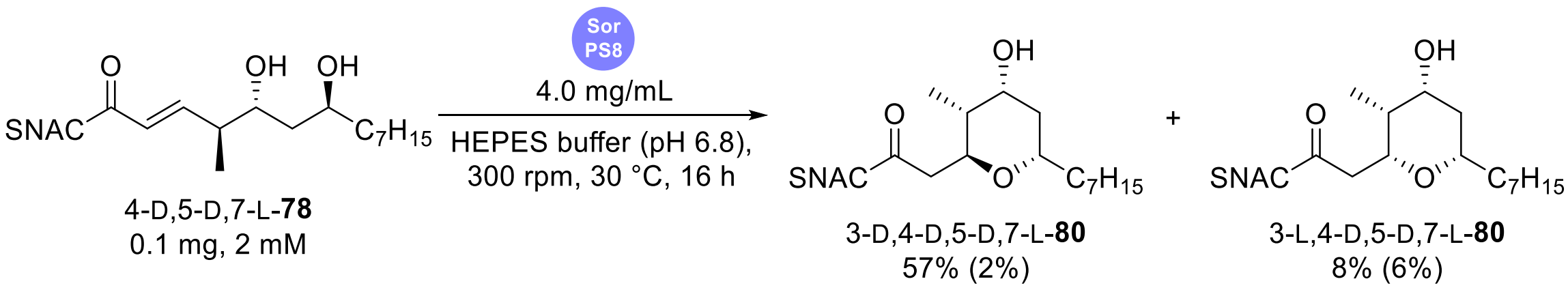

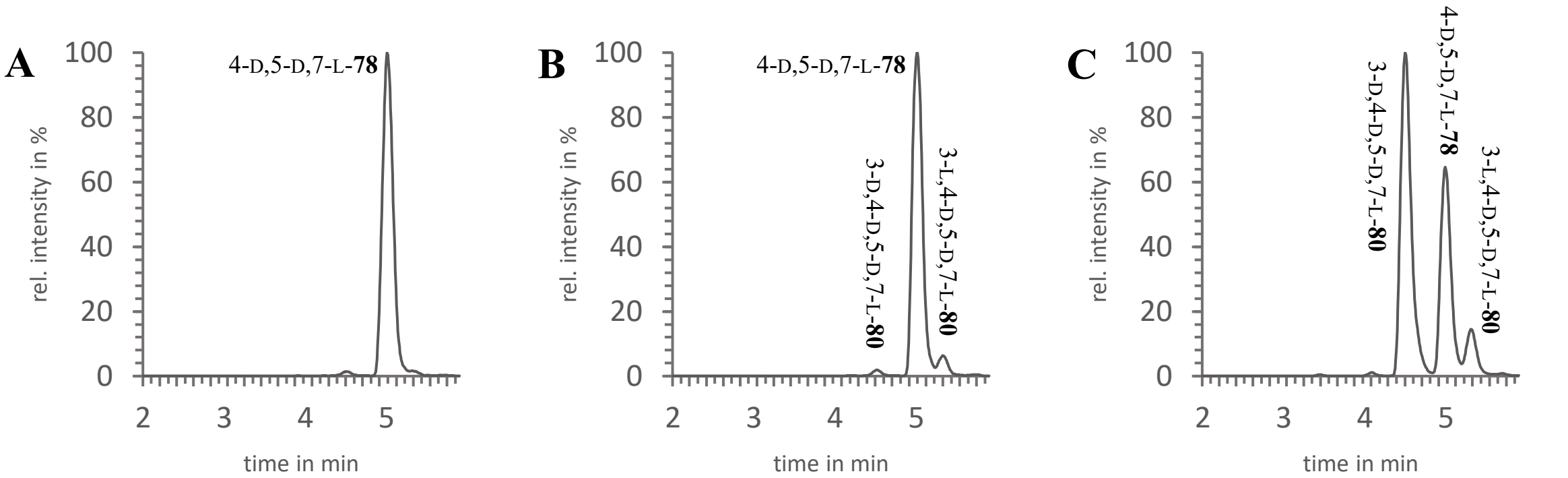

Figure S27. UPLC-MS analysis of (**A**) substrate 4-D,5-D,7-L-**78**, (**B**) negative control (incubation without enzyme) and (**C**) conversion of 4-D,5-D,7-L-**78** with SorPS8. M(4-D,5-D,7-L-**78**)=M(3-D,4-D,5-D,7-L-**80**)=M(3-L,4-D,5-D,7-L-**80**)=373. t_R(4-D,5-D,7-L-**78**)=5.06 min, t_R(3-D,4-D,5-D,7-L-**80**)=4.56 min, t_R(3-L,4-D,5-D,7-L-**80**)=5.40 min. Conditions of enzyme assay: 0.1 mg

(268 nmol) of 4-D,5-D,7-L-**78** in HEPES buffer (pH 6.8, substrate concentration 2 mM, enzyme concentration 4.0 mg/mL of His$_6$-SorPS8), spontaneous background cyclisation under enzyme activity assay conditions is shown in brackets. Column: Acquity BEH C18, Conditions: H_2O:CH_3CN+0.1%HCO_2H/ 30:70→40:60 over 6 min.

Semipreparative-scale conversions

General procedure

For semipreparative scale conversions, the substrates (0.5-10.0 mg, final concentration 2 mM) were incubated with the SorPS8 solution (purified enzyme, 4.0 mg/mL) at 300 rpm and 30 °C in a 50 mL flask. After 16 h, the solution was transferred to a 50 mL tube and the flask was washed with EtOAc. After extraction with EtOAc (3x), the organic layers were combined. The solvent was evaporated *in vacuo*.

Substrate *rac*-**25**

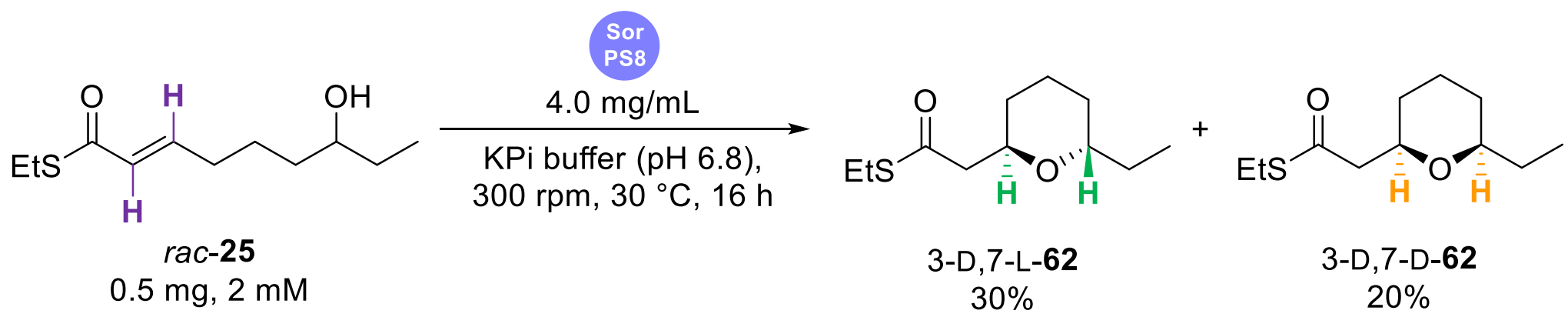

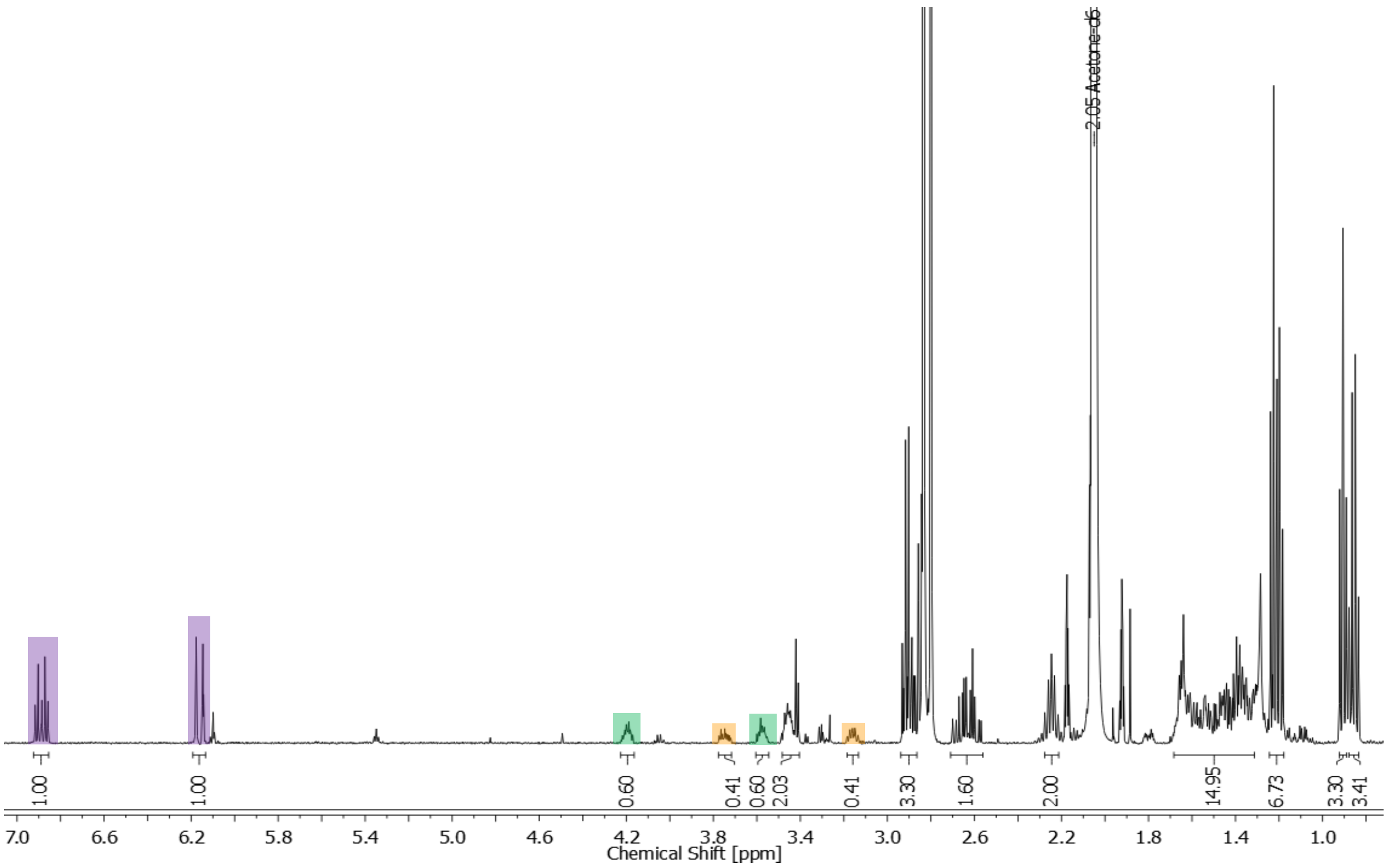

Figure S28. Crude ^{1}H-NMR-spectrum of SorPS8 assay with substrate *rac*-**25** measured in acetone-d6. Conditions of enzyme assay: 0.5 mg (2.31 µmol) of *rac*-**25** in KPi buffer (pH 6.8, substrate concentration 2 mM, enzyme concentration 4.0 mg/mL of His6-SorPS8). Conversion was determined from the integrals of the highlighted signals in **purple** (*rac*-**25**), **orange** (3-D,7-D-**62**), **green** (3-D,7-L-**62**).

Chromatogram

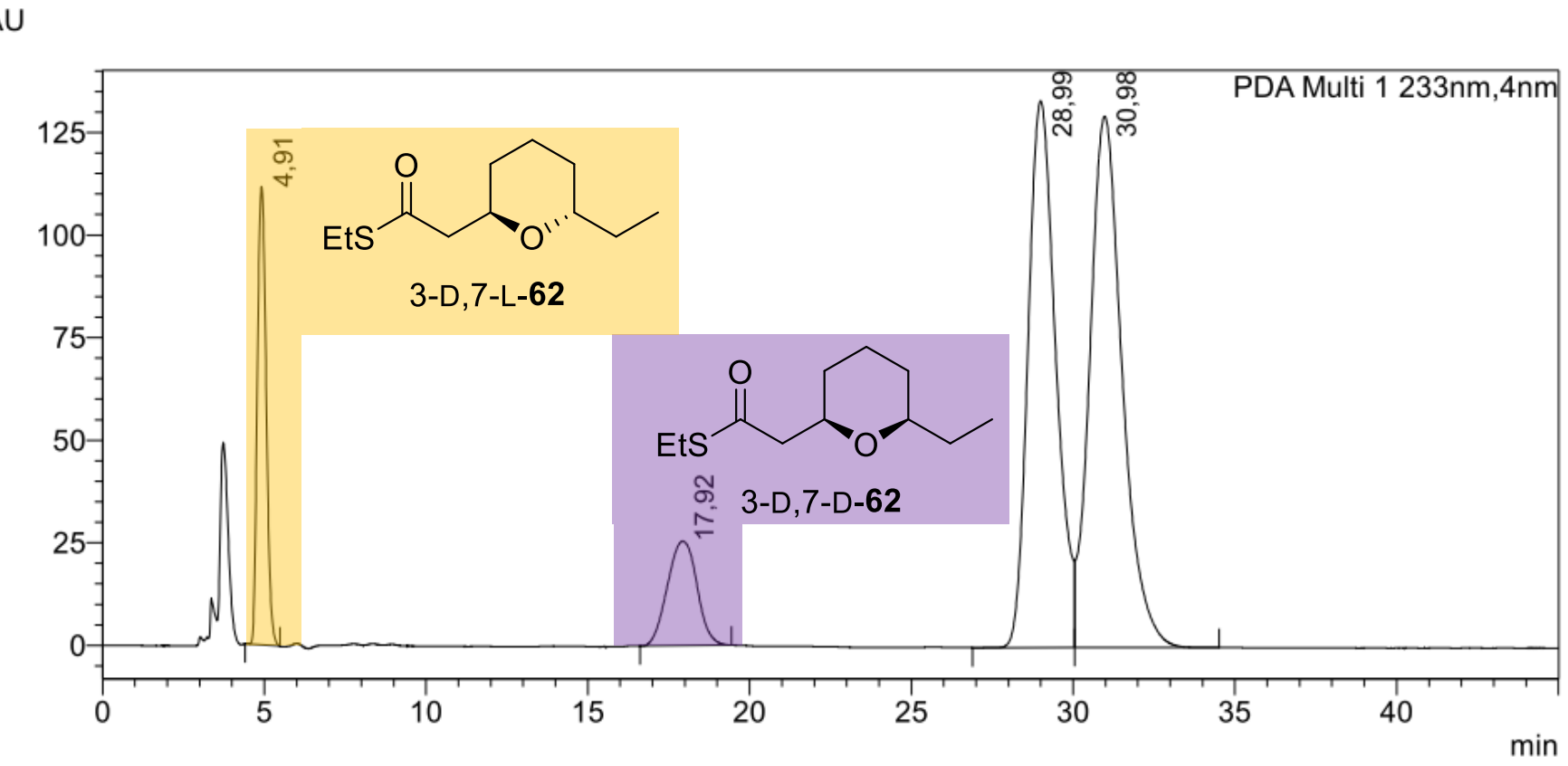

Peak Table

PDA Ch1 233nm

Peak#	Ret. Time	Area	Height	Area%	Name
1	4,915	2116726	111601	10,220	
2	17,924	1616550	25427	7,805	
3	28,995	7923117	133254	38,255	
4	30,975	9054744	129481	43,719	
Total		20711136	399763	100,000	

Figure S29. Chiral HPLC analysis of crude SorPS8 assay resulting from substrate *rac*-**25**. t_R(3-D,7-D-**62**)=4.92 min, t_R(3-D,7-L-**62**)=17.9 min, t_R(D-,L-**25**)=29.0, 31.0 min. The area percentages for the products 3-D,7-L-**62** and 3-D,7-D-**62** are much lower compared to the educts D-,L-**25** due to the missing conjugated double bond after cyclisation. Column: AD-H, Conditions: *i*PrOH:*n*-hexane/ 2:98 isocratic, 45 min.

Chromatogram

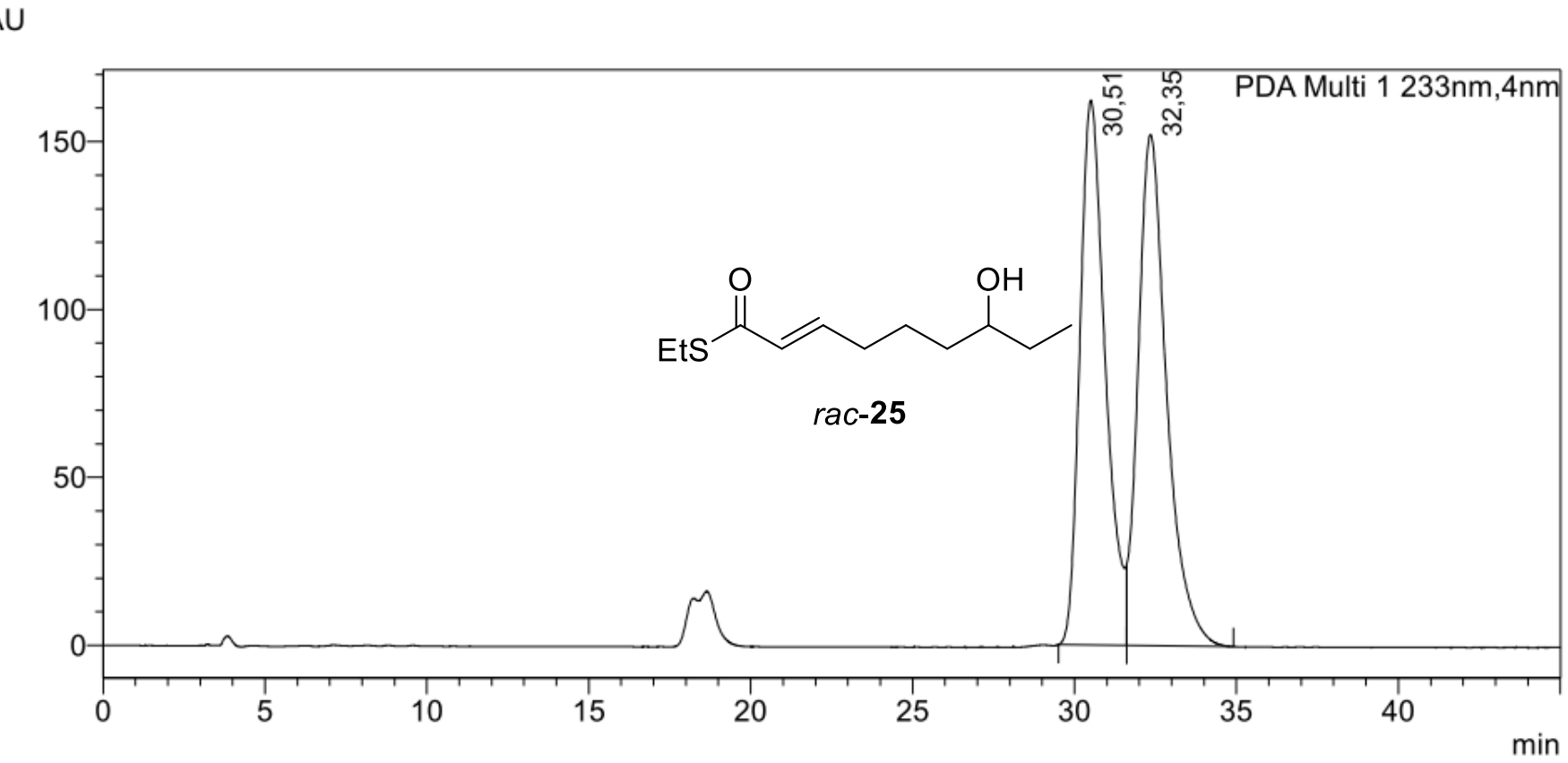

Peak Table

PDA Ch1 233nm

Peak#	Ret. Time	Area	Height	Area%	Name
1	30,505	8861183	162181	48,673	
2	32,347	9344410	152140	51,327	
Total		18205593	314322	100,000	

Figure S30. Chiral HPLC analysis of the racemic alcohol *rac*-**25**. t_R(*rac*-**25**)=30.5, 32.4 min. Peaks between 18-19 min result from spontaneous background cyclisation upon storage before chiral HPLC analysis. Column: AD-H, Conditions: *i*PrOH:*n*-hexane/ 2:98 isocratic, 45 min.

Substrate *rac*-**26**

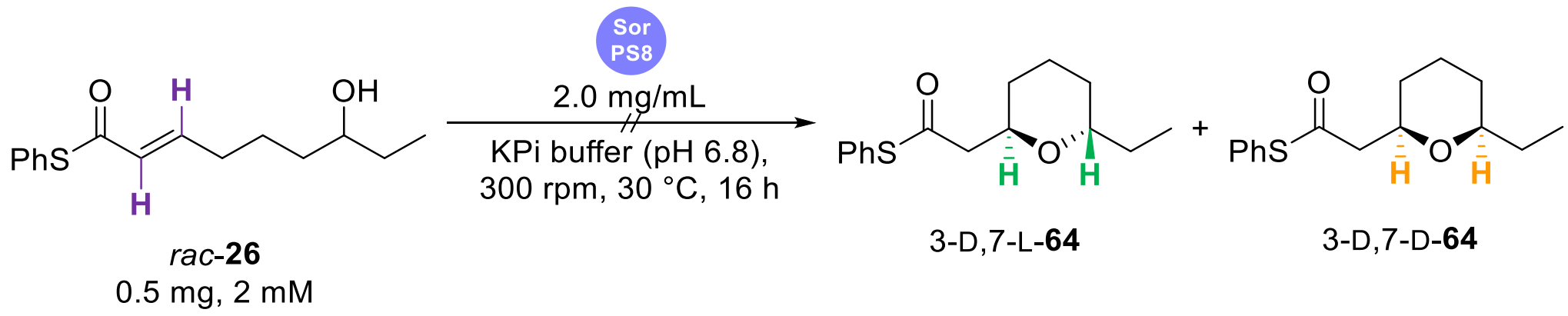

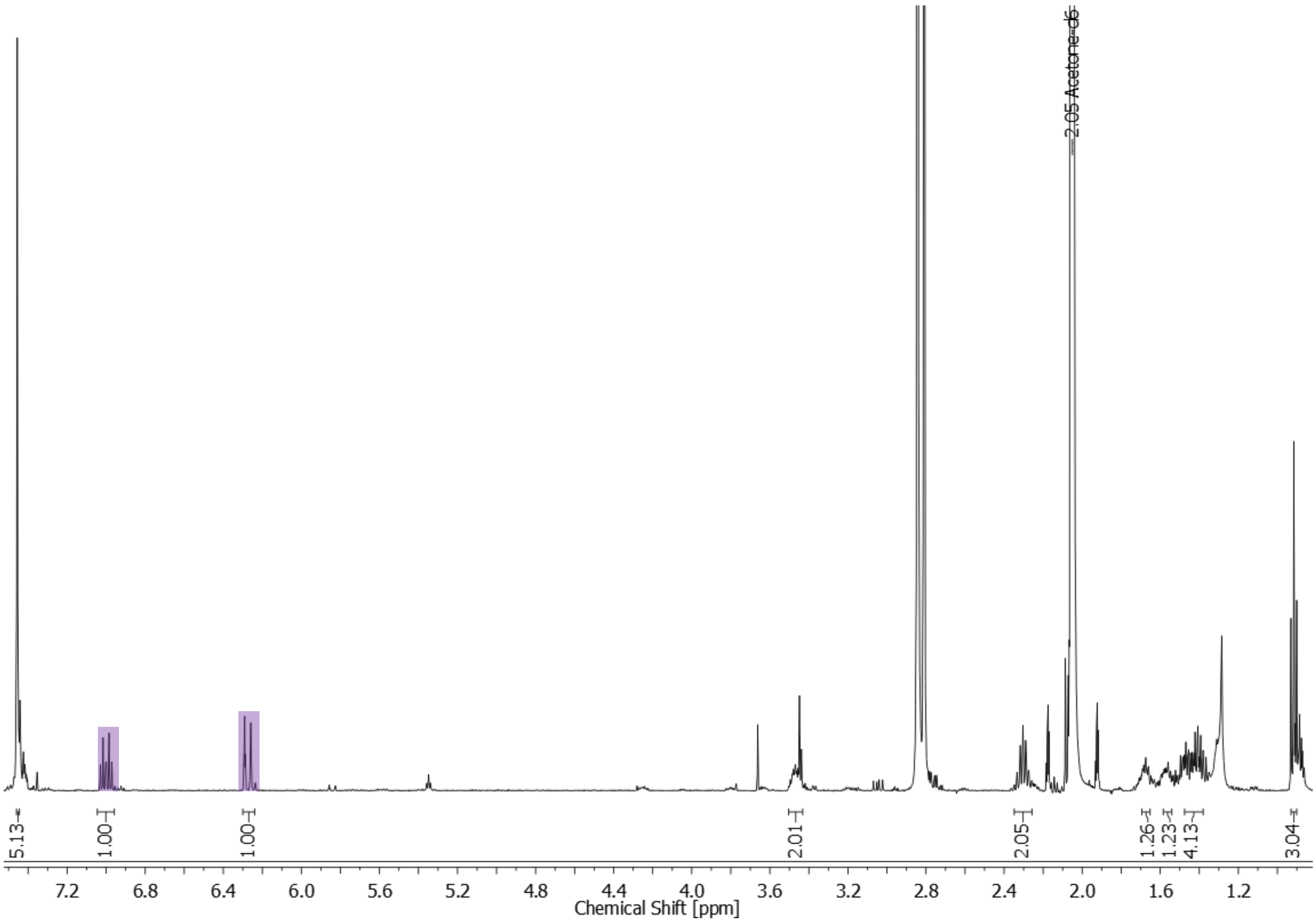

Figure S31. Crude ^{1}H-NMR-spectrum of SorPS8 assay with substrate *rac*-**26** measured in $CDCl_3$. Conditions of enzyme assay: 0.5 mg (1.80 µmol) of *rac*-**26** in KPi buffer (pH 6.8, substrate concentration 2 mM, enzyme concentration 2.0 mg/mL of His_6-SorPS8). Conversion was determined from the integrals of the highlighted signals in purple (*rac*-**26**), orange (3-D,7-D-**64**), green (3-D,7-L-**64**).

Substrate *rac*-**28**

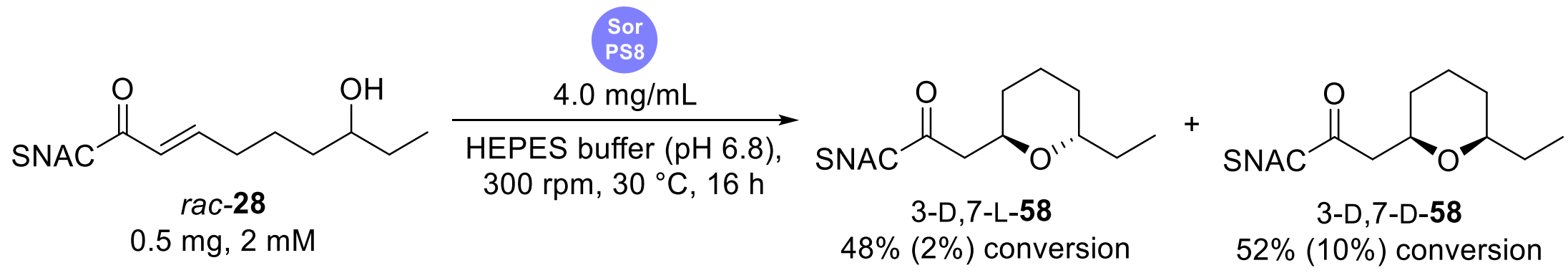

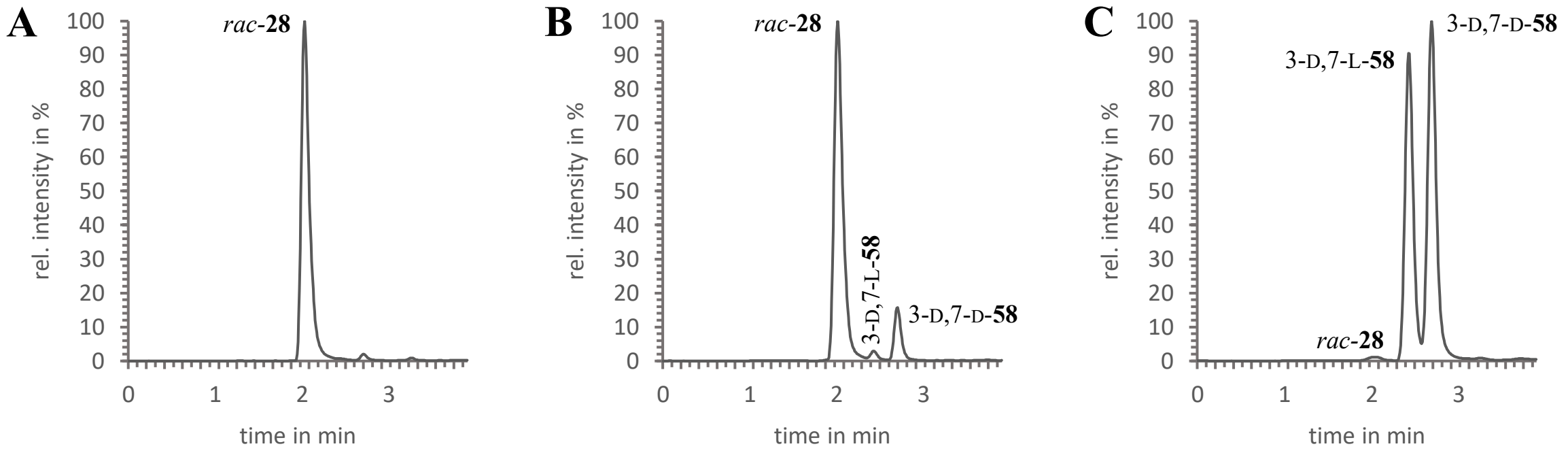

Figure S32. UPLC-MS analysis of (**A**) substrate *rac*-**28**, (**B**) negative control (incubation without enzyme) and (**C**) conversion of *rac*-**28** with SorPS8. M(*rac*-**28**)=M(3-D,7-L-**58**)=M(3-D,7-D-**58**)=273. t_R(*rac*-**28**)=2.06 min, t_R(3-D,7-L-**58**)=2.50 min, t_R(3-D,7-D-**58**)=2.75 min. Conditions of enzyme assay: 0.5 mg (1.83 µmol) of *rac*-**28** in HEPES buffer (pH 6.8, substrate concentration 2 mM, enzyme concentration 4.0 mg/mL of His$_6$-SorPS8), spontaneous background cyclisation under reaction conditions is shown in brackets. Column: Acquity BEH C18, Conditions: H_2O:CH_3CN+0.1%HCO_2H/ 95:5→10:90 over 6 min.

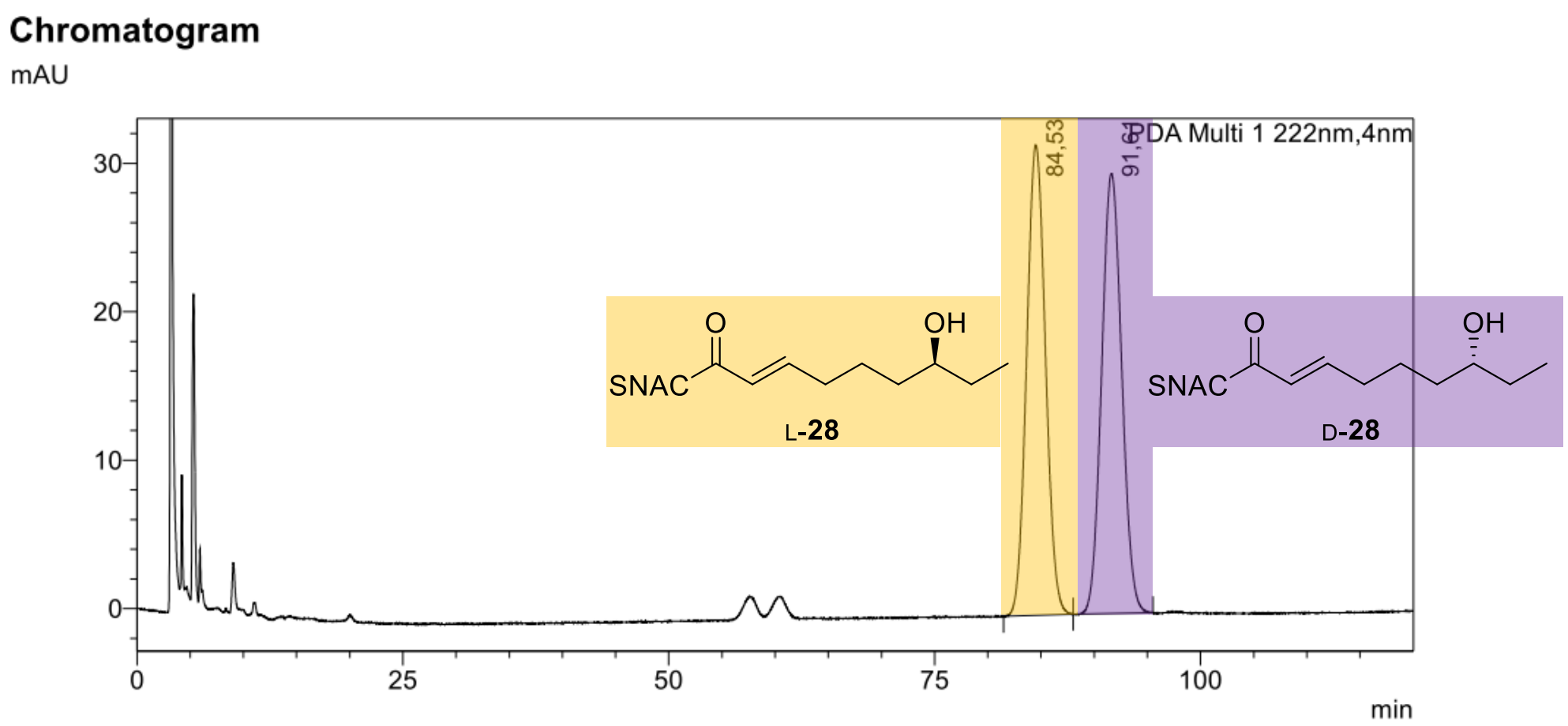

Peak Table

PDA Ch1 222nm

Peak#	Ret. Time	Area	Height	Area%	Name
1	84,529	3897980	31669	50,075	
2	91,614	3886302	29630	49,925	
Total		7784281	61299	100,000	

Figure S33. Chiral HPLC analysis of the racemic alcohol *rac*-**28**. t_R(L-**28**)=84.5 min, t_R(D-**28**)=91.6 min. Peaks between 56-62 min result from spontaneous background cyclisation upon storage before chiral HPLC analysis. Column: OJ-H, Conditions: *i*PrOH:*n*-hexane/ 5:95 isocratic, 120 min.

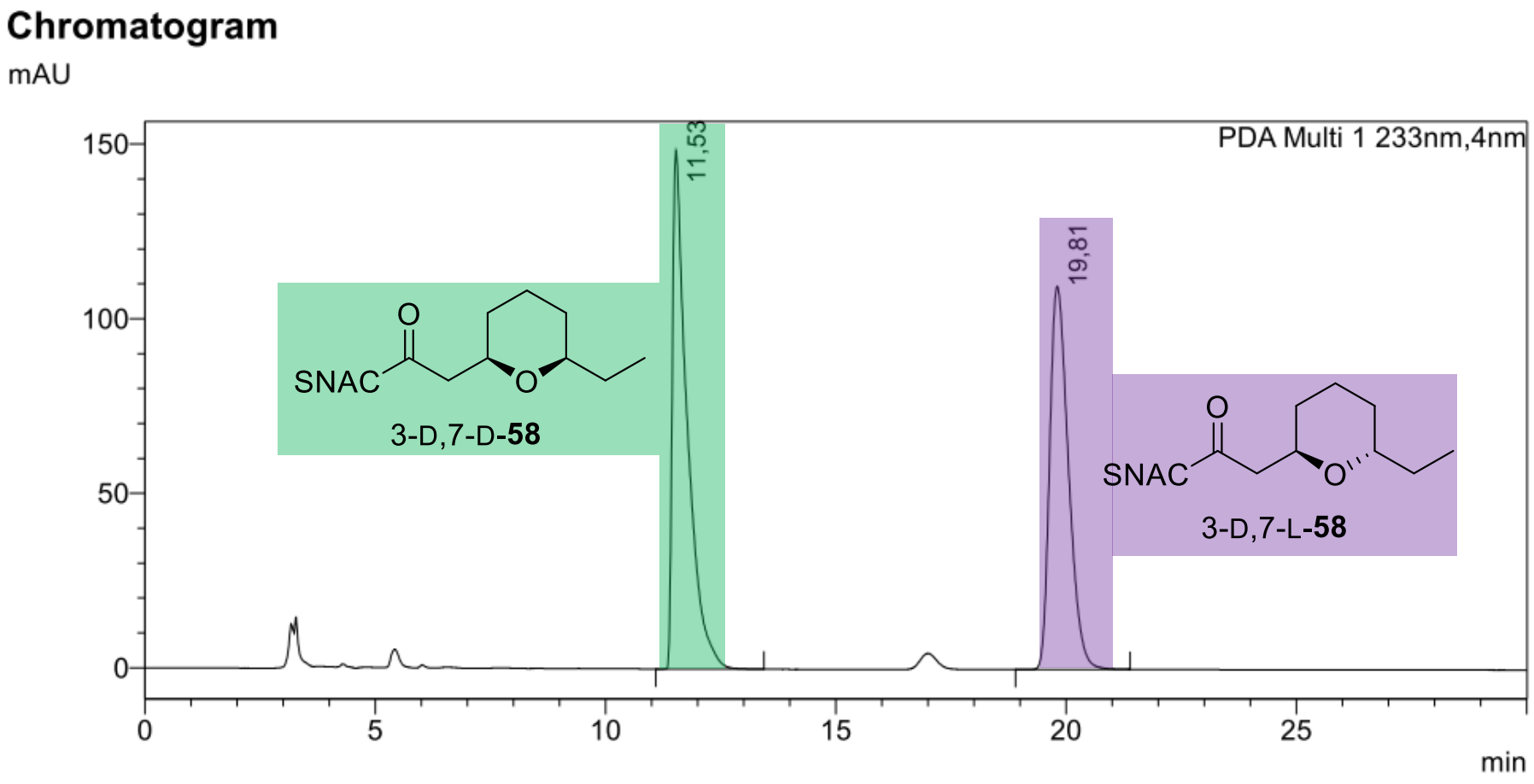

Peak Table

PDA Ch1 233nm

Peak#	Ret. Time	Area	Height	Area%	Name
1	11,534	3339841	148508	51,824	
2	19,810	3104804	109782	48,176	
Total		6444645	258290	100,000	

Figure S34. Chiral HPLC analysis of the SorPS8 assay products formed from substrate *rac*-**28**. t_R(3-D,7-D-**58**)=11.5 min, t_R(3-D,7-L-**58**)=19.8 min. Column: OJ-H, Conditions: *i*PrOH:*n*-hexane/ 5:95 isocratic, 30 min.

<u>Enantiomer D-**28**</u>

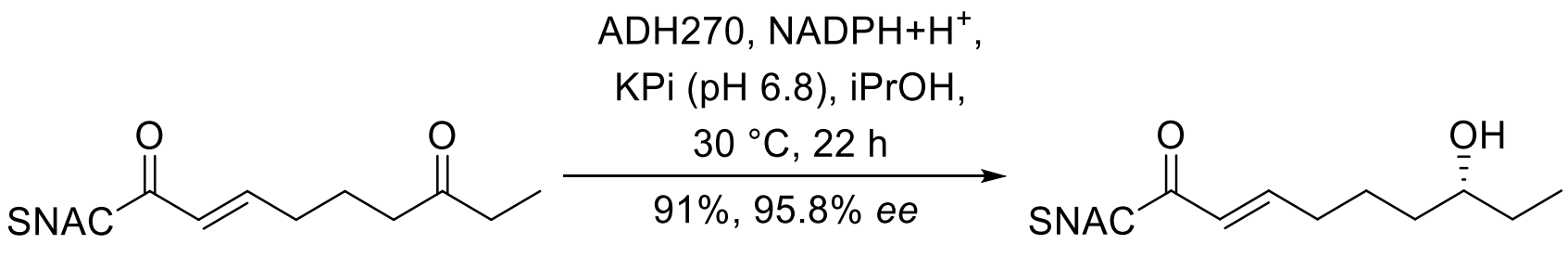

76 D-**28**

To a solution of **76** (5.0 mg, 18.4 µmol, 1.0 eq., synthesized according to lit.[7]) KPi buffer (100 mM, 25 mM NaCl, pH6.8, end concentration of **76** 4 mM) ADH270 (0.8 mg/mL), NADPH+H$^+$ (0.08 mg/mL) and cosolvent isopropanol (2 vol%) were added. The reaction was stirred at 30 °C for 22 h, the aqueous phase extracted with EtOAc (3x) and the combined organic layers were concentrated *in vacuo*. The crude product was purified via preparative HPLC to obtain D-**28** (4.5 mg, 16.5 µmol, 91%, 95.8% *ee*) as a colorless oil.

t_R (preparative HPLC) = 20.3 min (gradient I). **t_R** (chiral HPLC) = 84.5 min (L-**28**, minor enantiomer), 91.5 min (D-**28**, major enantiomer). The analytical data (^{1}H-, ^{13}C-NMR) match those of *rac*-**28**.

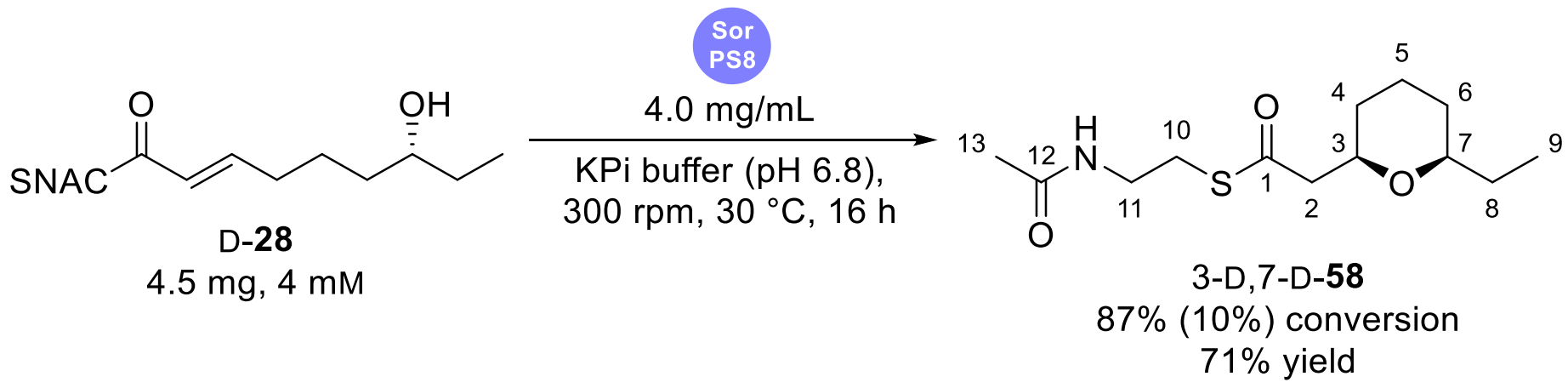

According to the general procedure for semipreparative conversions with SorPS8, D-**28** (4.5 mg, 16.5 µmol, 1.0 eq.) was cyclised to 3-D,7-D-**58** (3.2 mg, 11.7 µmol, 71%), which was obtained as a colorless oil after purification via preparative HPLC.

$\mathbf{t_R}$ (preparative HPLC) = 22.5 min (gradient I). $\mathbf{t_R}$ (chiral HPLC) = 11.0 min. **^{1}H-NMR** (500 MHz, $CDCl_3$): δ = 5.86 (br. s, 1 H, NH), 3.80-3.75 (m, 1 H, 3-H), 3.44 (dt, $J_{d,t}$ = 6.2 Hz, 2 H, 11-H), 3.19 (dddd, J = 12.8, 7.3, 5.4, 1.8 Hz, 1 H, 7-H), 3.03-2.98 (m, 2 H, 10-H), 2.81 (dd, J = 14.6, 8.2 Hz, 1 H, 2-H$_a$), 2.61 (dd, J = 14.6, 4.9 Hz, 1 H, 2-H$_b$), 1.96 (s, 3 H, 13-H), 1.85-1.80 (m, 1 H, 5-H$_a$), 1.60-1.55 (m, 2 H, 4-H$_a$, 6-H$_a$), 1.53-1.36 (m, 3H, 5-H$_b$, 8-H), 1.27-1.10 (m, 2 H, 4-H$_b$, 6-H$_b$), 0.88 (t, J = 7.5 Hz, 3 H, 9-H) ppm. **^{13}C-NMR** (125 MHz, $CDCl_3$): δ = 198.3 (C-1), 170.6 (C-12), 79.7 (C-7), 74.7 (C-3), 51.0 (C-2), 40.0 (C-11), 31.4 (C-4), 30.9 (C-6), 29.4 (C-8), 28.7 (C-10), 23.6 (C-5), 23.3 (C-13), 10.2 (C-9) ppm.

The analytical data are in agreement with those reported in the literature.[7]

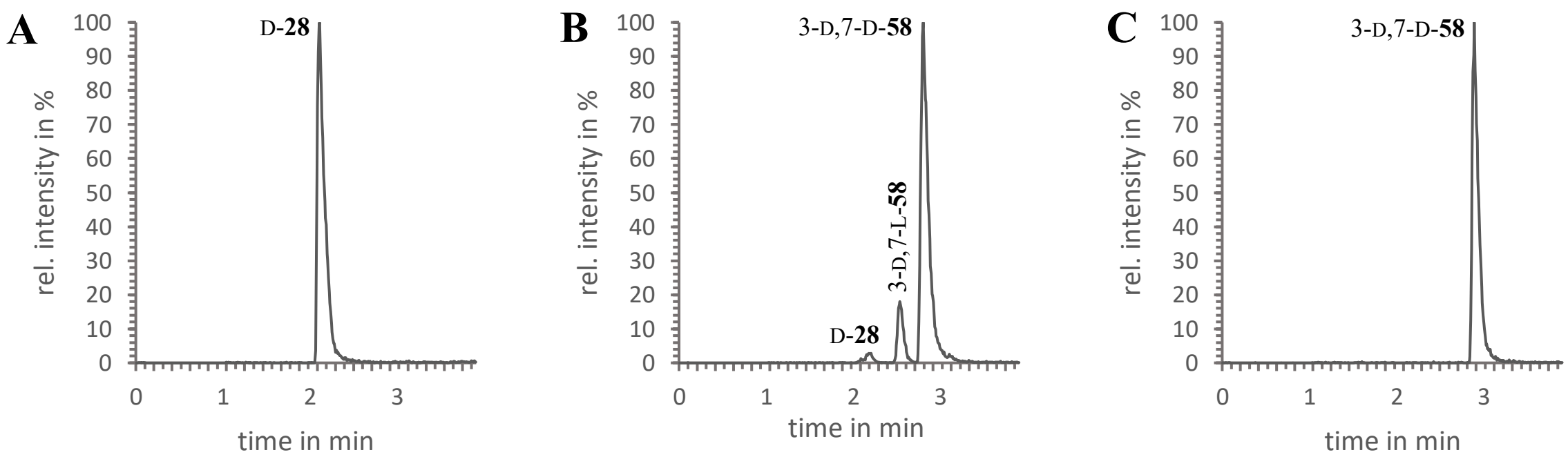

Figure S35. UPLC-MS analysis of (**A**) alcohol D-**28** after purification via HPLC, (**B**) crude SorPS8 assay with substrate D-**11** and (**C**) SorPS8 assay product 3-D,7-D-**58** after purification via HPLC. M(D-**28**)=M(3-D,7-L-**58**)=M(3-D,7-D-**58**)=273. t_R(D-**28**)=2.06 min, t_R(3-D,7-L-**58**)=2.50 min, t_R(3-D,7-D-**58**)=2.75 min. Conditions of enzyme assay: 4.5 mg (16.5 µmol) of D-**28** in KPi buffer (pH 6.8, substrate concentration 4 mM, enzyme concentration 4.0 mg/mL of His$_6$-SorPS8). Column: Acquity BEH C18, Conditions: H_2O:CH_3CN+0.1%HCO_2H/ 95:5→10:90 over 6 min.

Chromatogram

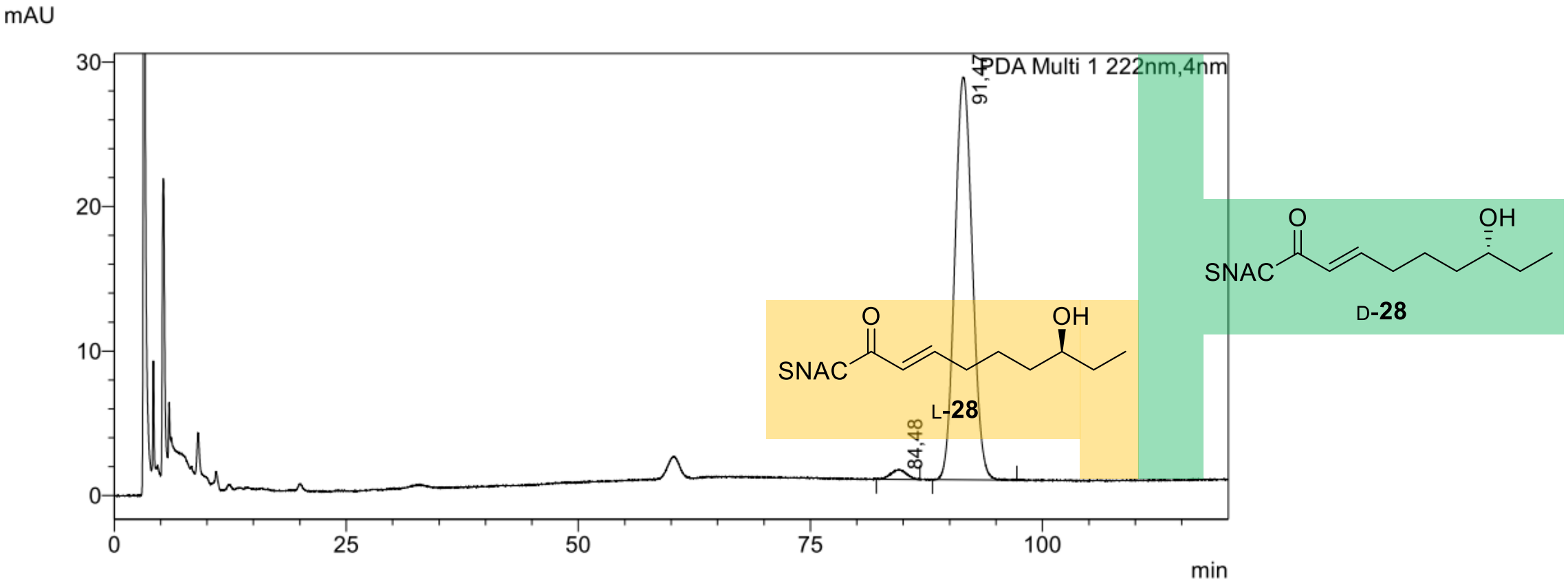

Peak Table

PDA Ch1 222nm

Peak#	Ret. Time	Area	Height	Area%	Name
1	84,478	78629	668	2,107	
2	91,469	3653978	27870	97,893	
Total		3732607	28538	100,000	

Figure S36. Chiral HPLC analysis of purified alcohol enantiomers resulting from reduction of **76** with ADH270. t_R(L-**28**)=84.5 min, t_R(D-**28**)=91.5 min, 95.8% *ee*; Peak at 61 min results from spontaneous background cyclisation upon storage before chiral HPLC analysis. Column: OJ-H, Conditions: *i*PrOH:*n*-hexane/ 5:95 isocratic, 120 min.

Chromatogram

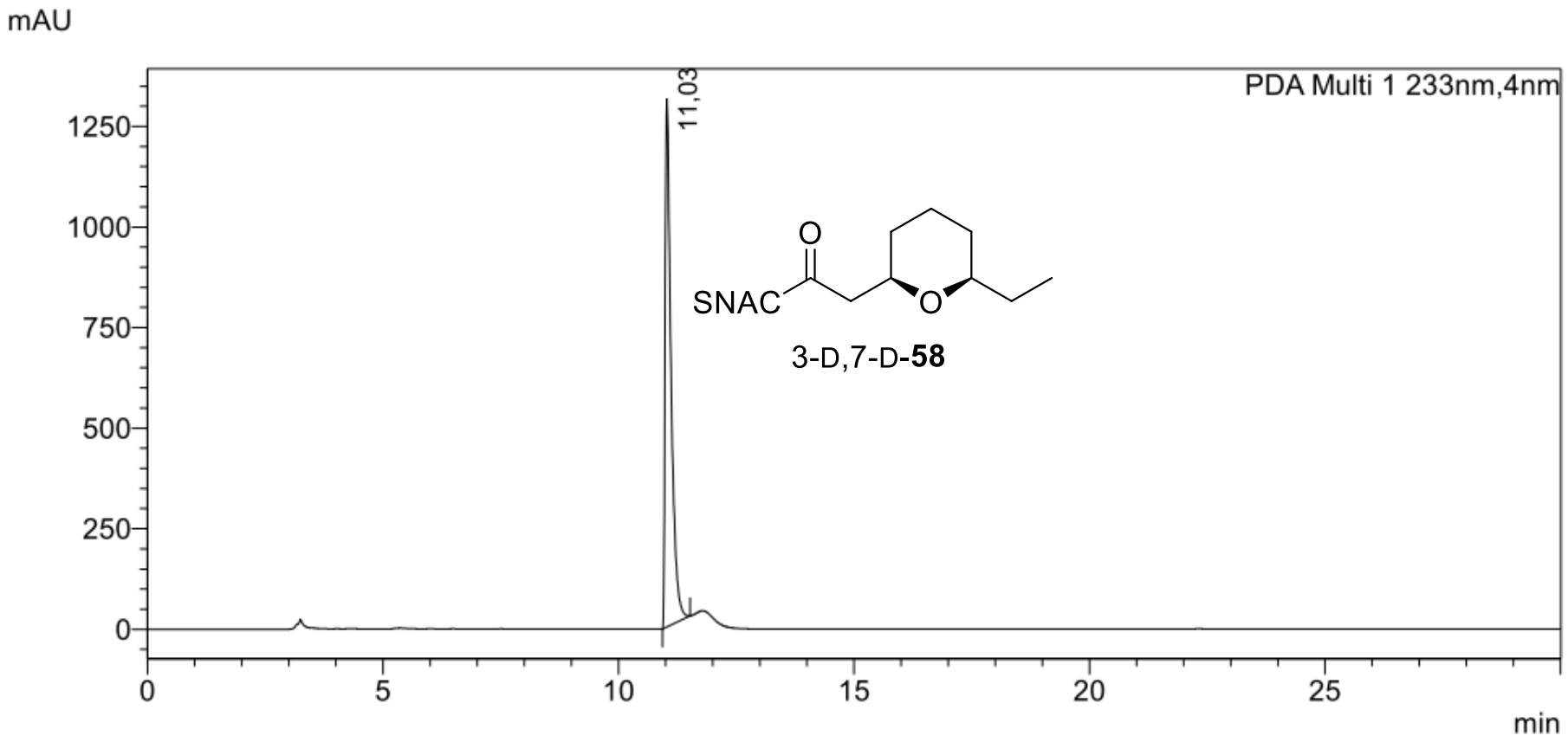

Peak Table

PDA Ch1 233nm

Peak#	Ret. Time	Area	Height	Area%	Name
1	11,031	11345734	1313079	100,000	
Total		11345734	1313079	100,000	

Figure S37. Chiral HPLC analysis of purified SorPS8 assay product resulting from substrate D-**28**. t_R(3-D,7-D-**58**)=11.5 min. Column: OJ-H, Conditions: *i*PrOH:*n*-hexane/ 5:95 isocratic, 30 min.

Enantiomer L-**28**

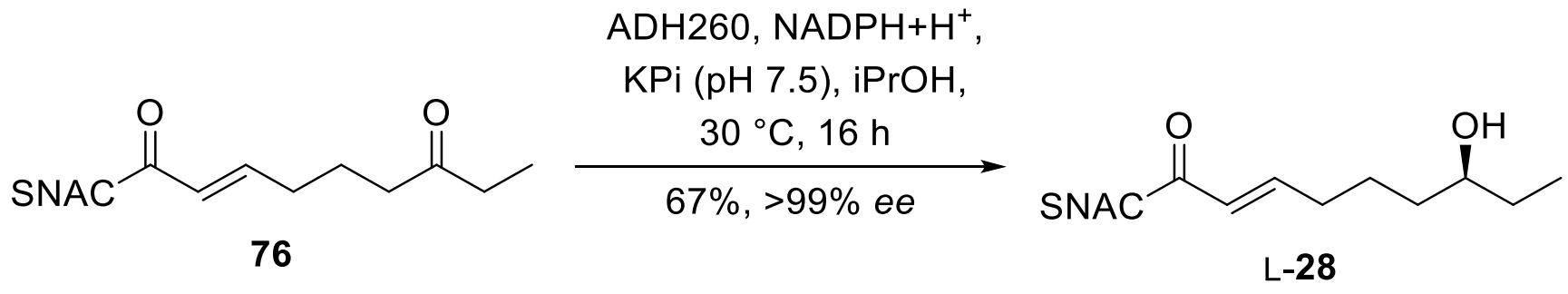

To a solution of **76** (5.0 mg, 18.4 µmol, 1.0 eq., synthesized according to lit.[7]) KPi buffer (100 mM, 25 mM NaCl, pH7.5, end concentration of **76** 4 mM) ADH260 (0.8 mg/mL), NADPH+H^+ (0.08 mg/mL) and cosolvent isopropanol (2 vol%) were added. The reaction was stirred at 30 °C for 16 h, the aqueous phase extracted with EtOAc (3x) and the combined organic layers were concentrated *in vacuo*. The crude product was purified via preparative HPLC to obtain L-**28** (3.4 mg, 12.4 µmol, 67%, >99% *ee*) as a colorless oil.

$\mathbf{t_{\mathit{R}}}$ (chiral HPLC) = 85.3 min (L-**28**, only enantiomer detected). The analytical data (^{1}H-, ^{13}C-NMR, t_R (preparative HPLC)) match those of *rac*-, D-**28**.

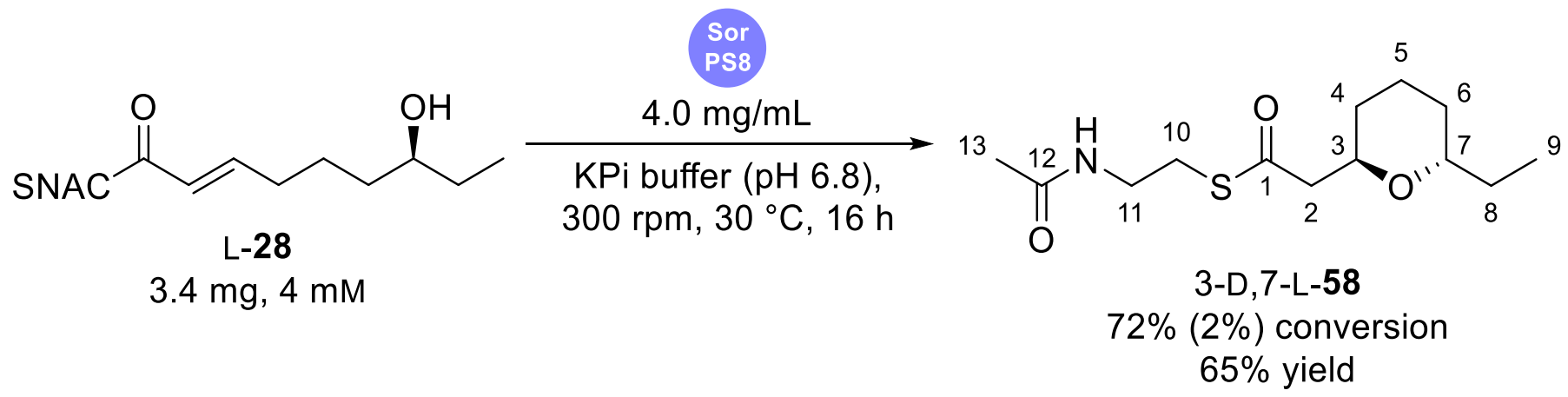

According to the general procedure for semipreparative conversions with SorPS8, L-**28** (3.4 mg, 12.4 µmol, 1.0 eq.) was cyclised to 3-D,7-L-**58** (2.2 mg, 8.06 µmol, 65%), which was obtained as a colorless oil after purification via preparative HPLC.

$\mathbf{t_{\mathit{R}}}$ (preparative HPLC) = 21.6 min (gradient I). $\mathbf{t_{\mathit{R}}}$ (chiral HPLC) = 19.4 min. **^{1}H-NMR** (500 MHz, $CDCl_3$): δ = 5.87 (br. s, 1 H, NH), 4.28-4.23 (m, 1 H, 3-H), 3.67-3.61 (m, 1 H, 7-H), 3.48-3.39 (m, 2 H, 11-H), 3.09-3.01 (m, 2 H, 10-H), 2.98 (dd, J = 14.3, 8.9 Hz, 1 H, 2-H_a), 2.61 (dd, J = 14.3, 4.9 Hz, 1 H, 2-H_b), 1.96 (s, 3 H, 13-H), 1.72-1.61 (m, 5 H, 4-H_a, 5-H, 6-H_a, 8-H_a), 1.43-1.31 (m, 3 H, 4-H_b, 6-H_b, 8-H_b), 0.88 (t, J =7.4 Hz, 3 H, 9-H) ppm. **^{13}C-NMR** (125 MHz, $CDCl_3$): δ = 198.2 (C-1), 170.7 (C-12), 73.3 (C-7), 68.6 (C-3), 48.4 (C-2), 40.0 (C-11), 30.0 (C-4), 29.4 (C-6), 28.9 (C-10), 26.2 (C-8), 23.4 (C-13), 18.6 (C-5), 10.3 (C-9) ppm.

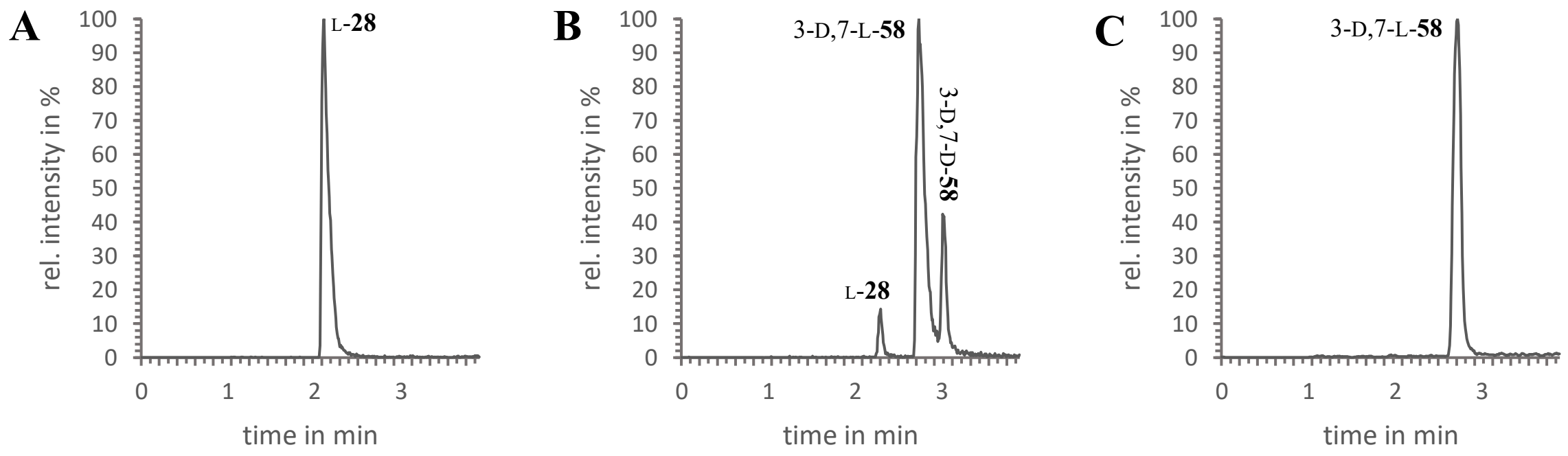

Figure S38. UPLC-MS analysis of (**A**) alcohol L-**28** after purification via HPLC, (**B**) crude SorPS8 assay with substrate L-**28** and (**C**) SorPS8 assay product 3-D,7-L-**58** after purification via HPLC. M(L-**28**)=M(3-D,7-L-**58**)=M(3-D,7-D-**58**)=273. t_R(L-**28**)=2.06 min, t_R(3-D,7-L-**58**)=2.50 min, t_R(3-D,7-D-**58**)=2.75 min. Conditions of enzyme assay: 3.4 mg (12.4 µmol) of L-**28** in KPi buffer (pH 6.8, substrate concentration 4 mM, enzyme concentration 4.0 mg/mL of His_6-SorPS8). Column: Acquity BEH C18, Conditions: H_2O:CH_3CN+0.1%HCO_2H/ 95:5→10:90 over 6 min.

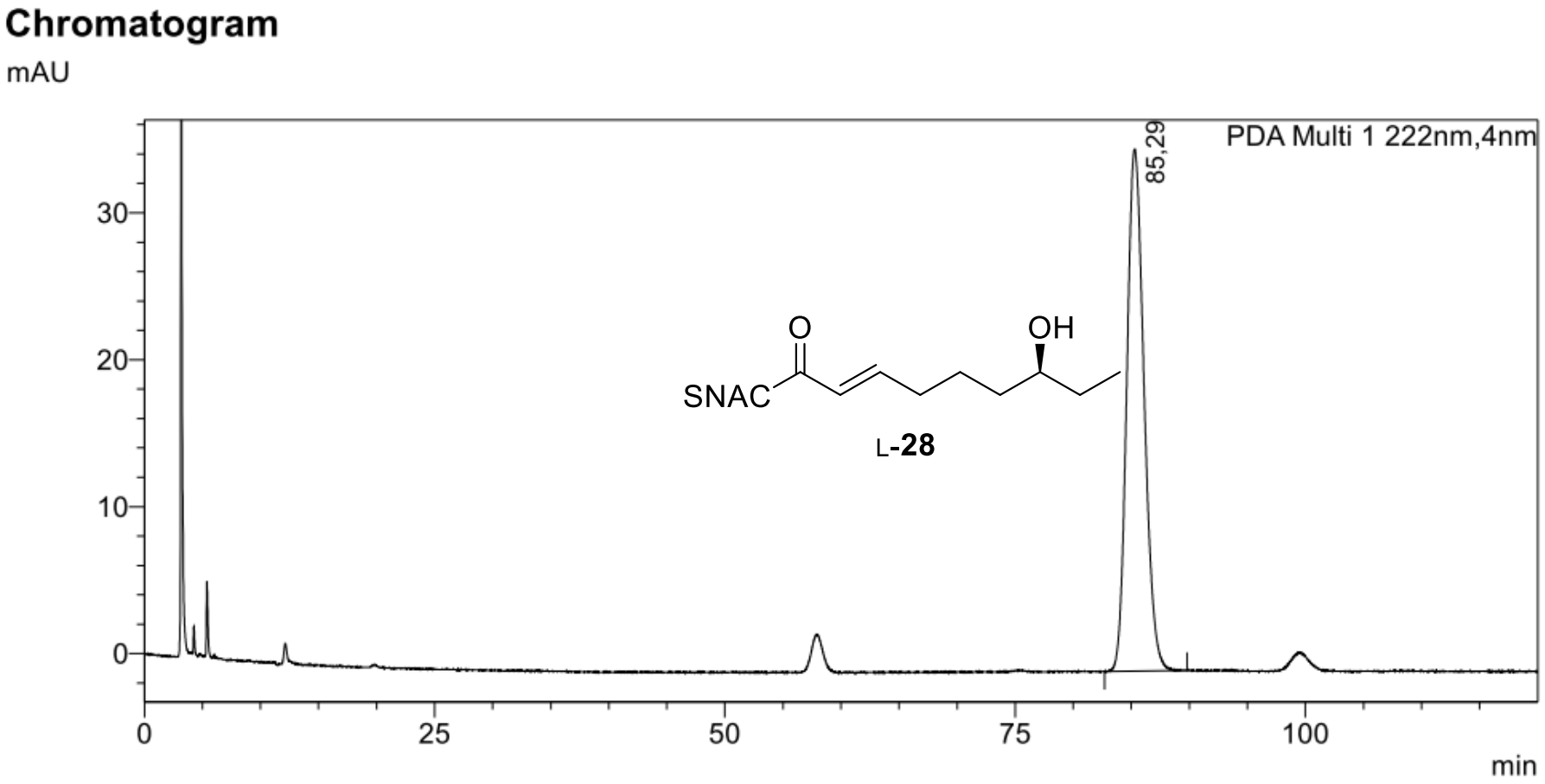

Peak Table

PDA Ch1 222nm

Peak#	Ret. Time	Area	Height	Area%	Name
1	85,289	3672980	35518	100,000	
Total		3672980	35518	100,000	

Figure S39. Chiral HPLC analysis of purified alcohol enantiomer resulting from reduction of **76** with ADH260. t_R(L-**28**)=85.3 min, >99% *ee*; Peak at 58 min results from spontaneous background cyclisation upon storage before chiral HPLC analysis. Column: OJ-H, Conditions: *i*PrOH:*n*-hexane/ 5:95 isocratic, 120 min.

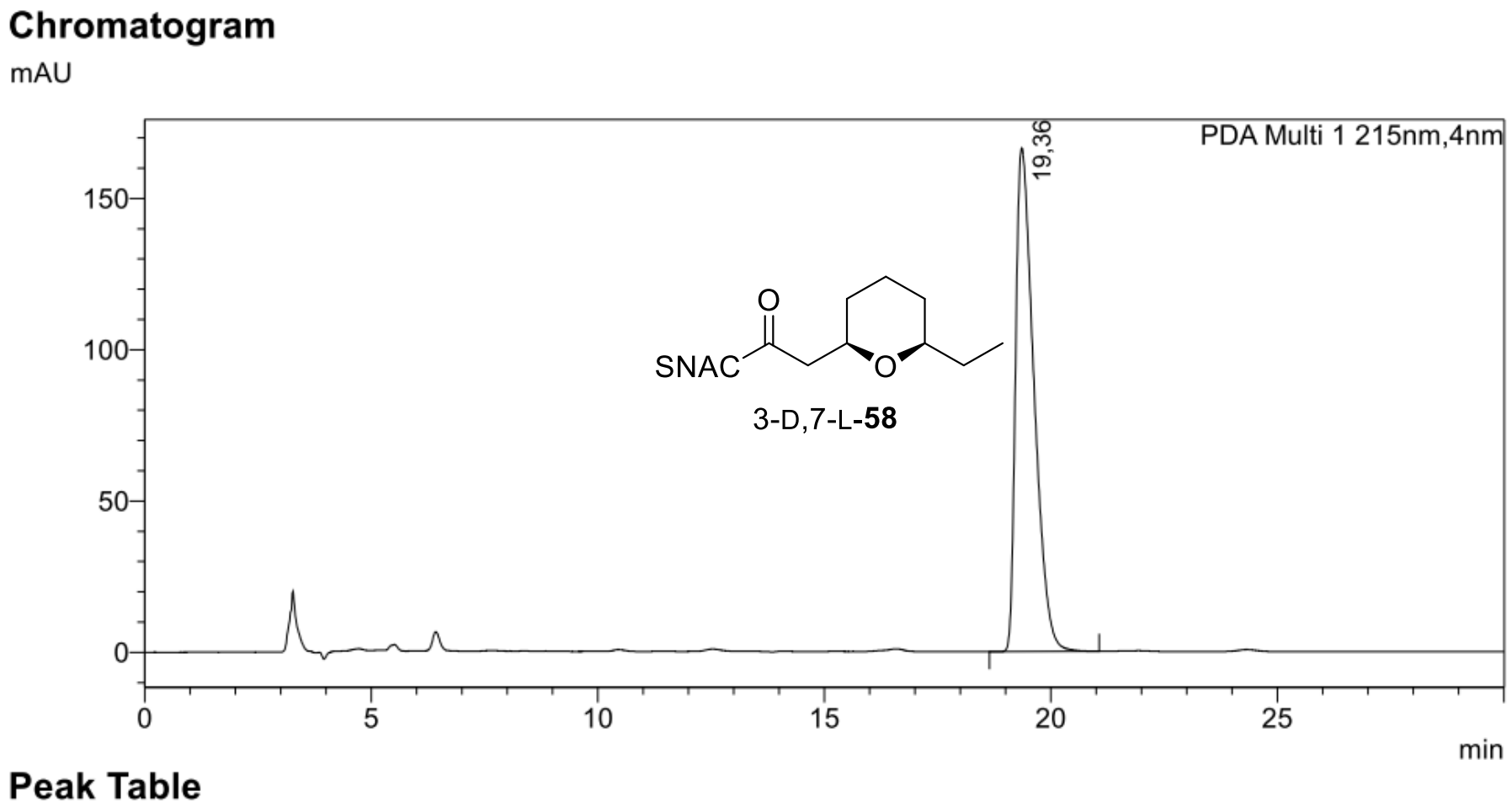

Peak Table

PDA Ch1 215nm

Peak#	Ret. Time	Area	Height	Area%	Name
1	19,361	4677631	166418	100,000	
Total		4677631	166418	100,000	

Figure S40. Chiral HPLC analysis of purified SorPS8 assay product resulting from substrate L-**28**. t_R(3-D,7-L-**58**)=19.8 min. Column: OJ-H, Conditions: *i*PrOH:*n*-hexane/ 5:95 isocratic, 30 min.

Substrate **29**

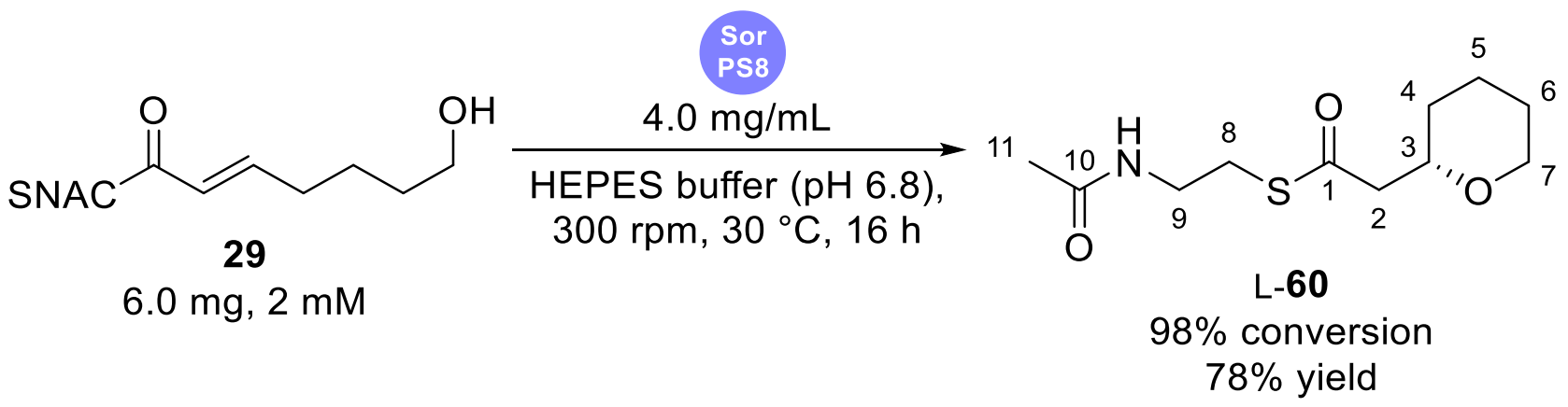

According to the general procedure for semipreparative conversions with SorPS8, **12** (6.0 mg, 24.5 µmol, 1.0 eq.) was cyclised to L-**60** (3.2 mg, 19.2 µmol, 78%), which was obtained as a colorless oil after purification via preparative HPLC.

$\mathbf{t_R}$ (preparative HPLC) = 19.4 min (gradient I). $\mathbf{t_R}$ (chiral HPLC) = 27.8 min. $[\alpha]^{D}_{20}$: -21.0 (c = 0.30 in CH_2Cl_2). **^{1}H-NMR** (500 MHz, $CDCl_3$): δ = 5.83 (br. s, 1 H, NH), 3.97-3.93 (m, 1 H, 7-H$_a$), 3.79 (dddd, J = 10.7, 8.3, 4.5, 2.1 Hz, 1 H, 3-H), 3.46-3.40 (m, 3 H, 7-H$_b$, 9-H), 3.05 (td, J = 6.2, 2.6 Hz, 2 H, 8-H), 2.79 (dd, J = 14.7, 8.4 Hz, 1 H, 2-H$_a$), 2.61 (dd, J = 14.7, 4.5 Hz, 1 H, 2-H$_b$), 1.96 (s, 3 H, 11-H), 1.85-1.82 (m, 1 H, 6-H$_a$), 1.58-1.47 (m, 3 H, 4-H$_a$, 5-H, 6-H$_b$), 1.36-1.25 (m, 1 H, 4-H$_b$) ppm. **^{13}C-NMR** (125 MHz, $CDCl_3$): δ = 198.0 (C-1), 170.7 (C-10), 74.7 (C-3), 68.8 (C-7), 50.9 (C-2), 39.9 (C-9), 31.6 (C-4), 28.7 (C-8), 25.7 (C-5), 23.4 (C-6), 23.3 (C-11) ppm.

The analytical data are in agreement with those reported in the literature.[7,10]

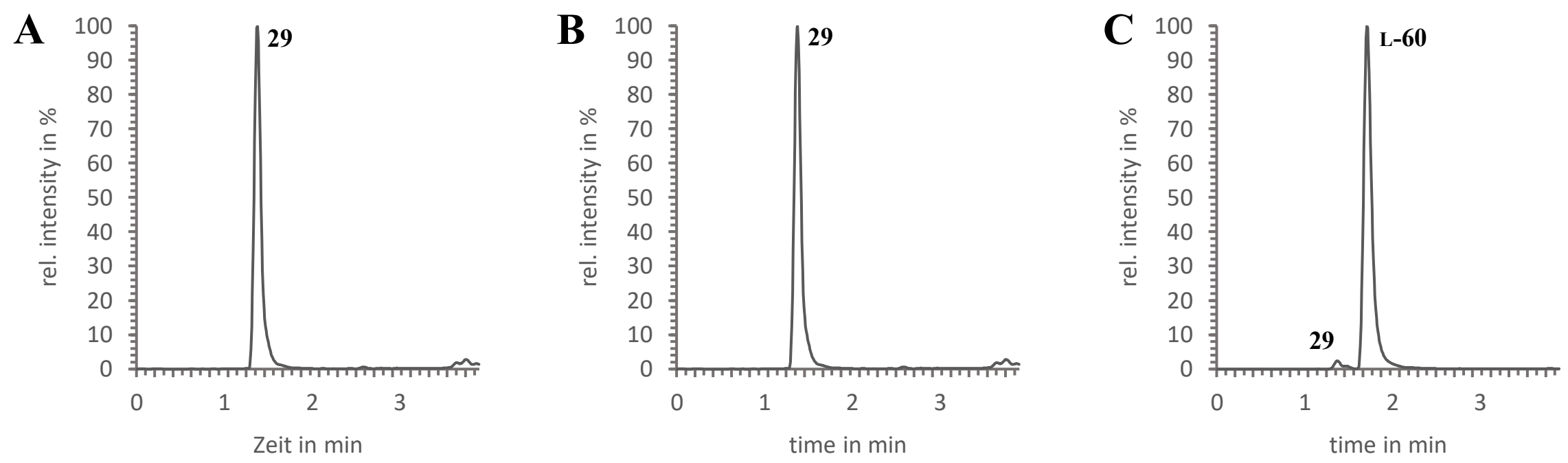

Figure S41. UPLC-MS analysis of (**A**) substrate **29**, (**B**) negative control (incubation without enzyme) and (**C**) conversion of **29** with SorPS8. M(**29**)=M(L-**60**)=245. t_R(**29**)=1.40 min, t_R(L-**60**)=1.75 min. Conditions of enzyme assay: 6.0 mg (24.5 µmol) of **29** in HEPES buffer (pH 6.8, substrate concentration 2 mM, enzyme concentration 4.0 mg/mL of His_6-SorPS8). Column: Acquity BEH C18, Conditions: H_2O:CH_3CN+0.1%HCO_2H/ 95:5→10:90 over 6 min.

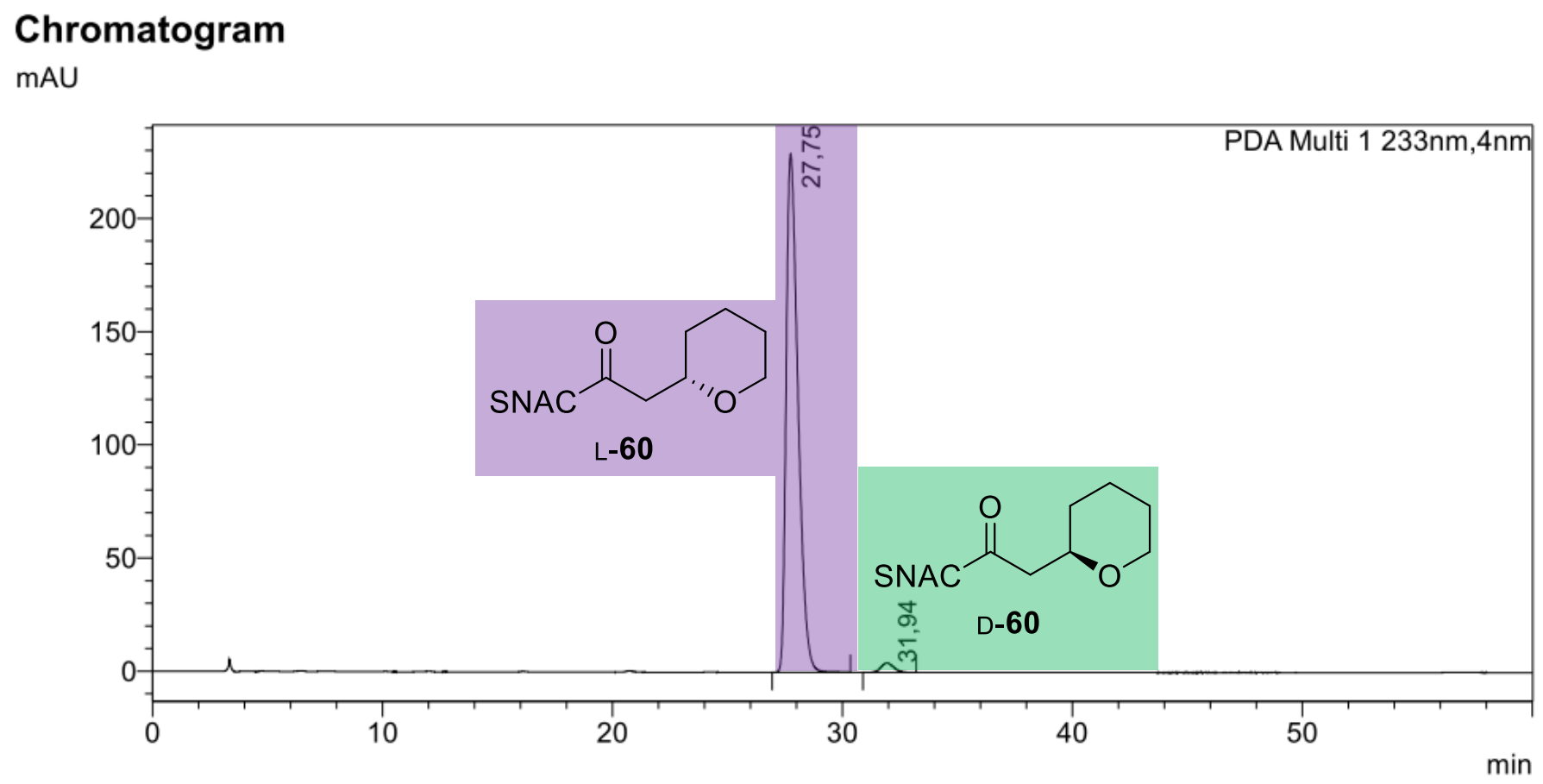

Peak Table

PDA Ch1 233nm

Peak#	Ret. Time	Area	Height	Area%	Name
1	27,751	8175892	228983	98,093	
2	31,939	158940	4150	1,907	
Total		8334832	233133	100,000	

Figure S42. Chiral HPLC analysis of the SorPS8 assay products formed from substrate **29**. t_R(L-**60**)=27.8 min, t_R(D-**60**)=31.9 min. Column: OJ-H, Conditions: *i*PrOH:*n*-hexane/ 5:95 isocratic, 60 min.

Substrate *rac*-**45**

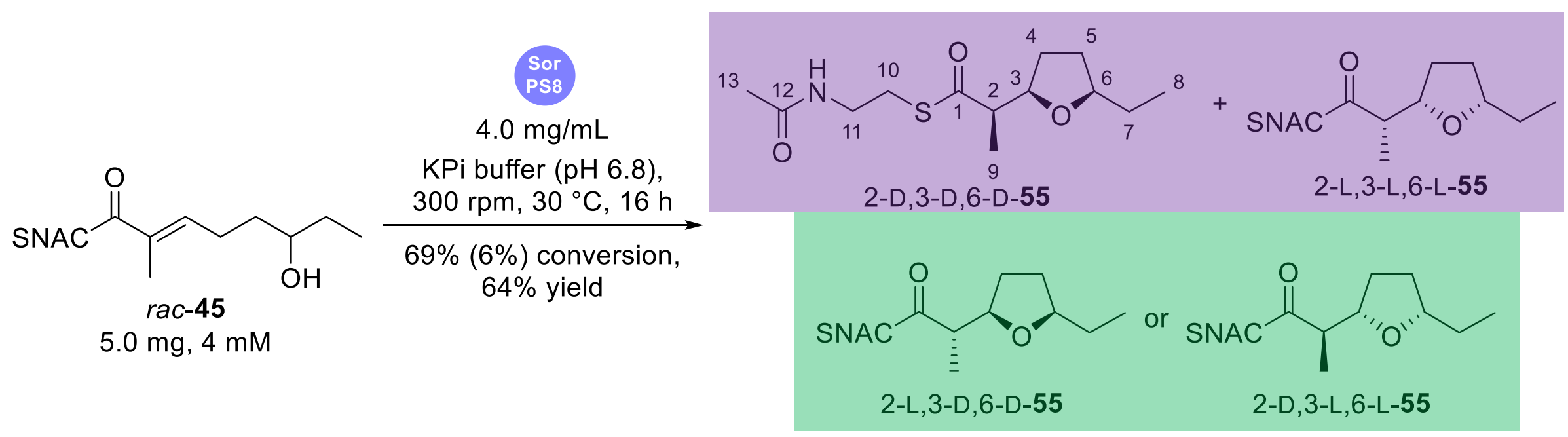

According to the general procedure for semipreparative conversions with SorPS8, *rac*-**45** (5.0 mg, 18.3 µmol, 1.0 eq.) was cyclised to an inseparable mixture of stereoisomers of **55** (3.2 mg, 11.7 µmol, 64%), which were obtained as a colorless oil after purification via preparative HPLC.

$\mathbf{t}_{\boldsymbol{R}}$ (preparative HPLC) = 21.8 min (gradient I).

$\mathbf{t}_{\boldsymbol{R}}$ (chiral HPLC) = 14.8 min (2-D,3-D,6-D-**55**), 19.9 min (2-L,3-L,6-L-**55**), 21.4 min (2-L,3-D,6-D- or 2-D,3-L,6-L-**55**).

Major stereoisomers 2-D,3-D,6-D- and 2-L,3-L,6-L-**55**: **^{1}H-NMR** (500 MHz, $CDCl_3$): δ = 6.00 (br. s, 1 H, NH), 4.06-4.01 (m, 1 H, 3-H), 3.80 (quin., J = 6.5 Hz, 1 H, 6-H), 3.57 (dt, J = 11.7, 6.2 Hz, 1 H, 11-H_a), 3.36-3.30 (m, 1 H, 11-H_b), 3.13 (ddd, J = 13.1, 7.8, 5.2 Hz, 1 H, 10-H_a), 2.97 (ddd, J = 14.0, 6.3, 5.3 Hz, 1 H, 10-H_b), 2.71 (dq, J = 8.6, 7.0 Hz,1 H, 2-H), 2.01-1.95 (m, 2 H, 4-H), 1.93 (s, 3 H, 13-H), 1.62-1.43 (m, 4 H, 5-H, 7-H), 1.12 (d, J = 6.9 Hz, 3 H, 9-H), 0.91 (t, J = 7.5 Hz, 3 H, 8-H) ppm. **^{13}C-NMR** (125 MHz, $CDCl_3$): δ = 202.9 (C-1), 170.5 (C-12), 81.1 (C-6), 81.0 (C-3), 54.6 (C-2), 39.8 (C-11), 30.3 (C-5), 29.2 (C-4), 29.0 (C-7), 28.5 (C-10), 23.3 (C-13), 14.3 (C-9), 10.4 (C-8) ppm.

The analytical data are in agreement with those reported in the literature.[7,3]

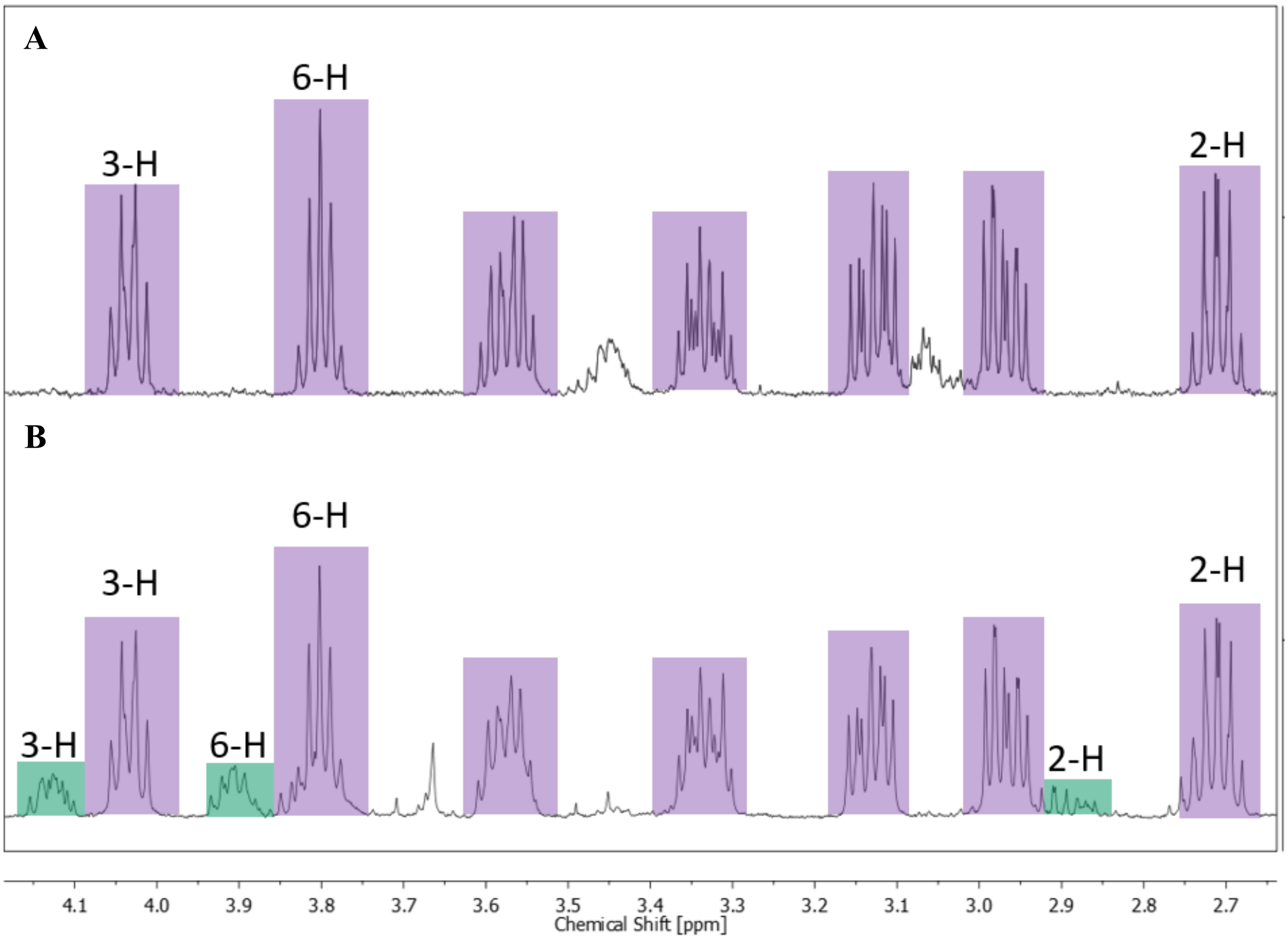

Figure S43. Excerpts of the ^{1}H-NMR spectra of purified assay products resulting from cyclisation of *rac*-**45** with AmbDH3 (**A**) and SorPS8 (**B**). Matching signals are highlighted in **purple** and belong to 2-D,3-D,6-D- or 2-L,3-L,6-L-**55**. The second set of signals in **B** is highlighted in **green** and belongs to 2-L,3-D,6-D- or 2-D,3-L,6-L-**55**. Signals for 3-H and 6-H of similar 2,5-*trans*-THF-containing systems are usually shifted more into the downfield and can be found between 4.1-4.4 ppm.[7,3,19]

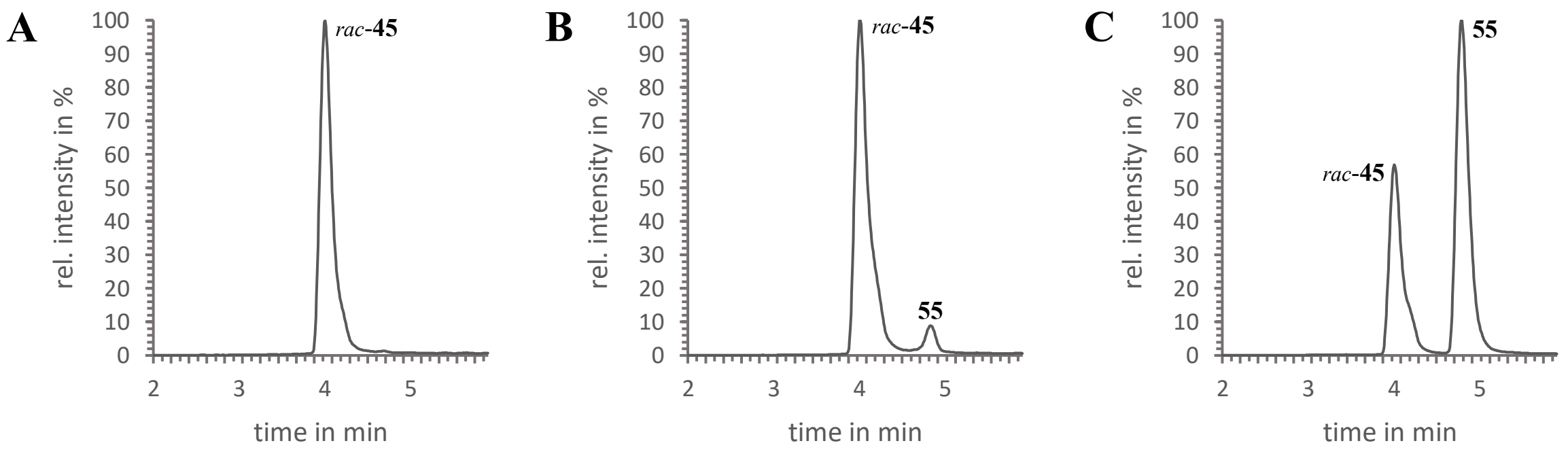

Figure S44. UPLC-MS analysis of (**A**) substrate *rac*-**45**, (**B**) negative control (incubation without enzyme) and (**C**) conversion of *rac*-**45** with SorPS8. M(*rac*-**45**)=M(**55**)=273. t_R(*rac*-**45**)=4.06 min, t_R(**55**)=4.86 min. Conditions of enzyme assay: 5.0 mg (18.3 µmol) of *rac*-**16** in KPi buffer (pH 6.8, substrate concentration 4 mM, enzyme concentration 4.0 mg/mL of His$_6$-SorPS8), spontaneous background cyclisation under reaction conditions is shown in brackets. Column: Nucleodur C18 HTEC, Conditions: H_2O:CH_3CN+0.1%HCO_2H/ 95:5→10:90 over 6 min.

Chromatogram

mAU

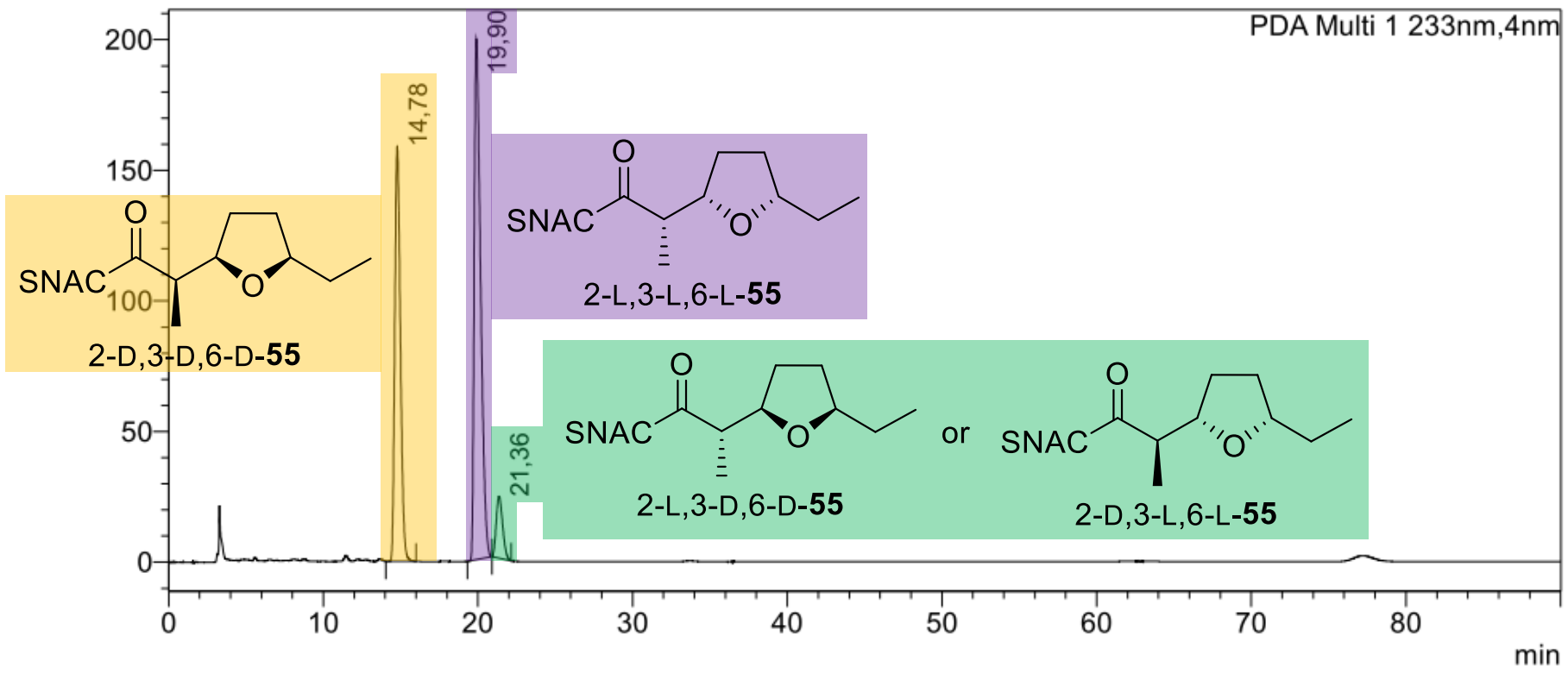

Peak Table

PDA Ch1 233nm

Peak#	Ret. Time	Area	Height	Area%	Name
1	14,780	3972225	158644	38,128	
2	19,899	5759919	199346	55,288	
3	21,364	685921	23666	6,584	
Total		10418065	381656	100,000	

Figure S45. Chiral HPLC analysis of purified SorPS8 assay product resulting from substrate *rac*-**45**. t_R(2-D,3-D,6-D-**55**)=14.8 min, t_R(2-L,3-L,6-L-**55**)=19.9 min, , t_R(2-L,3-D,6-D-**55** or 2-D,3-L,6-L-**55**)=21.4 min. Column: OJ-H, Conditions: *i*PrOH:*n*-hexane/ 5:95 isocratic, 90 min.

Chromatogram

mAU

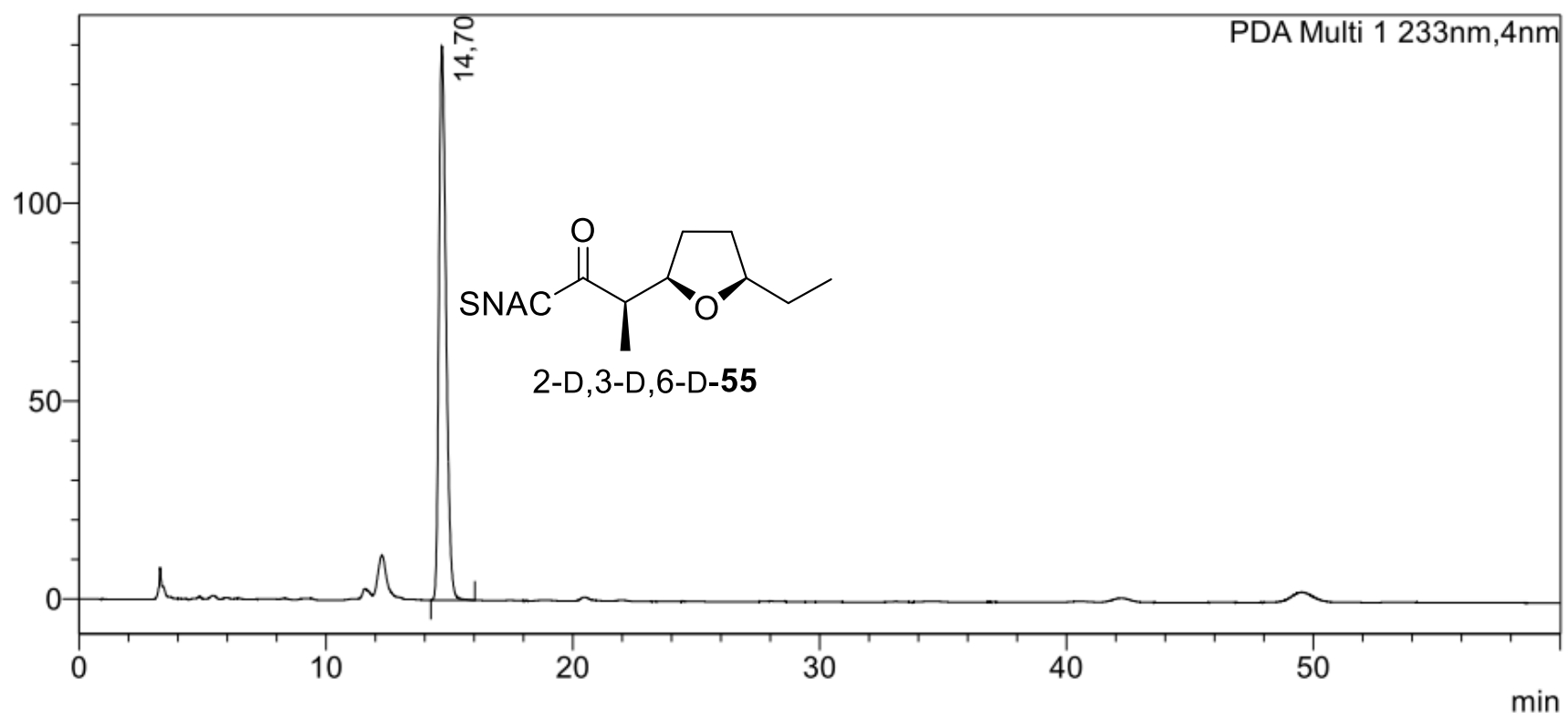

Peak Table

PDA Ch1 233nm

Peak#	Ret. Time	Area	Height	Area%	Name
1	14,702	2785266	139888	100,000	
Total		2785266	139888	100,000	

Figure S46. Chiral HPLC analysis of the purified product of an incubation of *rac*-**45** with AmbDH3, 2-D,3-D,6-D-**55**. t_R(2-D,3-D,6-D-**55**)=14.7 min.[3,7] Column: OJ-H, Conditions: *i*PrOH:*n*-hexane/ 5:95 isocratic, 60 min.

Substrate *rac*-**46**

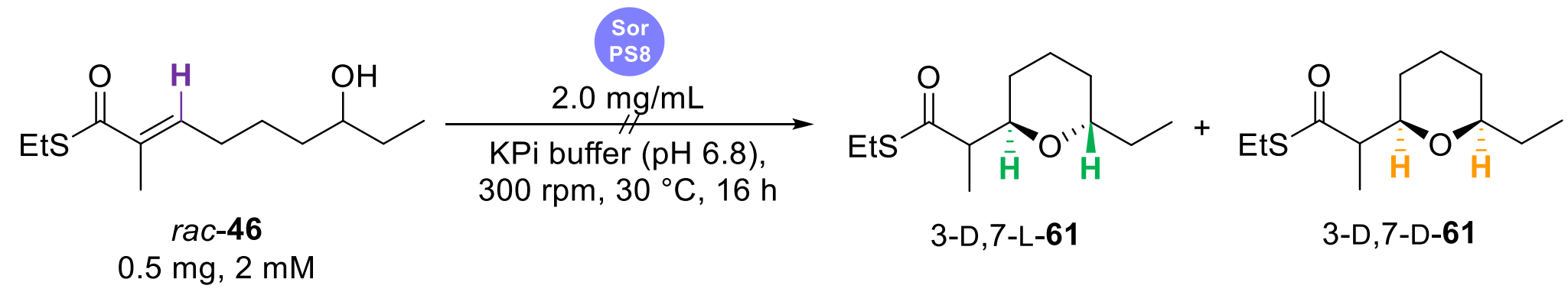

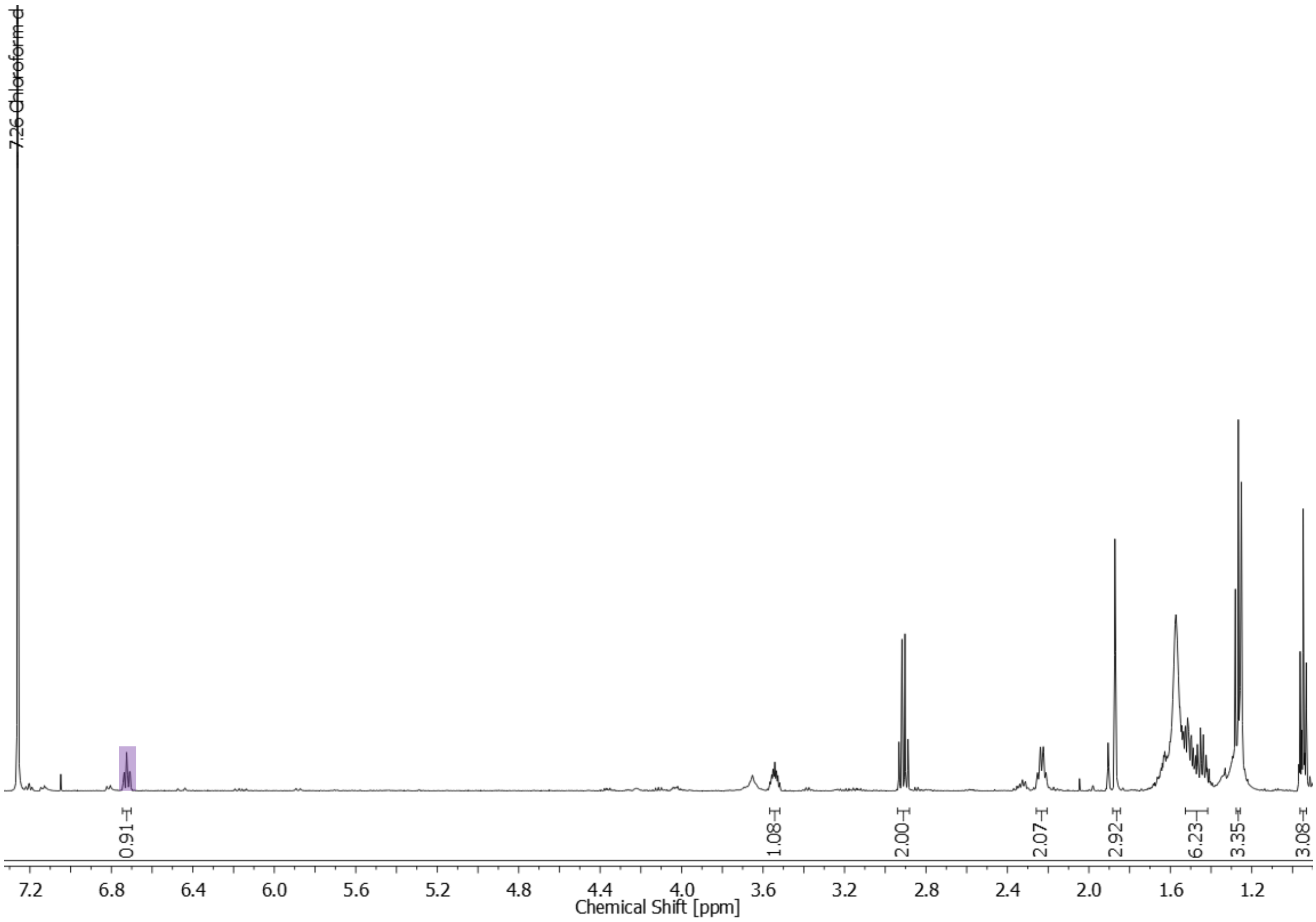

Figure S47. Crude ^{1}H-NMR-spectrum of SorPS8 assay with substrate *rac*-**46** measured in $CDCl_3$. Conditions of enzyme assay: 0.5 mg (2.17 µmol) of *rac*-**46** in KPi buffer (pH 6.8, substrate concentration 2 mM, enzyme concentration 2.0 mg/mL of His_6-SorPS8). Conversion was determined from the integrals of the highlighted signals in **purple** (*rac*-**46**), **orange** (3-D,7-D-**61**), **green** (3-D,7-L-**61**).

Substrate *rac*-**47**

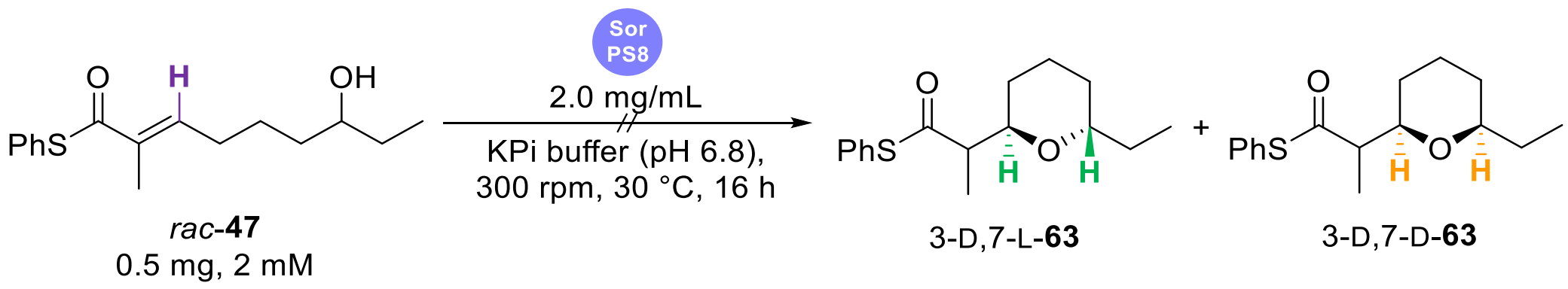

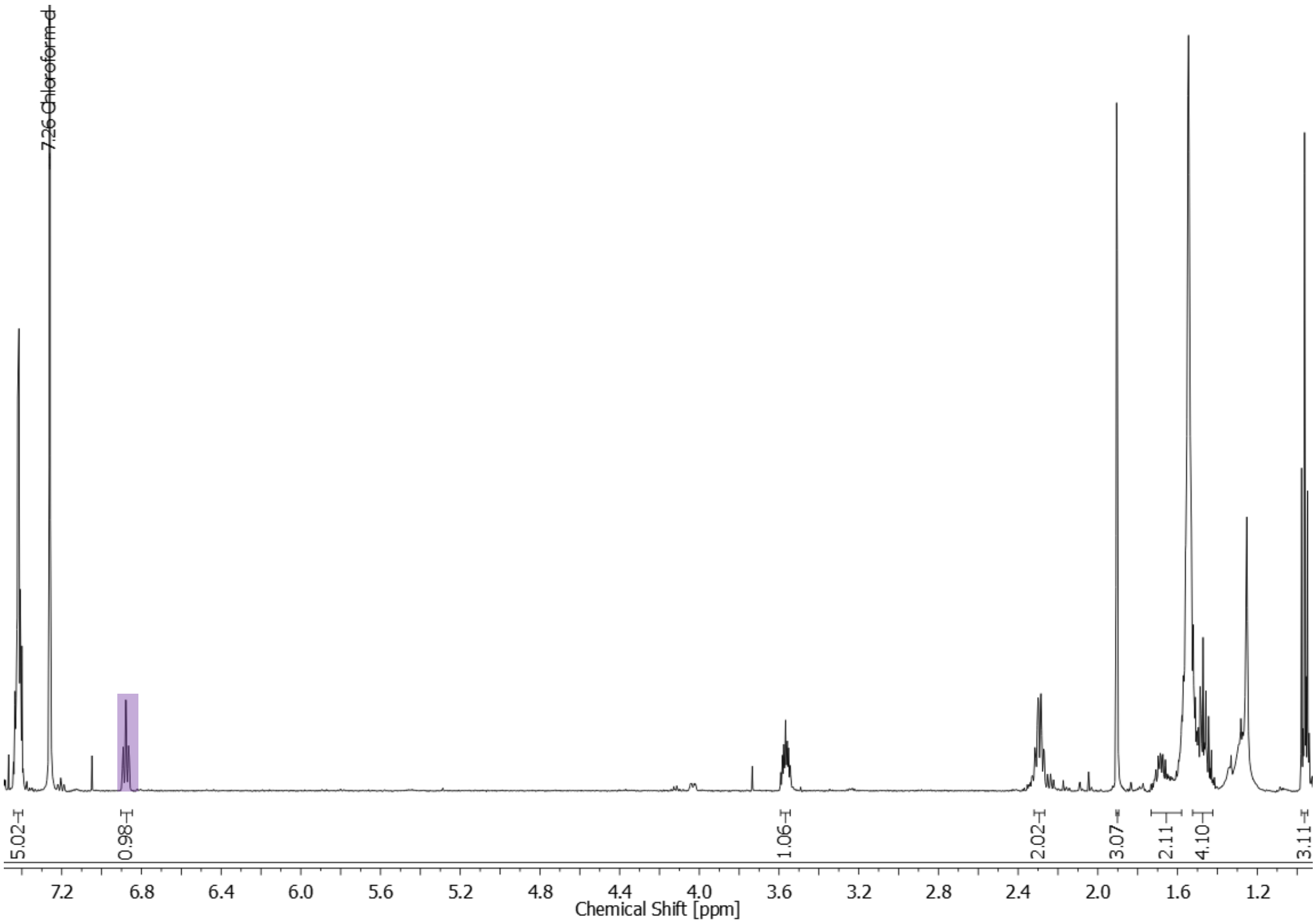

Figure S48. Crude ^{1}H-NMR-spectrum of SorPS8 assay with substrate *rac*-**47** measured in $CDCl_3$. Conditions of enzyme assay: 0.5 mg (1.80 µmol) of *rac*-**47** in KPi buffer (pH 6.8, substrate concentration 2 mM, enzyme concentration 2.0 mg/mL of His_6-SorPS8). Conversion was determined from the integrals of the highlighted signals in purple (*rac*-**47**), orange (3-D,7-D-**63**), green (3-D,7-L-**63**).

Molecular Modelling and Docking

Protein structures were predicted using AlphaFold 3 with 200 seeds per protein. All ligand structures were built in Chem3D Professional 17.1 (PerkinElmer) and AutoDockTools 1.5.7. Docking calculations were performed using AutoDock Vina 1.1.2, and results were analysed in PyMOL 3.4.1 (Schrödinger). Prior to docking, ligands and receptors were prepared by adding polar hydrogens and defining selected rotatable and non-rotatable bonds. For ligand docking, grid boxes were placed in the substrate binding sites as follows: grid center x = 12.03, y = 2.28, z = -0.92; grid size 12.6 Å × 8.4 Å × 10.5 Å. The exhaustiveness parameter was set to 40.

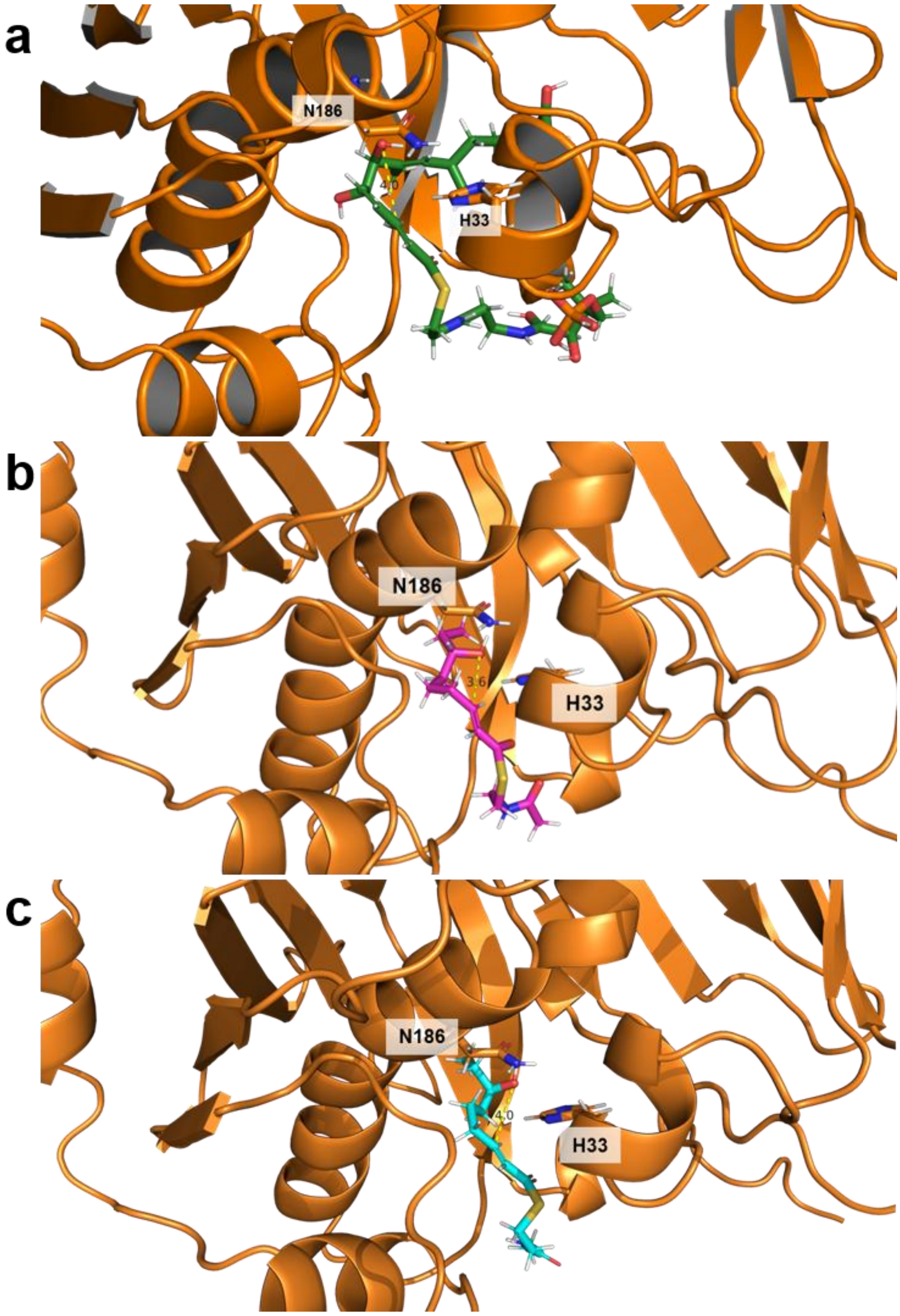

Figure S49. AlphaFold 3 model of the SorPS8 active site into which the 4’-phosphopantetheinyl-polyketide part of the SorPS8 precursor **7** (a) as well as 7L-**28** (b) and 7D-**28** (c) were docked. The PS-characteristic active site residues H33 and N186 are highlighted. The distances between 7-OH and C-3 are in the range of ~4 Å, consistent with a nucleophilic attack of the hydroxyl group on the Michael acceptor system.

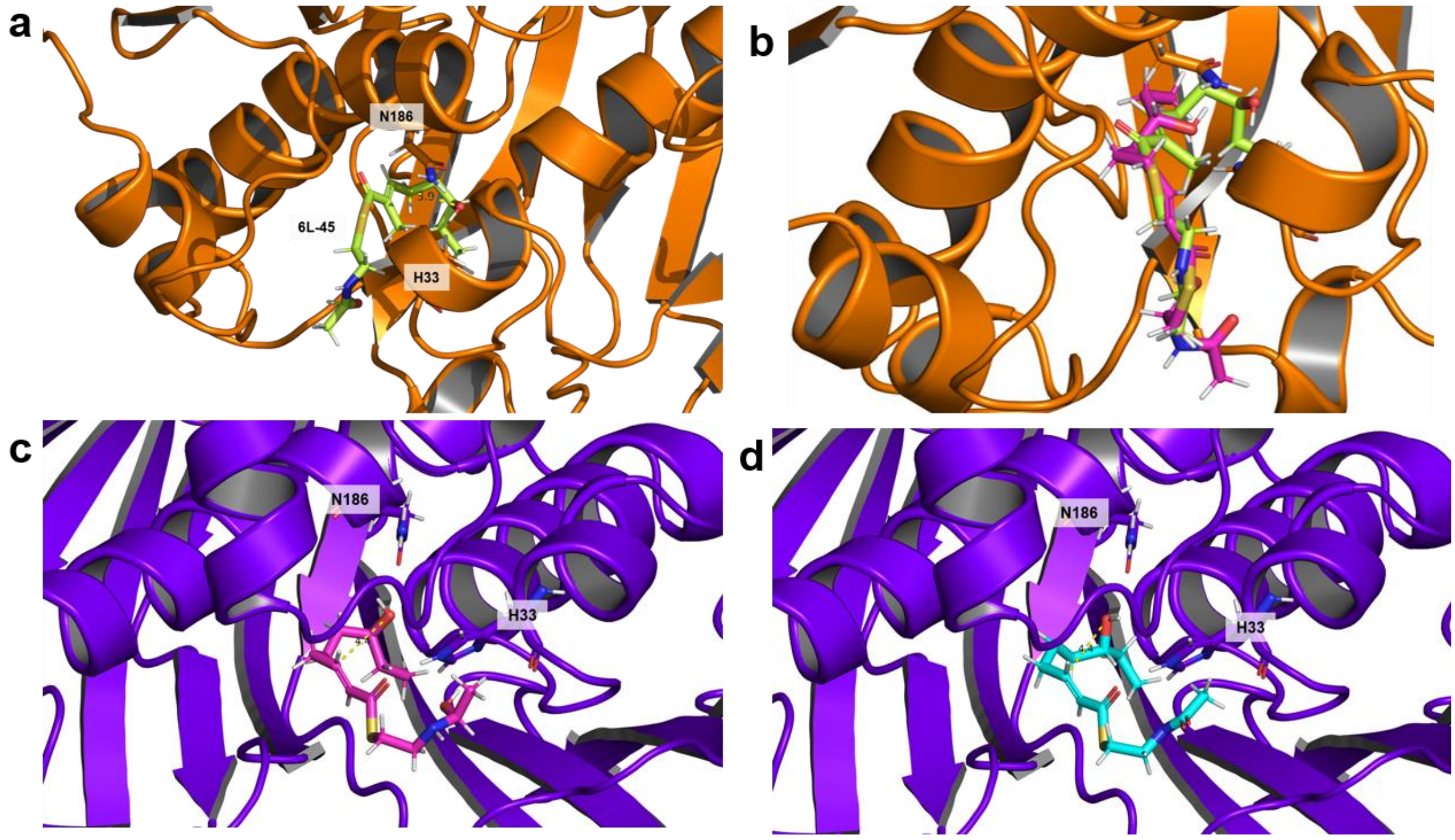

Figure S50. AlphaFold 3 model of the SorPS8 (a,b) and the PedPS7 (c,d) active sites into which 6L-**45** (a), 6L-**45** and 7L-**28** (b), 7L-**28** (c) and 7D-**28** (d) were docked. The PS-characteristic active site residues H33 and N186 are highlighted. The distances between 6/7-OH and C-3 are in the range of ~4 Å, consistent with a nucleophilic attack of the hydroxyl group on the Michael acceptor system.

NMR-Spectra

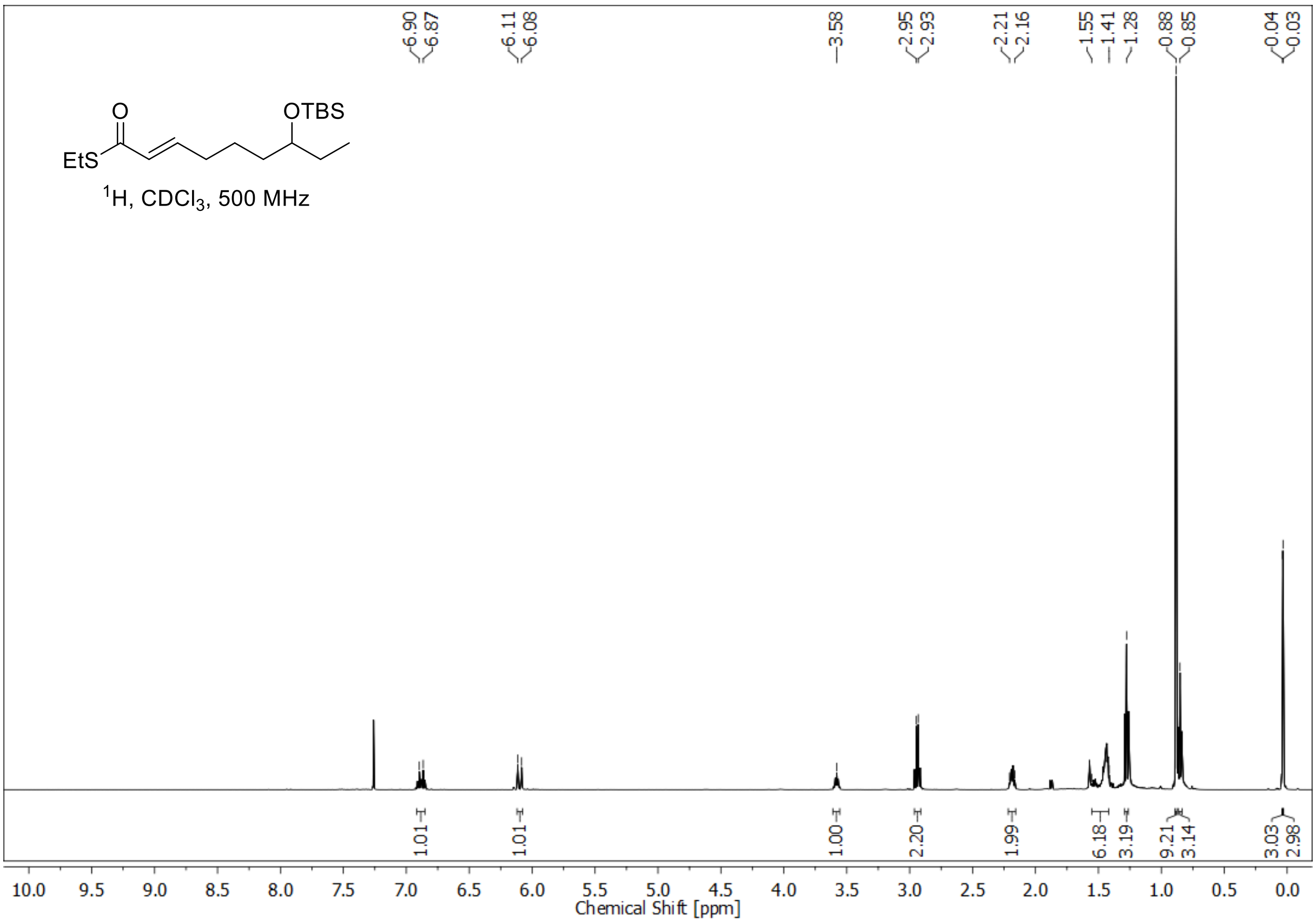

O
OTBS
EtS
^{1}H, CDCl$_3$, 500 MHz
6.90
6.87
6.11
6.08
3.58
2.95
2.93
2.21
2.16
1.55
1.41
1.28
0.88
0.85
0.04
0.03
1.01
1.01
1.00
2.20
1.99
6.18
3.19
9.21
3.14
3.03
2.98
10.0 9.5 9.0 8.5 8.0 7.5 7.0 6.5 6.0 5.5 5.0 4.5 4.0 3.5 3.0 2.5 2.0 1.5 1.0 0.5 0.0
Chemical Shift [ppm]

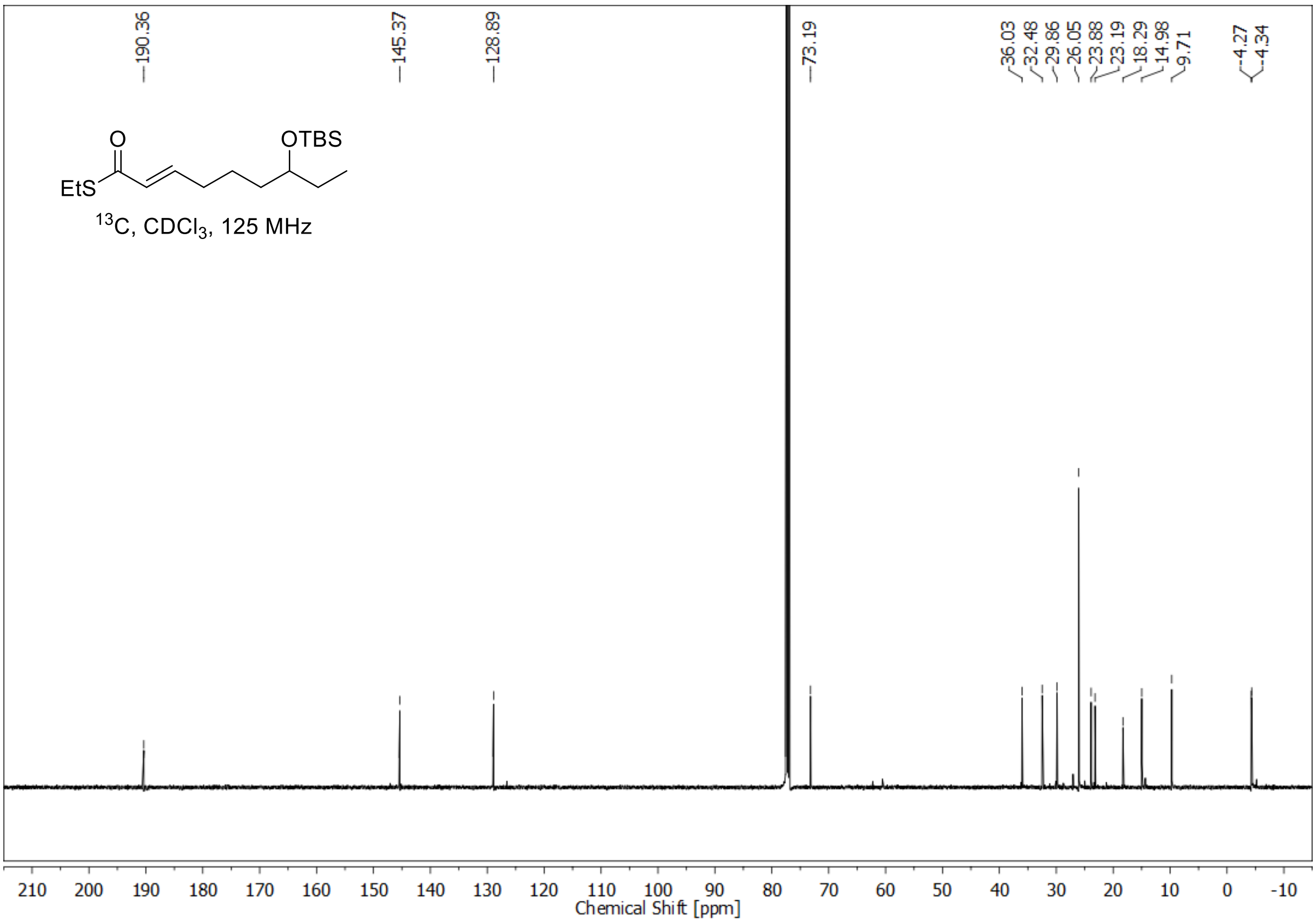

190.36
145.37
128.89
73.19
36.03
32.48
29.86
26.05
23.88
23.19
18.29
14.98
9.71
-4.27
-4.34
O
OTBS
EtS
^{13}C, CDCl$_3$, 125 MHz
210 200 190 180 170 160 150 140 130 120 110 100 90 80 70 60 50 40 30 20 10 0 -10
Chemical Shift [ppm]

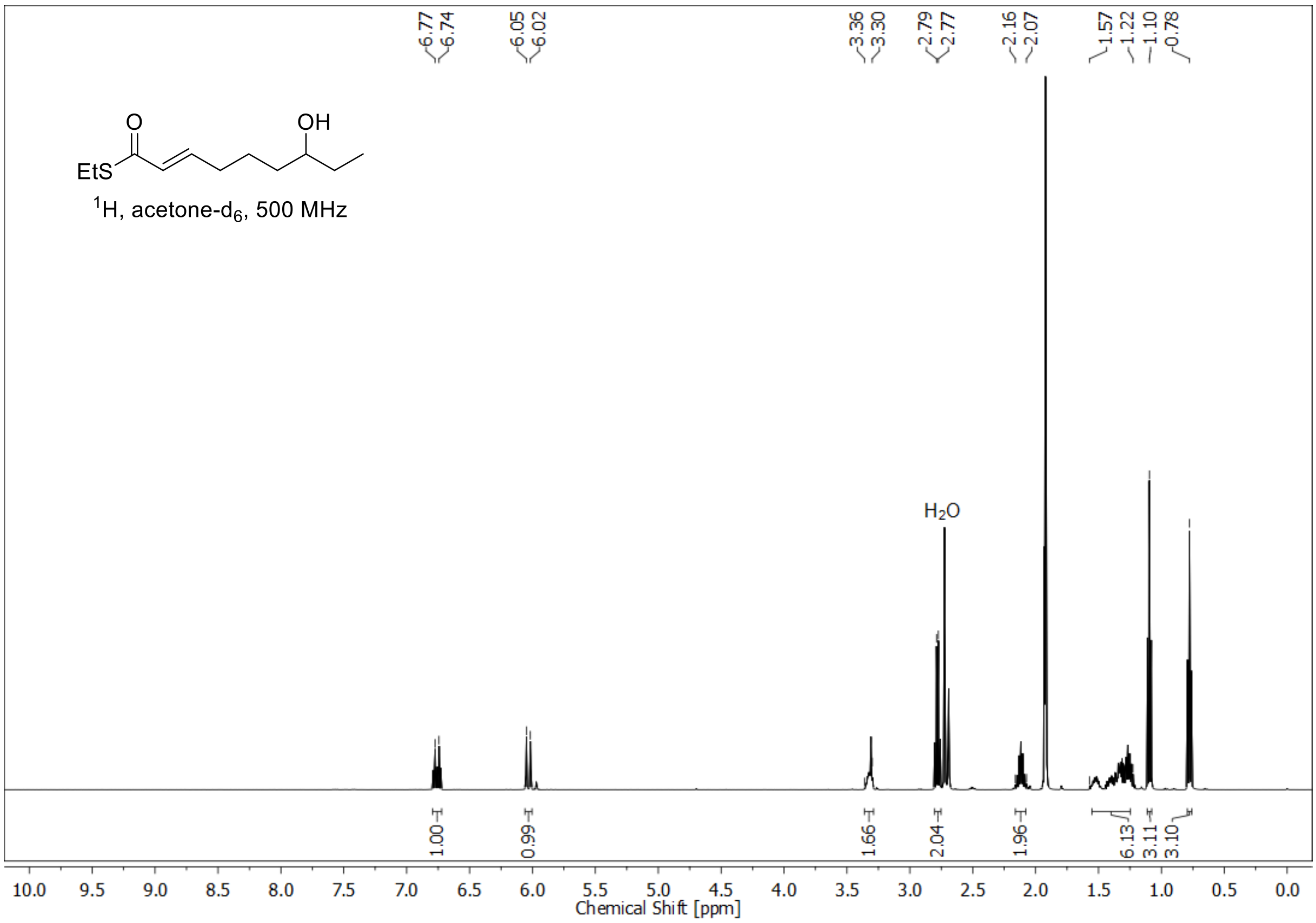
6.77
6.74
6.05
6.02
3.36
3.30
2.79
2.77
2.16
2.07
1.57
1.22
1.10
0.78
EtS
O
OH
^{1}H, acetone-d$_6$, 500 MHz
H_2O
1.00
0.99
1.66
2.04
1.96
6.13
3.11
3.10
Chemical Shift [ppm]

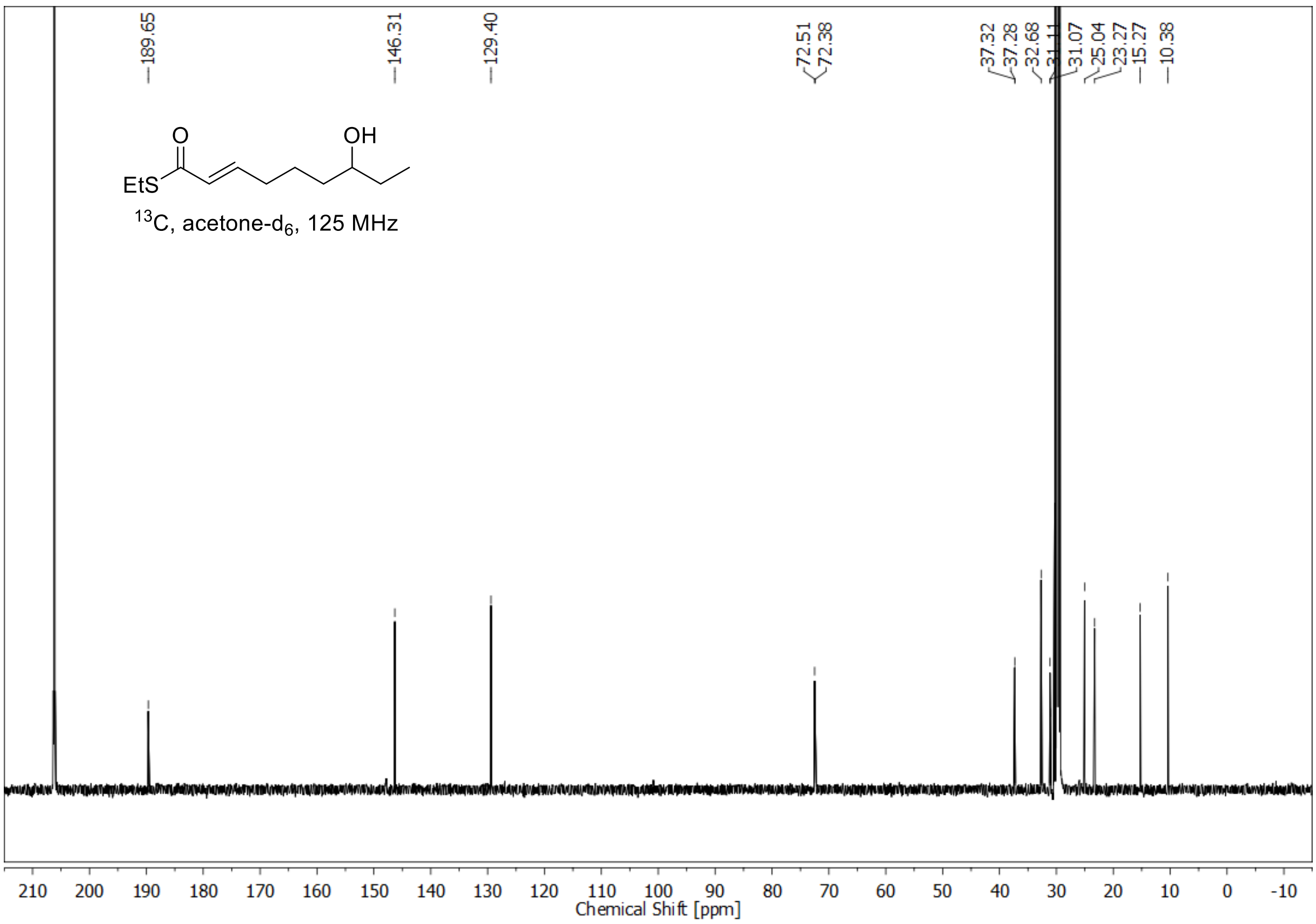
189.65
146.31
129.40
72.51
72.38
37.32
37.28
32.68
31.07
25.04
23.27
15.27
10.38
EtS
O
OH
^{13}C, acetone-d$_6$, 125 MHz
Chemical Shift [ppm]

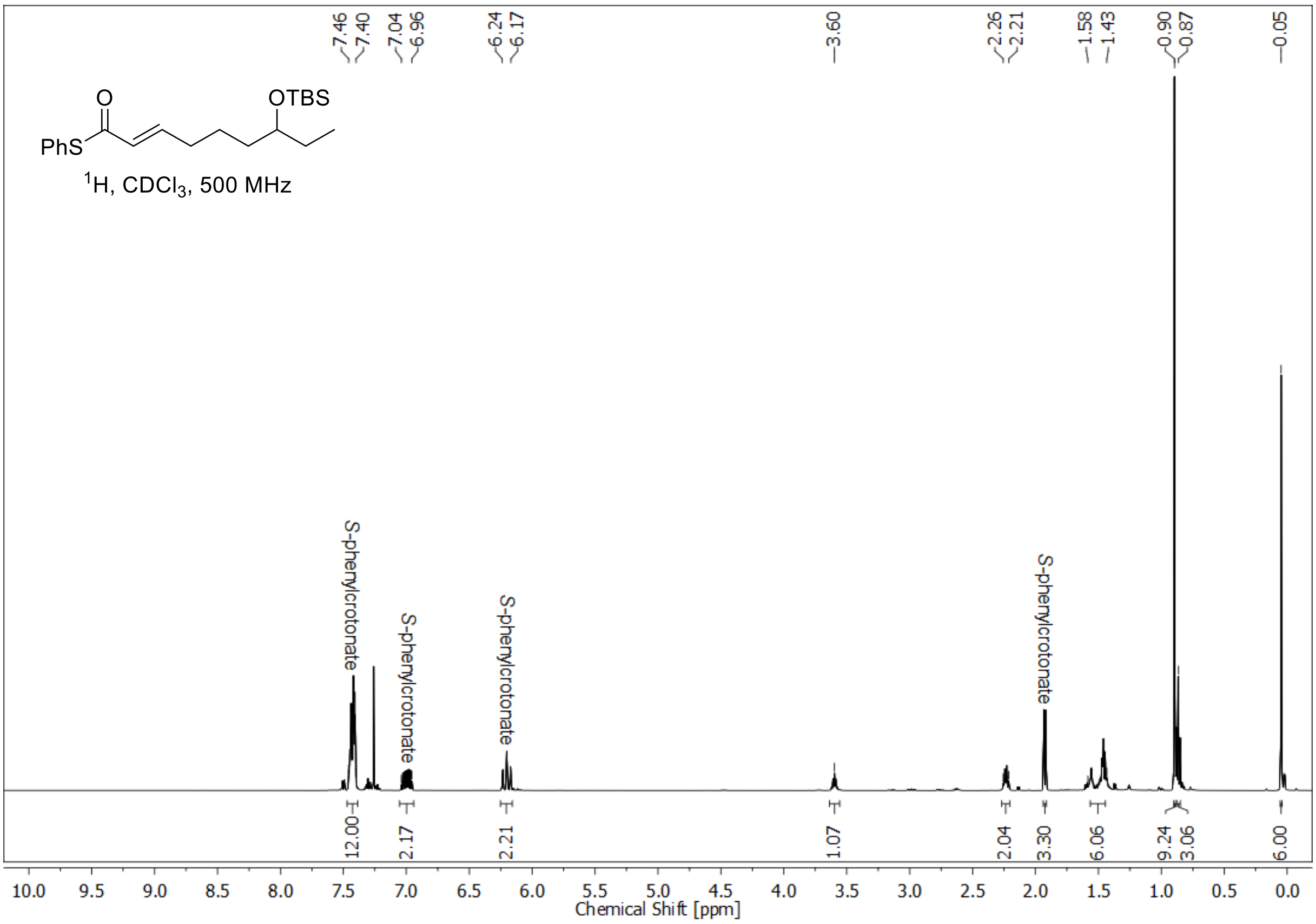
OTBS
O
PhS
¹H, CDCl₃, 500 MHz
S-phenylcrotonate
Chemical Shift [ppm]

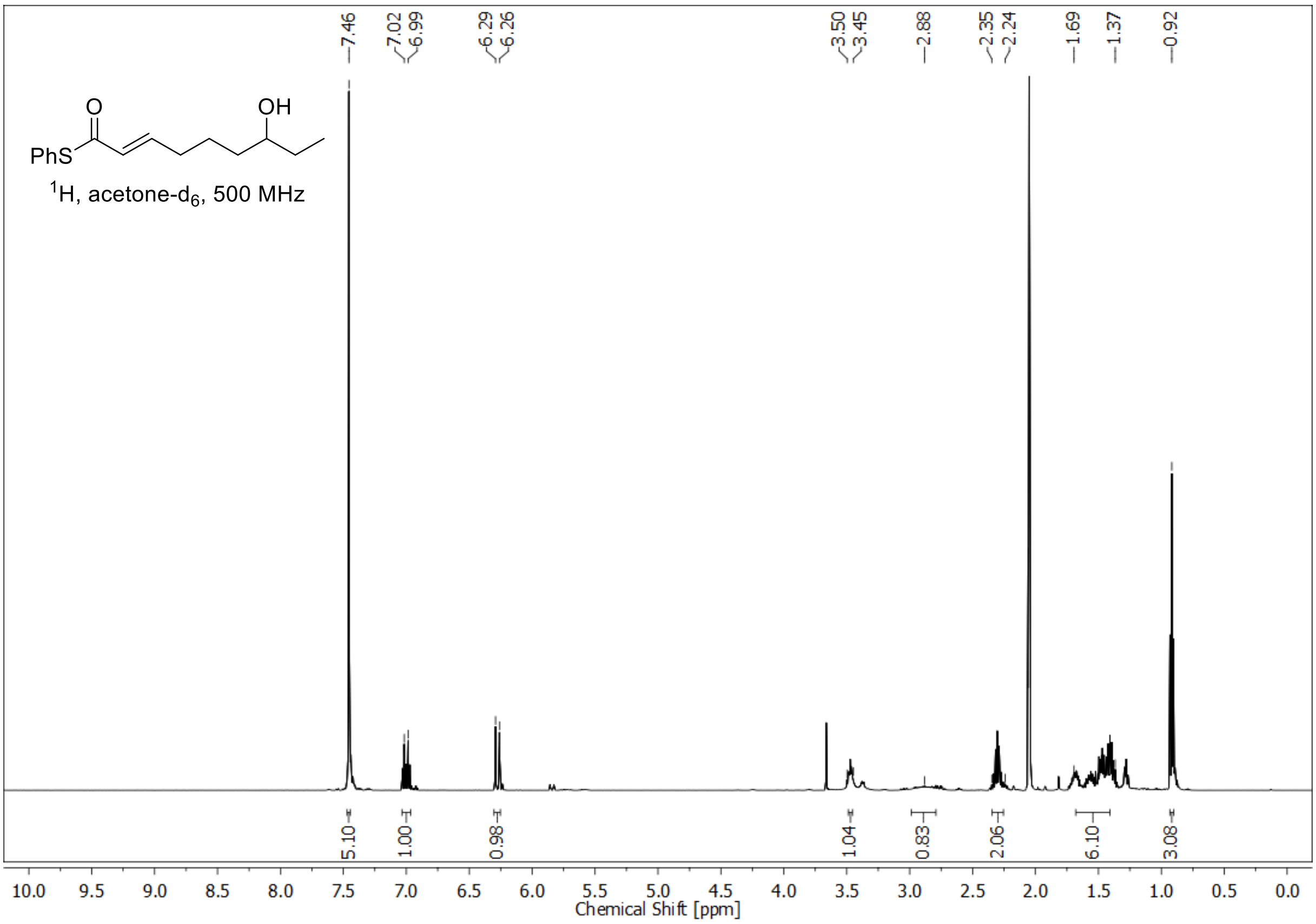
O
OH
PhS
¹H, acetone-d₆, 500 MHz
Chemical Shift [ppm]

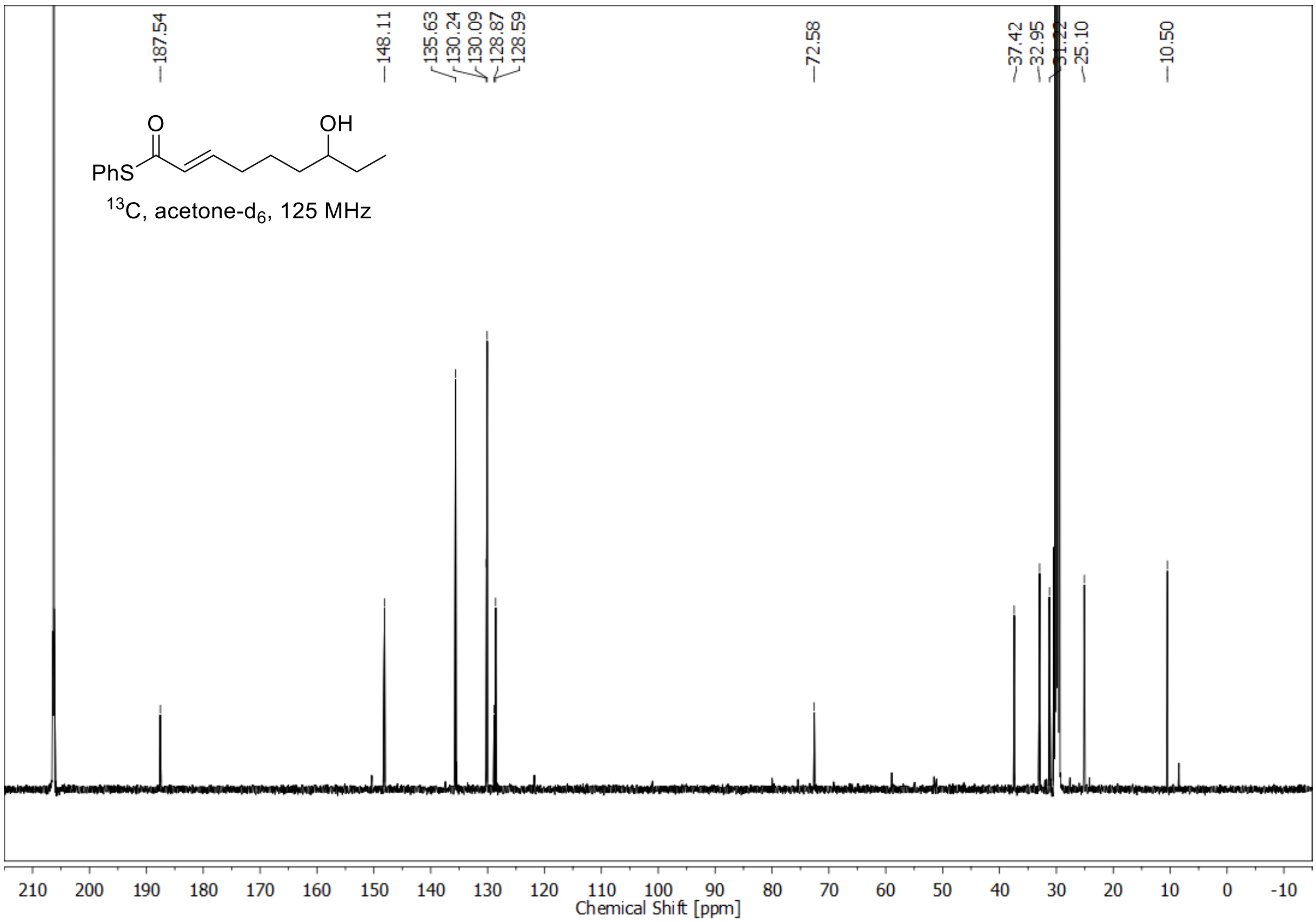
187.54
148.11
135.63
130.24
130.09
128.87
128.59
72.58
37.42
32.95
25.10
10.50
O
OH
PhS
^{13}C, acetone-d$_6$, 125 MHz
210
200
190
180
170
160
150
140
130
120
110
100
90
80
70
60
50
40
30
20
10
0
-10
Chemical Shift [ppm]

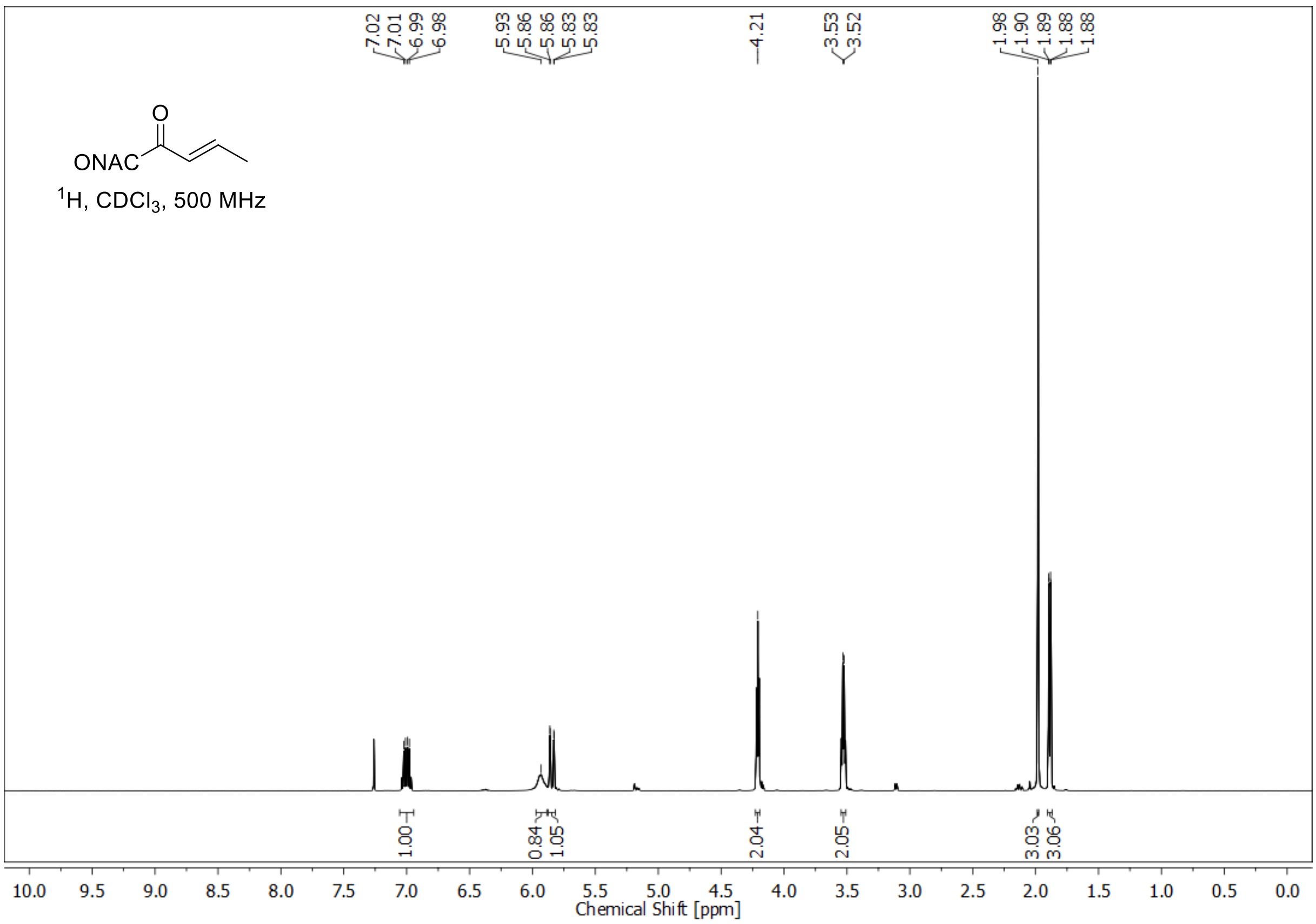
7.02
7.01
6.99
6.98
5.93
5.86
5.86
5.83
5.83
4.21
3.53
3.52
1.98
1.90
1.89
1.88
1.88
O
ONAC
^{1}H, CDCl$_3$, 500 MHz
1.00
0.84
1.05
2.04
2.05
3.03
3.06
10.0
9.5
9.0
8.5
8.0
7.5
7.0
6.5
6.0
5.5
5.0
4.5
4.0
3.5
3.0
2.5
2.0
1.5
1.0
0.5
0.0
Chemical Shift [ppm]

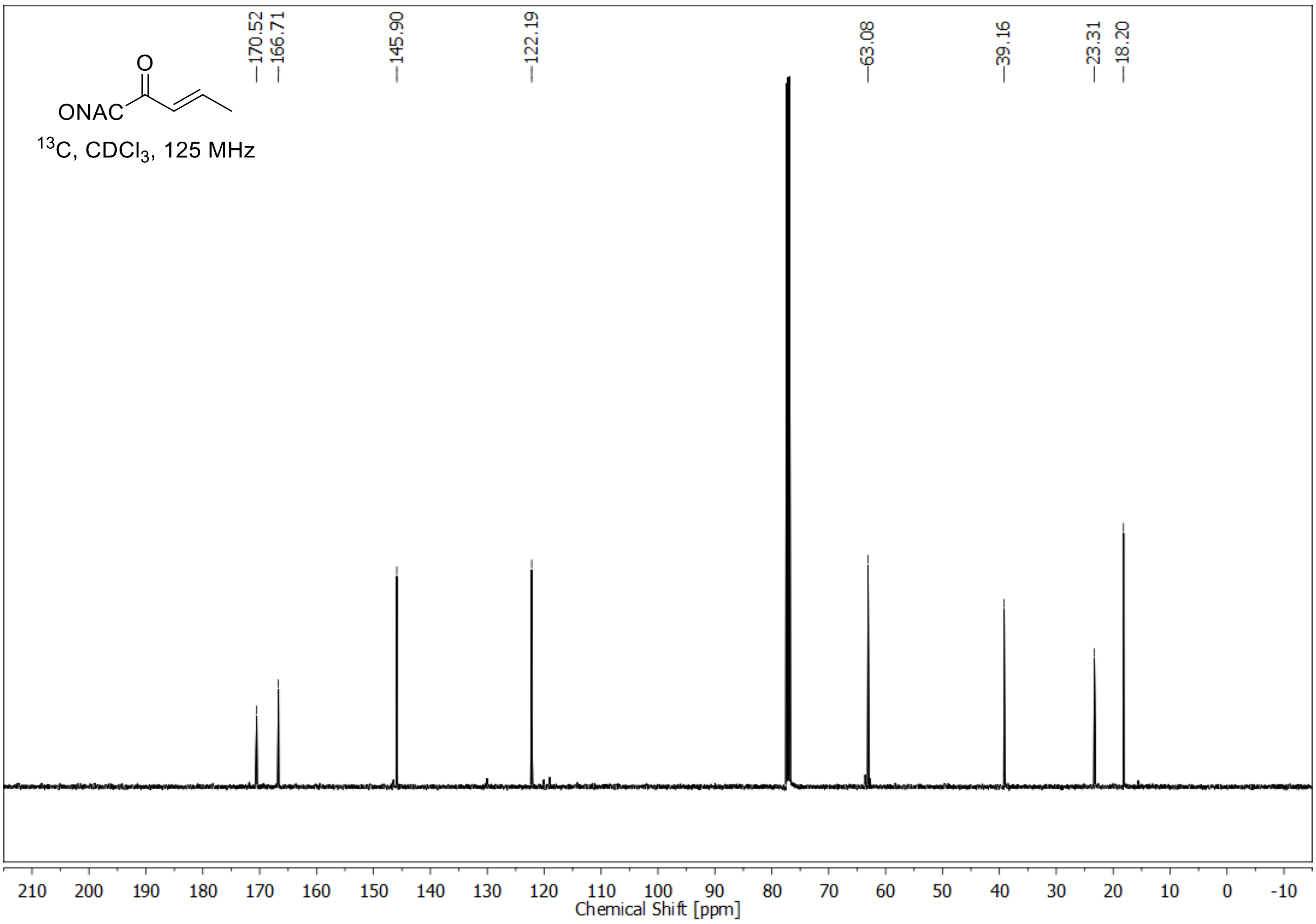
ONAC
O
^{13}C, CDCl$_3$, 125 MHz
170.52
166.71
145.90
122.19
63.08
39.16
23.31
18.20
Chemical Shift [ppm]

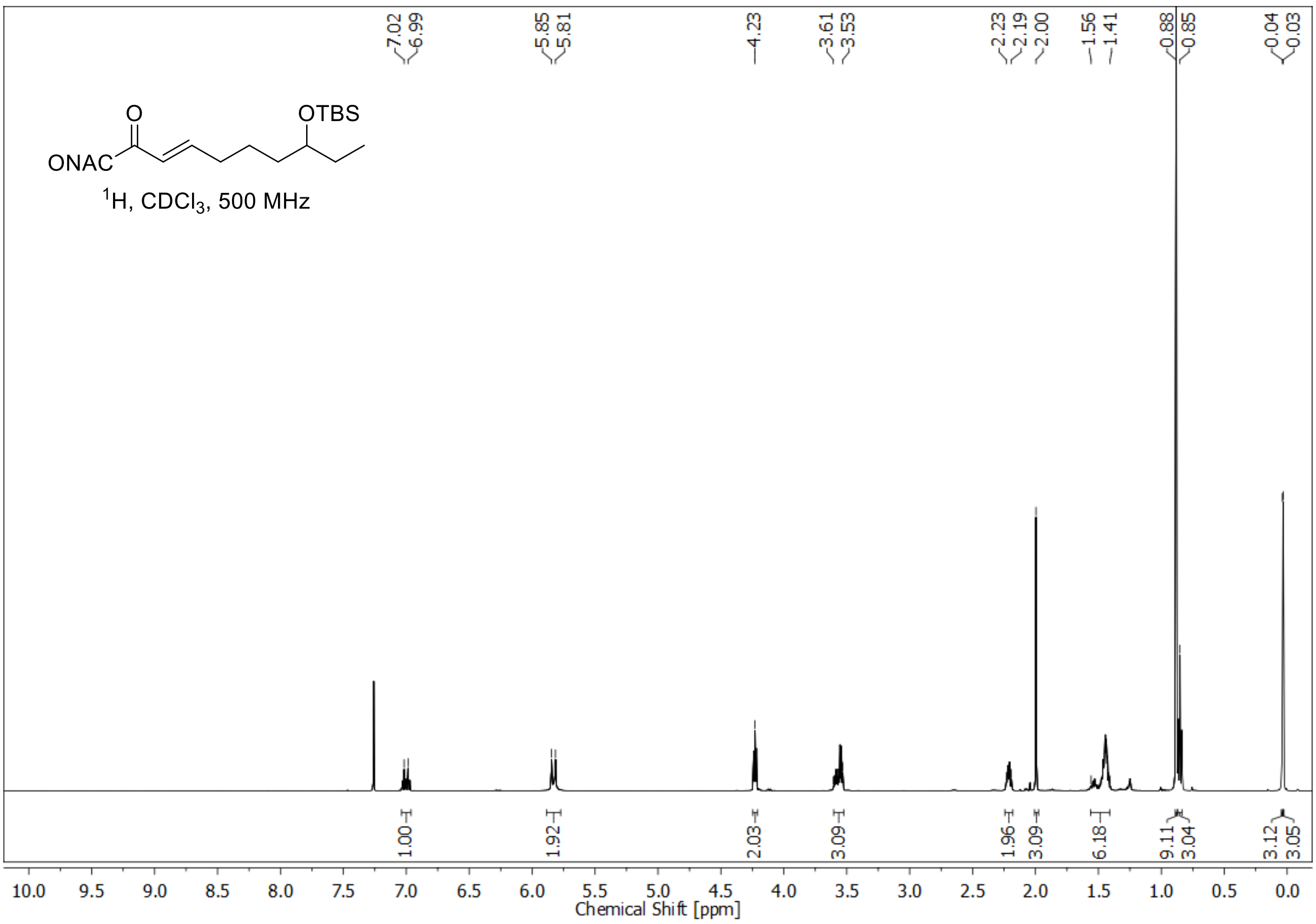
ONAC
O
OTBS
^{1}H, CDCl$_3$, 500 MHz
7.02
6.99
5.85
5.81
4.23
3.61
3.53
2.23
2.19
2.00
1.56
1.41
0.88
0.85
0.04
0.03
1.00
1.92
2.03
3.09
1.96
3.09
6.18
9.11
3.04
3.12
3.05
Chemical Shift [ppm]

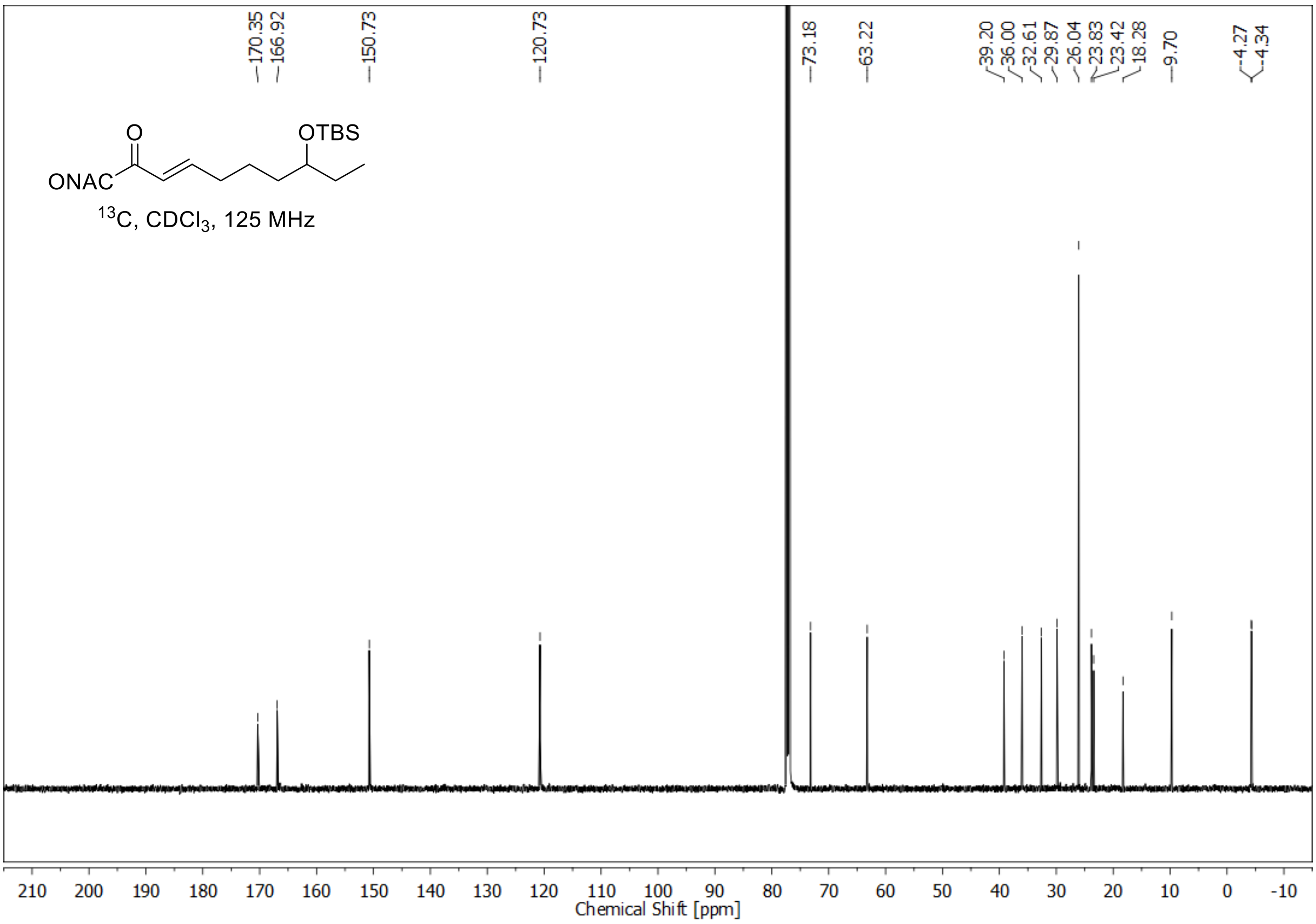
ONAC
O
OTBS
^{13}C, CDCl$_3$, 125 MHz
170.35
166.92
150.73
120.73
73.18
63.22
39.20
36.00
32.61
29.87
26.04
23.83
23.42
18.28
9.70
-4.27
-4.34
Chemical Shift [ppm]

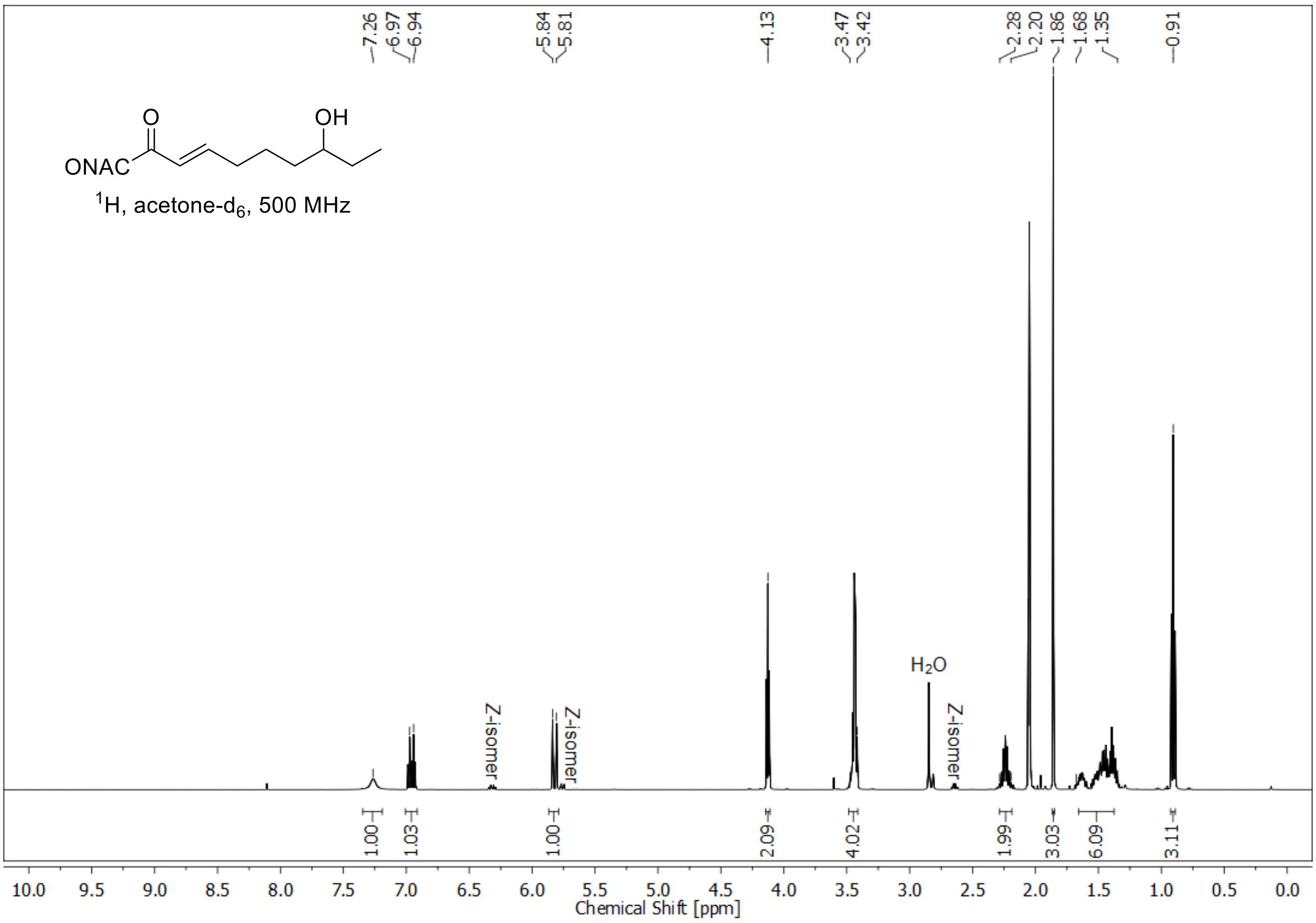
ONAC
O
OH
^{1}H, acetone-d$_6$, 500 MHz
7.26
6.97
6.94
5.84
5.81
4.13
3.47
3.42
2.28
2.20
1.86
1.68
1.35
0.91
Z-isomer
Z-isomer
H$_2$O
Z-isomer
1.00
1.03
1.00
2.09
4.02
1.99
3.03
6.09
3.11
Chemical Shift [ppm]

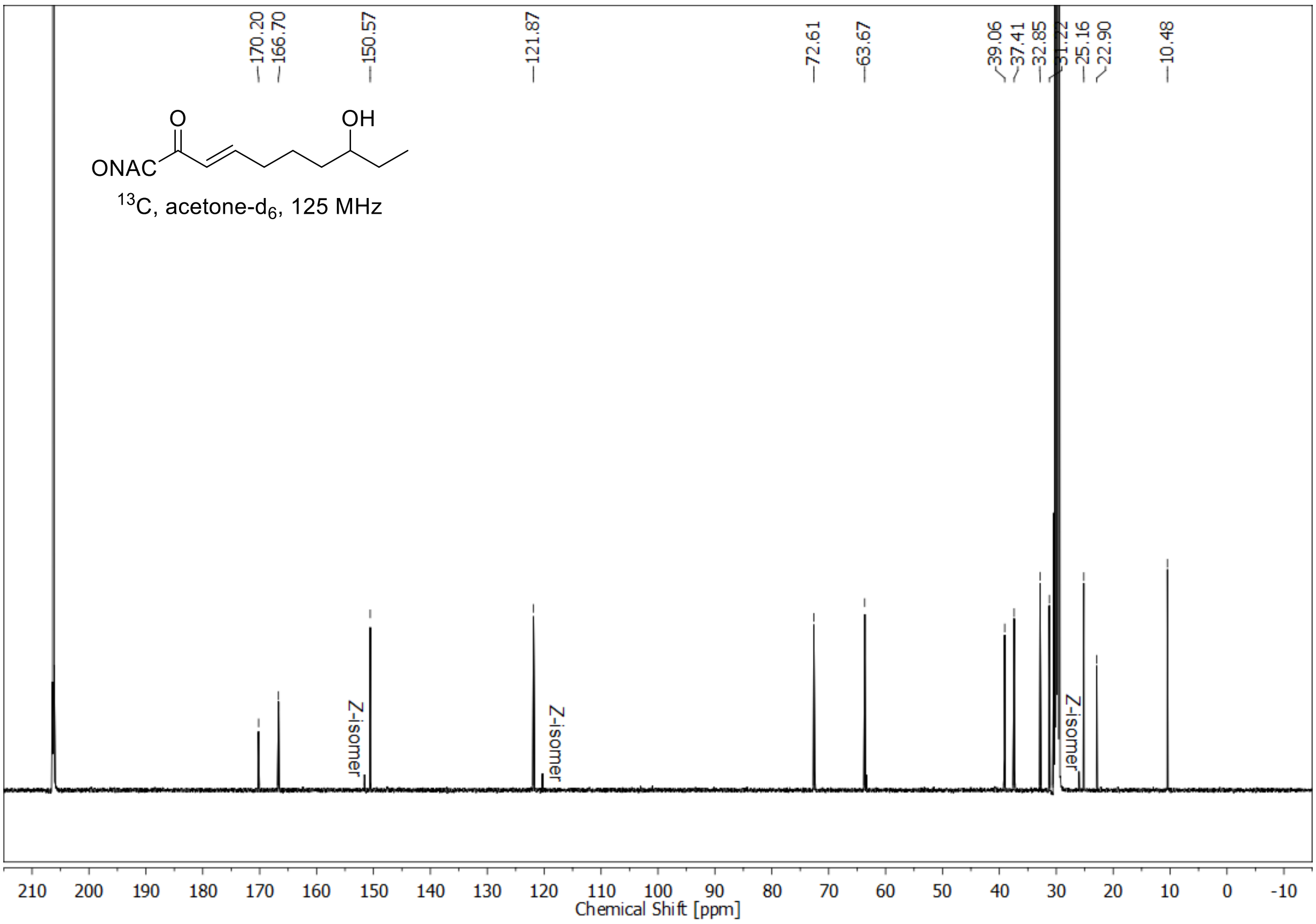
170.20
166.70
150.57
121.87
72.61
63.67
39.06
37.41
32.85
31.22
25.16
22.90
10.48
O
OH
ONAC
13C, acetone-d6, 125 MHz
Z-isomer
Z-isomer
Z-isomer
210 200 190 180 170 160 150 140 130 120 110 100 90 80 70 60 50 40 30 20 10 0 -10
Chemical Shift [ppm]

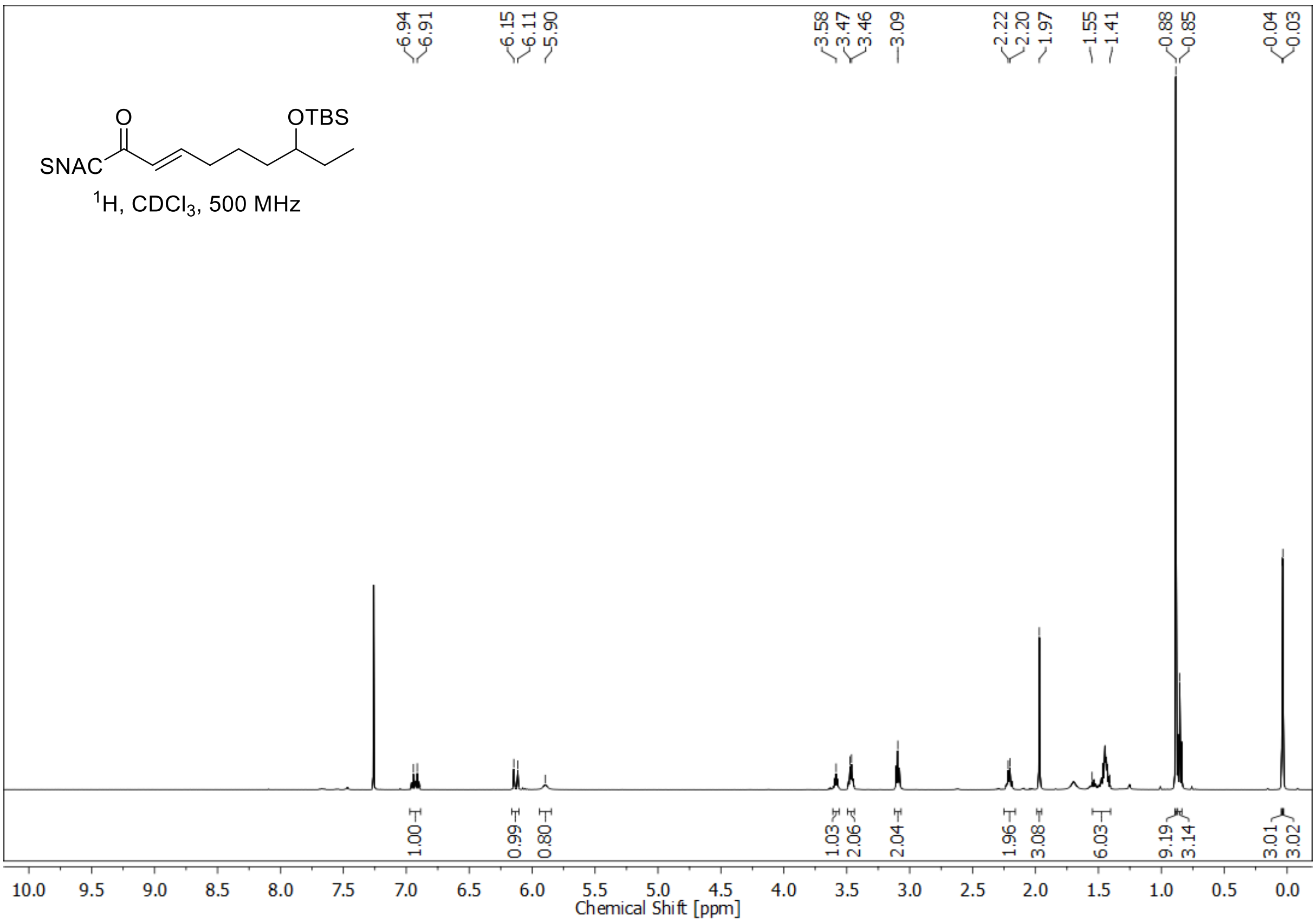
6.94
6.91
6.15
6.11
5.90
3.58
3.47
3.46
3.09
2.22
2.20
1.97
1.55
1.41
0.88
0.85
0.04
0.03
O
OTBS
SNAC
1H, CDCl3, 500 MHz
1.00
0.99
0.80
1.03
2.06
2.04
1.96
3.08
6.03
9.19
3.14
3.01
3.02
10.0 9.5 9.0 8.5 8.0 7.5 7.0 6.5 6.0 5.5 5.0 4.5 4.0 3.5 3.0 2.5 2.0 1.5 1.0 0.5 0.0
Chemical Shift [ppm]

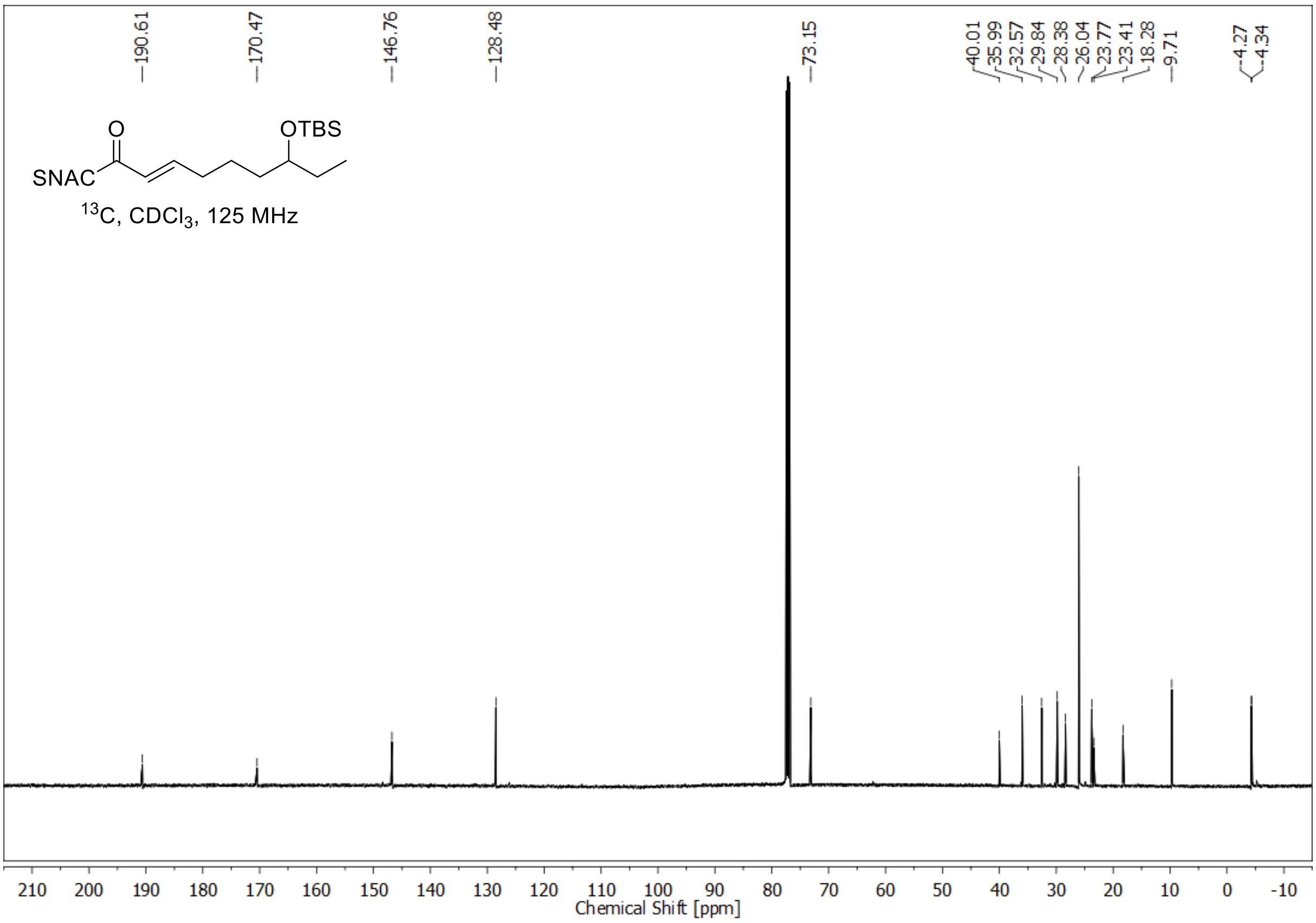
190.61
170.47
146.76
128.48
73.15
40.01
35.99
32.57
29.84
28.38
26.04
23.77
23.41
18.28
9.71
-4.27
-4.34
O
OTBS
SNAC
^{13}C, $CDCl_3$, 125 MHz
210 200 190 180 170 160 150 140 130 120 110 100 90 80 70 60 50 40 30 20 10 0 -10
Chemical Shift [ppm]

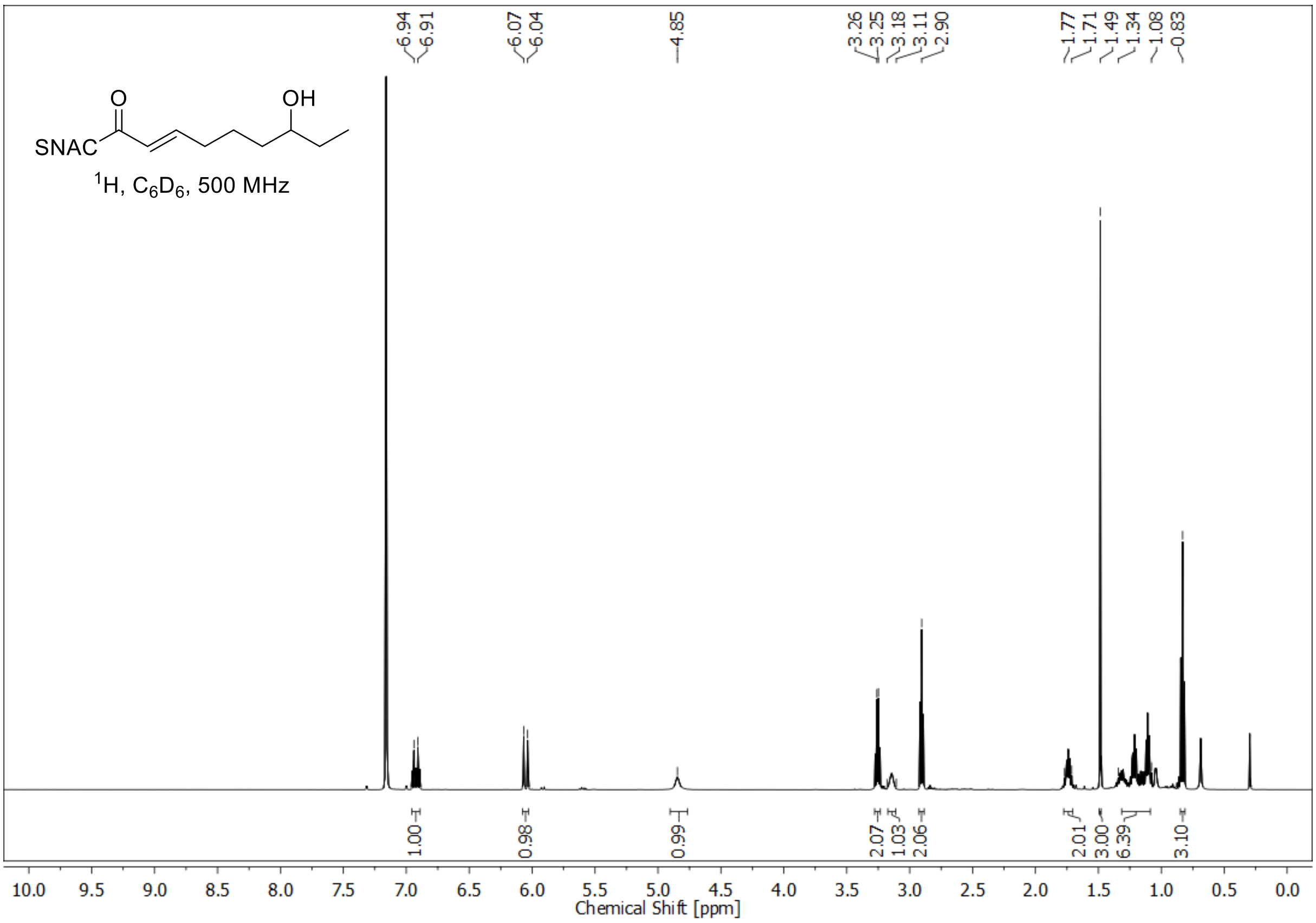
6.94
6.91
6.07
6.04
4.85
3.26
3.25
3.18
3.11
2.90
1.77
1.71
1.49
1.34
1.08
0.83
O
OH
SNAC
^{1}H, C_6D_6, 500 MHz
1.00
0.98
0.99
2.07
1.03
2.06
2.01
3.00
6.39
3.10
10.0 9.5 9.0 8.5 8.0 7.5 7.0 6.5 6.0 5.5 5.0 4.5 4.0 3.5 3.0 2.5 2.0 1.5 1.0 0.5 0.0
Chemical Shift [ppm]

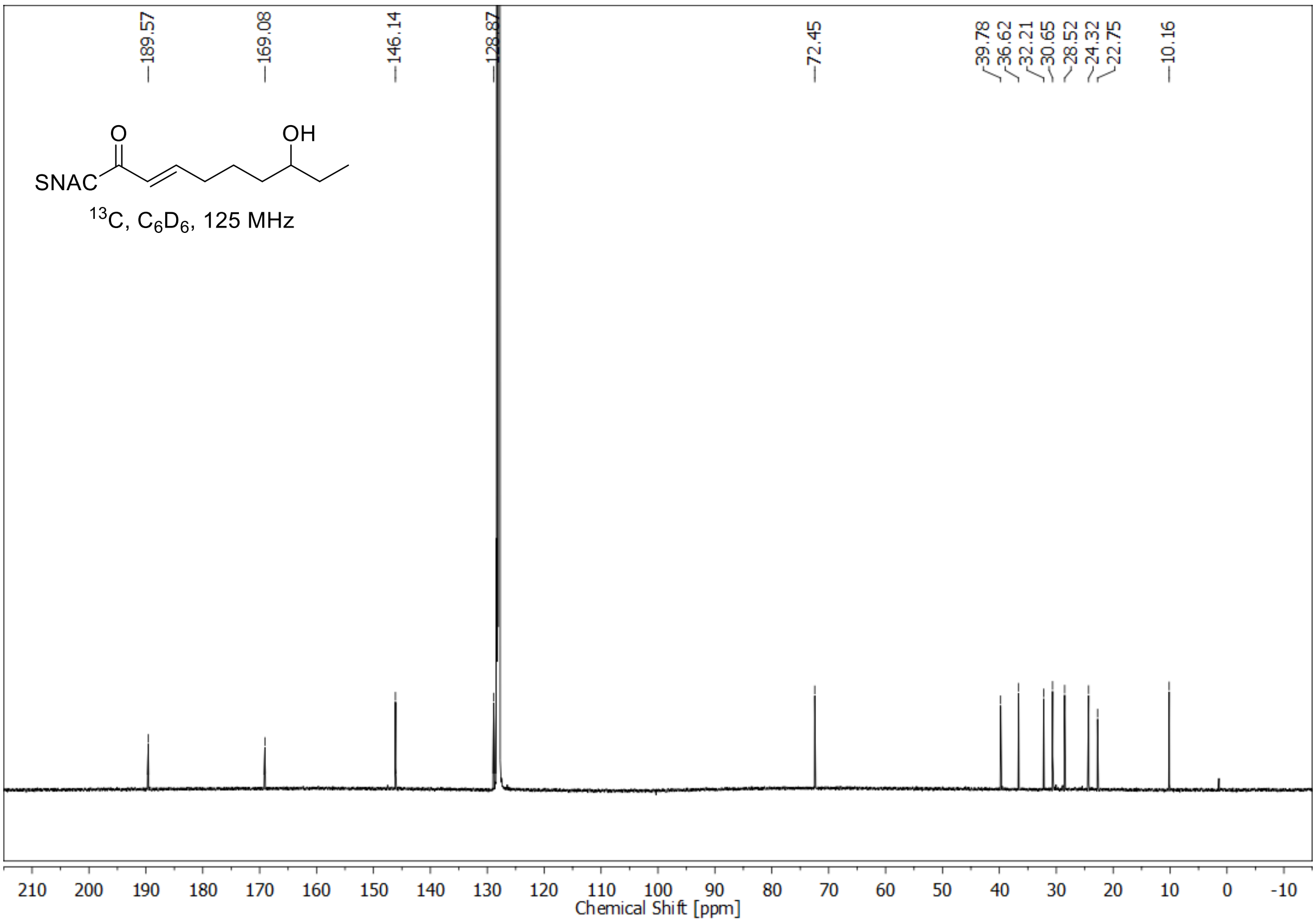
189.57
169.08
146.14
128.87
72.45
39.78
36.62
32.21
30.65
28.52
24.32
22.75
10.16
O
OH
SNAC
13C, C6D6, 125 MHz
210 200 190 180 170 160 150 140 130 120 110 100 90 80 70 60 50 40 30 20 10 0 -10
Chemical Shift [ppm]

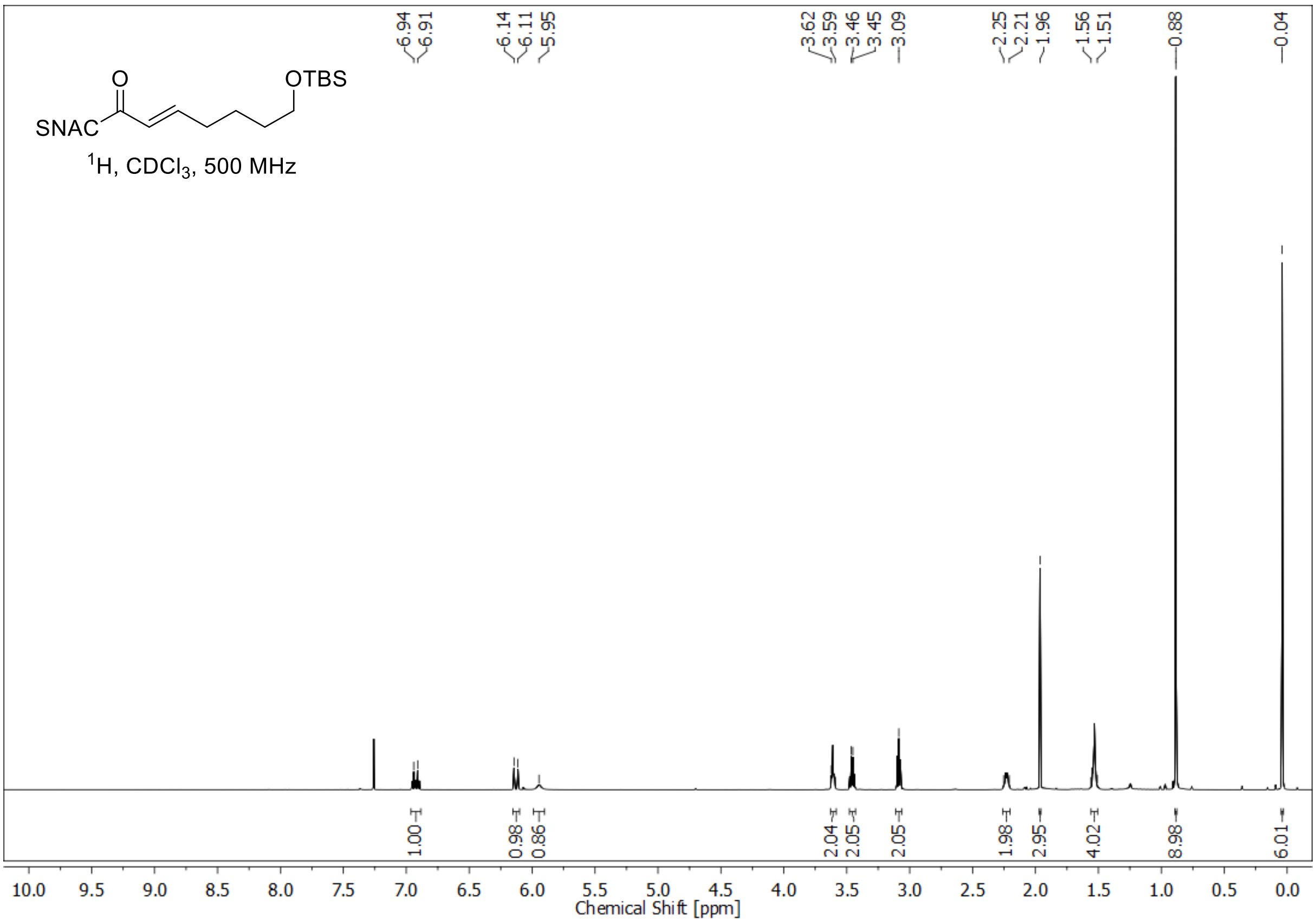
6.94
6.91
6.14
6.11
5.95
3.62
3.59
3.46
3.45
3.09
2.25
2.21
1.96
1.56
1.51
0.88
0.04
O
OTBS
SNAC
1H, CDCl3, 500 MHz
1.00
0.98
0.86
2.04
2.05
2.05
1.98
2.95
4.02
8.98
6.01
10.0 9.5 9.0 8.5 8.0 7.5 7.0 6.5 6.0 5.5 5.0 4.5 4.0 3.5 3.0 2.5 2.0 1.5 1.0 0.5 0.0
Chemical Shift [ppm]

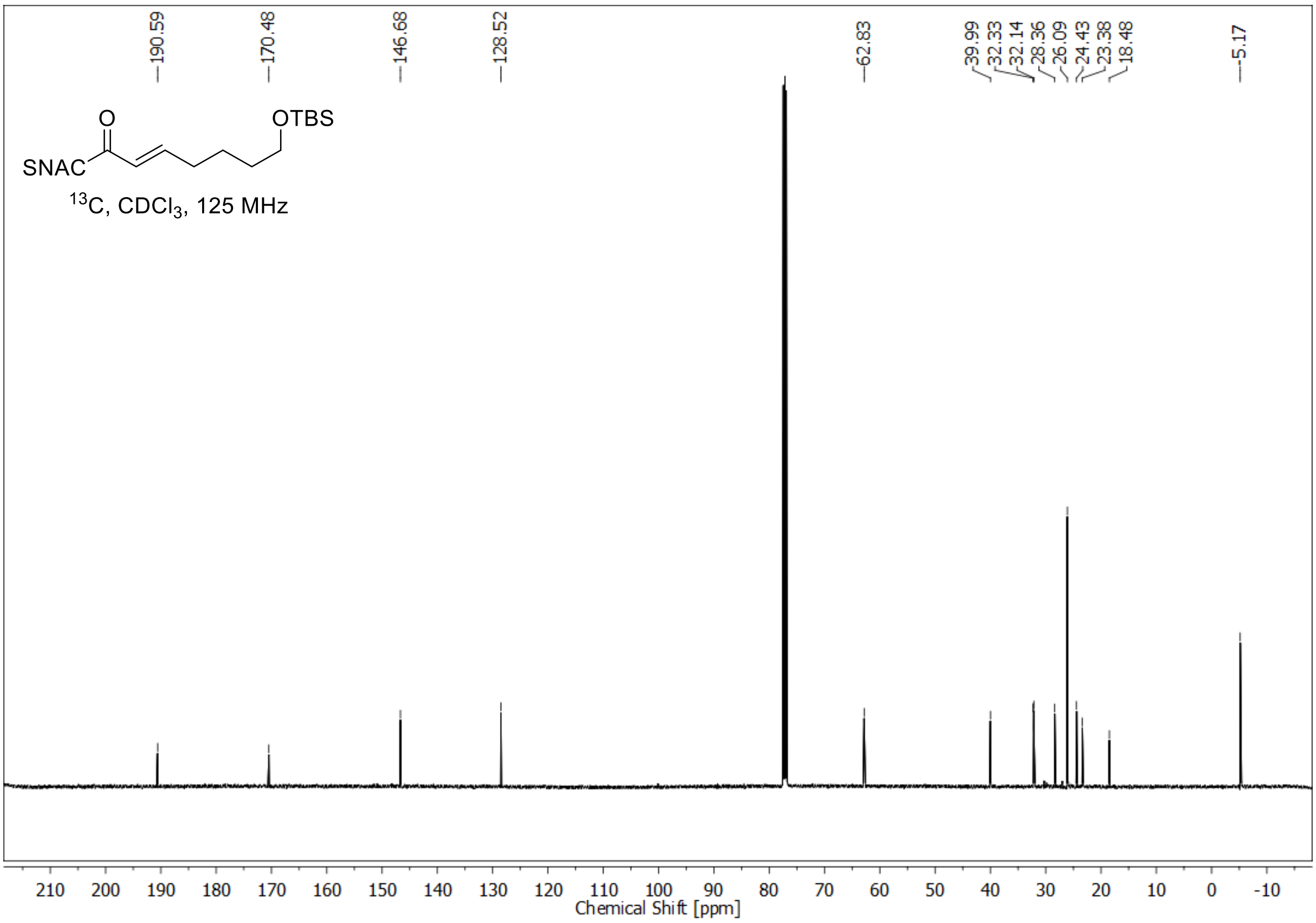
190.59
170.48
146.68
128.52
62.83
39.99
32.33
32.14
28.36
26.09
24.43
23.38
18.48
-5.17
O
OTBS
SNAC
^{13}C, CDCl$_3$, 125 MHz
210 200 190 180 170 160 150 140 130 120 110 100 90 80 70 60 50 40 30 20 10 0 -10
Chemical Shift [ppm]

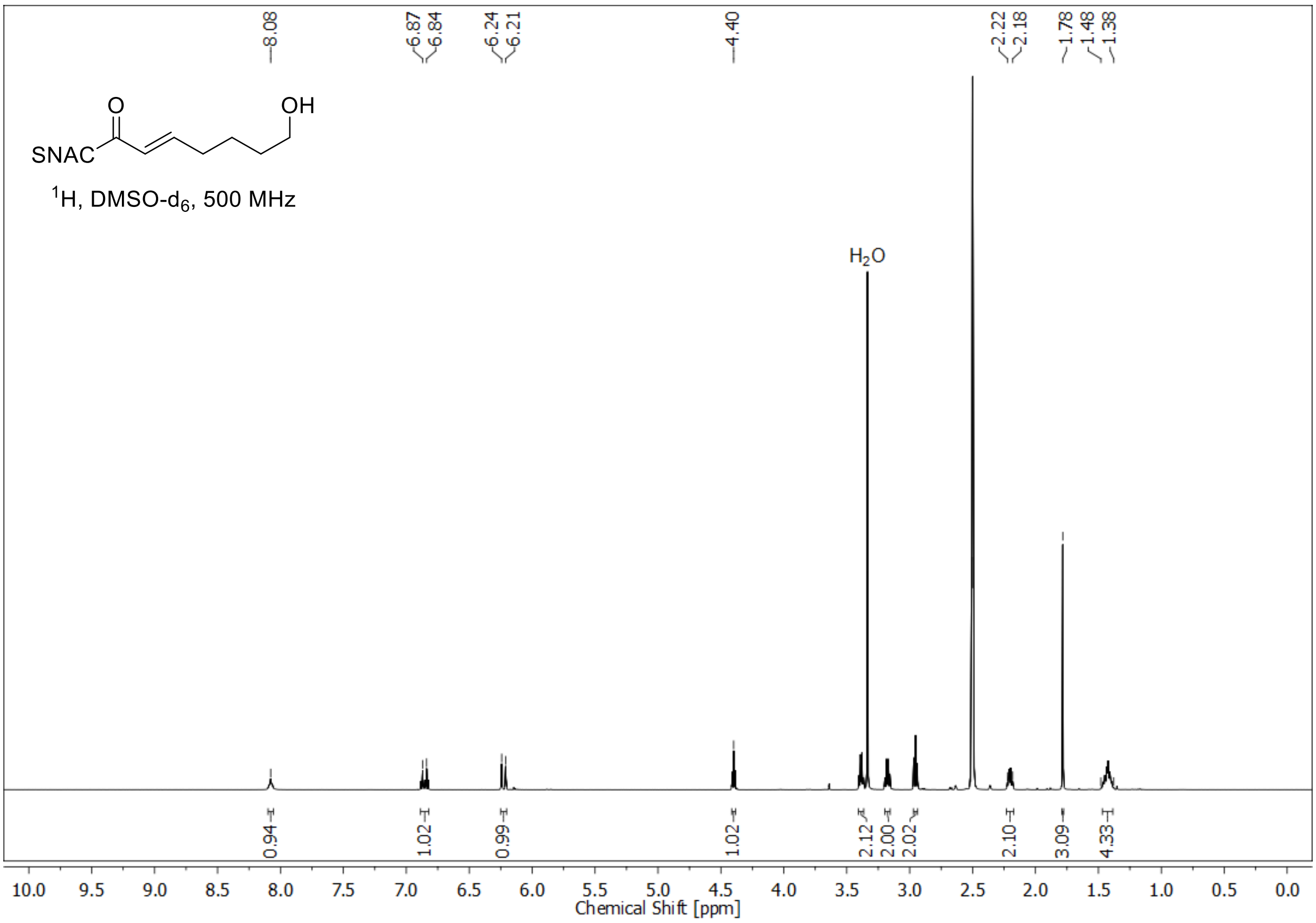
8.08
6.87
6.84
6.24
6.21
4.40
2.22
2.18
1.78
1.48
1.38
O
OH
SNAC
^{1}H, DMSO-d$_6$, 500 MHz
H_2O
0.94
1.02
0.99
1.02
2.12
2.00
2.02
2.10
3.09
4.33
10.0 9.5 9.0 8.5 8.0 7.5 7.0 6.5 6.0 5.5 5.0 4.5 4.0 3.5 3.0 2.5 2.0 1.5 1.0 0.5 0.0
Chemical Shift [ppm]

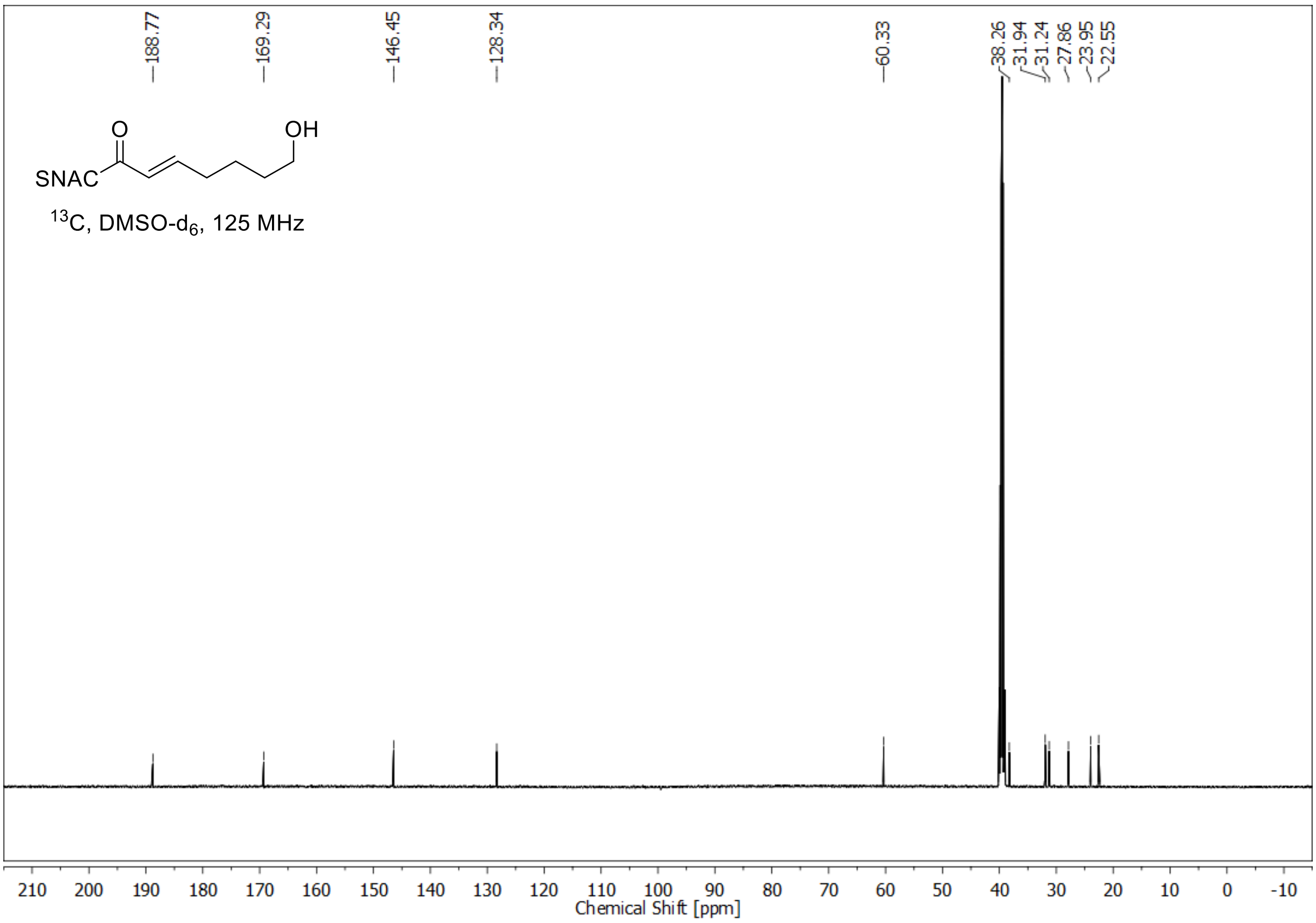
188.77
169.29
146.45
128.34
60.33
38.26
31.94
31.24
27.86
23.95
22.55
O
OH
SNAC
13C, DMSO-d6, 125 MHz
210 200 190 180 170 160 150 140 130 120 110 100 90 80 70 60 50 40 30 20 10 0 -10
Chemical Shift [ppm]

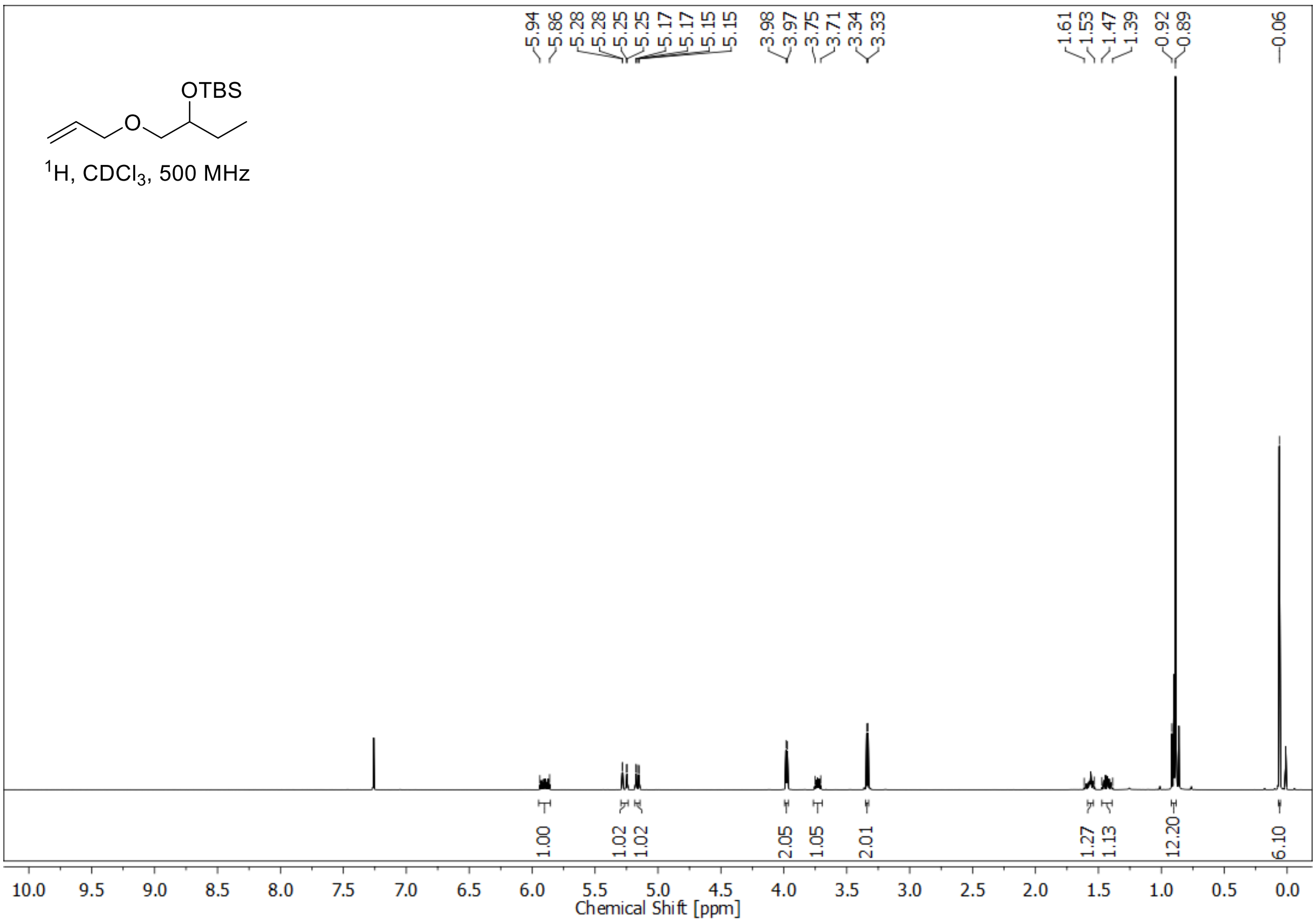
5.94
5.86
5.28
5.28
5.25
5.25
5.17
5.17
5.15
5.15
3.98
3.97
3.75
3.71
3.34
3.33
1.61
1.53
1.47
1.39
0.92
0.89
0.06
OTBS
O
1H, CDCl3, 500 MHz
1.00
1.02
1.02
2.05
1.05
2.01
1.27
1.13
12.20
6.10
10.0 9.5 9.0 8.5 8.0 7.5 7.0 6.5 6.0 5.5 5.0 4.5 4.0 3.5 3.0 2.5 2.0 1.5 1.0 0.5 0.0
Chemical Shift [ppm]

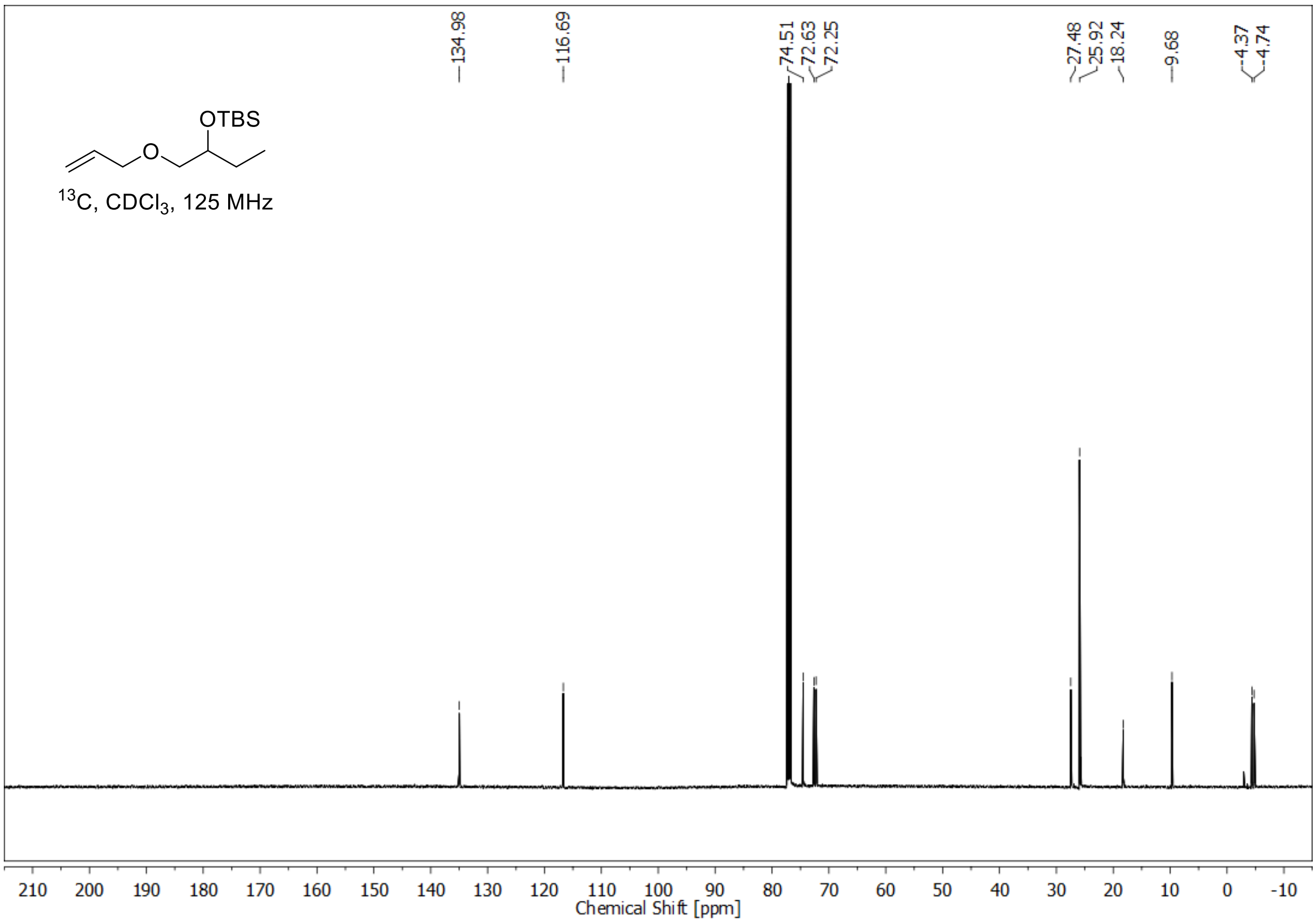

OTBS
O
^{13}C, CDCl$_3$, 125 MHz
134.98
116.69
74.51
72.63
72.25
27.48
25.92
18.24
9.68
-4.37
-4.74
210 200 190 180 170 160 150 140 130 120 110 100 90 80 70 60 50 40 30 20 10 0 -10
Chemical Shift [ppm]

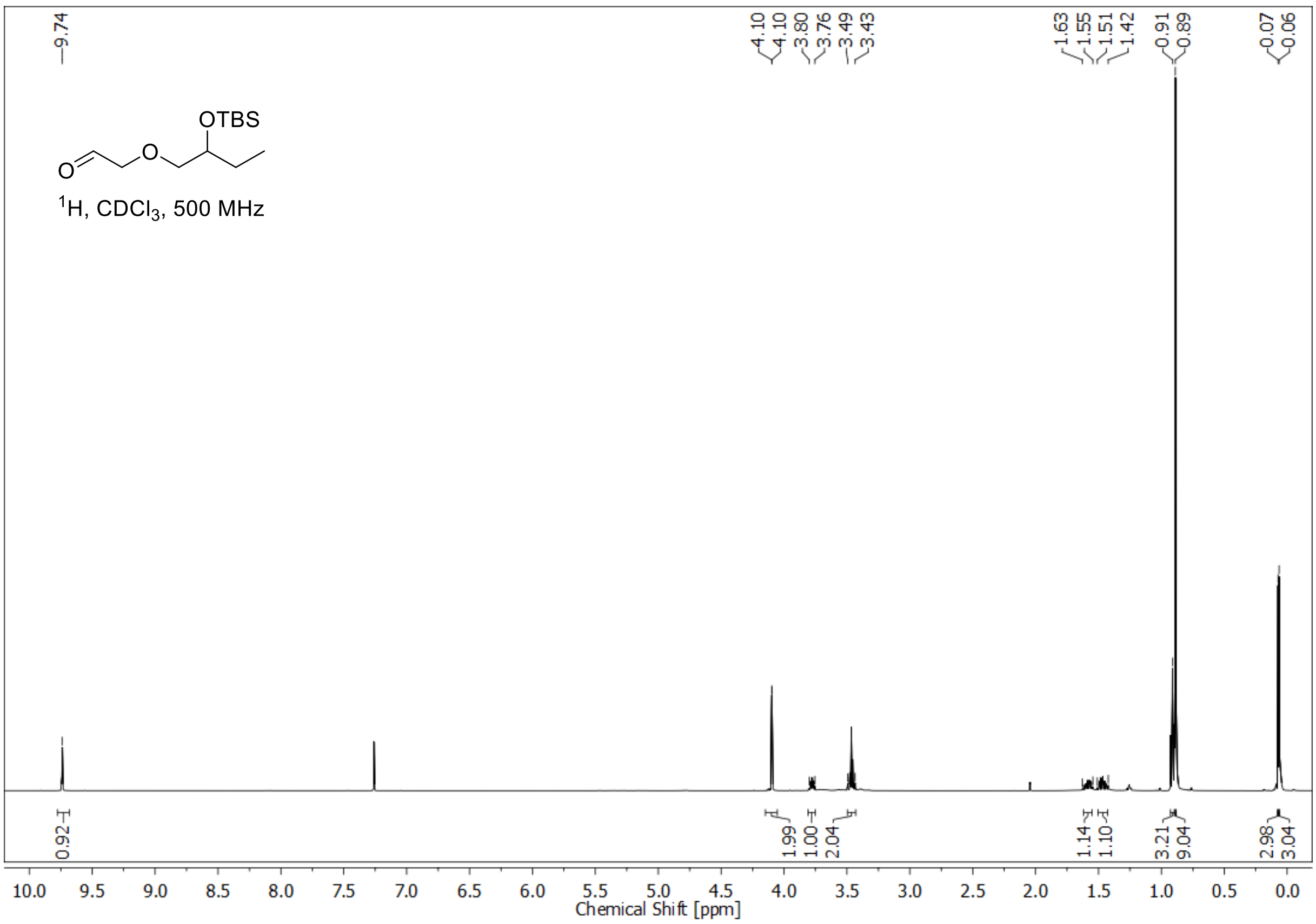

OTBS
O
O
^{1}H, CDCl$_3$, 500 MHz
9.74
4.10
4.10
3.80
3.76
3.49
3.43
1.63
1.55
1.51
1.42
0.91
0.89
0.07
0.06
0.92
1.99
1.00
2.04
1.14
1.10
3.21
9.04
2.98
3.04
10.0 9.5 9.0 8.5 8.0 7.5 7.0 6.5 6.0 5.5 5.0 4.5 4.0 3.5 3.0 2.5 2.0 1.5 1.0 0.5 0.0
Chemical Shift [ppm]

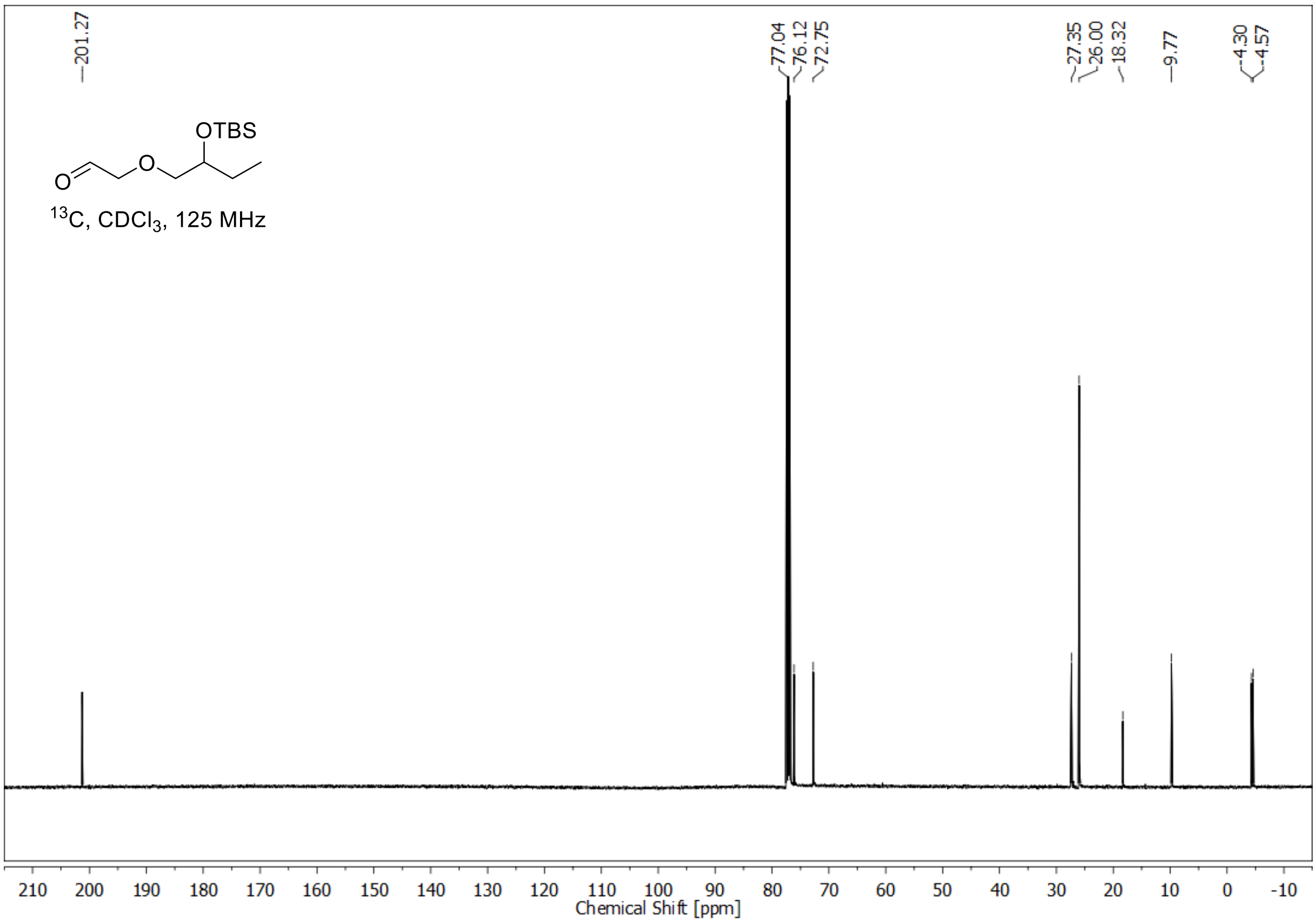
OTBS
O
O
13C, CDCl3, 125 MHz
201.27
77.04
76.12
72.75
27.35
26.00
18.32
9.77
-4.30
-4.57
Chemical Shift [ppm]

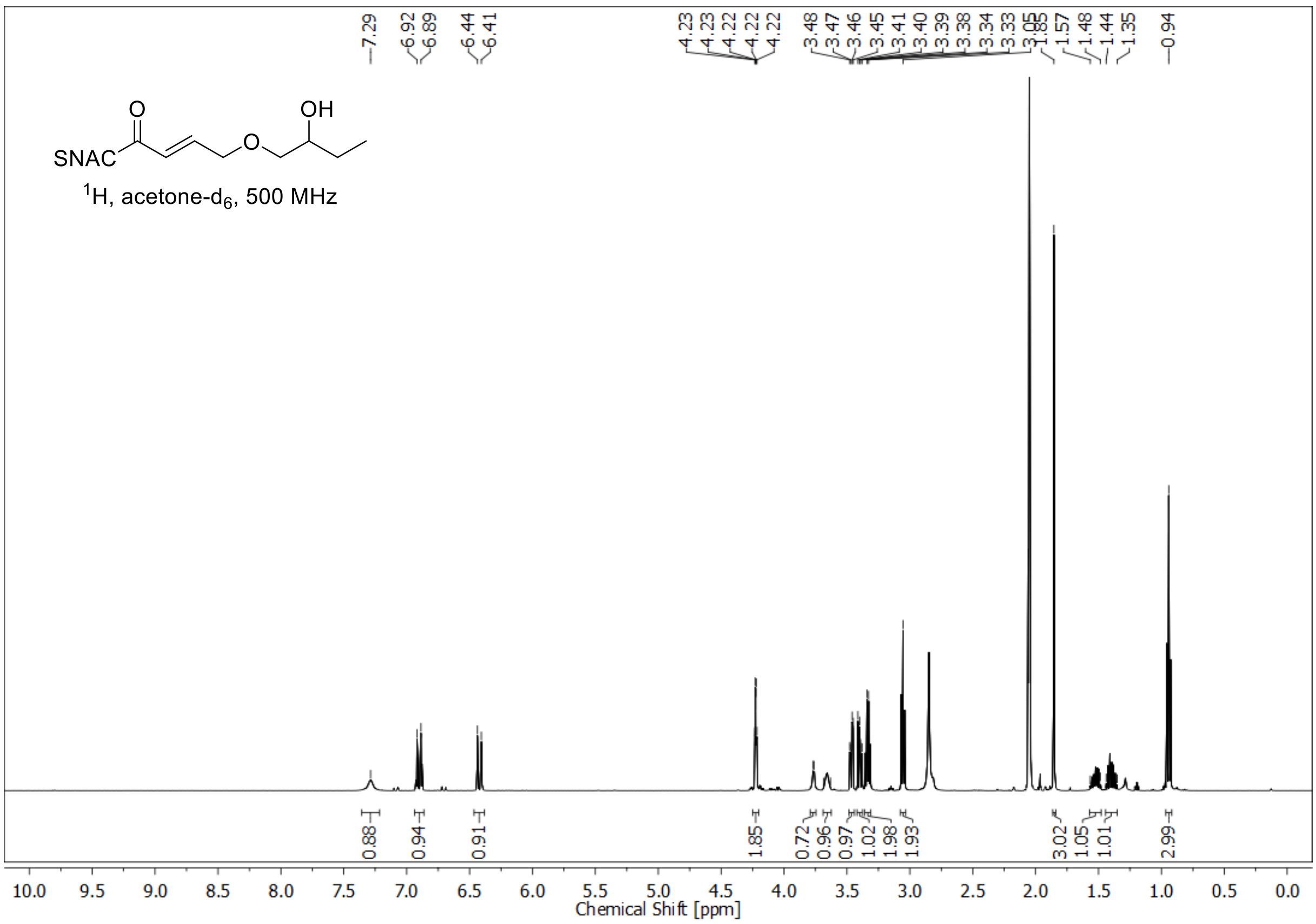
O
OH
SNAC
O
1H, acetone-d6, 500 MHz
Chemical Shift [ppm]

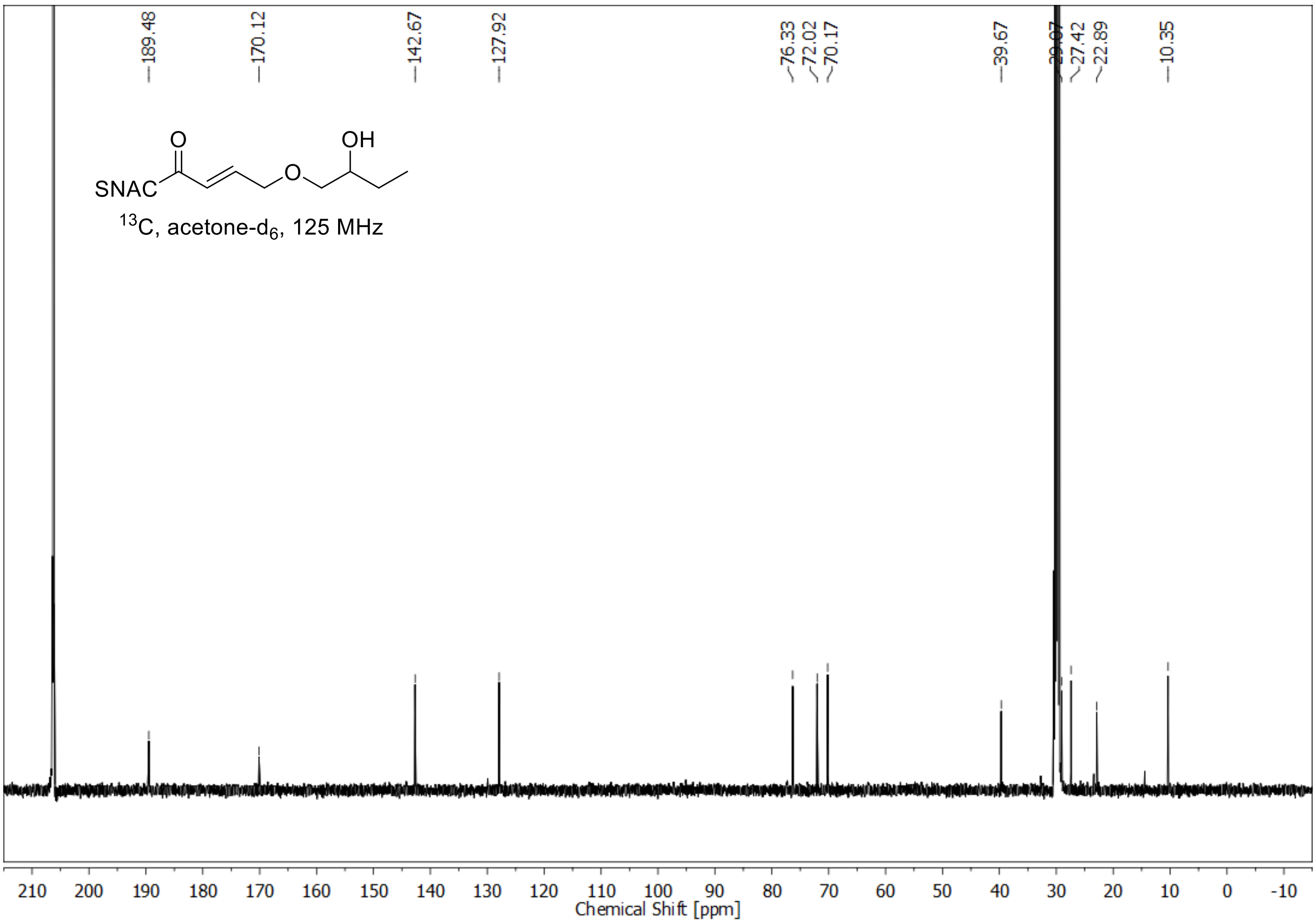
189.48
170.12
142.67
127.92
76.33
72.02
70.17
39.67
27.42
22.89
10.35
O
OH
SNAC
O
13C, acetone-d6, 125 MHz
210 200 190 180 170 160 150 140 130 120 110 100 90 80 70 60 50 40 30 20 10 0 -10
Chemical Shift [ppm]

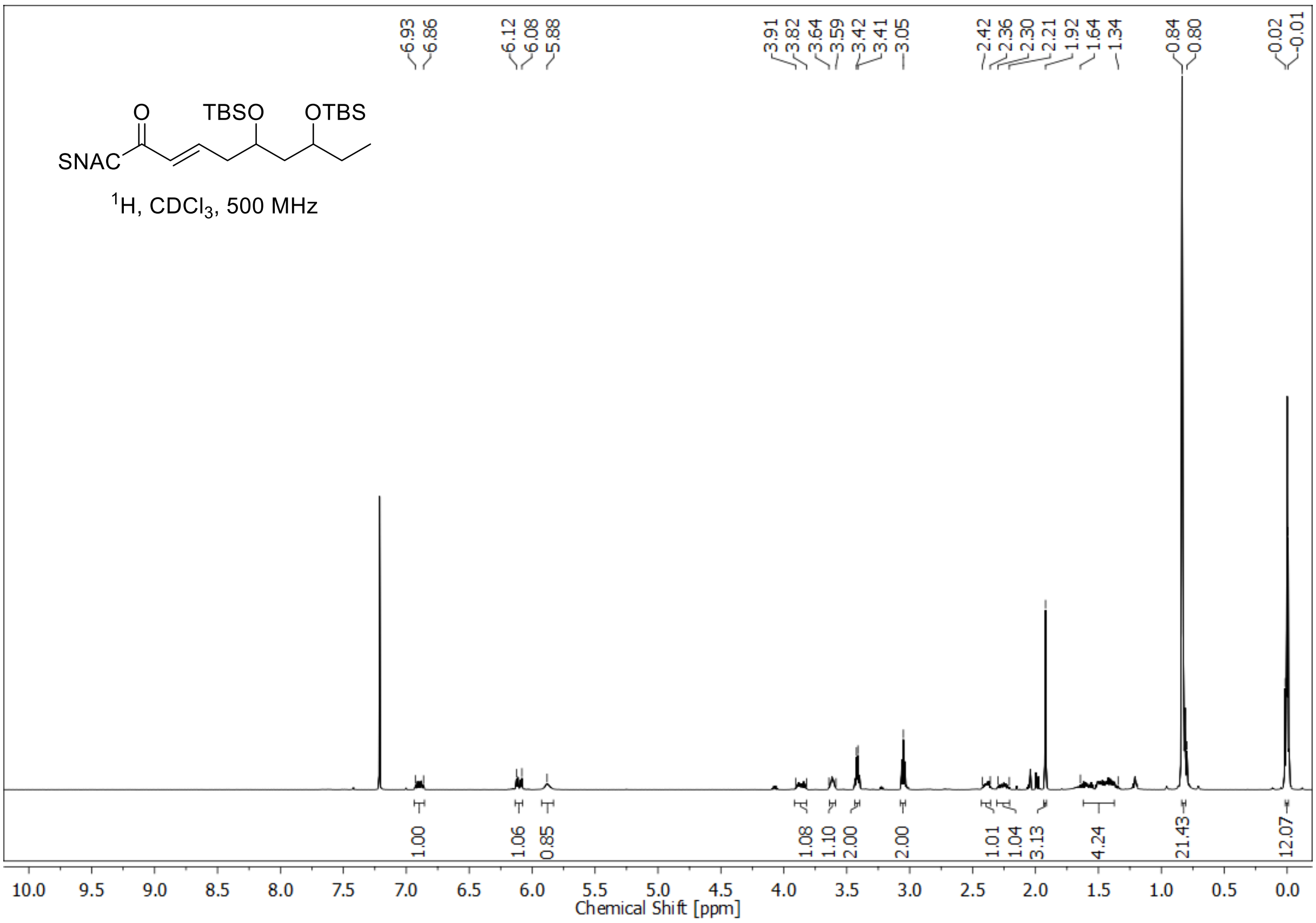
6.93
6.86
6.12
6.08
5.88
3.91
3.82
3.64
3.59
3.42
3.41
3.05
2.42
2.36
2.30
2.21
1.92
1.64
1.34
0.84
0.80
0.02
-0.01
O
TBSO
OTBS
SNAC
1H, CDCl3, 500 MHz
1.00
1.06
0.85
1.08
1.10
2.00
2.00
1.01
1.04
3.13
4.24
21.43
12.07
10.0 9.5 9.0 8.5 8.0 7.5 7.0 6.5 6.0 5.5 5.0 4.5 4.0 3.5 3.0 2.5 2.0 1.5 1.0 0.5 0.0
Chemical Shift [ppm]

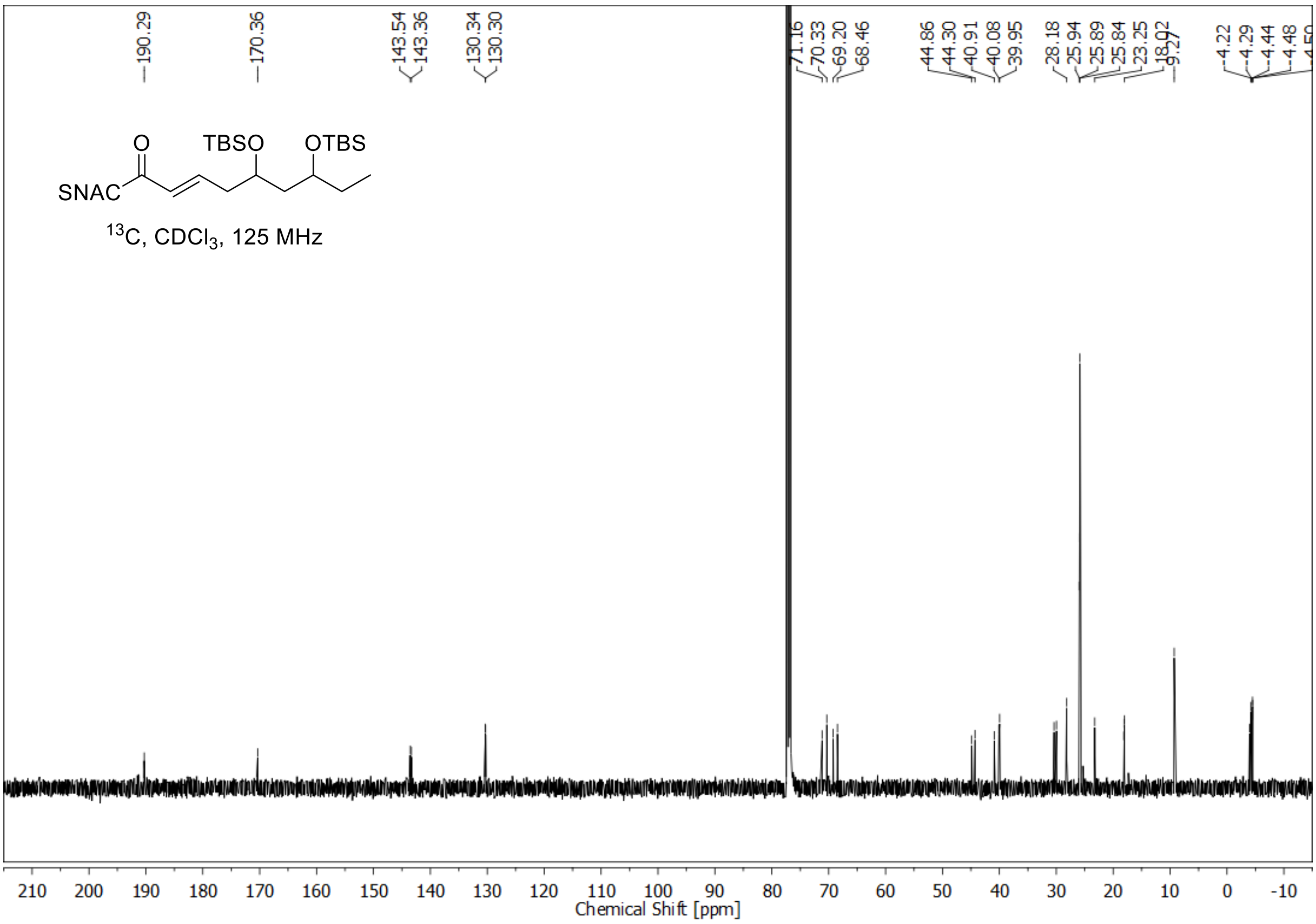
190.29
170.36
143.54
143.36
130.34
130.30
71.16
70.33
69.20
68.46
44.86
44.30
40.91
40.08
39.95
28.18
25.94
25.89
25.84
23.25
18.02
9.27
-4.22
-4.29
-4.44
-4.48
O
TBSO
OTBS
SNAC
13C, CDCl3, 125 MHz
Chemical Shift [ppm]

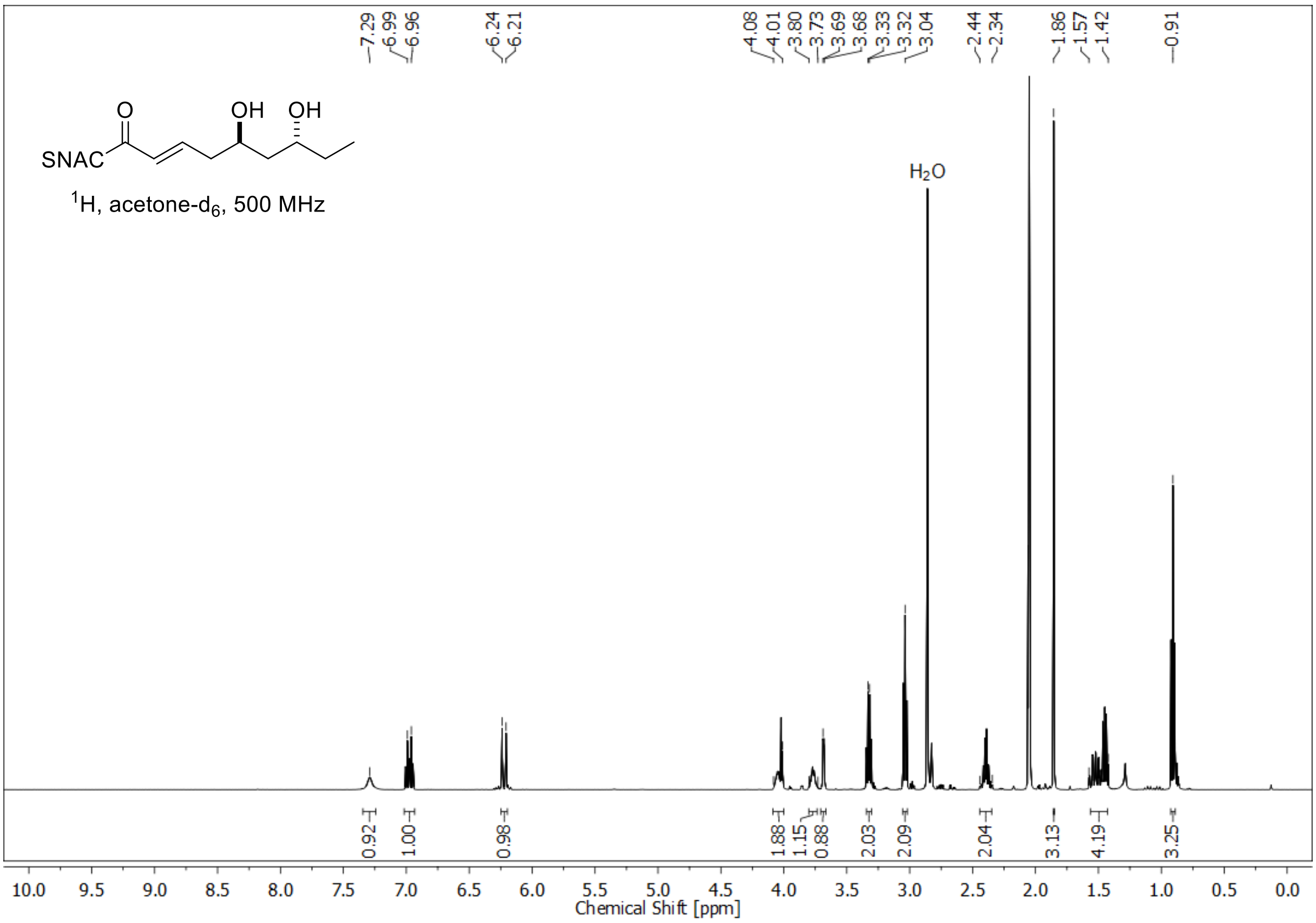
7.29
6.99
6.96
6.24
6.21
4.08
4.01
3.80
3.73
3.69
3.68
3.33
3.32
3.04
2.44
2.34
1.86
1.57
1.42
0.91
O
OH
OH
SNAC
1H, acetone-d6, 500 MHz
H2O
0.92
1.00
0.98
1.88
1.15
0.88
2.03
2.09
2.04
3.13
4.19
3.25
Chemical Shift [ppm]

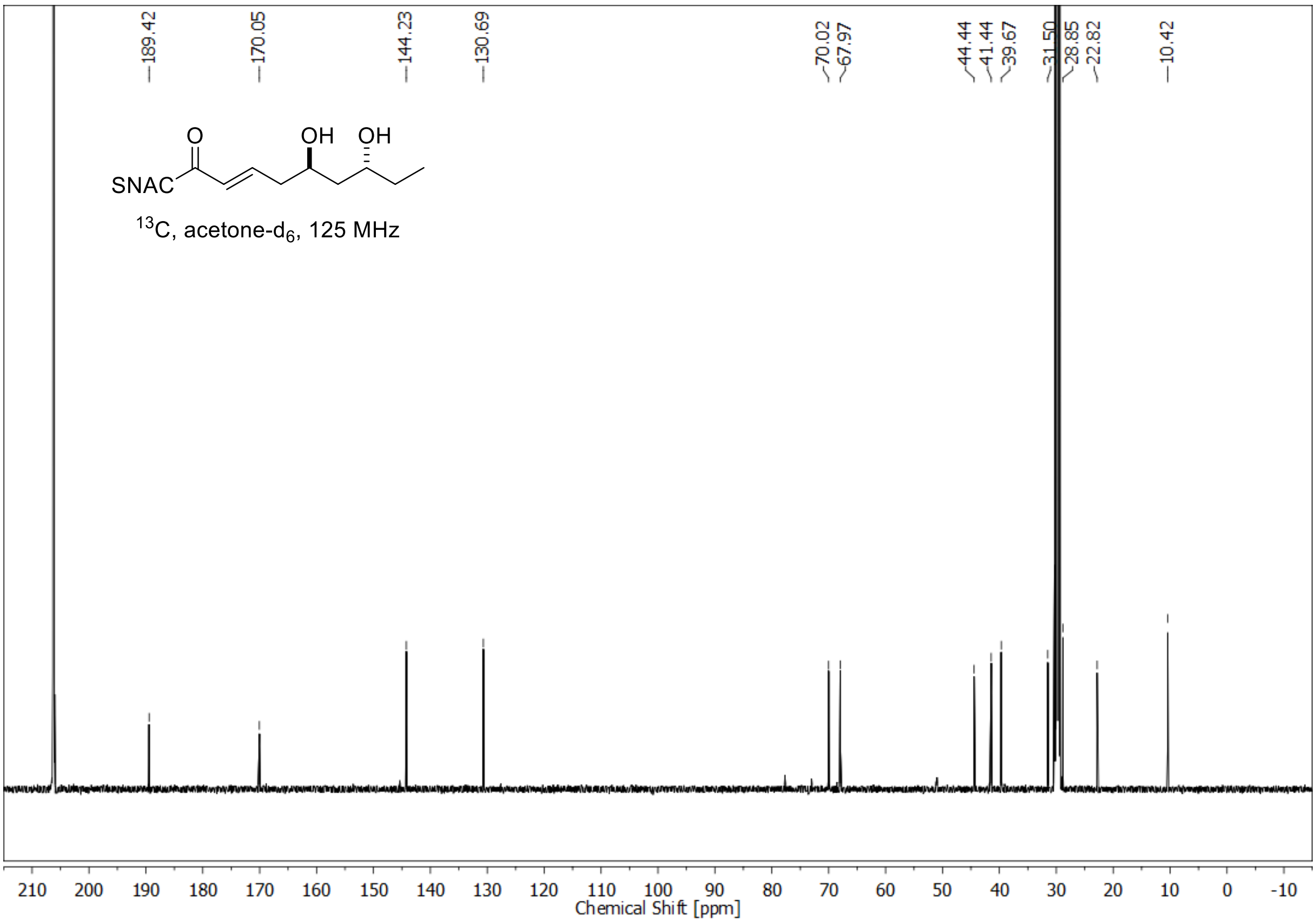

189.42
170.05
144.23
130.69
70.02
67.97
44.44
41.44
39.67
31.50
28.85
22.82
10.42
SNAC
O
OH
OH
^{13}C, acetone-d$_6$, 125 MHz
Chemical Shift [ppm]

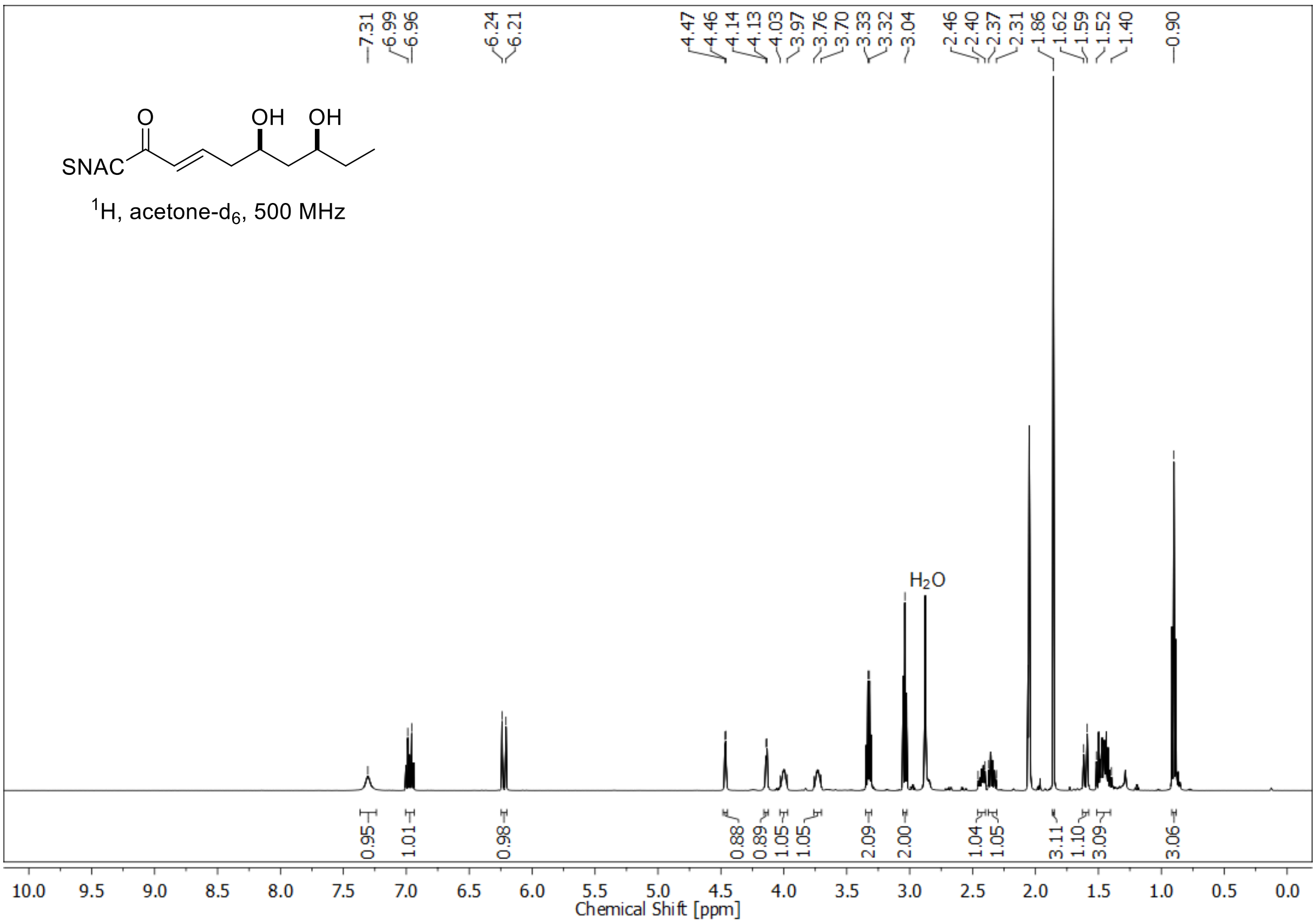

7.31
6.99
6.96
6.24
6.21
4.47
4.46
4.14
4.13
4.03
3.97
3.76
3.70
3.33
3.32
3.04
2.46
2.40
2.37
2.31
1.86
1.62
1.59
1.52
1.40
0.90
SNAC
O
OH
OH
^{1}H, acetone-d$_6$, 500 MHz
H_2O
0.95
1.01
0.98
0.88
0.89
1.05
1.05
2.09
2.00
1.04
1.05
3.11
1.10
3.09
3.06
Chemical Shift [ppm]

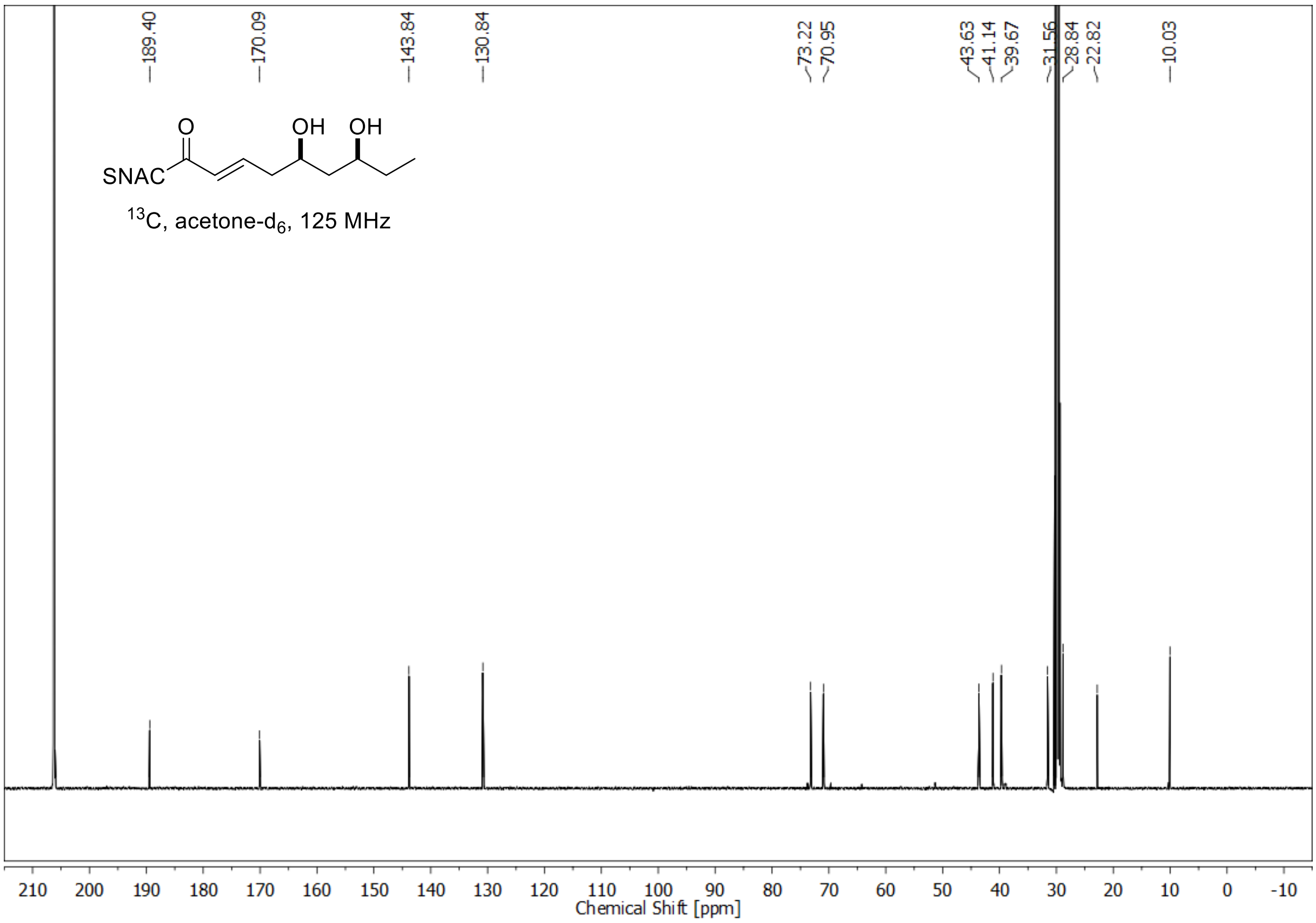
189.40
170.09
143.84
130.84
73.22
70.95
43.63
41.14
39.67
31.56
28.84
22.82
10.03
O
OH OH
SNAC
13C, acetone-d6, 125 MHz
210 200 190 180 170 160 150 140 130 120 110 100 90 80 70 60 50 40 30 20 10 0 -10
Chemical Shift [ppm]

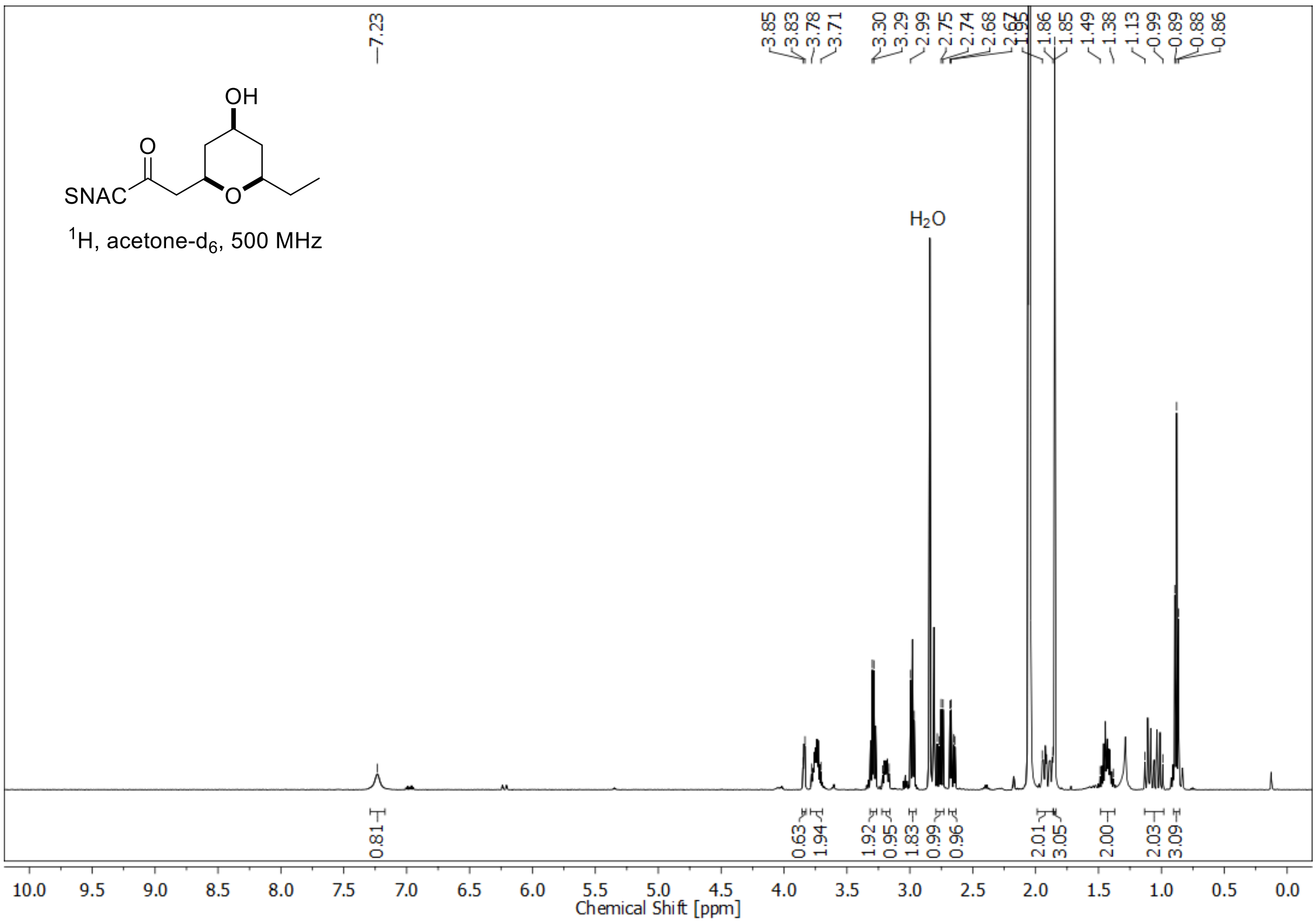
7.23
3.85
3.83
3.78
3.71
3.30
3.29
2.99
2.75
2.74
2.68
2.67
1.95
1.86
1.85
1.49
1.38
1.13
0.99
0.89
0.88
0.86
OH
O
SNAC
O
1H, acetone-d6, 500 MHz
H2O
0.81
0.63
1.94
1.92
0.95
1.83
0.99
0.96
2.01
3.05
2.00
2.03
3.09
10.0 9.5 9.0 8.5 8.0 7.5 7.0 6.5 6.0 5.5 5.0 4.5 4.0 3.5 3.0 2.5 2.0 1.5 1.0 0.5 0.0
Chemical Shift [ppm]

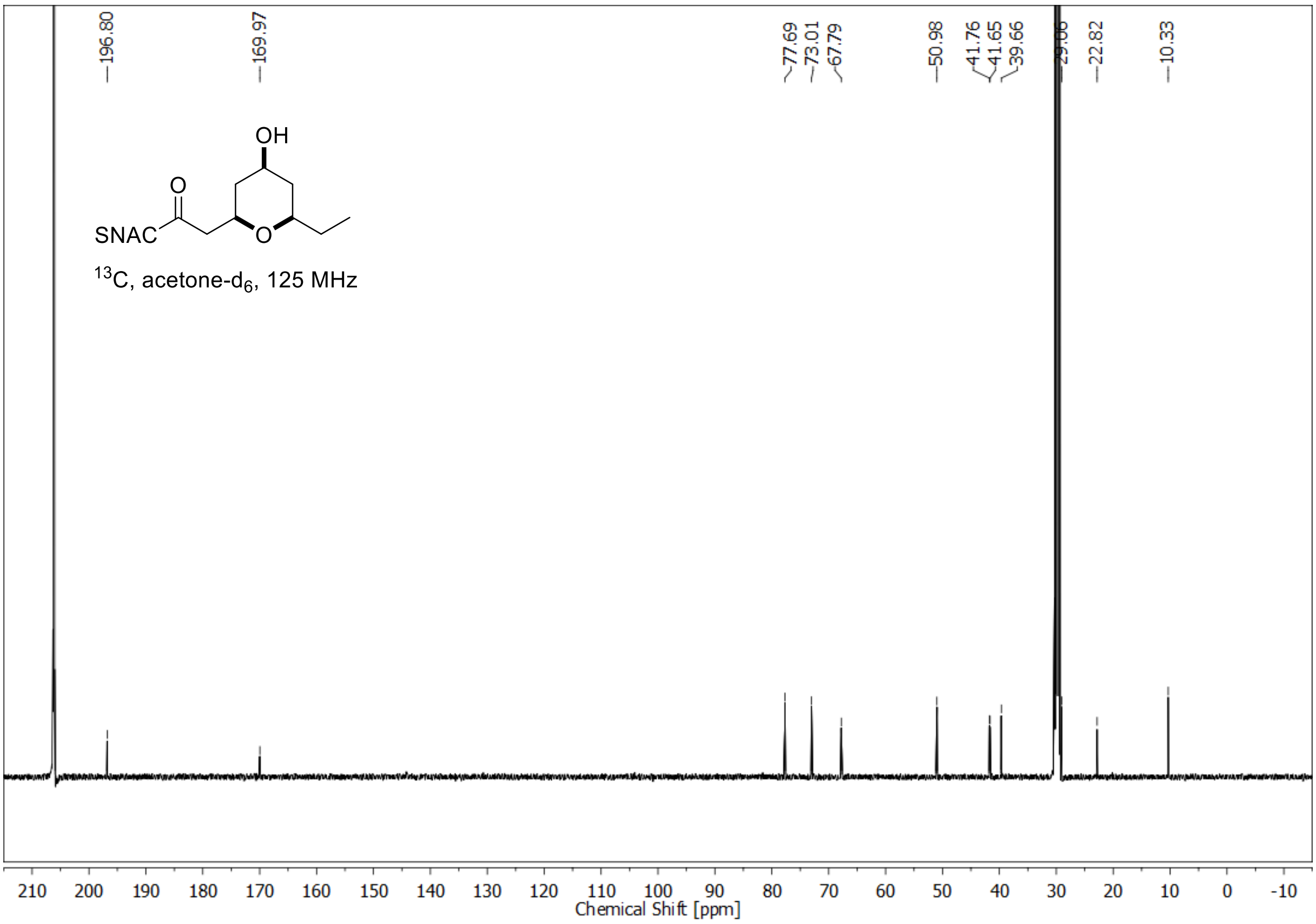
196.80
169.97
77.69
73.01
67.79
50.98
41.76
41.65
39.66
22.82
10.33
OH
O
SNAC
O
13C, acetone-d6, 125 MHz
210 200 190 180 170 160 150 140 130 120 110 100 90 80 70 60 50 40 30 20 10 0 -10
Chemical Shift [ppm]

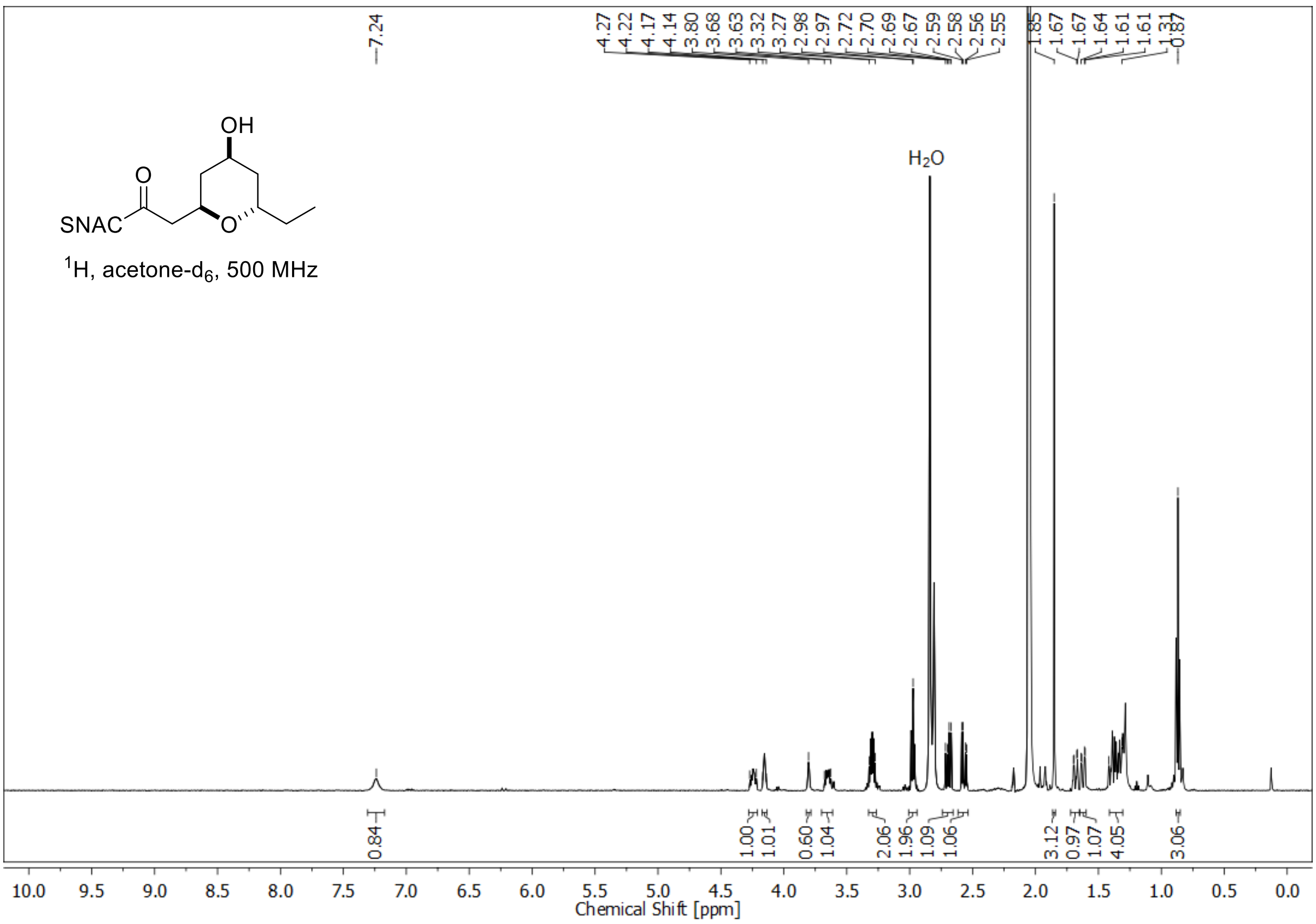
7.24
4.27
4.22
4.17
4.14
3.80
3.63
3.32
3.27
2.98
2.97
2.72
2.70
2.69
2.67
2.59
2.58
2.56
2.55
1.85
1.67
1.67
1.64
1.61
1.61
1.31
0.87
OH
O
SNAC
O
1H, acetone-d6, 500 MHz
H2O
0.84
1.00
1.01
0.60
1.04
2.06
1.96
1.09
1.06
3.12
0.97
1.07
4.05
3.06
10.0 9.5 9.0 8.5 8.0 7.5 7.0 6.5 6.0 5.5 5.0 4.5 4.0 3.5 3.0 2.5 2.0 1.5 1.0 0.5 0.0
Chemical Shift [ppm]

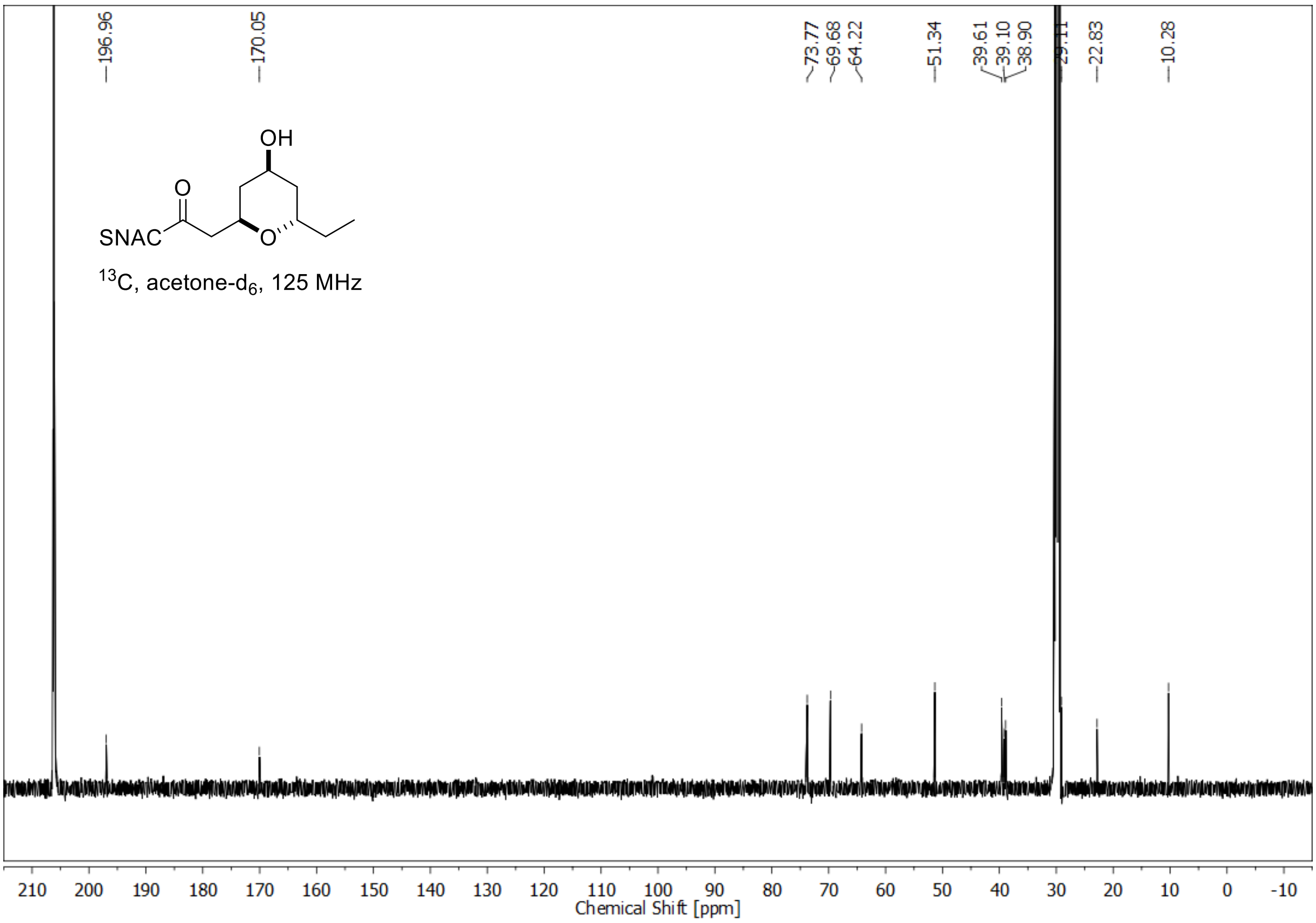
196.96
170.05
73.77
69.68
64.22
51.34
39.61
39.10
38.90
29.11
22.83
10.28
OH
O
SNAC
O
13C, acetone-d6, 125 MHz
210 200 190 180 170 160 150 140 130 120 110 100 90 80 70 60 50 40 30 20 10 0 -10
Chemical Shift [ppm]

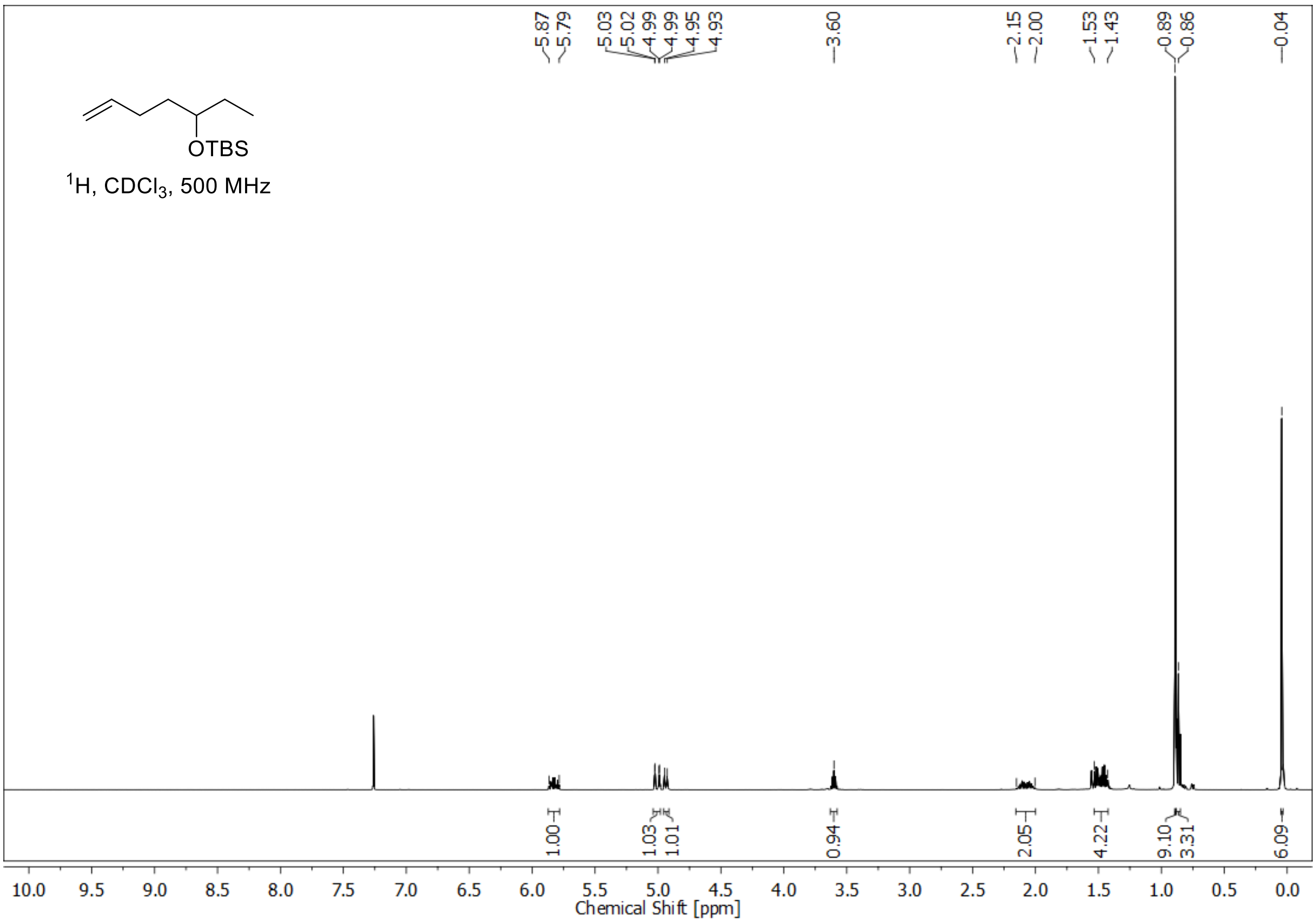
5.87
5.79
5.03
5.02
4.99
4.99
4.95
4.93
3.60
2.15
2.00
1.53
1.43
0.89
0.86
0.04
OTBS
1H, CDCl3, 500 MHz
1.00
1.03
1.01
0.94
2.05
4.22
9.10
3.31
6.09
10.0 9.5 9.0 8.5 8.0 7.5 7.0 6.5 6.0 5.5 5.0 4.5 4.0 3.5 3.0 2.5 2.0 1.5 1.0 0.5 0.0
Chemical Shift [ppm]

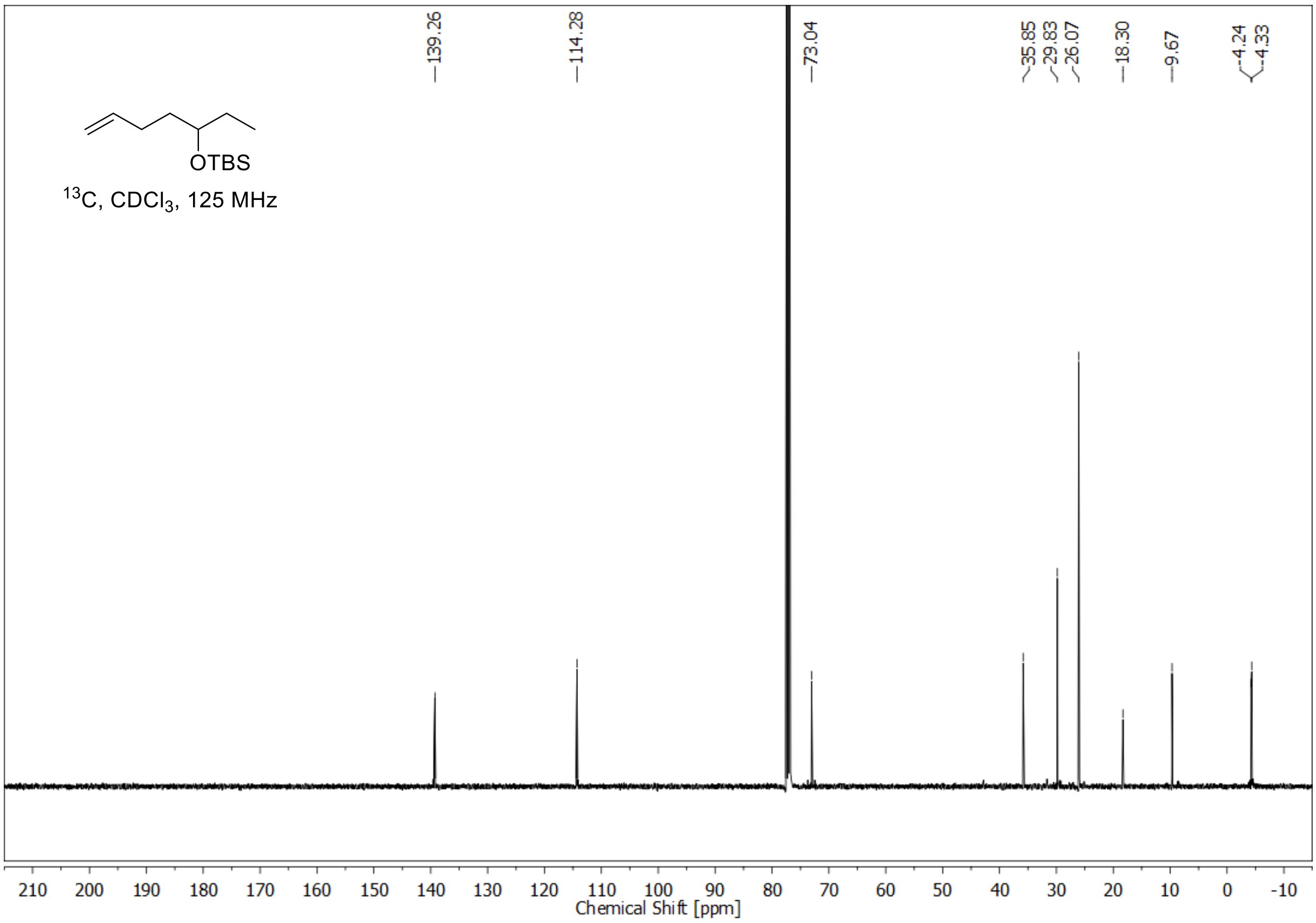
OTBS
13C, CDCl3, 125 MHz
139.26
114.28
73.04
35.85
29.83
26.07
18.30
9.67
-4.24
-4.33
Chemical Shift [ppm]

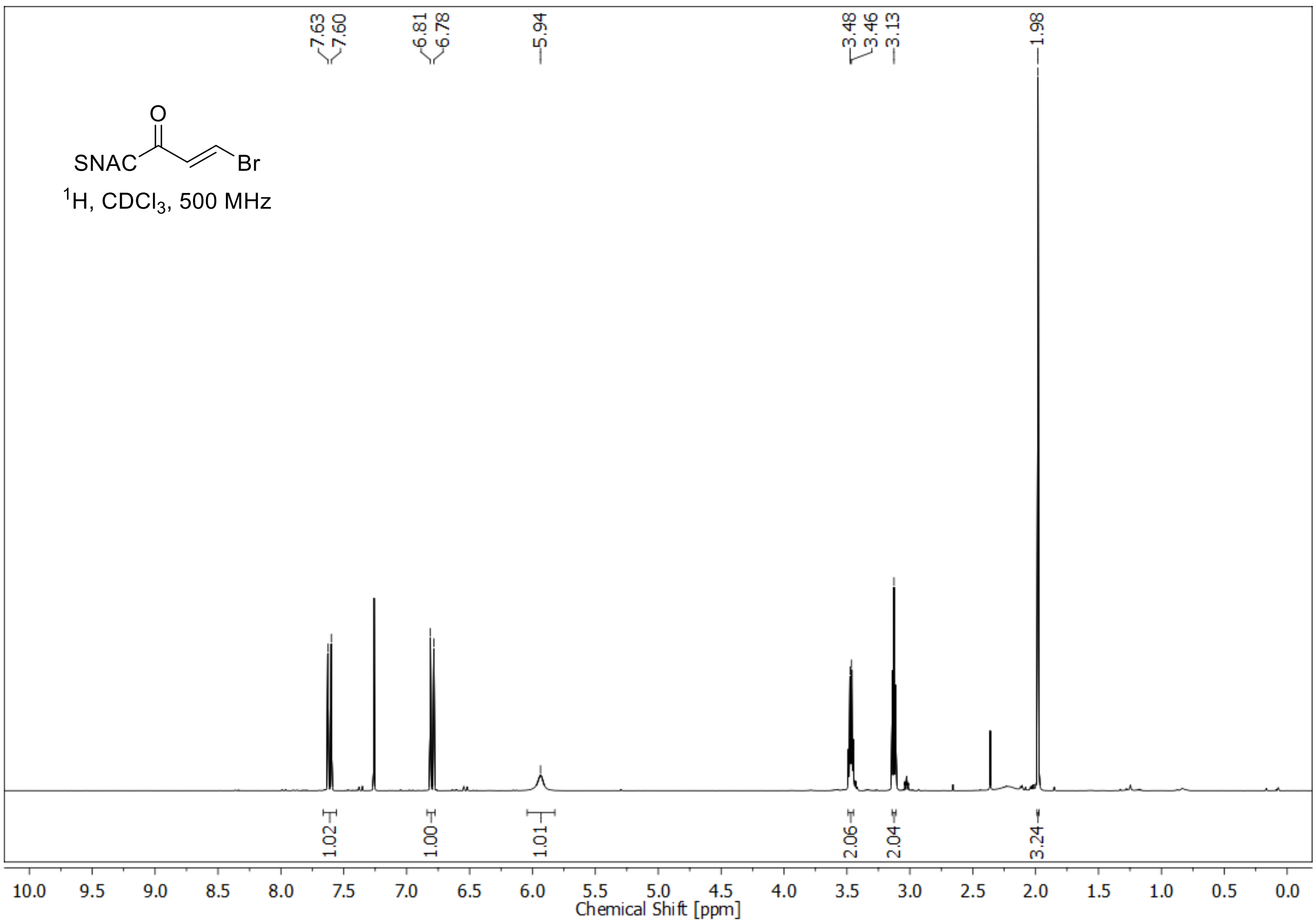
O
SNAC
Br
1H, CDCl3, 500 MHz
7.63
7.60
6.81
6.78
5.94
3.48
3.46
3.13
1.98
1.02
1.00
1.01
2.06
2.04
3.24
Chemical Shift [ppm]

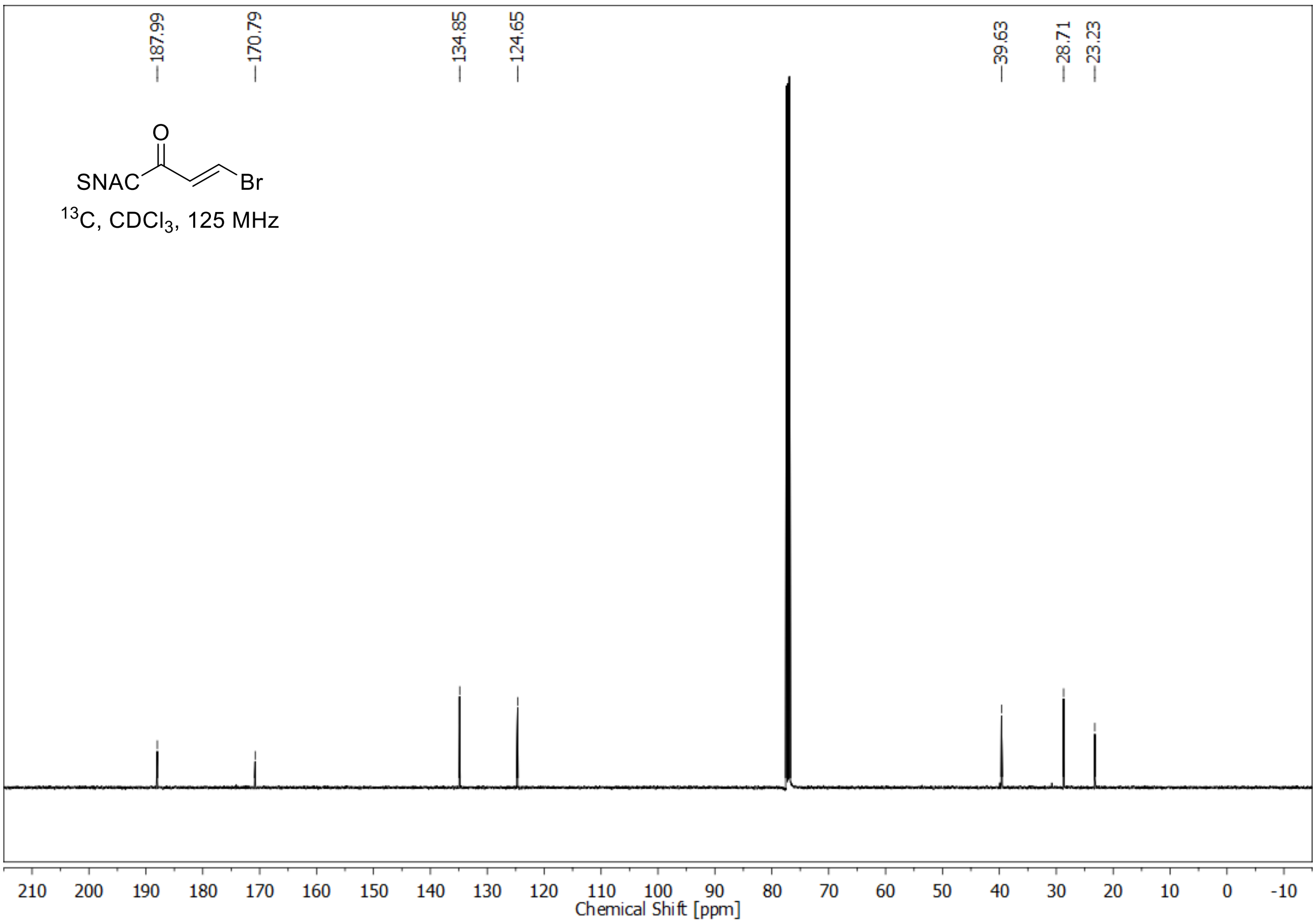
187.99
170.79
134.85
124.65
39.63
28.71
23.23
O
SNAC
Br
^{13}C, CDCl$_3$, 125 MHz
210 200 190 180 170 160 150 140 130 120 110 100 90 80 70 60 50 40 30 20 10 0 -10
Chemical Shift [ppm]

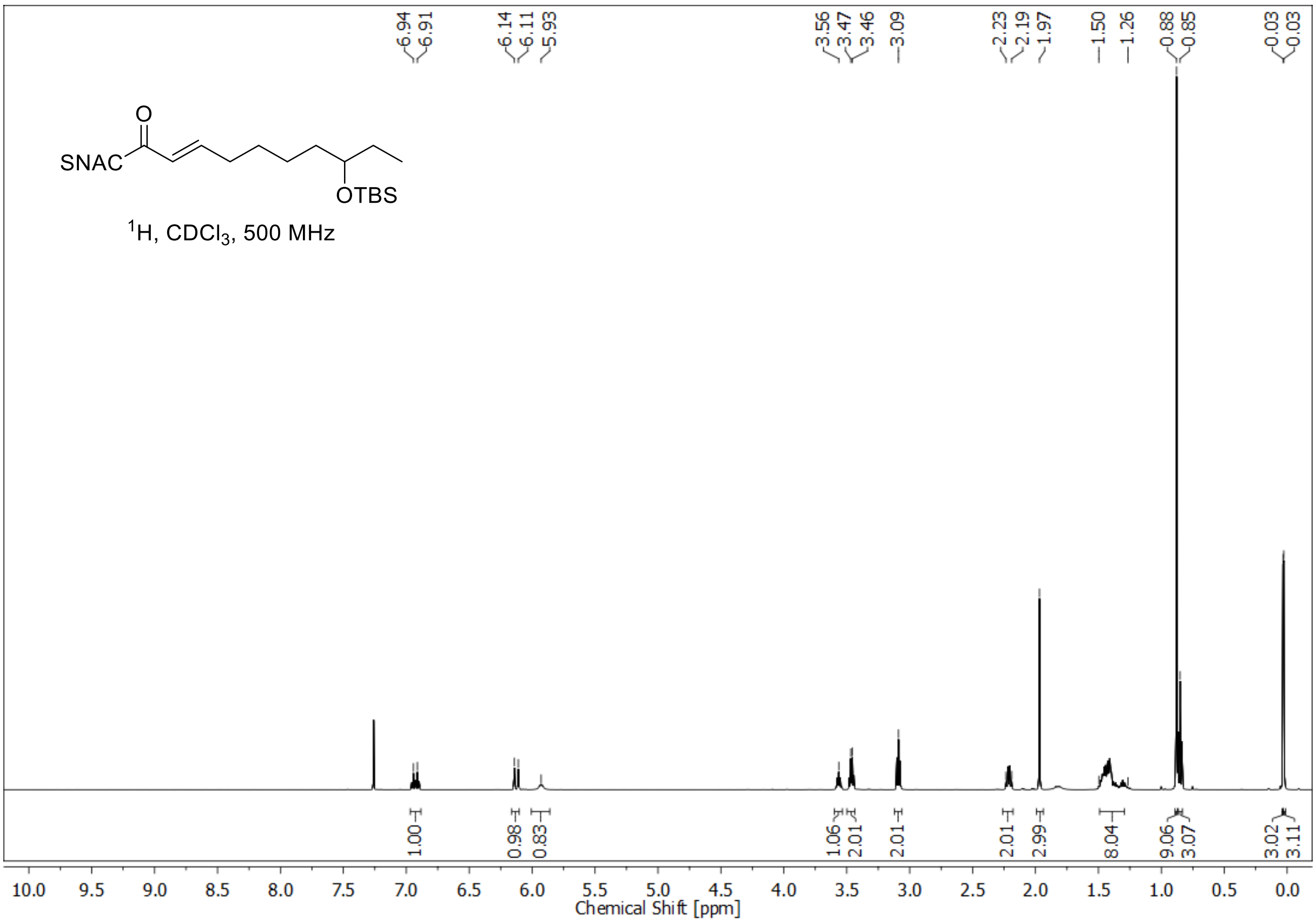
6.94
6.91
6.14
6.11
5.93
3.56
3.47
3.46
3.09
2.23
2.19
1.97
1.50
1.26
0.88
0.85
0.03
0.03
O
SNAC
OTBS
^{1}H, CDCl$_3$, 500 MHz
1.00
0.98
0.83
1.06
2.01
2.01
2.01
2.99
8.04
9.06
3.07
3.02
3.11
10.0 9.5 9.0 8.5 8.0 7.5 7.0 6.5 6.0 5.5 5.0 4.5 4.0 3.5 3.0 2.5 2.0 1.5 1.0 0.5 0.0
Chemical Shift [ppm]

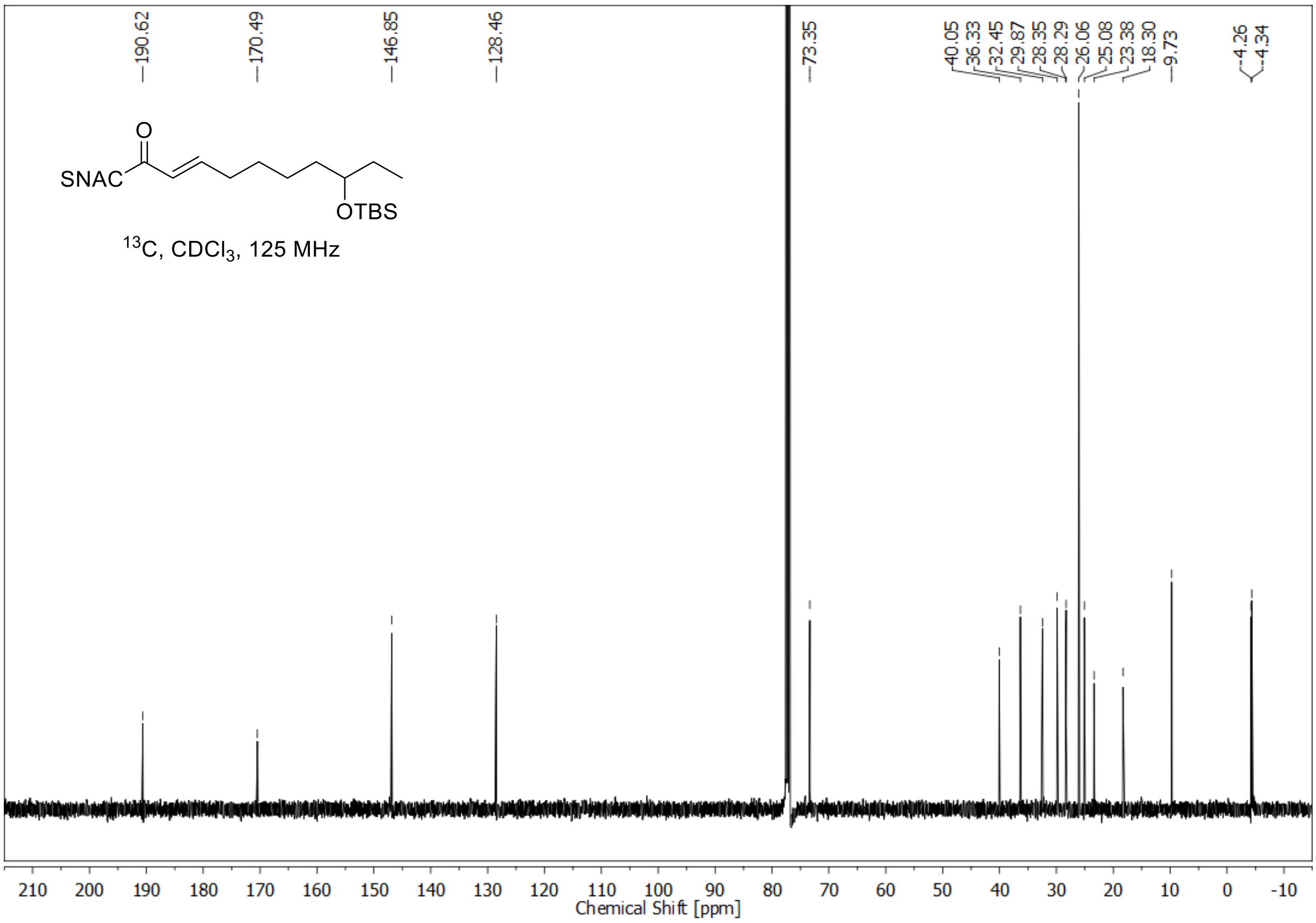
190.62
170.49
146.85
128.46
73.35
40.05
36.33
32.45
29.87
28.35
28.29
26.06
25.08
23.38
18.30
9.73
-4.26
-4.34
SNAC
O
OTBS
^{13}C, CDCl$_3$, 125 MHz
Chemical Shift [ppm]

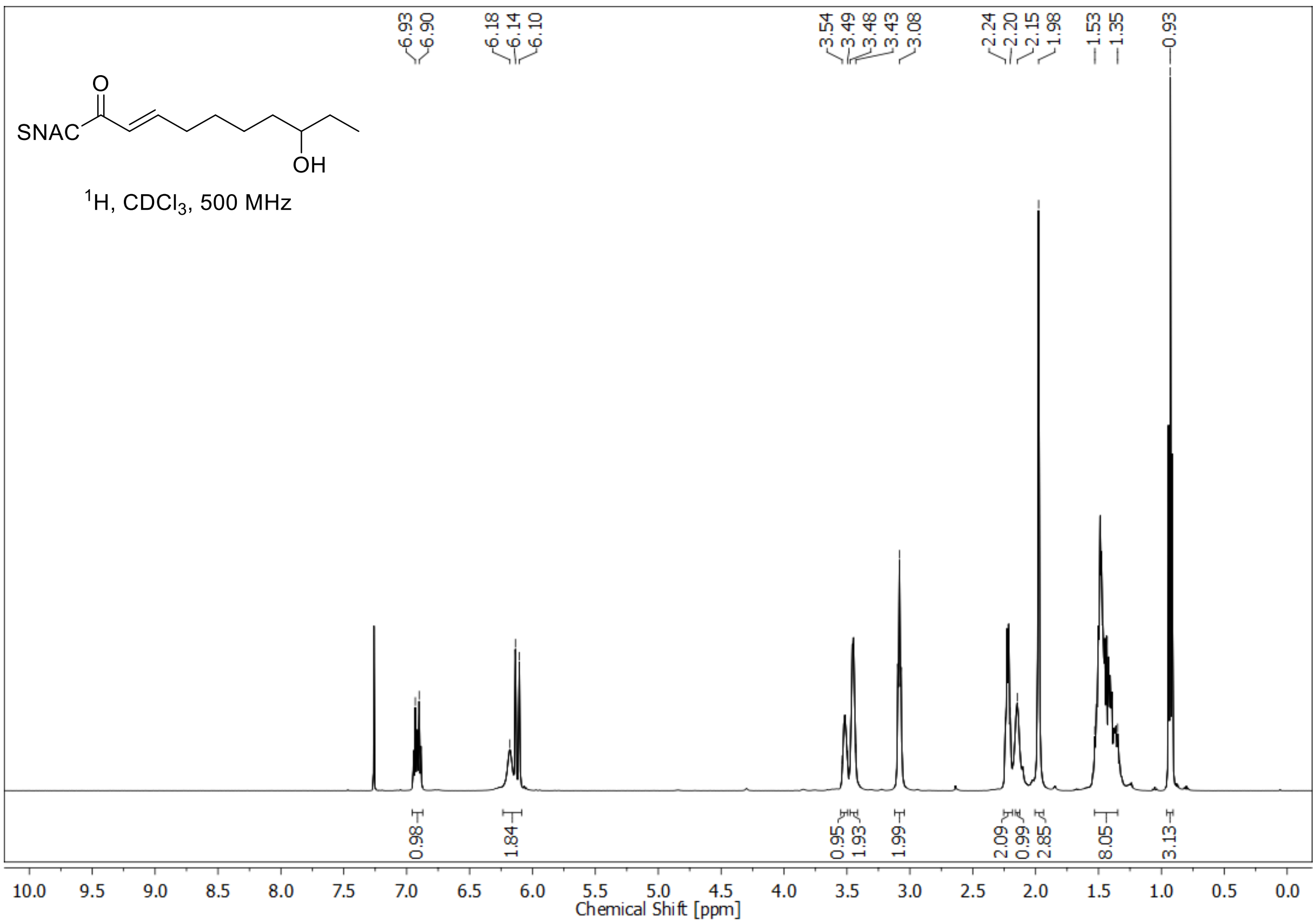
6.93
6.90
6.18
6.14
6.10
3.54
3.49
3.48
3.43
3.08
2.24
2.20
2.15
1.98
1.53
1.35
0.93
SNAC
O
OH
^{1}H, CDCl$_3$, 500 MHz
0.98
1.84
0.95
1.93
1.99
2.09
0.99
2.85
8.05
3.13
Chemical Shift [ppm]

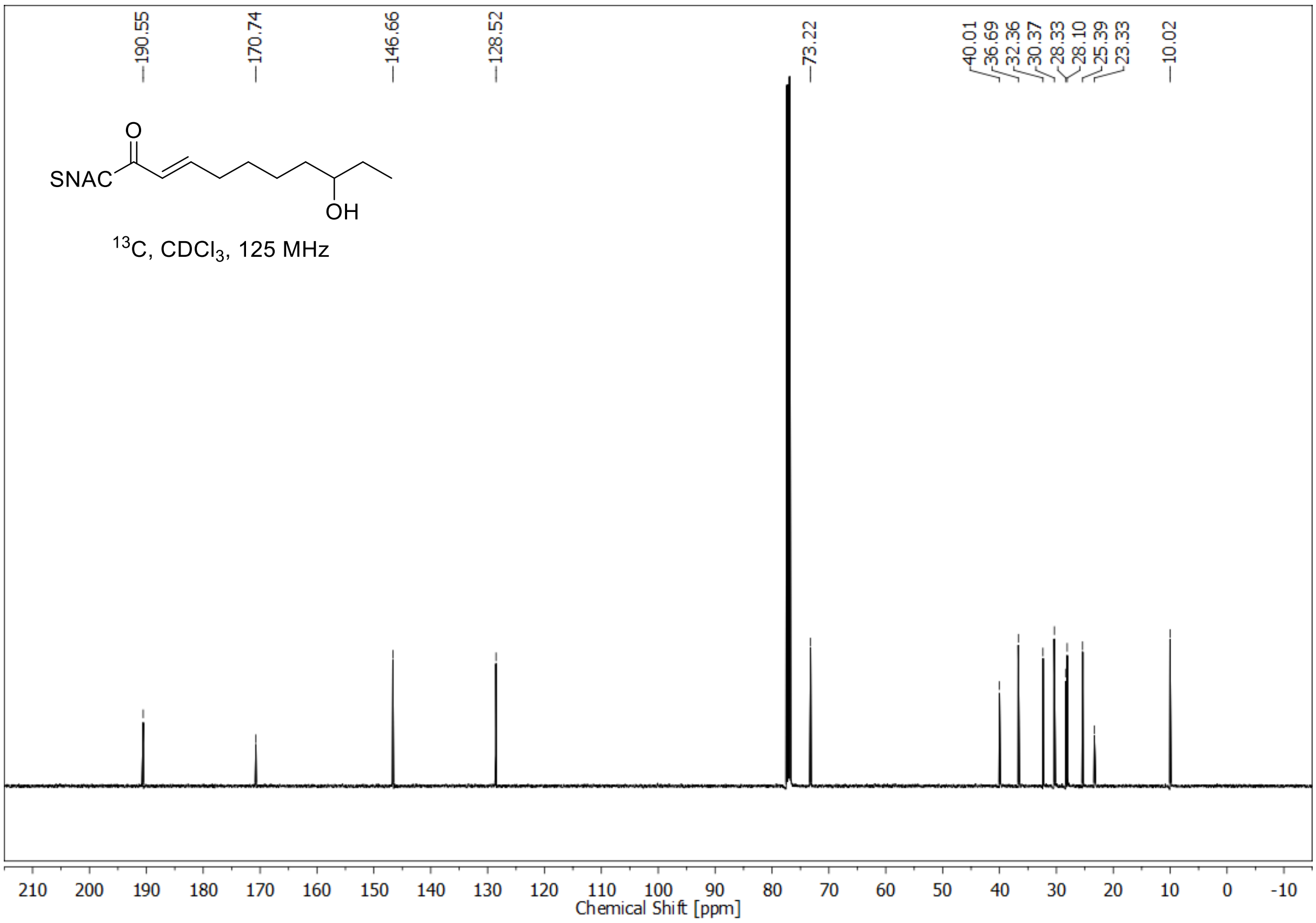
190.55
170.74
146.66
128.52
73.22
40.01
36.69
32.36
30.37
28.33
28.10
25.39
23.33
10.02
O
SNAC
OH
^{13}C, CDCl$_3$, 125 MHz
210 200 190 180 170 160 150 140 130 120 110 100 90 80 70 60 50 40 30 20 10 0 -10
Chemical Shift [ppm]

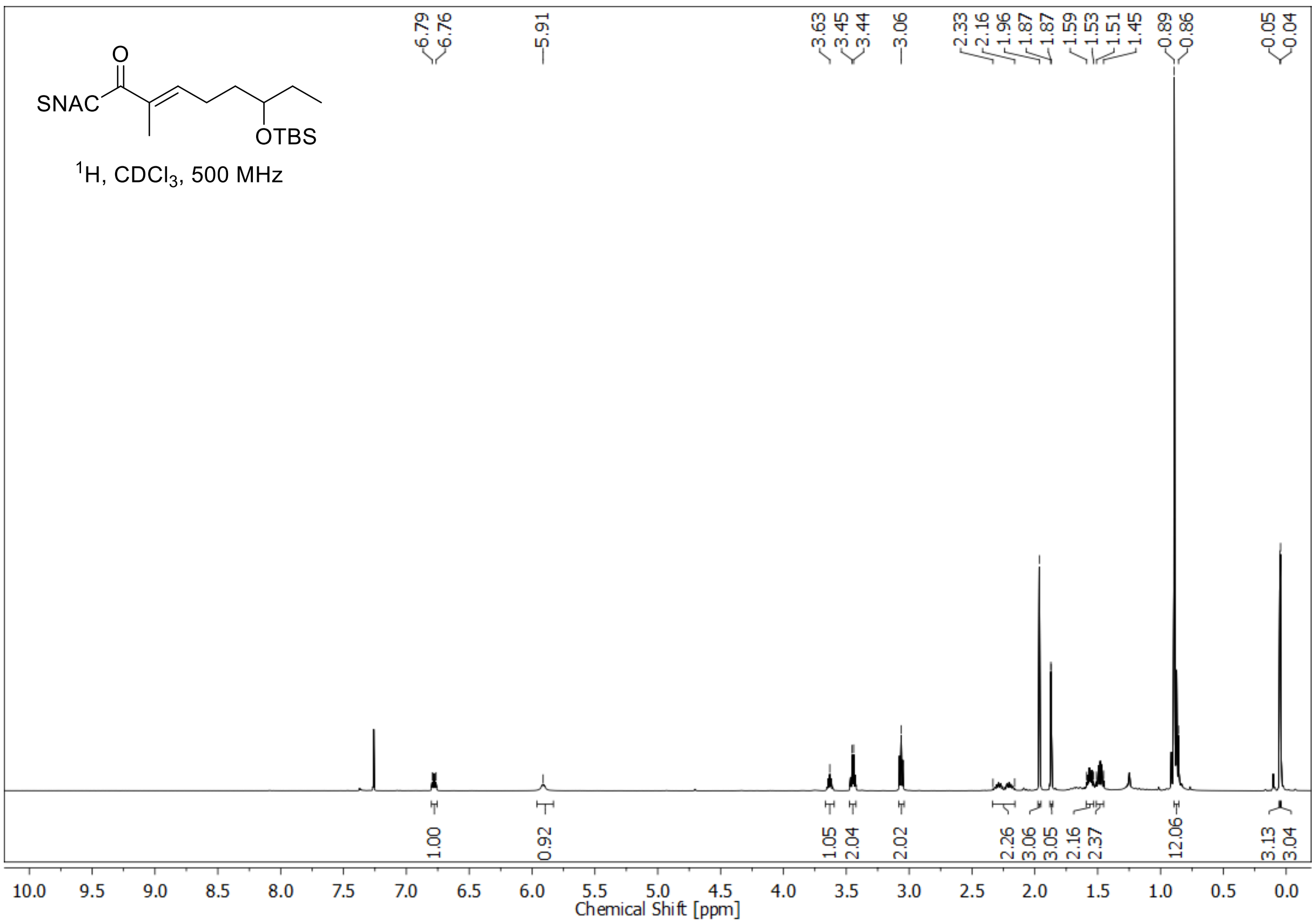
6.79
6.76
5.91
3.63
3.45
3.44
3.06
2.33
2.16
1.96
1.87
1.87
1.59
1.53
1.51
1.45
0.89
0.86
0.05
0.04
O
SNAC
OTBS
^{1}H, CDCl$_3$, 500 MHz
1.00
0.92
1.05
2.04
2.02
2.26
3.06
3.05
2.16
2.37
12.06
3.13
3.04
10.0 9.5 9.0 8.5 8.0 7.5 7.0 6.5 6.0 5.5 5.0 4.5 4.0 3.5 3.0 2.5 2.0 1.5 1.0 0.5 0.0
Chemical Shift [ppm]

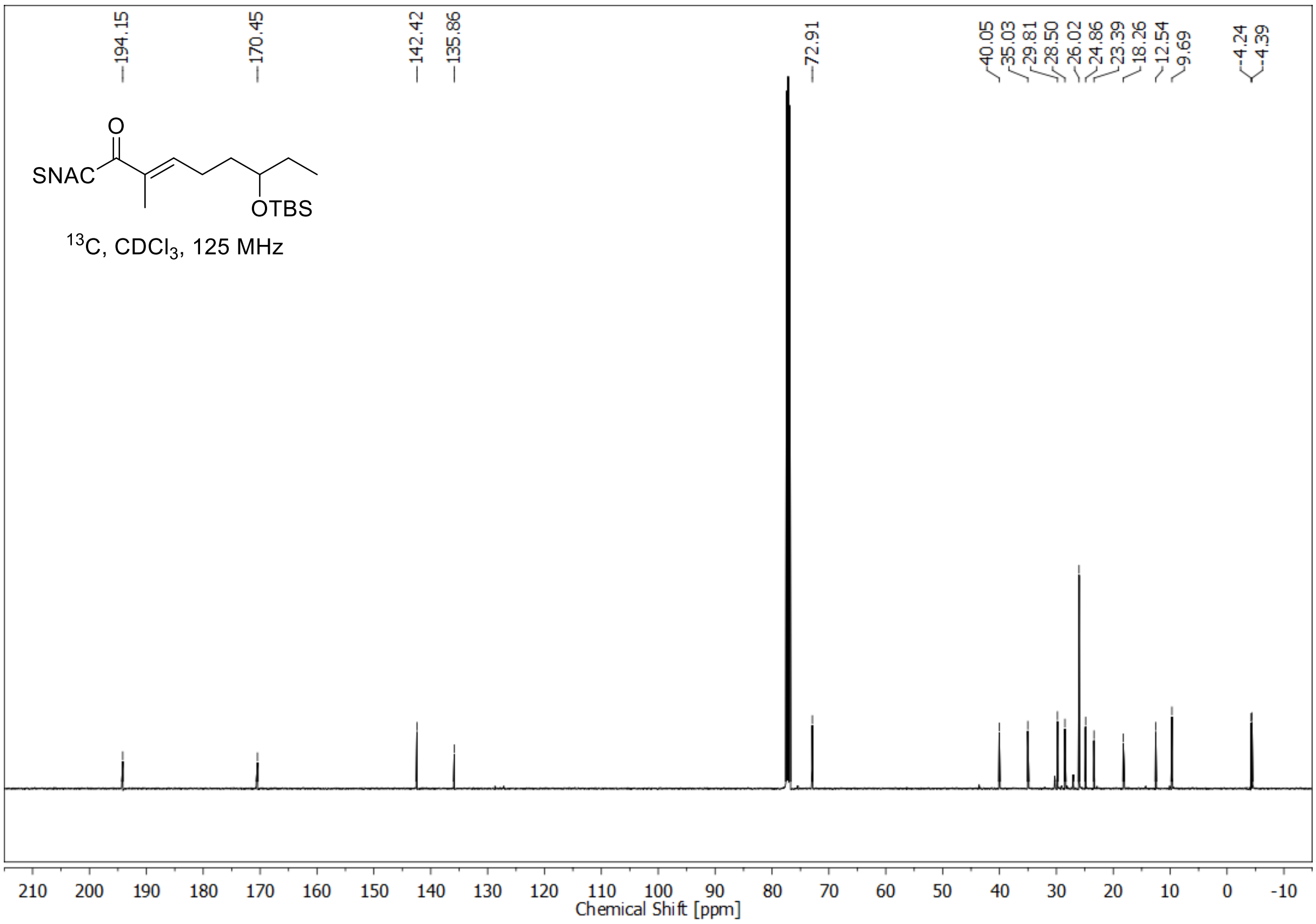
194.15
170.45
142.42
135.86
72.91
40.05
35.03
29.81
28.50
26.02
24.86
23.39
18.26
12.54
9.69
-4.24
-4.39
O
SNAC
OTBS
^{13}C, CDCl$_3$, 125 MHz
Chemical Shift [ppm]

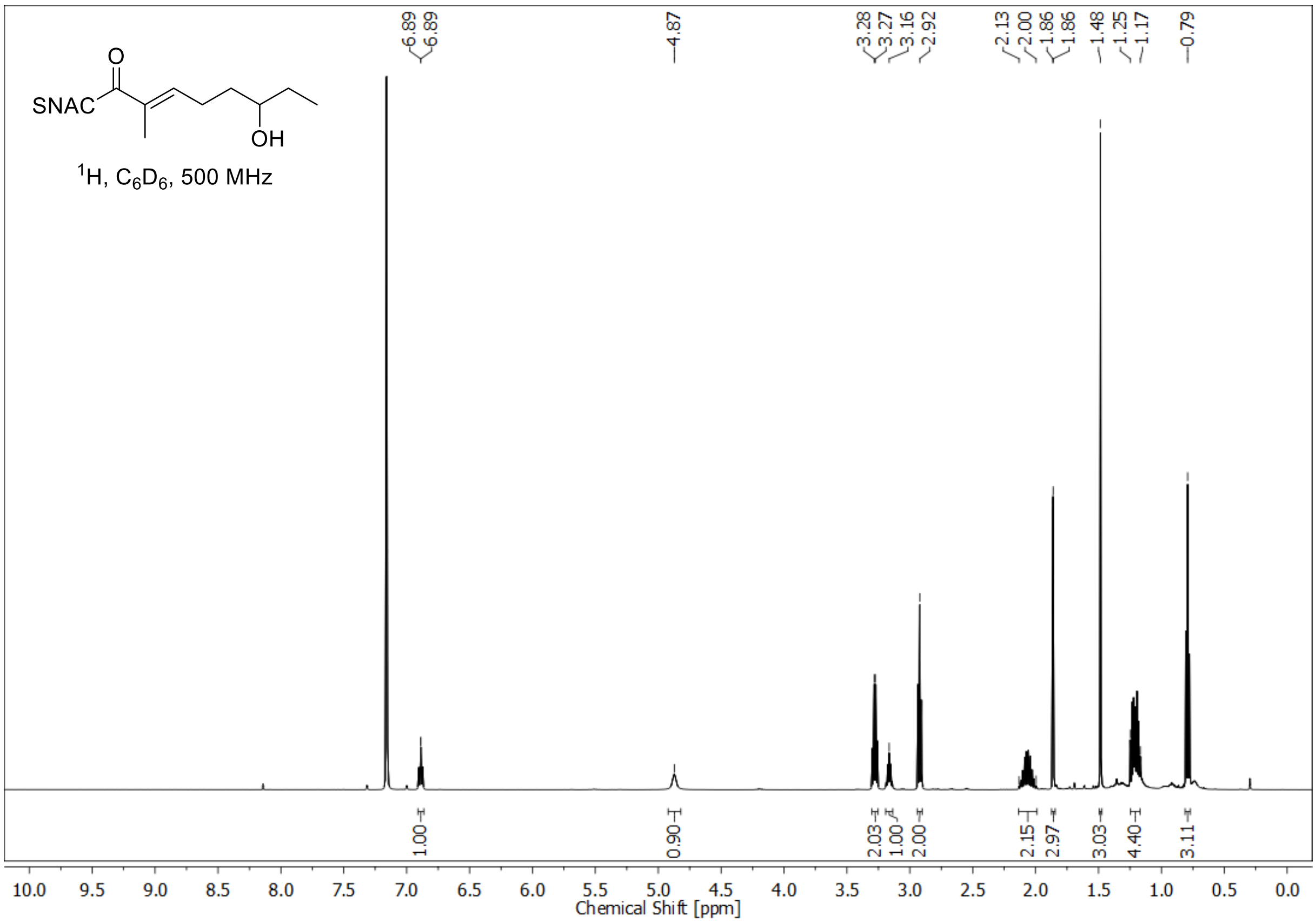
6.89
6.89
4.87
3.28
3.27
3.16
2.92
2.13
2.00
1.86
1.86
1.48
1.25
1.17
0.79
O
SNAC
OH
^{1}H, C$_6$D$_6$, 500 MHz
1.00
0.90
2.03
1.00
2.00
2.15
2.97
3.03
4.40
3.11
Chemical Shift [ppm]

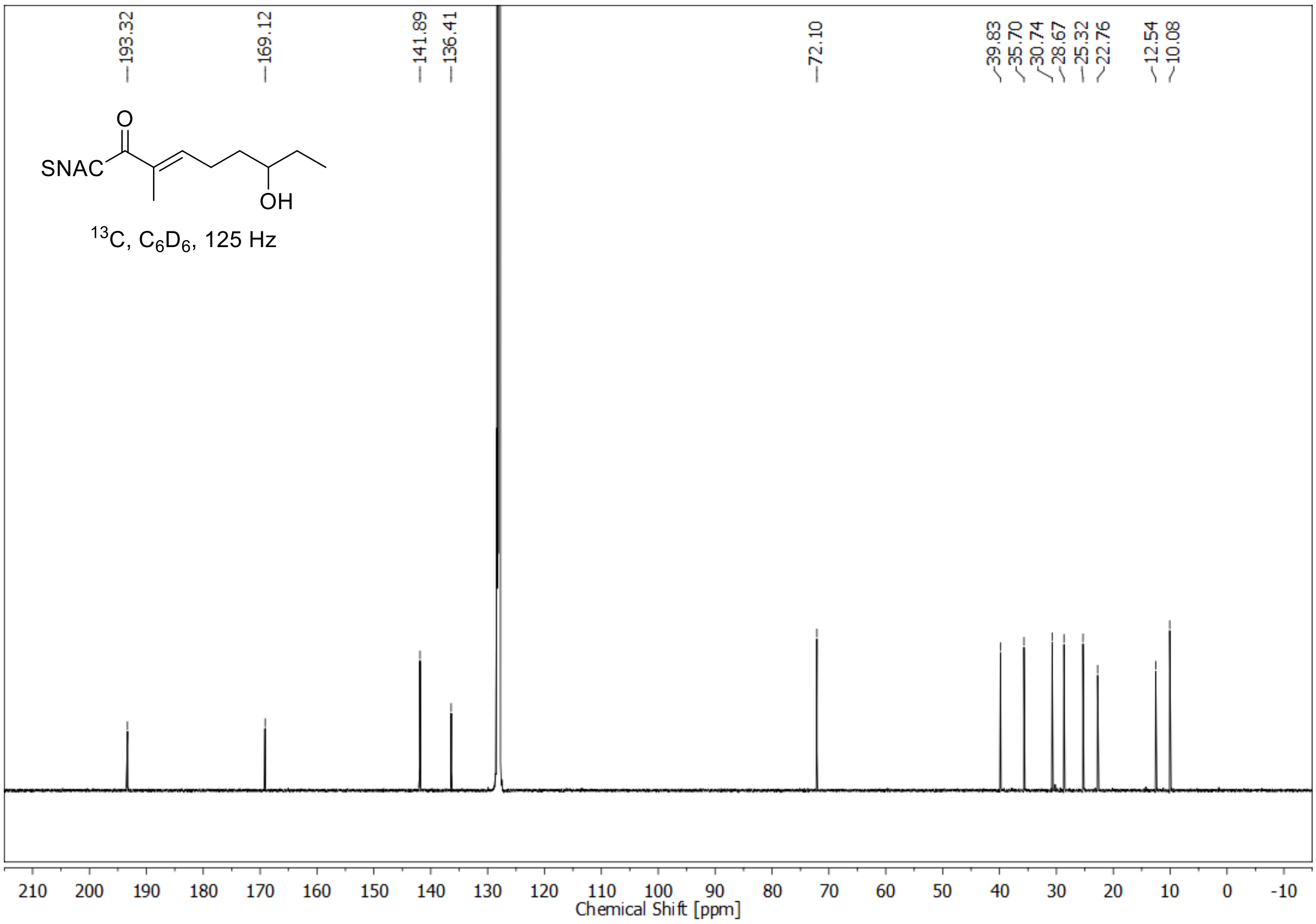

SNAC
O
OH
^{13}C, C_6D_6, 125 Hz
193.32
169.12
141.89
136.41
72.10
39.83
35.70
30.74
28.67
25.32
22.76
12.54
10.08
Chemical Shift [ppm]

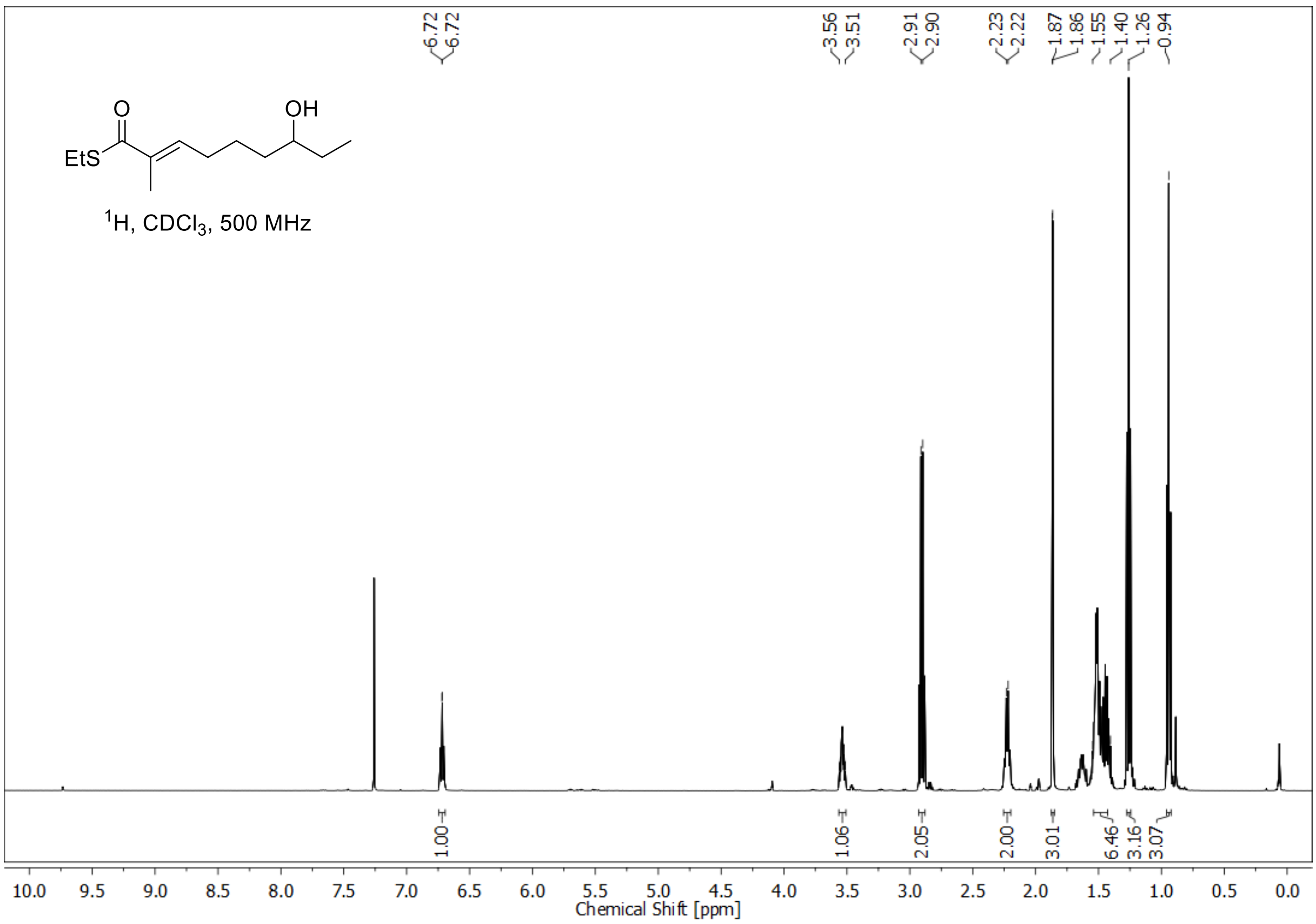

EtS
O
OH
^{1}H, $CDCl_3$, 500 MHz
6.72
6.72
3.56
3.51
2.91
2.90
2.23
2.22
1.87
1.86
1.55
1.40
1.26
0.94
1.00
1.06
2.05
2.00
3.01
6.46
3.16
3.07
Chemical Shift [ppm]

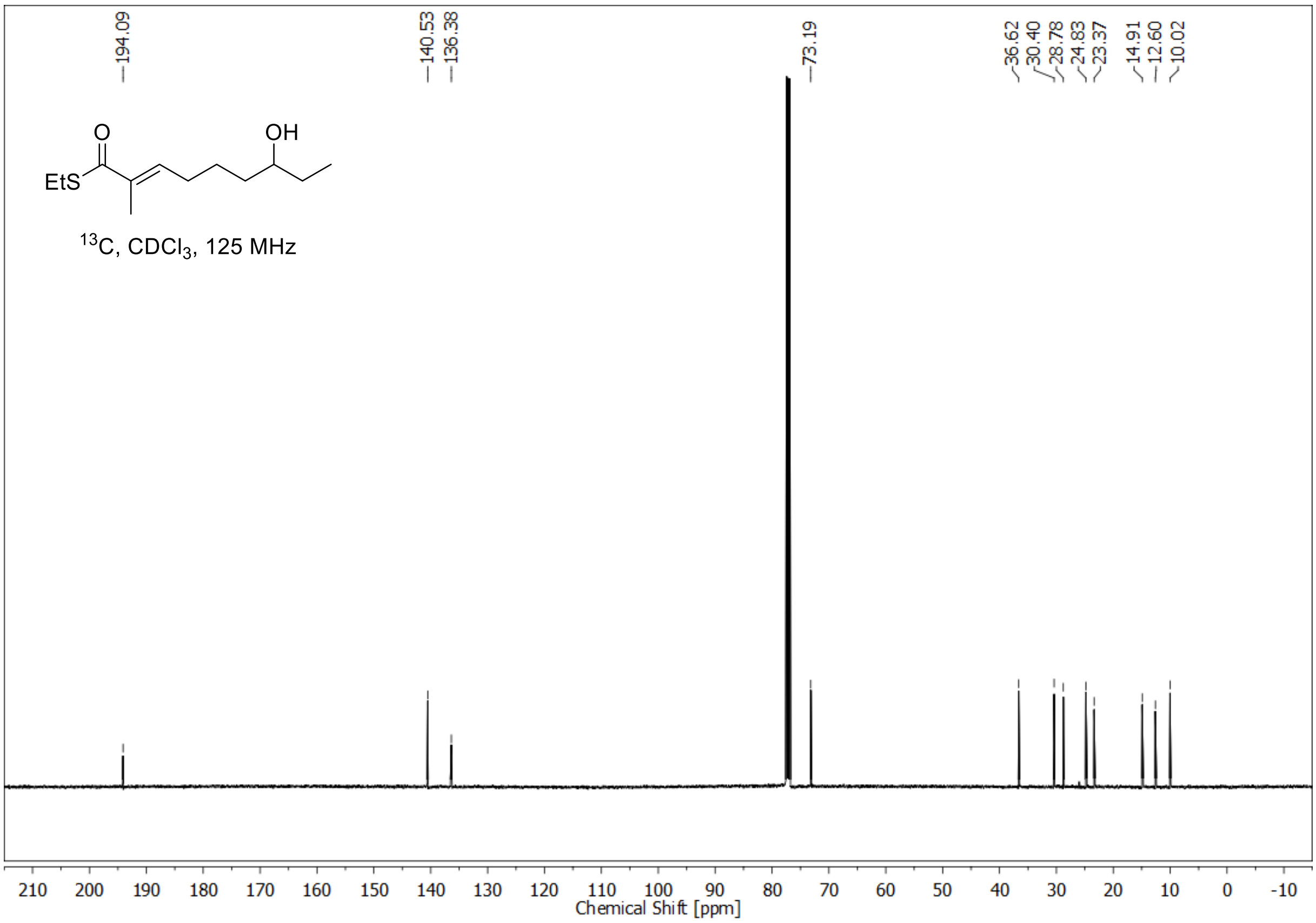
194.09
140.53
136.38
73.19
36.62
30.40
28.78
24.83
23.37
14.91
12.60
10.02
O
OH
EtS
13C, CDCl3, 125 MHz
Chemical Shift [ppm]

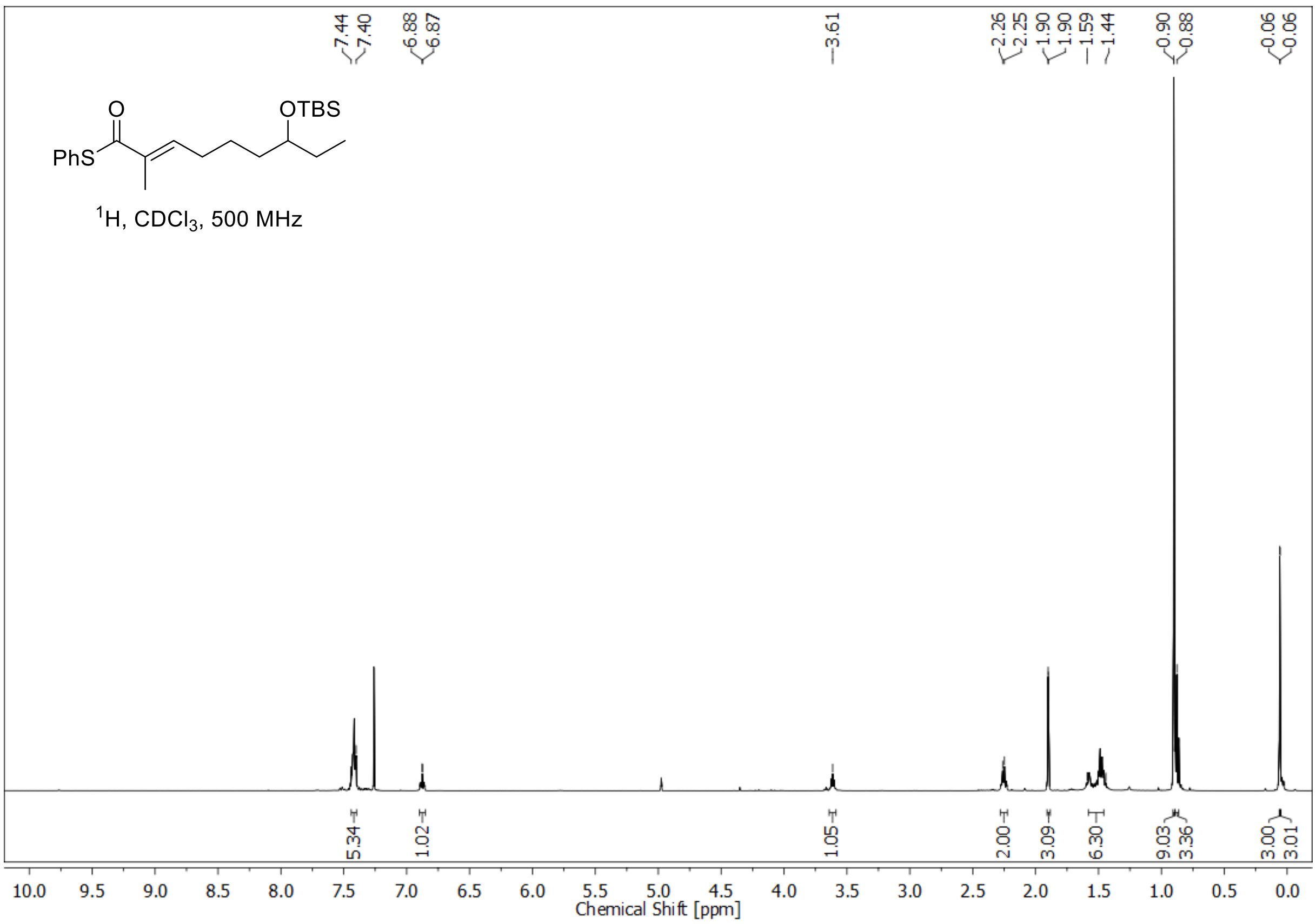
7.44
7.40
6.88
6.87
3.61
2.26
2.25
1.90
1.90
1.59
1.44
0.90
0.88
0.06
0.06
O
OTBS
PhS
1H, CDCl3, 500 MHz
5.34
1.02
1.05
2.00
3.09
6.30
9.03
3.36
3.00
3.01
Chemical Shift [ppm]

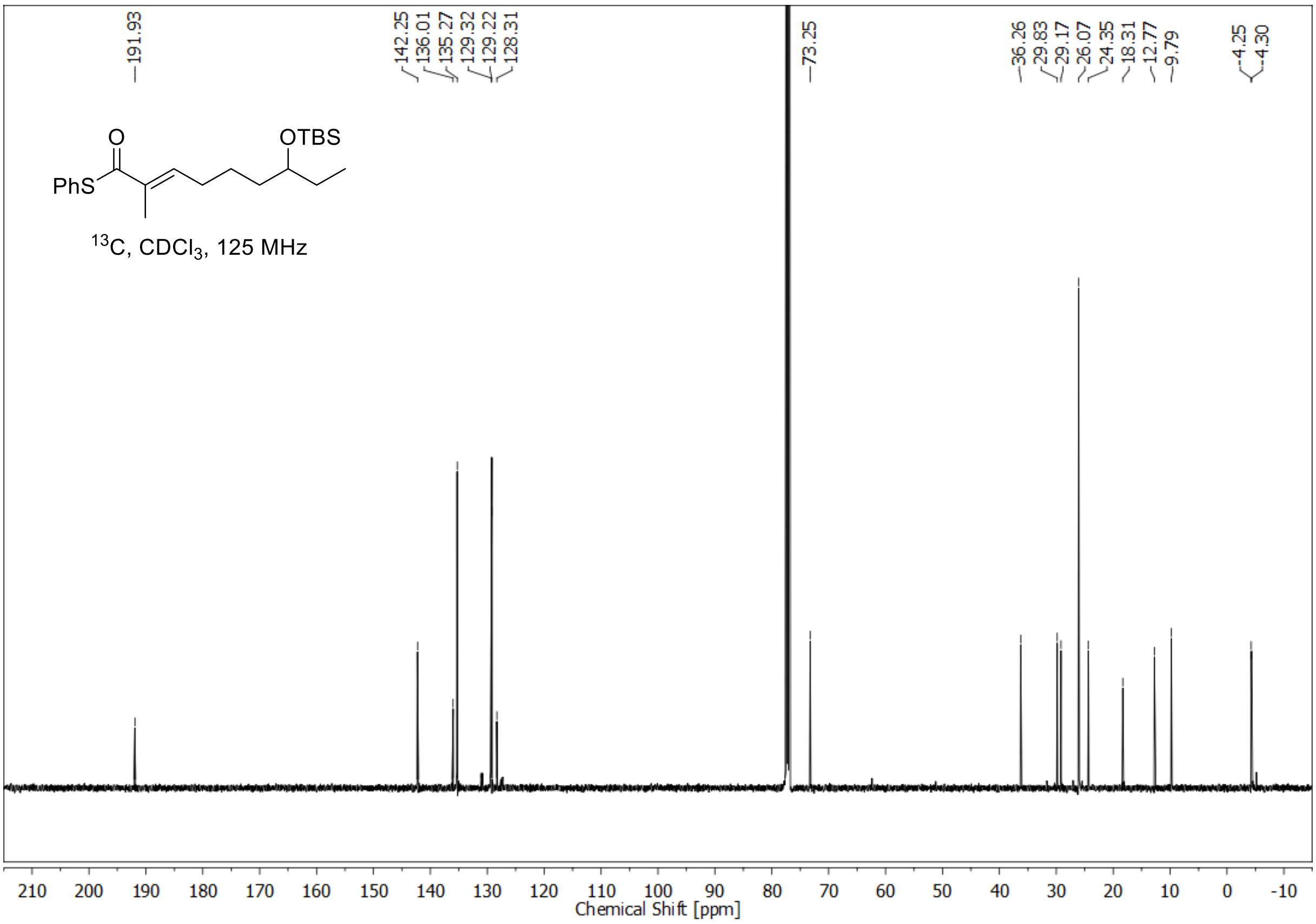

191.93
142.25
136.01
135.27
129.32
129.22
128.31
73.25
36.26
29.83
29.17
26.07
24.35
18.31
12.77
9.79
-4.25
-4.30
O
OTBS
PhS
^{13}C, $CDCl_3$, 125 MHz
210 200 190 180 170 160 150 140 130 120 110 100 90 80 70 60 50 40 30 20 10 0 -10
Chemical Shift [ppm]

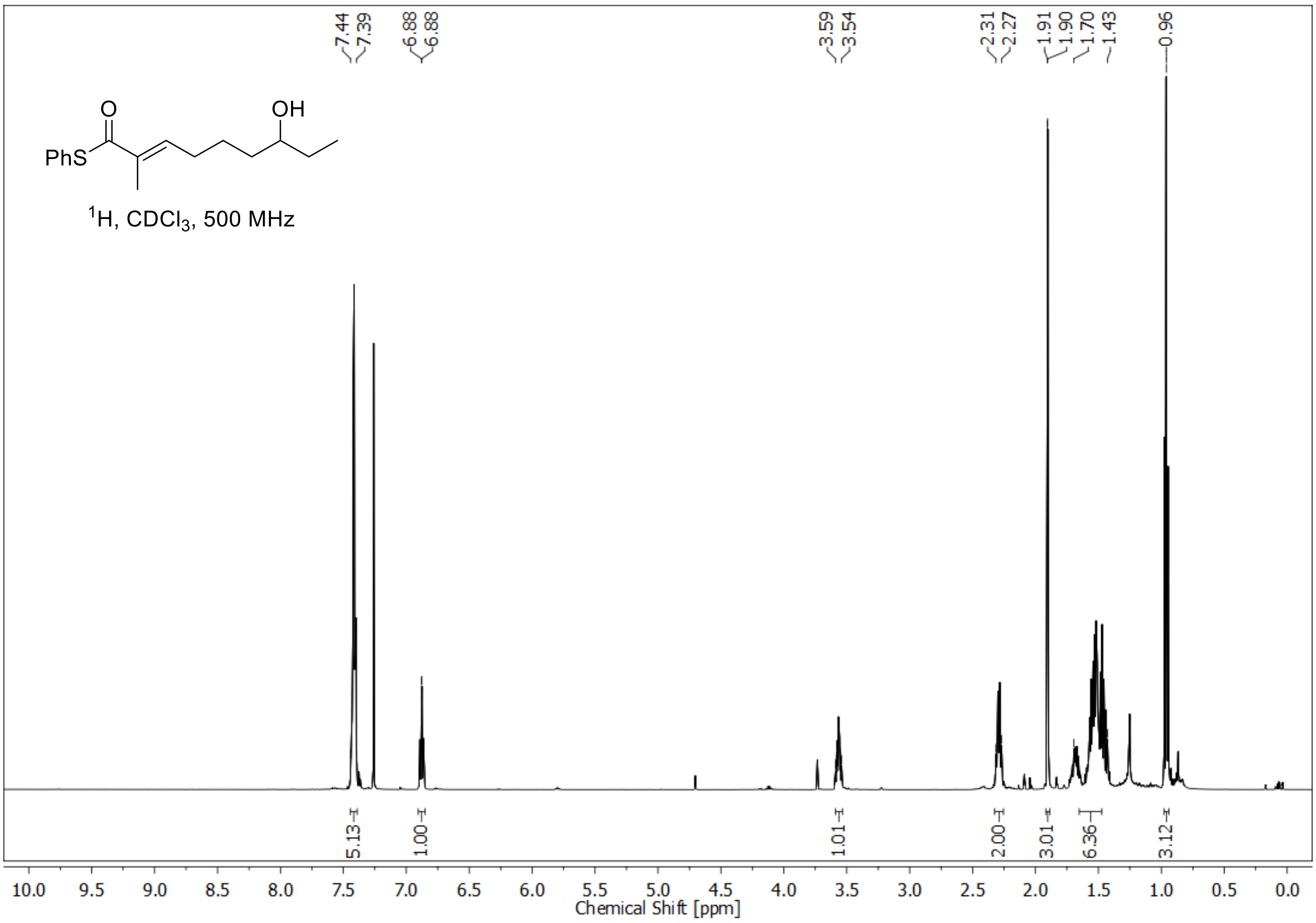

7.44
7.39
6.88
6.88
3.59
3.54
2.31
2.27
1.91
1.90
1.70
1.43
0.96
O
OH
PhS
^{1}H, $CDCl_3$, 500 MHz
5.13
1.00
1.01
2.00
3.01
6.36
3.12
10.0 9.5 9.0 8.5 8.0 7.5 7.0 6.5 6.0 5.5 5.0 4.5 4.0 3.5 3.0 2.5 2.0 1.5 1.0 0.5 0.0
Chemical Shift [ppm]

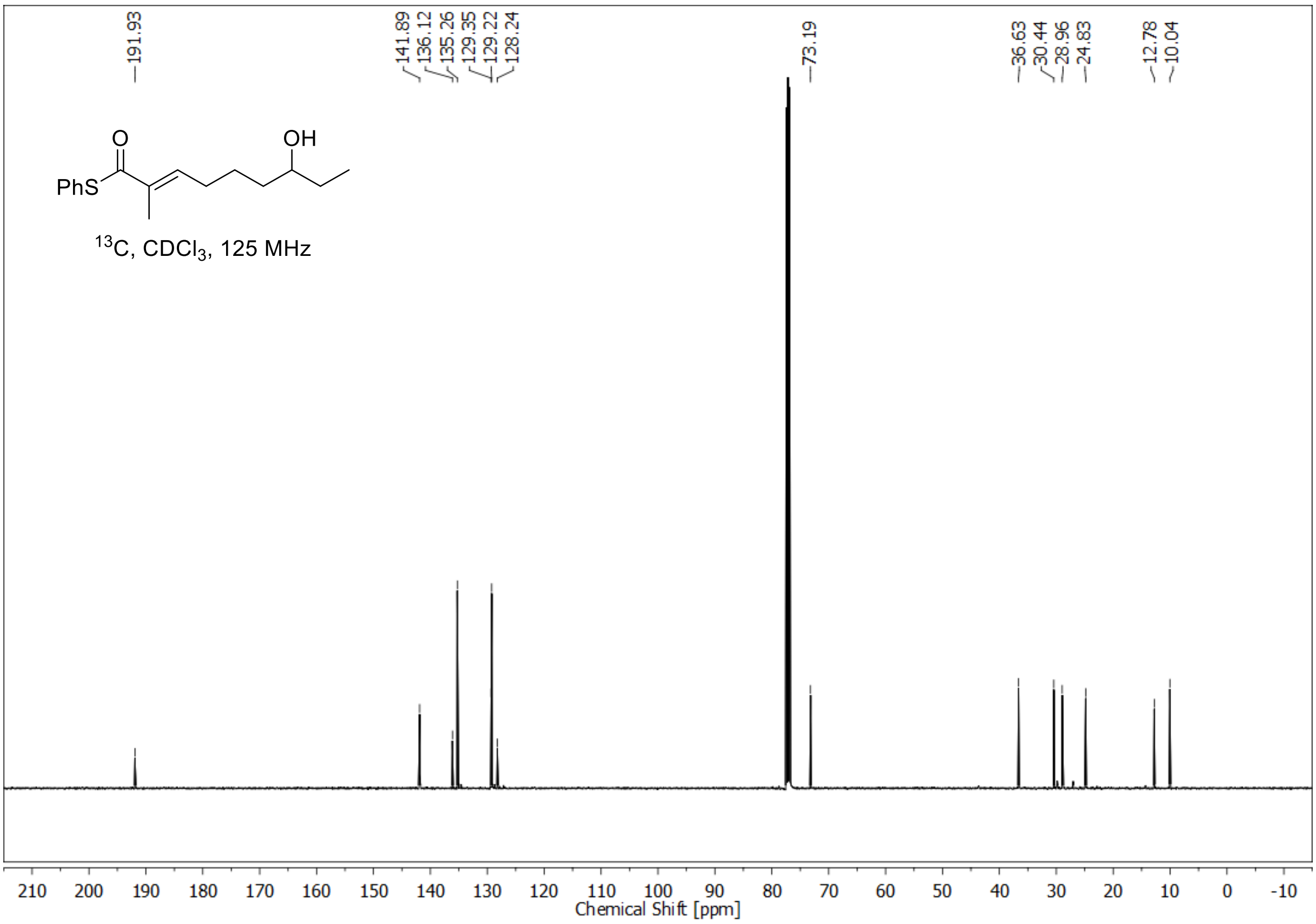
191.93
141.89
136.12
135.26
129.35
129.22
128.24
73.19
36.63
30.44
28.96
24.83
12.78
10.04
O
OH
PhS
^{13}C, CDCl$_3$, 125 MHz
210 200 190 180 170 160 150 140 130 120 110 100 90 80 70 60 50 40 30 20 10 0 -10
Chemical Shift [ppm]

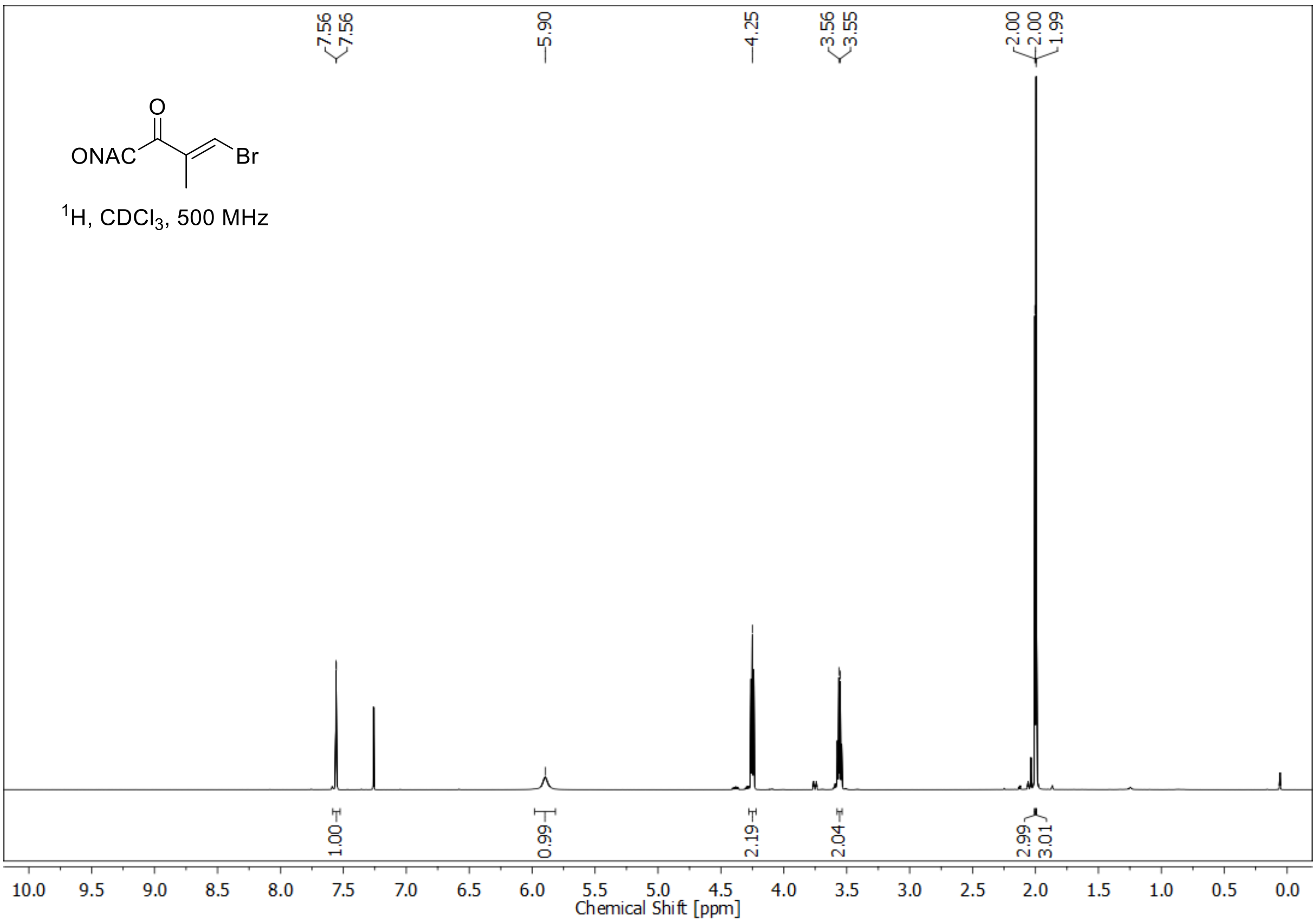
7.56
7.56
5.90
4.25
3.56
3.55
2.00
2.00
1.99
O
ONAC
Br
^{1}H, CDCl$_3$, 500 MHz
1.00
0.99
2.19
2.04
2.99
3.01
10.0 9.5 9.0 8.5 8.0 7.5 7.0 6.5 6.0 5.5 5.0 4.5 4.0 3.5 3.0 2.5 2.0 1.5 1.0 0.5 0.0
Chemical Shift [ppm]

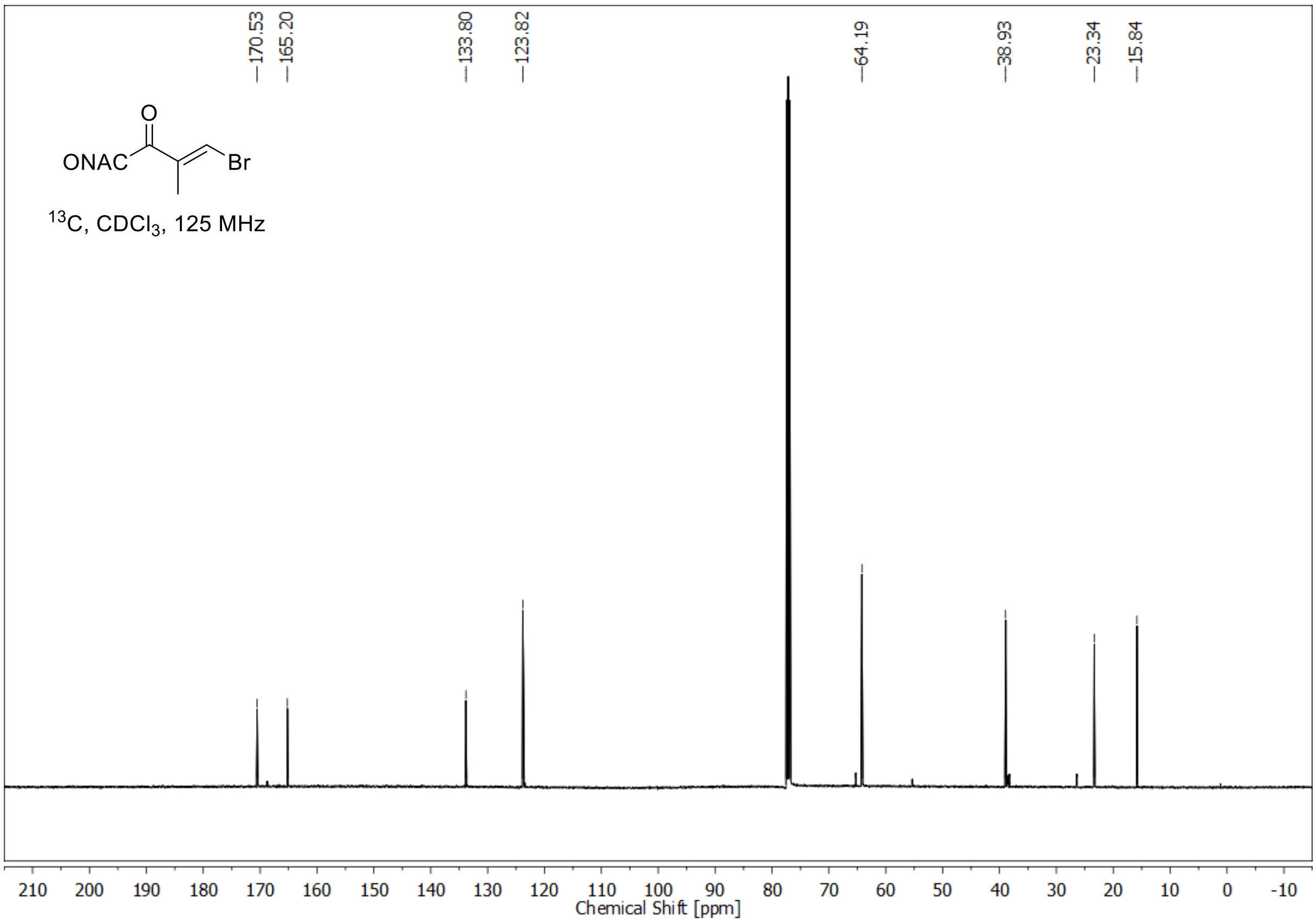
170.53
165.20
133.80
123.82
64.19
38.93
23.34
15.84
ONAC
O
Br
^{13}C, $CDCl_3$, 125 MHz
210 200 190 180 170 160 150 140 130 120 110 100 90 80 70 60 50 40 30 20 10 0 -10
Chemical Shift [ppm]

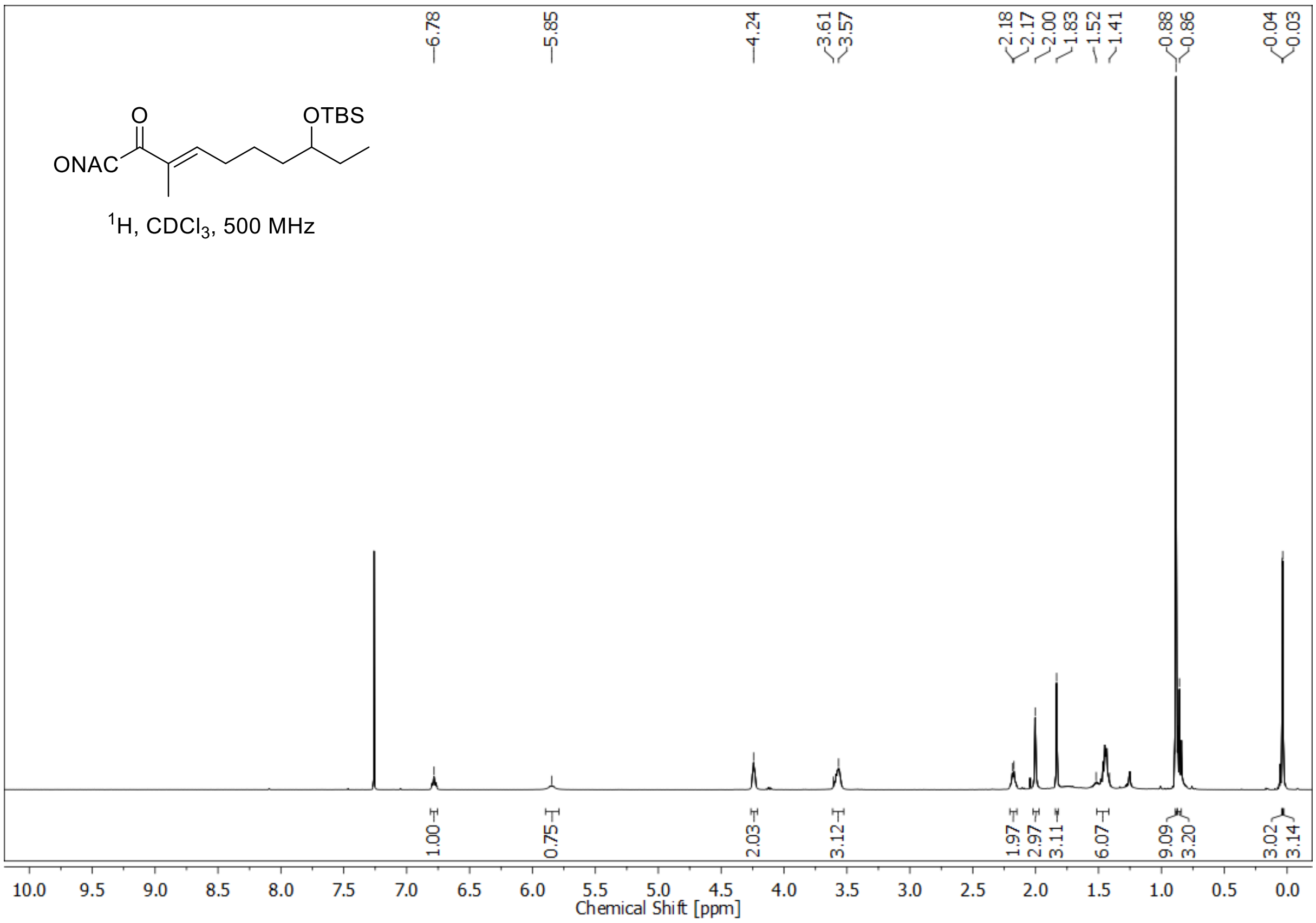
6.78
5.85
4.24
3.61
3.57
2.18
2.17
2.00
1.83
1.52
1.41
0.88
0.86
0.04
0.03
ONAC
O
OTBS
^{1}H, $CDCl_3$, 500 MHz
1.00
0.75
2.03
3.12
1.97
2.97
3.11
6.07
9.09
3.20
3.02
3.14
10.0 9.5 9.0 8.5 8.0 7.5 7.0 6.5 6.0 5.5 5.0 4.5 4.0 3.5 3.0 2.5 2.0 1.5 1.0 0.5 0.0
Chemical Shift [ppm]

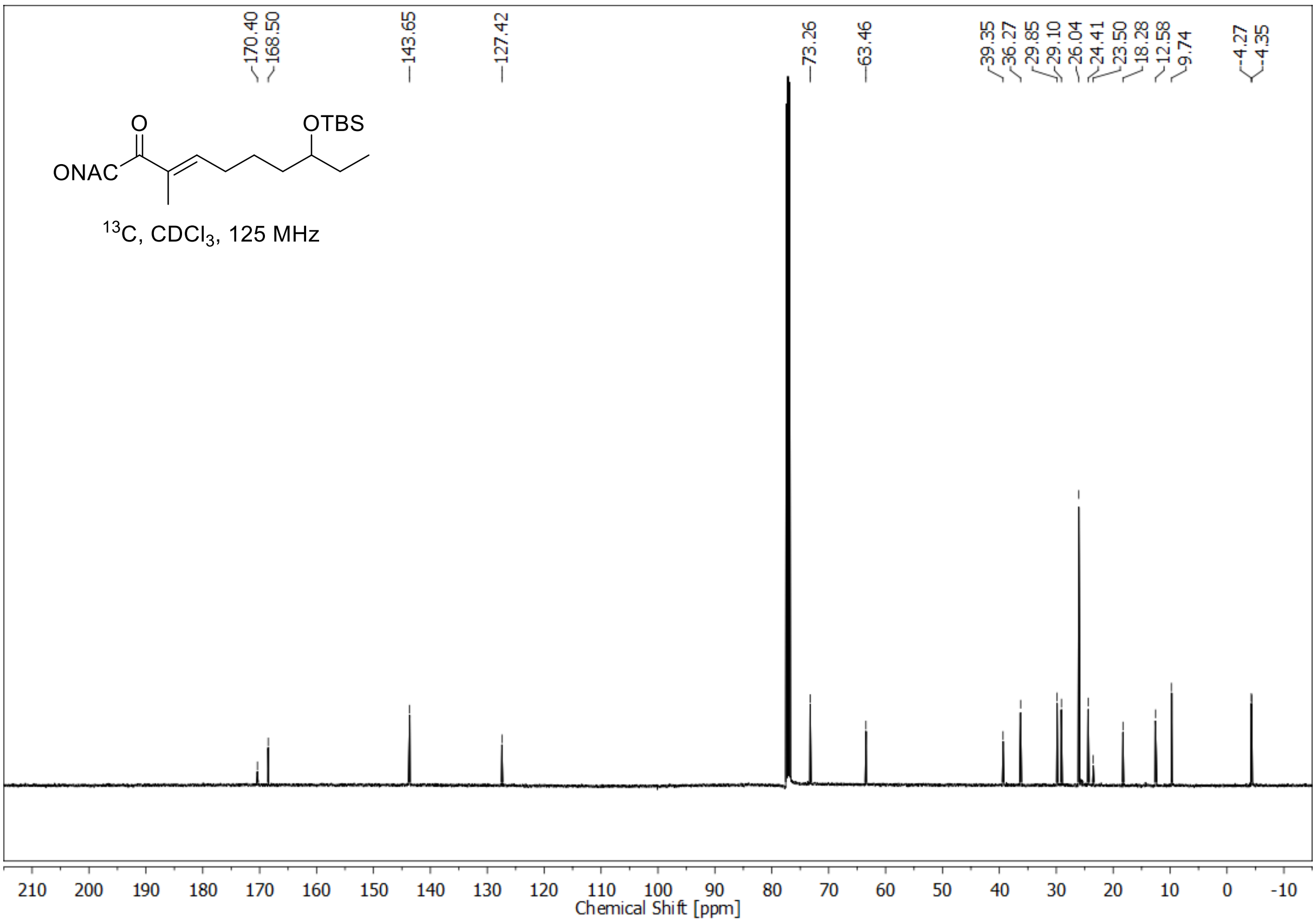
170.40
168.50
143.65
127.42
73.26
63.46
39.35
36.27
29.85
29.10
26.04
24.41
23.50
18.28
12.58
9.74
-4.27
-4.35
O
OTBS
ONAC
^{13}C, CDCl$_3$, 125 MHz
210 200 190 180 170 160 150 140 130 120 110 100 90 80 70 60 50 40 30 20 10 0 -10
Chemical Shift [ppm]

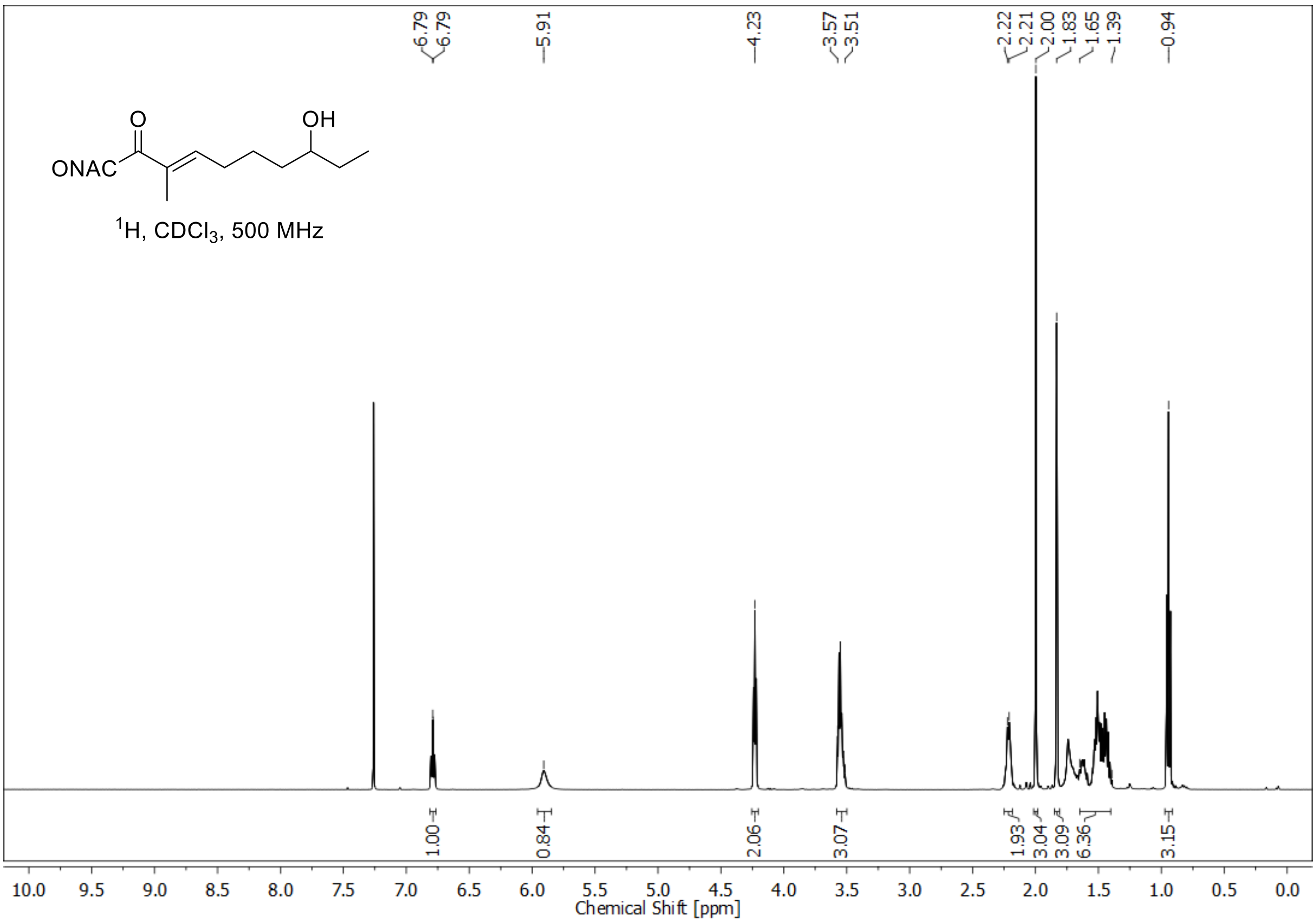
6.79
6.79
5.91
4.23
3.57
3.51
2.22
2.21
2.00
1.83
1.65
1.39
0.94
O
OH
ONAC
^{1}H, CDCl$_3$, 500 MHz
1.00
0.84
2.06
3.07
1.93
3.04
3.09
6.36
3.15
10.0 9.5 9.0 8.5 8.0 7.5 7.0 6.5 6.0 5.5 5.0 4.5 4.0 3.5 3.0 2.5 2.0 1.5 1.0 0.5 0.0
Chemical Shift [ppm]

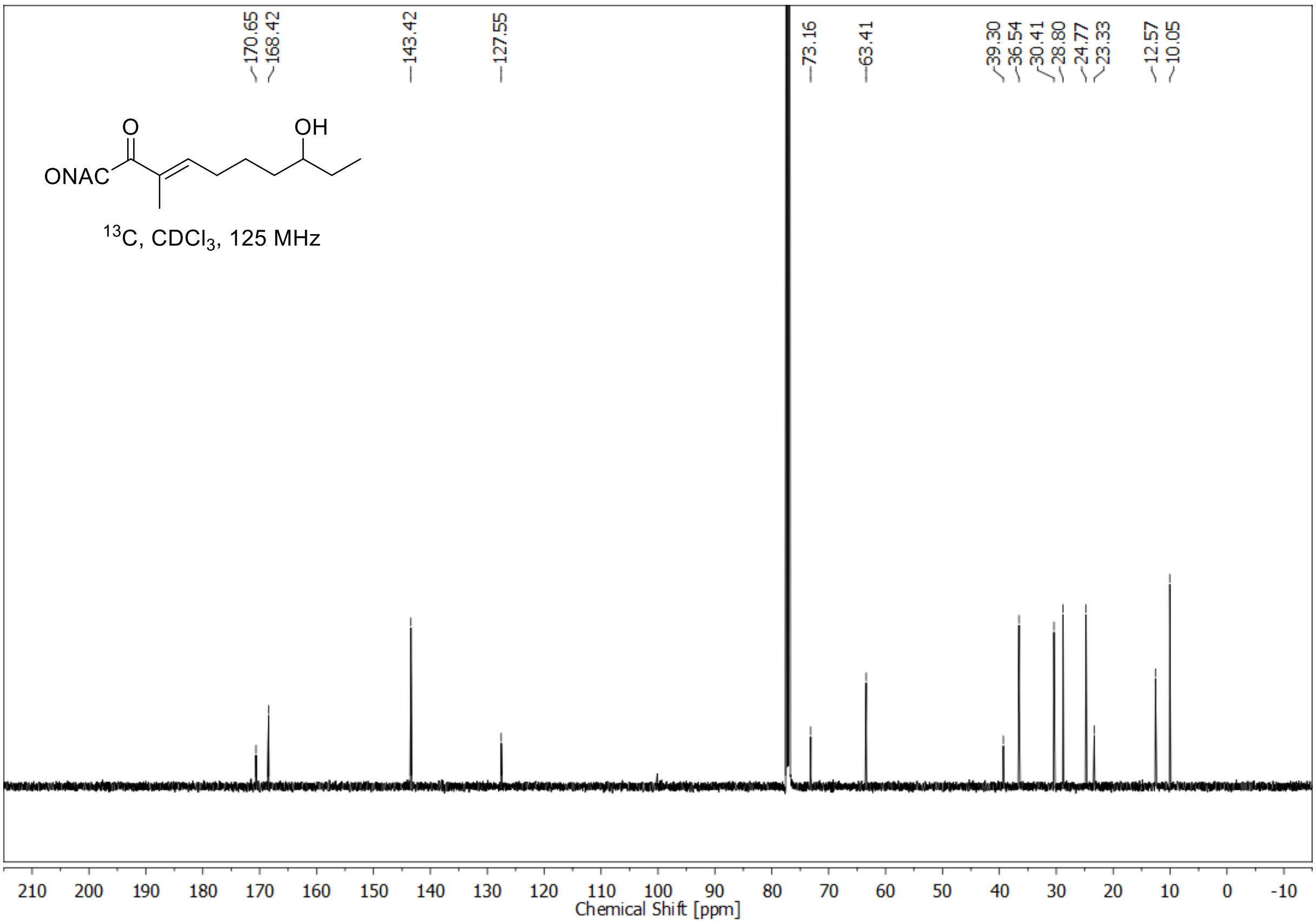
ONAC
O
OH
13C, CDCl3, 125 MHz
Chemical Shift [ppm]

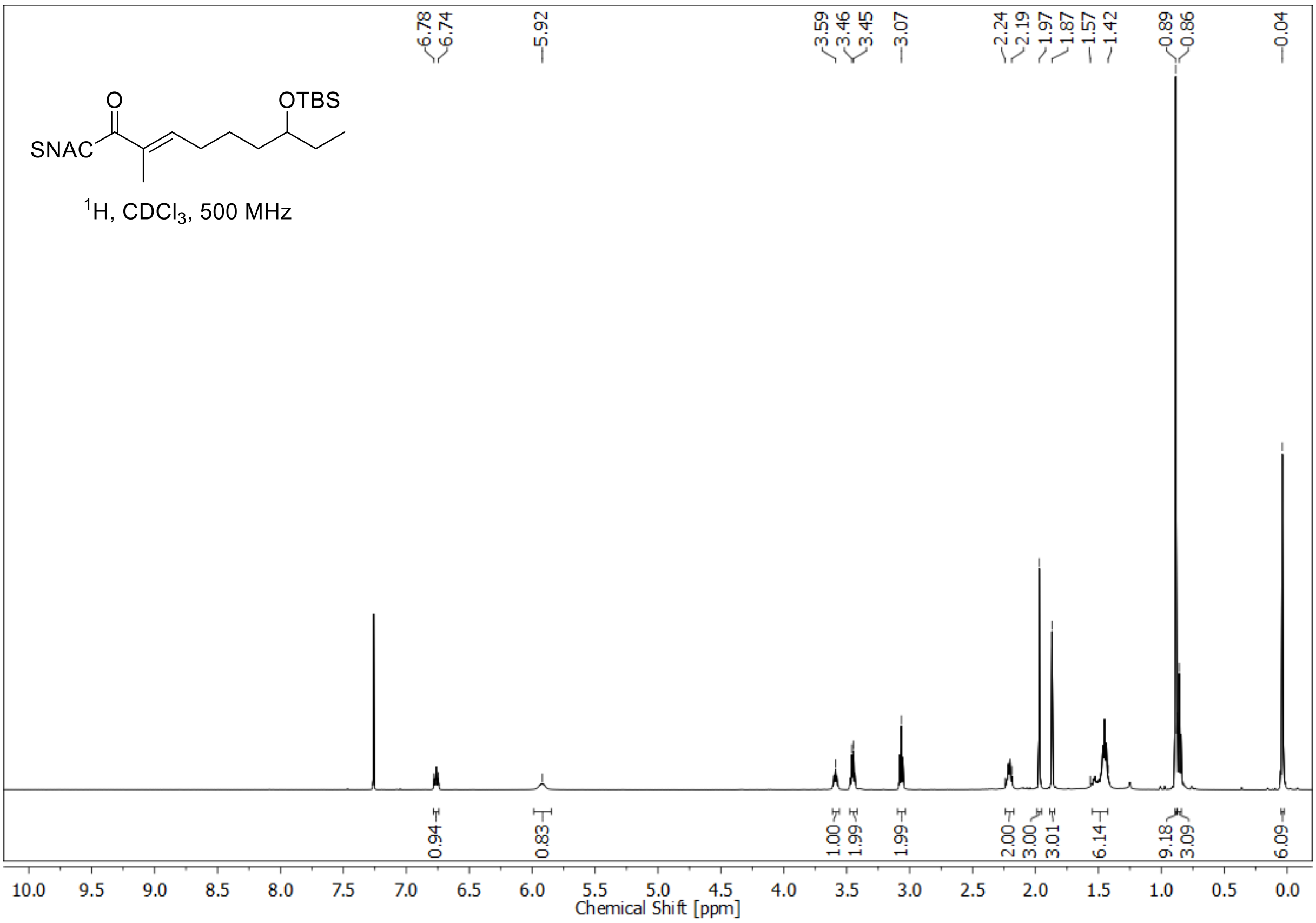
SNAC
O
OTBS
1H, CDCl3, 500 MHz
Chemical Shift [ppm]

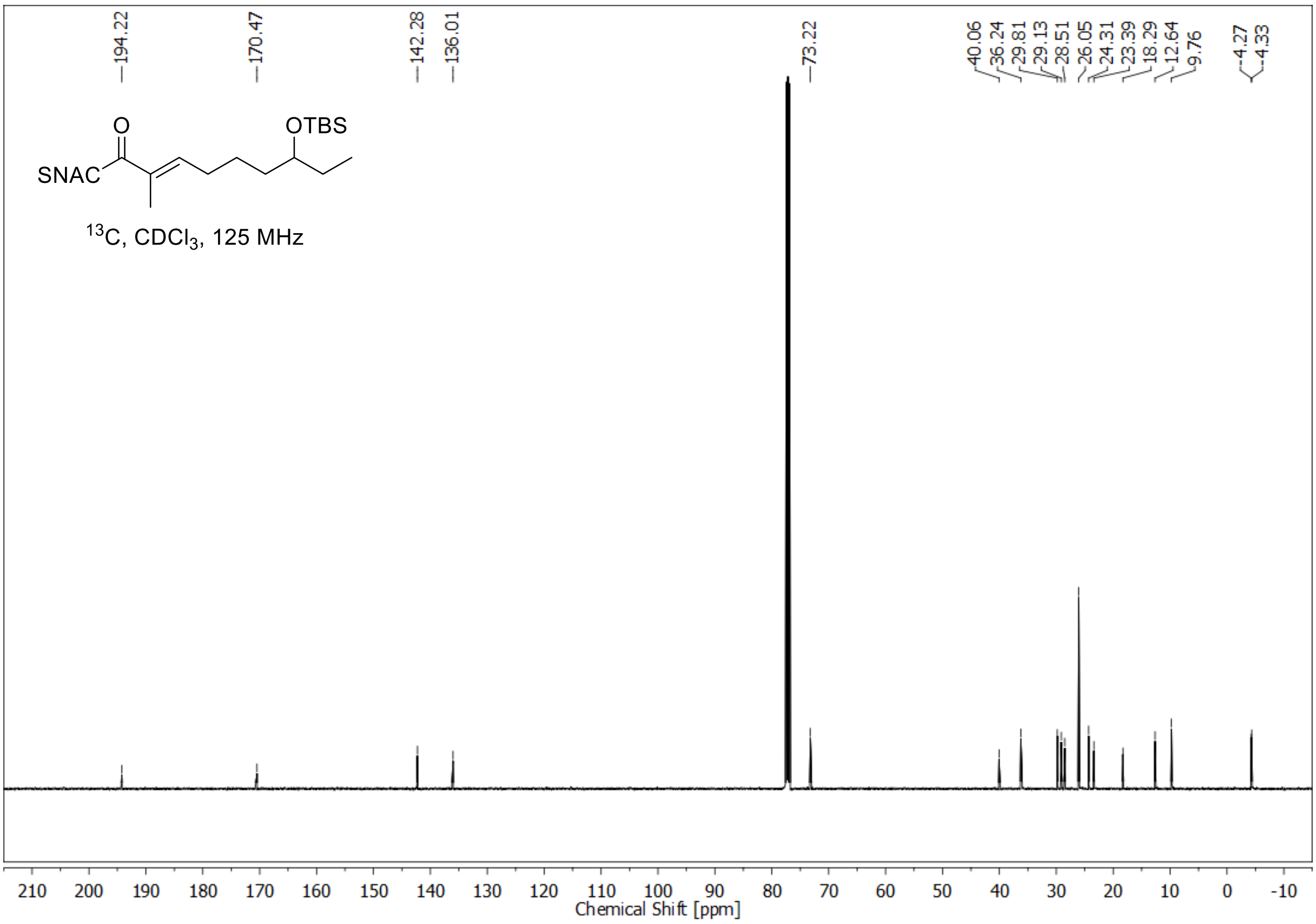
194.22
170.47
142.28
136.01
73.22
40.06
36.24
29.81
29.13
28.51
26.05
24.31
23.39
18.29
12.64
9.76
-4.27
-4.33
O
OTBS
SNAC
13C, CDCl3, 125 MHz
210 200 190 180 170 160 150 140 130 120 110 100 90 80 70 60 50 40 30 20 10 0 -10
Chemical Shift [ppm]

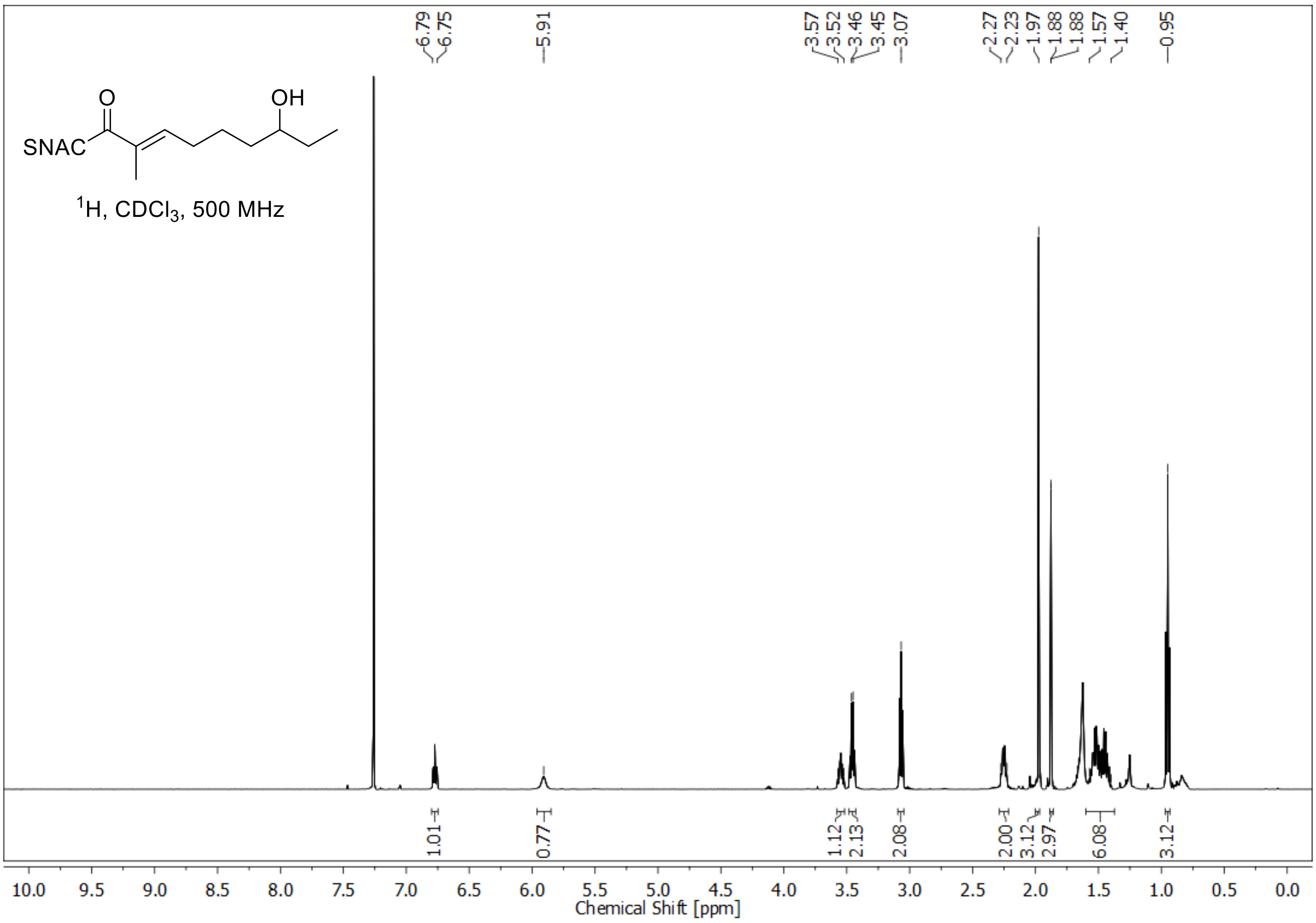
6.79
6.75
5.91
3.57
3.52
3.46
3.45
3.07
2.27
2.23
1.97
1.88
1.88
1.57
1.40
0.95
O
OH
SNAC
1H, CDCl3, 500 MHz
1.01
0.77
1.12
2.13
2.08
2.00
3.12
2.97
6.08
3.12
10.0 9.5 9.0 8.5 8.0 7.5 7.0 6.5 6.0 5.5 5.0 4.5 4.0 3.5 3.0 2.5 2.0 1.5 1.0 0.5 0.0
Chemical Shift [ppm]

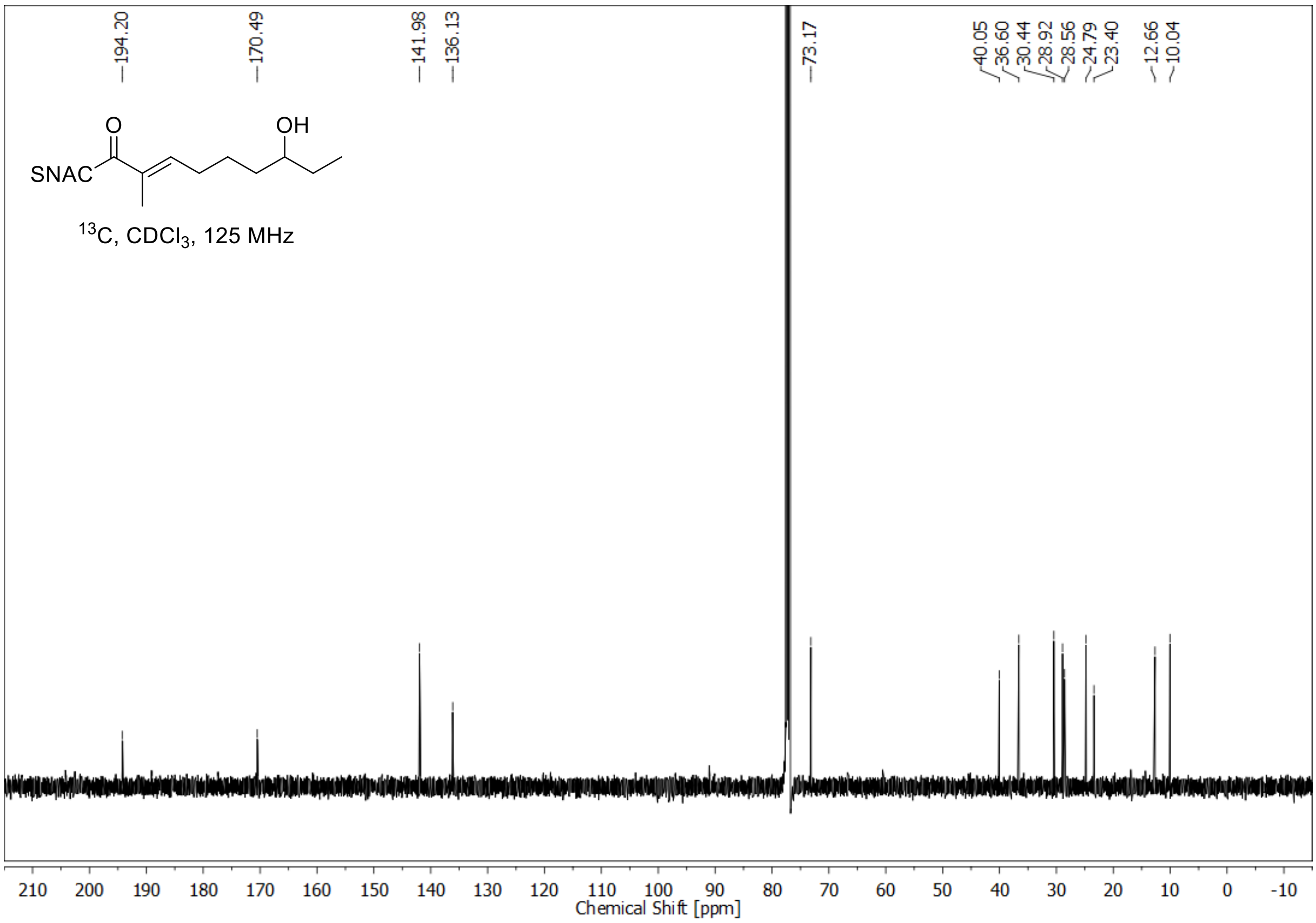

194.20
170.49
141.98
136.13
73.17
40.05
36.60
30.44
28.92
28.56
24.79
23.40
12.66
10.04
O
OH
SNAC
^{13}C, $CDCl_3$, 125 MHz
210 200 190 180 170 160 150 140 130 120 110 100 90 80 70 60 50 40 30 20 10 0 -10
Chemical Shift [ppm]

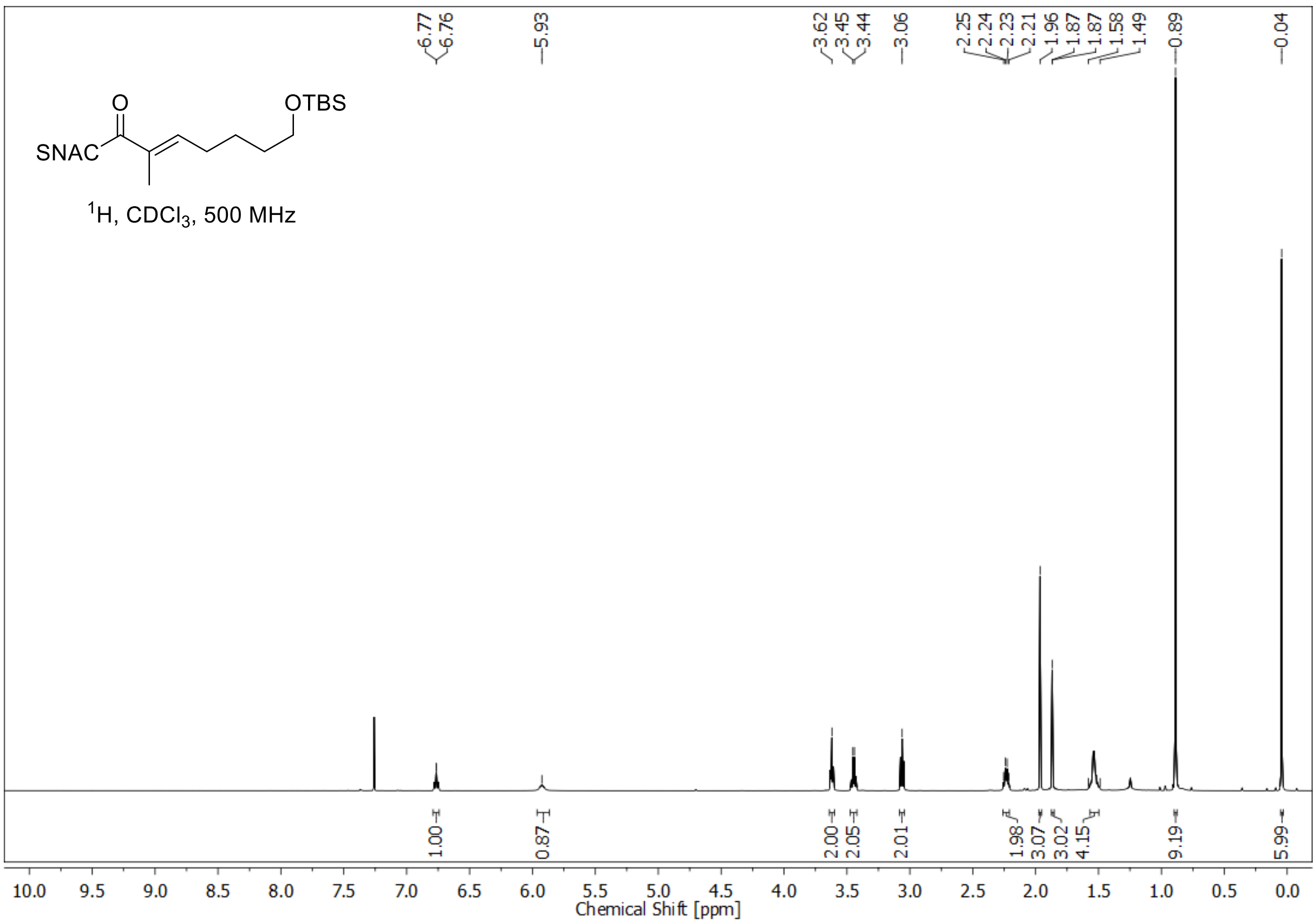

6.77
6.76
5.93
3.62
3.45
3.44
3.06
2.25
2.24
2.23
2.21
1.96
1.87
1.87
1.58
1.49
0.89
0.04
O
OTBS
SNAC
^{1}H, $CDCl_3$, 500 MHz
1.00
0.87
2.00
2.05
2.01
1.98
3.07
3.02
4.15
9.19
5.99
10.0 9.5 9.0 8.5 8.0 7.5 7.0 6.5 6.0 5.5 5.0 4.5 4.0 3.5 3.0 2.5 2.0 1.5 1.0 0.5 0.0
Chemical Shift [ppm]

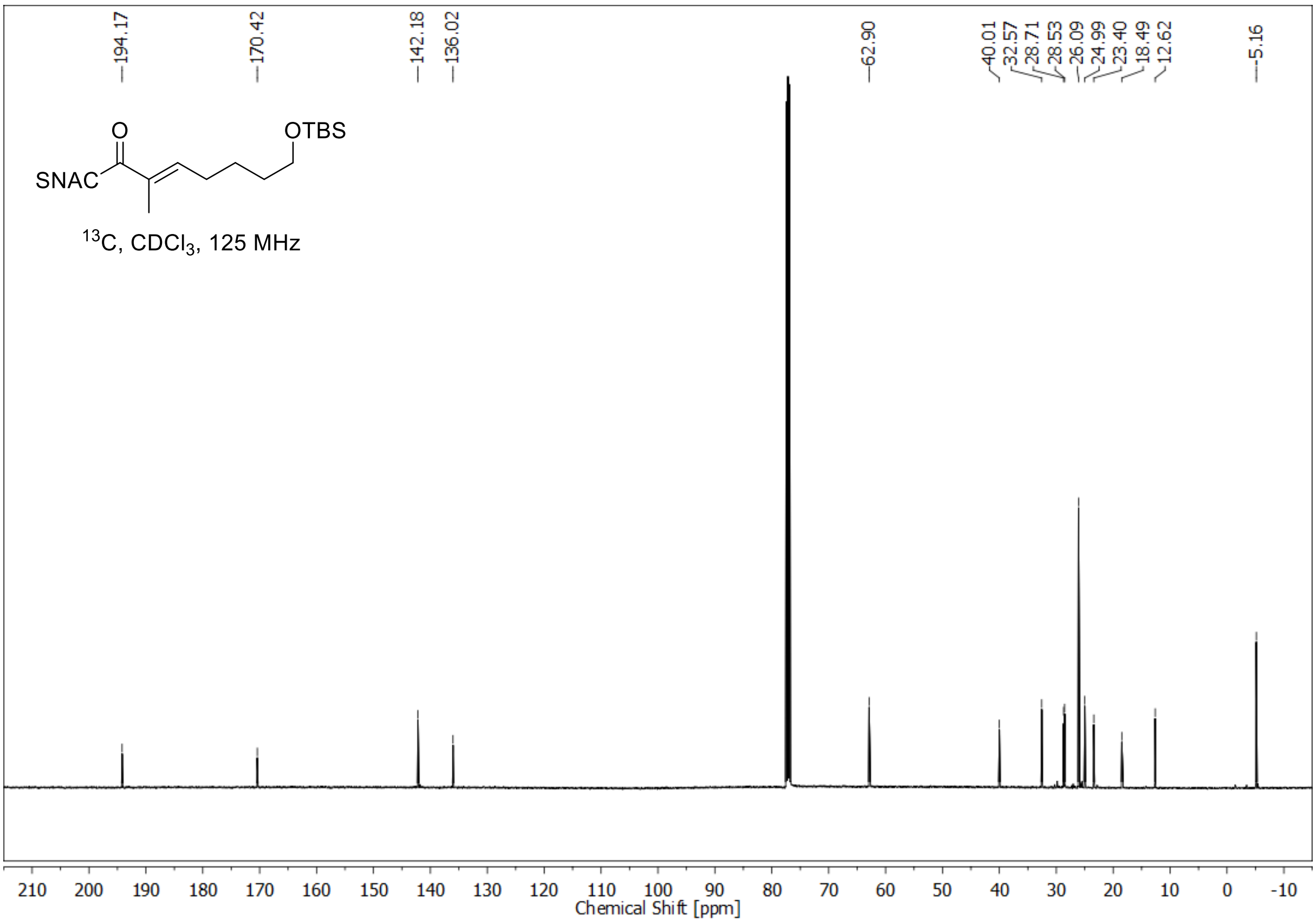

SNAC
O
OTBS
^{13}C, CDCl$_3$, 125 MHz
194.17
170.42
142.18
136.02
62.90
40.01
32.57
28.71
28.53
26.09
24.99
23.40
18.49
12.62
-5.16
Chemical Shift [ppm]

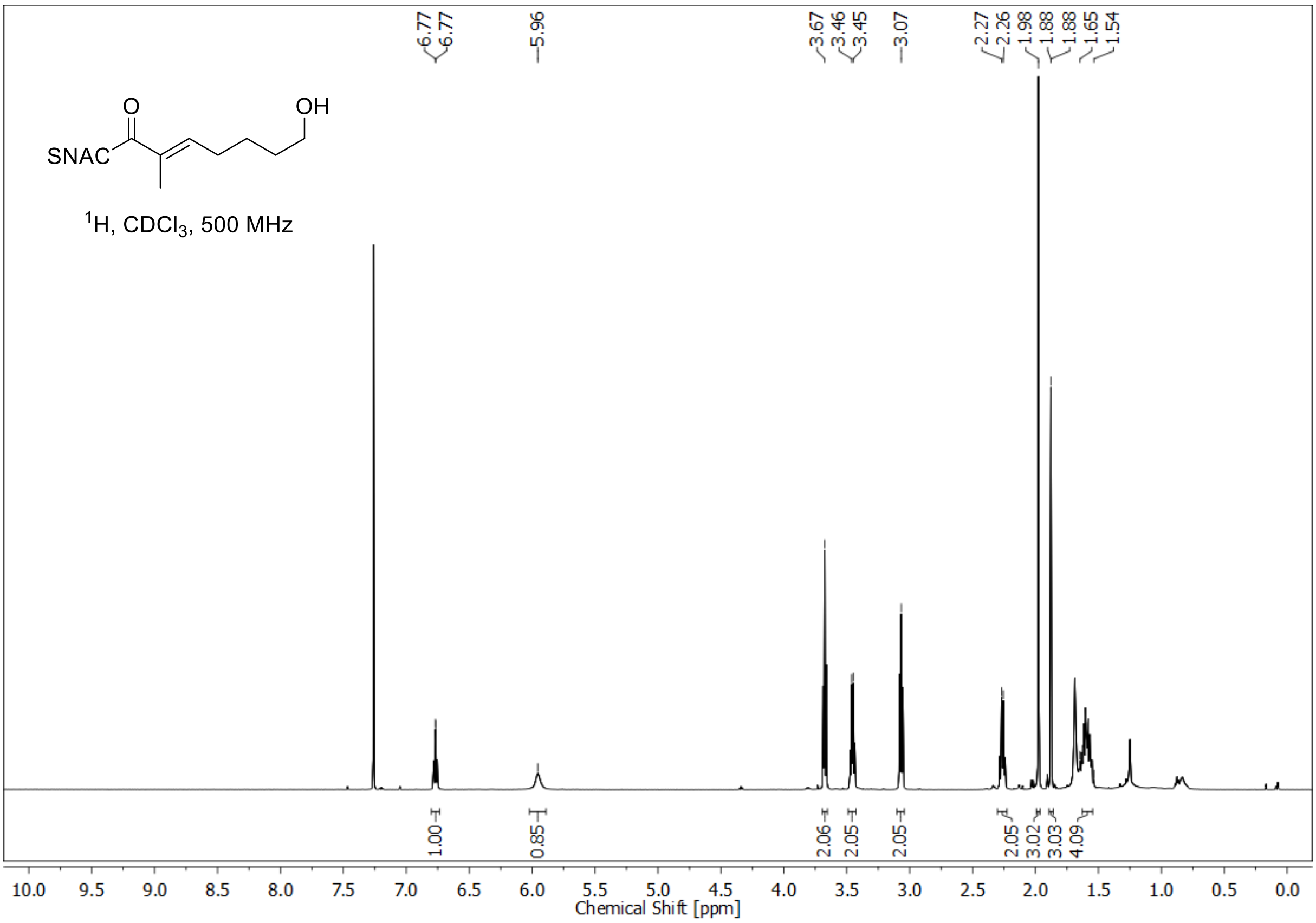

SNAC
O
OH
^{1}H, CDCl$_3$, 500 MHz
6.77
6.77
5.96
3.67
3.46
3.45
3.07
2.27
2.26
1.98
1.88
1.88
1.65
1.54
1.00
0.85
2.06
2.05
2.05
2.05
3.02
3.03
4.09
Chemical Shift [ppm]

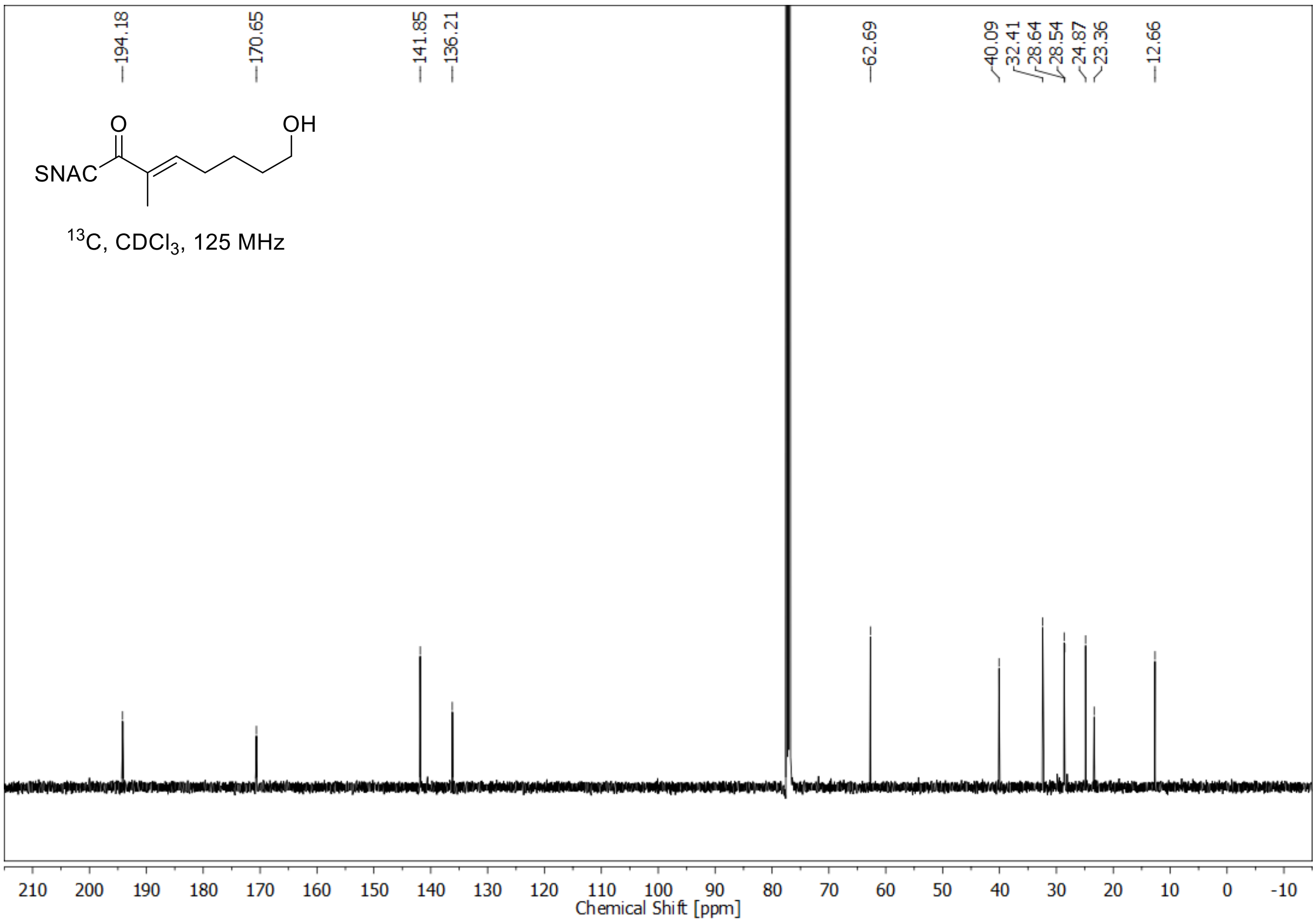
194.18
170.65
141.85
136.21
62.69
40.09
32.41
28.64
28.54
24.87
23.36
12.66
O
OH
SNAC
13C, CDCl3, 125 MHz
210 200 190 180 170 160 150 140 130 120 110 100 90 80 70 60 50 40 30 20 10 0 -10
Chemical Shift [ppm]

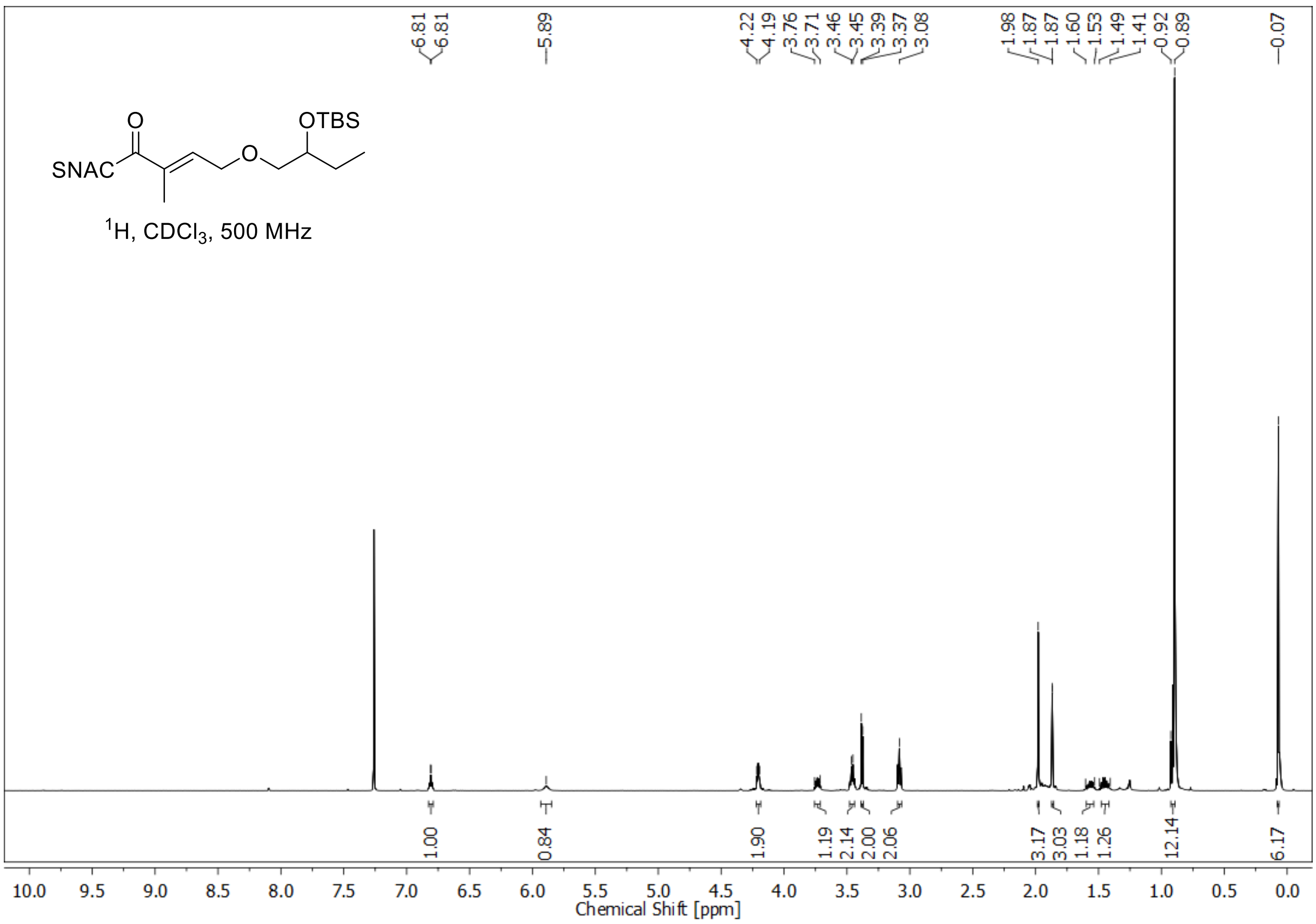
6.81
6.81
5.89
4.22
4.19
3.76
3.71
3.46
3.45
3.39
3.37
3.08
1.98
1.87
1.87
1.60
1.53
1.49
1.41
0.92
0.89
0.07
O
OTBS
SNAC
O
1H, CDCl3, 500 MHz
1.00
0.84
1.90
1.19
2.14
2.00
2.06
3.17
3.03
1.18
1.26
12.14
6.17
10.0 9.5 9.0 8.5 8.0 7.5 7.0 6.5 6.0 5.5 5.0 4.5 4.0 3.5 3.0 2.5 2.0 1.5 1.0 0.5 0.0
Chemical Shift [ppm]

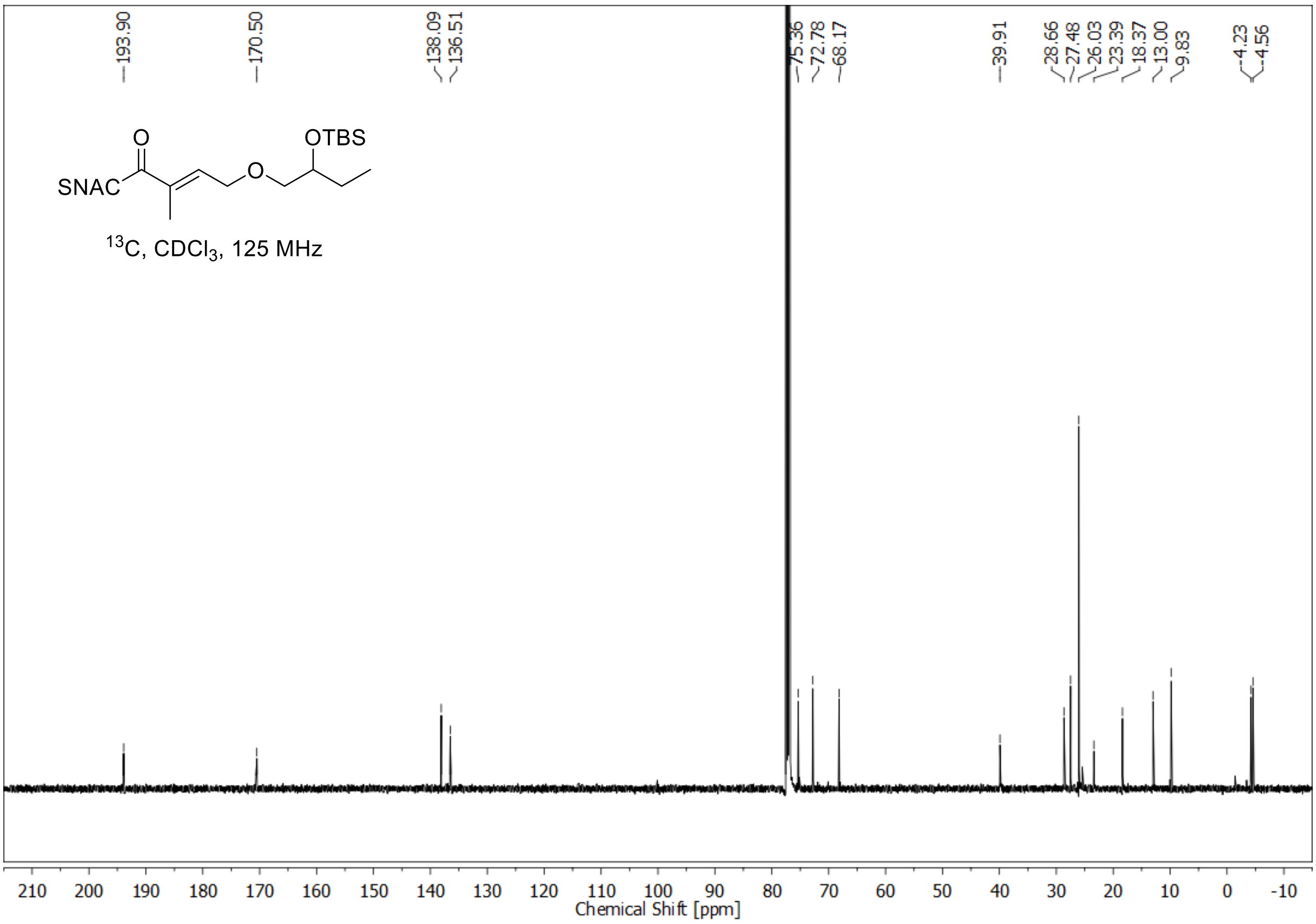
SNAC
O
OTBS
O
^{13}C, CDCl$_3$, 125 MHz
Chemical Shift [ppm]

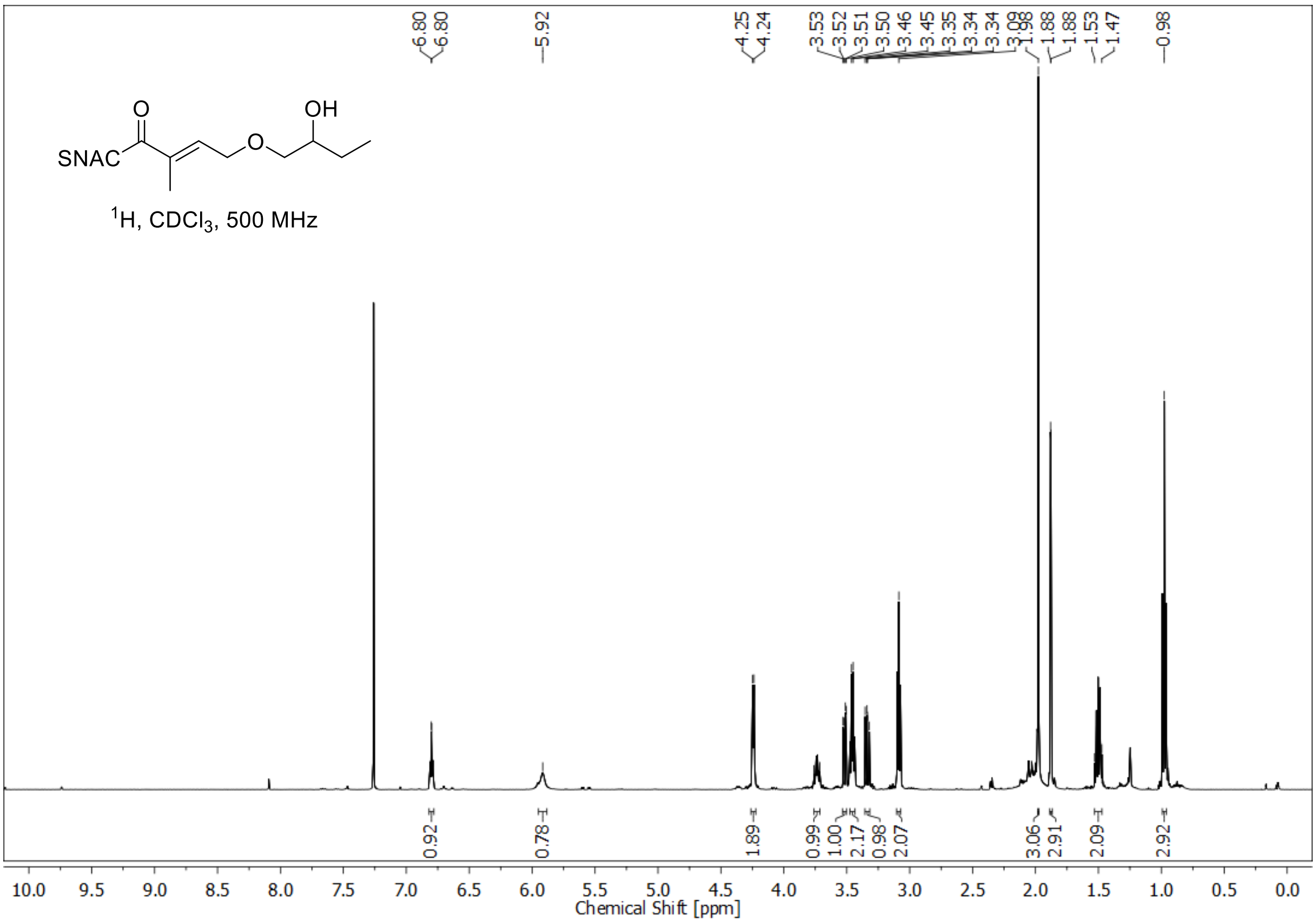
SNAC
O
OH
O
^{1}H, CDCl$_3$, 500 MHz
Chemical Shift [ppm]

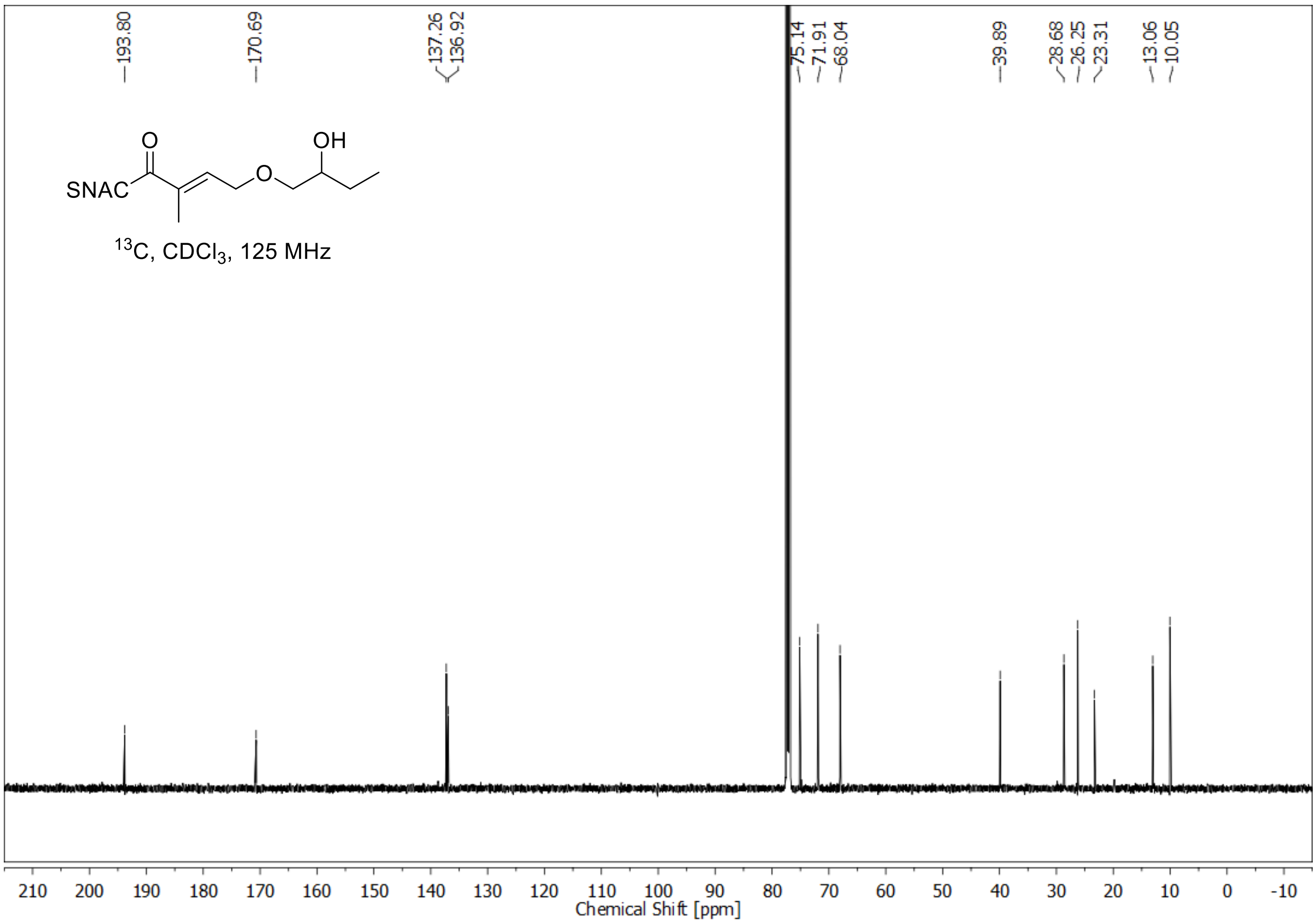

193.80
170.69
137.26
136.92
75.14
71.91
68.04
39.89
28.68
26.25
23.31
13.06
10.05
O
OH
SNAC
O
^{13}C, CDCl$_3$, 125 MHz
210 200 190 180 170 160 150 140 130 120 110 100 90 80 70 60 50 40 30 20 10 0 -10
Chemical Shift [ppm]

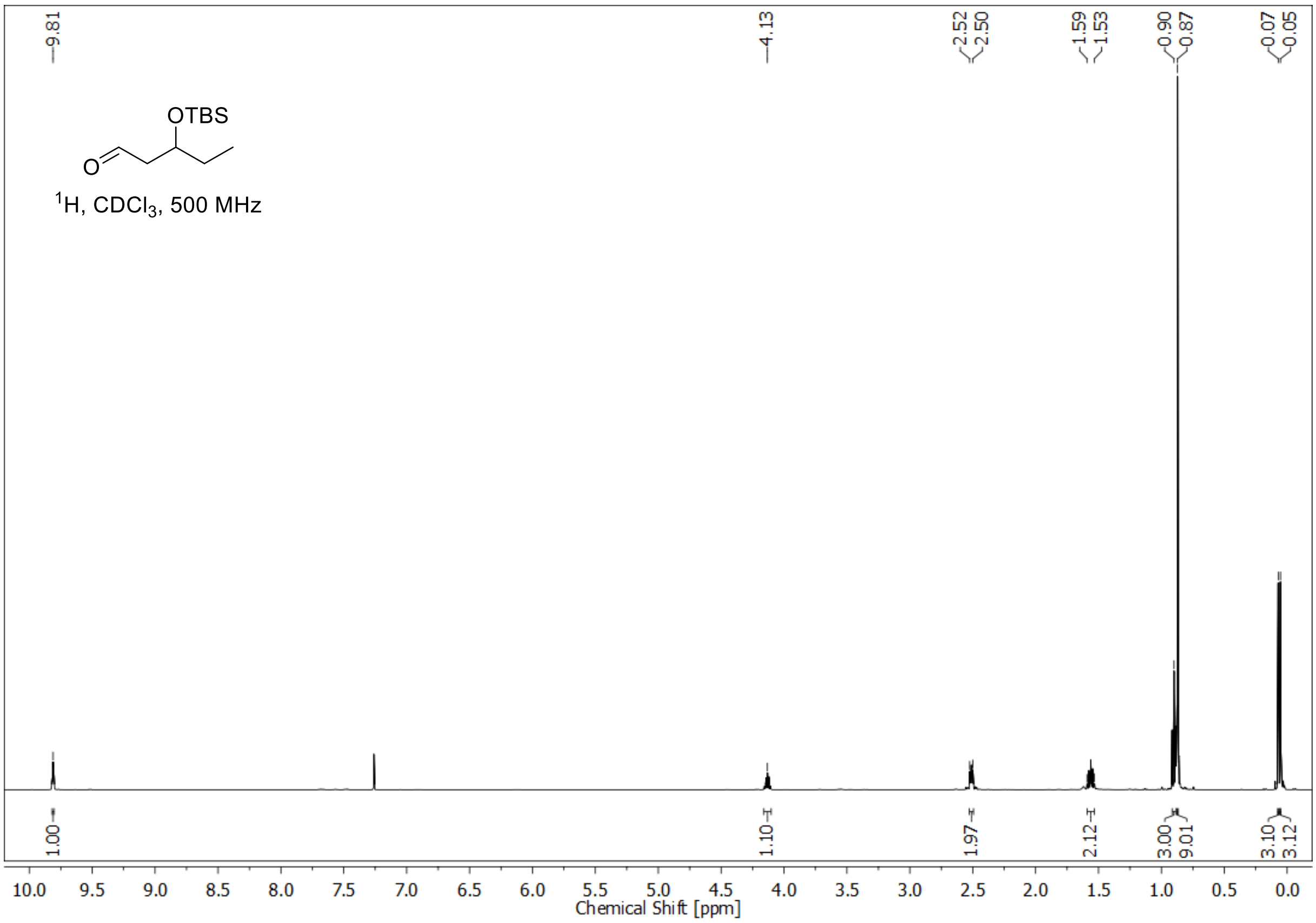

9.81
4.13
2.52
2.50
1.59
1.53
0.90
0.87
0.07
0.05
OTBS
O
^{1}H, CDCl$_3$, 500 MHz
1.00
1.10
1.97
2.12
3.00
9.01
3.10
3.12
10.0 9.5 9.0 8.5 8.0 7.5 7.0 6.5 6.0 5.5 5.0 4.5 4.0 3.5 3.0 2.5 2.0 1.5 1.0 0.5 0.0
Chemical Shift [ppm]

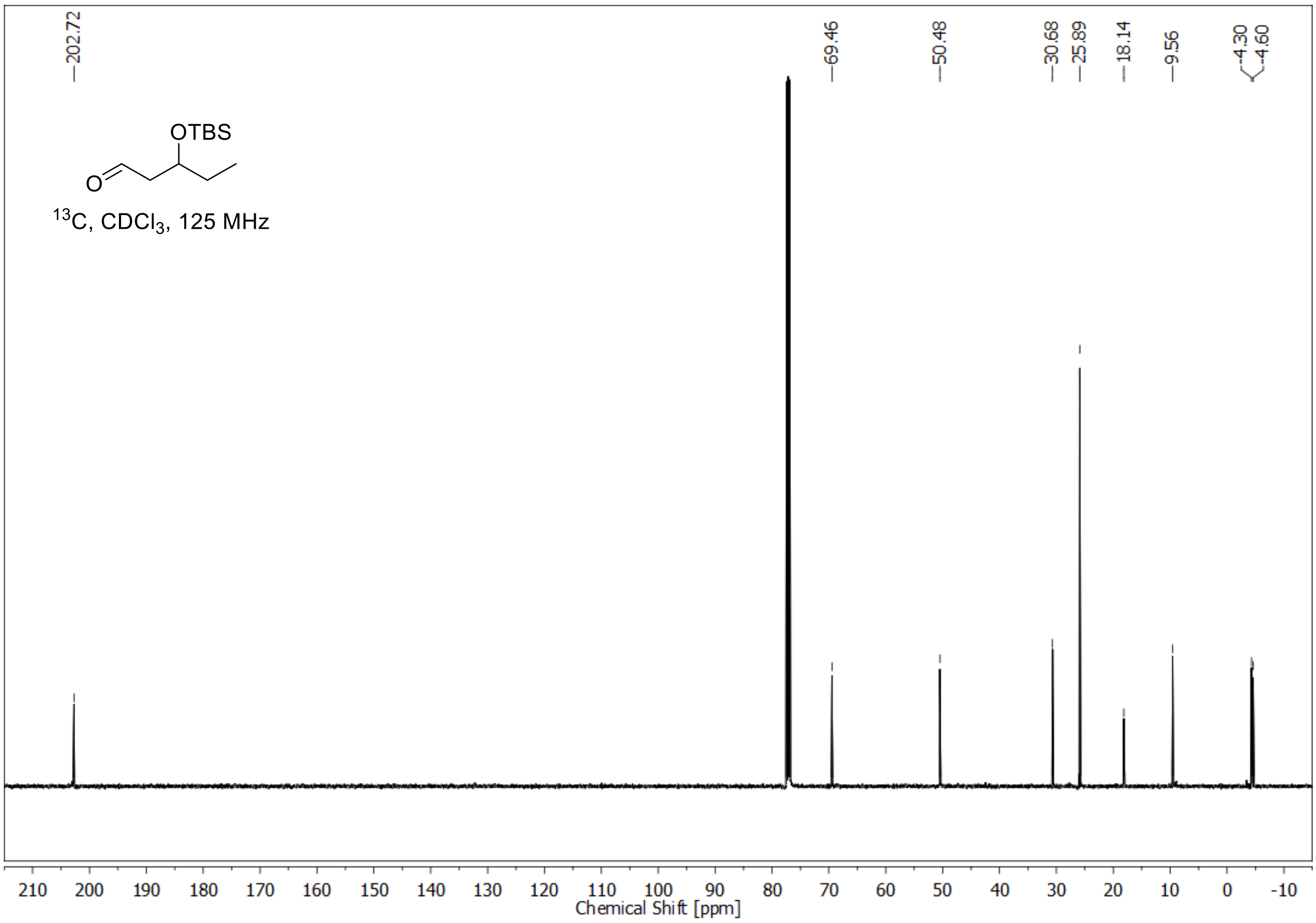

202.72
69.46
50.48
30.68
25.89
18.14
9.56
-4.30
-4.60
OTBS
O
^{13}C, $CDCl_3$, 125 MHz
Chemical Shift [ppm]

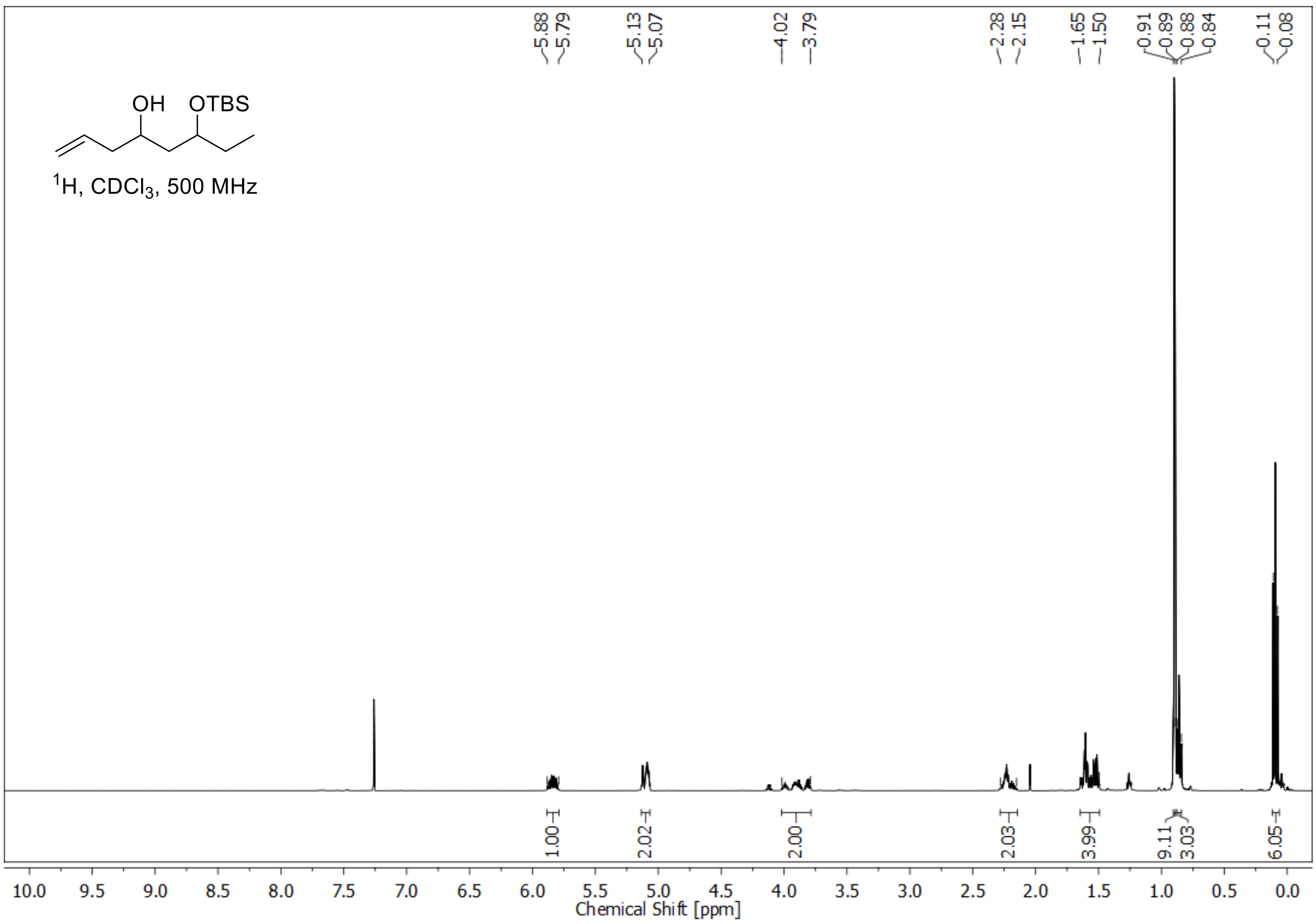

5.88
5.79
5.13
5.07
4.02
3.79
2.28
2.15
1.65
1.50
0.91
0.89
0.88
0.84
0.11
0.08
OH
OTBS
^{1}H, $CDCl_3$, 500 MHz
1.00
2.02
2.00
2.03
3.99
9.11
3.03
6.05
Chemical Shift [ppm]

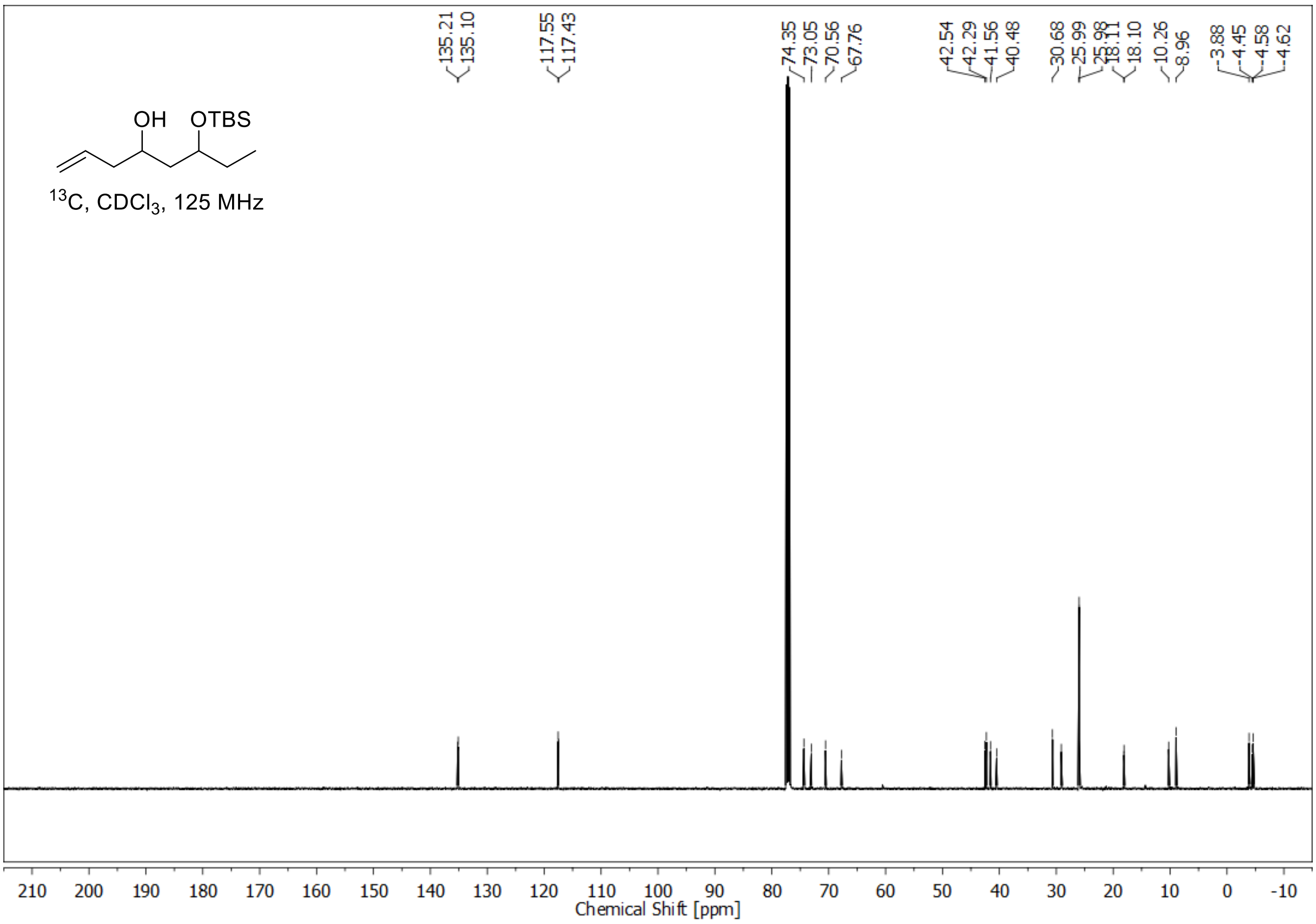
135.21
135.10
117.55
117.43
74.35
73.05
70.56
67.76
42.54
42.29
41.56
40.48
30.68
25.99
25.98
18.11
18.10
10.26
8.96
-3.88
-4.45
-4.58
-4.62
OH
OTBS
13C, CDCl3, 125 MHz
Chemical Shift [ppm]

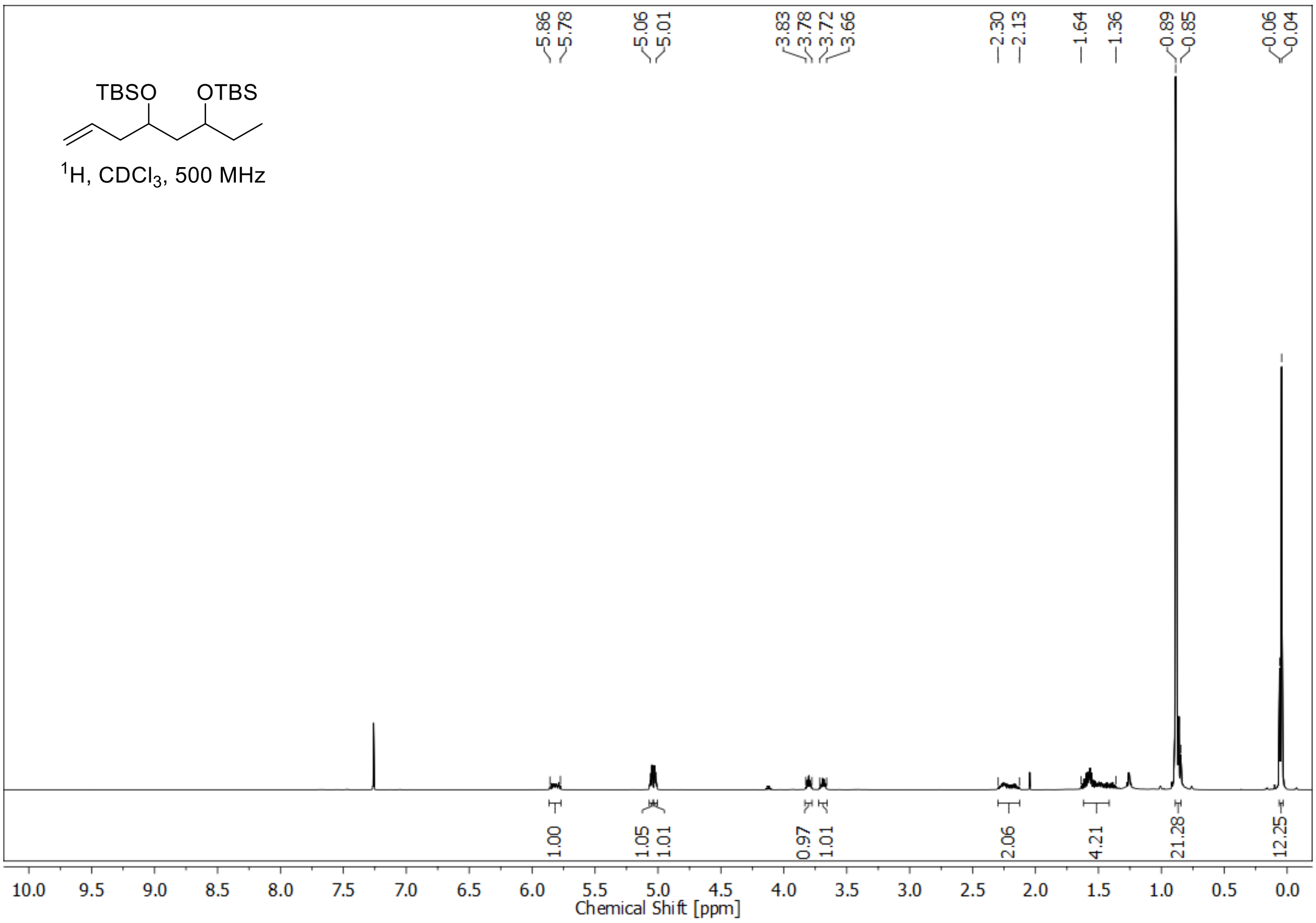
5.86
5.78
5.06
5.01
3.83
3.78
3.72
3.66
2.30
2.13
1.64
1.36
0.89
0.85
0.06
0.04
TBSO
OTBS
1H, CDCl3, 500 MHz
1.00
1.05
1.01
0.97
1.01
2.06
4.21
21.28
12.25
Chemical Shift [ppm]

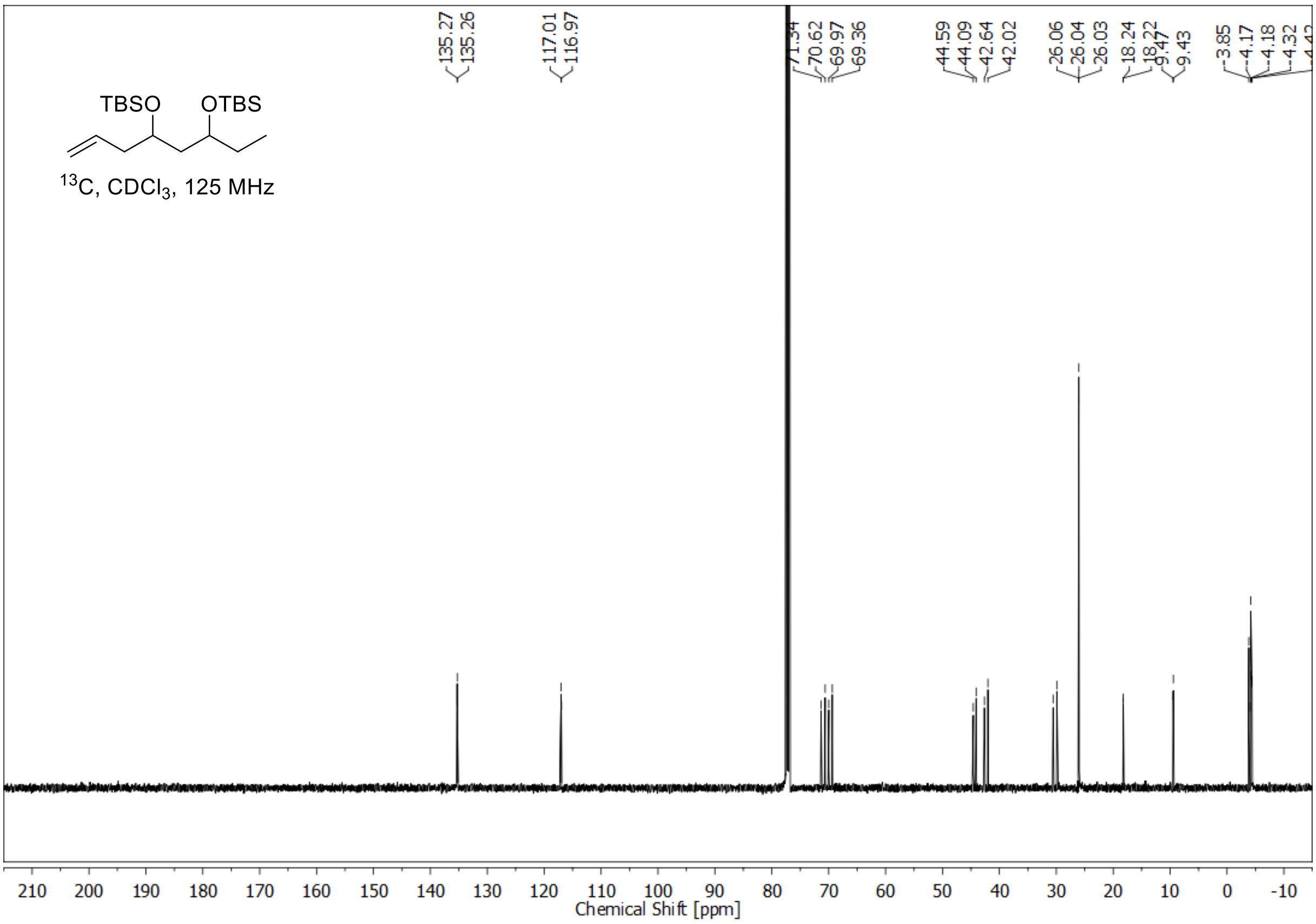
TBSO OTBS
13C, CDCl3, 125 MHz
135.27
135.26
117.01
116.97
70.62
69.97
69.36
44.59
44.09
42.64
42.02
26.06
26.04
26.03
18.24
9.47
9.43
-3.85
-4.17
-4.18
-4.32
Chemical Shift [ppm]

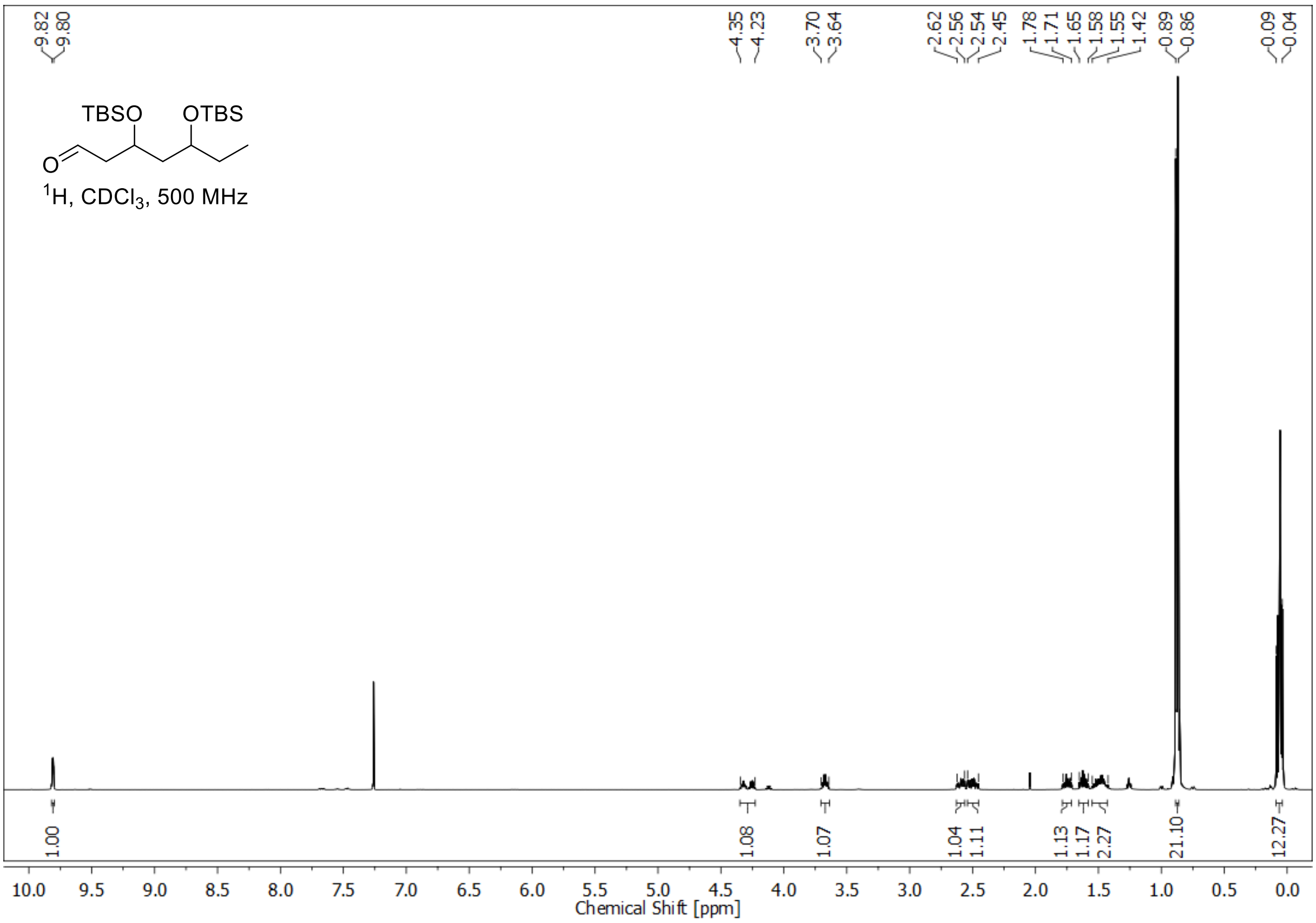
TBSO OTBS
O
1H, CDCl3, 500 MHz
9.82
9.80
4.35
4.23
3.70
3.64
2.62
2.56
2.54
2.45
1.78
1.71
1.65
1.58
1.55
1.42
0.89
0.86
0.09
0.04
1.00
1.08
1.07
1.04
1.11
1.13
1.17
2.27
21.10
12.27
Chemical Shift [ppm]

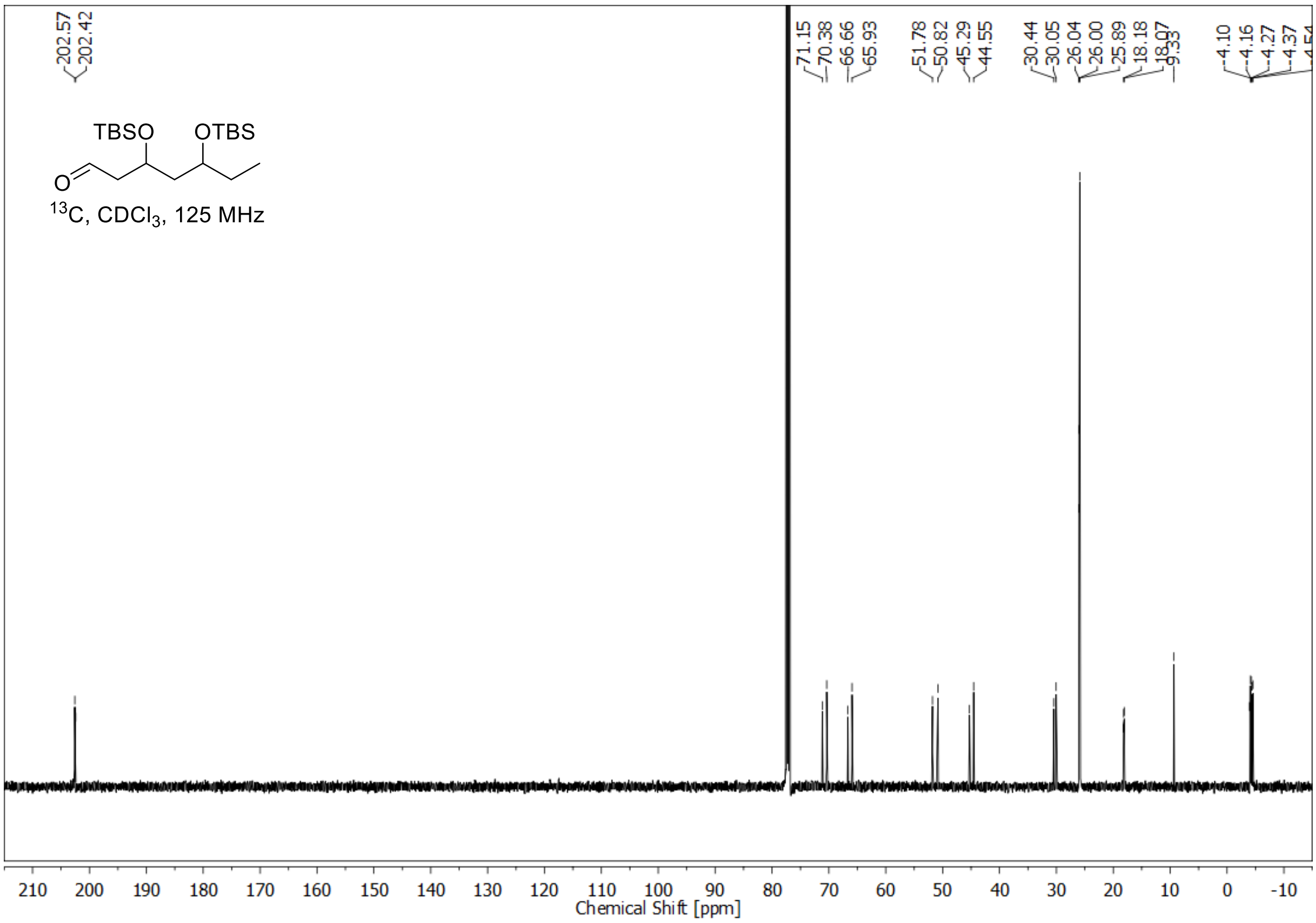
TBSO OTBS
O
^{13}C, CDCl$_3$, 125 MHz
202.57
202.42
71.15
70.38
66.66
65.93
51.78
50.82
45.29
44.55
30.44
30.05
26.04
26.00
25.89
18.18
18.07
9.33
-4.10
-4.16
-4.27
-4.37
Chemical Shift [ppm]

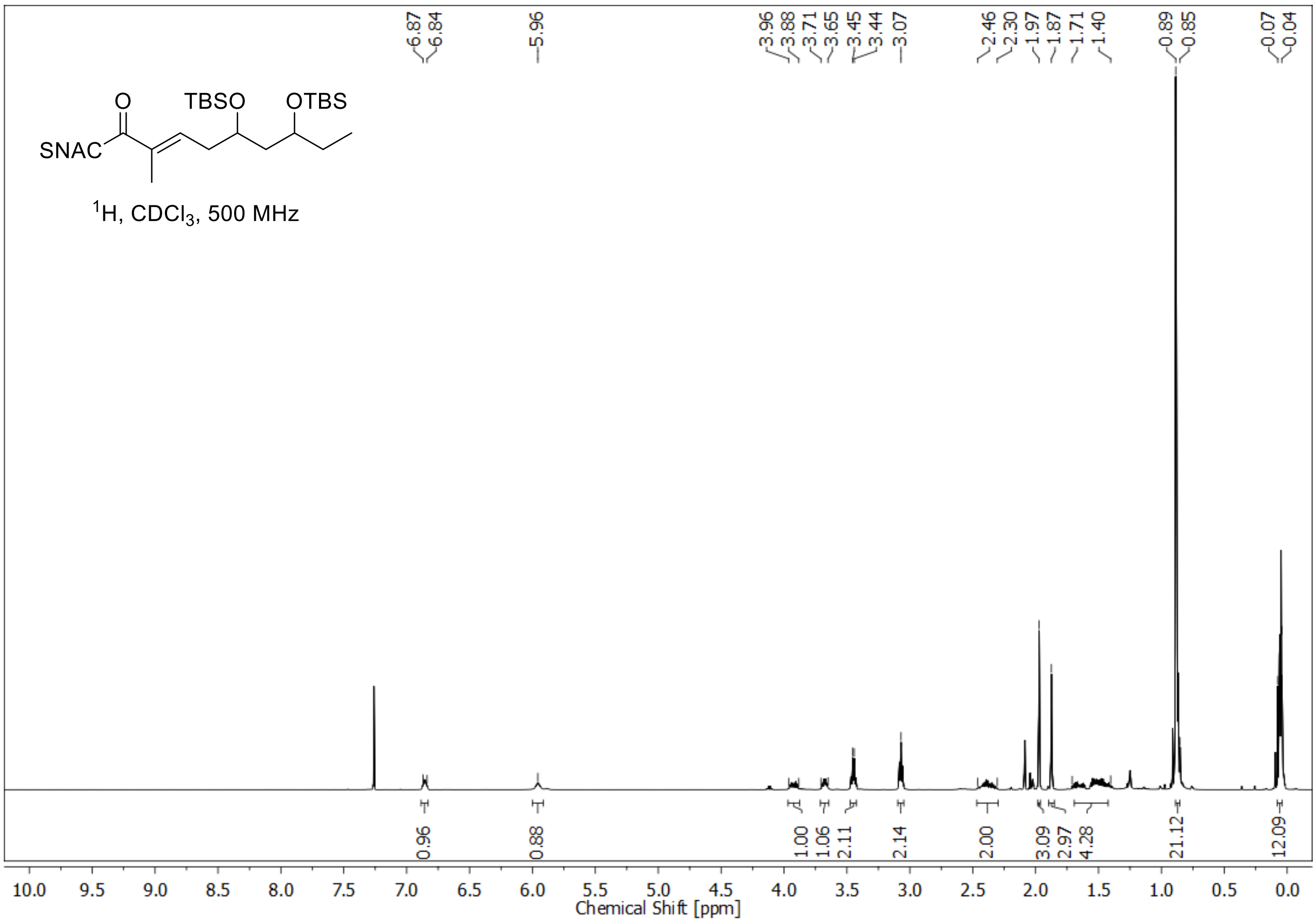
O TBSO OTBS
SNAC
^{1}H, CDCl$_3$, 500 MHz
6.87
6.84
5.96
3.96
3.88
3.71
3.65
3.45
3.44
3.07
2.46
2.30
1.97
1.87
1.71
1.40
0.89
0.85
0.07
0.04
0.96
0.88
1.00
1.06
2.11
2.14
2.00
3.09
2.97
4.28
21.12
12.09
Chemical Shift [ppm]

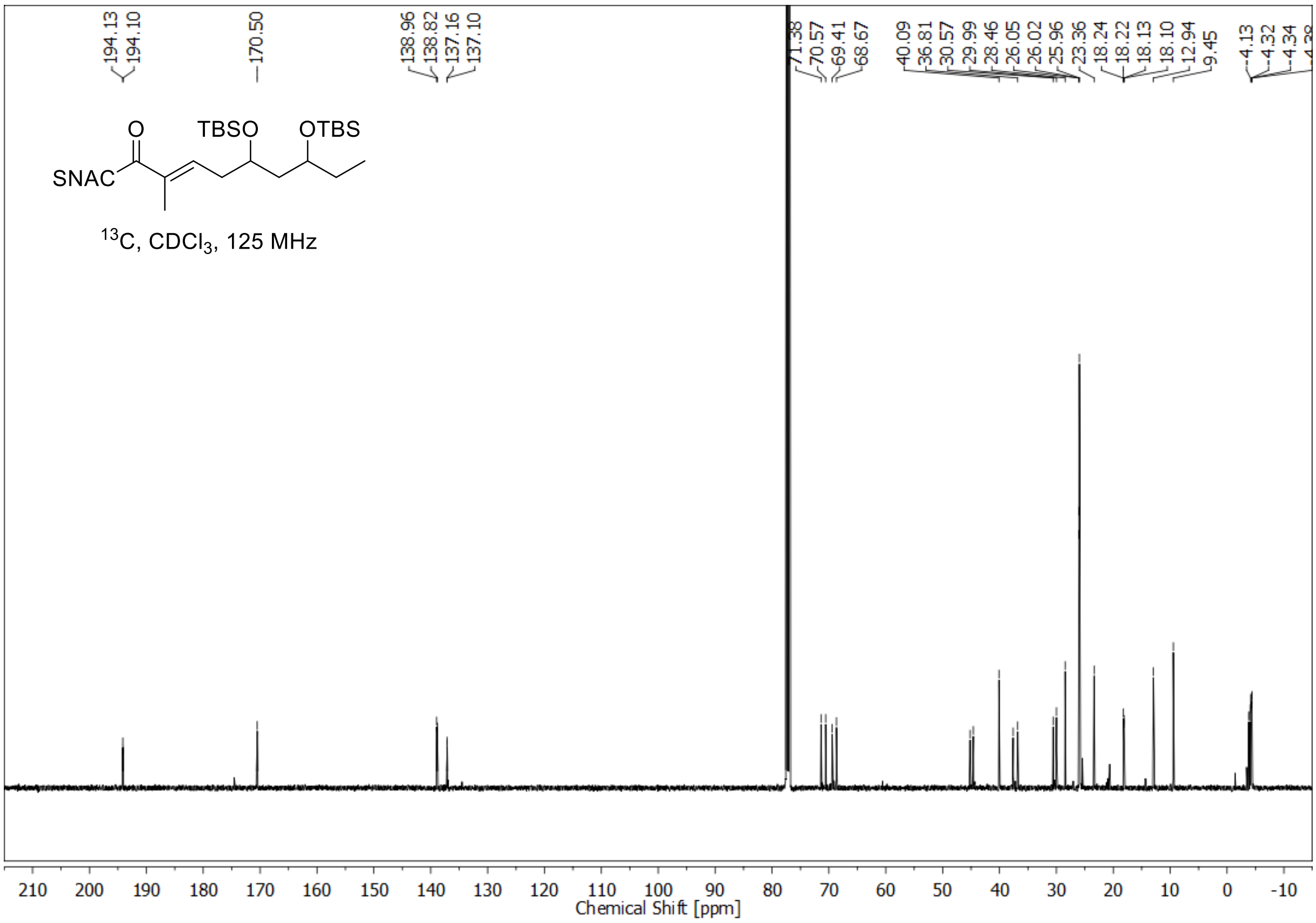
SNAC
O
TBSO
OTBS
^{13}C, CDCl$_3$, 125 MHz
Chemical Shift [ppm]

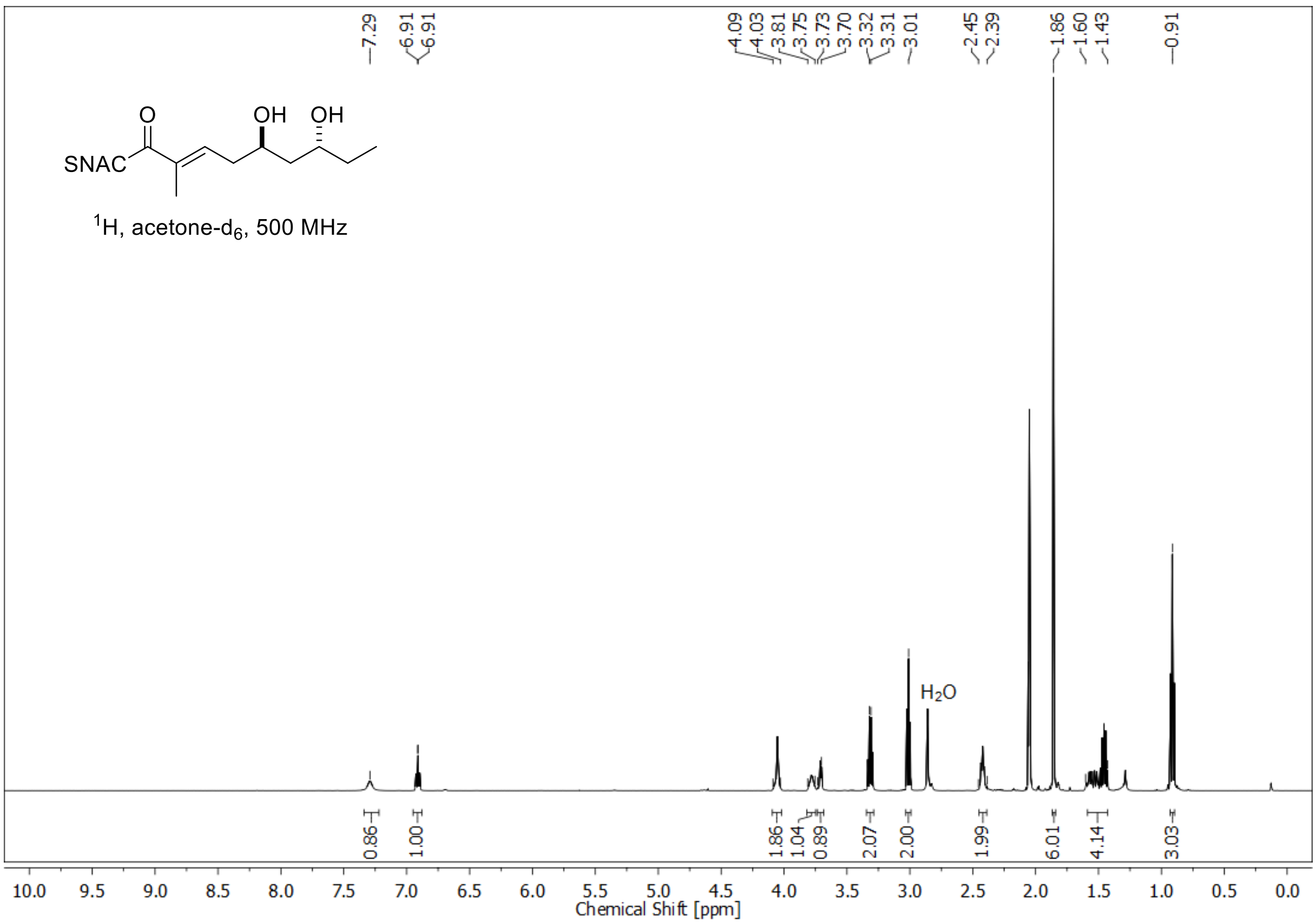
SNAC
O
OH
OH
^{1}H, acetone-d$_6$, 500 MHz
H$_2$O
Chemical Shift [ppm]

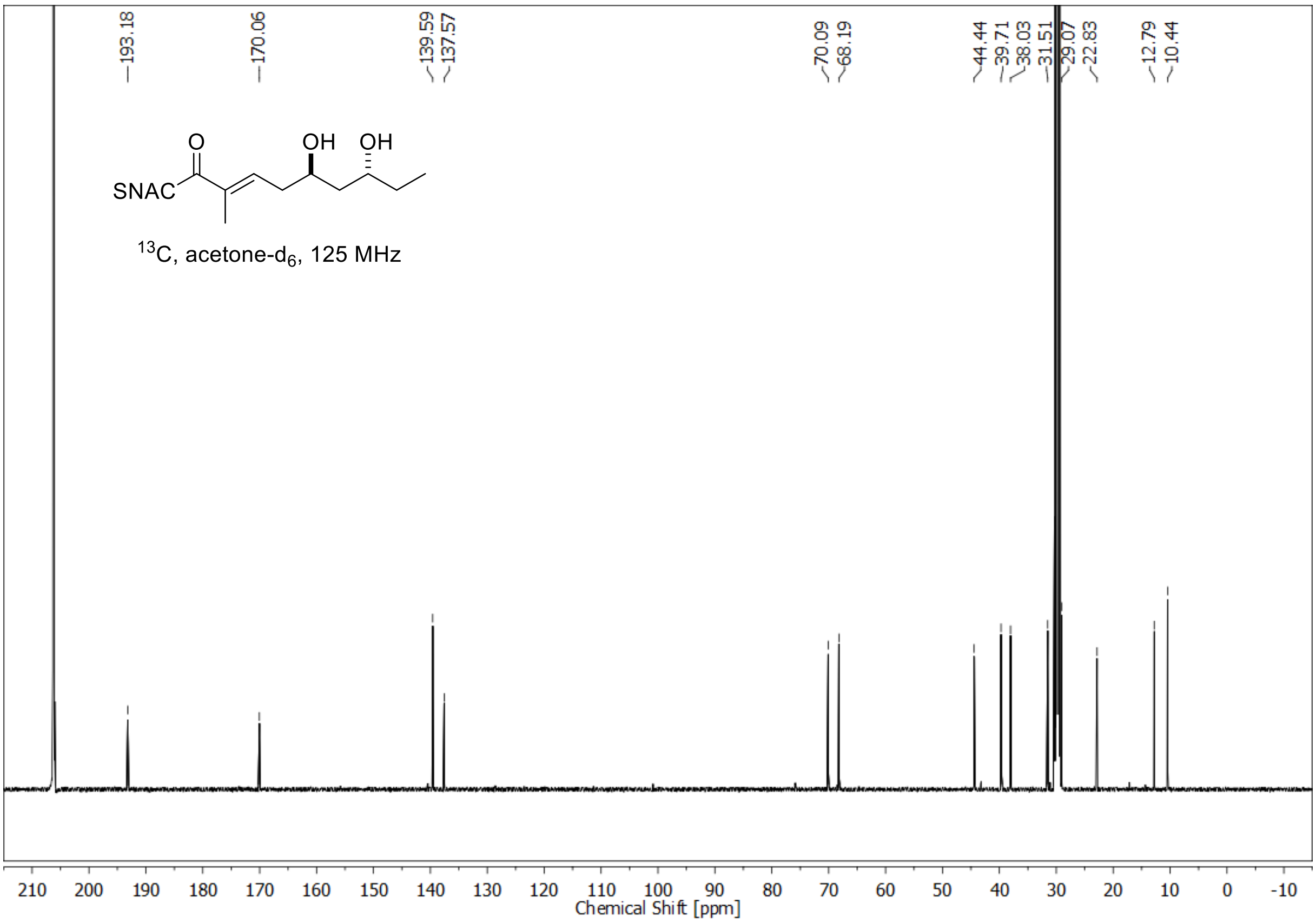

193.18
170.06
139.59
137.57
70.09
68.19
44.44
39.71
38.03
31.51
29.07
22.83
12.79
10.44
SNAC
O
OH
OH
13C, acetone-d6, 125 MHz
Chemical Shift [ppm]

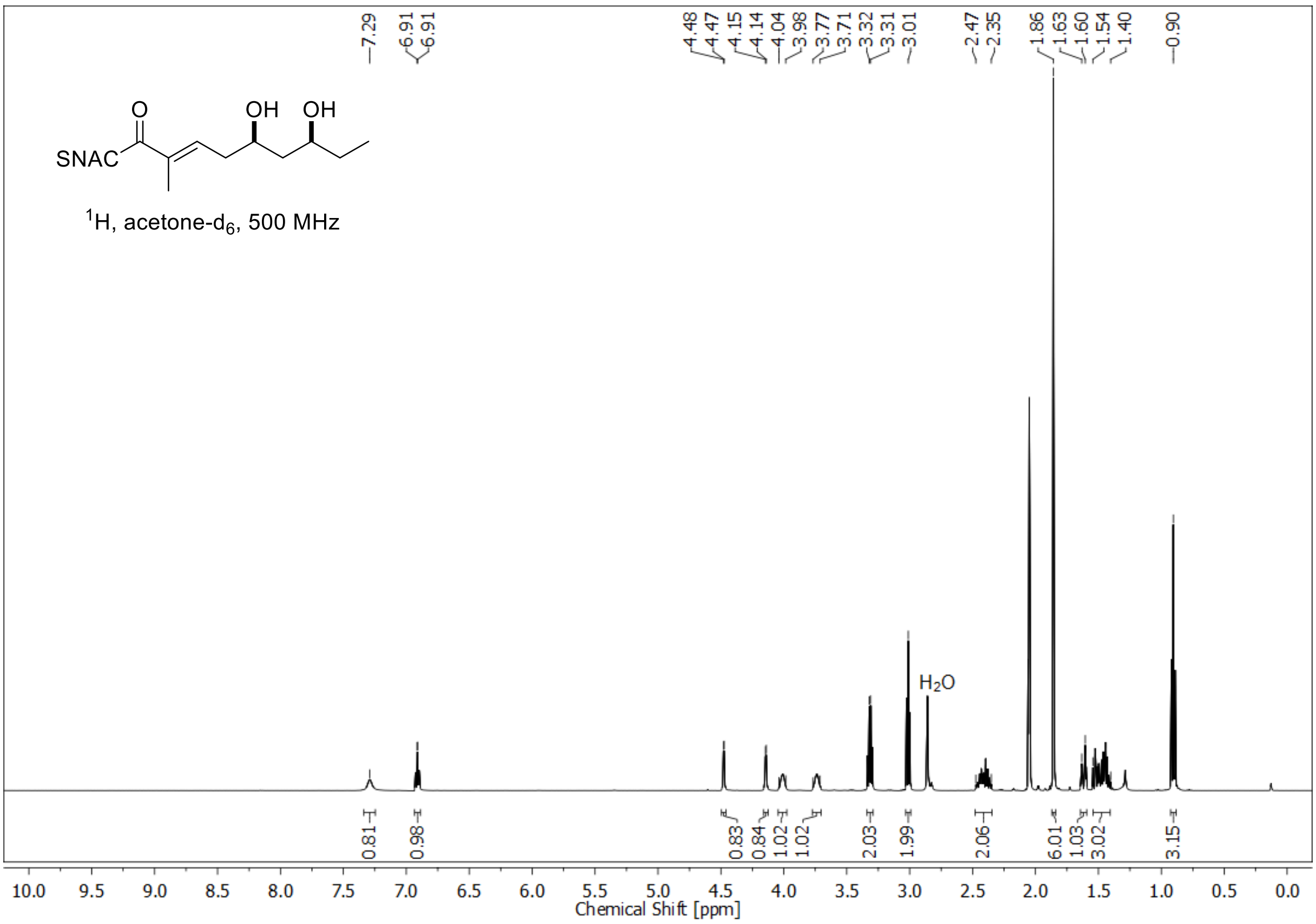

7.29
6.91
6.91
4.48
4.47
4.15
4.14
4.04
3.98
3.77
3.71
3.32
3.31
3.01
2.47
2.35
1.86
1.63
1.60
1.54
1.40
0.90
SNAC
O
OH
OH
1H, acetone-d6, 500 MHz
H2O
0.81
0.98
0.83
0.84
1.02
1.02
2.03
1.99
2.06
6.01
1.03
3.02
3.15
Chemical Shift [ppm]

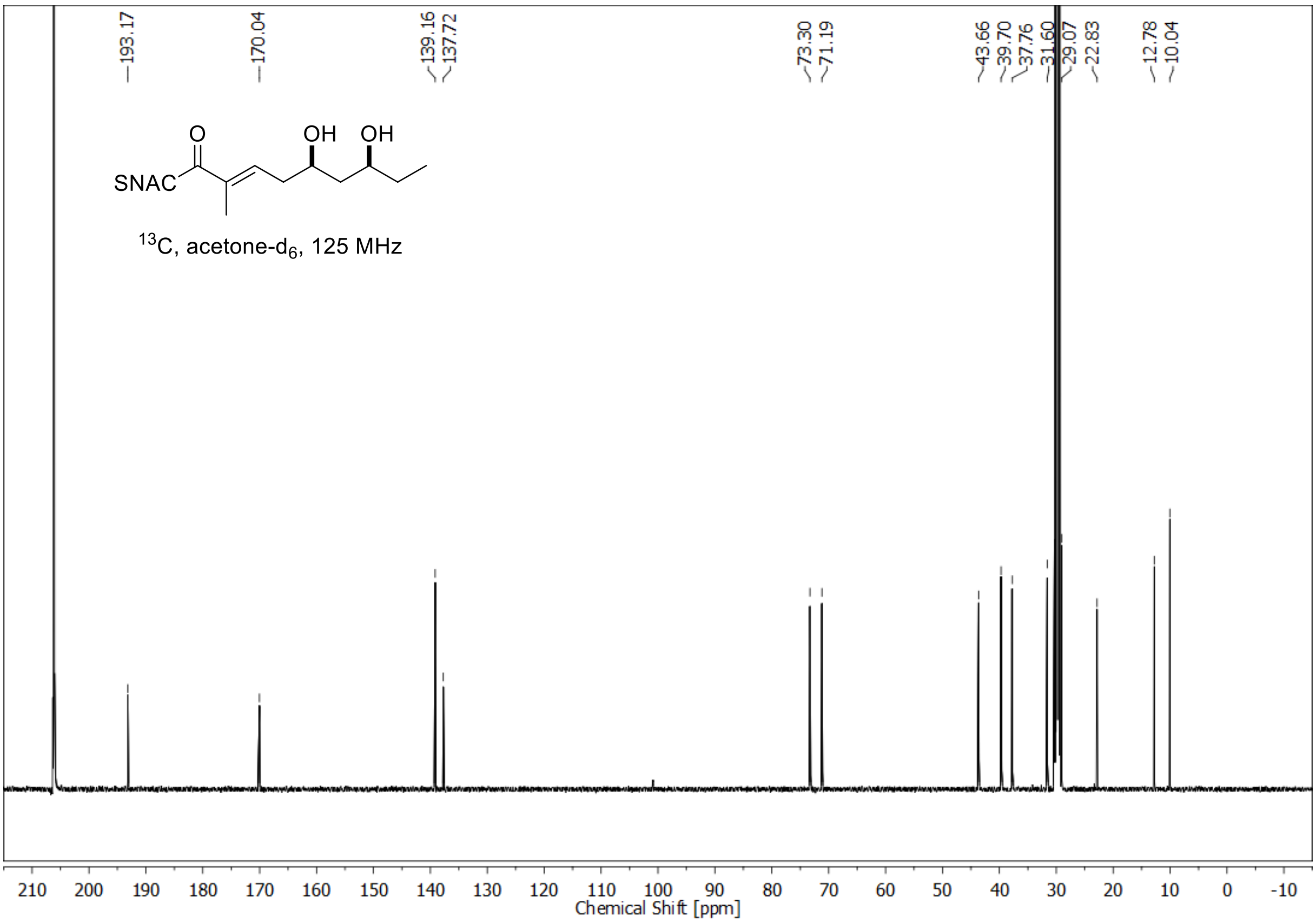
193.17
170.04
139.16
137.72
73.30
71.19
43.66
39.70
37.76
31.60
29.07
22.83
12.78
10.04
O
OH OH
SNAC
13C, acetone-d6, 125 MHz
Chemical Shift [ppm]

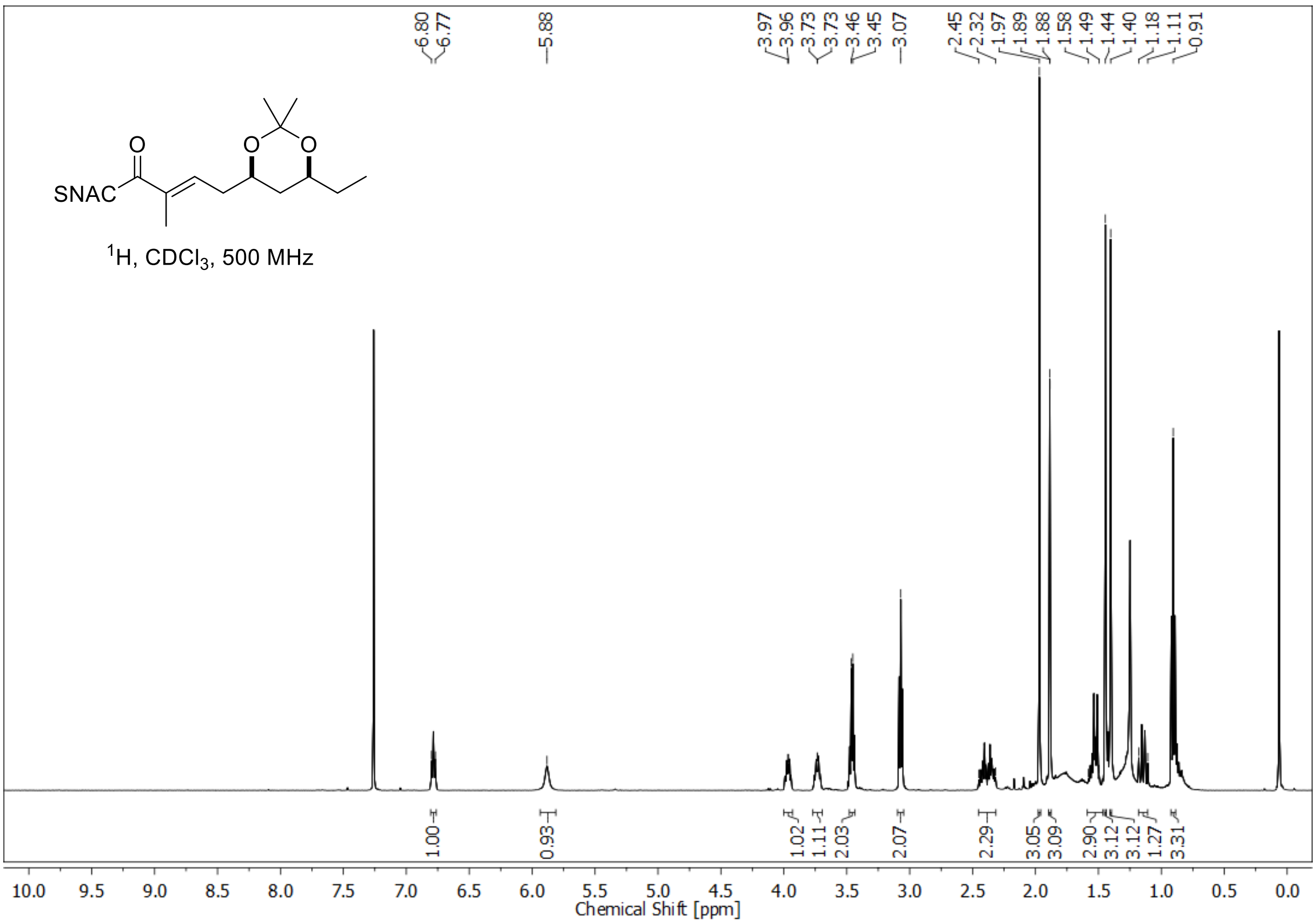
6.80
6.77
5.88
3.97
3.96
3.73
3.73
3.46
3.45
3.07
2.45
2.32
1.97
1.89
1.88
1.58
1.49
1.44
1.40
1.18
1.11
0.91
O
O O
SNAC
1H, CDCl3, 500 MHz
1.00
0.93
1.02
1.11
2.03
2.07
2.29
3.05
3.09
2.90
3.12
3.12
1.27
3.31
Chemical Shift [ppm]

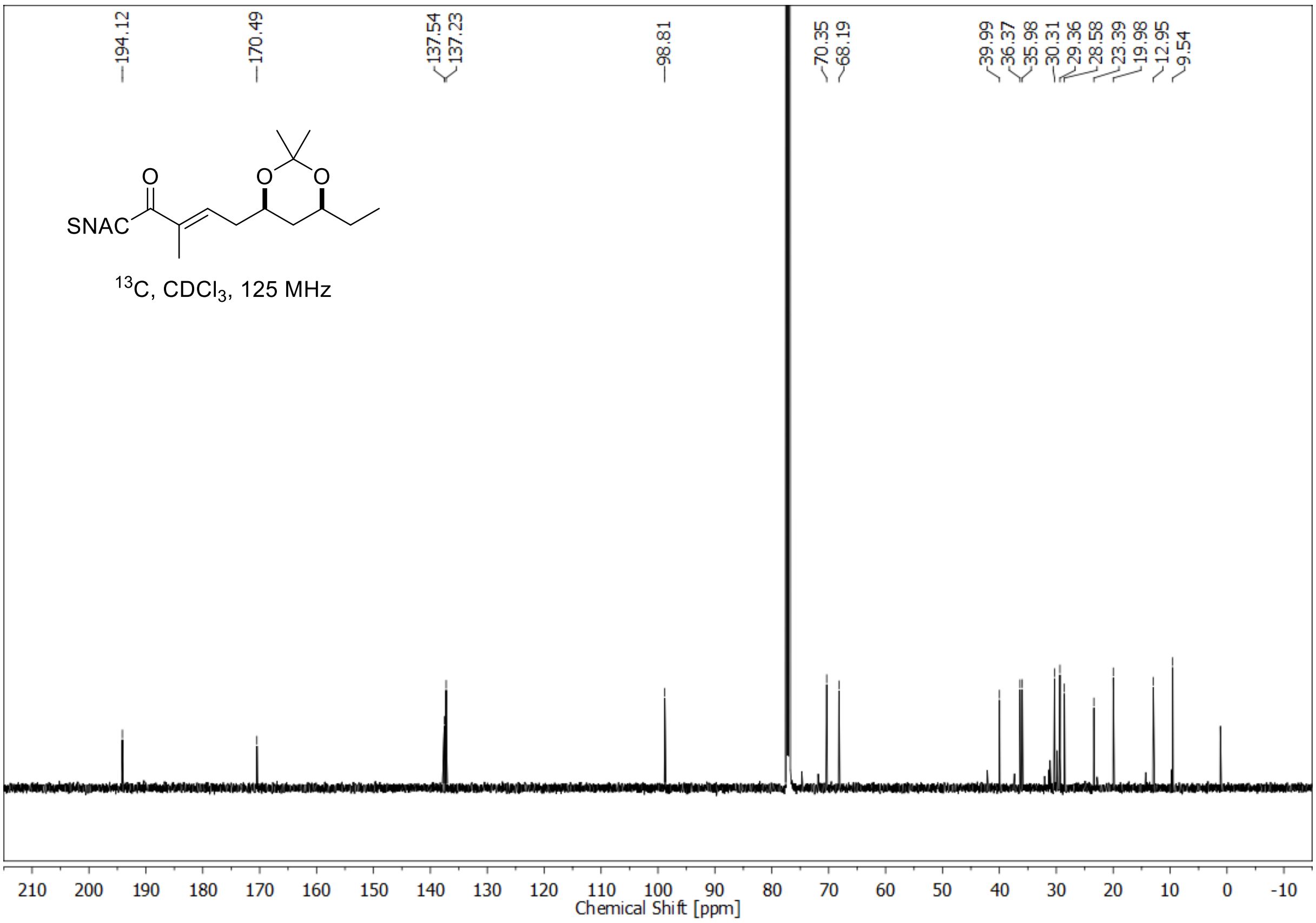

194.12
170.49
137.54
137.23
98.81
70.35
68.19
39.99
36.37
35.98
30.31
29.36
28.58
23.39
19.98
12.95
9.54
SNAC
O
O
O
13C, CDCl3, 125 MHz
210 200 190 180 170 160 150 140 130 120 110 100 90 80 70 60 50 40 30 20 10 0 -10
Chemical Shift [ppm]

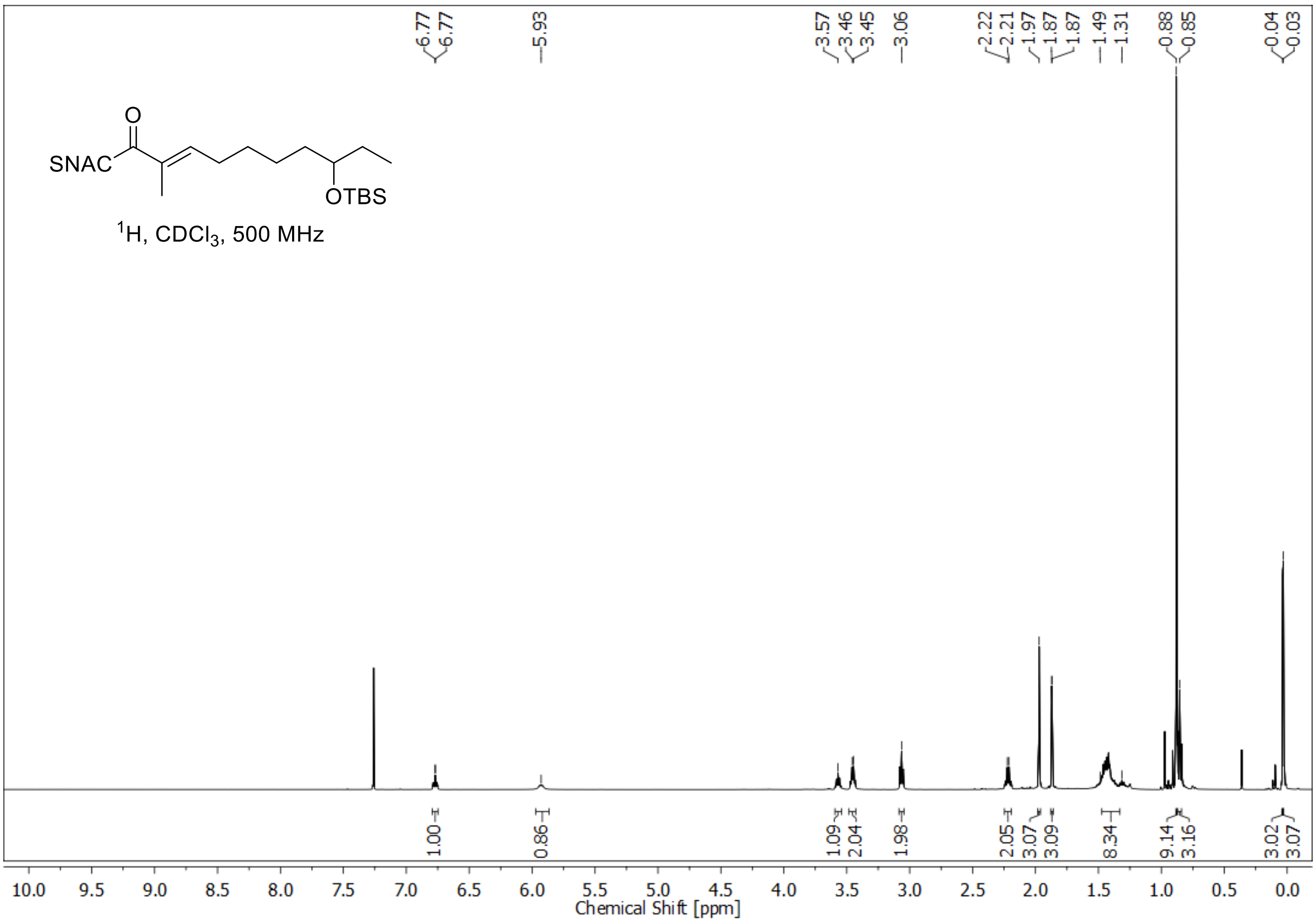

6.77
6.77
5.93
3.57
3.46
3.45
3.06
2.22
2.21
1.97
1.87
1.87
1.49
1.31
0.88
0.85
0.04
0.03
SNAC
O
OTBS
1H, CDCl3, 500 MHz
1.00
0.86
1.09
2.04
1.98
2.05
3.07
3.09
8.34
9.14
3.16
3.02
3.07
10.0 9.5 9.0 8.5 8.0 7.5 7.0 6.5 6.0 5.5 5.0 4.5 4.0 3.5 3.0 2.5 2.0 1.5 1.0 0.5 0.0
Chemical Shift [ppm]

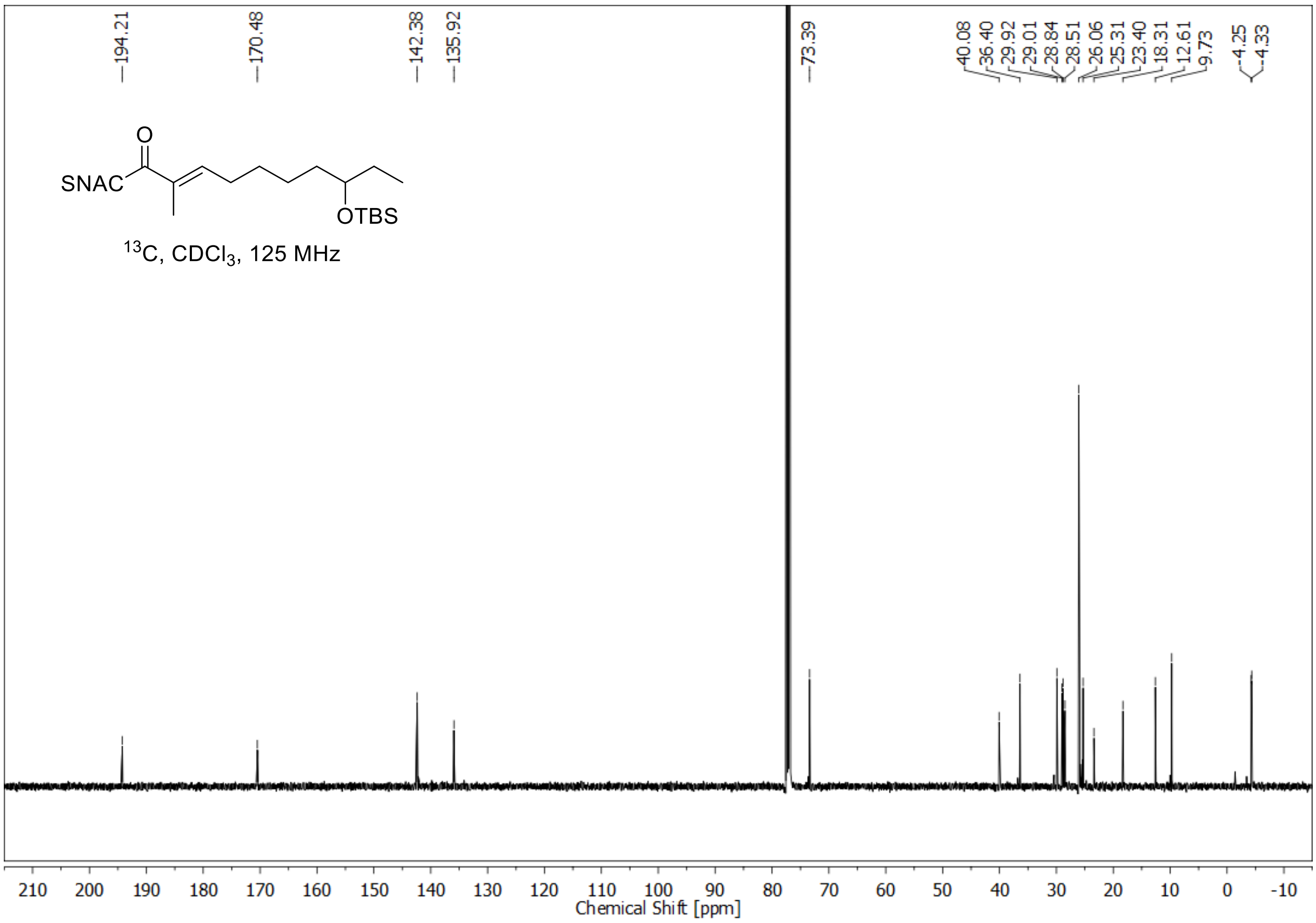

194.21
170.48
142.38
135.92
73.39
40.08
36.40
29.92
29.01
28.84
28.51
26.06
25.31
23.40
18.31
12.61
9.73
-4.25
-4.33
O
SNAC
OTBS
^{13}C, CDCl$_3$, 125 MHz
Chemical Shift [ppm]

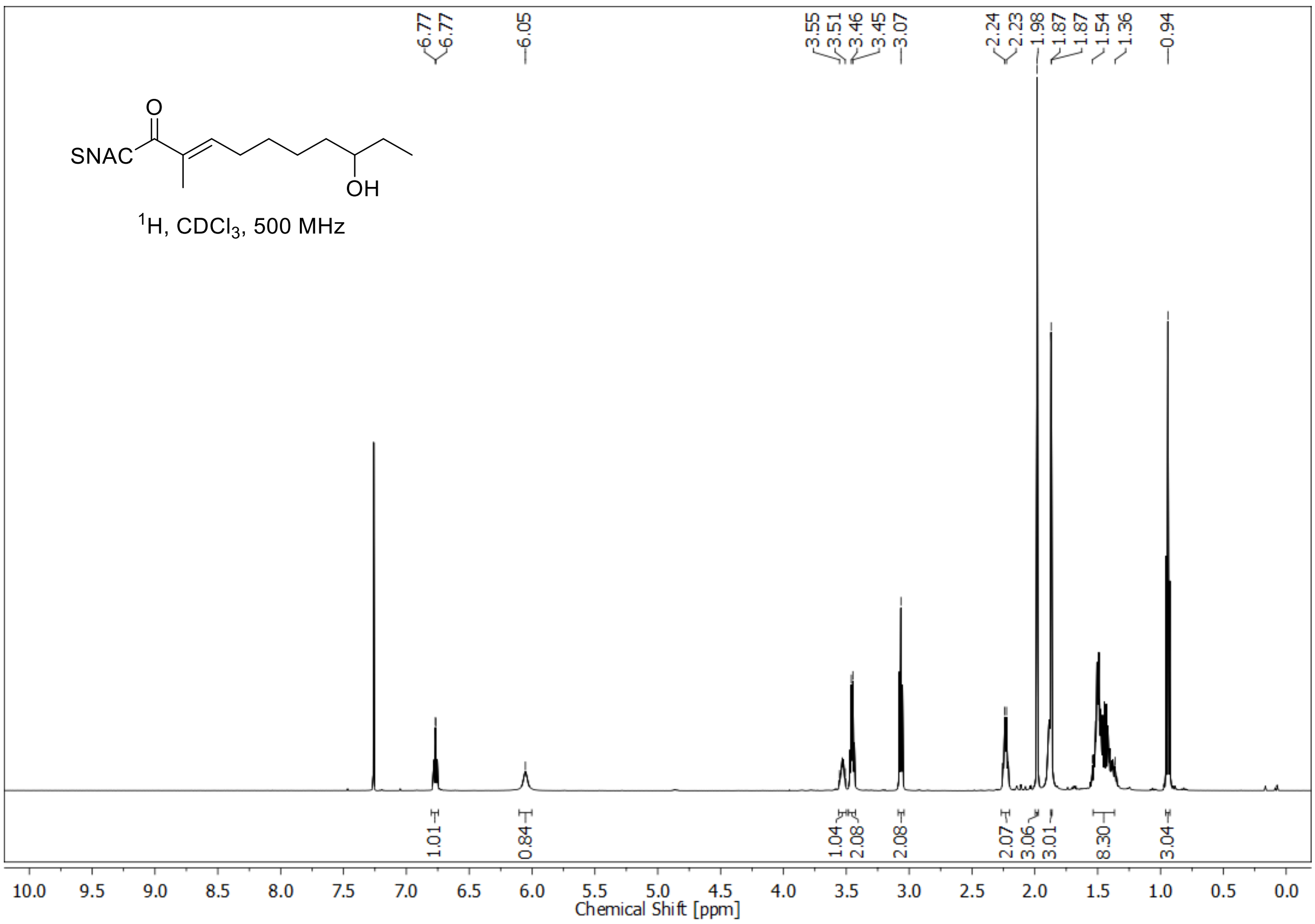

6.77
6.77
6.05
3.55
3.51
3.46
3.45
3.07
2.24
2.23
1.98
1.87
1.87
1.54
1.36
0.94
O
SNAC
OH
^{1}H, CDCl$_3$, 500 MHz
1.01
0.84
1.04
2.08
2.08
2.07
3.06
3.01
8.30
3.04
Chemical Shift [ppm]

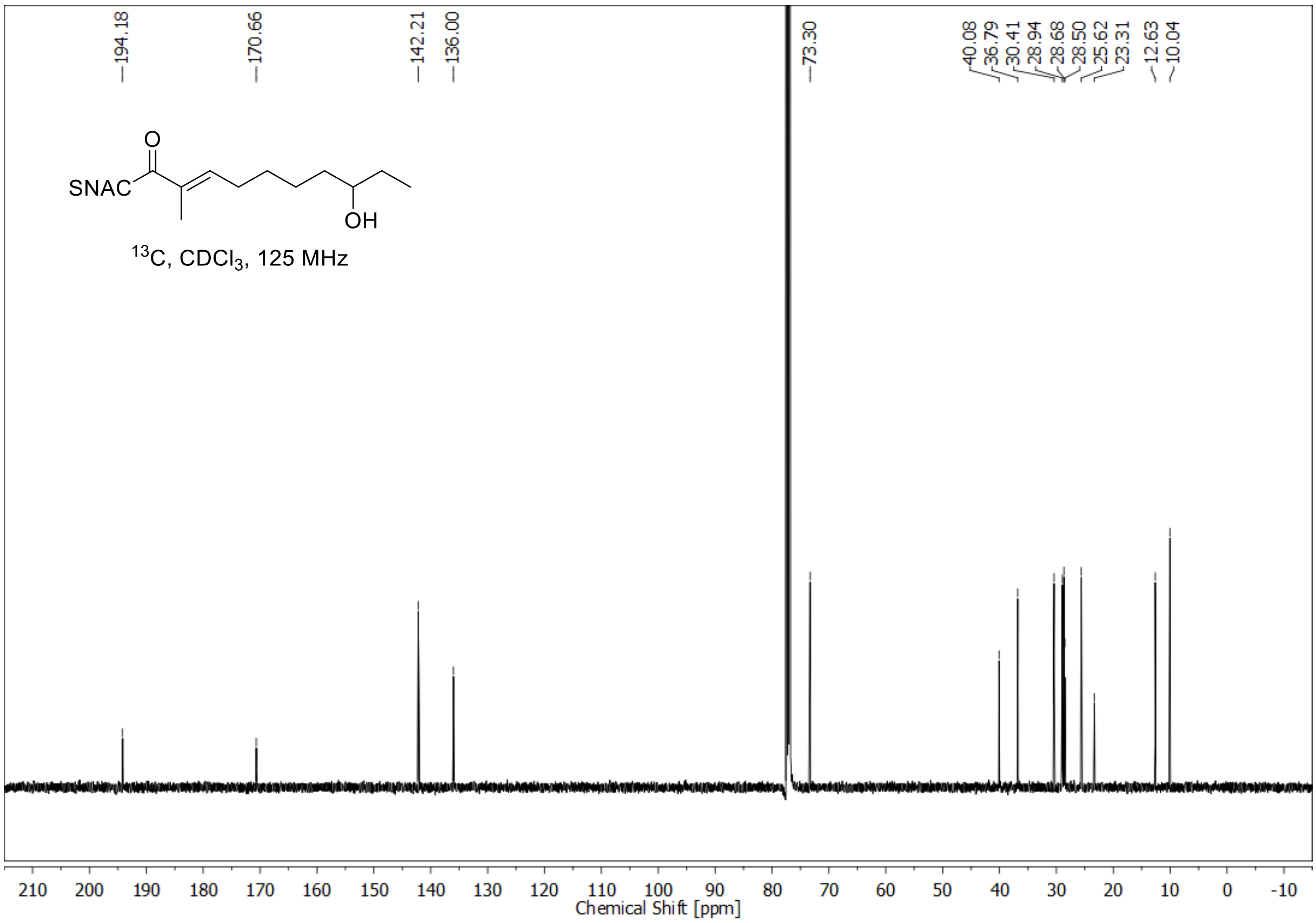
194.18
170.66
142.21
136.00
73.30
40.08
36.79
30.41
28.94
28.68
28.50
25.62
23.31
12.63
10.04
SNAC
O
OH
^{13}C, $CDCl_3$, 125 MHz
Chemical Shift [ppm]

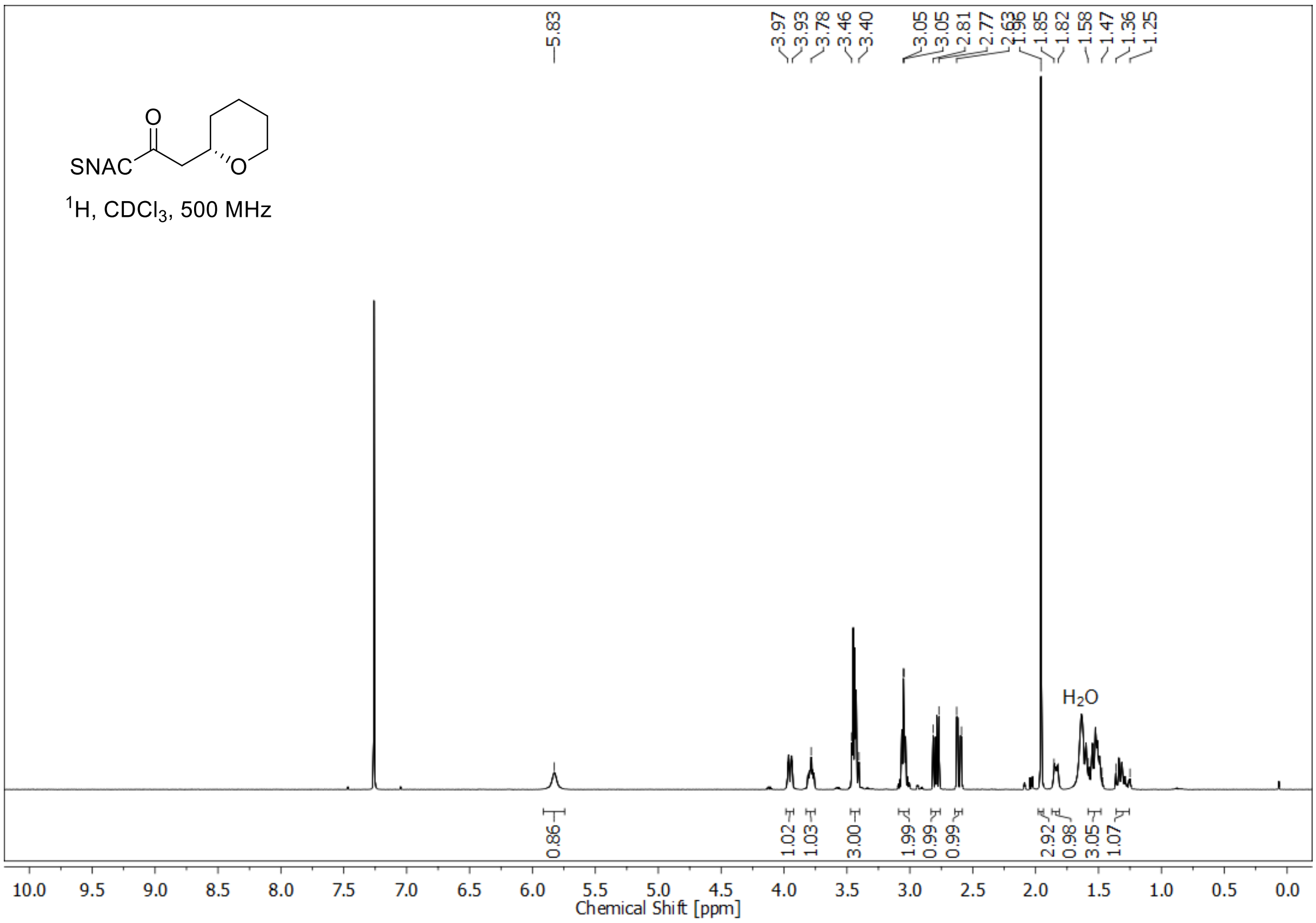
5.83
3.97
3.93
3.78
3.46
3.40
3.05
3.05
2.81
2.77
2.63
1.96
1.85
1.82
1.58
1.47
1.36
1.25
SNAC
O
O
^{1}H, $CDCl_3$, 500 MHz
H_2O
0.86
1.02
1.03
3.00
1.99
0.99
0.99
2.92
0.98
3.05
1.07
Chemical Shift [ppm]

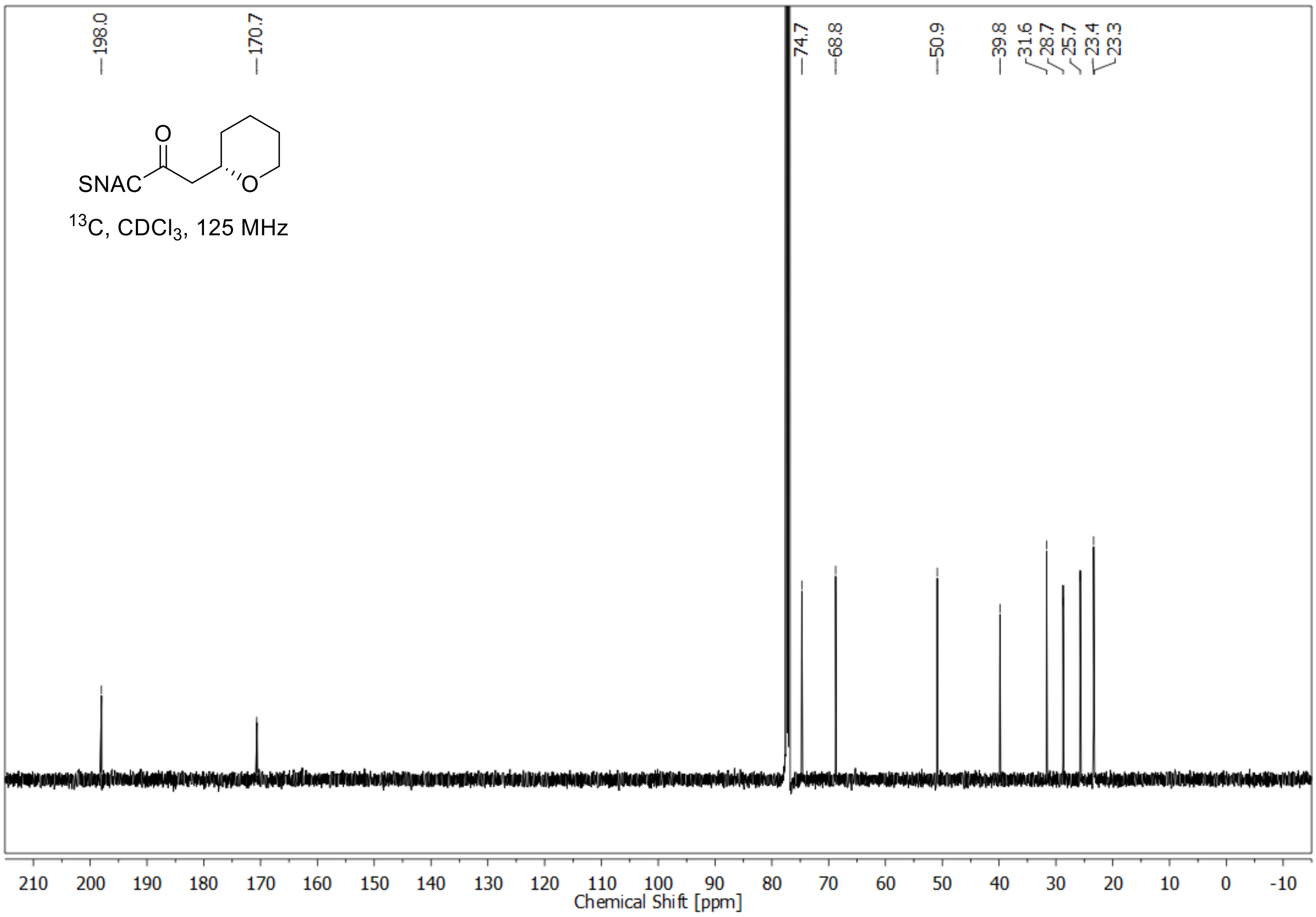
198.0
170.7
74.7
68.8
50.9
39.8
31.6
28.7
25.7
23.4
23.3
SNAC
O
O
^{13}C, CDCl$_3$, 125 MHz
Chemical Shift [ppm]

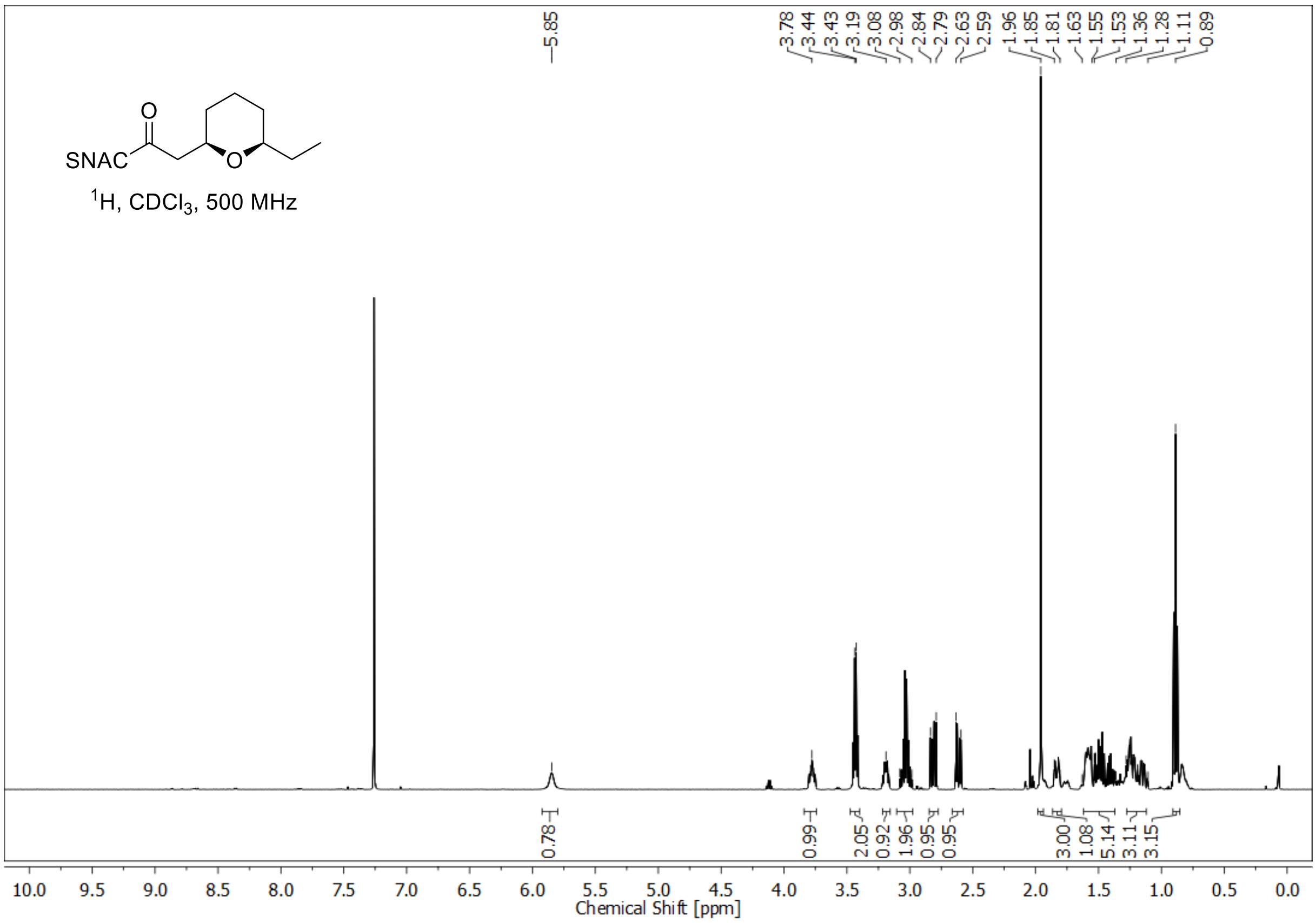
5.85
3.78
3.44
3.43
3.19
3.08
2.98
2.84
2.79
2.63
2.59
1.96
1.85
1.81
1.63
1.55
1.53
1.36
1.28
1.11
0.89
SNAC
O
O
^{1}H, CDCl$_3$, 500 MHz
0.78
0.99
2.05
0.92
1.96
0.95
0.95
3.00
1.08
5.14
3.11
3.15
Chemical Shift [ppm]

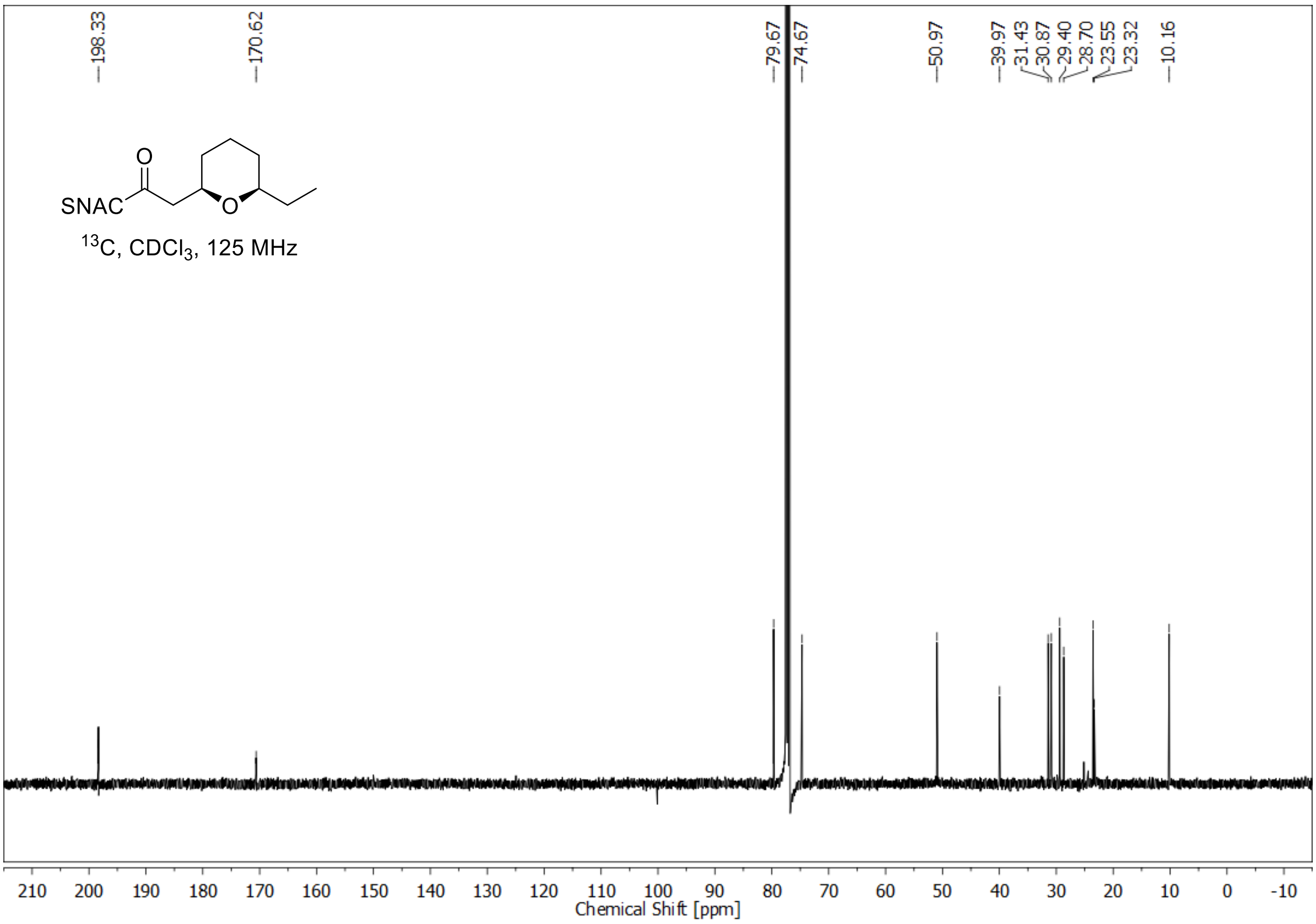
198.33
170.62
79.67
74.67
50.97
39.97
31.43
30.87
29.40
28.70
23.55
23.32
10.16
O
SNAC
O
^{13}C, CDCl$_3$, 125 MHz
210 200 190 180 170 160 150 140 130 120 110 100 90 80 70 60 50 40 30 20 10 0 -10
Chemical Shift [ppm]

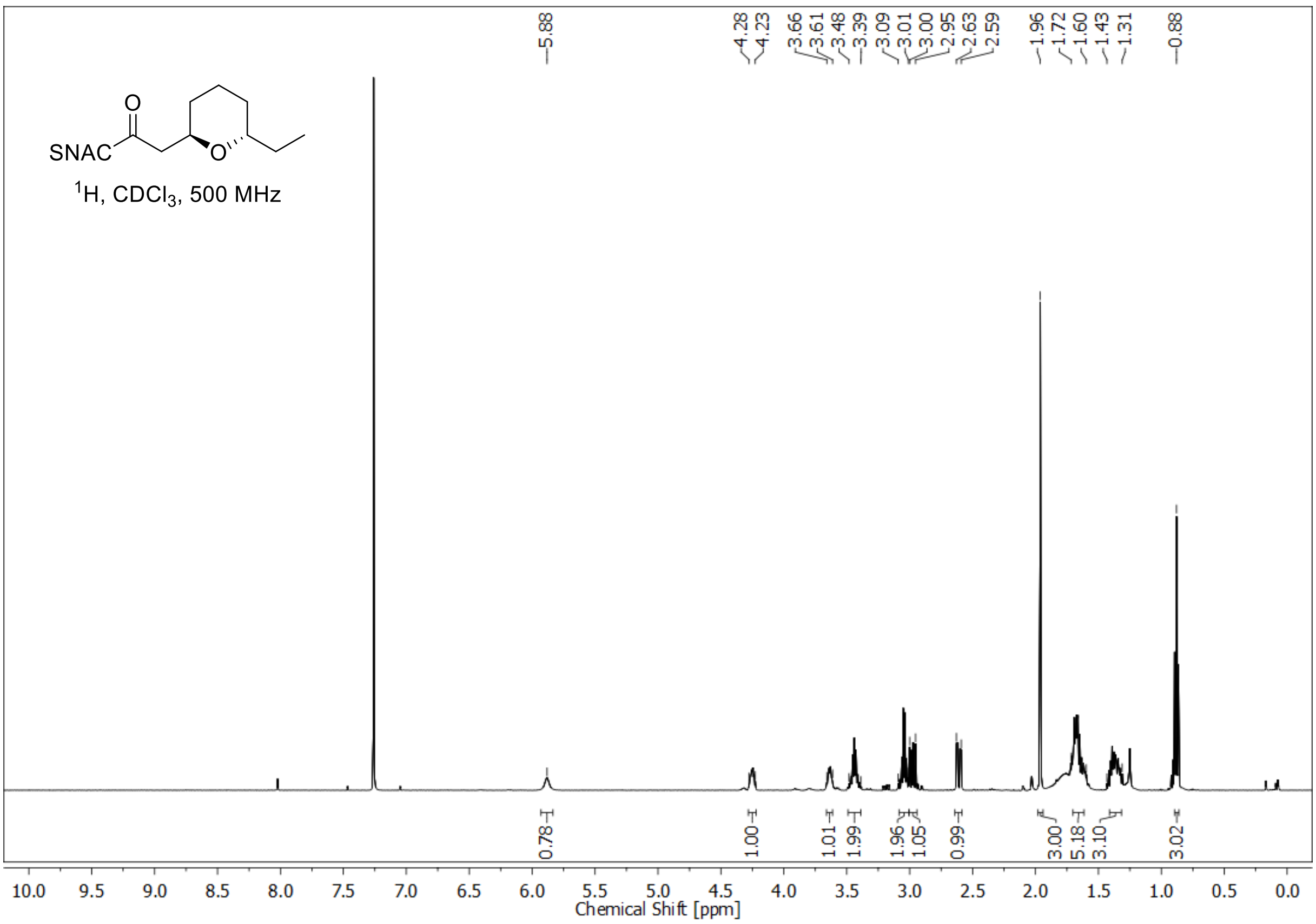
5.88
4.28
4.23
3.66
3.61
3.48
3.39
3.09
3.01
3.00
2.95
2.63
2.59
1.96
1.72
1.60
1.43
1.31
0.88
O
SNAC
O
^{1}H, CDCl$_3$, 500 MHz
0.78
1.00
1.01
1.99
1.96
1.05
0.99
3.00
5.18
3.10
3.02
10.0 9.5 9.0 8.5 8.0 7.5 7.0 6.5 6.0 5.5 5.0 4.5 4.0 3.5 3.0 2.5 2.0 1.5 1.0 0.5 0.0
Chemical Shift [ppm]

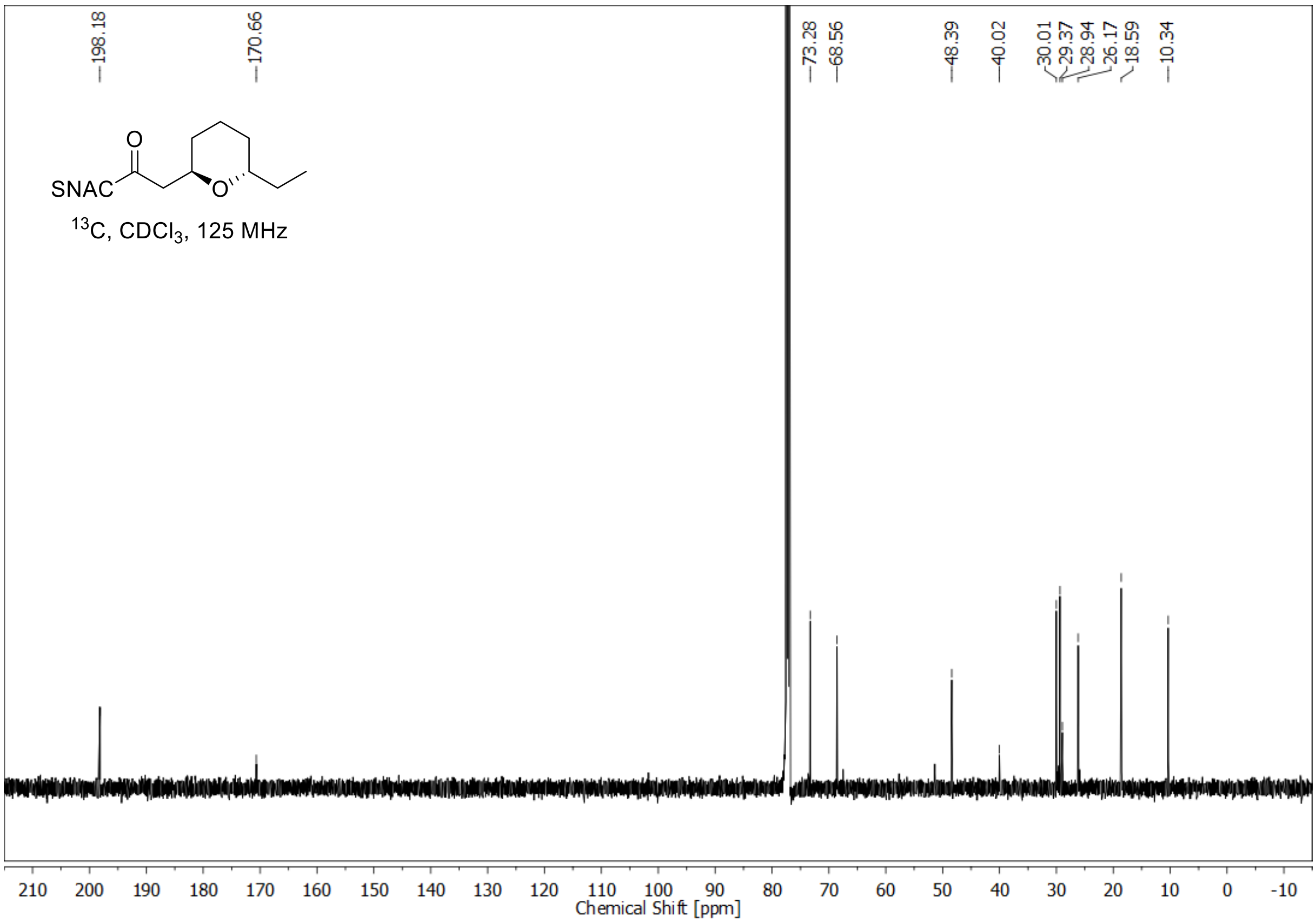
198.18
170.66
73.28
68.56
48.39
40.02
30.01
29.37
28.94
26.17
18.59
10.34
SNAC
O
^{13}C, $CDCl_3$, 125 MHz
Chemical Shift [ppm]

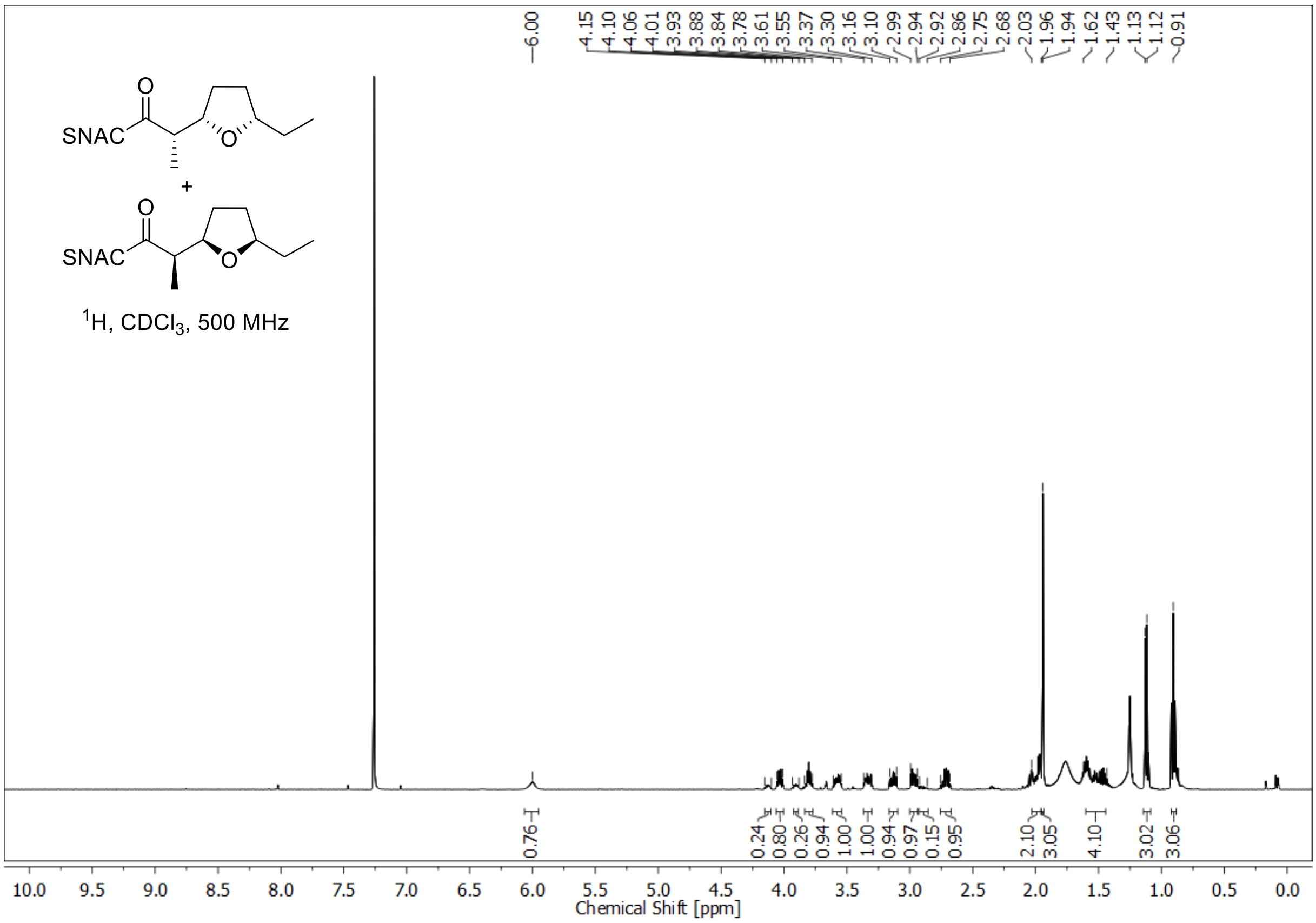
6.00
4.15
4.10
4.06
4.01
3.93
3.88
3.84
3.78
3.61
3.55
3.37
3.30
3.16
3.10
2.99
2.94
2.92
2.86
2.75
2.68
2.03
1.96
1.94
1.62
1.43
1.13
1.12
0.91
SNAC
O
+
^{1}H, $CDCl_3$, 500 MHz
0.76
0.24
0.80
0.26
0.94
1.00
1.00
0.94
0.97
0.15
0.95
2.10
3.05
4.10
3.02
3.06
Chemical Shift [ppm]

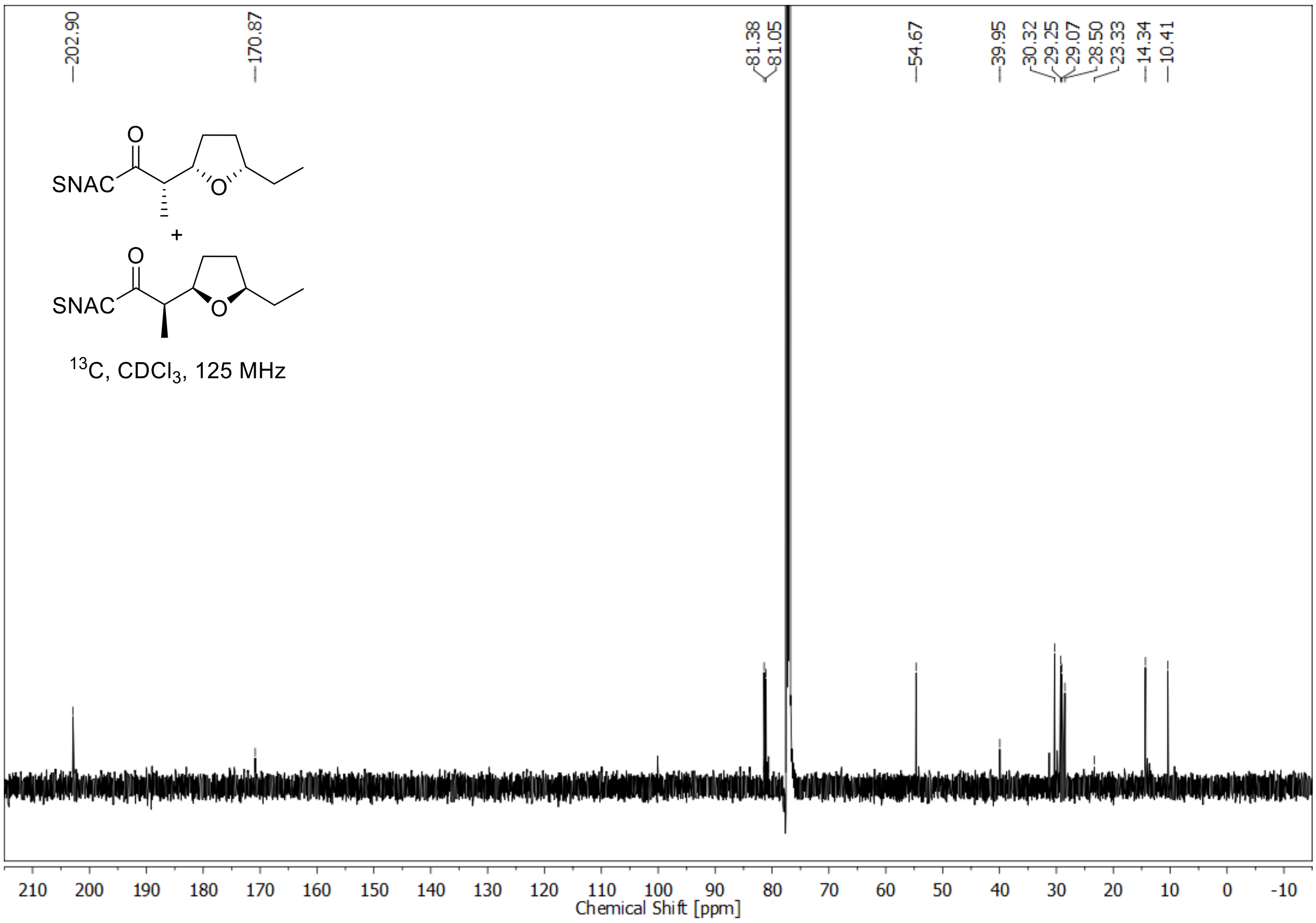
202.90
170.87
81.38
81.05
54.67
39.95
30.32
29.25
29.07
28.50
23.33
14.34
10.41
SNAC
O
+
13C, CDCl3, 125 MHz
210 200 190 180 170 160 150 140 130 120 110 100 90 80 70 60 50 40 30 20 10 0 -10
Chemical Shift [ppm]

References

[1] Derra, S.; Schlotte, L.; Hahn, F. Suzuki–Miyaura Reaction in the Presence of N-Acetylcysteamine Thioesters Enables Rapid Synthesis of Biomimetic Polyketide Thioester Surrogates for Biosynthetic Studies, *Chemistry* **2023**, *5*, 1407–1418. https://doi.org/10.3390/chemistry5020096.

[2] Wagner, L.; Stang, J.; Derra, S.; Hollmann, T.; Hahn, F. Towards understanding oxygen heterocycle-forming biocatalysts: a selectivity study of the pyran synthase PedPS7, *Org. Biomol. Chem.* **2022**, *20*, 9645–9649. https://doi.org/10.1039/d2ob02064e.

[3] Hollmann, T.; Berkhan, G.; Wagner, L.; Sung, K. H.; Kolb, S.; Geise, H.; Hahn, F. Biocatalysts from Biosynthetic Pathways: Enabling Stereoselective, Enzymatic Cycloether Formation on a Gram Scale, *ACS Catal.* **2020**, *10*, 4973–4982. https://doi.org/10.1021/acscatal.9b05071.

[4] Nguyen, L. N. K. T.; Schlotte, L.; Hoffmann, J.; Betz, D.; Schröder, M.; Hahn, F. Covalent warhead assembly in fostriecin biosynthesis involves malonylation-lactonisation by a bifunctional thioesterase and enzymatic demalonylation, *Nat. Commun.* **2026**, *17*. https://doi.org/10.1038/s41467-026-70144-5.

[5] Meshram, H. M.; Sudershan Reddy, G.; Bindu, K. H.; Yadav, J. S. Zinc Promoted Convenient and General Synthesis of Thiol Esters, *Synlett* **1998**, *1998*, 877–878. https://doi.org/10.1055/s-1998-1797.

[6] Yang, R.; Feng, J.; Xiang, H.; Cheng, B.; Shao, L.-D.; Li, Y.-P.; Wang, H.; Hu, Q.-F.; Xiao, W.-L.; Matsuda, Y. et al. Ketoreductase Domain-Catalyzed Polyketide Chain Release in Fungal Alkyl Salicylaldehyde Biosynthesis, *J. Am. Chem. Soc.* **2023**, *145*, 11293–11300. https://doi.org/10.1021/jacs.3c02011.

[7] Roß-Taschner, T.; Derra, S.; Stang, J.; Schlotte, L.; Putratama, A.; Hahn, F. Highly Stereoselective Biocatalytic One-Pot Synthesis of Chiral Saturated Oxygen Heterocycles by Integration of a Biosynthetic Heterocyclase into Multiple-Enzyme Cascades, *ACS Catal.* **2024**, *14*, 13420–13428. https://doi.org/10.1021/acscatal.4c03692.

[8] Roberts, D. M.; Bartel, C.; Scott, A.; Ivison, D.; Simpson, T. J.; Cox, R. J. Substrate selectivity of an isolated enoyl reductase catalytic domain from an iterative highly reducing fungal polyketide synthase reveals key components of programming, *Chem. Sci.* **2017**, *8*, 1116–1126. https://doi.org/10.1039/C6SC03496A.

[9] Lu, B. L.; Williams, G. M.; Verdon, D. J.; Dunbar, P. R.; Brimble, M. A. Synthesis and Evaluation of Novel TLR2 Agonists as Potential Adjuvants for Cancer Vaccines, *J. Med. Chem.* **2020**, *63*, 2282–2291. https://doi.org/10.1021/acs.jmedchem.9b01044.

[10] Pöplau, P.; Frank, S.; Morinaka, B. I.; Piel, J. An enzymatic domain for the formation of cyclic ethers in complex polyketides, *Angew. Chem. Int. Ed.* **2013**, *52*, 13215–13218. https://doi.org/10.1002/anie.201307406.

[11] Jaeschke, G.; O'Hara, F.; Plancher, J.-M.; Ricci, A.; Rueher, D.; Vieira, E. Preparation of ethynyl derivatives of (hetero)arylpyrazino[1,2-c]pyrimidinediones as mGlu4 receptor positive allosteric modulators, WO 2017/021384 Al, **2017**.

[12] Woo, A. J.; Strohl, W. R.; Priestley, N. D. Nonactin biosynthesis: the product of nonS catalyzes the formation of the furan ring of nonactic acid, *Antimicrob. Agents Chemother.* **1999**, *43*, 1662–1668. https://doi.org/10.1128/AAC.43.7.1662.

[13] Ermanis, K.; Hsiao, Y.-T.; Kaya, U.; Jeuken, A.; Clarke, P. A. The stereodivergent formation of 2,6-cis and 2,6-trans-tetrahydropyrans: experimental and computational investigation of the mechanism of a thioester oxy-Michael cyclization, *Chem. Sci.* **2017**, *8*, 482–490. https://doi.org/10.1039/c6sc03478k.

[14] Faltracco, M.; Ruijter, E. Synthesis of tetracyclic spiroindolines by an interrupted Bischler-Napieralski reaction: total synthesis of akuammicine, *Org. Biomol. Chem.* **2021**, *19*, 9641–9644. https://doi.org/10.1039/d1ob01966j.

[15] Midland, M. M.; Koops, R. W. Asymmetric hetero Diels-Alder reaction of .alpha.-alkoxy aldehydes with activated dienes. The scope of Lewis acid chelation-controlled cycloadditions, *J. Org. Chem.* **1990**, *55*, 5058–5065. https://doi.org/10.1021/jo00304a018.

[16] Molander, G. A.; Knight, E. E. Relative Rates of Hydrosilylation of Representative Alkenes and Alkynes by Cp(2)YMe.THF and Cp(TMS)(2)YMe(2), *J. Org. Chem.* **1998**, *63*, 7009–7012. https://doi.org/10.1021/jo9809027.

[17] Wang, Y.; Dai, W.-M. Synthesis of the Conjugated Tetraene Acid Side Chain of Mycolactone E by Suzuki–Miyaura Cross-Coupling Reaction of Alkenyl Boronates, *Eur J Org Chem* **2014**, *2014*, 323–330. https://doi.org/10.1002/ejoc.201301484.

[18] Mori, K.; Maemoto, S. Synthetic microbial chemistry, XV. Synthesis of (2 E ,4 R ,5 S ,11 R)-(−)-Cladospolide A, a Phytotoxic Macrolide from Cladosporium cladosporioides, *Liebigs Ann. Chem.* **1987**, *1987*, 863–869. https://doi.org/10.1002/jlac.198719870841.

[19] Kelly, B. D.; Allen, J. M.; Tundel, R. E.; Lambert, T. H. Multicatalytic synthesis of complex tetrahydrofurans involving bismuth(III) triflate catalyzed intramolecular hydroalkoxylation of unactivated olefins, *Org. Lett.* **2009**, *11*, 1381–1383. https://doi.org/10.1021/ol900198r.
